# Evolutionarily diverse caveolins share a common structural framework built around amphipathic disks

**DOI:** 10.1083/jcb.202411175

**Published:** 2025-08-07

**Authors:** Bing Han, Sarah M. Connolly, Darrin T. Schultz, Louis F.L. Wilson, Alican Gulsevin, Jens Meiler, Erkan Karakas, Melanie D. Ohi, Anne K. Kenworthy

**Affiliations:** 1 Center for Membrane and Cell Physiology, University of Virginia, Charlottesville, VA, USA; 2 Department of Molecular Physiology and Biological Physics, University of Virginia School of Medicine, Charlottesville, VA, USA; 3 Life Sciences Institute, University of Michigan, Ann Arbor, MI, USA; 4 Department of Neuroscience and Developmental Biology, University of Vienna, Vienna, Austria; 5 Howard Hughes Medical Institute, University of Virginia School of Medicine, Charlottesville, VA, USA; 6 Department of Chemistry, Vanderbilt University, Nashville, TN, USA; 7 Institute for Drug Discovery, Leipzig University, Leipzig, Germany; 8 Department of Molecular Physiology and Biophysics, Vanderbilt University, Nashville, TN, USA; 9 Department of Cell and Developmental Biology, University of Michigan, Ann Arbor, MI, USA

## Abstract

Caveolins are a unique family of membrane remodeling proteins present broadly across animals (Metazoa), and in vertebrates form flask-shaped invaginations known as caveolae. While human caveolin-1 assembles into an amphipathic disk composed of 11 spirally packed protomers, the structural basis underlying caveolin function across animals remains elusive. Here, we predicted structures for 73 caveolins spanning animal diversity, as well as a newly identified choanoflagellate caveolin from *Salpingoeca rosetta*. This analysis revealed seven conserved structural elements and a propensity to assemble into amphipathic disks. Cryo-EM structures of caveolins from *S. rosetta* choanoflagellate and the purple sea urchin *Strongylocentrotus purpuratus* exhibit striking structural similarities to human caveolin-1, validating the structural predictions. Lastly, tracing the chromosomal evolutionary history of caveolins revealed its parahoxozoan ancestral chromosome and evolutionary branches on which caveolins translocated and expanded. These results show that caveolins possess an ancient structural framework predating Metazoa and provide a new structural paradigm to explore the molecular basis of caveolin function across diverse evolutionary lineages.

## Teaser

This study explores the evolutionary history of caveolins, a family of unusual membrane remodeling proteins. Using computational predictions and cryo-electron microscopy, the authors show both animal and choanoflagellate caveolins assemble into amphipathic disks composed of seven conserved structural elements.

## Introduction

Eukaryotes contain elaborate endomembrane systems consisting of morphologically and functionally distinct membrane-bound compartments. The construction and maintenance of these compartments relies on the actions of ancient families of proteins capable of remodeling membranes (Attwood et al., 2017; Field et al., 2007). To support enhanced requirements for cell–cell adhesion, communication, and signaling during the transition from single-celled eukaryotes to animals (Metazoa), a dramatic expansion in membrane-associated proteins occurred (Attwood et al., 2017). Among the membrane proteins thought to have first emerged in Metazoa is the caveolin family of membrane remodeling proteins (Attwood et al., 2017; Field et al., 2007; Kirkham et al., 2008). Best recognized for their role in vertebrates as structural components of caveolae, flask-shaped invaginations of the plasma membrane, caveolins have been identified in most metazoan clades (Field et al., 2007; Kirkham et al., 2008; Spisni et al., 2005). Thus, they have fulfilled essential biological roles since the ancestor of the clade existed ∼800 million years ago (Strassert et al., 2021). In humans, caveolins and caveolae are distributed throughout the body serving as important regulators of multiple organ systems (Bastiani and Parton, 2010; Chidlow and Sessa, 2010; Parton, 2018; Williams and Lisanti, 2004). Furthermore, caveolins and caveolae are broadly implicated in regulation of cell signaling, lipid metabolism, and sensing and responding to stress (Del Pozo et al., 2020; Lamaze et al., 2017; Nassoy and Lamaze, 2012; Parton, 2018; Parton and del Pozo, 2013).

Unlike vesicle coat proteins such as clathrin, COPI, and COPII that cycle on and off membranes and share evolutionary origins and structural features (Dacks and Robinson, 2017), caveolins are unrelated in sequence to other vesicle coat proteins and remain integrated in membranes throughout their life cycle. Most studies of caveolins have focused on their roles in mammalian cells where caveolae are often abundant (Parton et al., 2020b; Parton et al., 2021; Razani et al., 2002b). However, in some cell types lacking cavins, a second family of proteins required for caveolae biogenesis, caveolins can function independently of caveolae (Ariotti and Parton, 2013; Head and Insel, 2007; Kovtun et al., 2015; Pol et al., 2020). Since the cavin family appears to be found only in vertebrates (Hansen and Nichols, 2010), this suggests that in most organisms caveolins function independently of classically defined caveolae. To date, however, only a handful of examples of caveolins from non-vertebrate organisms have been studied (Bhattachan et al., 2020; Frank and Lisanti, 2006; Kirkham et al., 2008; Razani et al., 2002a; Tang et al., 1997). It is also unclear whether caveolins exist in ctenophores, the sister clade to all other animals (Schultz et al., 2023), and whether caveolins’ provenance can be traced back to known ancestral chromosomes (Simakov et al., 2022). Thus, our knowledge of the existence and functions of caveolins across evolutionary space is limited.

The molecular architecture of caveolins was unknown until the discovery of the structure of the homo-oligomeric 8S complex of human caveolin-1 (Cav1), an essential component of caveolae in non-muscle cells (Ohi and Kenworthy, 2022; Porta et al., 2022). Cryo-electron microscopy (cryo-EM) showed the complex is composed of 11 Cav1 protomers symmetrically arranged into an unusual amphipathic disklike structure predicted to fully insert into the cytoplasmic leaflet of the plasma membrane (Doktorova et al., 2025; Ohi and Kenworthy, 2022; Porta et al., 2022). However, whether related proteins from other organisms (including distantly related caveolin homologs) behave in the same way is completely unknown.

Here, we report that in addition to being expressed in metazoans, caveolin homologs exist in choanoflagellates, free-swimming unicellular protists, and the closest relatives of animals, suggesting that caveolins are of pre-metazoan and pre-choanozoan origin. We also find that ctenophores lack caveolins and that most of caveolin diversity in animals can be traced to a single ancestral chromosome in the ancestor of the Parahoxozoa. Using a combination of computational, phylogenetic, and structural approaches, we show that despite extreme sequence variability, even the most distantly related caveolin homologs share a surprisingly conserved set of structural elements. These findings suggest that caveolins share an ancient and conserved structural framework that diverse organisms co-opted to fulfill distinct physiological roles. They also provide a new framework to probe the structural basis for the function of caveolins across evolution.

## Results

### Consistent evidence for the existence of choanoflagellate caveolins

Caveolins have typically been treated as a metazoan-specific family (Attwood et al., 2017; Echarri and Del Pozo, 2012; Field et al., 2007; Kirkham et al., 2008). However, one sequence located on chromosome 10 in the choanoflagellate *Salpingoeca rosetta* (strain: ATCC 50818, genome assembly GCA_033442325.1) is currently annotated in the UniProt database (RRID:SCR_002380) as a caveolin (UniProt: F2U793). This sequence shares almost no recognizable sequence identity with human Cav1 (13%); however, if truly related, it would represent the most evolutionarily divergent caveolin found to date. To verify this annotation, we built a caveolin hidden Markov model (HMM) profile from an alignment based on a previously identified region of high conservation (Kirkham et al., 2008). Encouragingly, HMMER searches against the *S. rosetta* proteomes retrieved the F2U793 sequences with an E-value of 8.8 × 10^−11^, indicating a confident prediction of the homology. In contrast, a sequence from the filasterean protist *Capsaspora owczarzaki* (strain: ATCC 30864) annotated in UniProt as a caveolin (A0A0D2VH37) yielded an E-value >0.05, suggesting this annotation is spurious. Moreover, genomic frame-shifted HMM profile searches of the *C. owczarzaki* and ichthyosporean *Creolimax fragrantissima* genomes (de Mendoza et al., 2015; Schultz et al., 2023; Suga et al., 2013) did not yield identifiable caveolin proteins (E-value >0.05). These findings support the idea that caveolins are in fact not limited to Metazoa but are also present in some choanoflagellates, the closest relatives of Metazoa.

### Ancestral linkage group analysis reveals caveolin chromosomal origins

The unexpected finding of a putative caveolin in *S. rosetta* led us to revisit the evolutionary history of caveolins. Current evidence suggests all vertebrate caveolins descend from three ancestral sequences: CavX, CavY, and CavZ. CavX and CavZ were colocalized to the same chromosome in the ancestral vertebrate genome (Kirkham et al., 2008). Whereas CavY appears to have been lost in most vertebrates, CavX is proposed to have given rise to Cav1 and Cav3, the “canonical” caveola-forming family members, which also have closely related homologs in cnidarians (Kirkham et al., 2008). CavZ appears to have given rise to the Cav2 family (Kirkham et al., 2008).

To trace the chromosomal origins of caveolins and their emergence in animals, we analyzed ancestral linkage groups (ALGs) to identify the chromosome on which caveolin originated. ALGs are collections of sequences representing whole or partial ancestral chromosomes and provide a framework for reconstructing genome evolution. There were 29 ALGs in the ancestor of myriazoans (all animals except ctenophores) that remain highly conserved in several animal clades (Simakov et al., 2022), with some partially preserved on single chromosomes in the choanoflagellate *S. rosetta* (Schultz et al., 2023).

**Figure 1.**
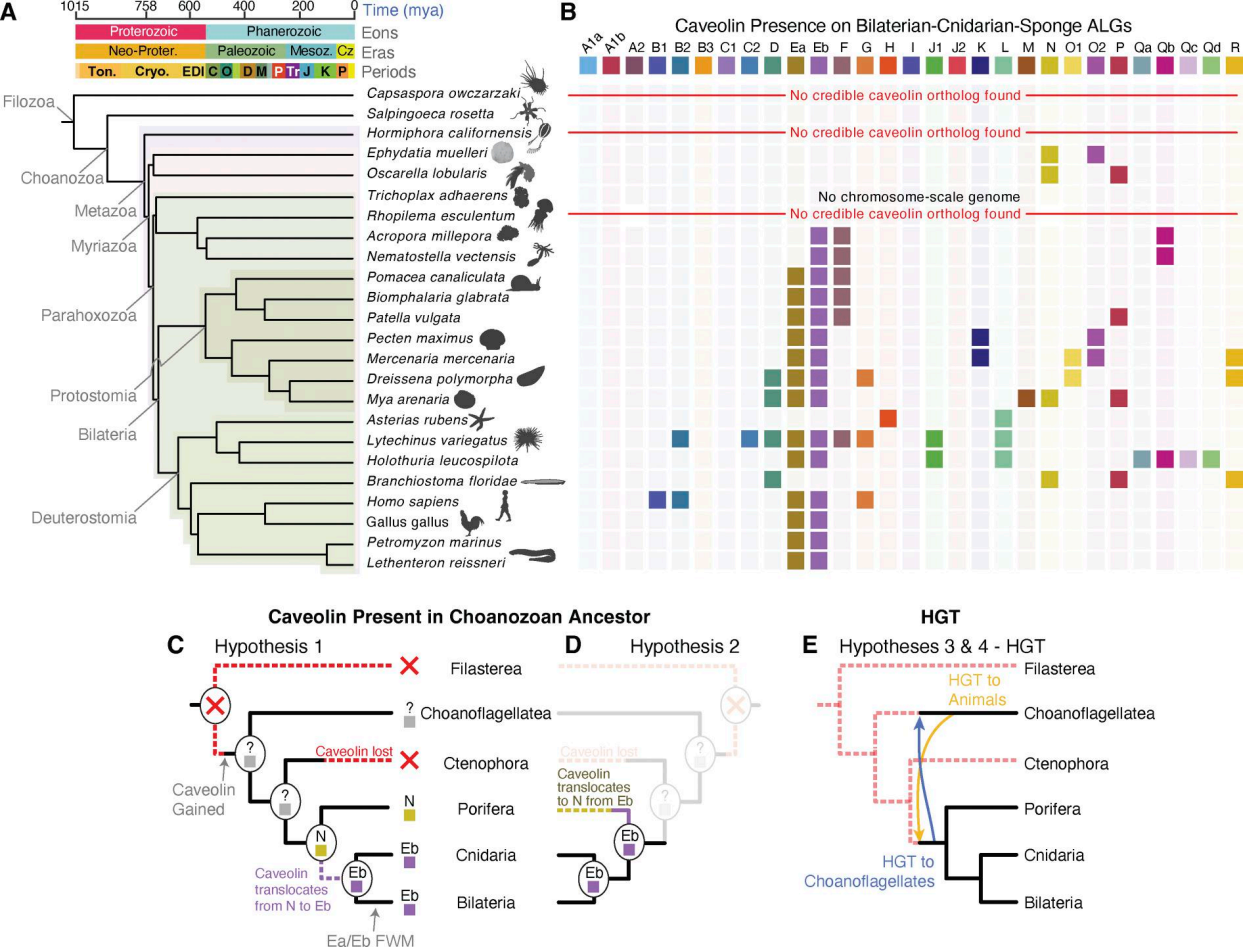
**Chromosomal origins of caveolin. (A)** Samples across animal diversity and in two unicellular relatives of animals were used to determine the ALG identities of chromosomes on which caveolins were present. **(B)** Caveolins are present on BCnS ALG Eb-bearing chromosomes in all but two species observed within the Parahoxozoa. In sponges, caveolins are present on ALG N in species from two anciently diverged sponge clades. The BCnS ALG identity of *S. rosetta* chromosome 10, on which caveolin is present, does not have a significant ALG identity. **(C–E)** Possible models for the chromosomal origins of caveolin. **(C and D)** Assuming that caveolin was present in the choanozoan ancestor, it must have been lost in the ctenophore lineage. Caveolin’s chromosomal provenance is parsimoniously equally likely to have been ALG N, followed by translocation to ALG Eb in the parahoxozoan ancestor (C), or ALG Eb in the ancestral myriazoan genome, followed by translocation to ALG N in the common ancestor of Demospongiae and Homoscleromorpha sponges. **(E)** Another potential explanation of caveolin’s provenance that accounts for its presence in both animals and choanoflagellates is HGT. This could have occurred in either direction after acquisition of the gene (i.e., from choanoflagellates to animals, or from animals to choanoflagellates). BCnS, bilaterian–cnidarian–sponge; HGT, horizontal gene transfer.

We analyzed chromosome-scale genomes spanning animal diversity that also have minimal chromosomal changes since their divergence from the myriazoan ancestor (Schultz et al., 2024, *Preprint*) (Fig. 1 A and Data S1). Using these genomes, we identified caveolin orthologs and identified the myriazoan ALGs associated with those chromosomes. No credible caveolin orthologs were found in the filasterean amoeba *Capsaspora owczarzaki*, ctenophores, or scyphozoan and hydrozoan cnidarians (Fig. 1 B). However, caveolins or caveolin-like genes were present in the chromosome-scale genomes of two distantly related sponges (∼400 million year divergence) and in anthozoan cnidarians (corals, anemones) (Fig. 1 B). Consistent with previous results (Kirkham et al., 2008), we found multiple caveolin paralogs in Parahoxozoa (Bilateria, Placozoa, and Cnidaria) (Fig. 1 B).

Using the myriazoan ALGs (Simakov et al., 2022), we identified a pattern of caveolin presence on ALG Eb-bearing chromosomes within the Parahoxozoa (Fig. 1 B). Exceptions include the lancelet *Branchiostoma floridae* and the sea star *Asterias rubens*, where caveolins are absent on Eb-bearing chromosomes (Fig. 1 B). In cnidarians, ALG Ea and Eb remain on separate chromosomes (de Mendoza et al., 2015), and we found putative caveolins on Eb-bearing chromosomes in two species that diverged 300–600 million years ago (dos Reis et al., 2015). Since ALGs Ea and Eb were separate in the ancestor of Parahoxozoa but fused before the bilaterian ancestor (de Mendoza et al., 2015), these results suggest that caveolin was ancestrally present on ALG Eb in the ancestor of Parahoxozoa (Fig. 1 B). Additionally, in sponges, we found caveolin-like sequences on ALG N-bearing chromosomes in two distantly related species (∼500 million years divergence) (Gold et al., 2015) (Fig. 1 B).

These findings suggest two equally parsimonious scenarios for caveolin’s chromosomal origins in myriazoans (Fig. 1, C and D). In one scenario, caveolin originated on ALG Eb in the myriazoan ancestor and later translocated to ALG N in sponges. In the other, caveolin originated on ALG N in the myriazoan ancestor and translocated to ALG Eb in the lineage leading to Parahoxozoa. Due to caveolin’s absence in ctenophores and the extensive rearrangements between animal and choanoflagellate genomes, we are not able to determine the chromosome on which caveolin originated in the animal ancestor, or whether caveolin was present in the Choanozoa ancestor. We also cannot rule out the possibility of horizontal gene transfer between the ancestors of myriazoans and choanoflagellates as an explanation of its origins in either clade (Fig. 1 E). Phylogenetic trees generated in the next section did not clarify these possibilities.

### Phylogenetic analysis provides further insights into the evolutionary history of caveolins

To better understand caveolin evolution, we conducted an updated phylogenetic analysis incorporating newly sequenced metazoan genomes (Kirkham et al., 2008). While genome data exist for over 3,200 animal species, vertebrates—despite comprising only 3.9% of species—account for >50% of sequenced genomes (Hotaling et al., 2021) potentially skewing a phylogenetic analysis. To gain a more balanced and comprehensive view of how caveolin evolved across different evolutionary branches, we selected caveolins from one representative species per metazoan phylum or superphylum of Metazoa, along with the previously identified *S. rosetta* caveolin.

For this analysis, we included caveolins from the sponge *Amphimedon queenslandica* (Porifera, Metazoa), a species of evolutionary interest due to its early divergence from the lineage leading to vertebrates (Fairclough et al., 2013; Srivastava et al., 2010). In the *A. queenslandica* reference proteome, we found five proteins with caveolin Pfam annotations (UniProt: A0A1X7UHP5, A0A1X7UGA1, A0A1X7TMH4, A0A1X7VPY7, and A0A1X7VRV8; note that Pfam data and new releases are available through InterPro [http://pfam.xfam.org/]). As with the *S. rosetta* sequence, the *A. queenslandica* sequences were highly dissimilar to human Cav1 (11–19% identity). A HMMER search against the *A. queenslandica* genome yielded significant hits (E-values: 5.1 × 10^−15^ to 2.5 × 10^−11^), suggesting that these putative sponge caveolins may be true homologs to bilaterian caveolins.

**Figure S1.**
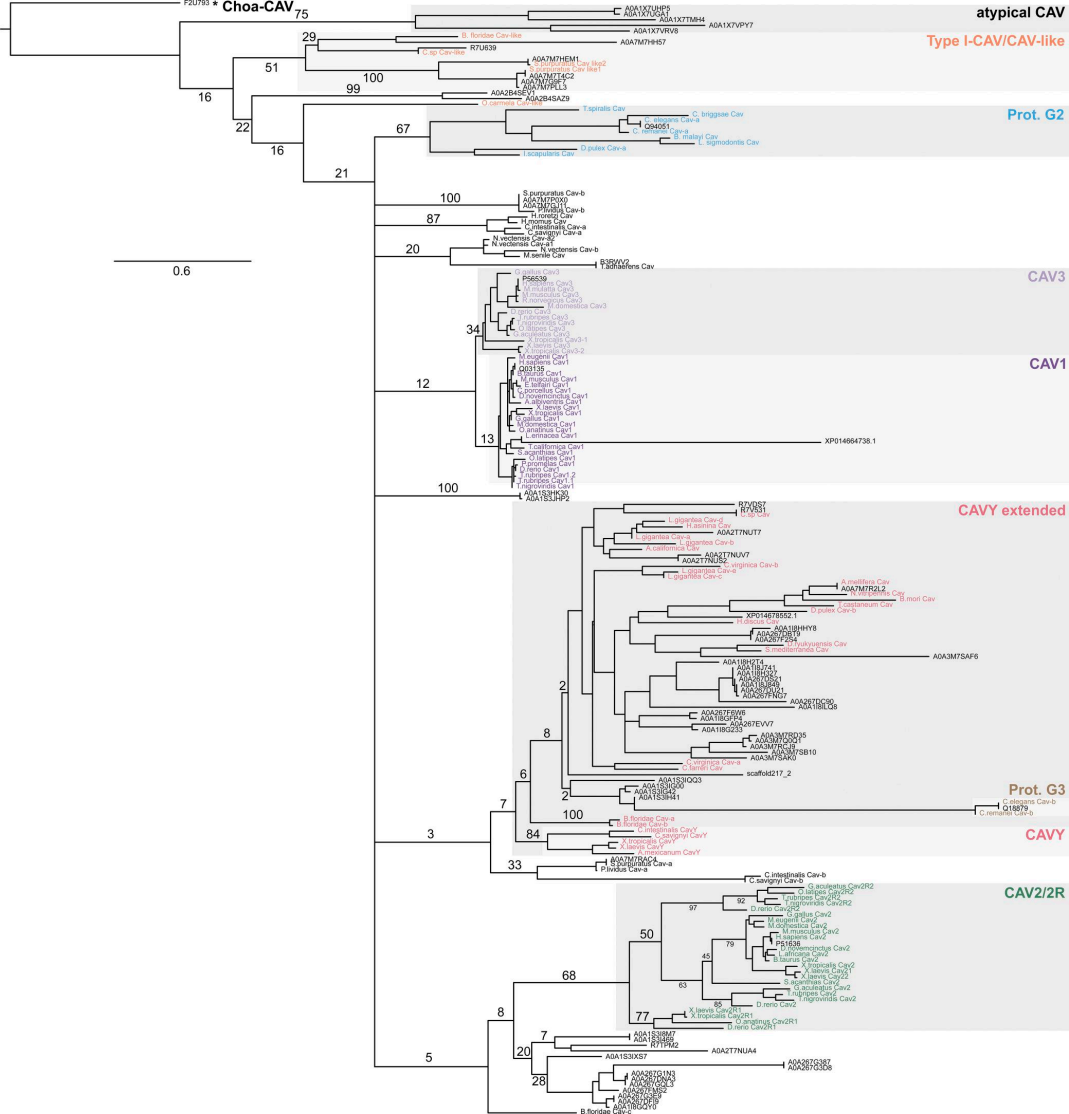
**Phylogenetic relationships of caveolin sequences inferred from a maximum-likelihood phylogeny.** A maximum-likelihood phylogeny was inferred for representative caveolins from the current study (black) in combination with caveolin sequences previously analyzed by Kirkham et al. (2008). Previously analyzed caveolins are color-coded according to their classifications. Rooting was performed under the assumption that the choanoflagellate sequence constitutes an outgroup. Support values (percent replication in 1,000 rapid bootstrap pseudoreplicates) are shown for the major splits. Branch lengths are proportional to the average number of substitutions per site (refer to scale). Splits denoting the higher order relationship between the apparent Prot. G2, CAV3, CAV1, CAVY (ext.), Prot. G3, and CAV2/2R clades received extremely low bootstrap support and are therefore represented by a polytomy in the final tree. The asterisk, *, indicates a caveolin-related protein from *S. rosetta*.

**Figure S2.**
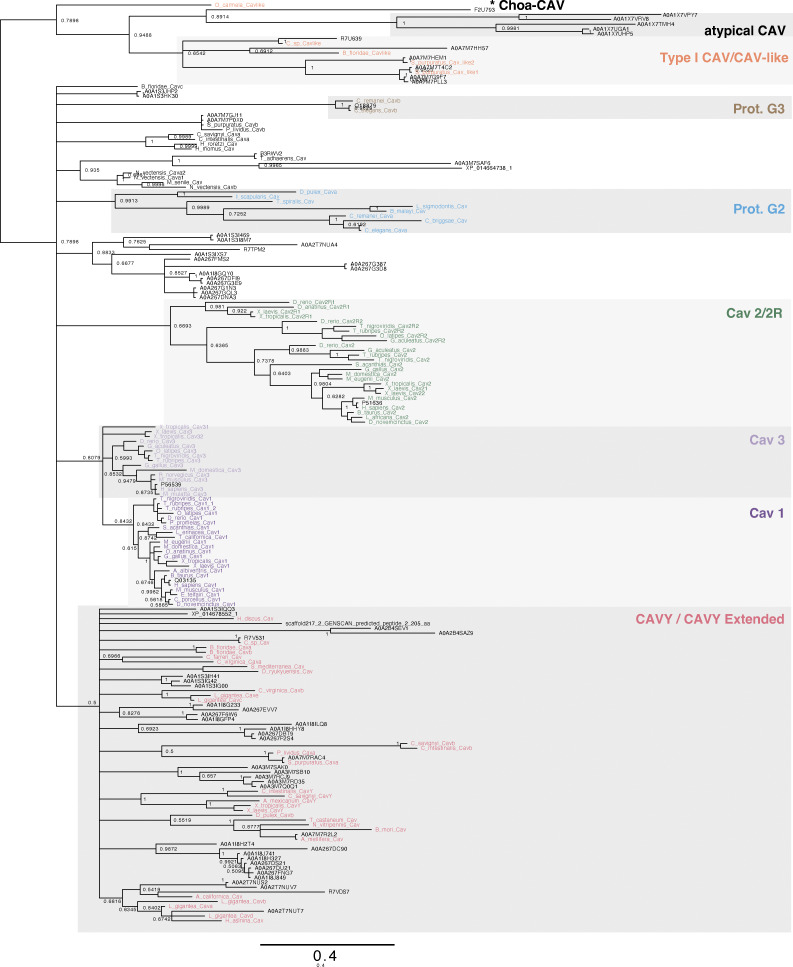
**Phylogenetic relationships of caveolin sequences inferred from an unrooted Bayesian tree.** An unrooted Bayesian tree inferred from the alignment used for the tree in Fig. S1. No columns were removed from the alignment using GUIDANCE. Posterior probabilities are shown next to their respective nodes. Sequences are colored in the same manner as in Fig. S1. The asterisk, *, indicates a caveolin-related protein from *S. rosetta*.

Next, we inferred maximum-likelihood and Bayesian phylogenies using 74 protein sequences with caveolin Pfam annotations from 15 distantly related holozoans (Data S2), along with a set of previously categorized caveolin and caveolin-like sequences (Kirkham et al., 2008). The resulting gene trees largely replicated, albeit with only moderate support values, the previously proposed clades (Kirkham et al., 2008), consisting of Cav1/3, Cav2/2R, CavY extended, Protostomia Group 1, Protostomia Group 2, and CAV-like (Figs. S1 and S2). However, there was poor support resolving the evolutionary history between these groups, which together form a polytomy in each tree. Many of the sequences of interest could be tentatively assigned to one of these previously defined groups (Figs. S1 and S2). However, the caveolin homologs from the sponge *A. queenslandica* formed their own monophyletic clade outside of the known clades. These results suggest an early divergence of sponge caveolins from those found in Parahoxozoa, accurately reflecting the species’ history despite the polytomies in the caveolin gene trees.

Given the long branch length connecting the *S. rosetta* sequence to the rest of the tree and the known evolutionary relationship between choanoflagellates and Metazoa, we designated this sequence as a provisional outgroup, named Choa-CAV (Fig. S1). We chose to designate the clade containing caveolin homologs from *A. queenslandica* group as “atypical caveolins” and the remaining groups as “typical caveolins”. We further broke down the typical caveolins into Type I-CAV and Type II-CAV. Almost all the relatively well-studied caveolins, such as human Cav1, Cav2, and Cav3, as well as *Caenorhabditis elegans* caveolins and *Apis mellifera* caveolin, belong to Type II-CAV. Type I-CAV corresponds to the CAV-like clade identified in a previous study (Kirkham et al., 2008). In the following sections, we will use this newly defined framework to trace the evolutionary trajectory and compare structural similarities and differences among caveolins.

### Caveolin protomers are predicted to organize into disk-shaped complexes composed of spiraling amphipathic α-helices

The existence of distantly related caveolin homologs with limited sequence similarity such as Choa caveolin raises the question as to whether they have similar folds and/or functions to human Cav1 (Porta et al., 2022). To investigate whether other metazoan caveolins and the newly identified choanoflagellate caveolin share similar structural features, we used AlphaFold2 (AF2) as a predictive tool (Jumper et al., 2021). Despite the unusual features of human Cav1, AF2 is able to predict its overall fold and reproduce its ability to oligomerize to form an amphipathic, disk-shaped structure characteristic of the Cav1 8S complex (Gulsevin et al., 2023).

**Figure 2.**
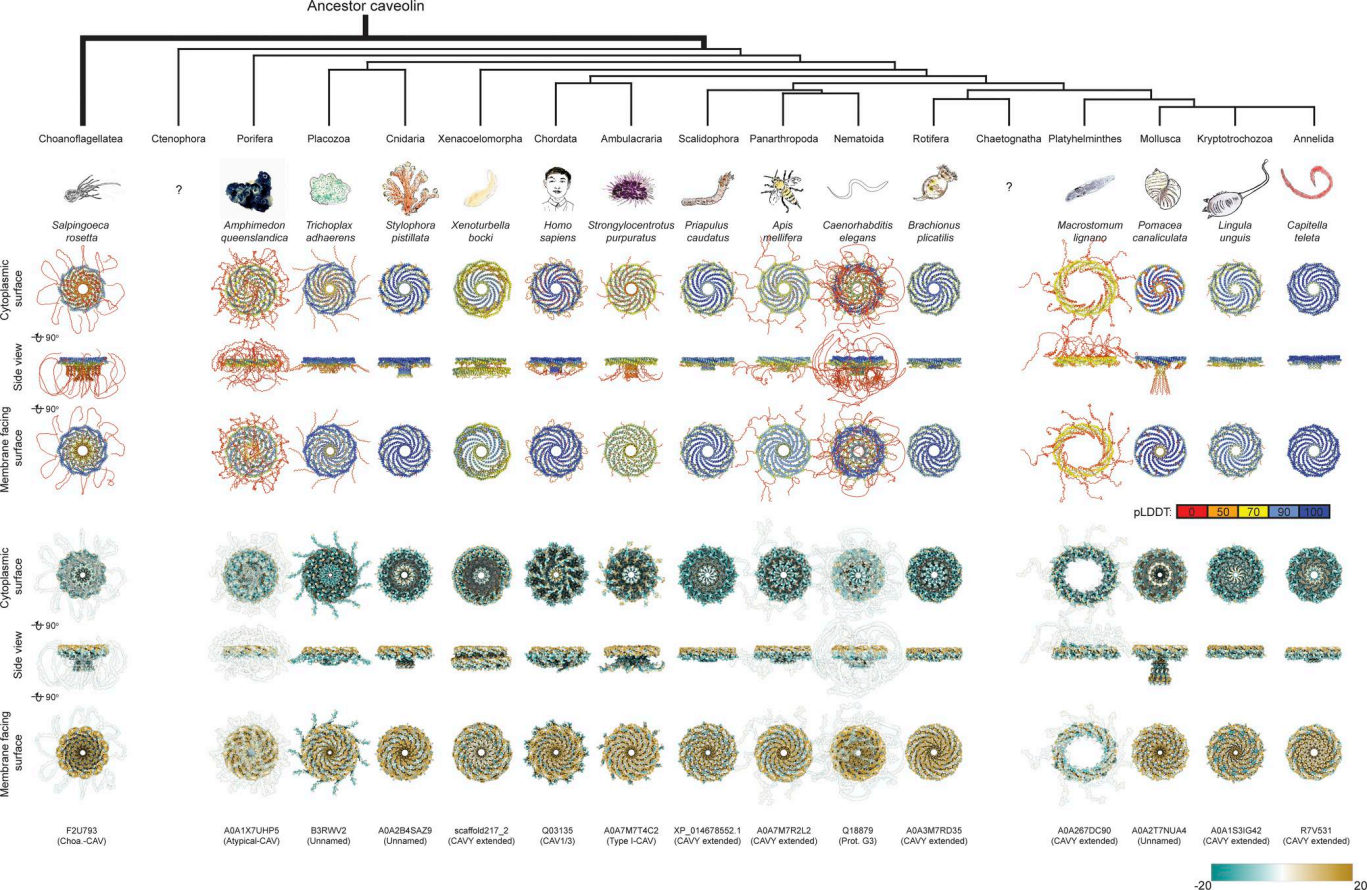
**Conserved structural features of metazoan and choanoflagellate caveolins highlighted by computational modeling predictions.** AF2.2 models for 11-mer caveolins from representative species of Choanoflagellatea and 14 different metazoan phyla/superphyla. Models in top three rows are in ribbon mode and colored by pLDDT confidence values. Models in bottom three rows are in surface mode and colored by lipophilicity values. To better demonstrate the distribution pattern of hydrophobicity in surface mode, we applied transparency to the N terminus of F2U793, A0A1X7UHP5, A0A7M7R2L2, Q18879, and the residues near the C terminus of A0A267DC90.

We predicted the structures of 74 Pfam-annotated caveolin family members from the representative species of each phylum or superphylum of Metazoa, as well as the Choa caveolin. A single example from each species is shown in Fig. 2. For our initial analysis, we generated *n*-mers of increasing size with AF2.1 (Data S3), and monomers, dimers, and 11-mers with AF2.2 (Data S4). In essentially all the examined species, the caveolins were predicted to form closely packed amphipathic disks or rings that spiral in a clockwise direction when viewed from the cytoplasmic face (Fig. 2). Most were also predicted to contain N-terminal disordered regions located around the outer rim of the complex and central β-barrels formed by parallel β-strands, similar to the structure of human Cav1. Interestingly, C-terminal disordered regions emanating from the central β-barrel were also visible in several caveolins (Fig. 2). Similar results were obtained when we used AF2.2 to predict the structure of the caveolins used in the ALG analysis, which included five metazoan phyla (Porifera, Cnidaria, Mollusca, Ambulacraria, and Chordata) (Data S4). In contrast, AF2.2 predicts that the sequence A0A0D2VH37 from *Capsaspora owczarzaki* forms a structure lacking signature caveolin structural elements (Data S4), consistent with the conclusions drawn from the sequence alignment.

While this study was underway, a new AlphaFold model, AlphaFold3 (AF3), was released (Abramson et al., 2024). To test whether AF3 yields different results, we used it to predict structures of monomers, dimers, and 11-mers for a subset of caveolins (Data S5). The AF2.1, AF2.2, and AF3.0 models for monomers, dimers, and oligomers are directly compared in Data S6. AF2.1, AF2.2, and AF3.0 all predict the helical disk–shaped assembly and corresponding secondary structures of caveolin oligomers. However, the details and confidence of the structures varied, especially for caveolin dimers. Furthermore, AF3 tended to predict that the intrinsically disordered N-terminal regions would have more secondary structural organization than suggested by AF2.2.

Together, these results suggest that even in the most distantly related species, caveolins can homo-oligomerize into amphipathic disks and contain similar structural elements as human Cav1. They also highlight the presence of additional structural elements such as a C-terminal variable region in some caveolins. For simplicity, in the subsequent analyses, we will focus on results obtained from caveolins obtained from the set of 74 Pfam-annotated caveolins as predicted using AF2.2.

### Caveolins across evolution consist of seven basic structural units

**Figure 3.**
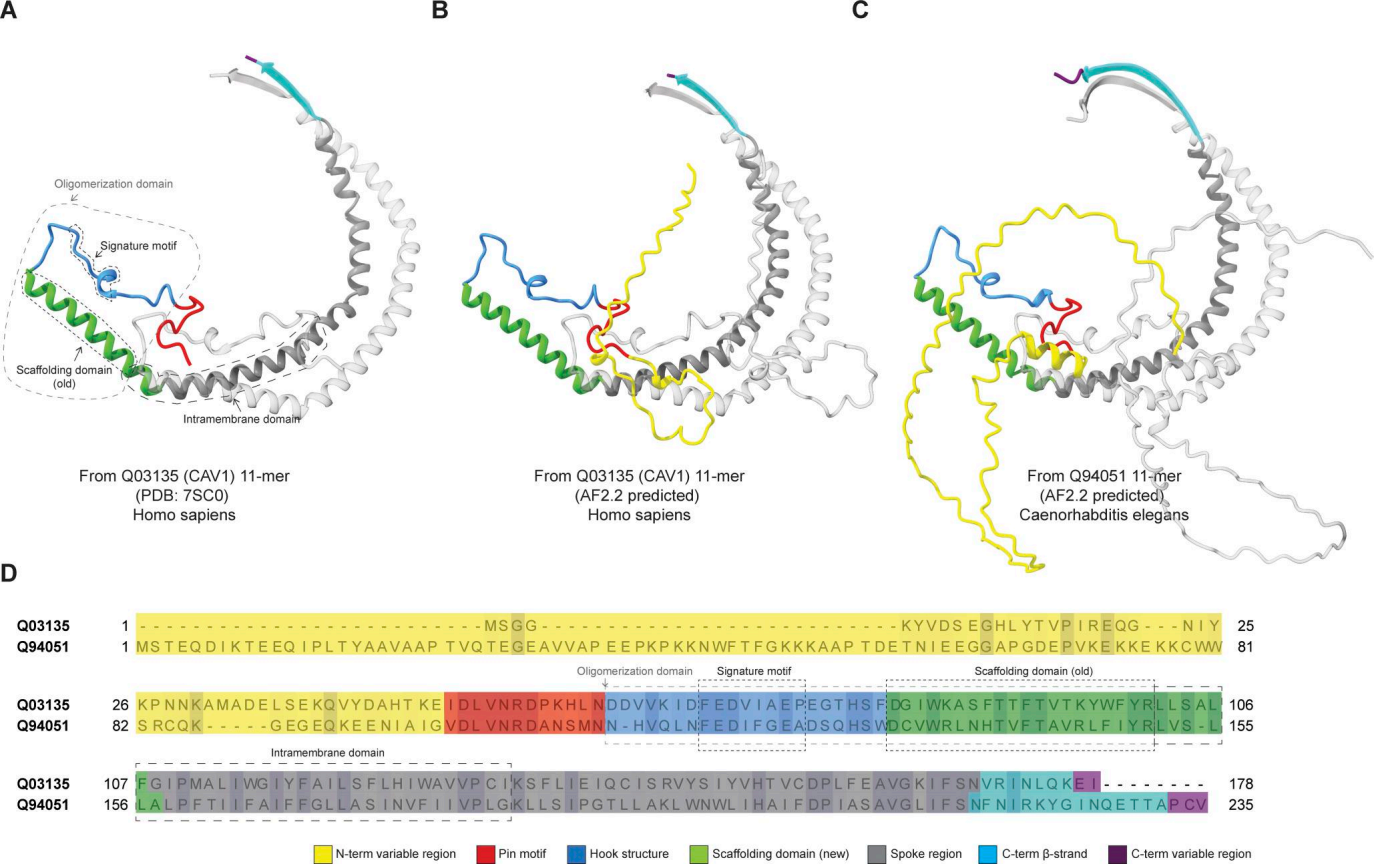
**Proposed structural elements of caveolins. (A–D)** Structural elements include a N-terminal variable region (yellow), pin motif (red), hook region (blue), scaffolding domain (green), spoke region (gray), β-strand (cyan), and C-terminal variable region (purple). For illustration purposes, elements are mapped onto the structures of (A) two neighboring protomers from the cryo-EM–based secondary structure model of the human Cav1 8S complex (PDB: 7SC0), (B) two neighboring protomers from the 11-mer of human Cav1 (Q03135) predicted by AF2.2, and (C) two neighboring protomers from the 11-mer of *C. elegans* caveolin (Q94051) predicted by AF2.2. (D) Comparison between the previous domain designations and our proposed segmentation using structural elements can be observed in both panel (A) and sequence alignments of human Cav1 and *C. elegans* caveolin Q94051.

We next sought to identify common structural motifs shared by these diverse caveolins. Previous studies identified a series of functionally important domains across mammalian caveolins, including the oligomerization, scaffolding, and intramembrane domains (Li et al., 1995; Parton et al., 2006; Root et al., 2015; Sargiacomo et al., 1995; Schlegel et al., 1999). However, these domains were primarily identified by sequence analysis or truncation studies and do not map in a straightforward way to the experimentally determined structure of human Cav1 (Porta et al., 2022). We thus identified seven basic structural units using the structure of human Cav1 and a computationally predicted structure of *C. elegans* caveolin (Q94051) as templates (Fig. 3):

(1) *N-terminal variable region*. In human Cav1, residues 1–48 are predicted to be disordered and were not resolved in the cryo-EM structure (Porta et al., 2022). Similarly, many other caveolins, including *C. elegans* caveolin Q94051, are predicted to contain N-terminal disordered regions (Fig. 3, yellow).
(2) *Pin motif*. The pin motif of human Cav1 (residues 49–60) makes critical contacts with each neighboring protomer at the rim of the 8S complex (Porta et al., 2022). A similar motif is predicted to exist in *C. elegans* caveolin Q94051 (Fig. 3, red).
(3) *Hook structure*. Residues 61–81 of human Cav1 consist of a loop that undergoes a 180° turn (Fig. 3, blue). A similar hook-shaped structural motif is predicted to exist in *C. elegans* caveolin Q94051 (Fig. 3). This structural element corresponds to the first half of the oligomerization domain (residues 61–101) of human Cav1. Embedded within this same region is the highly conserved signature motif (residues 68–75), consisting of a 3/10 helix followed by a short-coiled structure.
(4) *Scaffolding domain*. Residues 82–101 of human Cav1 are traditionally defined as the caveolin scaffolding domain (CSD). This corresponds to the initial α-helix (α-1) of the Cav1 protomer, which forms the periphery of the 8S complex disk (Porta et al., 2022). Importantly however, the α-1 helix extends beyond the classical boundaries of the CSD. Thus, we redefined the entire α-1 region (residues 81–107) as the scaffolding domain, considering its cohesive functional role in the experimental and predicted structures (Fig. 3, green).
(5) *Spoke region*. Residues 108–169 of human Cav1 consist of a series of α-helices connected in tandem by short loops, forming a semi-circle arc of about 180° (Porta et al., 2022). This region encompasses the residues formerly defined as the intramembrane domain (residues 102–134), as well as the helical region we previously designated as the spoke-like region (residues 135–169) (Fig. 3, gray). To reflect the structural continuity of this region, we here define it as the spoke region.
(6) *C-terminal β-strand*. Residues 170–176 at the C terminus of human Cav1 fold into a β-strand that assembles into an amphipathic parallel β-barrel with neighboring protomers (Porta et al., 2022). A β-strand is likewise predicted to exist in *C. elegans* caveolin (Fig. 3, cyan).
(7) *C-terminal variable region*. While the structure of human Cav1 essentially ends in a β-strand, a subset of other caveolins contain an additional C-terminal region that differs in length and composition across caveolins (Fig. 3, purple). Accordingly, we refer to this region as the C-terminal variable domain. The structure of this region is typically predicted by AF2 with low confidence, suggesting it is disordered (Data S3 and Data S4).

**Figure 4.**
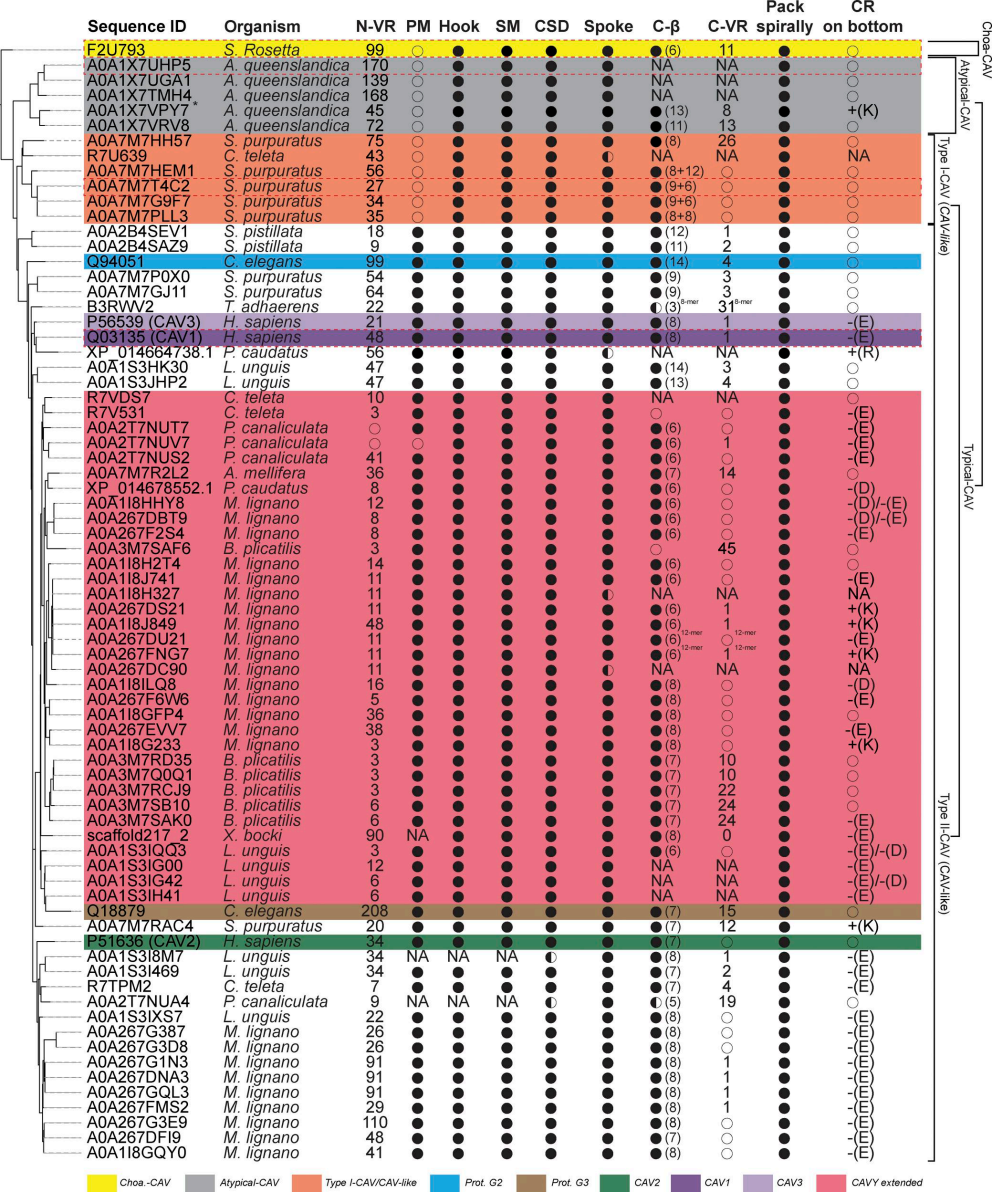
**Summary of the structural features of caveolins suggested by computational modeling predictions.** Phylogenetic tree shown on the left-hand side of the table is based on Fig. S1 A. N-VR, N terminus variable region; PM, pin motif; Hook, hook structure; SM, signature motif; CSD, caveolin scaffolding domain; Spoke, spoke region; C-β, C-terminal β-strand; C-VR, C terminus variable region; Pack spirally, whether the protein sequence is predicted to be assembled into a disk-shaped oligomer; CR, charged residues on the hydrophobic (bottom) side of complexes (“+” represents a positive charge, “−” represents a negative charge, and uppercase English letters represent the corresponding amino acid residue abbreviations). Structural features were summarized mainly based on AF2.2 predicted 11-mers unless otherwise noted in the upper right corner of particular features. ●, structural feature was predicted to be present; ◑, structural feature was predicted to be partially present; ○, structural feature was not predicted to be present. NA, not applicable (corresponding sequences were either missing or shifted due to sequence missing). *, A0A1X7VPY7 was not predicted by AF2.2 to form any homologous disk-like or ring-like complexes. However, it was predicted to form a hybrid complex structure with A0A1X7VRV8. The structural features listed for A0A1X7VPY7 are summarized from the model of the hybrid complex consisting of A0A1X7VPY7 (1 copy) and A0A1X7VRV8 (10 copies).

**Figure 5.**
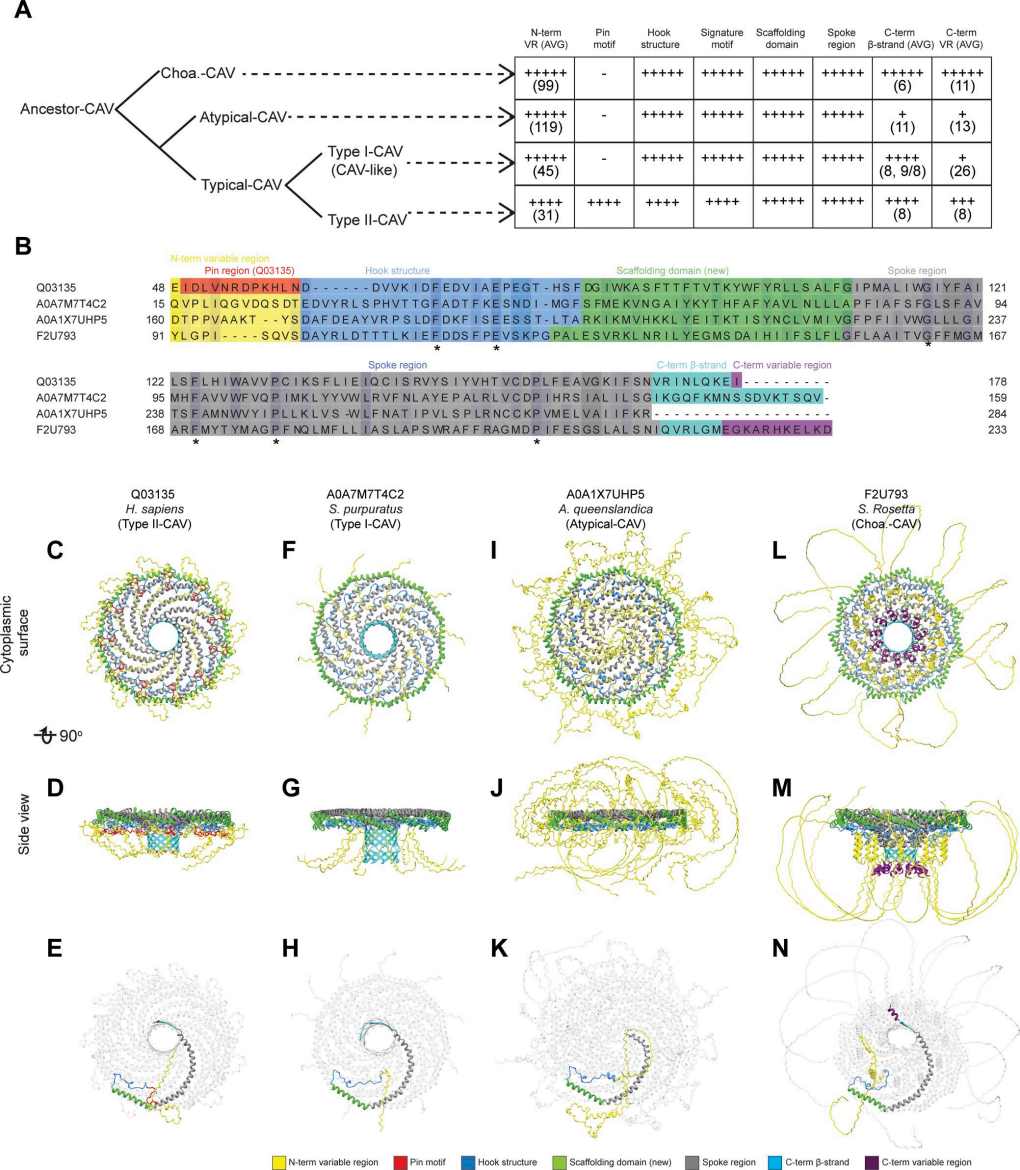
**Predicted structural features of representative metazoan and non-metazoan caveolins. (A)** Model summarizing the key structural similarities and difference in different groups of caveolins based on the phylogenetic analysis and structural comparisons presented in Fig. 4 and Data S3. The number of + symbols in each cell represents the frequency of occurrence of a specific structural unit in the caveolins of the corresponding clade. The number in parentheses indicates the average number of amino acids constituting the structural unit within the caveolin clades (rounded to the nearest integer). For Type I-CAV caveolins where the C-terminal β-strand is predicted to be discontinuous in two segments, the average is calculated separately for caveolins with a single C-terminal β-strand and for those with two segments. The results are separated by a comma for the two types of C-terminal β-strands, and the averages for the two segments are separated by a forward slash. **(B)** Sequence alignment of representative caveolins. Conserved residues are highlighted, with darker intensities corresponding to higher percent identity. Structural features are colored as follows: N-terminal variable region (yellow), pin motif (red), hook region (blue), scaffolding domain (green), spoke region (gray), β-strand (cyan), and C-terminal variable region (purple). **(C–N)** Different views of AF2.2 models of representative caveolins. **(C–E)** Human Cav1 (Type II-CAV, Q03135). **(F–H)***S. purpuratus* (Type I-CAV, A0A7M7T4C2). **(I–K)***A. queenslandica* (Atypical-CAV, A0A1X7UHP5). **(L–N)***S. rosetta* (Choa-CAV, F2U793). In C-N, structural features are colored as in panel B. To better display the structure of a single protomer within the complex, the other 10 protomers in the models in panels E, H, K, and N have been made transparent.

Next, we asked how these structural elements are utilized by different caveolins and how they change during evolution (Figs. 4 and 5). To illustrate key similarities and differences across evolutionarily distant caveolins, we selected four examples taken from the major classes of caveolins: (1) human Cav1, a Type II-CAV; (2) a Type I-CAV from *Strongylocentrotus purpuratus* (A0A7M7T4C2); (3) an atypical caveolin from *A. queenslandica* (A0A1X7UHP5); and (4) the Choa caveolin from *S. rosetta* (F2U793) (Fig. 5, B–N). All four caveolin classes are predicted to contain an N-terminal variable region, hook structure, scaffolding domain, and spoke region (Fig. 5, B–N). Despite being essential for the formation of human Cav1 complexes, the pin motif is found only in Type II caveolins (Fig. 4 and Fig. 5 A). The presence of the C-terminal β-strand also varied across clades. β-Strands were predicted to be absent from 60% of the atypical caveolins, including the representative *A. queenslandica* caveolin (Figs. 4 and 5), but were predicted to exist in an extended form in other clades. The occurrence of the C-terminal variable region was predicted to vary across caveolins, even within the same clade (Fig. 4).

**Figure S3.**
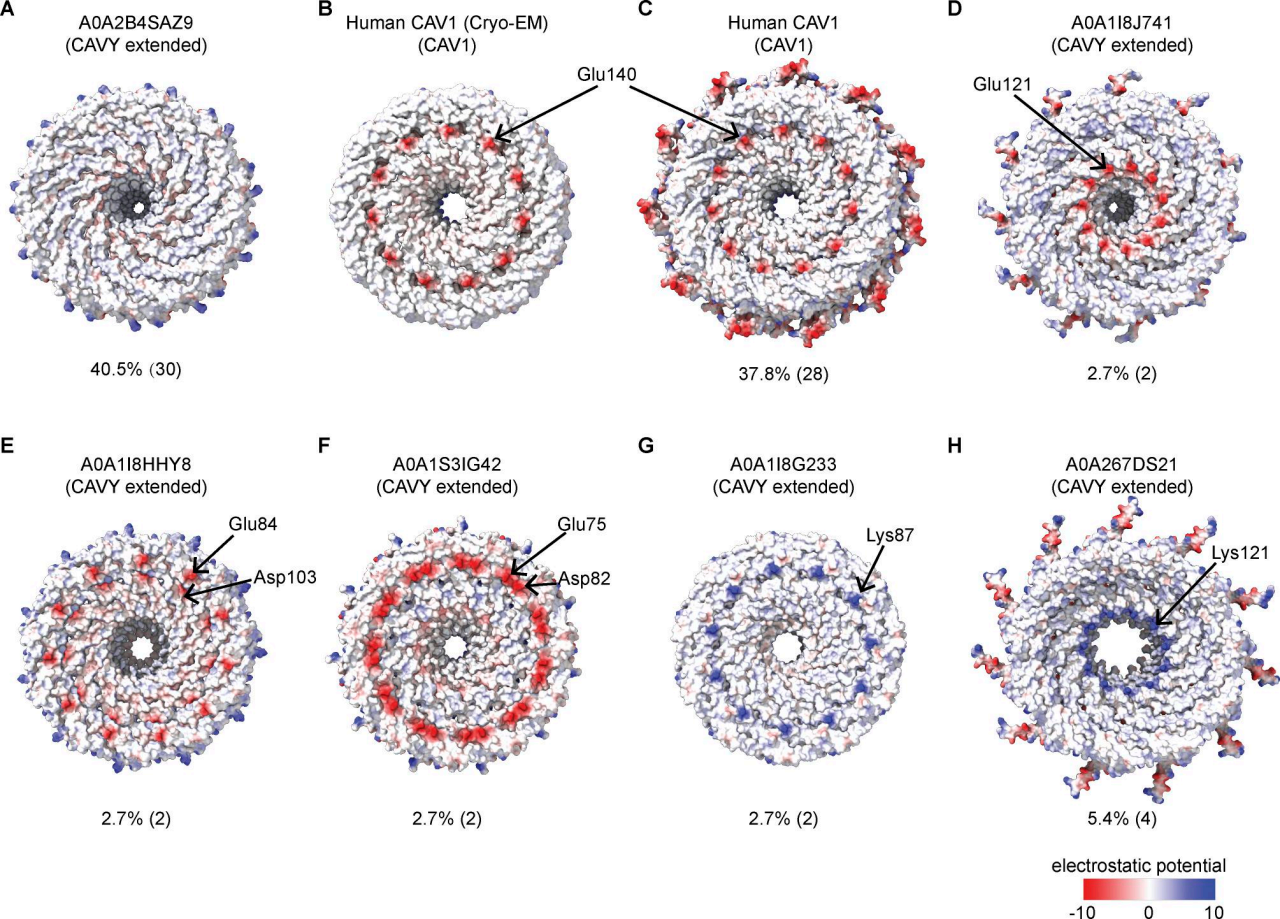
**Electrostatic potential distribution patterns on the proposed lipid bilayer-facing surface of the computationally modeled caveolin oligomers. (A–H)** Examples of different patterns of charged residues on the proposed membrane-facing surface are shown for representative caveolin oligomers predicted by AF2.2. They include (A) completely neutral surface; (B and C) a negatively charged ring contributed by a single Glu or a single aspartic acid (Asp) located in the middle of the spoke region; (D) a negatively charged ring contributed by a single Glu located near the C-terminal region of the spoke region; (E) two negatively charged rings contributed by a Glu or Asp in the middle of the spoke region; (F) a single negatively charged ring contributed by a Glu and an Asp in the middle of the spoke region, (G) a positively charged ring contributed by a lysine (Lys) in the middle of the spoke region, and (H) a positively charged ring contributed by a Lys near the C-terminal region of the spoke region. The percentage of caveolin complexes exhibiting each pattern is listed below each model (from a total of 74 caveolins investigated); Glu, glutamic acid; Asp, aspartic acid.

Finally, we examined the hydrophobic membrane–facing surfaces. Although all the 74 caveolin complexes we examined form a disk with a hydrophobic face, ∼40% of the complexes, including *S. rosetta* and *A. queenslandica* caveolins, have no charged residues on this face, whereas others, including human Cav1, contain a few charged residues that because of the symmetry of the complex form a charged ring circling the hydrophobic face (Figs. 4 and S3).

### Electron microscopy (EM) shows diverse caveolins can form disk-shaped oligomers

**Figure S4.**
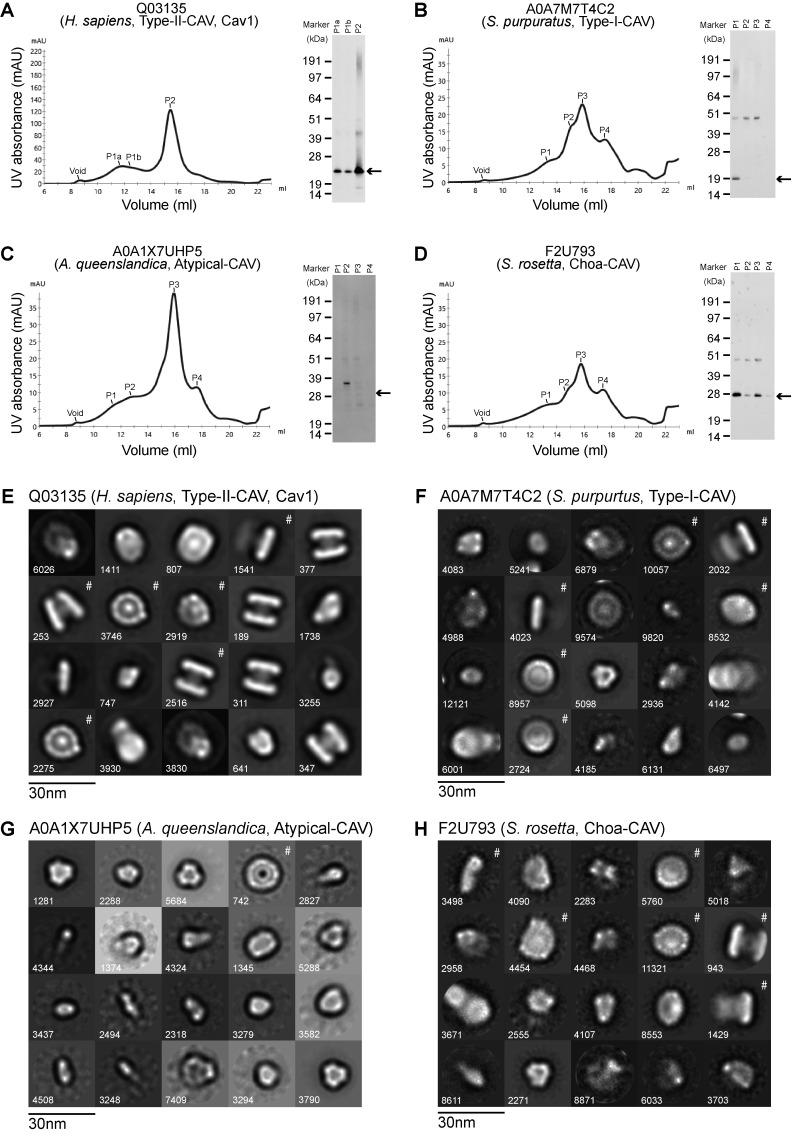
**FPLC traces, western blots of caveolin purifications, and negative stain EM averages of caveolin complexes. (A–D)** Indicated caveolin proteins were purified from *E. coli* membranes and applied to a Superose 6 10/300 Gl column. Elution profiles and western blotting results are shown for (A) human Cav1 (Type II-CAV, Q03135), (B) *S. purpuratus* caveolin (Type I-CAV, A0A7M7T4C2), (C) *A. queenslandica* caveolin (Atypical-CAV, A0A1X7UHP5), and (D) *S. rosetta* caveolin (Choa-CAV, F2U793). The position of the void and shoulders corresponding to various peaks (P1–P4) is indicated on each FPLC trace. Arrows on the western blots point to the expected position for monomers for each of the caveolins based on their predicted molecular weight. **(E–H)** Negative stain 2D class averages of human Cav1 (E), *S. purpuratus* caveolin (F), *A. queenslandica* caveolin (G), and *S. rosetta* caveolin (H). Classes denoted with # are shown in Fig. 6. The number of particles found in each class average is shown in the bottom left. Scale bar, 30 nm. The classes of smaller particles represent a membrane chaperone complex that is a structured protein contaminant in the purifications. For the case of *A. queenslandica* caveolin, the majority of 2D classes consist of these contaminant proteins. Consequently, only one class is marked as the caveolin complex. FPLC, fast protein liquid chromatography. Source data are available for this figure: SourceData FS4.

**Figure 6.**
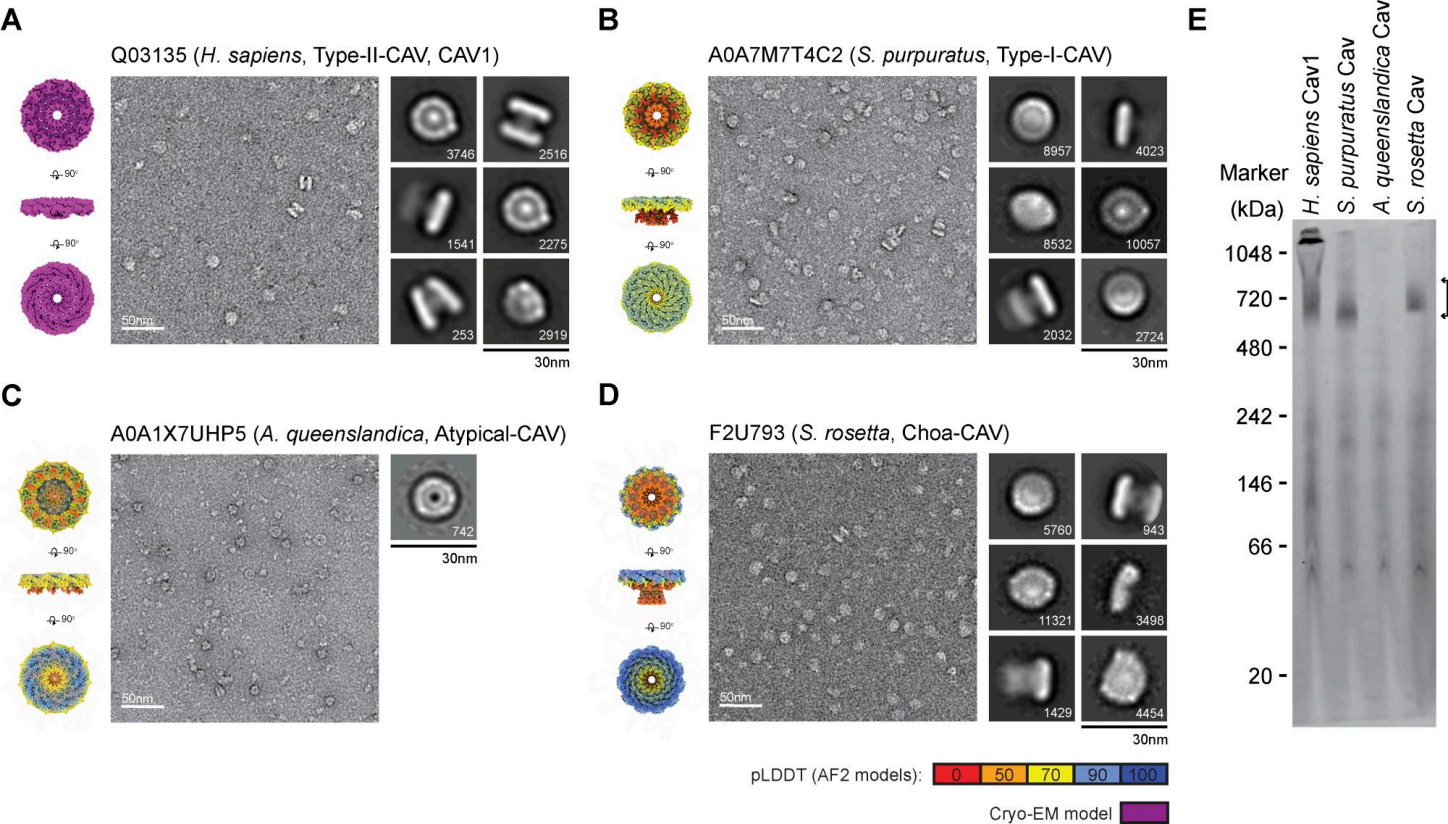
**Negative stain EM shows diverse caveolins assemble into disk-shaped complexes. (A)** *H. sapiens* Cav1, **(B)***S. purpuratus* caveolin, **(C)***A. queenslandica* caveolin, and **(D)***S. rosetta* caveolin. In each panel, surface-filling models for the cryo-EM structure or AF2.2 11-mer structures are shown on the left, representative images of negatively stained caveolin complexes are shown in the middle, and representative 2D averages of caveolins are shown on the right. The number of particles found in each class average is shown in the bottom right. Scale bar, 30 nm. **(E)** Western blot of Blue native gel with bracket marking the position of 8S-like complexes. Molecular weight markers are indicated. Source data are available for this figure: SourceData F6.

Computational modeling is useful to generate hypotheses but requires experimental validation (Terwilliger et al., 2023, *Preprint*). To test key predictions from our evolutionary and computational modeling analyses, we used a combination of biochemistry and negative stain EM to examine the structure of members of four major classes of caveolins. Previous studies have established caveolins expressed in *Escherichia coli* embed in the bacterial inner membrane (Walser et al., 2012) and can be purified from bacterial membranes in the presence of detergent (Han et al., 2023; Han et al., 2020; Porta et al., 2022). The regions of the structure usually associated with or embedded in the membrane become surrounded by a stabilizing detergent micelle (Porta et al., 2022). Using this strategy, human Cav1, *S. purpuratus* (A0A7M7T4C2), *A. queenslandica* (A0A1X7UHP5), and *S. rosetta* (F2U793) sequences were expressed in *E. coli* and purified in detergent using size-exclusion chromatography (SEC). Western blotting of fractions eluted from SEC confirmed that these caveolins form high molecular weight complexes (Fig. S4). To visualize their overall structure, we performed negative stain EM (Fig. 6).

Negative stain 2D class averages of the complexes from each purification were roughly the same size, and most assumed a disk-like geometry although there was some heterogeneity in the shape of the disks (Figs. 6 and S4). *S. purpuratus* and *S. rosetta* caveolins formed uniform disks with distinct inner and outer rings, similar to the appearance of human Cav1 in negative stain (Fig. 6, A, B, and D). The *A. queenslandica* caveolin complex, although not as well ordered, still appeared to form disk-like complexes, but lacked the central density observed in the human, *S. purpuratus*, and *S. rosetta* caveolin classes (Fig. 6 C). The *S. purpuratus* and *S. rosetta* caveolins migrated as stable complexes on blue native gels (Fig. 6 E). In contrast, the *A. queenslandica* caveolin complex was unstable, possibly due to its inability to form a β-barrel (Fig. 6 E). Taken together with our computational models, these results show members of all four classes of caveolins, even though evolutionarily distant, can assemble into disk-shaped complexes, suggesting a conserved structural “fingerprint” for the caveolin family of proteins.

### Cryo-EM reveals the molecular basis for the assembly of the *S. purpuratus* caveolin complex

**Figure 7.**
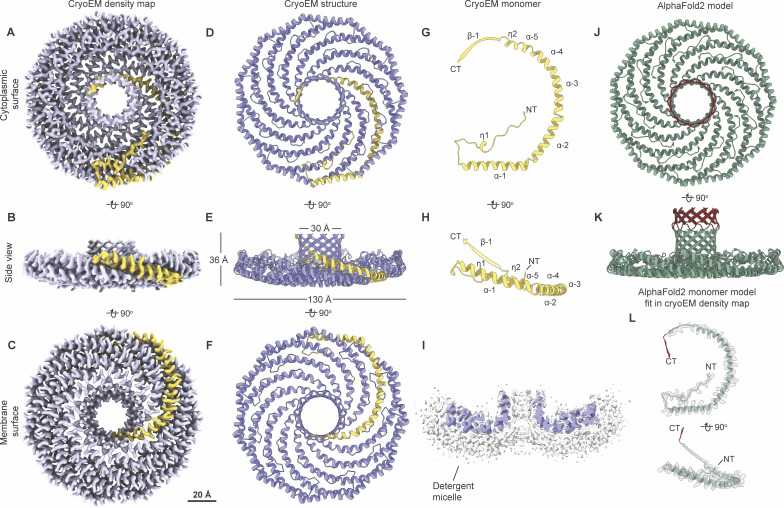
***S. purpuratus* caveolin forms an 11-mer complex. (A–C)** 3.1 Å resolution cryo-EM density map of the *S. purpuratus* caveolin complex with 11-fold symmetry. The complex is shown with ninety-degree rotated views displaying the cytoplasmic-facing surface (A), side (B), and membrane-facing surface (C). The complex has an overall disklike structure with 11 spiraling α-helices and a central β-barrel. A single protomer is highlighted in yellow. Scale bar, 20 Å. **(D–F)** Secondary structure model of the *S. purpuratus* caveolin in the same views as shown in panels A–C. **(G and H)** Secondary structure of *S. purpuratus* caveolin protomer with secondary features and N and C termini noted. **(I)** Central slice of the density map (purple) with the detergent micelle (gray). **(J and K)** AF2.2-predicted structure of the *S. purpuratus* caveolin 11-mer showing views of the cytoplasmic-facing surface (J) and side view (K). Structured regions predicted by AF2.2 that are not found in the cryo-EM structure are highlighted in burgundy. **(L)** Protomer from the AF2.2 11-mer model fit into the cryo-EM density map (gray outline) of a protomer.

**Figure S5.**
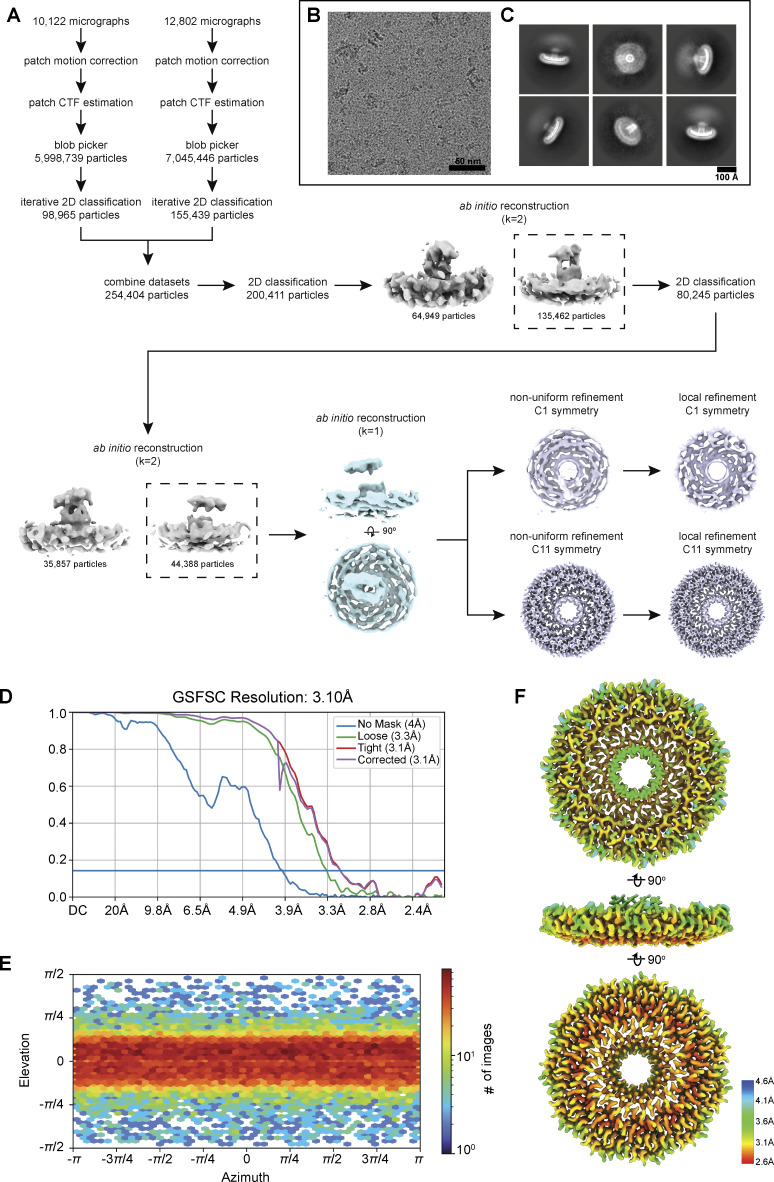
**Flowchart of cryo-EM processing steps for the *S. purpuratus* caveolin complex. (A)** Flowchart representing the classification and analysis of *S. purpuratus* caveolin complex micrographs. Two independently collected datasets were combined following preprocessing, particle picking, and initial 2D classification. *Ab initio* reconstructions that were used for further processing are noted with dashed boxes. *Ab initio* reconstruction used as an input for nonuniform refinement is shown in light blue in an *en face* view and rotated 90° around the x axis. Nonuniform and local refinements with no symmetry applied (C1) and 11-fold symmetry applied (C11) of the *S. purpuratus* caveolin complex are shown in lavender in an *en face* view. **(B)** Representative micrograph of the *S. purpuratus* caveolin complex. Scale bar, 50 nm. **(C)** Representative *S. purpuratus* caveolin complex 2D classes. Box size, 352 pix^2^ (390.7 × 390.7 Å). Scale bar, 100 Å. **(D)** GS-FSC of C11 refinement with no mask (blue line), loose mask (green line), tight mask (red line), and corrected mask (purple). Blue horizontal line, FSC = 0.143. **(E)** Euler angle plot of angles of particle distribution for the C11 reconstruction of the *S. purpuratus* caveolin complex. **(F)** Heat map of local resolution of C11 3D reconstruction, rotated around the x axis. GS-FSC, gold-standard Fourier shell correlation; FSC, Fourier shell correlation.

We used single-particle cryo-EM to determine a 3.1 Å resolution structure of the *S. purpuratus* caveolin complex and built a model spanning amino acids 29–152 (Figs. 7 and S5; and Table S1). Residues 1–28 and 153–159, regions predicted to be disordered (Fig. 4), were not visible in the map. The complex, ∼130 Å in diameter and ∼36 Å in height (Fig. 7 E), is composed of 11 spiraling α-helical protomers organized into a disk with a protruding β-barrel (Fig. 7, B and E). The variable N-terminal region extends along the cytoplasmic face of the complex before making a 180° turn at the hook structure. Amphipathic α-helices form the spoke region, with the α-1 helix forming the rim of the disk that is ∼15 Å in height, and the C terminus of each protomer is a β-strand that forms a central parallel β-barrel ∼30 Å in diameter (Fig. 7, G and H). An unstructured detergent micelle covers the hydrophobic face of the disk and reaches into the interior of the β-barrel, suggesting both regions interact with membrane (Fig. 7 I). No defined density was detected in the β-barrel even with no applied symmetry. Comparison of the experimental and AF2.2 structures shows the computational model did not correctly predict the length of the β-barrel or the curvature of the disk (Fig. 7, J–L).

### Cryo-EM structure of the choanoflagellate *S. rosetta* caveolin complex

**Figure 8.**
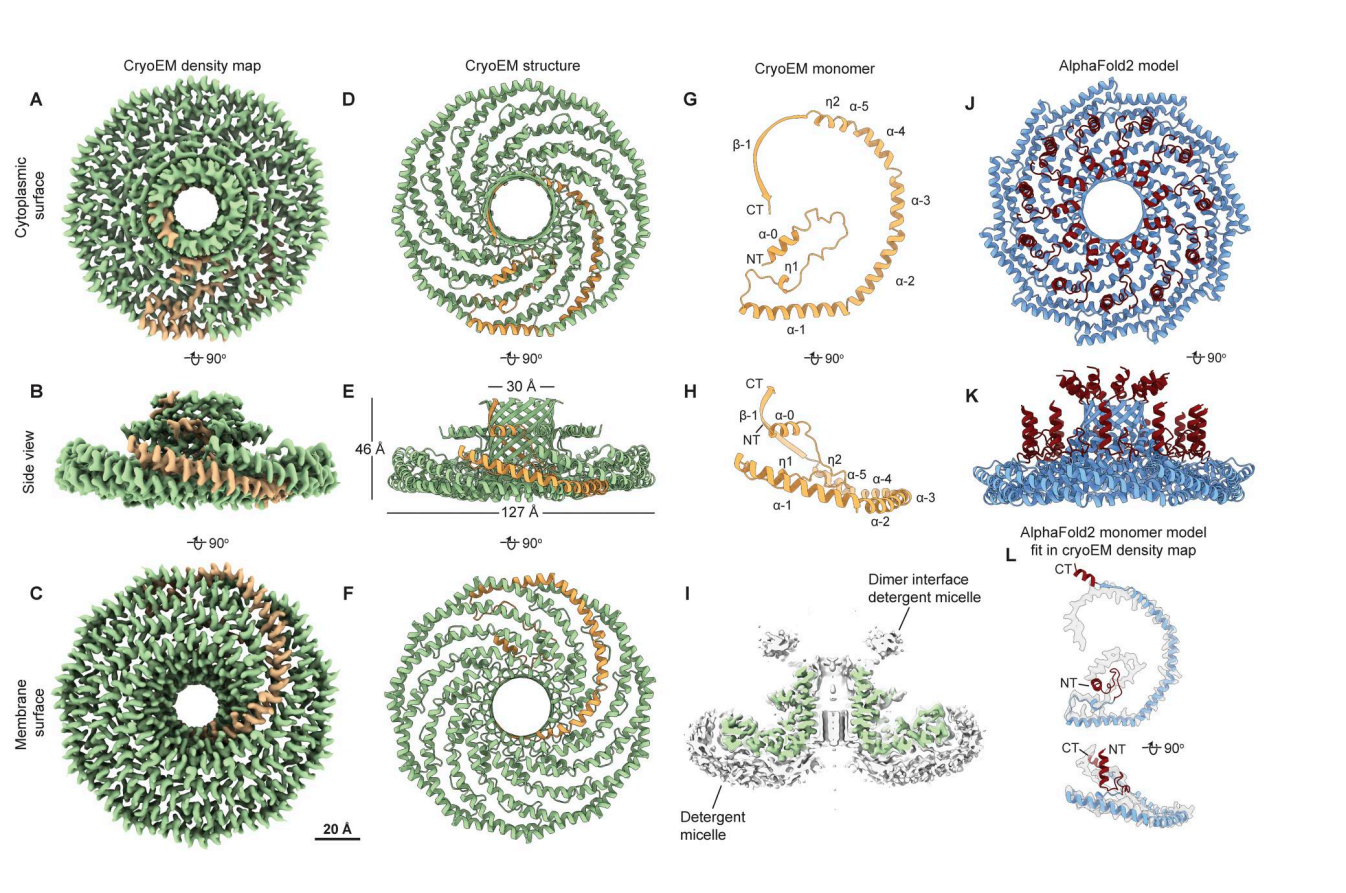
***S. rosetta* caveolin forms an 11-mer complex with an elongated β-barrel and extended N-terminal region.** (**A–C)** 2.9 Å resolution cryo-EM density of the *S. rosetta* caveolin complex with 11-fold symmetry. The complex is shown with ninety-degree rotated views displaying the cytoplasmic-facing surface (A), side (B), and membrane-facing surface (C). The complex has an overall disklike structure with 11 spiraling α-helices, a central elongated β-barrel, and extended N-terminal region. A single protomer is highlighted in orange. Scale bar, 20 Å. **(D–F)** Secondary structure model of the *S. rosetta* caveolin complex in the same views as shown in panels A–C. **(G and H)** Secondary structure of *S. rosetta* caveolin protomer with secondary features and N and C termini noted. **(I)** Central slice of the density map (green) with the detergent micelle (gray). **(J and K)** AF2.2-predicted structure of the *S. rosetta* caveolin 11-mer showing views of the cytoplasmic-facing surface (J) and side view (K). Structured regions predicted by AF2.2 that are not found in the cryo-EM structure are highlighted in burgundy. **(L)** Protomer from the AF2.2 11-mer model fit into the cryo-EM density map (gray outline) of a protomer.

**Figure S6.**
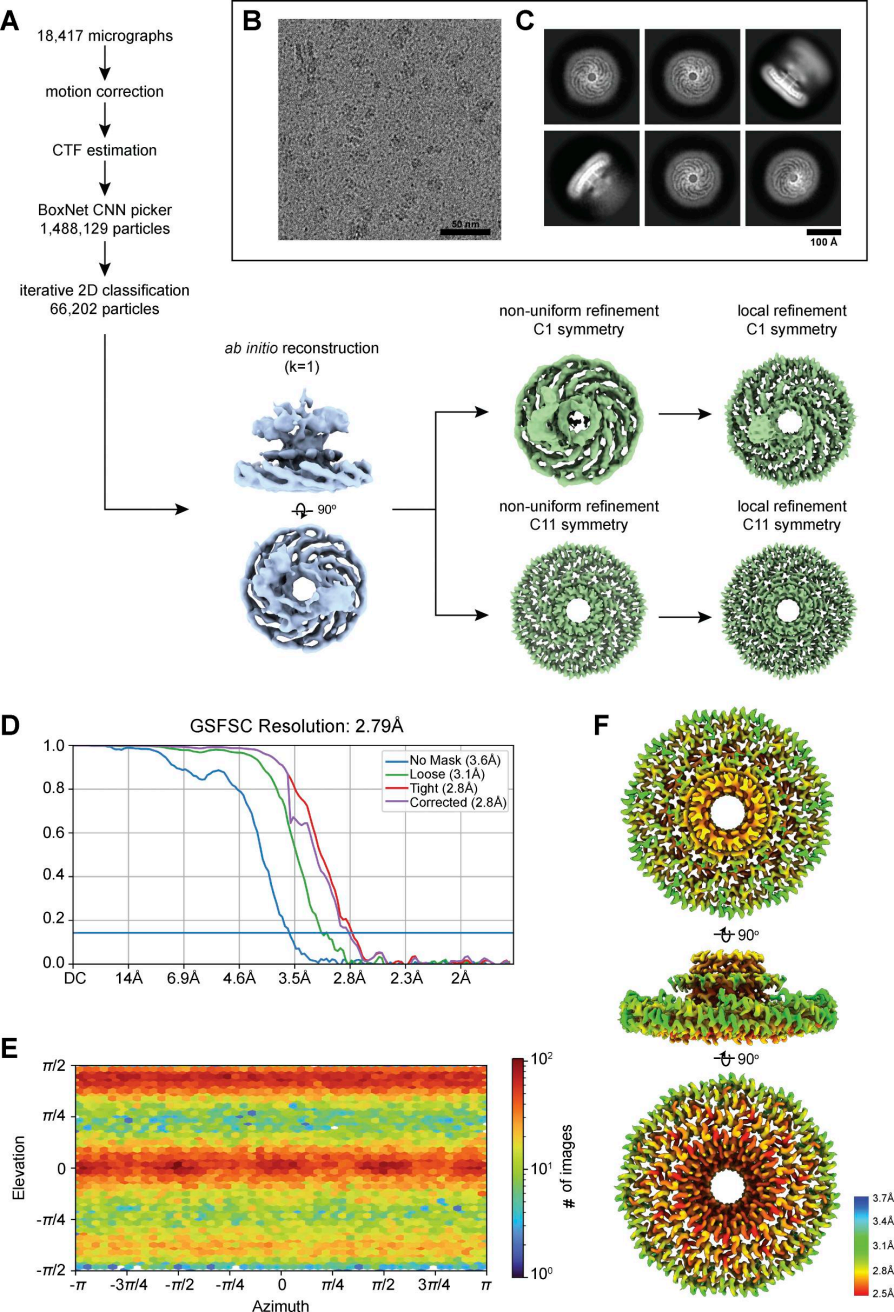
**Flowchart of cryo-EM processing steps for the *S. rosetta* caveolin complex. (A)** Flowchart depicting the classification and analysis of *S. rosetta* caveolin complex micrographs. *Ab initio* reconstruction used as an input for nonuniform refinement is shown in light blue in an *en face* view and rotated 90° around the x axis. Nonuniform and local refinements with no symmetry applied (C1) and 11-fold symmetry applied (C11) of the *S. rosetta* caveolin complex are shown in green in an *en face* view. **(B)** Representative micrograph of the *S. rosetta* caveolin complex. Scale bar, 50 nm. **(C)** Representative *S. rosetta* caveolin complex 2D classes. Box size, 300 pix^2^ (261 × 261 Å). Scale bar, 100 Å. **(D)** GS-FSC of C11 refinement with no mask (blue line), loose mask (green line), tight mask (red line), and corrected mask (purple). Blue horizontal line, FSC = 0.143. **(E)** Euler angle plot of angles of particle distribution for the C11 reconstruction of the *S. rosetta* caveolin complex. **(F)** Heat map of local resolution of C11 3D reconstruction, rotated around the x axis. GS-FSC, gold-standard Fourier shell correlation; FSC, Fourier shell correlation.

We next determined a 2.9 Å resolution cryo-EM structure of the *S. rosetta* caveolin complex (Figs. 8 and S6; and Table S1). A model of *S. rosetta* caveolin spanning amino acids 79–231 was built from the density map (Fig. 8, D–F). There was no density for the predicted disordered N-terminal region (a.a. 1–78) and the C-terminal residues (a.a. 232–233). The *S. rosetta* caveolin complex, ∼127 Å in diameter and ∼46 Å in height, is a disk composed of spiraling α-helices with a central β-barrel. The variable N-terminal region forms a short α-helix that is positioned about halfway up the β-barrel before snaking along the cytoplasmic-facing surface of the complex. Each protomer makes a 180° turn at the hook structure, which is followed by the spoke region. The α-1 helix forms the rim at the edge of the disk that is ∼16 Å in height (Fig. 8, G and H). Finally, *S. rosetta* caveolin has a C-terminal β-strand that forms a parallel β-barrel with a diameter of ∼30 Å and contributes to the ∼46 Å overall height of the complex (Fig. 8 E). The detergent micelle surrounds the hydrophobic face of the disk and reaches into the β-barrel (Fig. 8 I). As with the other caveolin complexes, no interpretable density was found in the β-barrel, even with no applied symmetry. For this complex, AF2.2 failed to predict the structure of the N- and C-terminal portions of *S. rosetta* caveolin or the correct curvature of the disk (Fig. 8, E and J–L).

### Evolutionarily distant caveolins share structural motifs but differ in dimensions

**Figure 9.**
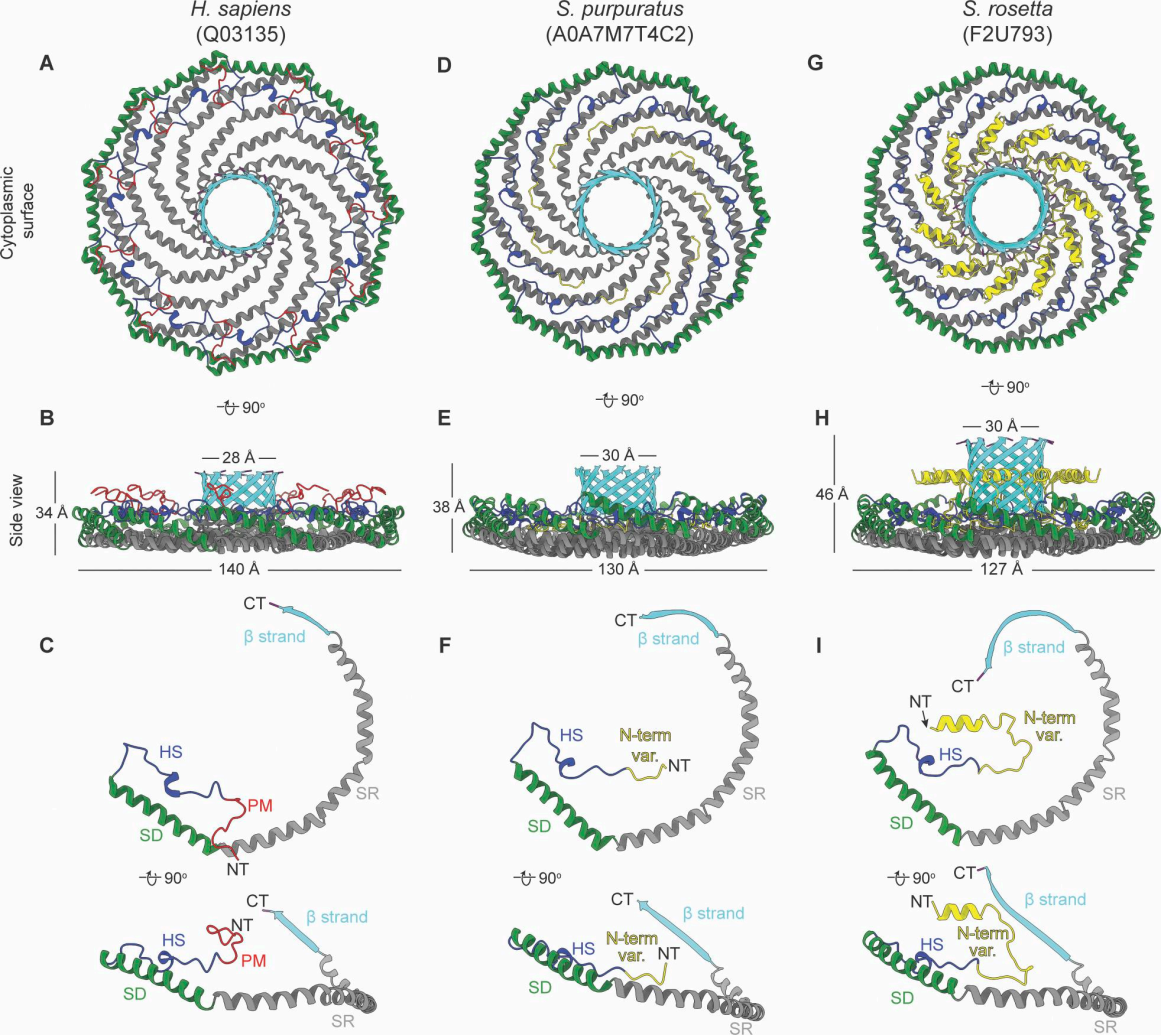
**Comparison of cryo-EM structures for *H. sapiens*, *S. rosetta*, and *S. purpuratus* caveolin complexes. (A, B, D, E, G, and H)** Cytoplasmic-facing surface and side views of cryo-EM structures are shown for human Cav1 (A and B), *S. purpuratus* caveolin (D and E), and *S. rosetta* caveolin (G and H). **(C, F, and I)** Protomer structures extracted from the 11-mers are shown in same views as the panel above. Domains are colored as follows: N-terminal variable region (yellow), PM (red), HS (blue), SD (green), SR (gray), β-strand (cyan). NT, N terminus; CT, C terminus; PM, pin motif; HS, hook structure; SD, scaffolding domain; SR, spoke region.

**Figure S7.**
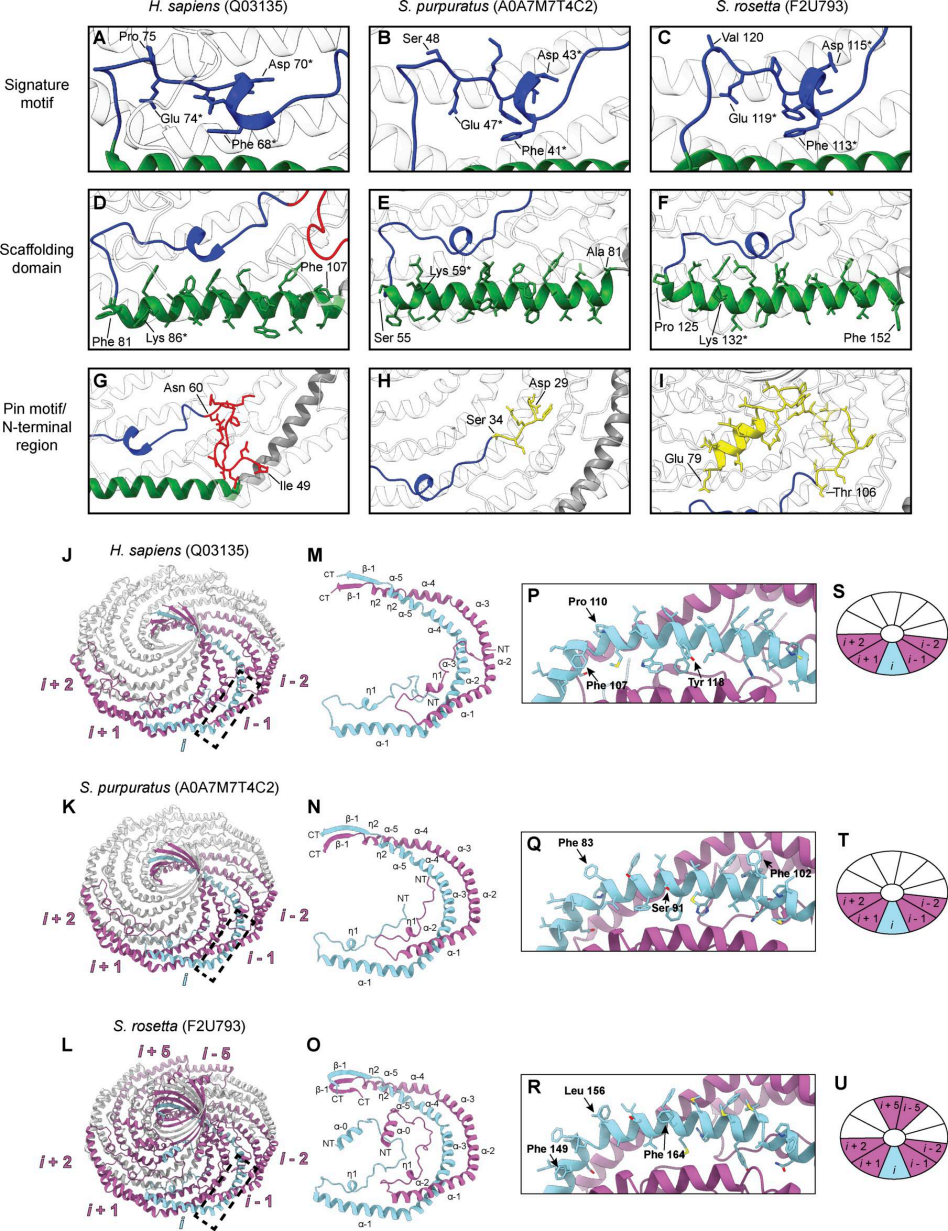
**Comparison of the signature motif, scaffolding domain, pin motif/N-terminal region, and protomer interfaces for *H. sapiens*, *S. rosetta*, and *S. purpuratus* caveolins. (A–I)** Detailed view of the signature motifs (A–C) (blue), scaffolding domains (D–F) (green), and pin motif (G) (red) or N-terminal variable regions (H and I) (yellow) of the human Cav1, *S. rosetta* caveolin, and *S. purpuratus* caveolin complexes. One protomer is colored according to the structural elements as described in Fig. 2, while other protomers of the complex are depicted in transparent gray. The first and last residues of the motifs are labeled, and any residues that are absolutely conserved between the three caveolins are labeled and marked with an asterisk. **(J–L)** Overall structure of caveolin complexes highlighting one protomer, *i*, in light blue and its interacting protomers in magenta. The interacting protomers are labeled *i* −2 to *i* + 2 for the caveolin complexes with the *S. rosetta* caveolin complex also exhibiting interactions at protomers *i* −5 and *i* + 5. **(M–O)** Packing of two protomers with secondary structure elements labeled. **(P–R)** Zoomed-in view of membrane-facing residues indicated by a dashed box on overall caveolin structures in J–L. Complexes are rotated −160° around the x axis from J–L to show the membrane-facing surface. Membrane-interacting residues of interest are noted. **(S–U)** Cartoon depiction of caveolin complexes to illustrate the organization of interacting protomers. Color and labeling scheme remain the same as (J–L).

We next directly compared the structures of the *S. purpuratus* and *S. rosetta* caveolin complexes with the previously determined structure of the human Cav1 complex (Fig. 9). Consistent with the notion that protein structure is more well conserved than sequence (Huang et al., 2013), the secondary structure of the protomers and organization of *S. purpuratus* and *S. rosetta* caveolin complexes are similar to human Cav1 despite sharing no significant sequence similarity (16%/35% identity/similarity for *S. purpuratus* caveolin and 13%/28% for *S. rosetta* caveolin) (Fig. 9). Each complex forms a disk with 11 spiraling α-helices and a central β-barrel, with each protomer forming contacts with two protomers to the left and two to the right (Fig. S7). The spoke regions and scaffolding domains that make up the disks contain a similar number of residues (88, 87, and 90 residues for human Cav1, *S. purpuratus*, and *S. rosetta* caveolins, respectively). However, both the *S. purpuratus* and *S. rosetta* caveolin complexes are smaller in diameter (∼130 Å and ∼127 Å, respectively) compared with human Cav1 (∼140 Å). Although the *S. purpuratus* and *S. rosetta* caveolin complexes exhibit tighter packing of the η1 and α1 helices than the human Cav1 complex, this packing loosens toward the rim of the complexes. The difference in diameter between the complexes is due to the increased curvature of the *S. purpuratus* and *S. rosetta* caveolin complexes compared with the human Cav1 complex. The three β-barrels have a similar outer diameter of ∼28–30 Å; however, the *S. rosetta* caveolin complex’s central β-barrel is ∼10 Å longer than the other caveolin complexes (Fig. 9, B, E, and H).

While the overall organization of the three caveolin complexes is similar, their N-terminal structured regions differ significantly. In human Cav1, the pin motif interacts with other protomers along the rim region and then is directly followed by the hook structure. However, the *S. purpuratus and S. rosetta* caveolins do not have pin motifs. In *S. purpuratus* caveolin, the N-terminal region extends outward from the middle of the hydrophilic α-helical face until it reaches the hook structure (a.a. 35–55) where it makes a ∼180° turn (Fig. 9). In contrast, for *S. rosetta* caveolin the N-terminal region forms a short α-helix (a.a. 79–88) about halfway up the C-terminal β-barrel and extends parallel to the disk on the cytoplasmic side of the complex (Fig. 9, G and H). As a result, in the *S. rosetta* complex, additional contacts are made with the *i* + 5 and *i* − 5 protomers as the variable N-terminal region of *i* rises up the central β-barrel and contacts the C-terminal β strands of *i* + 5 and *i* − 5 (Fig. S7).

### *S. purpuratus* and *S. rosetta* caveolin complexes form amphipathic disks with increased curvature compared with the human Cav1 complex

**Figure S8.**
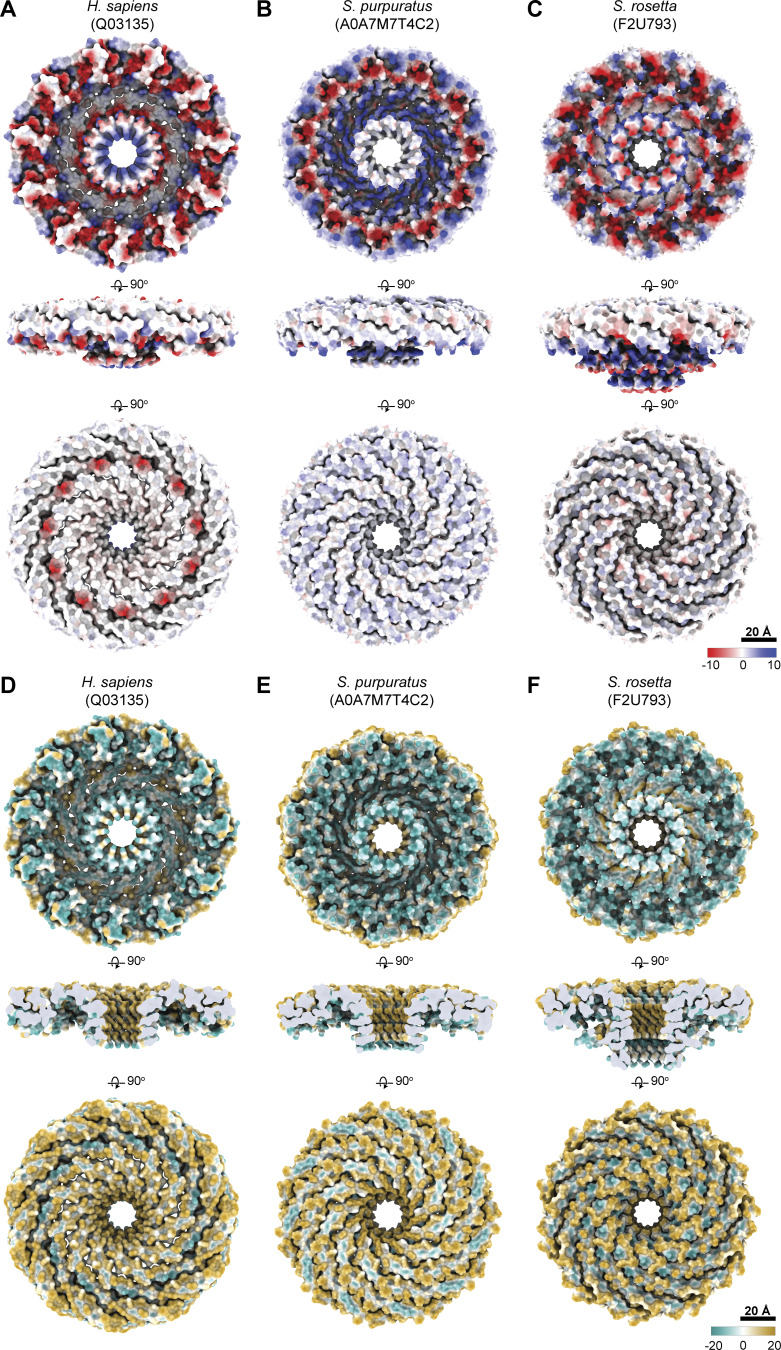
**Distribution of charged residues and hydrophobicity of the predicted membrane- and cytoplasmic-facing surfaces of *H. sapiens*, *S. rosetta*, and *S. purpuratus* caveolin complexes. (A–F)** Space-filling models of the caveolin complexes rotated 90°, showing (A–C) the charge of the amino acids or (D–F) hydrophobicity values. Note that side views in A–C are shown with the surface of the complex, whereas a cut through the center of the complex is shown in D–F.

A key feature of the human Cav1 8S complex is the amphipathic nature of the disk (Porta et al., 2022) (Fig. S8, A and D). The *S. purpuratus* and *S. rosetta* caveolin complexes likewise contain distinct hydrophobic and hydrophilic faces (Fig. S8, B, C, E, and F). In contrast to the ring of glutamic acid residues on the hydrophobic face of the human Cav1 complex, the hydrophobic surface of *S. purpuratus* and *S. rosetta* caveolins lacks any charged residues (Fig. S8, B and C). The hydrophilic faces of the complexes have an array of charged, but not conserved, residues (Fig. S8, A–C). Finally, the interior of the conserved central β-barrel is hydrophobic in all three complexes. Only the human Cav1 β-barrel is capped by a charged residue (Lys176) (Fig. S8, D–F).

**Figure 10.**
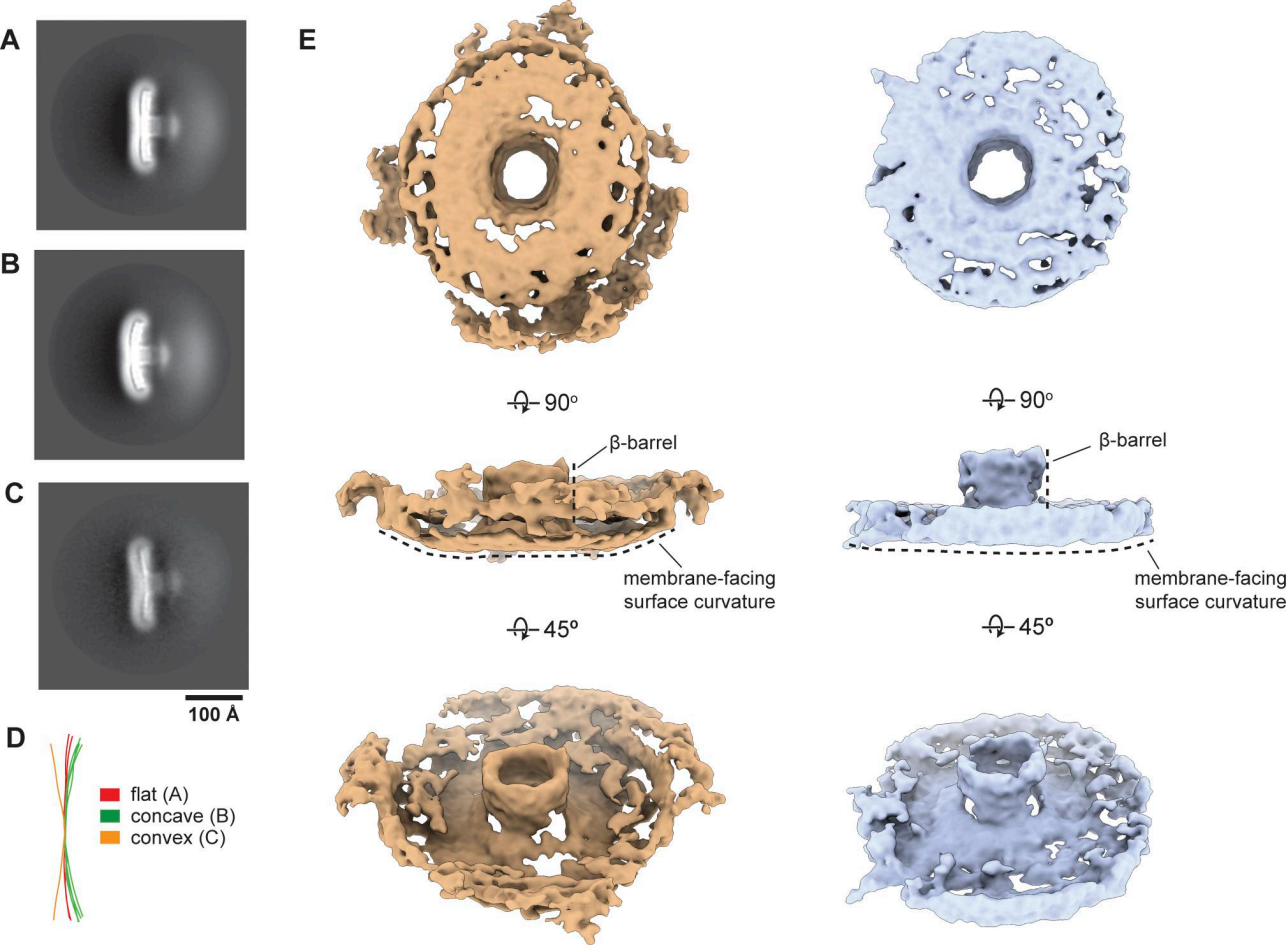
***S. purpuratus* caveolin displays various curvatures in 2D classes and 3DVA. (A–C)** *S. purpuratus* caveolin 2D class averages that show a flat (A), concave (B), and convex (C) curvature of the complex. Scale bar, 100 Å. **(D)** Difference in curvature is highlighted with overlaid traces of the membrane-facing surface from 2D class averages. **(E)** Structures representing the negative and positive values along the reaction coordinate of a 3DVA component calculated with a resolution limit of 8 Å. The complex on the left (orange) shows a concave membrane surface curvature, while the complex on the right (light blue) shows a flat membrane surface curvature and “lifting” of the β-barrel above the rim of the complex. The proposed membrane-facing surface is shown (top), rotated 90° around the x axis to show a side view (middle), and rotated an additional 45° to show a view of the predicted cytoplasmic-facing surface (bottom).

The membrane-facing surface of the human Cav1 8S complex is flat (Porta et al., 2022) (Fig. 9 A). In contrast, both the *S. purpuratus* and *S. rosetta* caveolin complexes are concave, with curvatures of ∼17° and ∼11°, respectively (Fig. 9, E and H). 2D averages of vitrified *S. purpuratus* caveolin complexes showed variations of curvature, including examples of complexes with either concave or convex curvatures (Fig. 10, A–D). While we were unable to determine high-resolution structures from these averages, 3D variability analysis (3DVA) of the *S. purpuratus* caveolin complex results in components that capture a range of continuous complex conformations (Video 1). Calculated using a filter resolution of 8 Å, these low-resolution structures represent the negative and positive values along the reaction coordinate for the component with the largest variance (Fig. 10 E). This analysis shows that the *S. purpuratus* caveolin disk can be concave, similar to the 3.1 Å structure, or can be flat. The differences in curvature are accommodated by the spoke region rising in pitch toward the center of the complex, which leads the β-barrel to be pushed “outward” ∼4–5 Å toward what would be the cytoplasm (Video 1). The 2D averages from the *S. rosetta* caveolin or human Cav1 samples did not show classes with different curvatures (Porta et al., 2022), and 3DVA did not identify components displaying significant variations in the shape of the disks. Thus, we conclude that the *S. purpuratus* caveolin complex is more flexible than the human or *S. rosetta* caveolin complexes under these experimental conditions.

**Video 1.** **3DVA of *S. purpuratus* Cav.** 3DVA of *S. purpuratus* caveolin shows 3D density maps along the variability component that exhibits the complex with a concave and flattened membrane surface conformation. Movement of the *S. purpuratus* caveolin complex from the concave to flattened membrane surface conformations results in the α-helices in the central spoke region lifting the beta barrel ∼5 Å. The complex is shown at a side view and then rotated ∼45° along the x axis.

## Discussion

Using a combination of computational and structural approaches, we now show that caveolins across evolution share the ability to assemble into amphipathic disk–shaped multimers composed of spiraling α-helices and a central protruding parallel β-barrel. Somewhat surprisingly considering the ability of AF2 to build disk-shaped complexes with different numbers of protomers, all three of the experimentally determined cryo-EM structures have 11-fold symmetry. While our analyses reveal many conserved features of caveolin oligomers, they also uncover striking differences such as highly variable N and C termini and variations in the curvature of the membrane-facing surface of the disk. These findings suggest that caveolins adopt a conserved structural framework built around an amphipathic disk, but are adaptable enough to accommodate significant molecular variations.

Classically, caveolins have been depicted as consisting of several major domains including a signature motif, scaffolding domain, oligomerization domain, intramembrane domain, and C-terminal region. Based on our structural and modeling results, we propose a new domain nomenclature, consisting of N-terminal variable region, pin motif, hook structure, scaffolding domain, spoke region, β-strand, and C-terminal variable region. Of these elements, the hook structure, scaffolding domain, and spoke region are found in almost every clade that was examined making them the most conserved structural features across caveolins. These regions of the protein contribute to oligomerization, as well as help define the hydrophobic membrane-facing surfaces of the complex. In human Cav1, the β-barrel also contributes to the proper packing of protomers into regular disk-shaped complexes and is disrupted by several disease-associated mutations (Han et al., 2020). Interestingly however, not all caveolins are predicted to have β-strands that contribute to β-barrels, such as the *A. queenslandica* caveolin studied here. This could explain why the *A. queenslandica* caveolin complexes are less regular and stable than those formed by caveolins capable of assembling central β-barrels. Whether the β-barrels fulfill additional physiological roles beyond complex organization and structural stability also remains to be determined.

By comparing the predicted and experimental structures of caveolins, we not only identified regions of caveolins that are structurally well conserved, but also elements that differ significantly across caveolins. While the pin motif appears essential for Type II-CAVs, such as the human Cav1 complex (Porta et al., 2022), it is probably the latest structural unit formed during the evolutionary process and is not essential for caveolins from other evolutionary branches to pack into oligomeric complexes. The length and composition of the N- and C-terminal unstructured regions are also highly variable across caveolins, suggesting that they may be tuned for specific functions in various organisms. Because both regions are expected to project into the cytoplasm, we speculate that they could represent a binding site for cytosolic proteins, including cavins in vertebrates (Tillu et al., 2021). We also noted that a subset of caveolins, including human Cav1, contain charged residues on the membrane-facing surface. The functional significance of these charged residues is not yet clear but could potentially impact lipid packing around the complex (Doktorova et al., 2025).

Although caveolins are found throughout Metazoa, they are not pan-metazoan. For example, it is well known that caveolins are not found in *Drosophila melanogaster* despite being present in other invertebrates (Kirkham et al., 2008; Parton et al., 2020a). In the current study, we now report that caveolins are absent in ctenophores and Chaetognatha, at least based on currently available genomes. We also found that caveolins are found not only across Metazoa, but also in the closest unicellular relative of animals: choanoflagellates. These findings place what was previously thought to be a core animal feature onto the ancestor of the Choanozoa, thereby expanding our understanding of the membrane biology of the unicellular ancestors to both animals and choanoflagellates. The finding that caveolin-related proteins are found in the choanoflagellate *S. rosetta* also implies that the proteins evolved independently in the two lineages for the past billion years if the protein was present in the ancestor of Choanozoa, or the past 600 million years in a scenario of horizontal gene transfer to the ancestor of the Myriazoa (Schultz et al., 2023). This raises the interesting question of what functional roles caveolins fulfill in choanoflagellates and how these relate to their functions in mammals. A potential clue is that several binding partners and signaling pathways that caveolins have been linked to in mammals are also found in choanoflagellates (King et al., 2003; Segawa et al., 2006; Shalchian-Tabrizi et al., 2008). They could also help buffer changes in membrane tension by a caveolae-independent mechanism (Lolo et al., 2023). As experimental approaches to study the cell biology of choanoflagellates continue to advance (Booth et al., 2018), it should be possible to test the structure–function relationship of this evolutionarily distant form of the protein in the future.

The unexpected identification of a choanoflagellate caveolin also prompted us to examine the evolutionary history of caveolins more deeply. In addition to performing conventional phylogenetic analysis, we examined the chromosome-scale evolutionary history of caveolins from ALGs. This powerful approach has recently been employed at the chromosome scale to resolve the long-argued question of the evolutionary relationship between sponges, ctenophores, and other animals (Schultz et al., 2023). Here, we traced ALG–caveolin colocalizations to track the chromosomal origins of caveolins in animals and infer the relationship between animal and choanoflagellate caveolins. The consistent presence of caveolin orthologs on chromosomes containing the Eb ALG suggests that the ancestral caveolin, which gave rise to all non-sponge caveolins, was present on ALG Eb in the ancestor of Parahoxozoa, dating its critical biological role to before the Cambrian explosion. The persistence of these sequences on homologous chromosomes, even in light of lineage-specific chromosomal changes, is similar to the conservation of biologically critical genes on single ALGs, such as the persistence of the Hox cluster on ALG B2 in parahoxozoans (Schultz et al., 2024, *Preprint*; Simakov et al., 2020). Future work to test whether this is the result of cis-regulatory constraints (Irimia et al., 2012) may reveal yet undiscovered loci important to caveolin biology.

There are several limitations to our study. While AF2 is able to predict the basic secondary structure and the overall organization of caveolins into spirally packed disks, it does less well in predicting the structure of the N- and C-terminal domains of *S. purpuratus* and *S. rosetta* caveolins. How the oligomeric state of caveolin complexes is controlled is also uncertain. The experimental structures of human Cav1, *S. purpuratus* caveolin, and *S. rosetta* caveolin are all 11-mers. However, AF2 predicts additional oligomeric states can exist beyond the experimentally observed 11-mers (although some are less likely to exist due to energetic strains) (Gulsevin et al., 2023). It is possible that the oligomerization state of the complexes may be influenced by expression and purification conditions of our experiments. The functional consequences of the structural differences between caveolins also remain to be determined. As one example, the differences in curvature in the membrane-facing surface observed in the cryo-EM structures of human, *S. purpuratus*, and *S. rosetta* caveolins could indicate they are optimized to bend membranes into different shapes and/or undergo conformational changes in response to changes in membrane tension. The functional impact of the extension of the β-barrel of *S. rosetta* compared with that of human and *S. purpuratus* caveolins will also require further study. Finally, although the relationships between caveolins within clades are clear, based on the current dataset the phylogenetic relationship between the clades is not well defined. Future analysis using alignments of whole caveolin sequences could help resolve this ambiguity.

In summary, we conclude that the ability of caveolins to assemble into amphipathic disks represents an ancient, unique mode of protein–lipid membrane interactions that predates the emergence of metazoans. Given these new insights, it should now become possible to uncover how caveolins control cellular function at a molecular level across an evolutionary scale.

## Materials and methods

### AlphaFold predictions

Predicted structures of caveolin monomers, dimers, and homo-oligomers were generated using AF2.1, AF2.2, or AF3.0. For AF2.1 predictions, we systematically increased the number of monomer input sequences until the upper limit of the residues that could be analyzed was reached. AlphaFold v2.1 predictions were performed using a Colab notebook named “AlphaFold2_advanced” with default settings (Mirdita et al., 2022). Due to the upper limit in the number of residues that could be analyzed by AF2.1, where indicated, caveolin sequences were truncated to exclude the predicted N-terminal disordered regions. AlphaFold v2.2 predictions were performed for caveolin monomers, dimers, and 11-mers using default settings via another Colab notebook named “alphafold21_predict_colab” provided by ChimeraX daily builds version (ChimeraX 1.4.0) (RRID:SCR_015872). Version v2.2 includes updated AlphaFold-Multimer model parameters. See https://github.com/deepmind/alphafold/releases for a description of differences in AF2.1.0 versus AF2.2. AlphaFold v3.0 predictions for caveolin monomers, dimers, and 11-mers were performed using the AlphaFold Server (https://alphafoldserver.com/) (Abramson et al., 2024). Unless otherwise stated, the rank model 1 of the 5 models’ output for each prediction is shown. Confidence levels of the predictions are rendered on the models using predicted local-distance difference test values (Jumper et al., 2021).

### Sequence alignments

Clustal Omega (RRID:SCR_001591) was used to perform sequence alignments. Jalview 2.11.2.4 (RRID:SCR_006459) (Waterhouse et al., 2009) was used for alignment image typesetting and exporting.

### HMMER searches

For HMMER searches, a HMM profile was built directly from a MAFFT alignment of previously reported truncated caveolin sequences (Kirkham et al., 2008) using hmmbuild from the HMMER package v 3.1b2 (RRID:SCR_005305) (Eddy, 2011). The profile was then searched against the *S. rosetta* ATCC 50818 NCBI reference genome and *A. queenslandica* genome v1.1 from Ensembl Metazoa (RRID:SCR_000800) using hmmsearch. To identify putative ctenophore caveolins, we used blastp v2.10.0^+^ (Altschul et al., 1997) with the human protein sequences as queries, and HMMER with the above models to query the *Hormiphora californensis* (Schultz et al., 2021) and *Bolinopsis microptera* (Schultz et al., 2023) genomes (GCA_020137815.1 and GCF_026151205.1) and transcriptomes (TSA GHXS00000000 and GCF_026151205.1 annotation).

### Phylogenetic analyses

The selected caveolin homologs were aligned with MAFFT v7.310 (RRID:SCR_011811) (Katoh et al., 2002; Katoh and Standley, 2013). The alignment was then truncated to a region corresponding to the residues 54–158 in human Cav1 using a simple Python (RRID:SCR_008394) script (https://doi.org/10.5281/zenodo.6562402, Wilson, 2022). Gaps were removed before combining the sequences with those from the supplementary information of Kirkham et al. (2008) and realigning with MAFFT. ProtTest3 v3.4.2 (Darriba et al., 2011) was then used to determine the best model of evolution (LG+I+Γ). Finally, a maximum-likelihood phylogeny was inferred using RAxML v8.2.11 (RRID:SCR_006086) (Stamatakis, 2006; Stamatakis, 2014) with 1,000 rapid bootstraps. The trimmed tree was produced in RStudio using the *ape* package (Paradis and Schliep, 2019). Trees were rendered using FigTree v1.4.4 (RRID:SCR_008515) and prepared for publication using Inkscape v1.0.1 (RRID:SCR_014479).

For the Bayesian analysis, gaps were removed before combining the sequences with those from the supplementary information of Kirkham et al. (2008) and realigning with MAFFT. Columns with a GUIDANCE2 score below 0.93 were removed from the alignment using GUIDANCE2 v2.0.2 (Sela et al., 2015). The alignment was converted to a nexus file with AliView v1.28 (RRID:SCR_002780) (Larsson, 2014). Bayesian phylogenies were inferred for the pre-GUIDANCE and GUIDANCE-filtered alignments with MrBayes v3.2.7a (RRID:SCR_012067) (Ronquist et al., 2012) using the parameters 1,000,000 generations, 4 chains, 0.2 temperature, 100 sampling frequency, 2,500 sump burn-in, 2,500 sumt burn-in, with model selection after burn-in. The BLOSUM model was selected by MrBayes in both runs, likely due to the high conservation of caveolin across phyla.

### Caveolin ALG analysis

ALG analysis was carried out using previously described methods (Schultz et al., 2023) using chromosome-scale animal genomes obtained from *Acropora millepora* (GCF_013753865.1), *Asterias rubens* (GCF_902459465.1), *Biomphalaria glabrata* (GCF_947242115.1), *Branchiostoma floridae* (GCF_000003815.2), *Dreissena polymorpha* (GCF_020536995.1), *Ephydatia muelleri* (Kenny et al., 2020), *Gallus gallus* (GCF_016699485.2), *Holothuria leucospilota* (GCA_029531755.1), *Homo sapiens* (GCF_000001405.40), *Lethenteron reissneri* (GCF_015708825.1), *Lytechinus pictus* (GCF_015342785.2), *Lytechinus variegatus* (GCF_018143015.1), *Mercenaria mercenaria* (GCF_021730395.1), *Mya arenaria* (GCF_026914265.1), *Nematostella vectensis* (GCF_932526225.1), *Oscarella lobularis* (GCF_947507565.1), *Patella vulgata* (GCF_932274485.2), *Pecten maximus* (GCF_902652985.1), *Petromyzon marinus* (GCF_010993605.1), *Pomacea canaliculata* (GCF_003073045.1), *Rhopilema esculentum* (Li et al., 2020), and *S. rosetta* (GCA_033442325.1) (Data S1). To assess the ALG homology with these chromosomal sequences, we used the snakefile “odp” from odp software v.0.3.3 (Schultz et al., 2023) with the option “ignore_autobreaks: True,” “diamond_or_blastp: diamond,” “duplicate_proteins: pass,” “plot_LGs: True,” and “plot_sp_sp: False.” We used the resulting .rbh files and .pdfs to identify significant (P ≤ 0.05, one-sided Bonferroni-corrected Fisher’s exact test) ALG homologies. To identify caveolin orthologs in each genome, we searched the annotation keywords for caveolin, and verified the putative identities using blastp v.2.16.0^+^ (RRID:SCR_001010) (Altschul et al., 1997) and HMMER v.3.4 (Potter et al., 2018). TimeTree v.5 (Kumar et al., 2022) was used to generate a figure of species divergence times, although the animal topology was adjusted after recent studies (Schultz et al., 2023; Strassert et al., 2021).

### Expression and purification of recombinant caveolins in *E. coli*

Protein expression and purification were performed following previously described protocols with minor modifications (Han et al., 2020). Genes encoding the entire caveolin polypeptide of *A. queenslandica* (UniProt Accession A0A1X7UHP5), *S. purpuratus* (UniProt Accession A0A7M7T4C2), and *S. rosetta* (UniProt Accession F2U793) were synthesized by GenScript, NJ, and subcloned into the pET20b(+) vector (Novagen) using NdeI and XhoI (New England Biolabs) restriction sites. The primers used (Integrated DNA Technologies) were as follows: *A. queenslandica* forward 5′-GCG​GCC​CAT​ATG​CCT​CCA​CCC​CCT​CCC​CCG-3′ and reverse 5′-GCG​GCC​CTC​GAG​TCT​TTT​GAA​GAT​AAT​GGC​CAC​AG-3′; *S. purpuratus* forward 5′-GCG​GCC​CAT​ATG​GAA​CTG​ATC​CAT​CCT​G-3′ and reverse 5′-GCG​GCC​CTC​GAG​CAC​CTG​GCT​GGT​CTT​AAC​ATC​AGA​GC-3′; and *S. rosetta* forward 5′-GCG​GCC​CAT​ATG​AGC​TAC​CAC-3′ and reverse 5′-GCG​GCC​CTC​GAG​GTC​CTT​CAG​TTC​CTT​GTG​G-3′. Resulting plasmids were verified by Sanger sequencing (Genewiz/Azenta Life Sciences). Caveolin proteins were expressed in *E. coli* BL21 using the autoinduction expression system (Han et al., 2020). Initially, an MDG starter culture of bacteria was incubated at 37°C and 250 rpm for 20 h. Subsequently, the culture was expanded using autoinducing ZYM-5052 media at 25°C and 300 rpm for 24 h. The *E. coli* cells were then washed with 0.9% NaCl and resuspended in a buffer containing 200 mM NaCl and 20 mM Tris–HCl, pH 8.0. Bacterial cells were homogenized using a French press pressure homogenizer, with 1 mM PMSF and DTT added immediately before homogenization. To remove large cell debris, the homogenate was centrifuged at 9,000 rpm for 15 min at 4°C. Total membranes were subsequently pelleted by centrifugation at 40,000 rpm (Ti-45 rotor; Beckman Coulter) for 1 h at 4°C. The membrane pellets were then homogenized using a Dounce tissue grinder in a buffer consisting of 200 mM NaCl, 20 mM Tris–HCl (pH 8.0), and 1 mM DTT. To solubilize caveolin proteins from the membranes, a 10% n-dodecyl-β-D-maltopyranoside (C12M) (Anatrace) stock solution was added to the membrane homogenate to a final concentration of 2%, and the mixture was gently stirred for 2 h at 4°C. Insoluble material was removed by centrifugation at 42,000 rpm (Ti-50.2 rotor) for 35 min, and the supernatant was subjected to Nickel Sepharose affinity purification. After washing the resin using 8 column volumes of a buffer composed of 200 mM NaCl, 20 mM Tris–HCl, pH 8.0, 1 mM DTT, 0.05% C12M, and 60 mM imidazole, the protein was eluted by increasing the imidazole concentration to 300 mM. The eluate containing caveolin was concentrated and further purified by SEC using a Superose 6 Increase 10/300 Gl column (GE Healthcare) in a buffer containing 200 mM NaCl, 20 mM Tris–HCl (pH 8.0), 1 mM DTT, and 0.05% C12M.

### Electrophoresis and western blotting

Electrophoresis and western blotting for both SDS–PAGE and blue native PAGE were performed as described previously (Copeland et al., 2017; Han et al., 2016; Han et al., 2023; Han et al., 2020; Han et al., 2015). Specifically, electrophoresis procedures were carried out using XCell SureLock Mini-Cell Electrophoresis System (EI0001; Thermo Fisher Scientific Inc.). SDS–PAGE electrophoresis was performed using the NuPAGE Bis-Tris system. Blue native PAGE electrophoresis was conducted using the NativePAGE Bis-Tris Mini Protein Gels system. The transfer step in western blotting was carried out using the Mini Trans-Blot Electrophoretic Transfer Cell (170-3930, 170-3935, 170-3989, 170-3936; Bio-Rad). Rabbit anti-6X His pAb (catalog number 137839) from Abcam was used at a 1:2,000 dilution. IRDye 800CW Donkey anti-Rabbit IgG Secondary Antibody was used at 1:10,000 dilution (926-32213; LI-COR Biotechnology). The western blotting results were scanned using a LI-COR Odyssey scanner (LI-COR Biotechnology). The 800-nm channel was used to capture signals from the target protein, while the 680-nm channel was used to detect the molecular weight bands.

### Negative stain EM and data processing

Caveolin samples were prepared for negative stain EM using established methods (Ohi et al., 2004). Briefly, a PELCO easiGlow glow discharge unit was used to glow-discharge 200-mesh copper grids covered with carbon-coated collodion film (EMS) for 30 s at 10 mA. Evolutionary caveolin samples (3.5 μl) were adsorbed to the grids and incubated for 1 min at room temperature. Samples were first washed with two drops of water and then stained with two drops of 0.7% (wt/vol) uranyl formate (EMS). The samples were then blotted until dry. A Morgagni transmission electron microscope operated at an accelerating voltage of 100 kV (Thermo Fisher Scientific) was used to image the samples at a nominal magnification of 22,000x (2.1 Å per pixel).

Negative stain EM datasets were collected with a Tecnai Spirit T12 transmission electron microscope operated at 120 kV (Thermo Fisher Scientific). Images were collected at a nominal magnification of 30,000x (2.086 Å per pixel). Data were collected using SerialEM v4.0.8 software (Mastronarde, 2005) on a 4 k × 4 k Rio camera (Gatan) with a −2.2 µm defocus value. All image processing was performed in RELION-4.0.0 (RRID:SCR_016501) (Kimanius et al., 2021). Approximately 1,000 particles were manually selected and 2D-classified, and clear resulting classes were selected and used as references for particle picking on all micrographs. Particles were extracted with a 144-pixel box size (30 × 30 nm boxes). The extracted particles were 2D-classified into 20 classes. The human Cav1, *S. purpuratus* caveolin, *A. queenslandica* caveolin, and *S. rosetta* caveolin datasets had 39,786, 124,021, 66,856, and 94,597 particles, respectively.

### Cryo-EM sample preparation

For single-particle cryo-EM, 4 μl of the protein sample (*S. purpuratus* caveolin: ∼0.02 mg/ml; *S. rosetta* caveolin: ∼0.04 mg/ml) was applied to a Quantifoil R 2/2, 200-mesh Cu grid with an ultrathin carbon layer (Electron Microscopy Services) that was glow-discharged for 30 s at 5 mA. Following a 30 s incubation, the sample was removed, and another 4 μl of protein sample was applied to the same grid. After a second 30 s incubation, the sample was blotted for 5 s with a blot force of 10 and was then plunge-frozen in a slurry of liquid ethane using a Vitrobot Mark IV (Thermo Fisher Scientific). The chamber was kept at 4°C with 100% humidity.

### Cryo-EM data collection

Micrographs of the *S. purpuratus* caveolin sample were collected on a Titan Krios transmission electron microscope (Thermo Fisher Scientific) operated at 300 kV and equipped with a K3 direct detection camera with a BioQuantum energy filter used with a slit width of 20 eV (Gatan). Images were collected at a nominal magnification of 81,000x (1.11 Å/pixel). Two datasets were collected of the *S. purpuratus* caveolin sample with 10,127 and 12,803 micrographs using SerialEM v4.0.3 software with a total dose of 60 e^−^/Å^2^ and a defocus range of −0.5 to −3 µm (Mastronarde, 2005).

Images of the *S. rosetta* caveolin sample were collected on a Titan Krios transmission electron microscope (Thermo Fisher Scientific). The microscope was operated at 300 kV and equipped with a K3 direct detection camera with a BioQuantum energy filter used with a slit width of 20 eV (Gatan). Images were collected at a nominal magnification of 105,000x (0.87 Å/pixel). A total of 18,417 micrographs were collected using SerialEM v4.0.3 software with a total dose of 59.17 e^−^/Å^2^ and a defocus range of −0.5 to −3 µm (Mastronarde, 2005).

### Image processing of the *S. purpuratus* caveolin complex

All image processing, 2D classification, and 3D refinements were performed in cryoSPARC v4.2.1 and v4.3.1 (RRID:SCR_016501) (Punjani et al., 2017). Two independently collected datasets of 10,127 and 12,803 movies, respectively (22,930 total movies), were corrected for local-beam–induced drift using patch motion correction. Patch CTF estimation was used to estimate the local CTF parameters. Following exposure curation to keep only micrographs with a CTF estimation of ≤5 Å, 22,845 total micrographs were subjected to circular blob picking. After blob picking, 13,044,185 initial picks were extracted in 352-pixel^2^ boxes (390.72 × 390.72 Å). Iterative 2D classification resulted in 200,411 particles. These particles were input into a two-class *ab initio* 3D reconstruction. The 135,462 particles that contributed to the reconstruction yielding a better defined caveolin complex were subjected to a further round of 2D classification, resulting in 80,245 particles. These particles were used for another two-class *ab initio* 3D reconstruction. The 44,388 particles contributing to the better resolved *ab initio* reconstruction were input into a single-class *ab initio* 3D reconstruction with higher initial and maximum resolution parameters. The resulting *ab initio* 3D reconstruction and the 44,388 particles contributing to it were used as inputs for C1 and C11 nonuniform refinements. The C1 nonuniform refinement resulted in a map with a resolution of 6.6 Å, and the C11 nonuniform refinement produced a map with a resolution of 3.2 Å. Local masks were generated in ChimeraX and RELION-4.0 to mask out the detergent micelle (Kimanius et al., 2021; Meng et al., 2023). These masks were then used to run local refinements with C1 and C11 symmetry. The C1 refinement resulted in a map of 6.2 Å resolution, and the C11 refinement reached a final resolution of 3.1 Å. All 3DVA was carried out in cryoSPARC v4.3.1 (RRID:SCR_016501) (Punjani and Fleet, 2021).

### Image processing of the *S. rosetta* caveolin complex

18,417 movies were motion-corrected, and CTF was estimated using Warp (Tegunov and Cramer, 2019). Particles were picked using the BoxNet2 centralized neural network (CNN) in Warp, and 1,488,129 initial particles were extracted with a box size of 300-pixel^2^ (261 × 261 Å). Iterative 2D classification of particles in cryoSPARC v4.2.1 resulted in 66,202 particles (Punjani et al., 2017). These particles were then input to a single-class *ab initio* 3D reconstruction that resulted in a reconstruction exhibiting 11 spiraling α-helices. The 66,202 particles contributing to the *ab initio* reconstruction were used for a nonuniform refinement with C1 symmetry that resulted in a map with a resolution of 3.8 Å. Imposing C11 symmetry for nonuniform refinement of the same particles resulted in a 3.4 Å map. Using ChimeraX and RELION-4.0, masks were generated to mask out the detergent micelle (Kimanius et al., 2021; Meng et al., 2023). Local refinement using these masks resulted in a C1 refinement with a final resolution of 3.0 Å and a C11 refinement with a final resolution of 2.9 Å.

### Model building, refinement, and validation

Models of the *S. purpuratus* and *S. rosetta* caveolin proteins were built using ModelAngelo with the respective sequences input into the program (Jamali et al., 2024). The output models resulting from ModelAngelo were then further refined using ISOLDE within ChimeraX and Phenix real-space refinement (Croll, 2018; Liebschner et al., 2019; Meng et al., 2023). The final model of *S. purpuratus* caveolin included residues 29–152, and the model of *S. rosetta* caveolin contained residues 79–231. Both models were validated within Phenix (RRID:SCR_014224) (Liebschner et al., 2019). Maps and models are deposited in the EMDB and PDB (*S. purpuratus* caveolin complex: EMDB-47022, PDB-9DN0; *S. rosetta* caveolin complex: EMDB-47023, PDB-9DN1).

### Online supplemental material

Eight supplementary figures are included. Fig. S1 shows the phylogenetic relationships of caveolin sequences inferred from a maximum-likelihood phylogeny; Fig. S2 shows the phylogenetic relationships of caveolin sequences inferred from an unrooted Bayesian tree; Fig. S3 shows electrostatic potential distribution patterns on the proposed lipid bilayer-facing surface of the computationally modeled caveolin oligomers; Fig. S4 shows fast protein liquid chromatography traces, western blots of caveolin purifications, and negative stain EM averages of caveolin complexes; Fig. S5 shows a flowchart of cryo-EM processing steps for the *S. purpuratus* caveolin complex; Fig. S6 shows a flowchart of cryo-EM processing steps for the *S. rosetta* caveolin complex; Fig. S7 shows a comparison of the signature motif, scaffolding domain, pin motif/N-terminal region, and protomer interfaces for *H. sapiens*, *S. rosetta*, and *S. purpuratus* caveolins; and Fig. S8 shows the distribution of charged residues and hydrophobicity of the predicted membrane and cytoplasmic-facing surfaces of *H. sapiens*, *S. rosetta*, and *S. purpuratus* caveolin complexes. Table S1 shows a summary of cryo-EM data collection, refinement, and validation statistics. Video 1 documenting 3DVA of *S. purpuratus* CAV is also provided. Finally, six supplementary datasets are included. Data S1 contains source genomes, annotated proteins, and inferred ALG identities used to infer the ancestral caveolin–ALG relationships (related to Fig. 1), Data S2 shows an alignment of the caveolin sequences clustered in Figs. S1 and S2, Data S3 shows predicted structures of caveolin monomers and oligomers using AlphaFold2.1, Data S4 shows predicted structures of caveolin monomers and oligomers using AlphaFold2.2, Data S5 shows predicted structures of select caveolin monomers, dimers, and 11-mers using AlphaFold3, and Data S6 shows a comparison of AlphaFold prediction results for select caveolins across different AlphaFold versions.

## Supplementary Material

Table S1 shows cryo-EM data collection, refinement, and validation statistics.

Data S1 shows source genomes, annotated proteins, and inferred ALG identities used to infer the ancestral caveolin–ALG relationships (related to Fig 1).

Data S2 shows alignment of the caveolin sequences clustered in Fig. S1 and S2.

Data S3 shows predicted structures of caveolin monomers and oligomers using AlphaFold2.1.

Data S4 shows predicted structures of caveolin monomers and oligomers using AlphaFold2.2.

Data S5 shows predicted structures of select caveolin monomer, dimers, and 11-mers using AlphaFold3.

Data S6 shows comparison of AlphaFold prediction results for select caveolins across different AlphaFold versions.

SourceData F6 is the source file for Fig. 6.

SourceData FS4 is the source file for Fig. S4.

## Data Availability

Maps and models are deposited in the EMDB and PDB (*S. purpuratus* caveolin complex: EMDB-47022, PDB-9DN0; *S. rosetta* caveolin complex: EMDB-47023, PDB-9DN1). All other data needed to evaluate the conclusions in the paper are present in the paper and/or the Supplementary Materials.
