# Supplementary material for: Evolutionarily diverse caveolins share a common structural framework built around amphipathic disks
Source: J Cell Biol. 2025 Aug 7;224(9):e202411175. doi: 10.1083/jcb.202411175 (PMC12330381; doi:10.1083/jcb.202411175)
Supplement: Table S1 — shows cryo-EM data collection, refinement, and validation statistics. [file jcb_202411175_tables1.docx]

**Table S1. Cryo-EM data collection, refinement, and validation statistics**

|  | *S. purpuratus* Caveolin complex  (EMDB-47022)  (PDB-9DN0) | *S. rosetta* Caveolin complex  (EMDB-47023)  (PDB-9DN1) |
| --- | --- | --- |
| **Data collection and processing**  Magnification | 81,000x | 105,000x |
| Voltage (kV)  Electron exposure (e^-^/Å^2^)  Defocus range (μm)  Pixel size (Å)  Symmetry imposed  Initial particle images (no.)  Final particle images (no.)  Map resolution (Å)  FSC threshold  Map resolution range (Å) | 300  60.0  -0.5 to -3  1.11  C11  13,044,185  135,462  3.1  0.143  2.6-4.0 | 300  59.2  -0.5 to -3  0.87  C11  1,488,129  66,202  2.9  0.143  2.5-3.3 |
| **Refinement**  Initial model used (PDB code)  Model resolution (Å)  FSC threshold  Map sharpening *B* factor (Å^2^)  Model composition  Non-hydrogen atoms  Protein residues  Ligands  *B* factors (Å^2^)  Protein  Ligand  R.m.s. deviations  Bond lengths (Å)  Bond angles (º)  Validation  MolProbity score  Clash score  Poor rotamers (%)  Ramachandran plot  Favored (%)  Allowed (%)  Disallowed (%) | Initial model generated by ModelAngelo  3.3  0.5  147.90  11,088  1364  None  -100  None  0.002  0.580  0.94  1.58  0.00  97.84  2.16  0.00 | Initial model generated by ModelAngelo  3.0  0.5  148.57  13,409  1,683  None  -80  None  0.003  0.659  0.69  0.56  0.64  98.01  1.99  0.00 |
