# Supplementary material for: Evolutionarily diverse caveolins share a common structural framework built around amphipathic disks
Source: J Cell Biol. 2025 Aug 7;224(9):e202411175. doi: 10.1083/jcb.202411175 (PMC12330381; doi:10.1083/jcb.202411175)
Supplement: Data S3 — shows predicted structures of caveolin monomers and oligomers using AlphaFold2.1. [file jcb_202411175_datas3.pdf]

File S3. Predicted structures of caveolin monomers and oligomers using AlphaFold2.1.

Note:

The 2D example color sketches were generated from the 3D model by AlphaFold2_advanced notebook.

The left panel of the monomers were colored by N → C; the left panel of the oligomers were colored by chain; the right panels were colored by pLDDT values as below color key indicated:

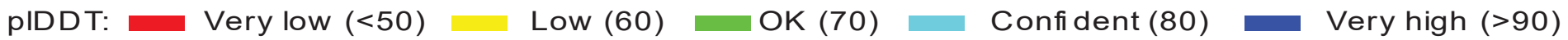

Five models were generated for each prediction. The 2D sketches were based on rank 1 models (R1) in this supplemental file if there is no special note was left under the pLDDT value.

Salpingoeca rosetta

F2U793

	pLDDT		
1-mer	73.91	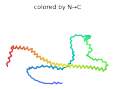	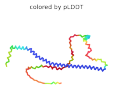
2-mer	59.81	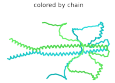	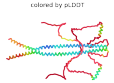
3-mer	47.29	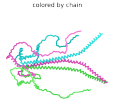	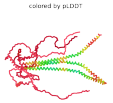
4-mer	57.45	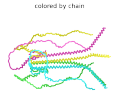	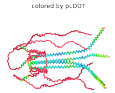
5-mer	42.52	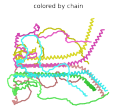	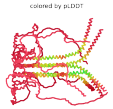
6-mer	42.05	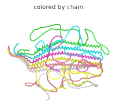	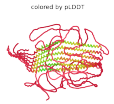
7-mer	45.02	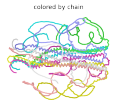	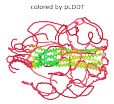

Amphimedon queenslandica

	A0A1X7UHP5	A0A1X7UGT7<br>A0A1X7UHP5 (182-285)	A0A1X7VPY7	A0A1X7UGA1	A0A1X7VRV8	A0A1X7TMH4
	pLDDT	*pLDDT*	*pLDDT*	*pLDDT*	*pLDDT*	*pLDDT*
1-mer	68.65		69.28	64.32	73.34	67.66
2-mer	41.36<br>(R5)		61.25	50.96	48.33<br>(R3)	42.28<br>(R3)
3-mer	40.06<br>(R2)		54.53	41.10<br>(R2)	46.90	38.45<br>(R2)
4-mer	42.18	82.77	46.63	40.04	37.42	36.54
5-mer		55.49	41.76	37.93	36.84	
6-mer		53.68	39.07		35.60	
7-mer		53.93	37.63			

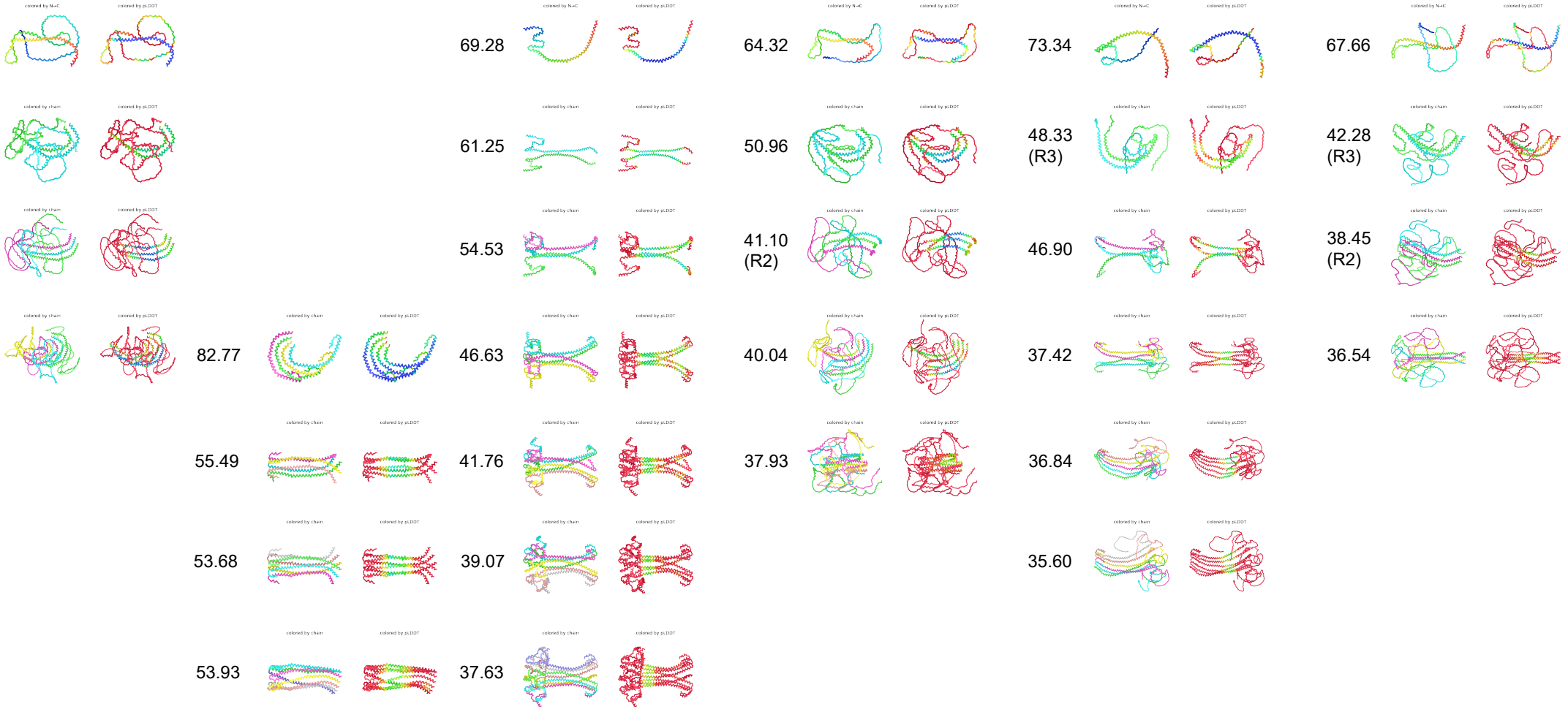

Oscarella carmela

EC368417.1

EC368417.1
(22-160)

	pLDDT		*pLDDT*	
1-mer	75.60	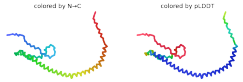		
2-mer	73.64	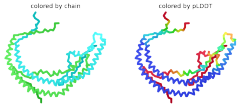		
3-mer	73.41	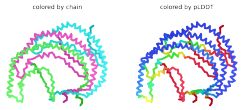		
4-mer	72.32	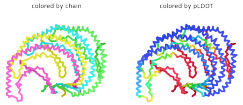		
5-mer	71.13	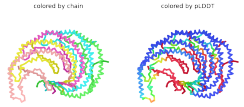		
6-mer	69.85	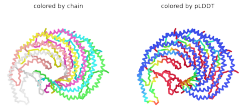	78.14	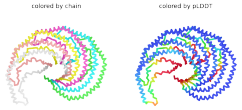
7-mer	68.87	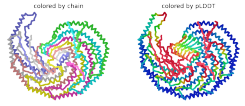	70.80<br>(R3)	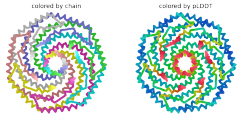
8-mer	56.33	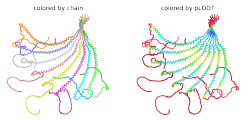	75.50	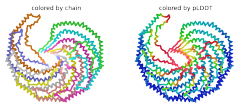
9-mer			59.62	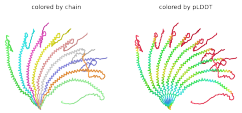

Trichoplax adhaerens

B3RWV2

B3RWV2 (1-151)

	pLDDT		*pLDDT*	
1-mer	84.57	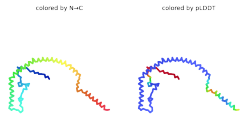		
2-mer	77.88	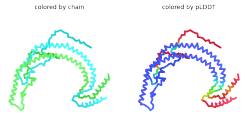		
3-mer	75.07	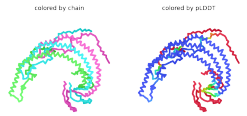		
4-mer	74.30	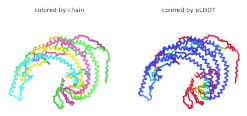		
5-mer	73.37	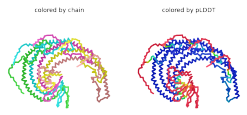		
6-mer	70.10	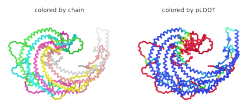		
7-mer	69.58	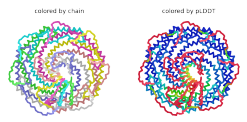	75.76 (R2)	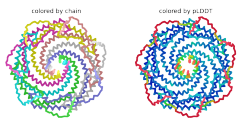
8-mer			77.55	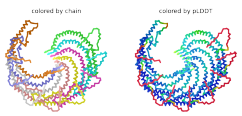

Stylophora pistillata

	A0A2B4SEV1		A0A2B4SAZ9	
	pLDDT		*pLDDT*	
1-mer	80.64	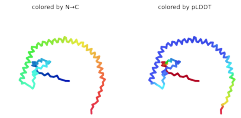	88.47	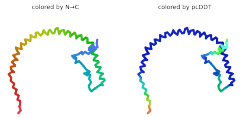
2-mer	79.69	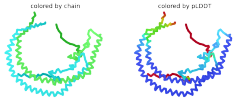	87.52	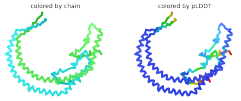
3-mer	82.25	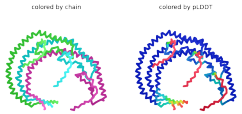	87.38	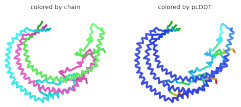
4-mer	80.80	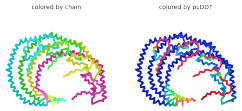	85.36	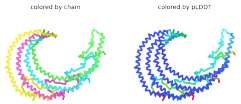
5-mer	80.20	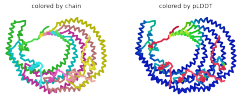	83.95	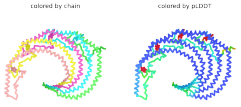
6-mer	60.22 (R5)	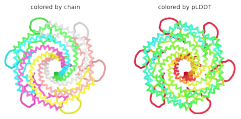	80.22	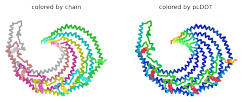
7-mer	71.63 (R3)	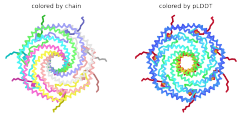	71.44	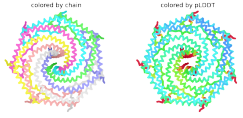
8-mer	76.17	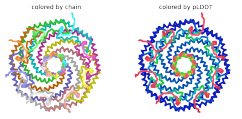	78.22	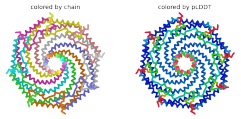
9-mer			79.52	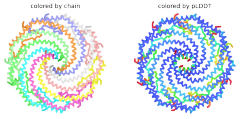

Homo sapiens

	**Q03135（CAV1）**		Q03135 32-178 (Beta-CAV1)		**P51636 (CAV2)**		**P51636 (CAV2)**		**P56539 (CAV3)**	
	pLDDT		*pLDDT*		*pLDDT*		*pLDDT*		*pLDDT*	
1-mer	78.74	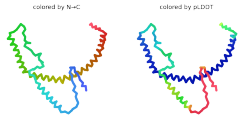	85.84	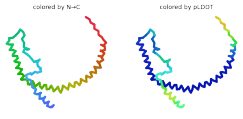	78.75	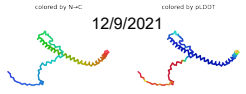 12/9/2021	78.42	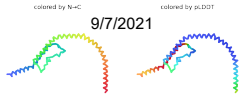 9/7/2021	89.43	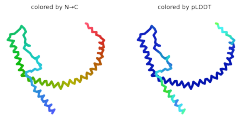
2-mer	71.65	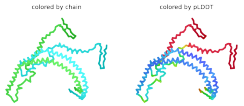	80.75	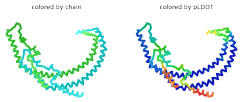	77.96	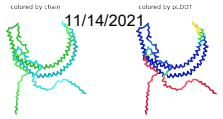 11/14/2021	78.05	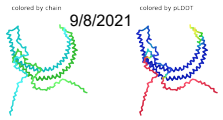 9/8/2021	86.34	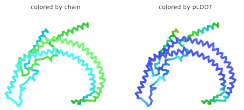
3-mer	72.78	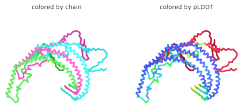	83.56	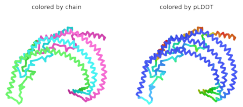	78.13	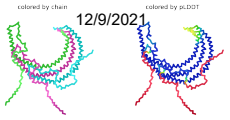 12/9/2021	78.22	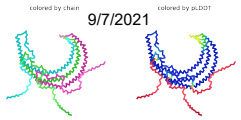 9/7/2021	86.93	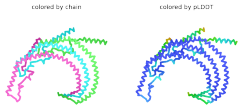
4-mer	72.63	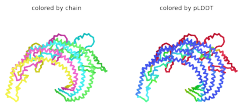	83.92	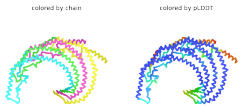	77.83	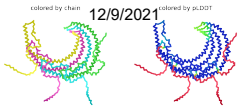 12/9/2021	78.28	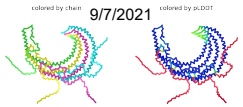 9/7/2021	86.56	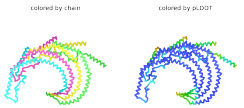
5-mer	73.18	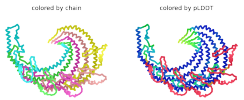	83.13	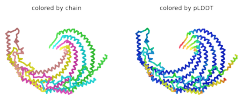	77.62	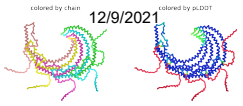 12/9/2021	76.36	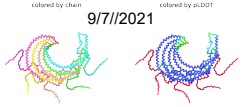 9/7//2021	86.01	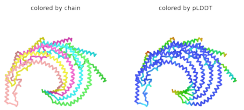
6-mer	69.58	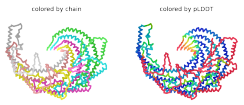	67.52 (R4)	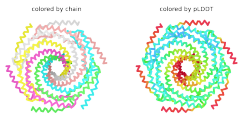	73.79	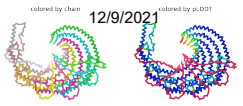 12/9/2021	74.37	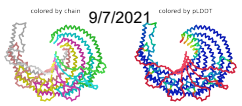 9/7/2021	85.48	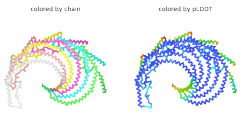
7-mer	68.40 (R2)	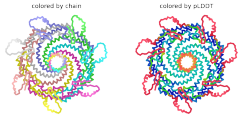	77.40 (R2)	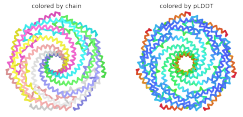	67.51	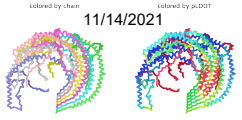 11/14/2021	73.70	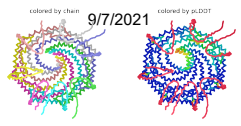 9/7/2021	80.75 (R2)	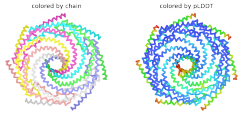
8-mer			80.60 (R2)	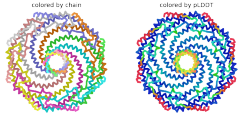	58.30	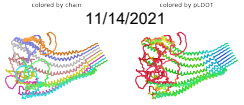 11/14/2021	58.30	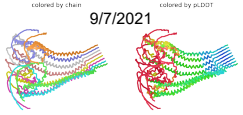 9/7/2021	80.57	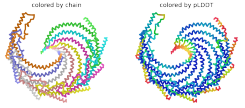
8-mer			64.71	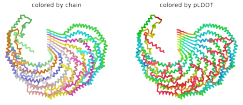						

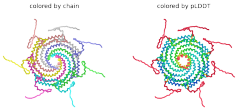
65.78(R3)

Strongylocentrotus purpuratus

	**A0A7M7HEM1**	A0A7M7HEM1 (40-192)	**A0A7M7GJ11**	A0A7M7GJ11 (48-196)	**A0A7M7HH57**	A0A7M7HH57 (53-218)
	pLDDT	*pLDDT*	*pLDDT*	*pLDDT*	*pLDDT*	*pLDDT*
1-mer	71.52		77.68		67.60	
2-mer	63.88		74.03		58.89	
3-mer	64.72		73.39		57.35	
4-mer	65.88		72.43		56.15	
5-mer	62.42		71.39		53.61	
6-mer	60.00		67.54		52.04	
7-mer		65.02 (R2)		80.10 (R2)		62.06
8-mer		70.13		81.94 (R2)		58.49

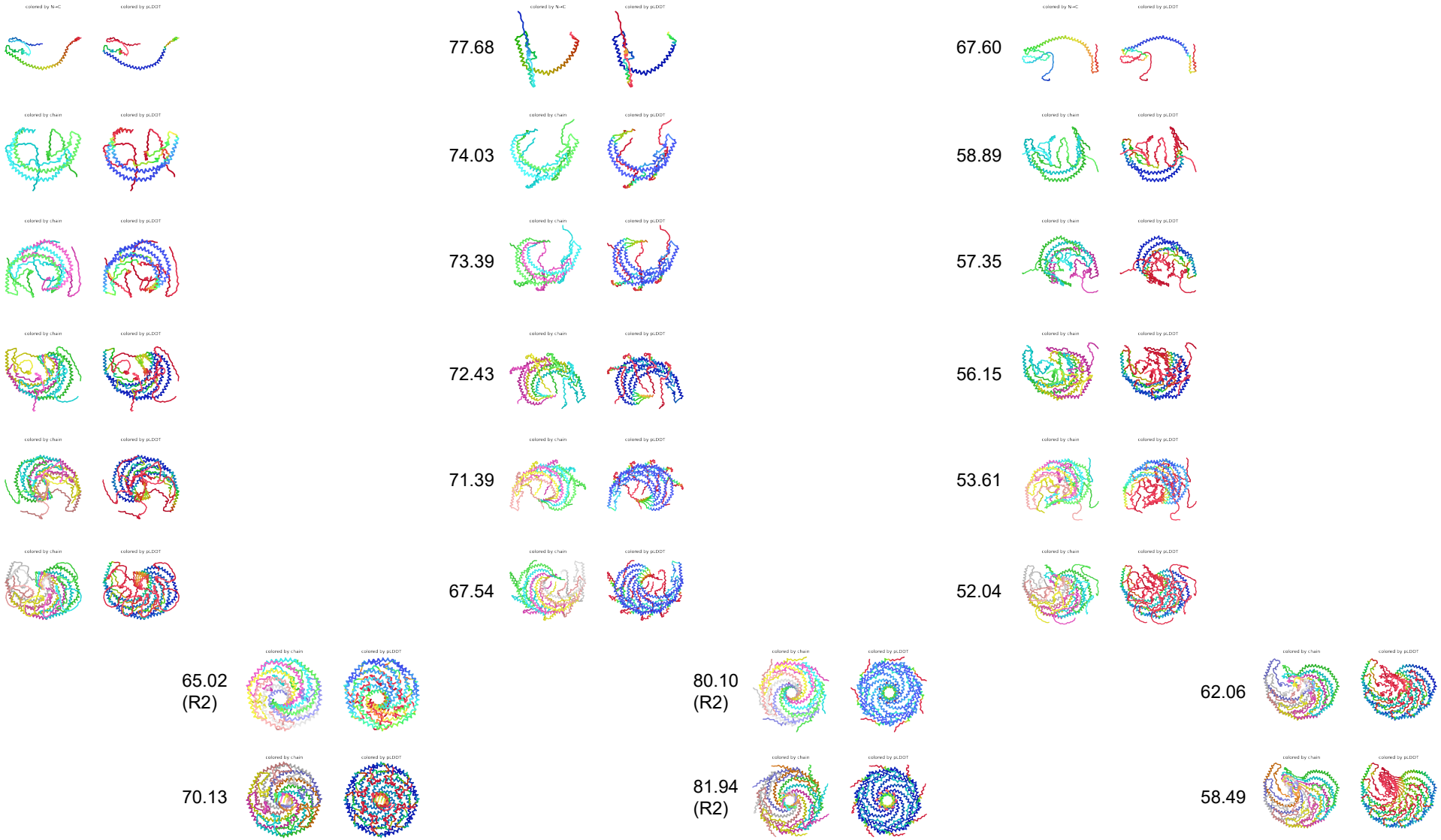

Strongylocentrotus purpuratus

	A0A7M7RAC4		A0A7M7T4C2		A0A7M7P0X0		A0A7M7G9F7		A0A7M7PLL3	
	pLDDT		*pLDDT*		*pLDDT*		*pLDDT*		*pLDDT*	
1-mer	86.56	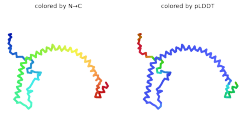	75.62	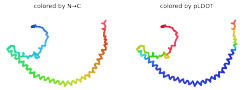	77.44	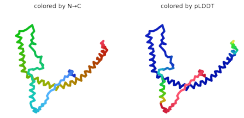	74.60	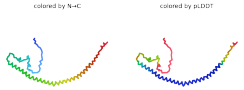	76.79	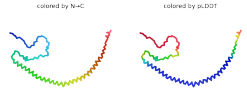
2-mer	84.35	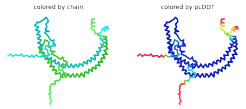	66.51	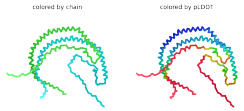	75.56	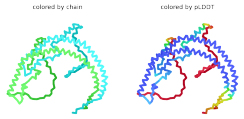	66.39	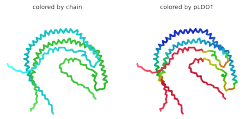	69.26	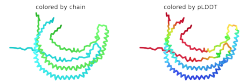
3-mer	85.09	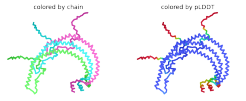	65.39	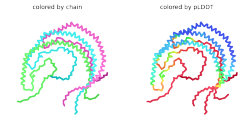	75.46	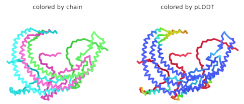	64.79	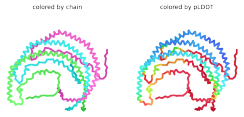	68.09	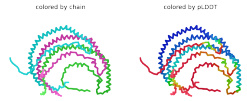
4-mer	84.47	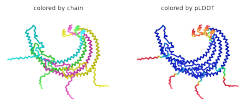	64.73	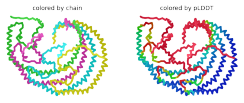	74.73	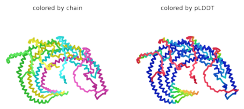	62.76	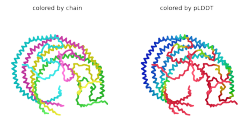	67.23	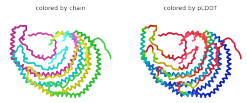
5-mer	83.58	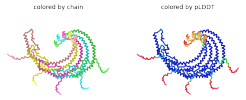	67.18	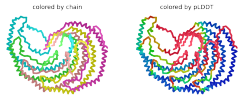	74.92	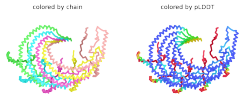	62.72	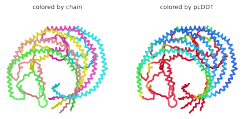	66.90	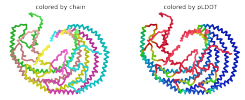
6-mer	68.55 (R4)	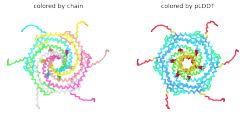	47.44 (R2)	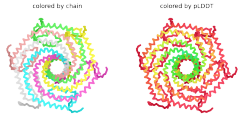	67.05 (R2)	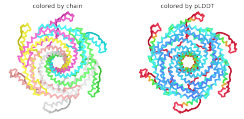	48.11 (R2)	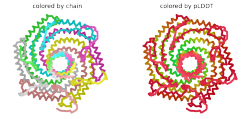	62.18	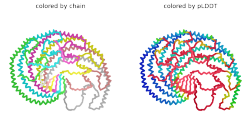
7-mer	72.61 (R2)	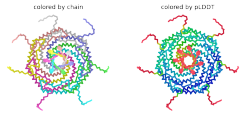	56.63 (R2)	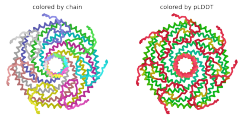	70.19 (R2)	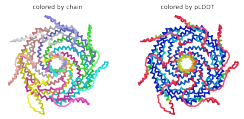	58.15 (R2)	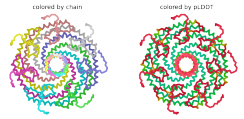	59.98	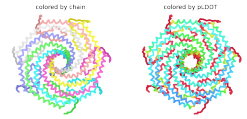
8-mer	75.00	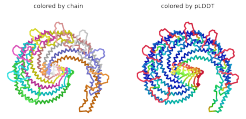	63.56	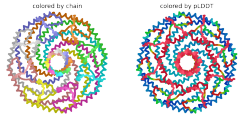			61.97	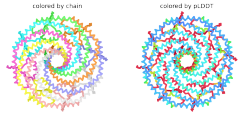	64.18	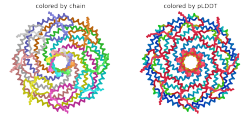

Priapulus caudatus

	XP_014678552.1		XP_014664738.1	
	pLDDT		*pLDDT*	
1-mer	92.04	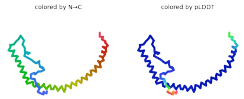	63.94	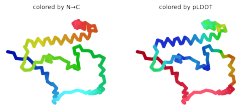
2-mer	87.97	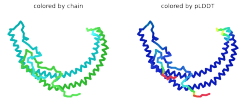	51.44	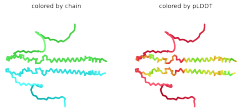
3-mer	88.48	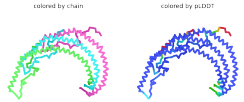	52.25	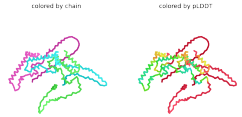
4-mer	87.26	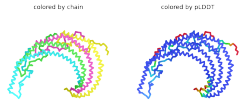	48.50	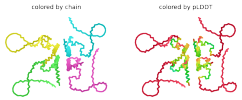
5-mer	86.68	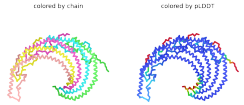	47.72	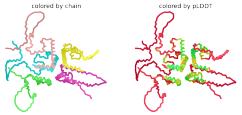
6-mer	72.19 (R3)	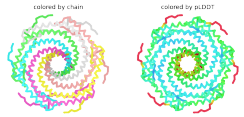	47.78	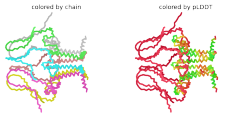
7-mer	77.05 (R2)	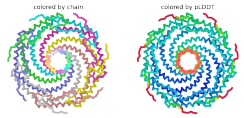	45.26	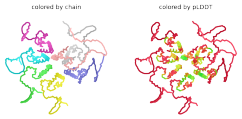
8-mer	83.06	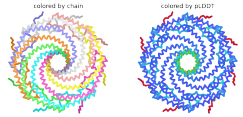	41.55	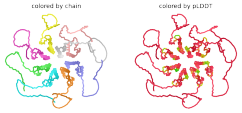
9-mer	48.14	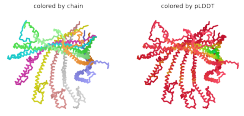		

Apis mellifera

A0A7M7R2L2

A0A7M7GWE0
A0A7M7R2L2 (24-177)

	pLDDT		*pLDDT*	
1-mer	80.39	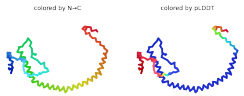		
2-mer	72.87	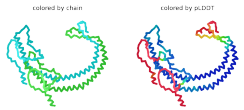		
3-mer	74.73	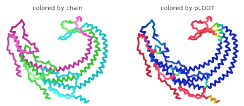		
4-mer	74.72	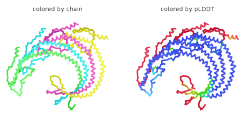		
5-mer	74.02	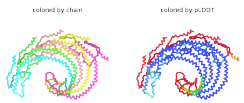		
6-mer	73.72	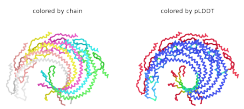		
7-mer	65.85	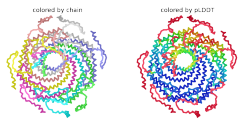	70.61 (R2)	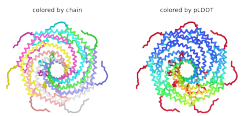
8-mer			76.08	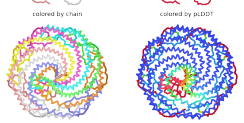

Caenorhabditis elegans

Q18879

Q18879（192-351）

Q94051

H2L2G6 Q94051（110-235）

	Q18879 *pLDDT*	Q18879（192-351） *pLDDT*	Q94051 *pLDDT*	H2L2G6 Q94051（110-235） *pLDDT*
1-mer	57.76 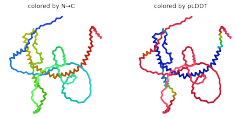		68.02 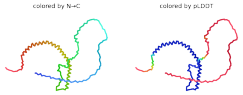	
2-mer	54.24 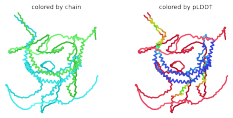		63.28 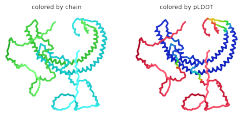	
3-mer	51.82 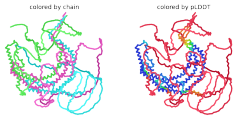		63.32 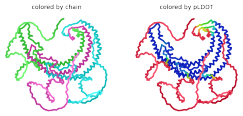	
4-mer		79.74 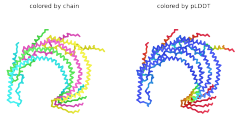	61.95 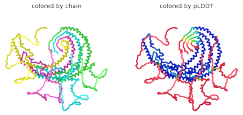	
5-mer		80.34 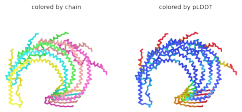	59.08 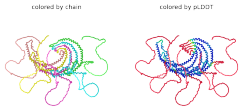	
6-mer		67.27 (R4) 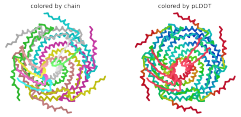		83.91 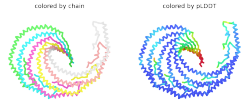
7-mer		74.16 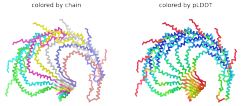		73.61 (R4) 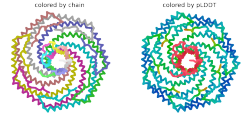
8-mer		61.28 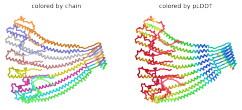		79.90 (R2) 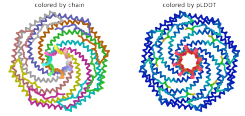
9-mer				74.55 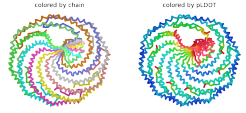
10-mer				64.11 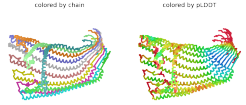

Brachionus plicatilis

	A0A3M7SAK0	A0A3M7SAF6	A0A3M7SAF6 (1-132)	A0A3M7RD35	A0A3M7RCJ9	A0A3M7SB10	A0A3M7Q0Q1
	pLDDT	*pLDDT*	*pLDDT*	*pLDDT*	*pLDDT*	*pLDDT*	*pLDDT*
1-mer	87.10	82.78		92.58	90.03	86.77	92.75
2-mer	82.46	76.09		87.69	85.27	80.28	85.81
3-mer	81.39	72.98		88.74	83.61	80.22	89.24
4-mer	80.98	71.70		87.76	83.17	79.85	87.42
5-mer	80.50	48.99 (R4)		84.76	82.73	79.84	86.84
6-mer	75.65	68.53		73.01 (R4)	69.33 (R4)	68.47 (R4)	75.12 (R2)
7-mer	74.14 (R2)	67.40	73.48 (R2)	78.86	75.24 (R2)	72.45 (R2)	79.72
8-mer	77.24		43.14	82.37	78.78	75.94	82.50
9-mer				82.97			83.19

Macrostomum lignano

	A0A267DFI9	A0A267DC90	A0A267G3E9	A0A267G3E9 (94-239)	A0A267G387	A0A1I8H2T4	A0A1I8J741
	pLDDT	*pLDDT*	*pLDDT*	*pLDDT*	*pLDDT*	*pLDDT*	*pLDDT*
1-mer	81.25	69.11	68.19		86.88	90.56	91.82
2-mer	81.08	56.05	65.30		84.62	85.81	88.21
3-mer	77.90	52.91	64.88		83.63	86.70	84.55
4-mer	76.51	48.76	64.06		81.44	86.84	85.62
5-mer	75.70	42.36	62.85		80.54	86.24	86.27
6-mer	74.27	40.78		69.53 (R3)	79.54	66.97 (R4)	65.82 (R4)
7-mer	71.10	37.40		77.11 (R2)	71.08 (R2)	71.43	73.22 (R2)
8-mer				75.89	74.09 (R2)	81.22	81.82
9-mer				60.63		69.56	78.52

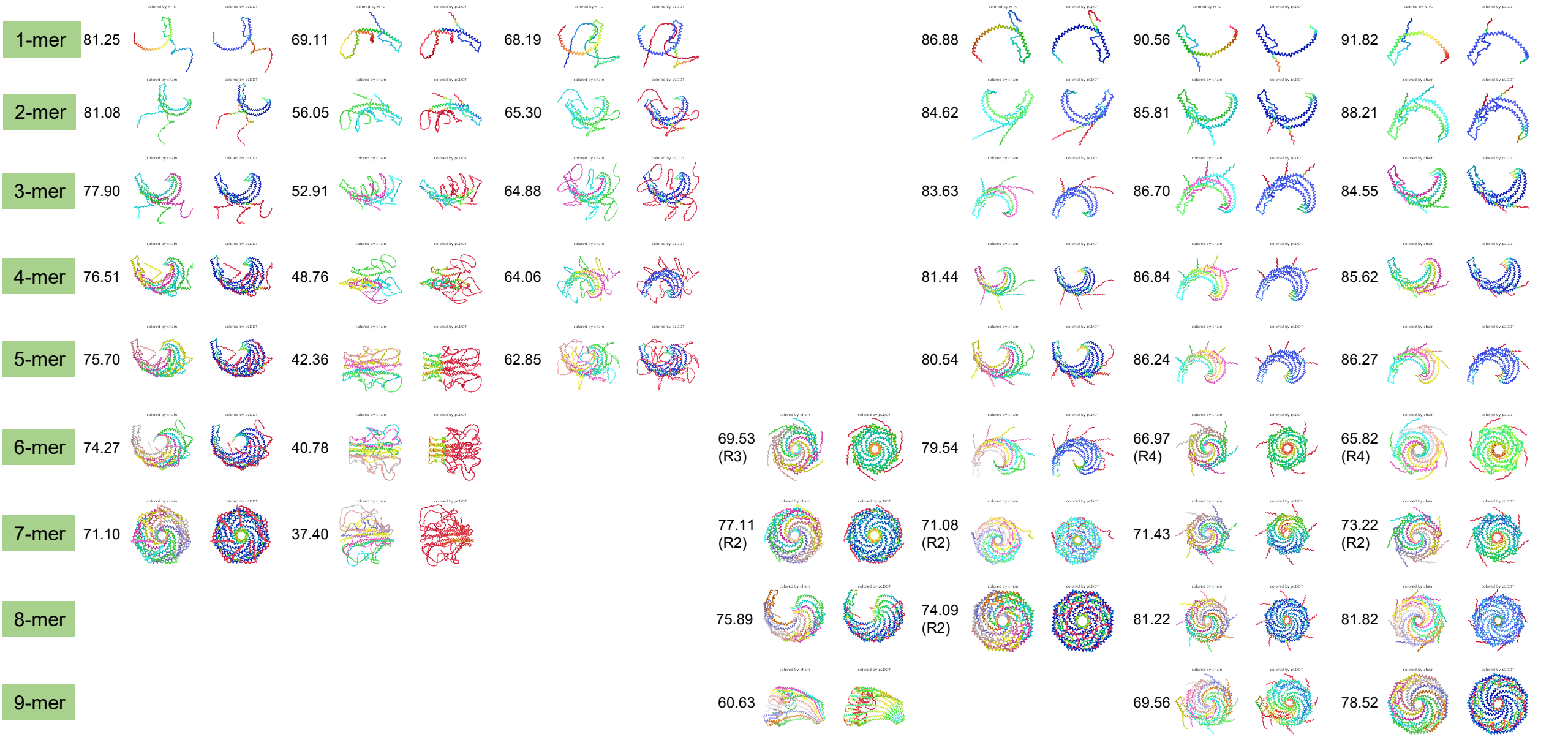

Macrostomum lignano

	A0A267G1N3	A0A267DS21	A0A267DU21	A0A267GQL3	A0A267GQL3 (70-221)	A0A267G3D8	A0A1I8GQY0
	pLDDT	*pLDDT*	*pLDDT*	*pLDDT*	*pLDDT*	*pLDDT*	*pLDDT*
1-mer	70.70	91.04	91.65	70.38		86.00	80.01
2-mer	67.66	84.34	83.53	67.77		81.41	78.32
3-mer	66.66	85.29	86.99	66.43		84.06	78.15
4-mer	65.65	87.02	87.12	65.99		82.84	78.03
5-mer	65.11	83.66	86.48	54.51 (R5)		80.31	61.93 R4
6-mer	57.02 (R2)	68.64 (R2)	68.27 (R4)		67.83 (R3)	67.54 (R4)	74.72
7-mer	62.07	73.78 (R2)	73.60 (R2)		73.09 (R2)	70.81 (R2)	72.76 (R2)
8-mer		81.20	82.23		79.09 (R2)	74.80 (R2)	
9-mer		80.80	81.09				

Macrostomum lignano

	A0A267EVV7	A0A267DNA3	A0A267DNA3 (69-221)	A0A267F6W6	A0A267FNG7	A0A1I8GFP4	A0A1I8J849
	pLDDT	*pLDDT*	*pLDDT*	*pLDDT*	*pLDDT*	*pLDDT*	*pLDDT*
1-mer	86.25	70.34		92.13	91.42	84.58	79.45
2-mer	79.71	67.52		84.98	86.06	73.86	74.12
3-mer	81.58	66.79		87.18	85.55	78.40	75.55
4-mer	80.37	65.78		88.11	85.58	78.63	75.04
5-mer	78.45	55.22 (R5)		87.92	86.07	78.50	73.65
6-mer	60.77		81.73	84.67	85.64	72.65	61.99 (R3)
7-mer	69.98 (R2)		74.54	75.53 (R2)	76.64 (R2)	70.79 (R2)	65.26
8-mer				83.80	81.10	67.84	
9-mer				55.89	65.07 (R2)		

Macrostomum lignano

	A0A1I8HHY8		A0A1I8ILQ8		A0A267DBT9		A0A267F2S4		A0A267FMS2		A0A1I8H327	
	pLDDT		*pLDDT*		*pLDDT*		*pLDDT*		*pLDDT*		*pLDDT*	
1-mer	91.05	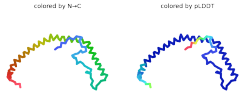	89.47	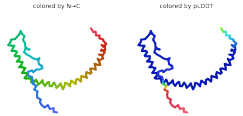	91.97	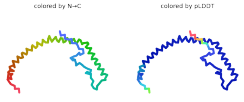	92.13	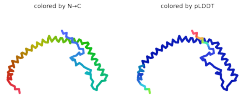	85.04	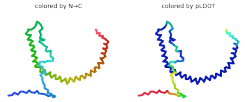	87.80	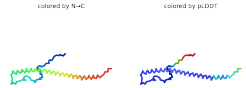
2-mer	87.07	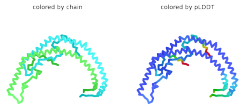	84.92	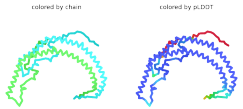	87.83	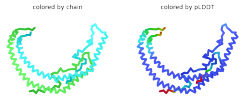	89.62	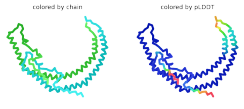	83.07	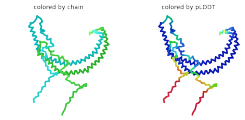	72.48	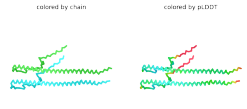
3-mer	88.45	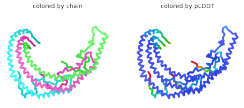	85.73	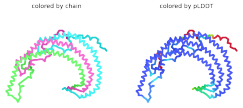	89.22	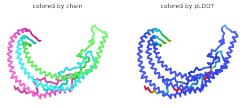	88.17	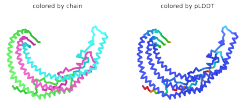	82.25	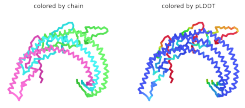	61.33	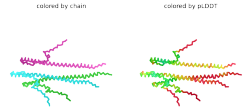
4-mer	87.04	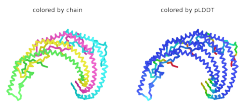	85.42	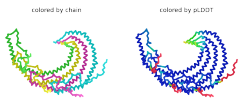	88.29	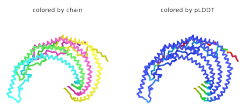	88.27	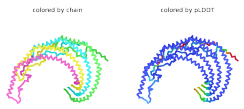	79.79	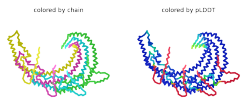	56.07	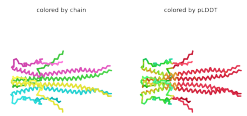
5-mer	87.59	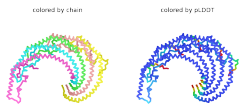	84.51	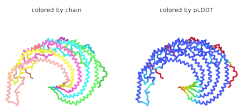	87.82	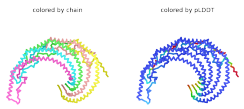	88.48	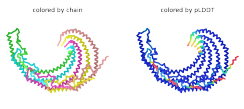	78.59	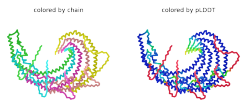	57.23	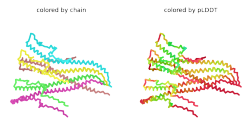
6-mer	66.71 (R3)	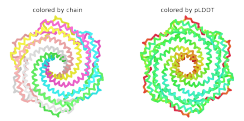	68.30 (R4)	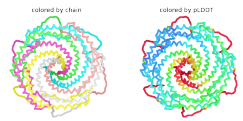	65.06 (R3)	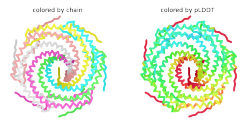	87.59	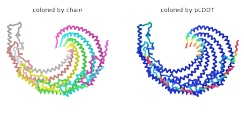	74.81	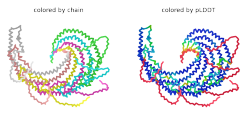	49.18	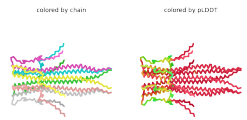
7-mer	85.47	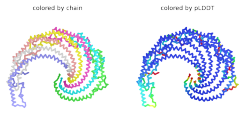	76.65 (R2)	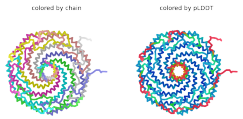	84.93	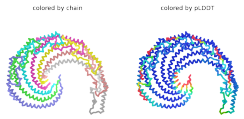	77.34 (R2)	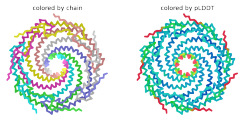	71.65 (R2)	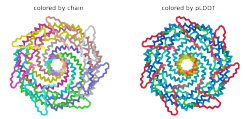	51.47	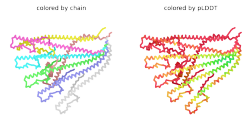
8-mer	84.65	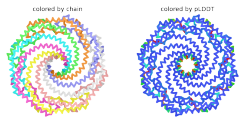	62.06	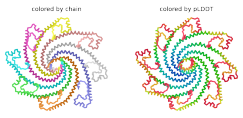	83.89	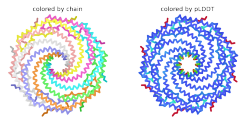	83.62	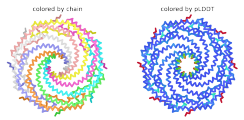	62.40	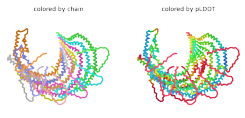	62.52	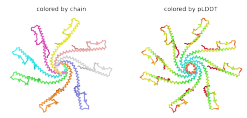
9-mer	51.78	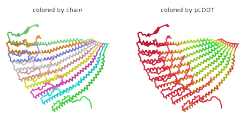			48.53	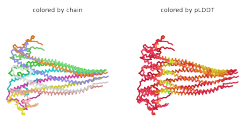	50.50	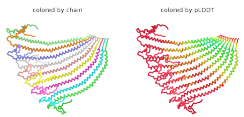			51.88	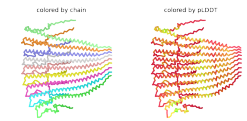

Macrostomum lignano

	A0A1I8ID24			A0A1I8G233		
	pLDDT	colored by N→C	colored by pLDDT	*pLDDT*	colored by N→C	colored by pLDDT
1-mer	93.29	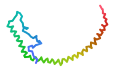	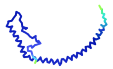	93.09	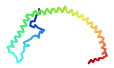	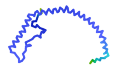
		colored by chain	colored by pLDDT		colored by chain	colored by pLDDT
2-mer	83.86	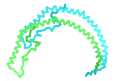	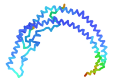	91.90	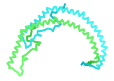	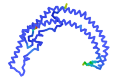
		colored by chain	colored by pLDDT		colored by chain	colored by pLDDT
3-mer	88.69	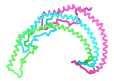	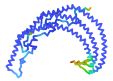	89.88	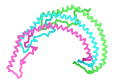	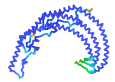
		colored by chain	colored by pLDDT		colored by chain	colored by pLDDT
4-mer	88.52	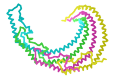	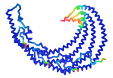	89.12	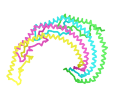	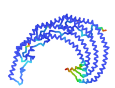
		colored by chain	colored by pLDDT		colored by chain	colored by pLDDT
5-mer	88.54	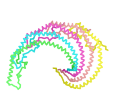	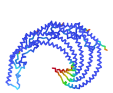	89.29	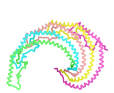	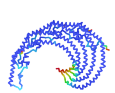
		colored by chain	colored by pLDDT		colored by chain	colored by pLDDT
6-mer	85.04	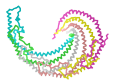	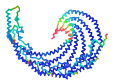	66.35 (R4)	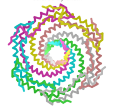	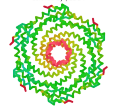
		colored by chain	colored by pLDDT		colored by chain	colored by pLDDT
7-mer	73.63 (R2)	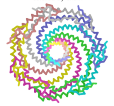	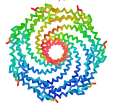	72.50 (R2)	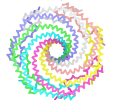	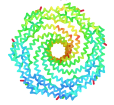
		colored by chain	colored by pLDDT		colored by chain	colored by pLDDT
8-mer	68.26	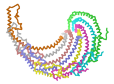	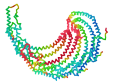	77.40 (R2)	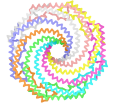	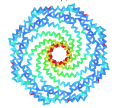
		colored by chain	colored by pLDDT		colored by chain	colored by pLDDT
9-mer	52.16	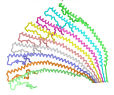	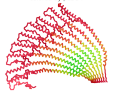	76.85	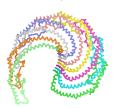	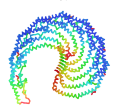
		colored by chain	colored by pLDDT		colored by chain	colored by pLDDT
10-mer	50.28	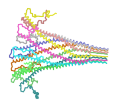	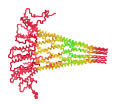	52.68	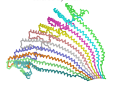	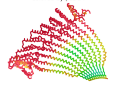

Pomacea canaliculata

	A0A2T7NUS2		A0A2T7NUA4		A0A2T7NUV7		A0A2T7NUT7	
	pLDDT		*pLDDT*		*pLDDT*		*pLDDT*	
1-mer	87.54	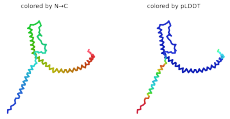	80.77	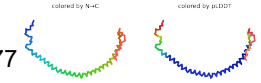	85.86	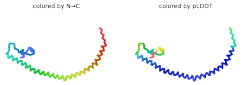	85.17	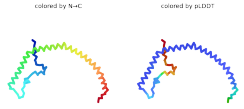
2-mer	82.04	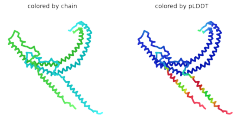	76.76	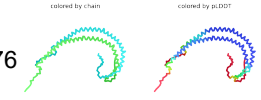	82.24	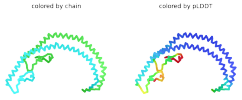	80.85	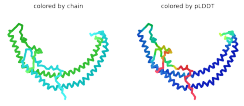
3-mer	81.44	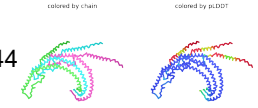	75.78	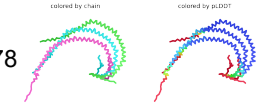	81.75	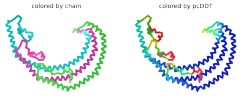	80.06	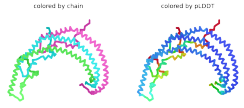
4-mer	80.94	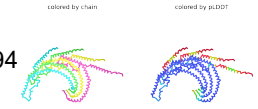	75.50	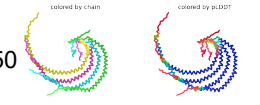	78.78	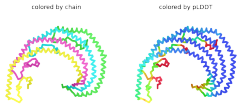	80.86	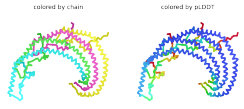
5-mer	79.31	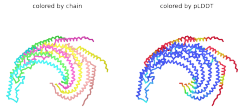	76.44	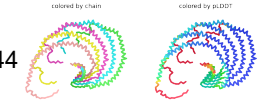	79.97	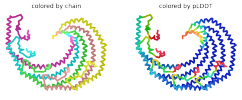	80.61	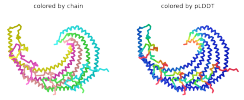
6-mer	55.24<br>(R2)	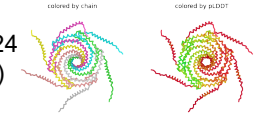	78.05	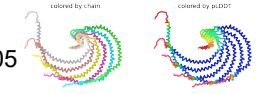	80.45	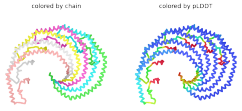	76.12	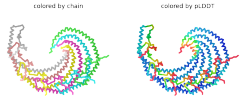
7-mer	55.29<br>(R2)	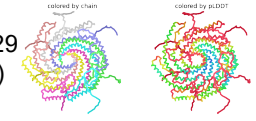	78.07	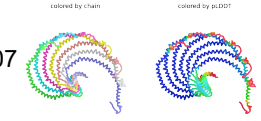	62.98	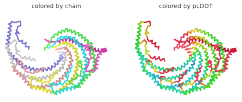	66.98	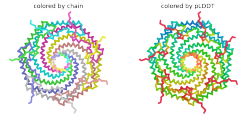
8-mer	51.03	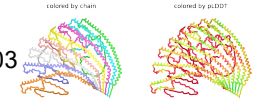	74.37	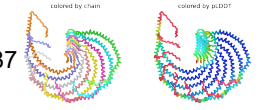	50.80	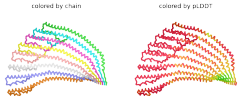	50.94	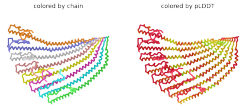
9-mer			71.66	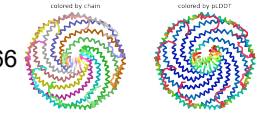	50.71	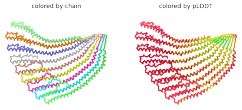	48.77	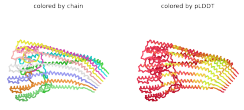
10-mer			66.02	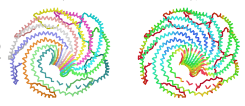	52.01	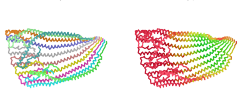		

Lingula unguis

	A0A1S3I469		A0A1S3IH41		A0A1S3IG42		A0A1S3JHP2		A0A1S3HK30		A0A1S3IG00		A0A1S3I8M7	
	pLDDT		*pLDDT*		*pLDDT*		*pLDDT*		*pLDDT*		*pLDDT*		*pLDDT*	
1-mer	85.99	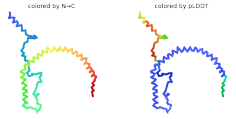	92.28	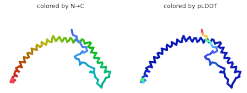	92.89	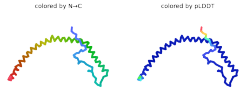	73.34	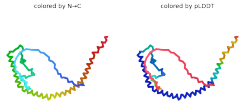	75.90	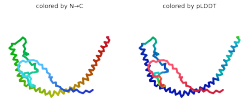	90.71	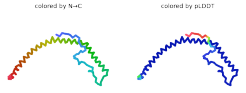	74.41	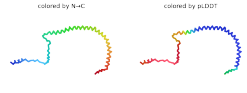
2-mer	81.14	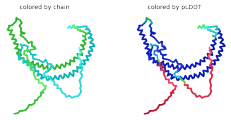	88.03	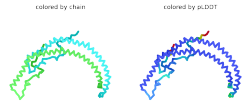	89.13	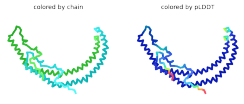	74.19	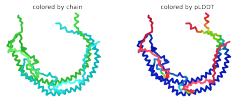	74.16	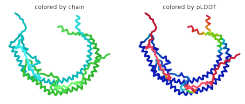	86.45	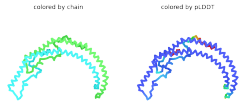	71.83	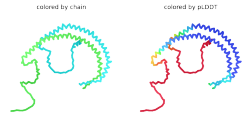
3-mer	80.93	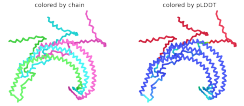	84.64	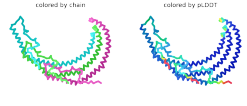	87.16	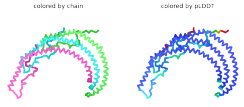	73.84	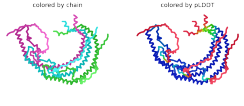	74.00	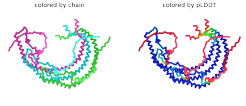	86.38	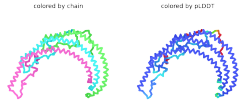	71.06	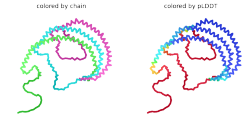
4-mer	80.47	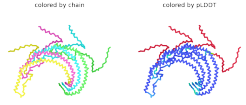	87.76	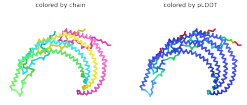	86.74	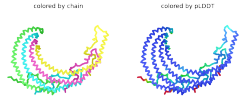	73.45	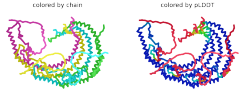	73.86	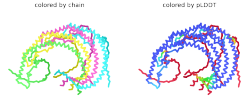	85.14	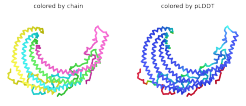	71.16	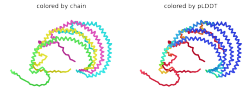
5-mer	79.51	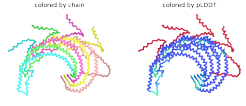	88.36	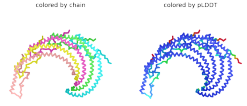	88.19	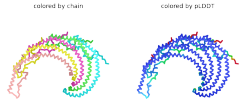	73.08	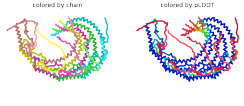	73.15	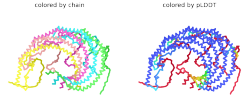	87.03	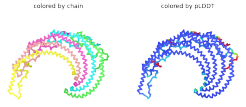	70.41	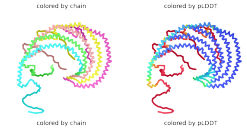
6-mer	65.86 (R3)	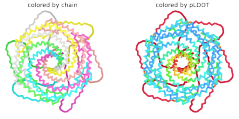	65.38 (R4)	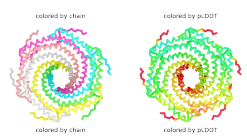	88.29	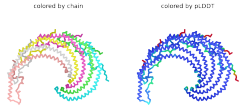	69.13	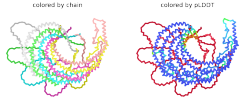	65.29 (R2)	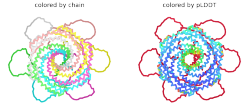	86.86	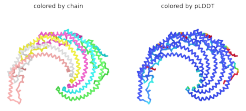	68.22	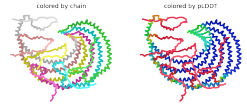
7-mer	69.32 (R2)	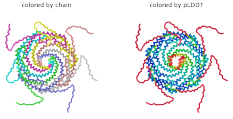	73.82 (R2)	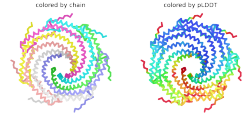	84.82	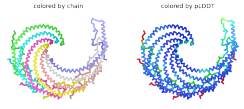	68.69	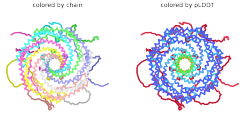	68.19 (R2)	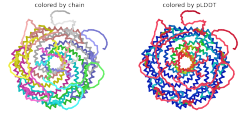	75.14 (R2)	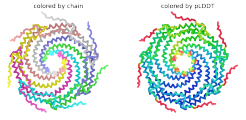	57.11	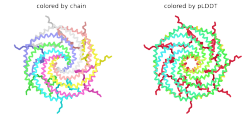
8-mer	61.24	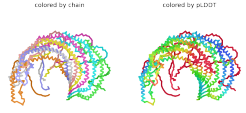	52.28	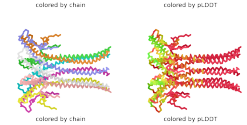	85.09	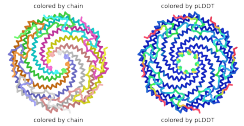					53.04	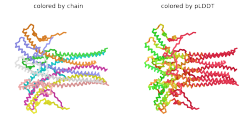	58.00	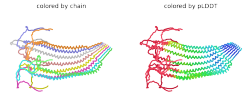
9-mer			49.61	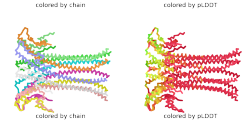	49.15	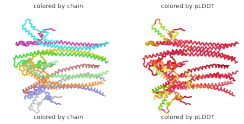								
10-mer			42.15	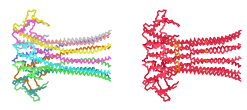	42.17	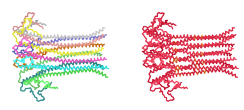								

Lingula unguis

	A0A1S3IQQ3		A0A1S3IXS7	
	pLDDT		*pLDDT*	
1-mer	94.45	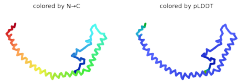	87.16	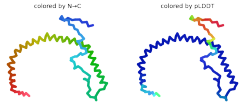
2-mer	92.96	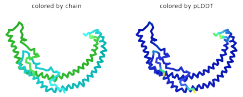	89.62	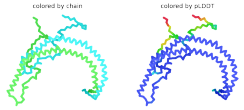
3-mer	91.70	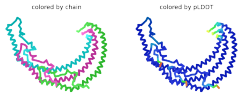	86.85	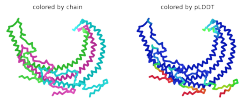
4-mer	89.61	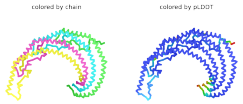	85.40	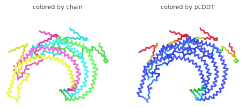
5-mer	90.29	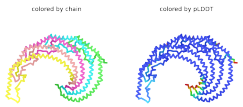	60.84 (R5)	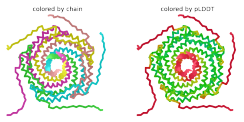
6-mer	89.08	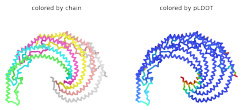	66.66 (R5)	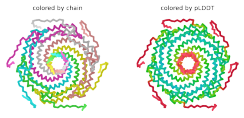
7-mer	75.25	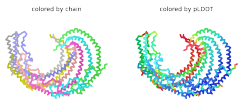	72.85 (R2)	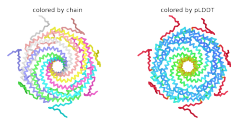
8-mer	54.57	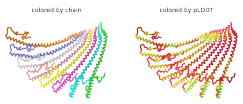	79.75	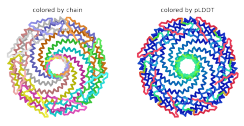

Capitella teleta

	R7U639		R7V531		R7VDS7		R7TPM2	
	pLDDT		*pLDDT*		*pLDDT*		*pLDDT*	
1-mer	73.81	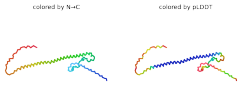	95.01	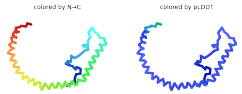	93.47	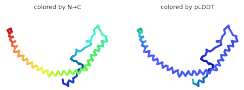	90.74	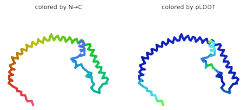
2-mer	52.32	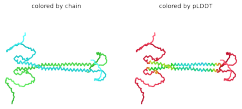	92.53	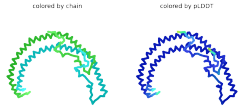	89.80	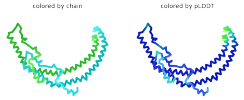	87.08	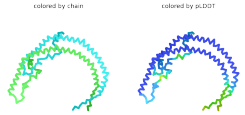
3-mer	42.62	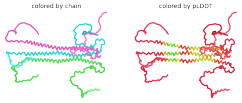	92.93	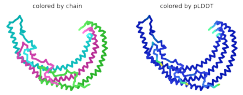	88.78	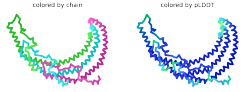	86.82	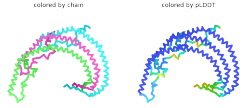
4-mer	36.23	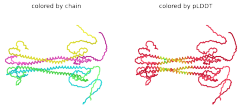	92.71	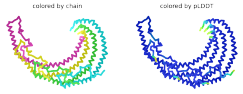	89.90	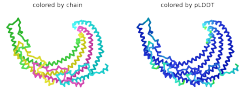	85.83	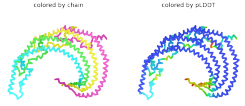
5-mer	35.00	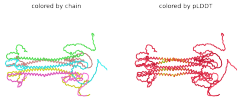	92.03	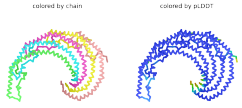	89.31	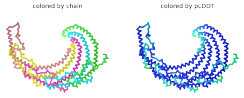	85.64	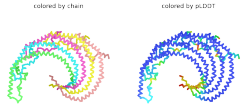
6-mer	35.33	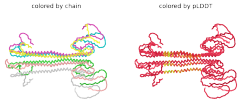	56.04 (R4)	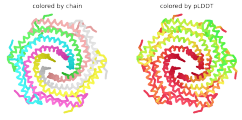	88.90	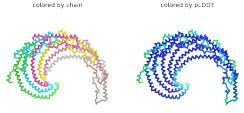	68.62 (R3)	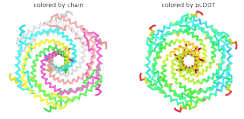
7-mer	33.30	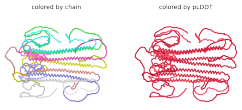	76.51 (R2)	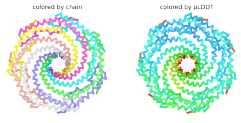	74.74 (R2)	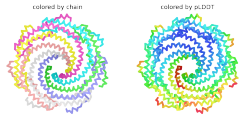	77.14 (R2)	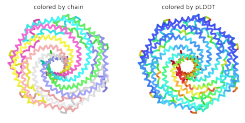
8-mer			89.18	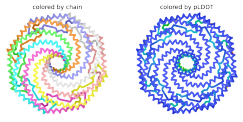	84.13	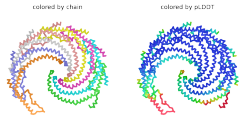	75.10	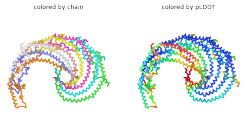
9-mer			53.60	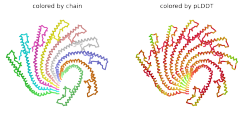	41.67	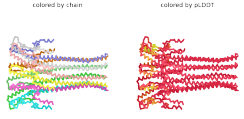	59.68	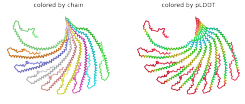
10-mer			53.35	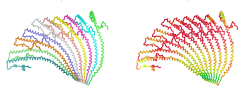	41.35	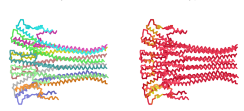		
