# Supplementary material for: Evolutionarily diverse caveolins share a common structural framework built around amphipathic disks
Source: J Cell Biol. 2025 Aug 7;224(9):e202411175. doi: 10.1083/jcb.202411175 (PMC12330381; doi:10.1083/jcb.202411175)

File S4. Predicted structures of caveolin monomers andoligomers using AlphaFold2.2.

Note:

Five models were generated for each prediction. For each prediction, the rank 1 model was displayed in ribbons/slabs mode in the left panel.

Models were all colored by pLDDT values as below color key indicated:

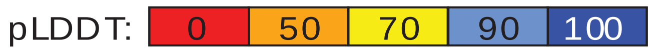

The PAE plots of all five models were displayed in the right panels.

F2U793 *S. Rosetta*

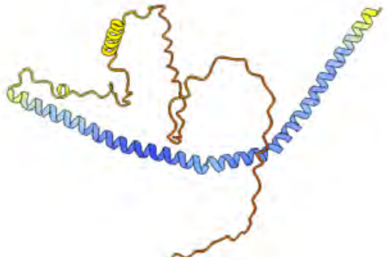

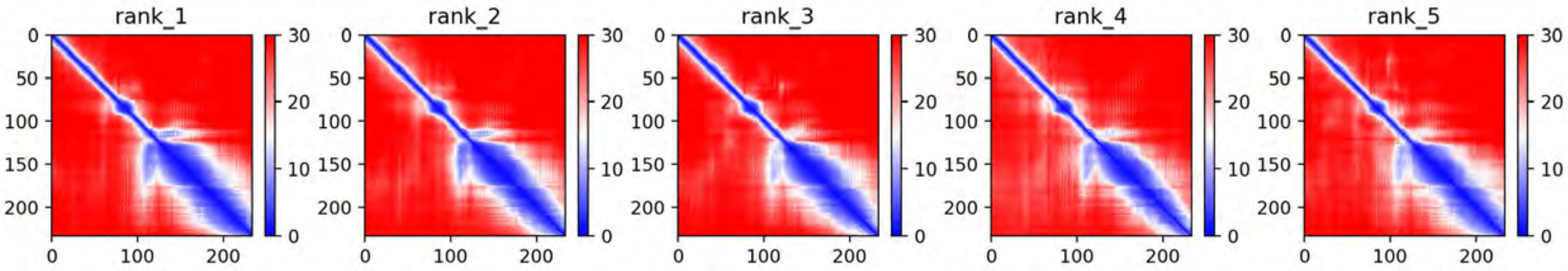

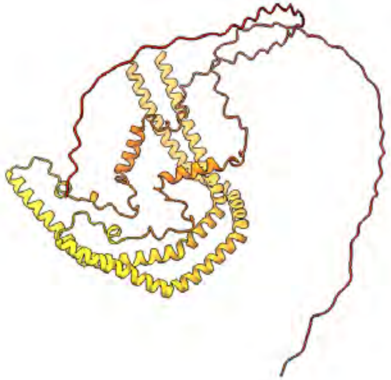

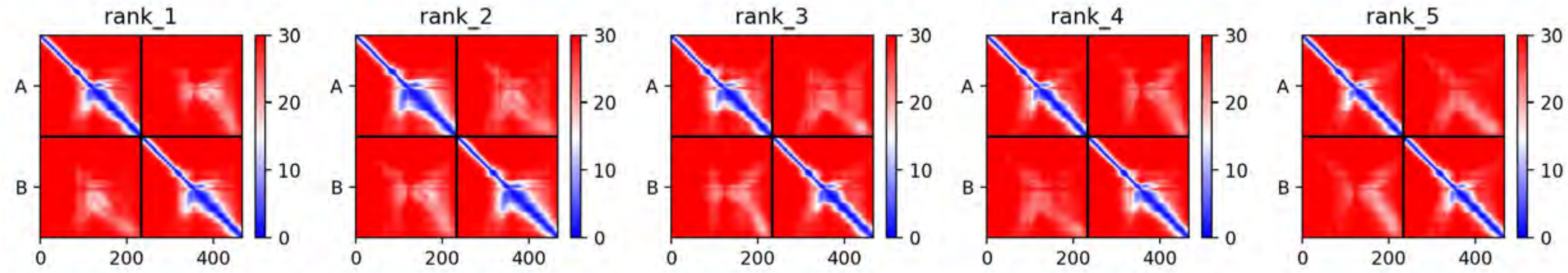

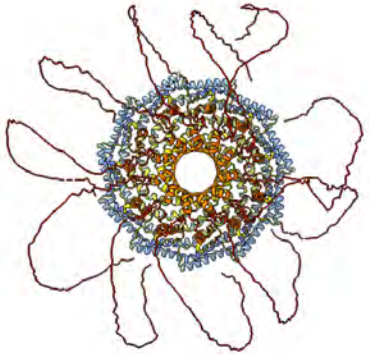

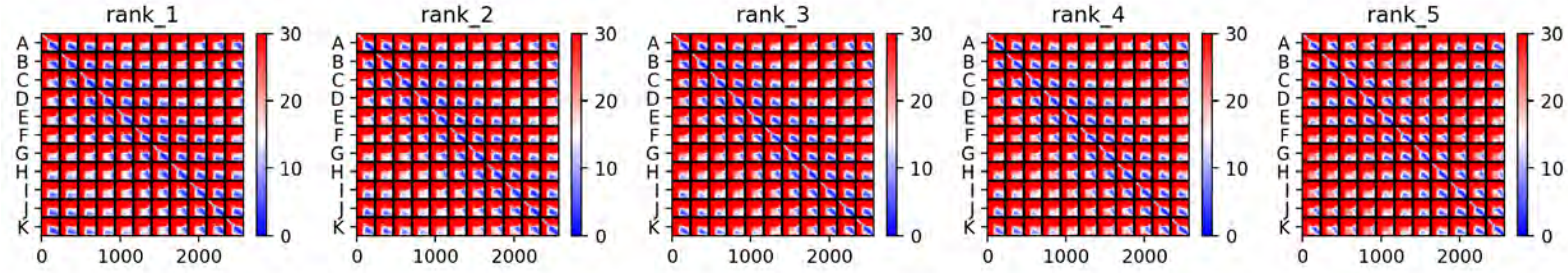

A0A1X7UHP5 ***A. queenslandica***

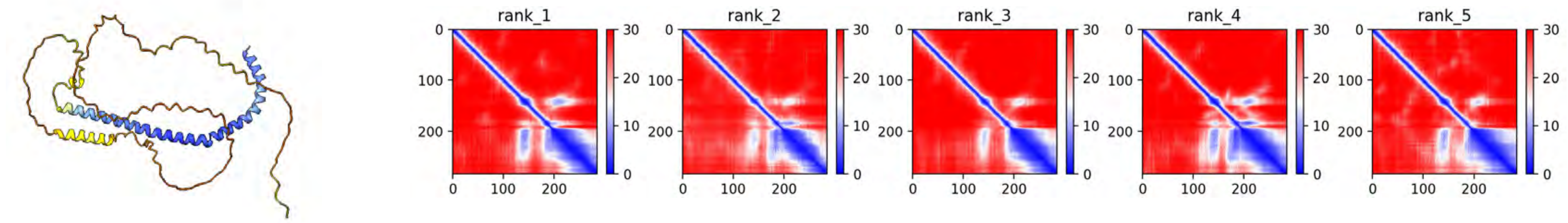

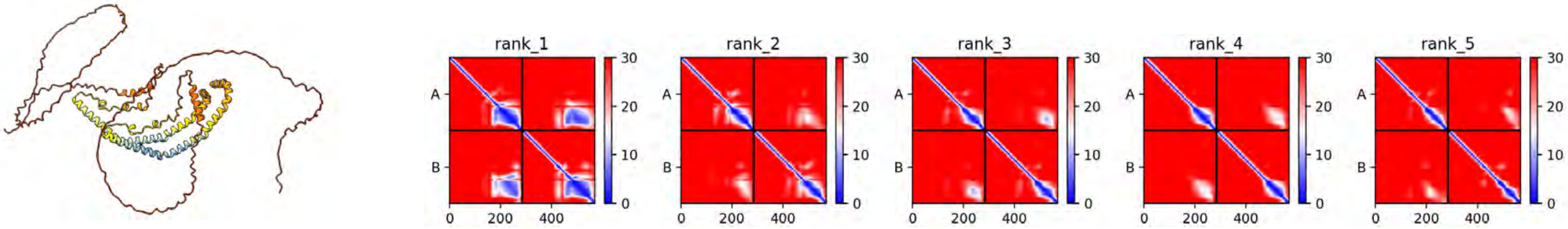

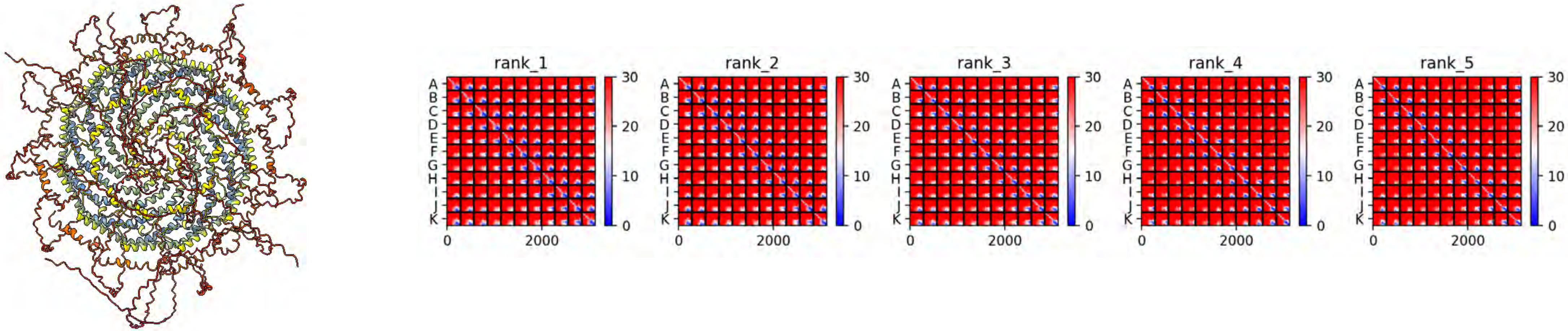

A0A1X7UGA1 ***A. queenslandica***

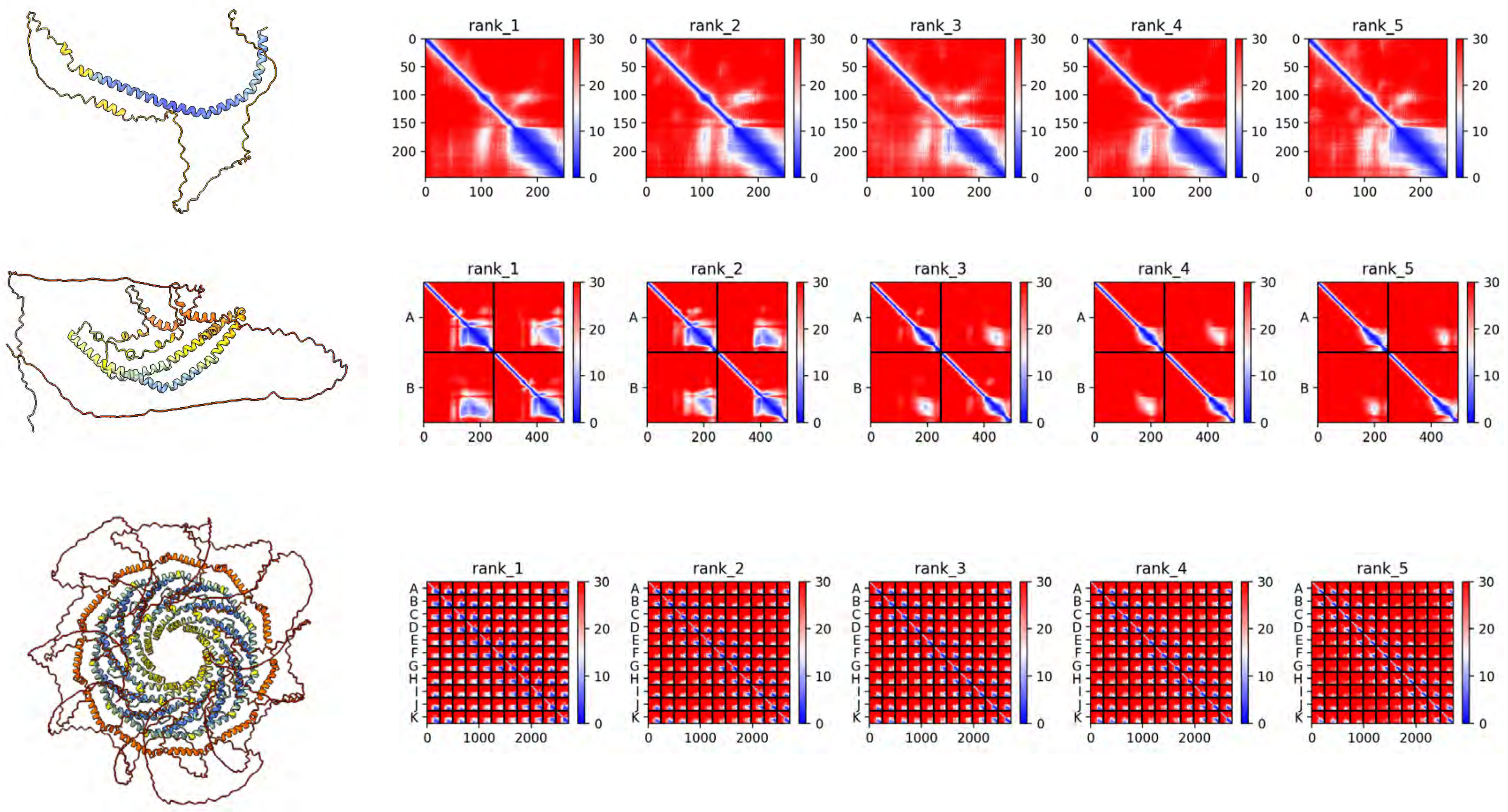

A0A1X7TMH4 *A. queenslandica*

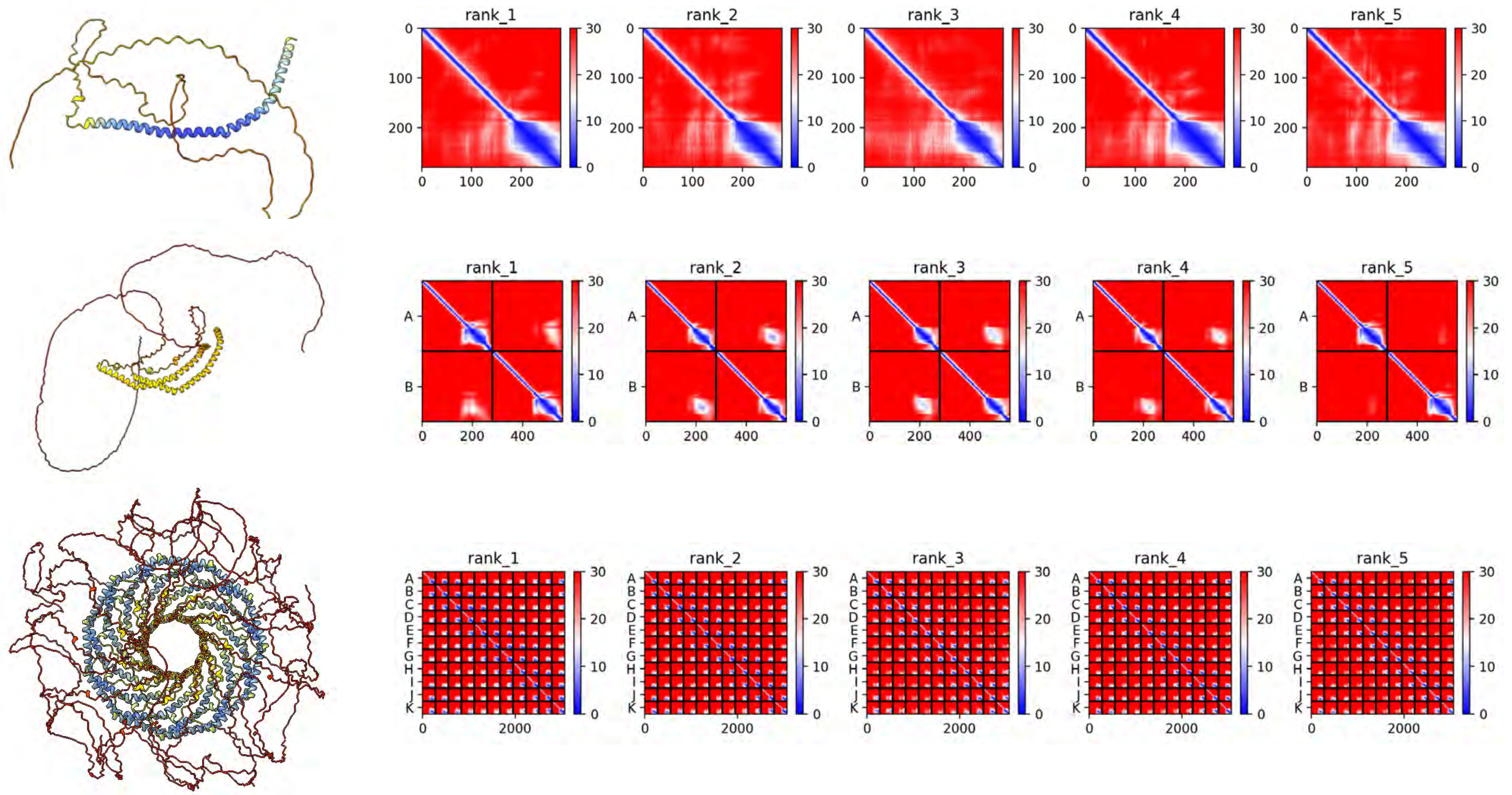

A0A1X7VPY7 *A. queenslandica*

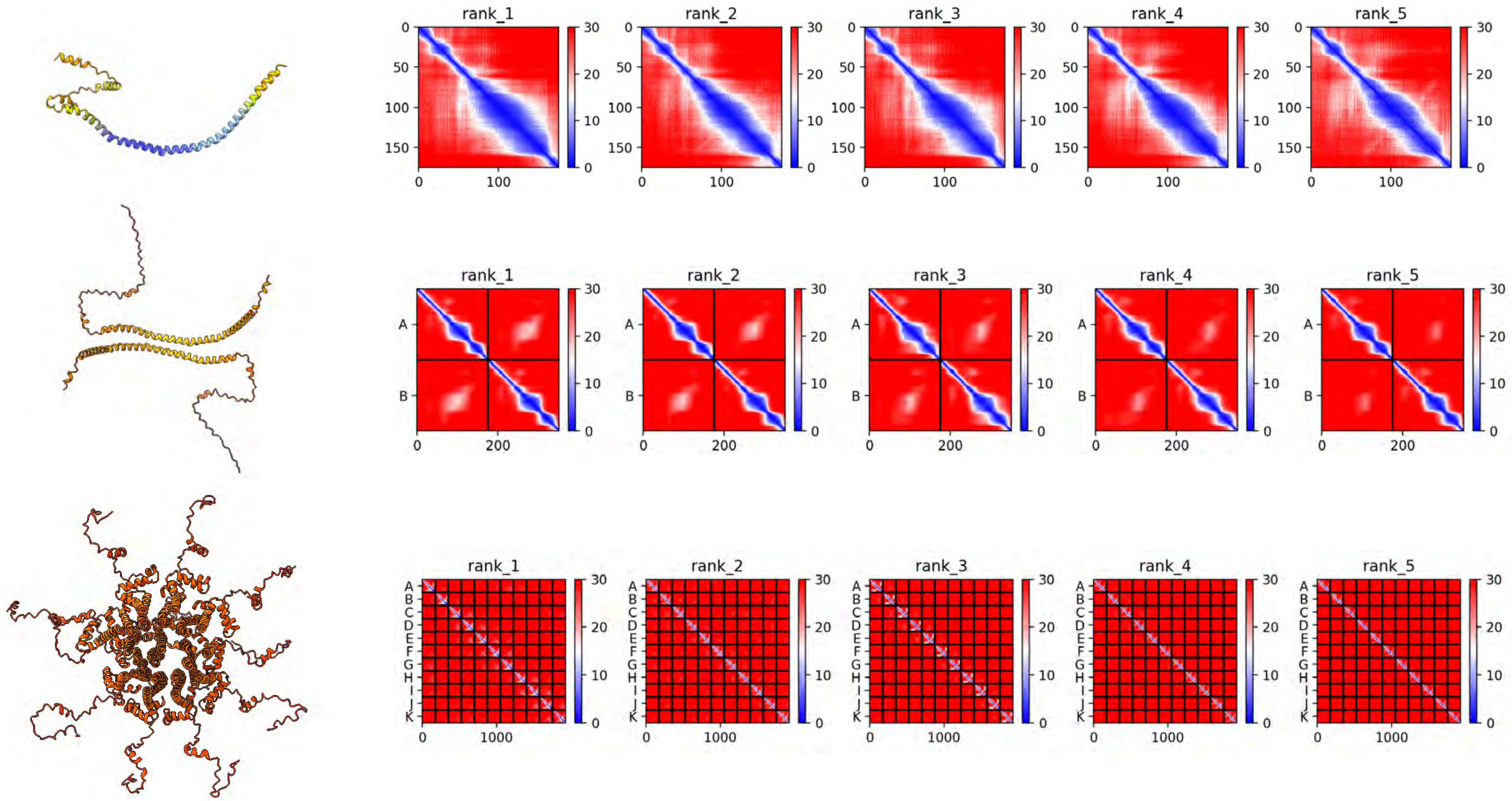

A0A1X7VRV8 *A. queenslandica*

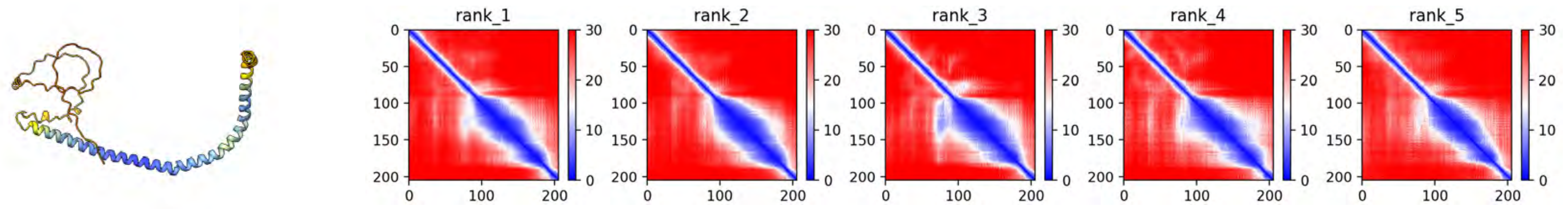

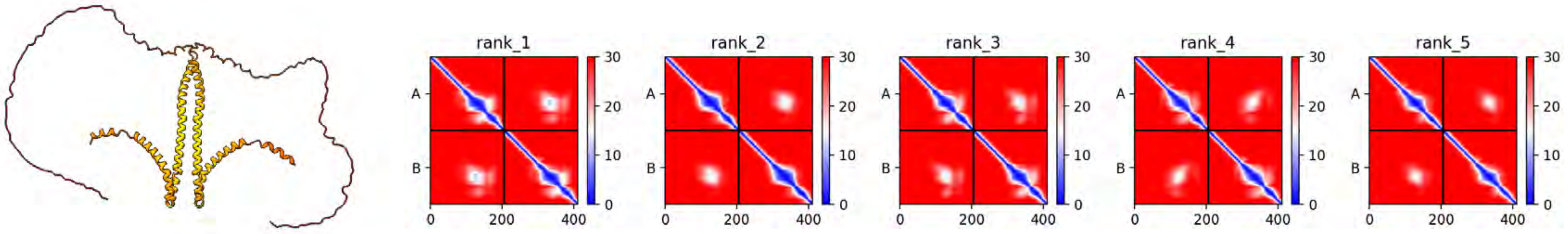

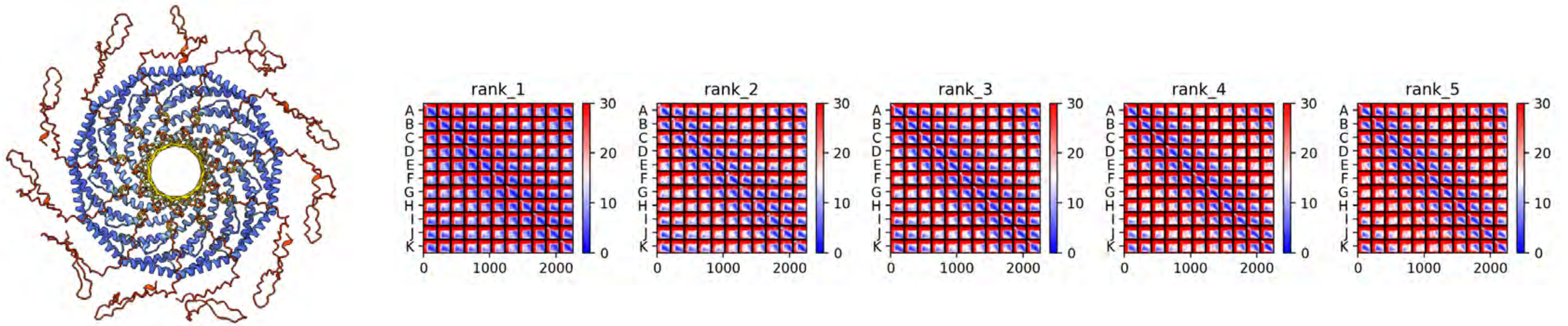

A0A7M7HH57 *S. purpuratus*

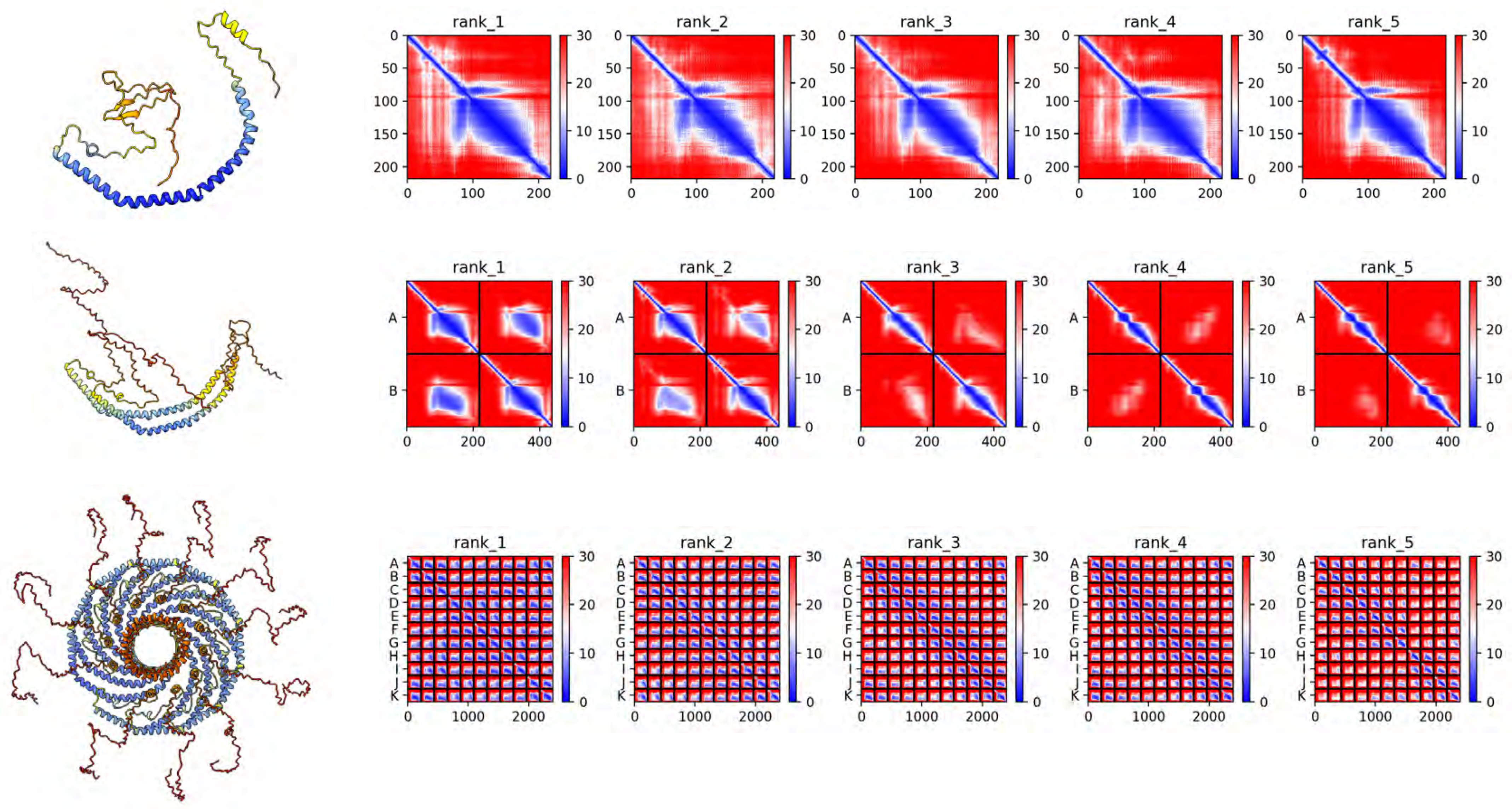

R7U639 ***C. teleta***

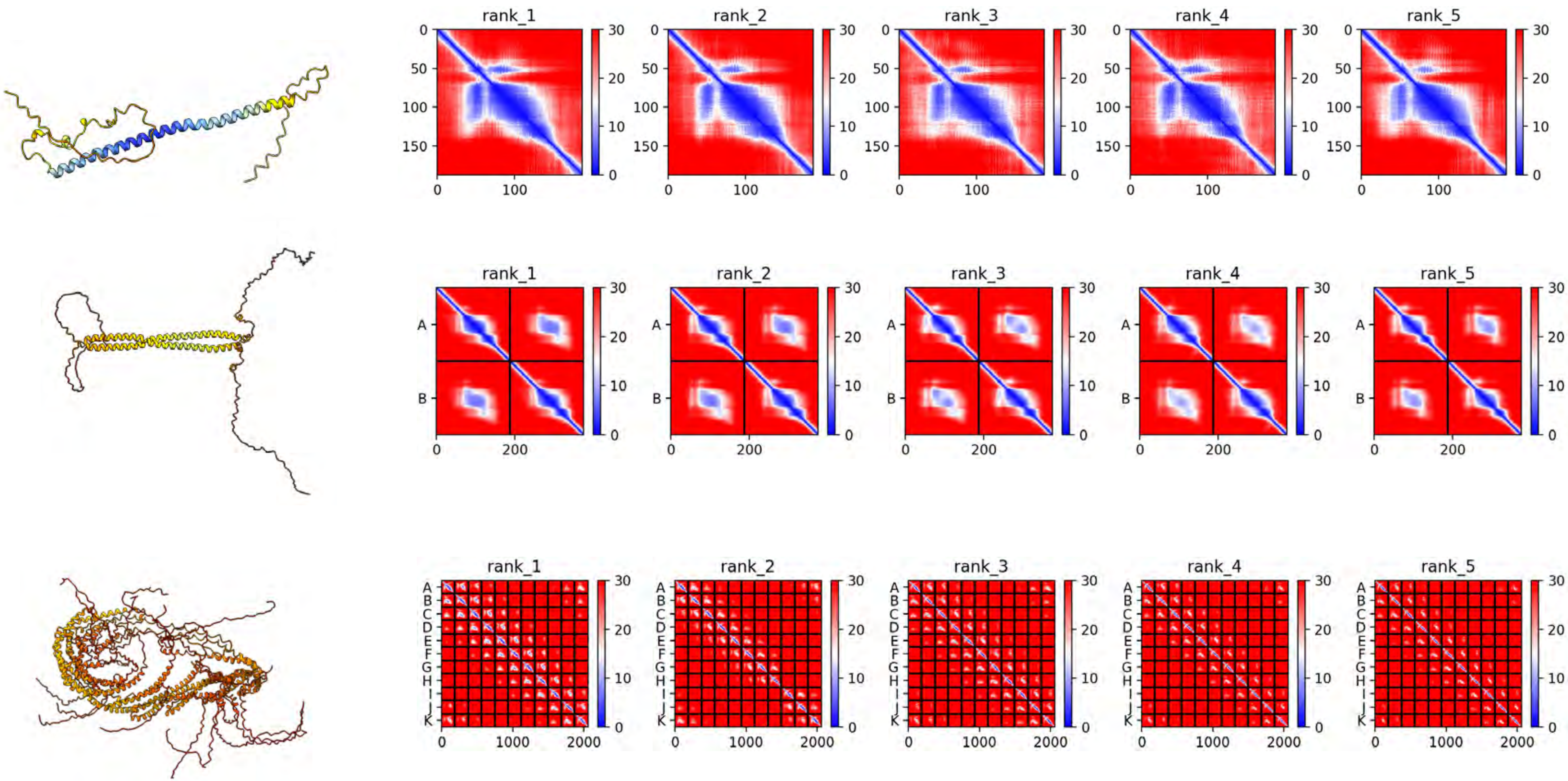

A0A7M7HEM1 *S. purpuratus*

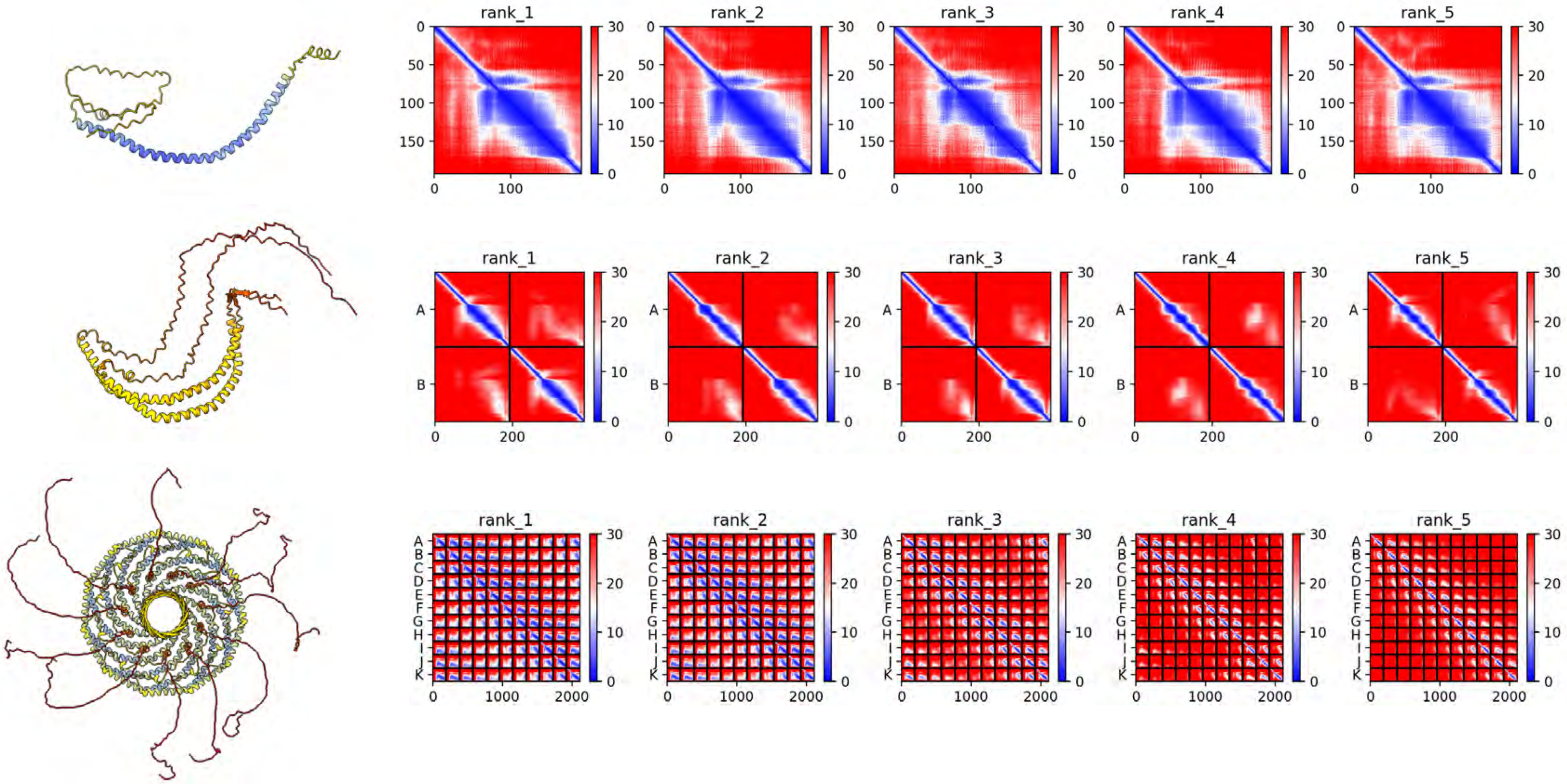

A0A7M7T4C2 *S. purpuratus*

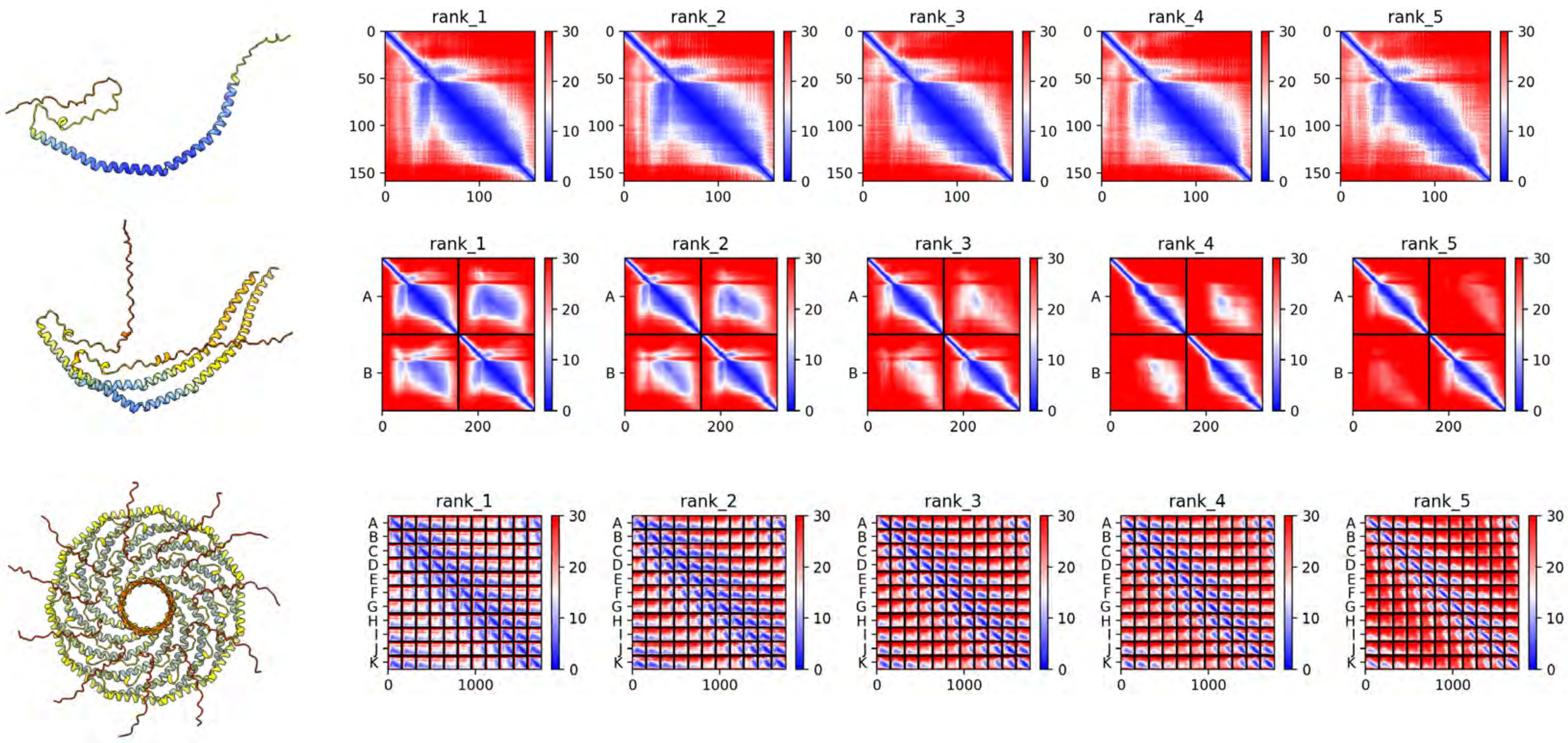

A0A7M7G9F7 *S. purpuratus*

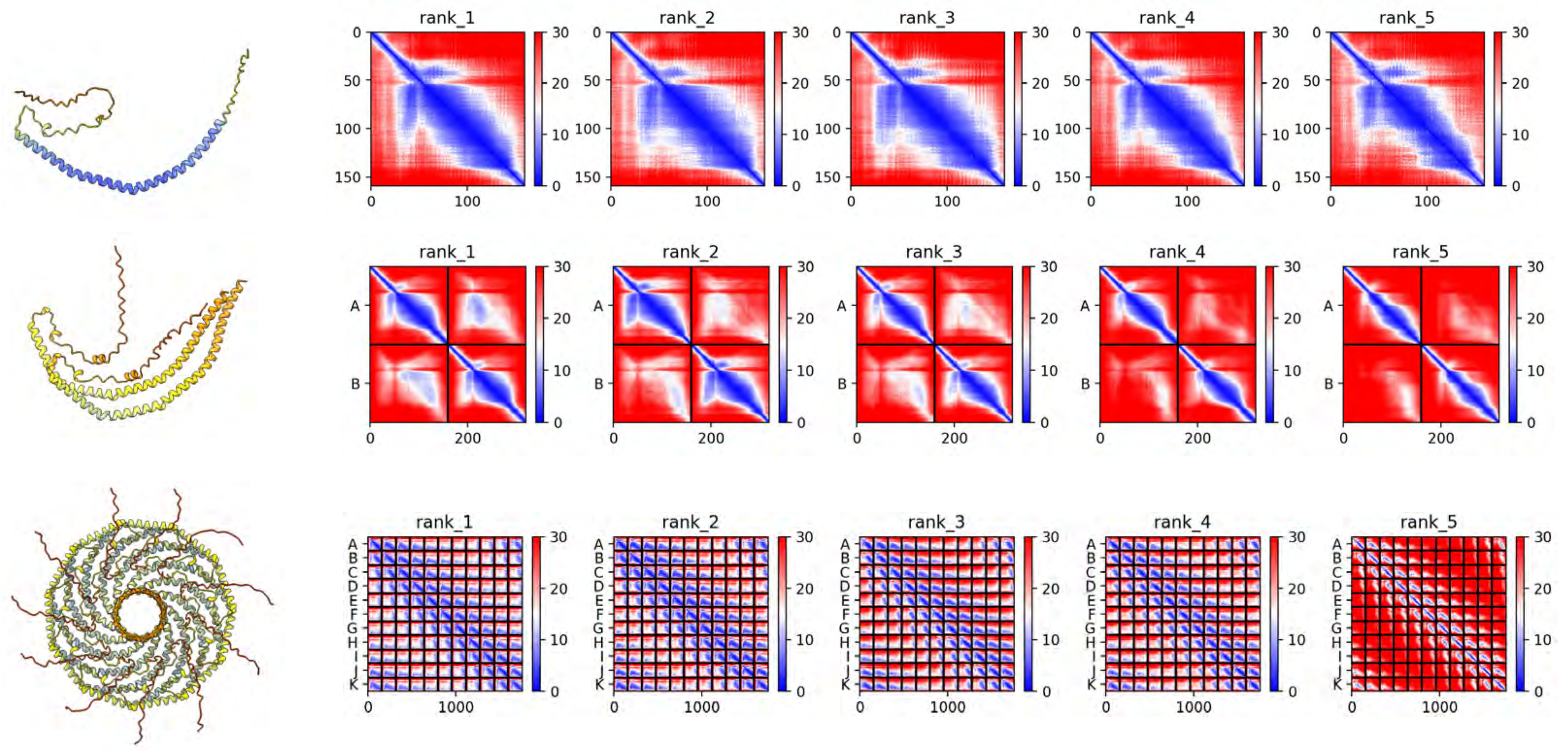

A0A7M7PLL3 *S. purpuratus*

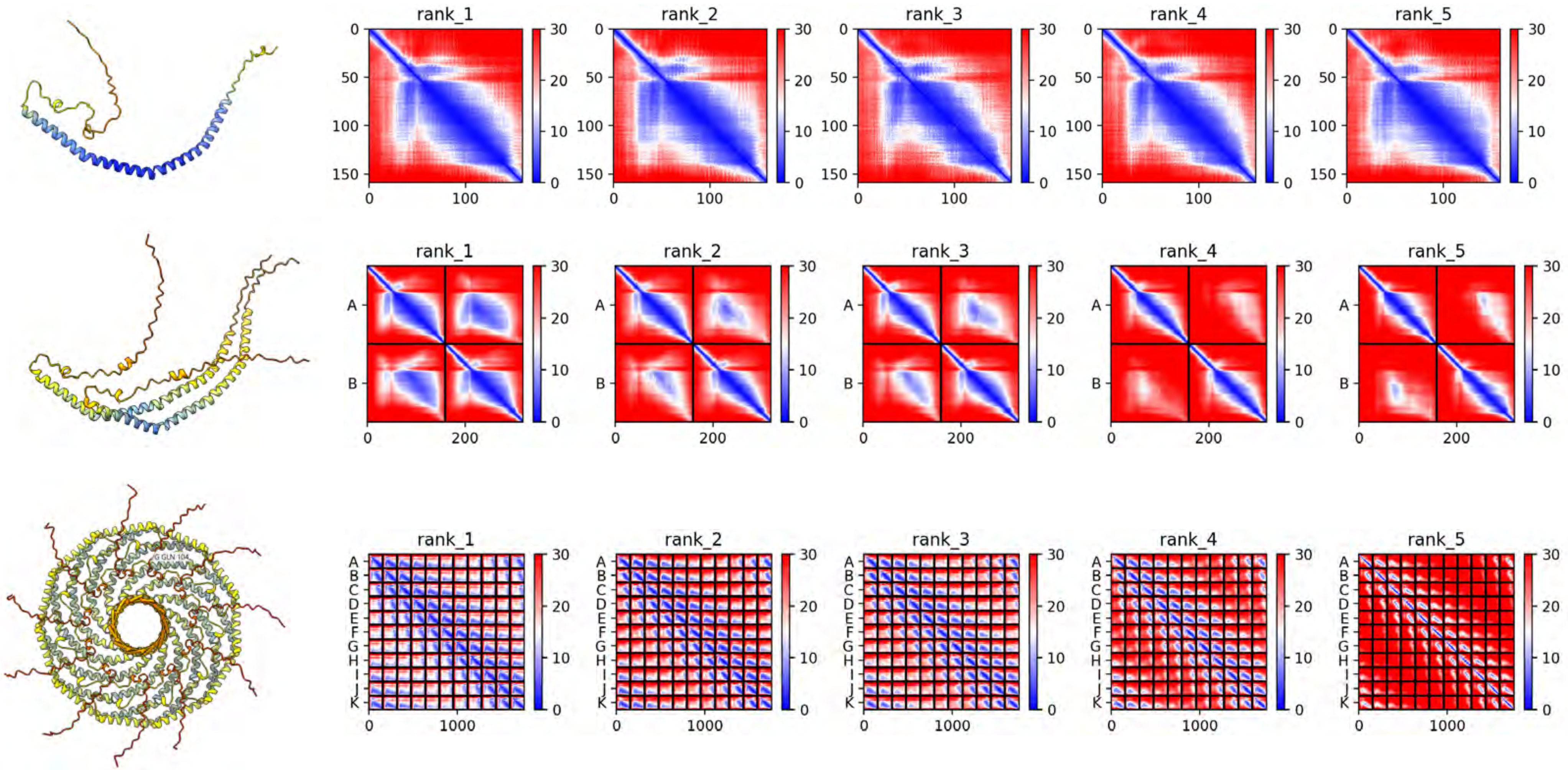

Q94051 *C. elegans*

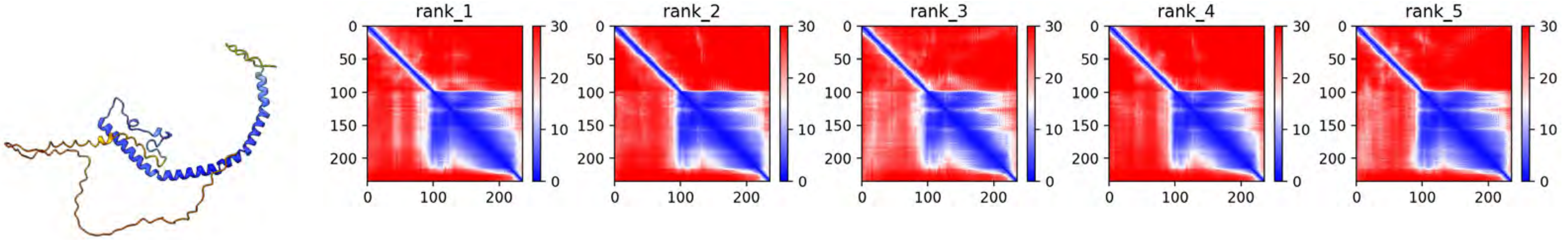

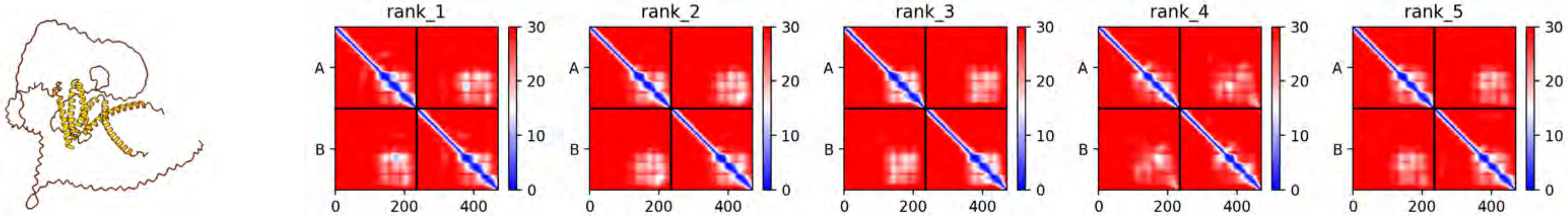

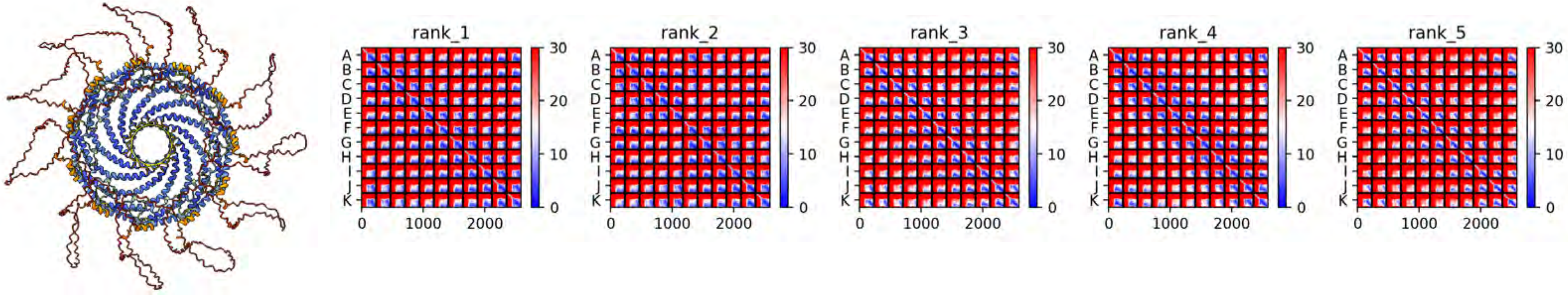

A0A2T7NUA4 *P. canaliculata*

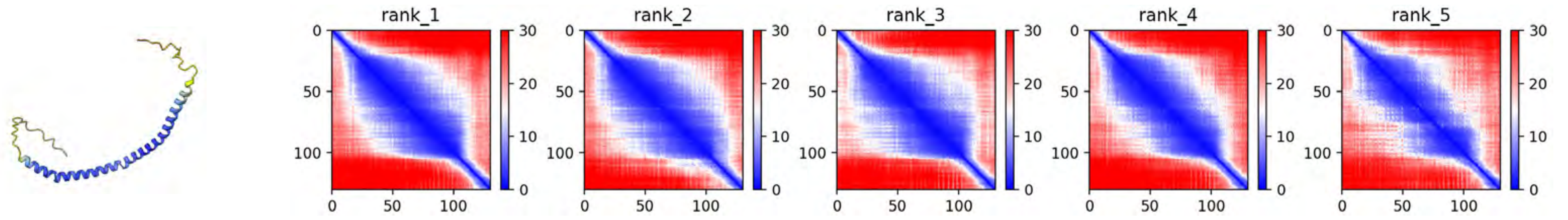

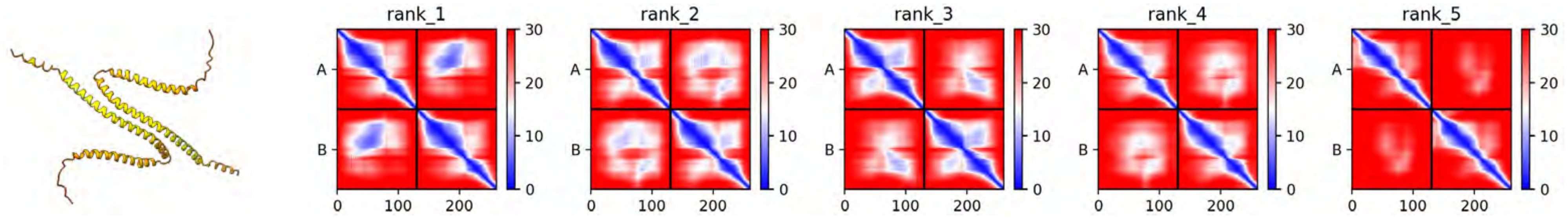

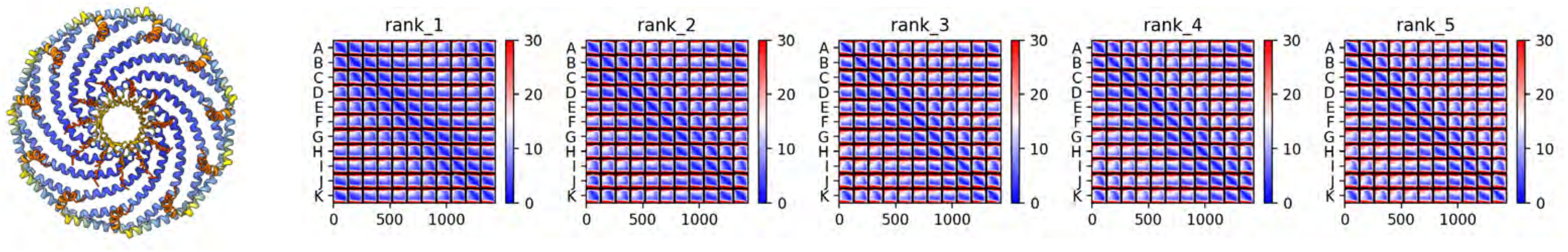

Q18879 *C. elegans*

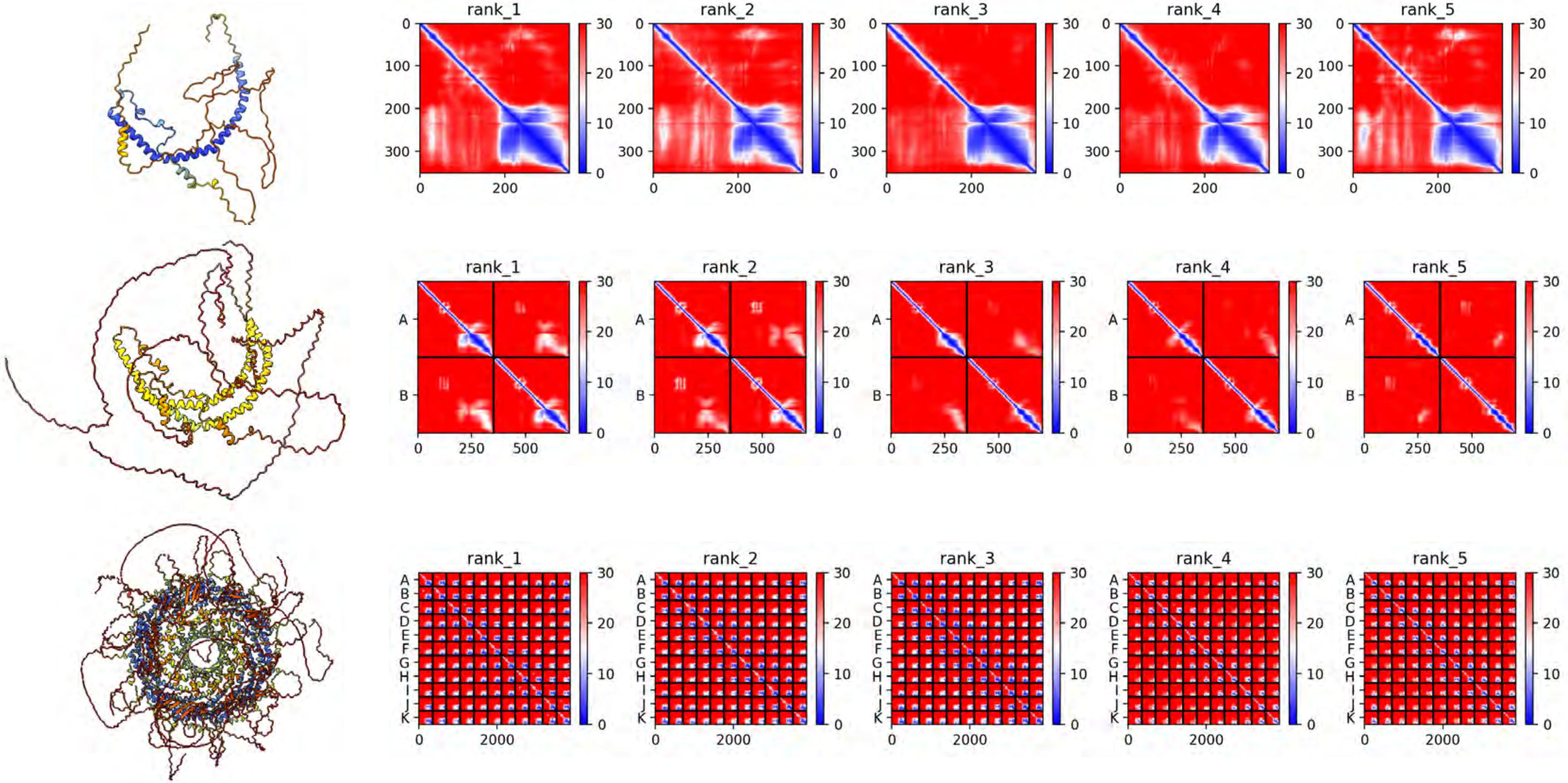

A0A7M7GJ11 *S. purpuratus*

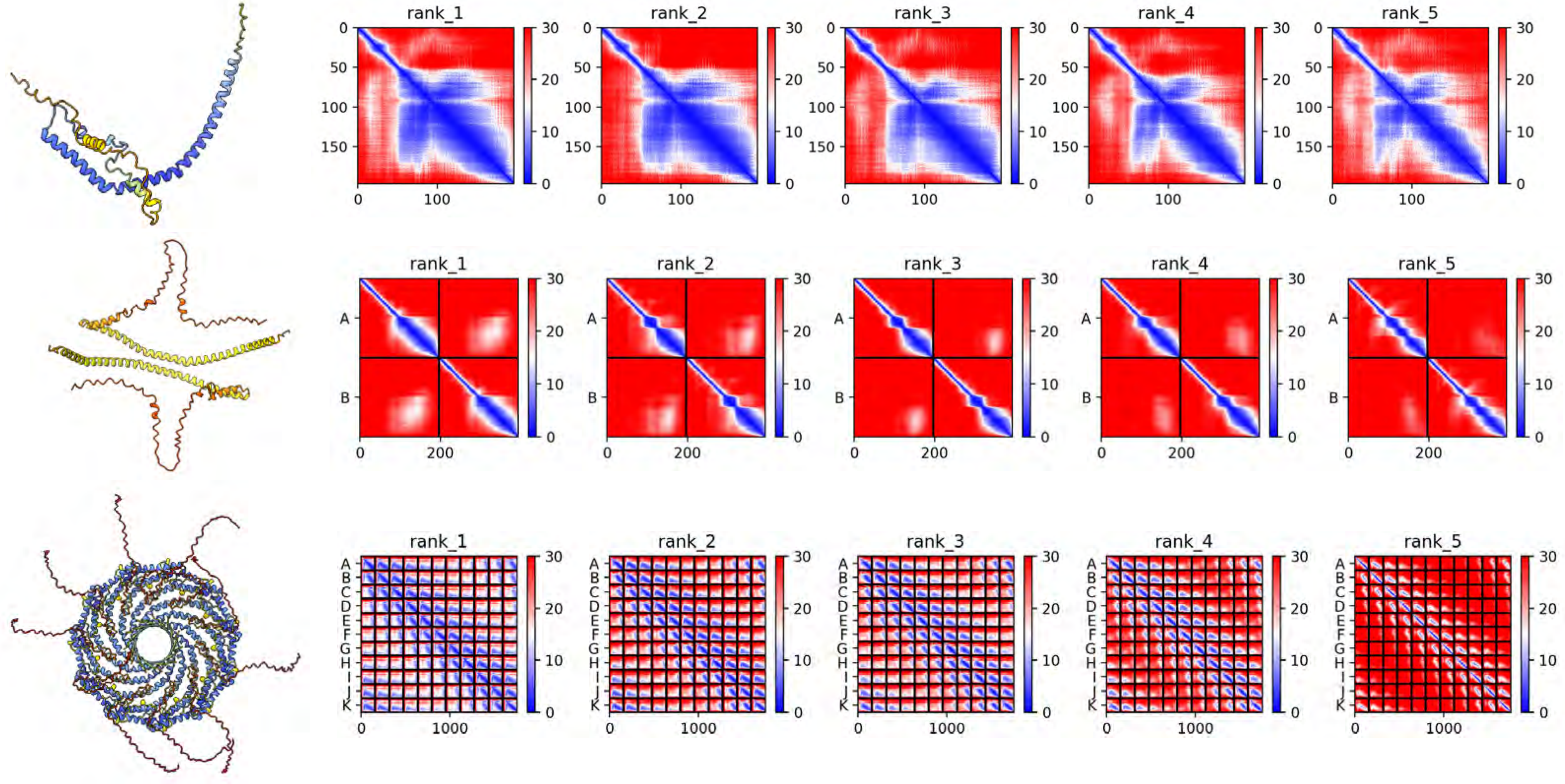

A0A7M7P0X0 *S. purpuratus*

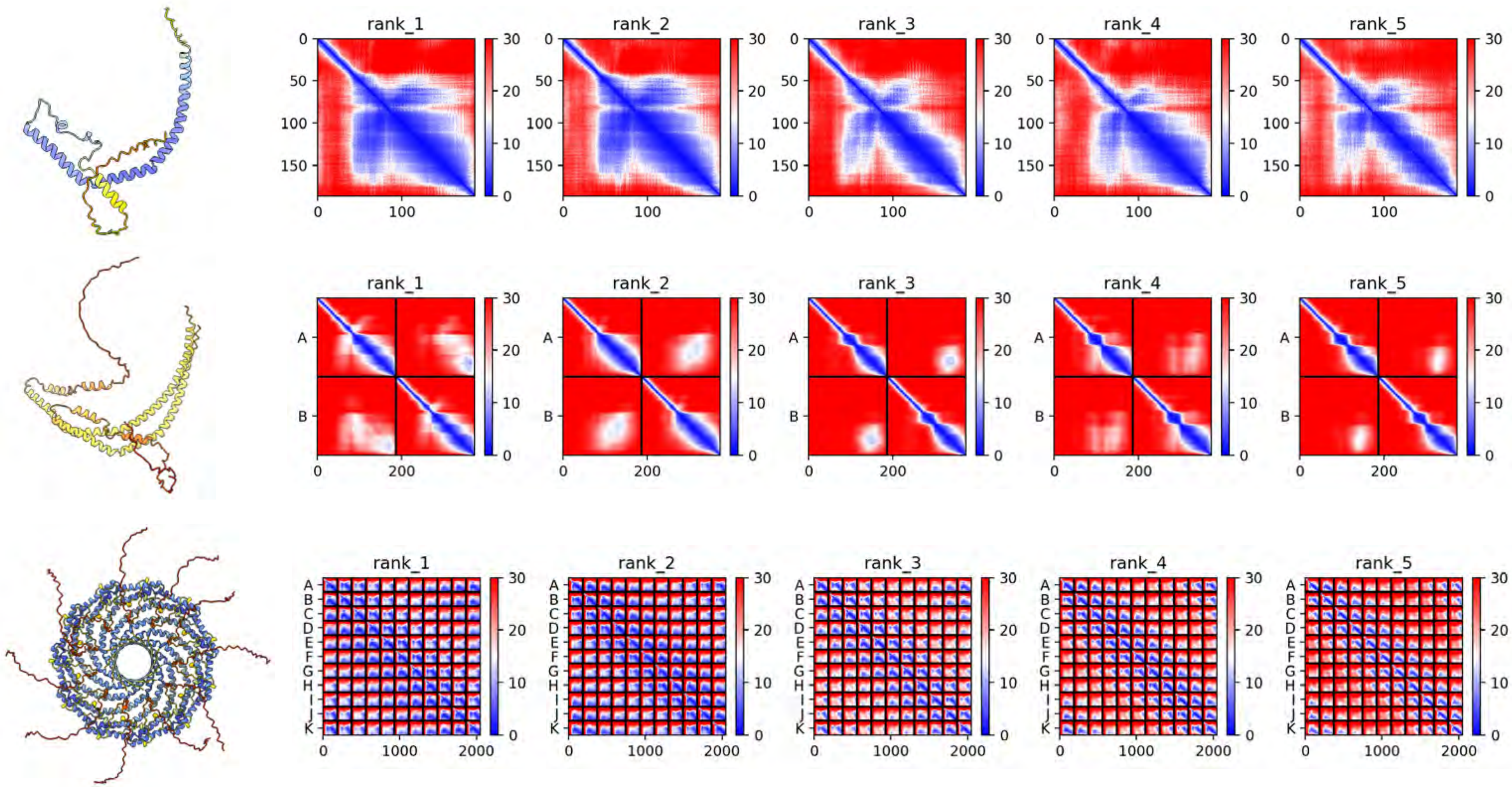

B3RWV2 *T. adhaerens*

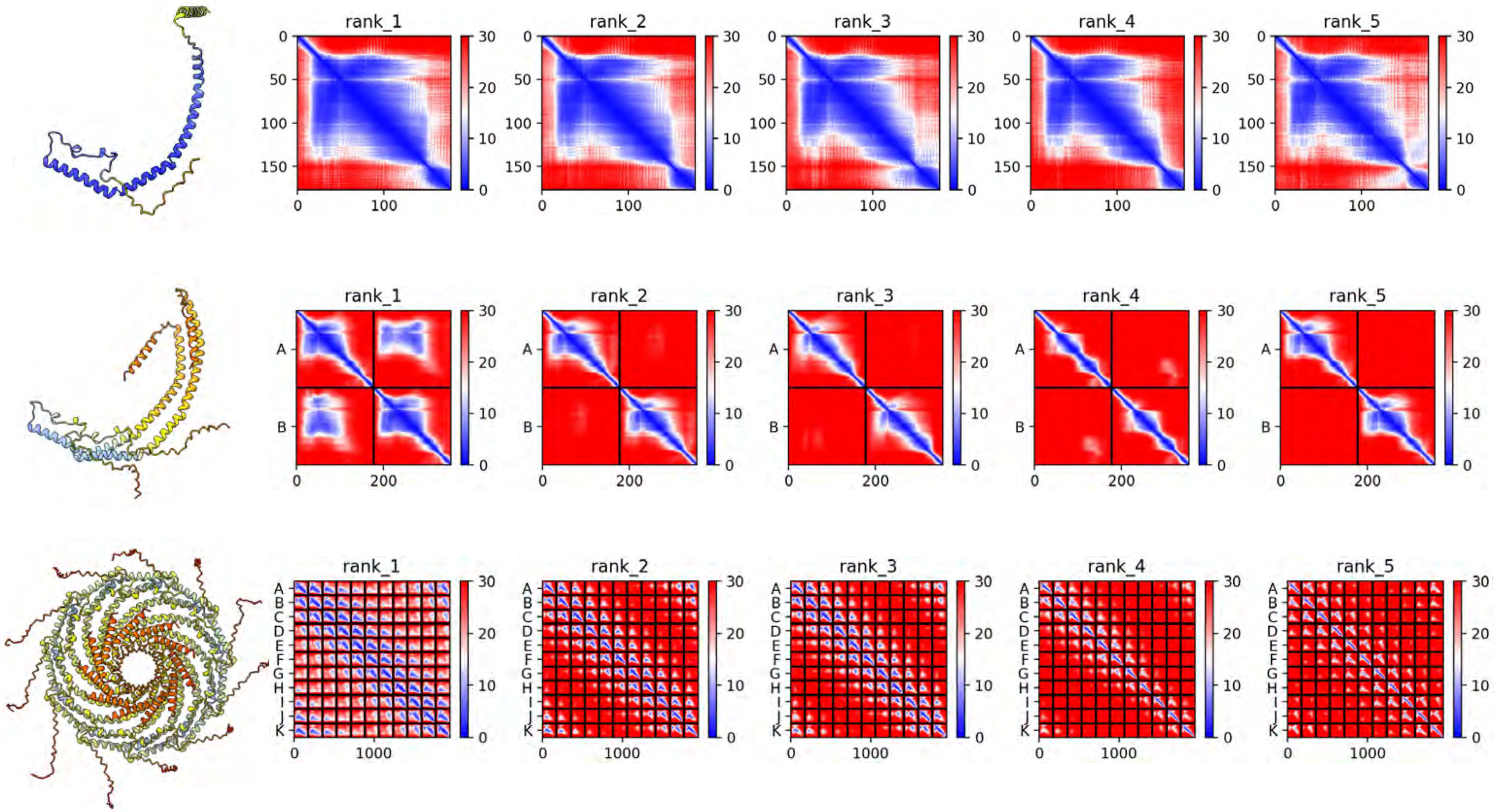

R7TPM2 *C. teleta*

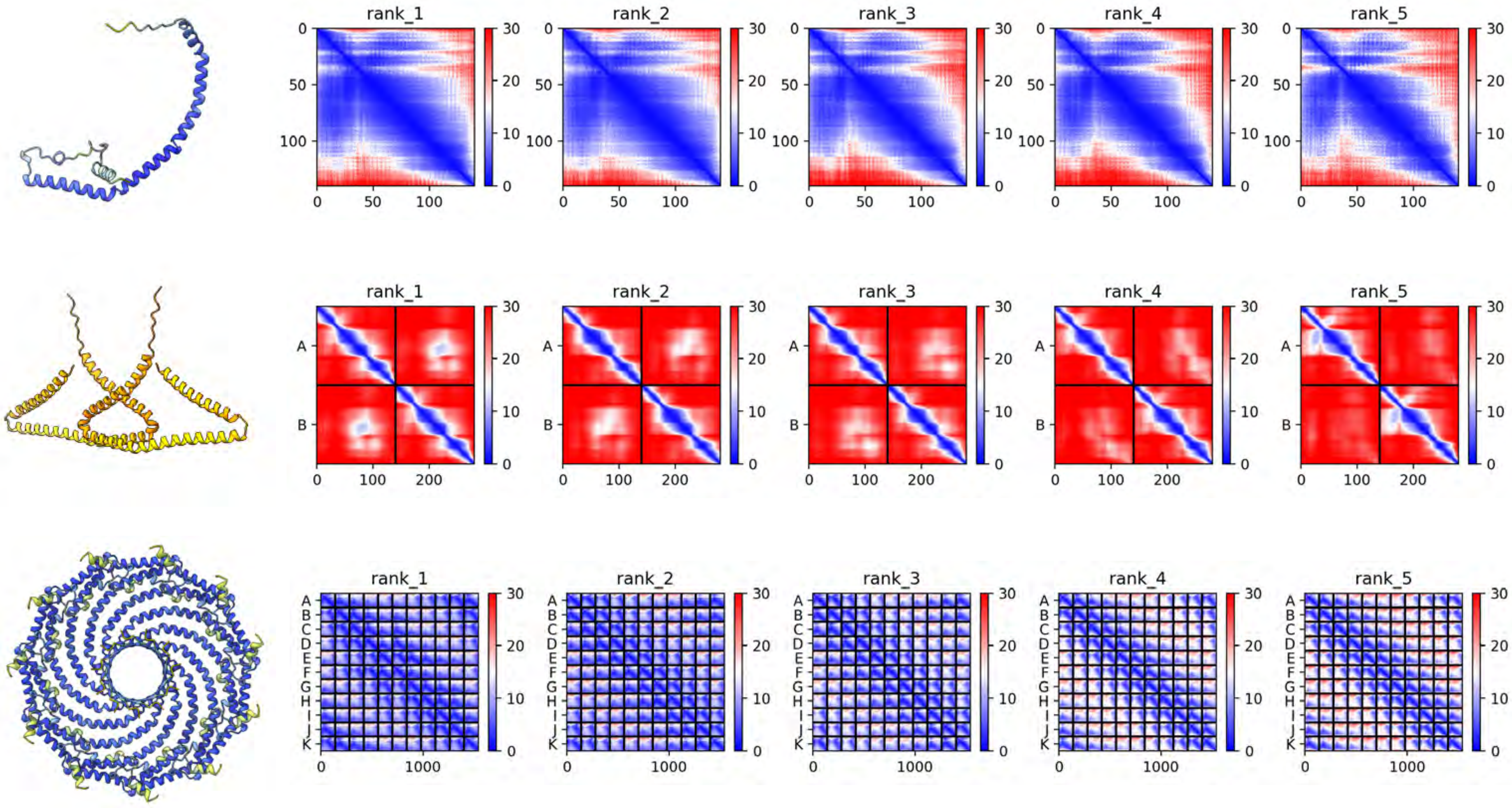

A0A1I8GQY0 *M. lignano*

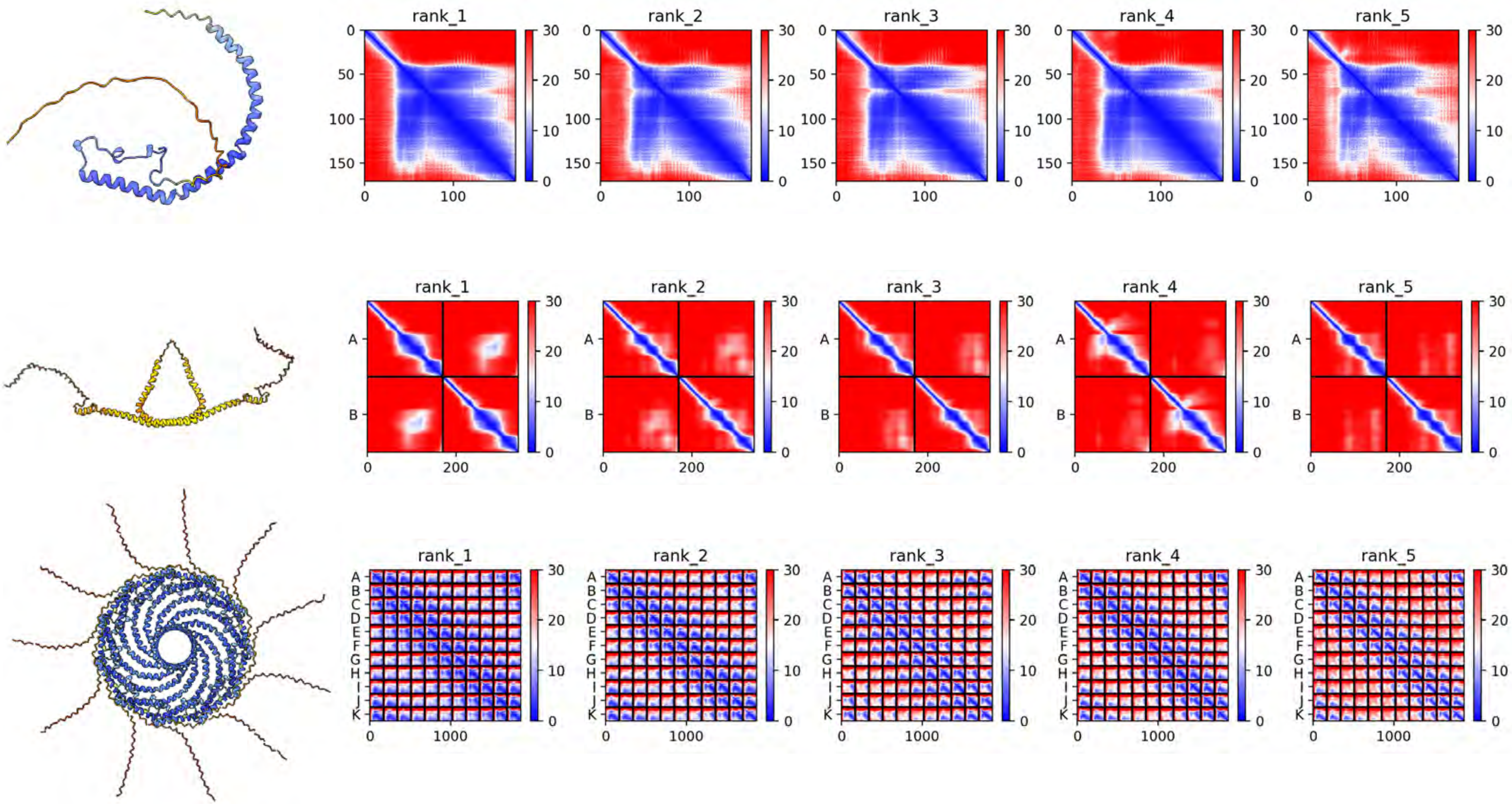

A0A267DFI9 *M. lignano*

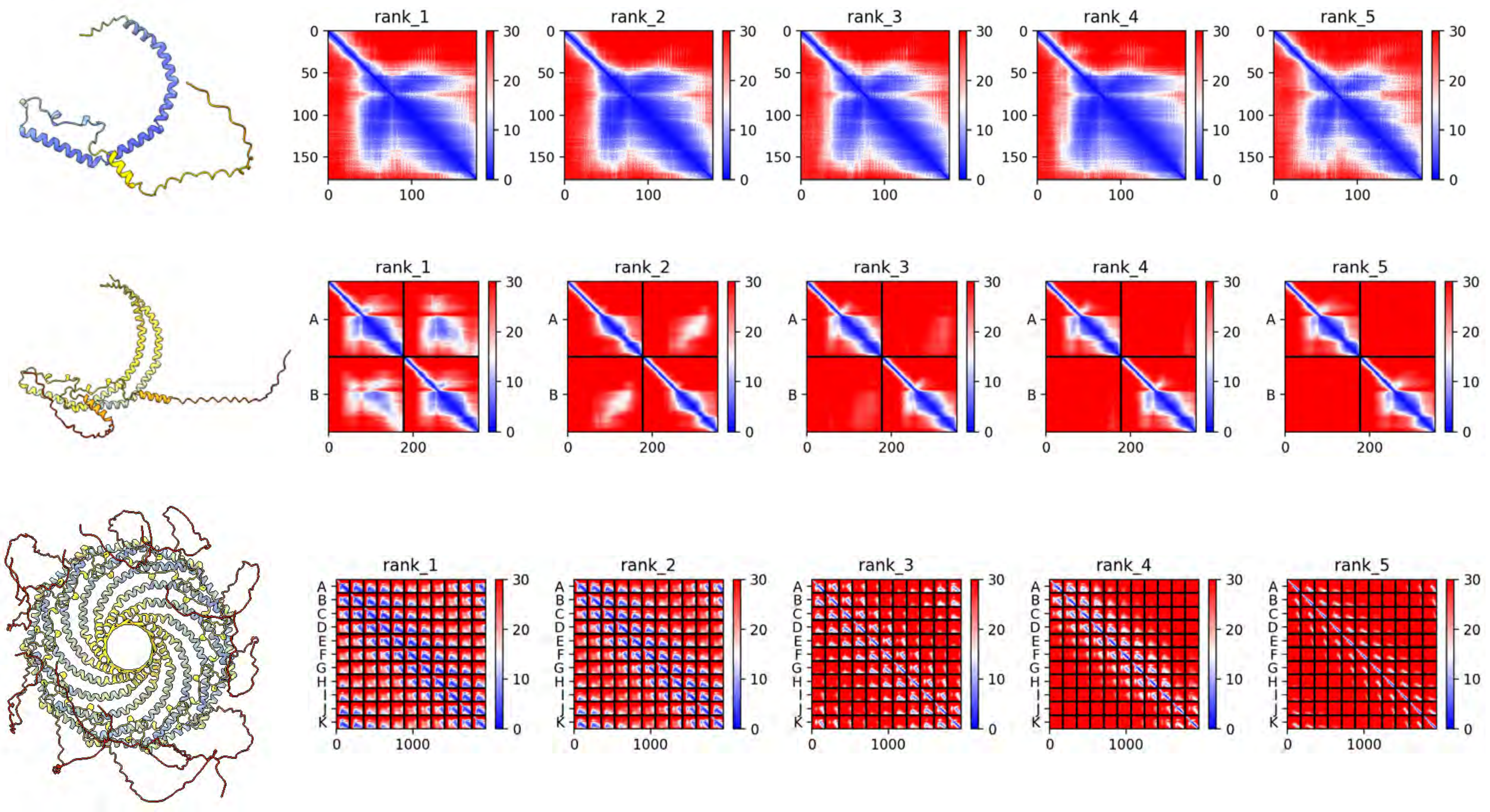

A0A267G3E9 *M. lignano*

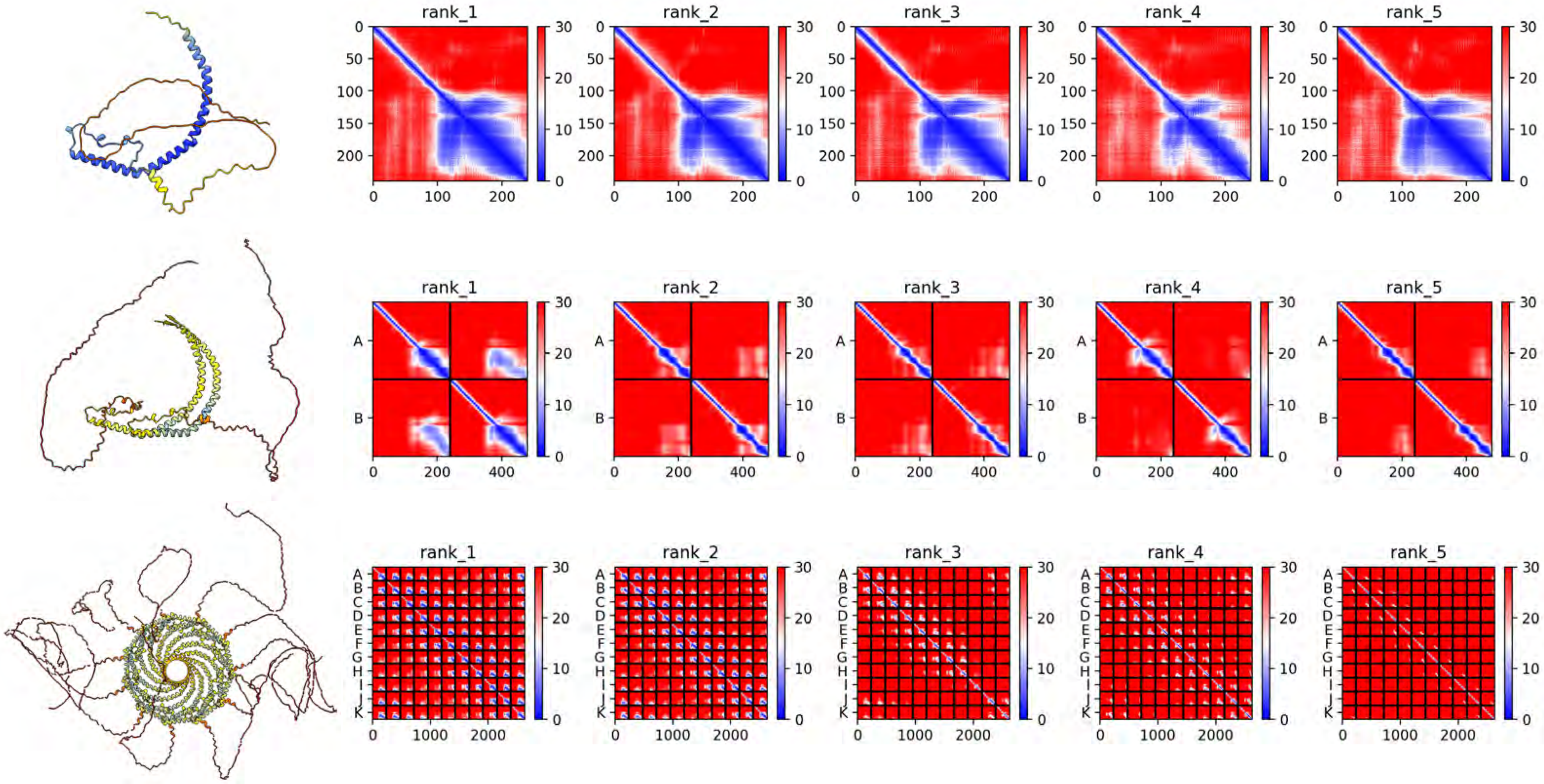

A0A267GQL3 *M. lignano*

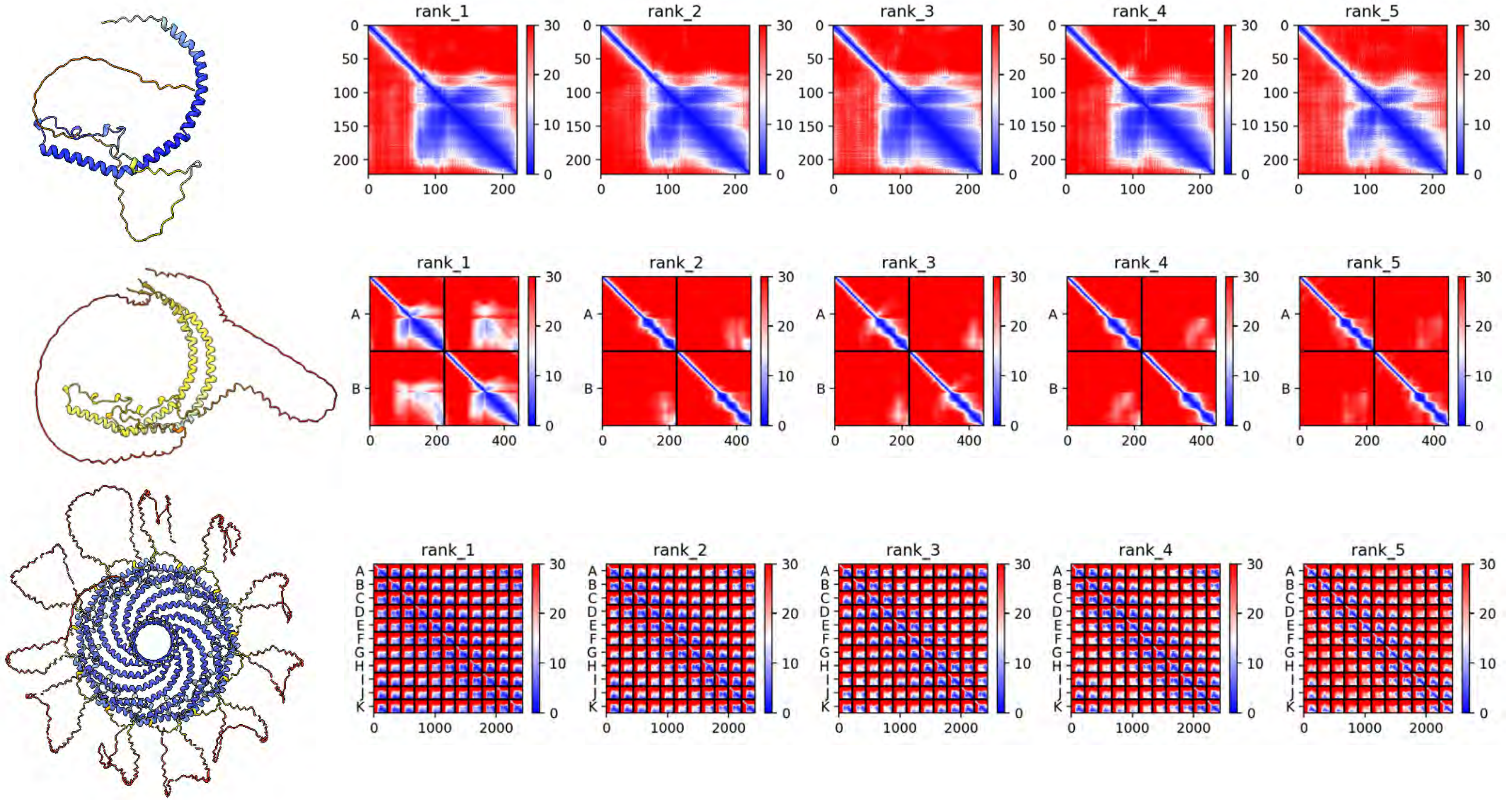

A0A267G3D8 *M. lignano*

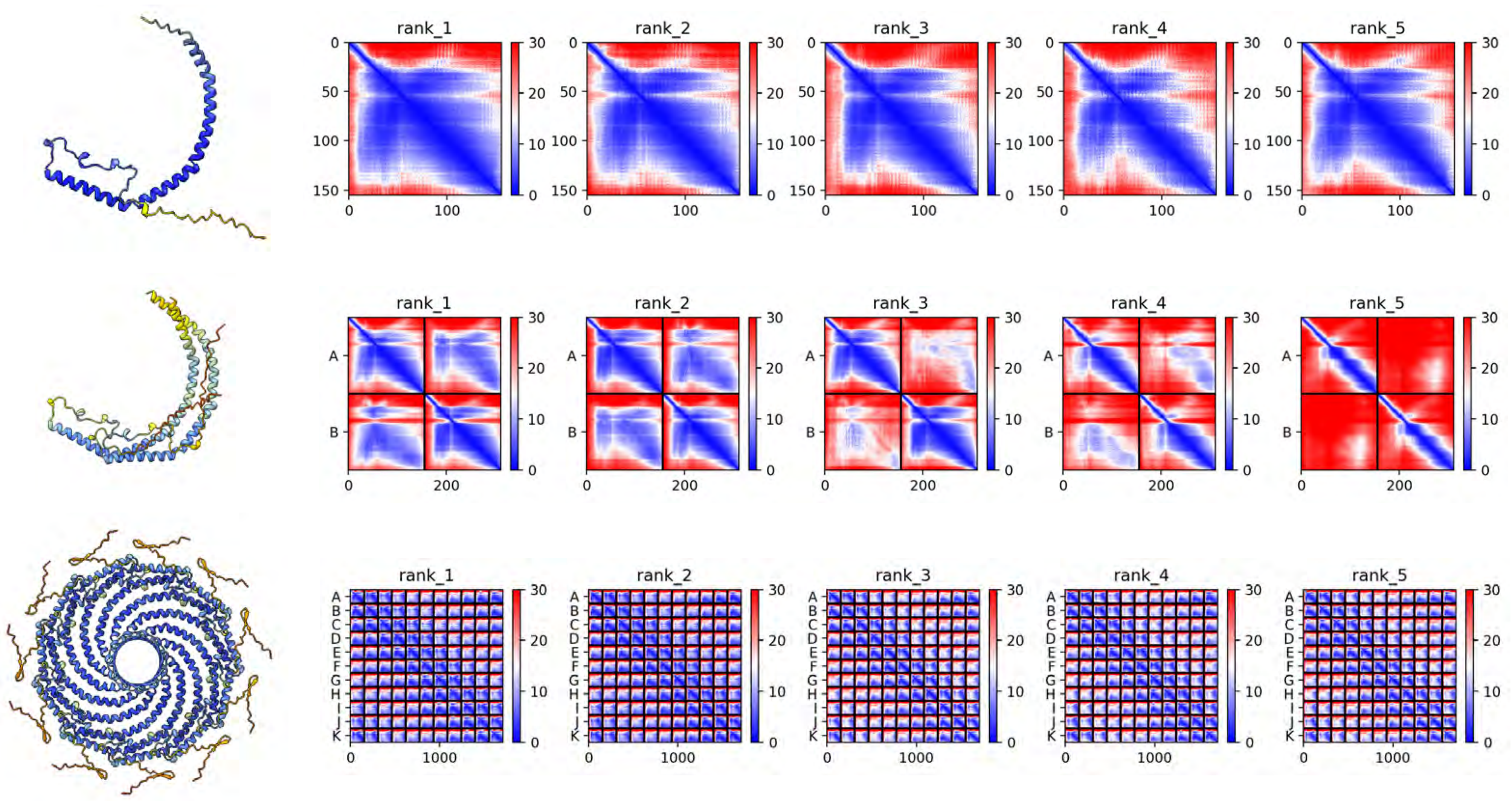

A0A267G387 *M. lignano*

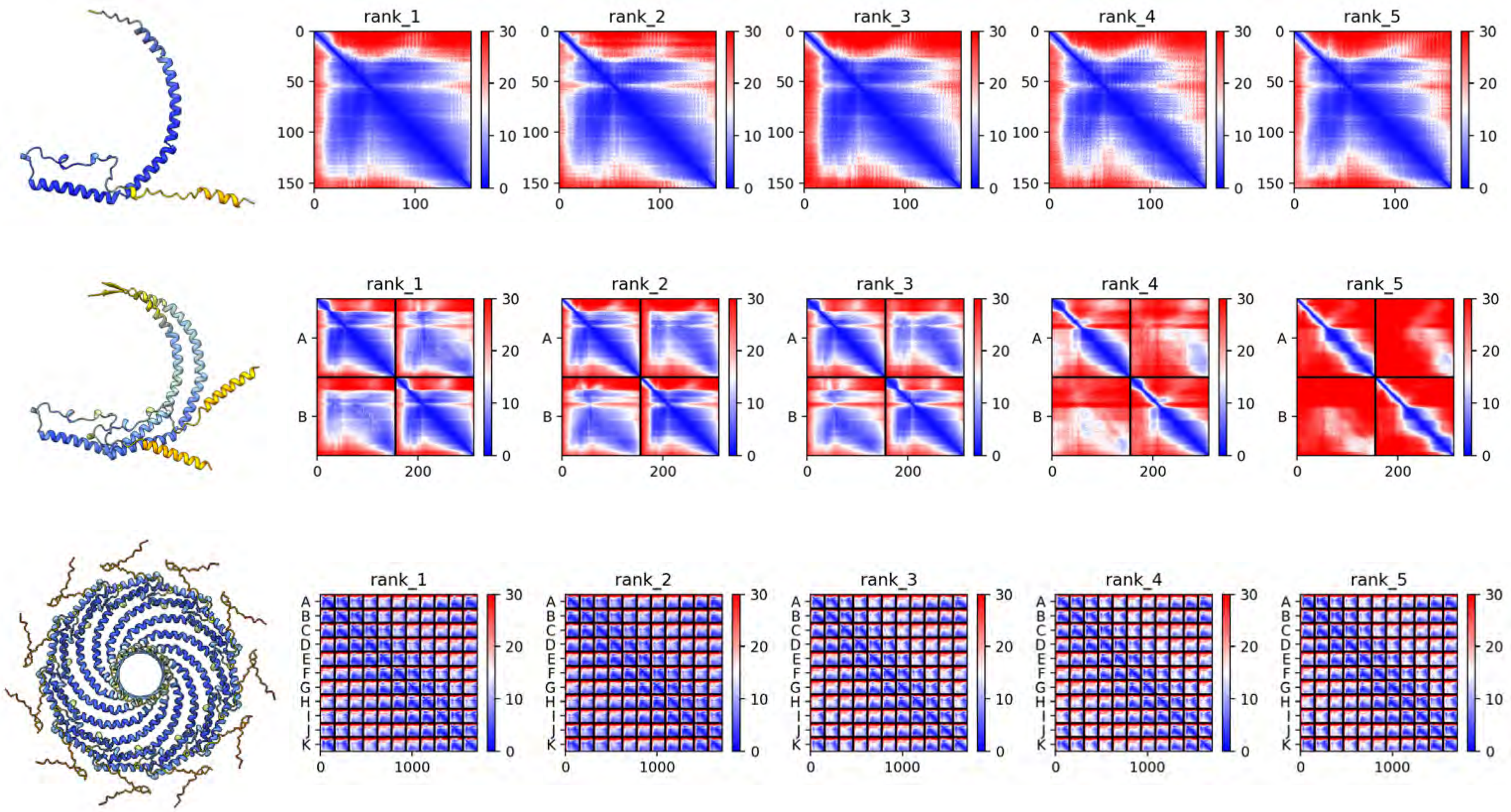

A0A267G1N3 *M. lignano*

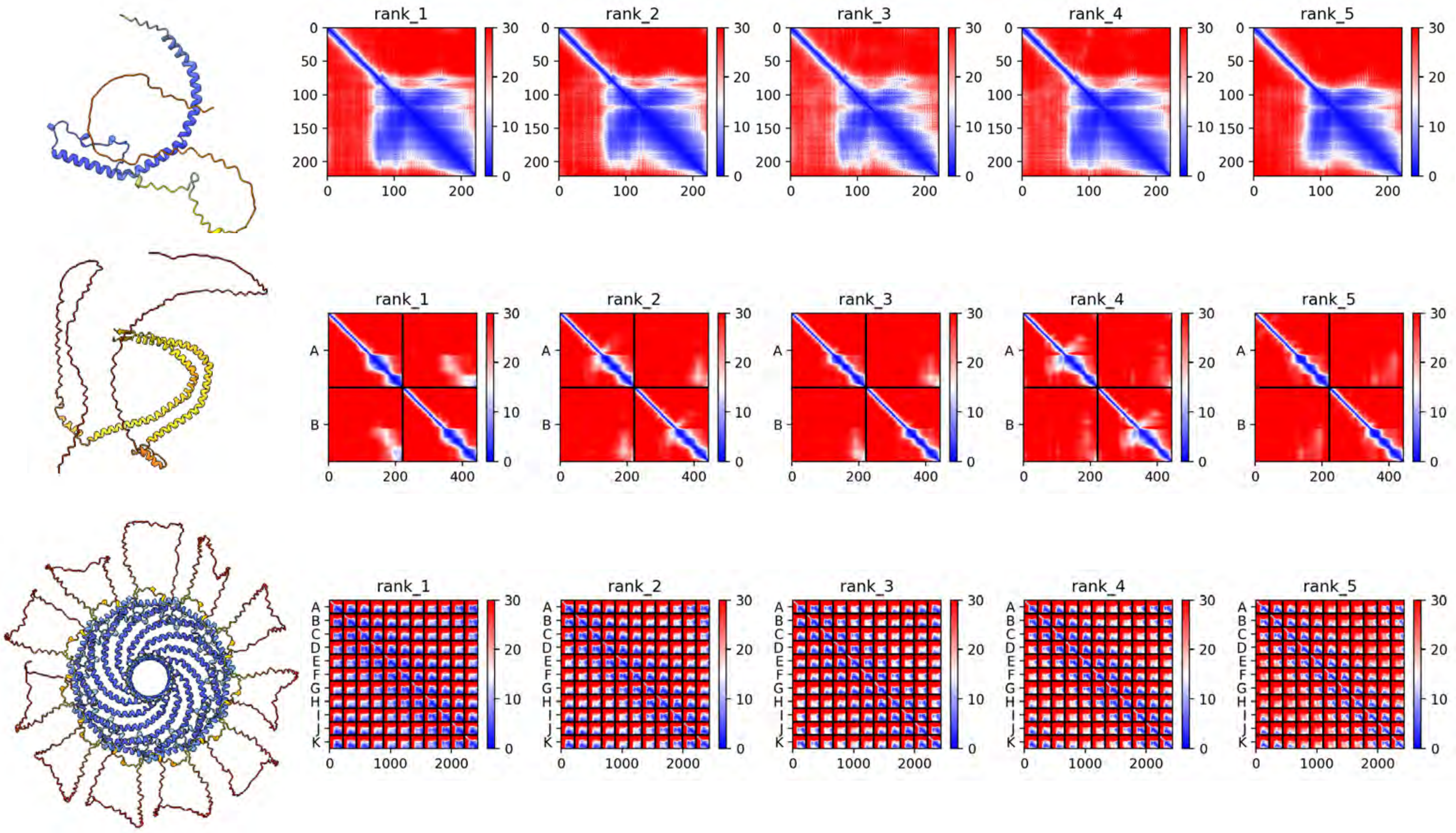

A0A267DNA3 *M. lignano*

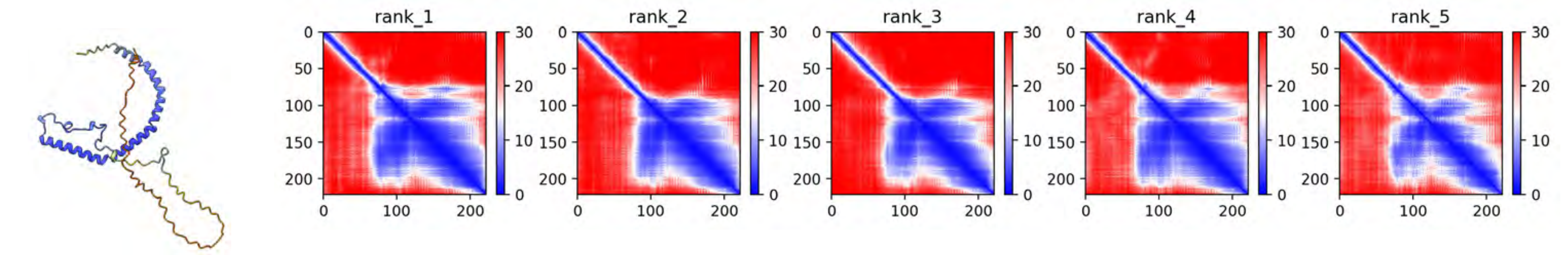

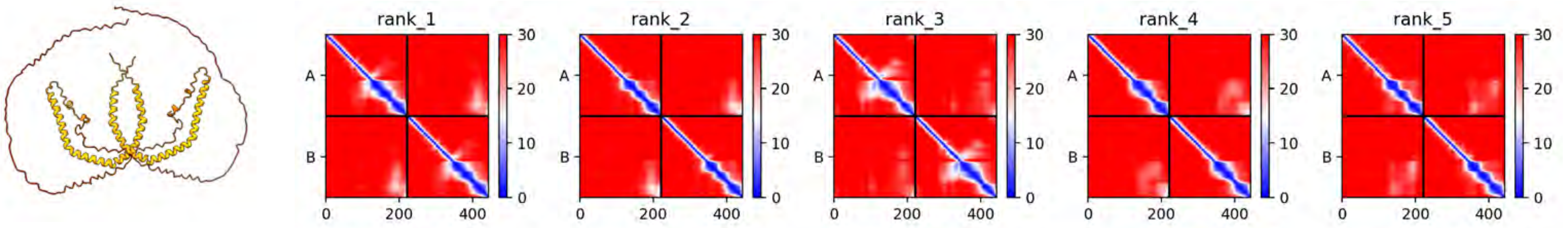

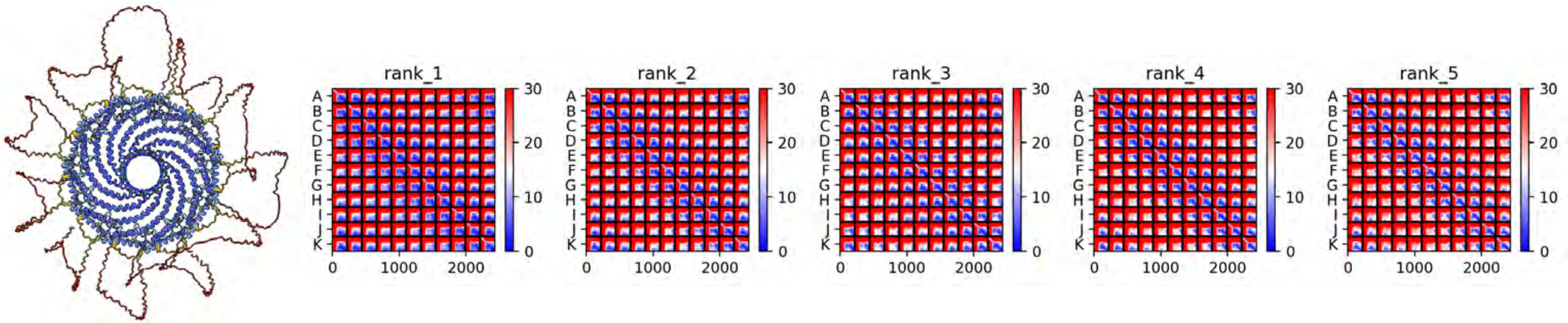

A0A267FMS2 *M. lignano*

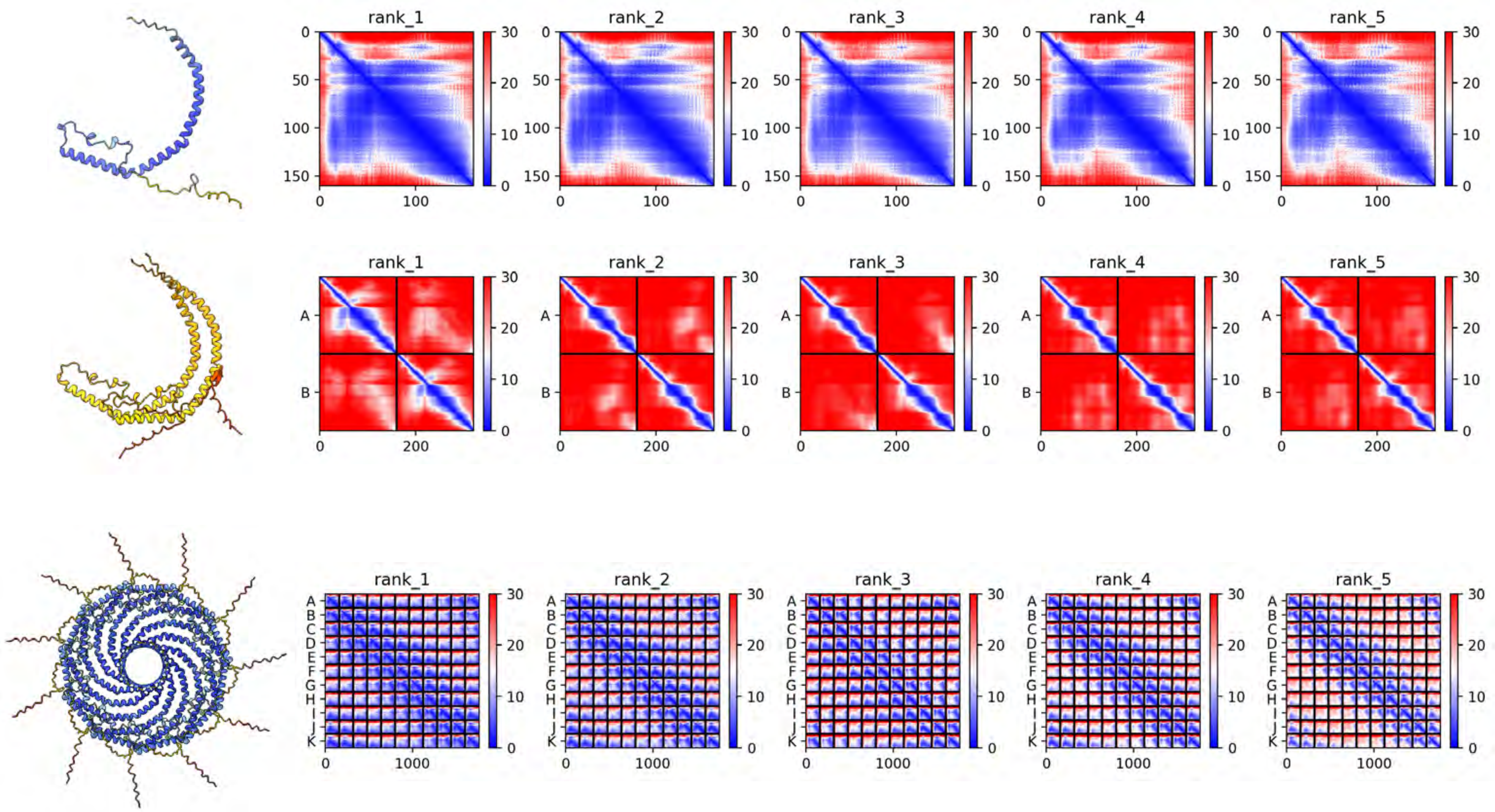

XP_014664738.1 *P. caudatus*

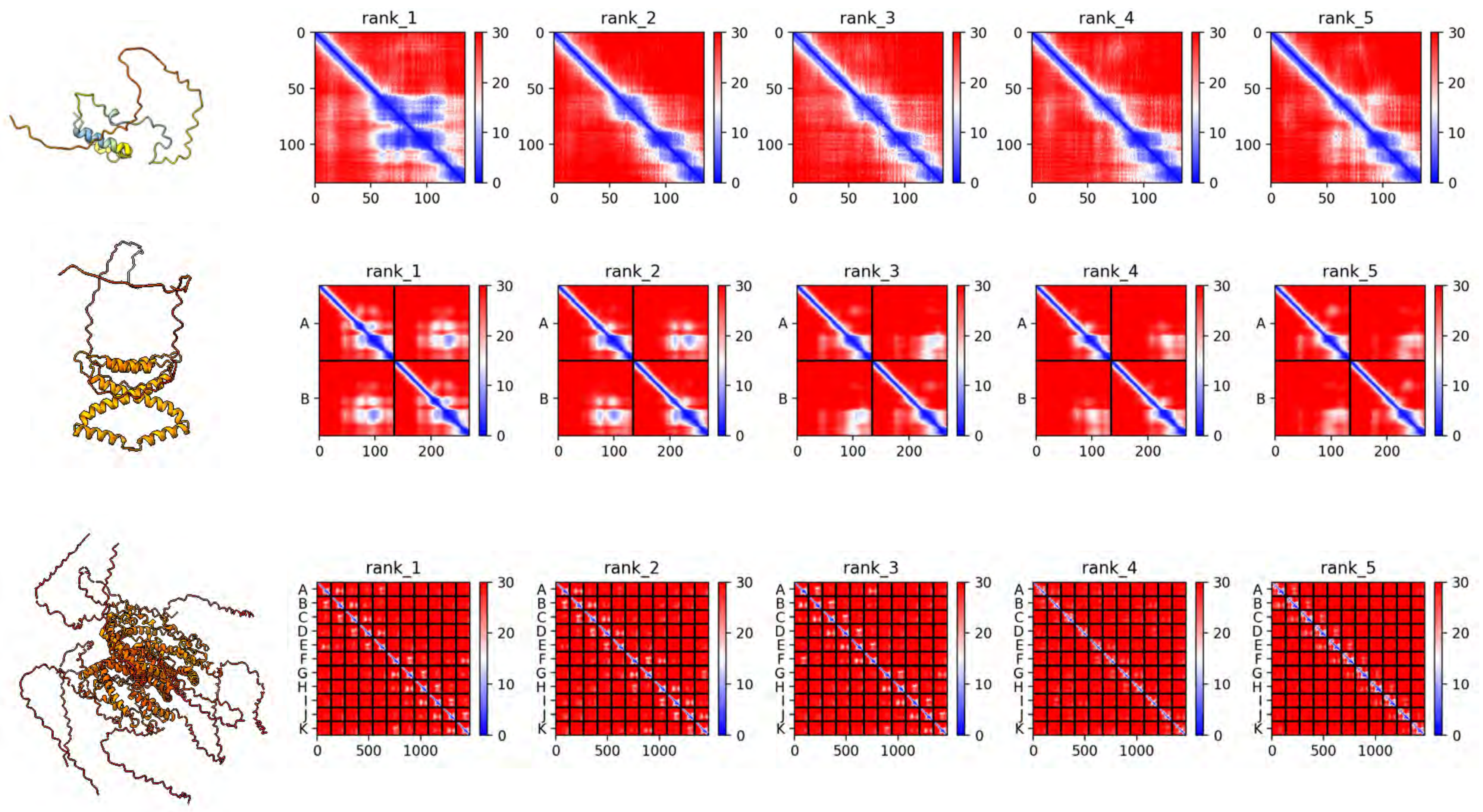

A0A1S3IXS7 *L. unguis*

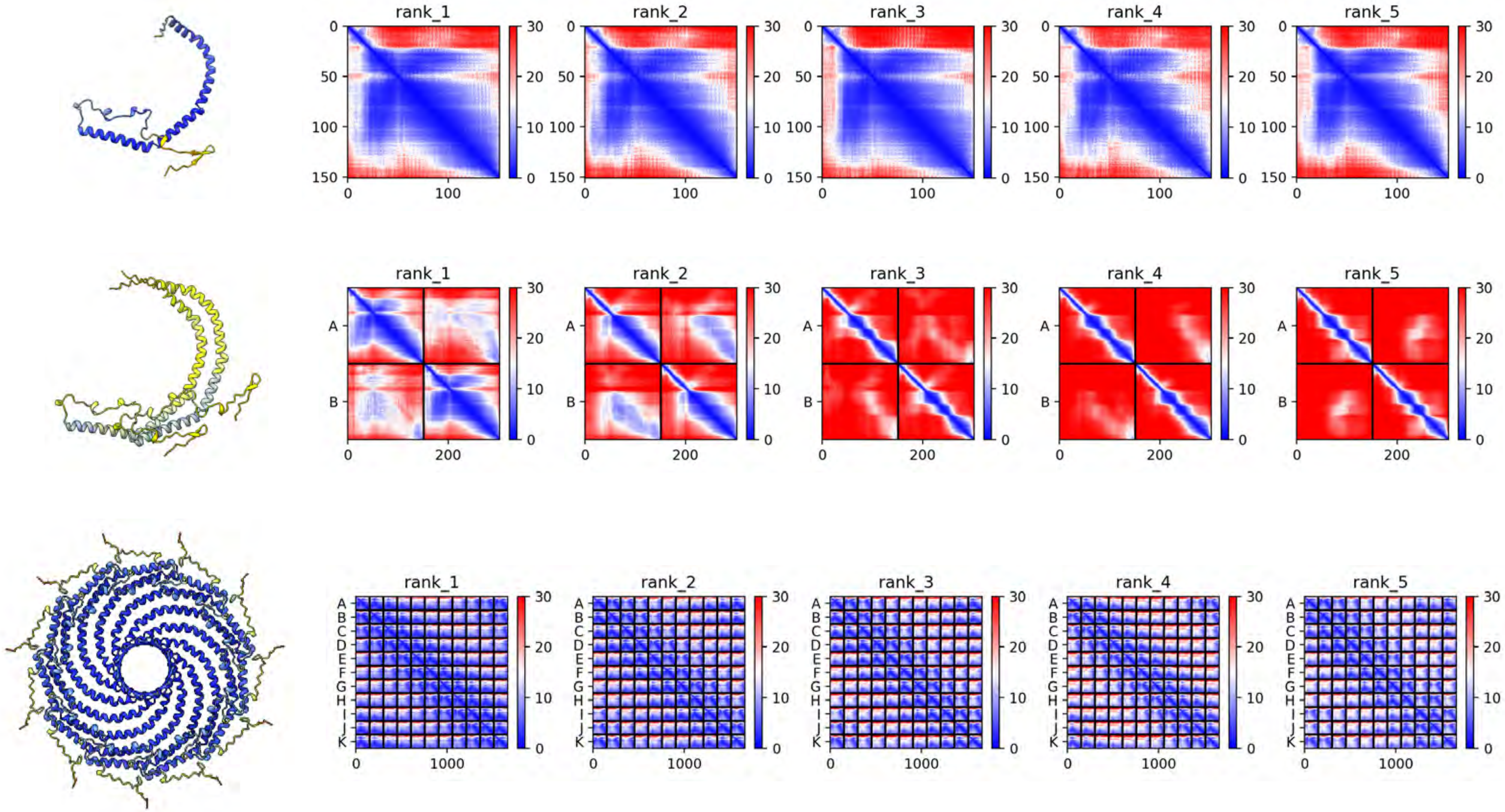

A0A1S3I8M7 *L. unguis*

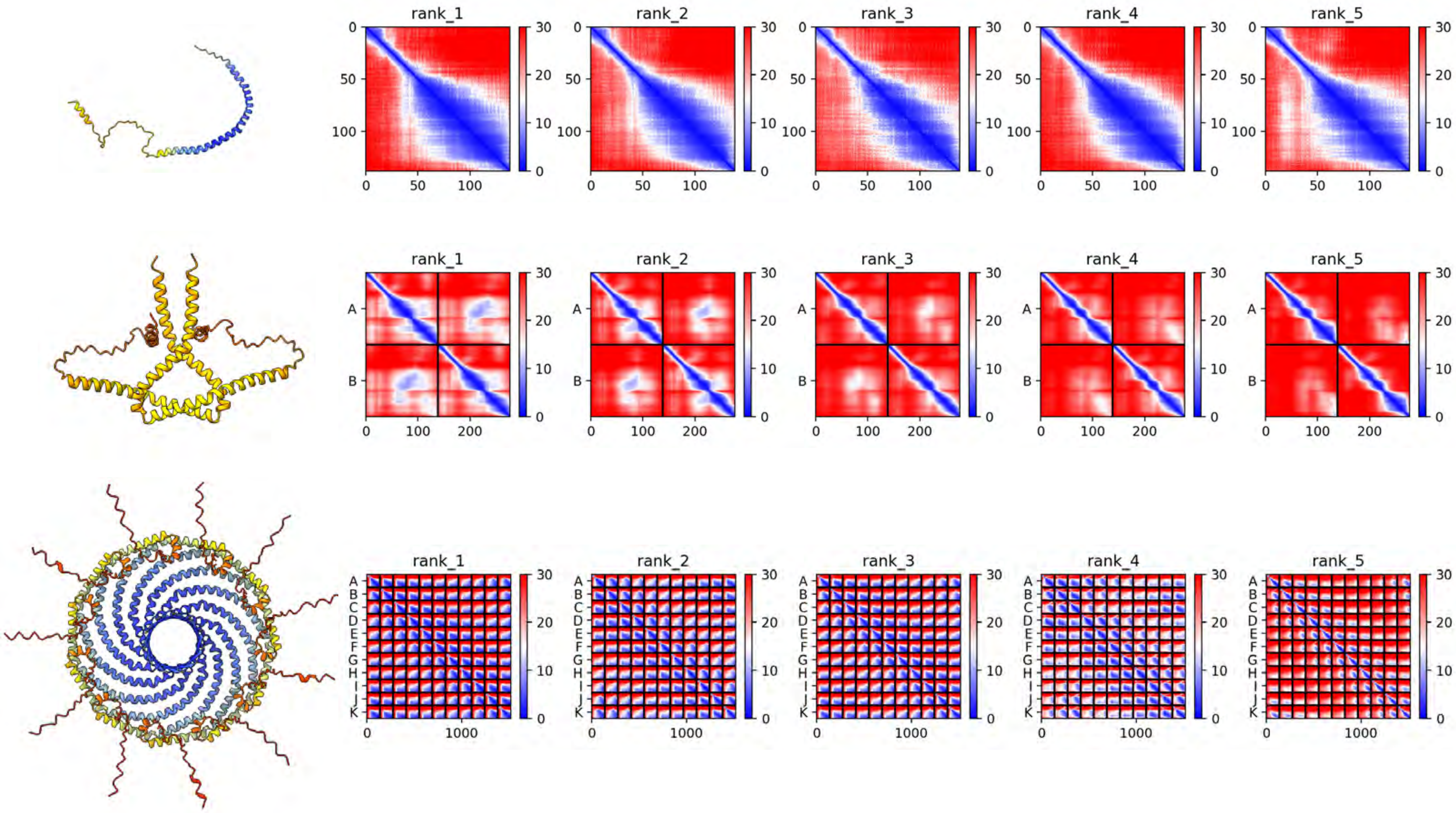

A0A1S3I469 *L. unguis*

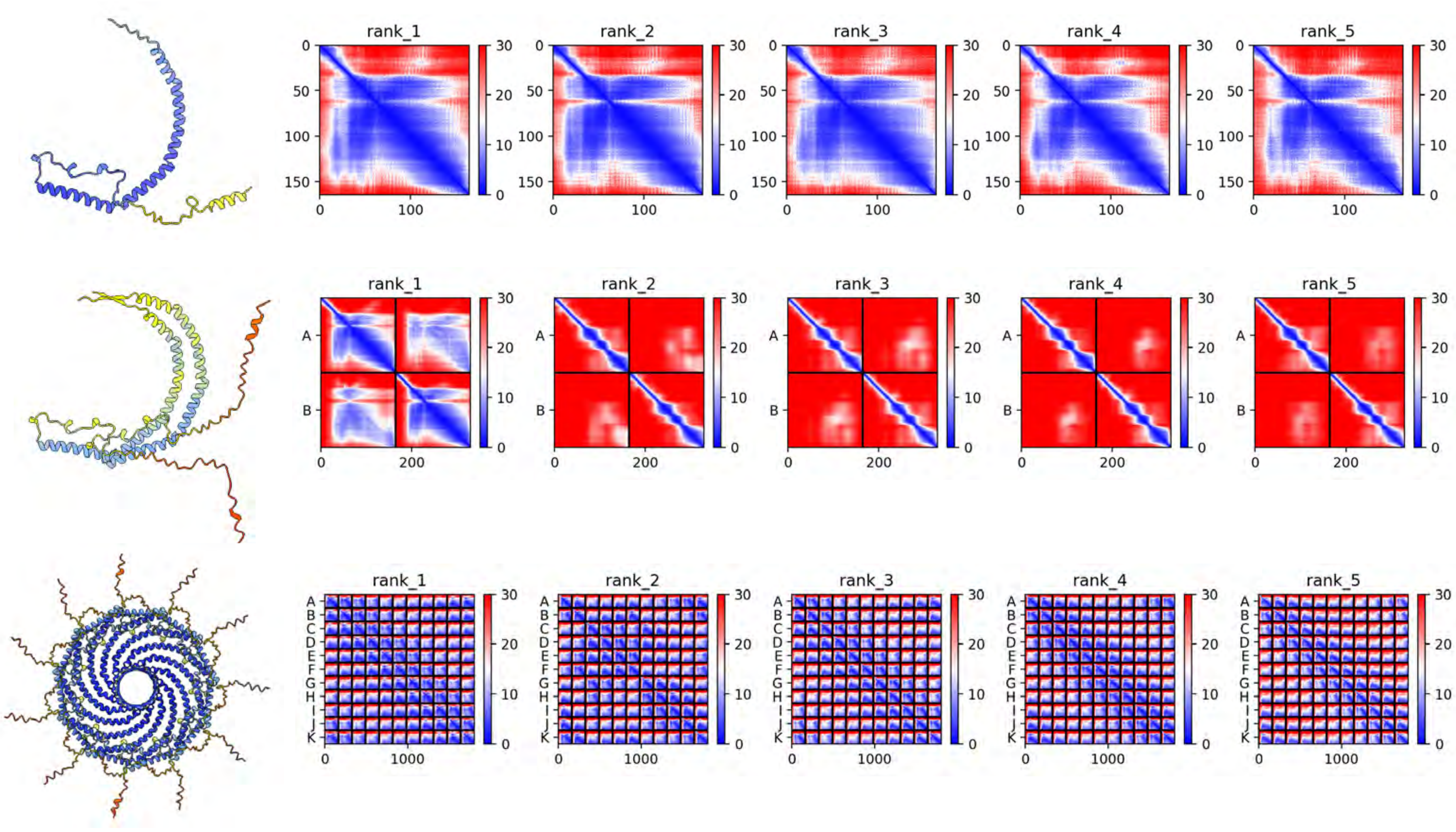

P51636 (CAV2) *H. sapiens*

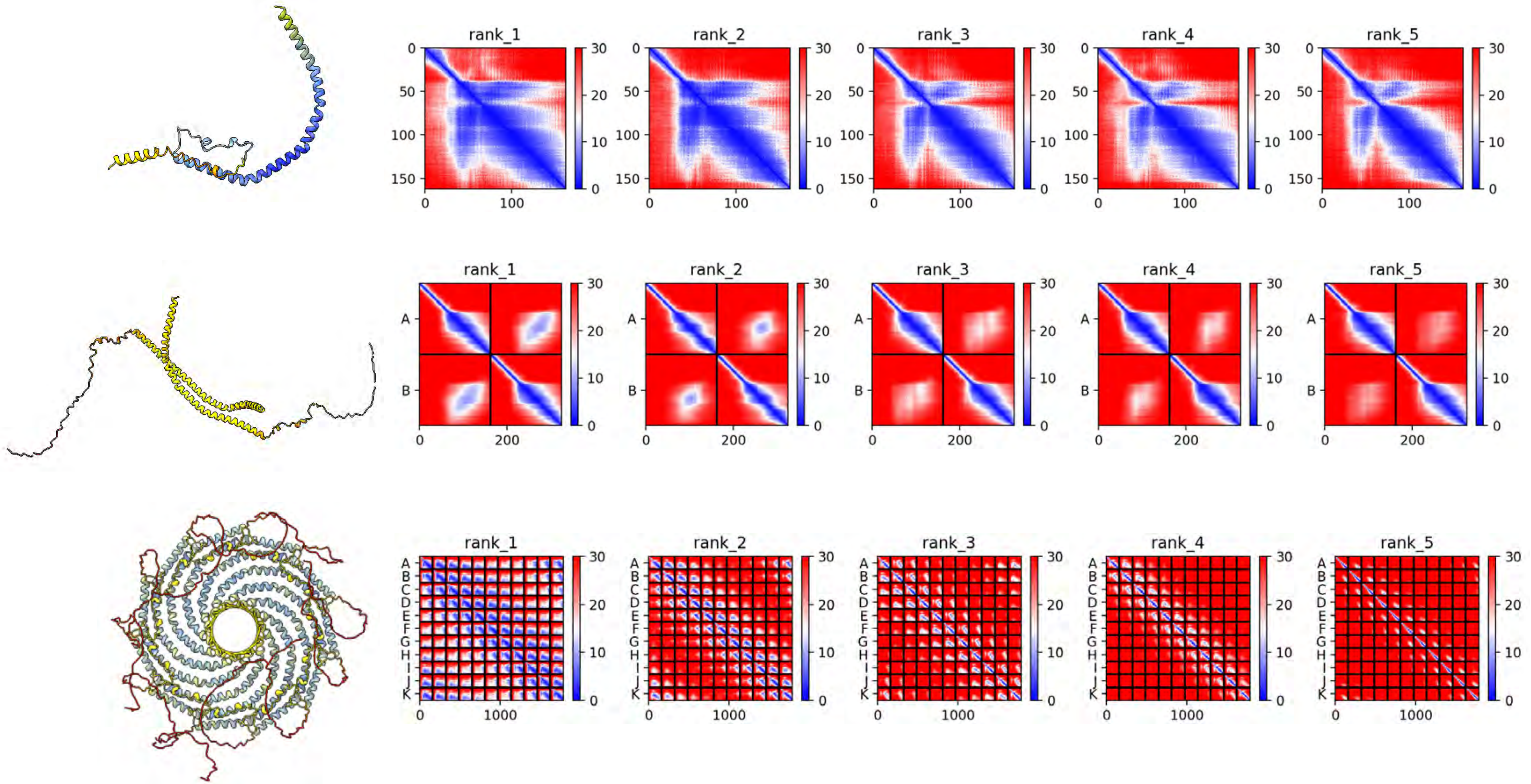

P56539 (CAV3) *H. sapiens*

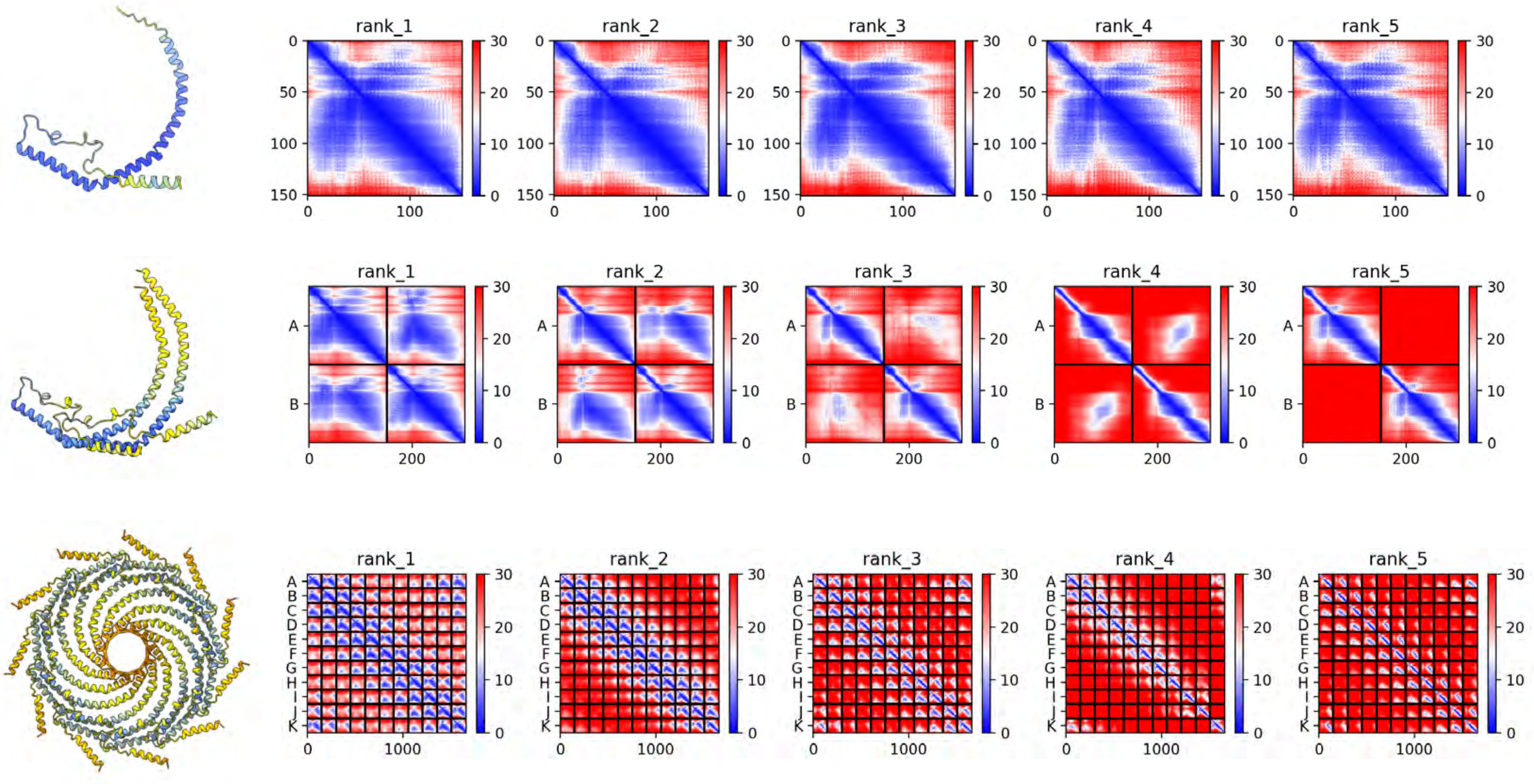

Q03135 (CAV1) *H. sapiens*

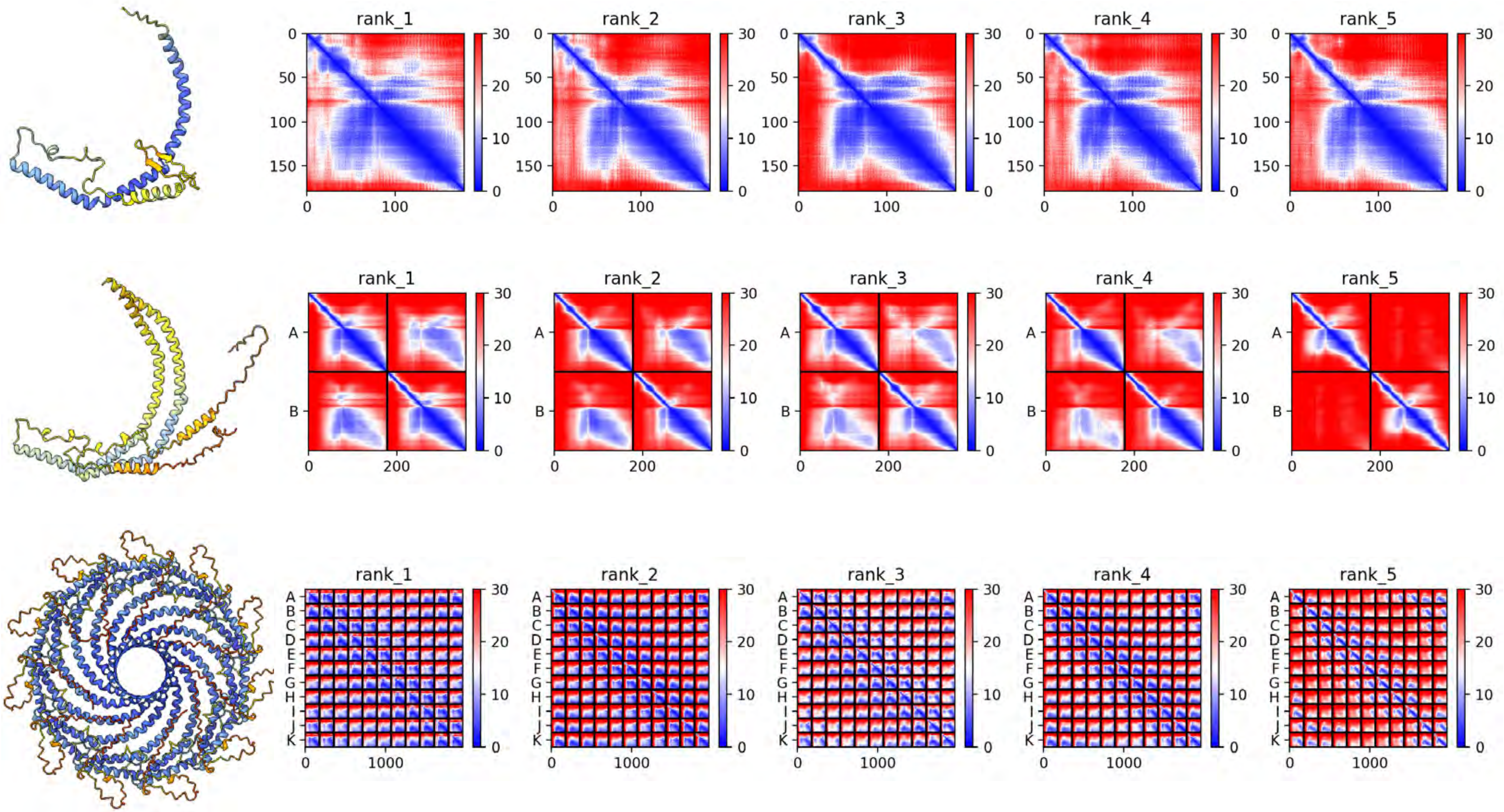

A0A1S3HK30 *L. unguis*

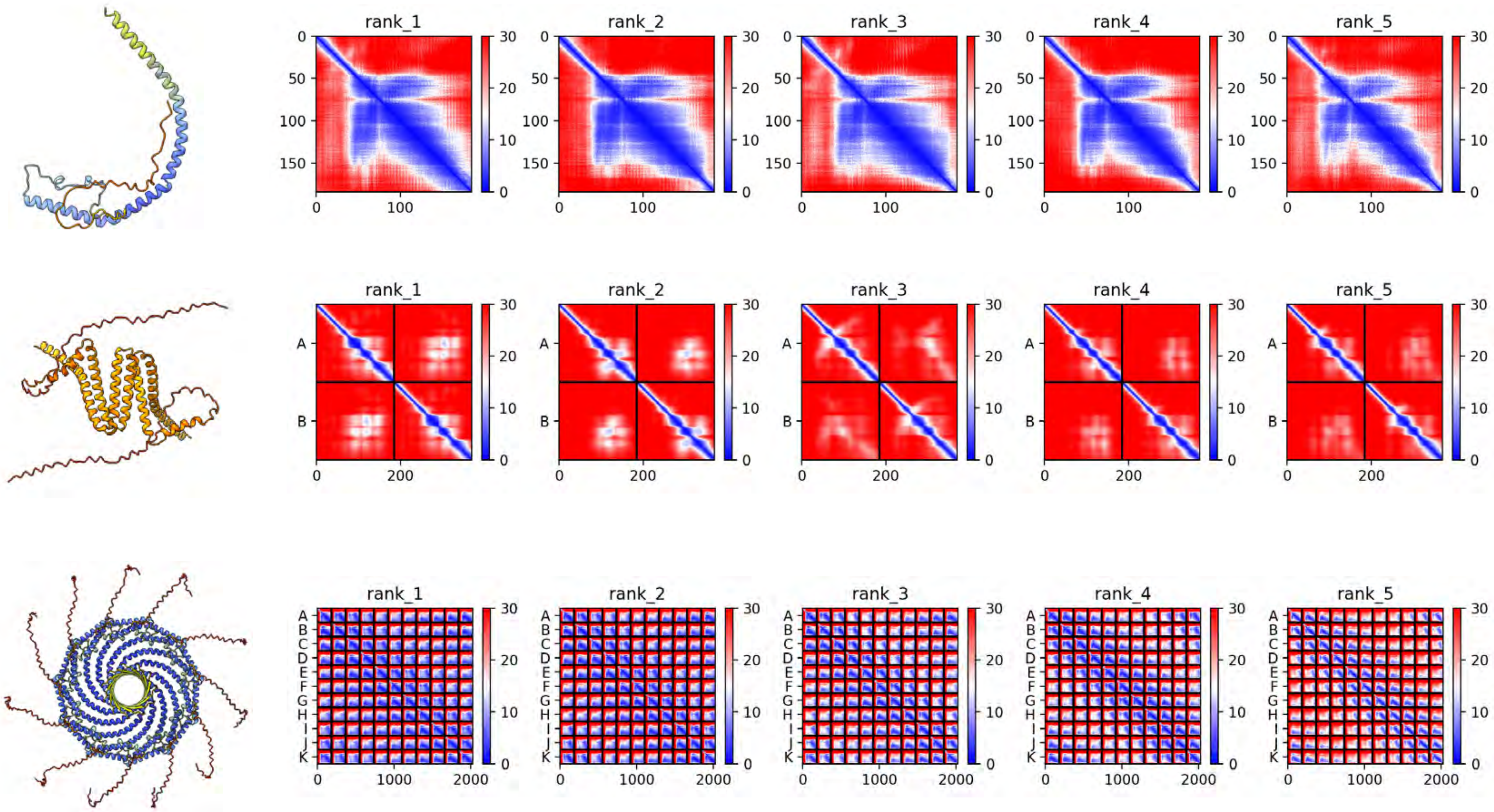

A0A1S3JHP2 *L. unguis*

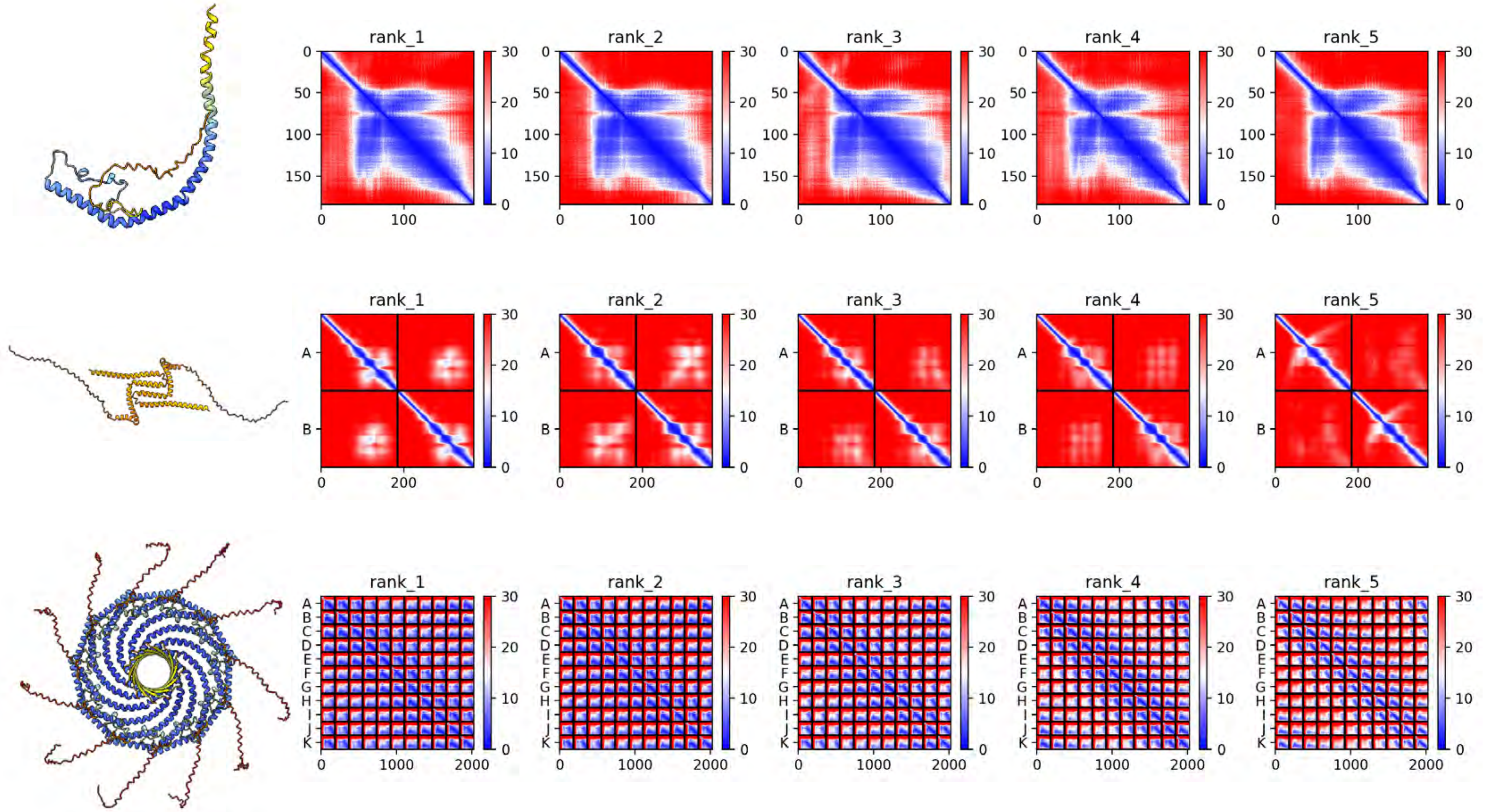

A0A1S3IQQ3 *L. unguis*

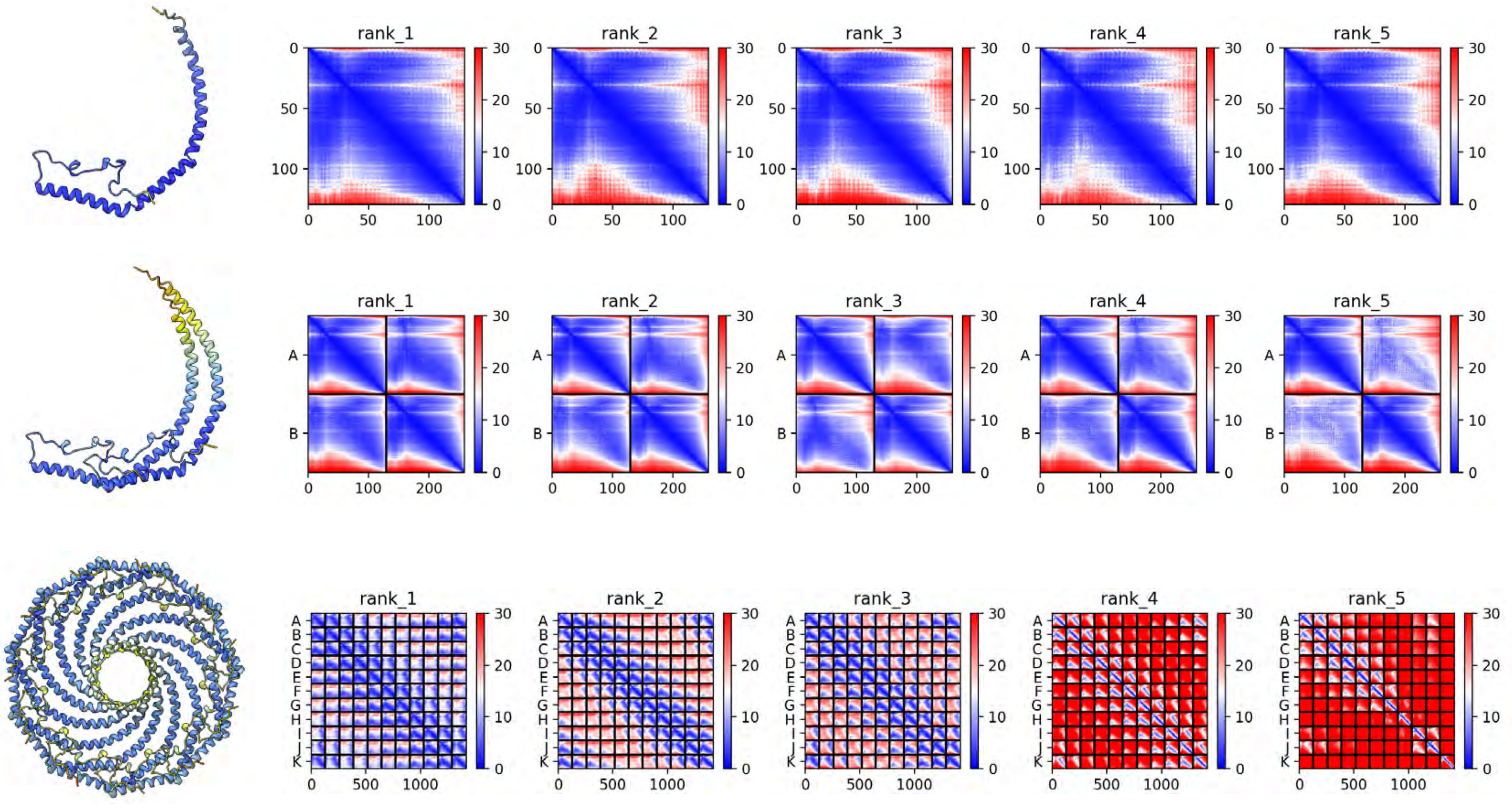

A0A1S3IG00 *L. unguis*

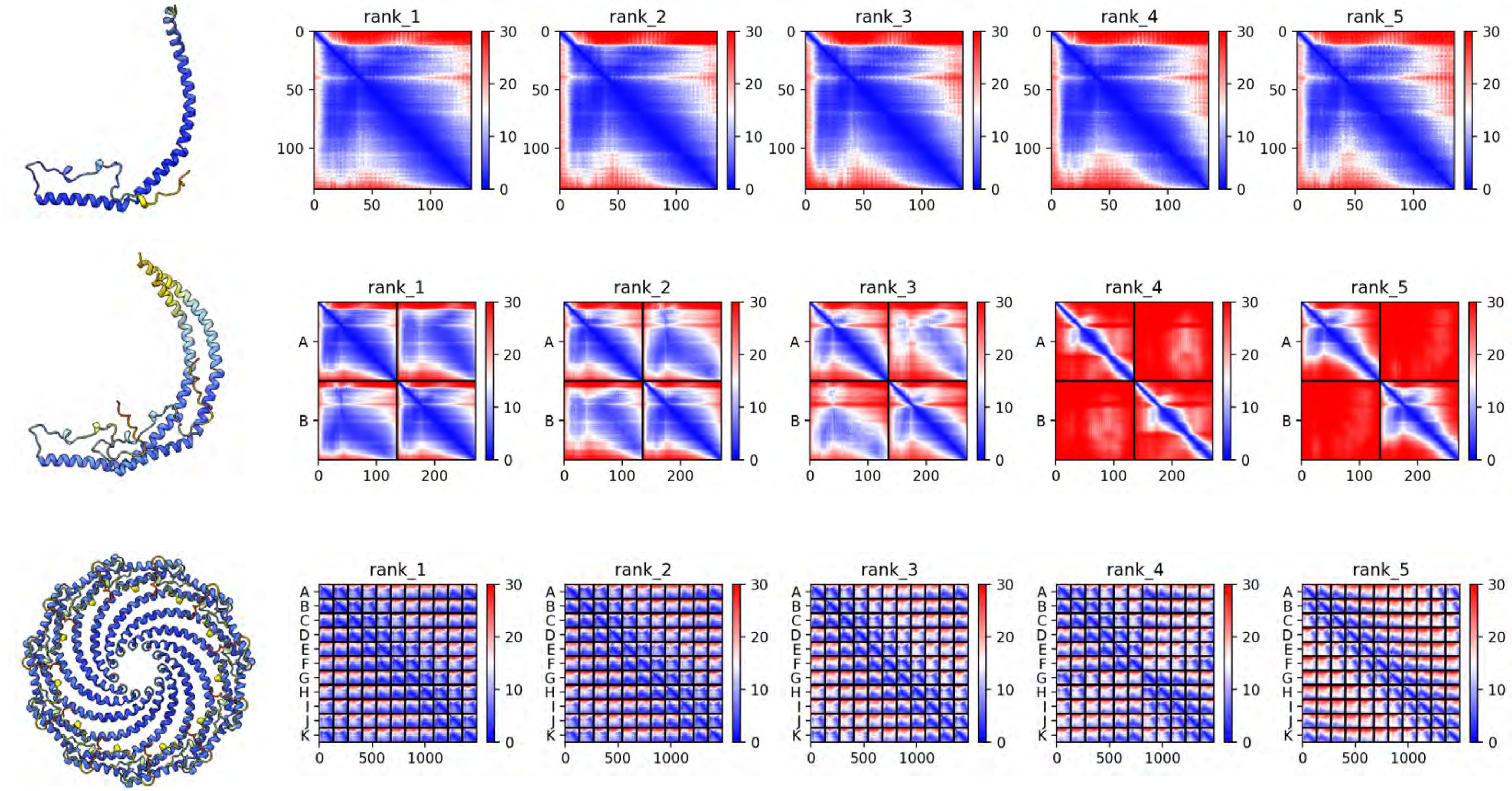

A0A1S3IG42 *L. unguis*

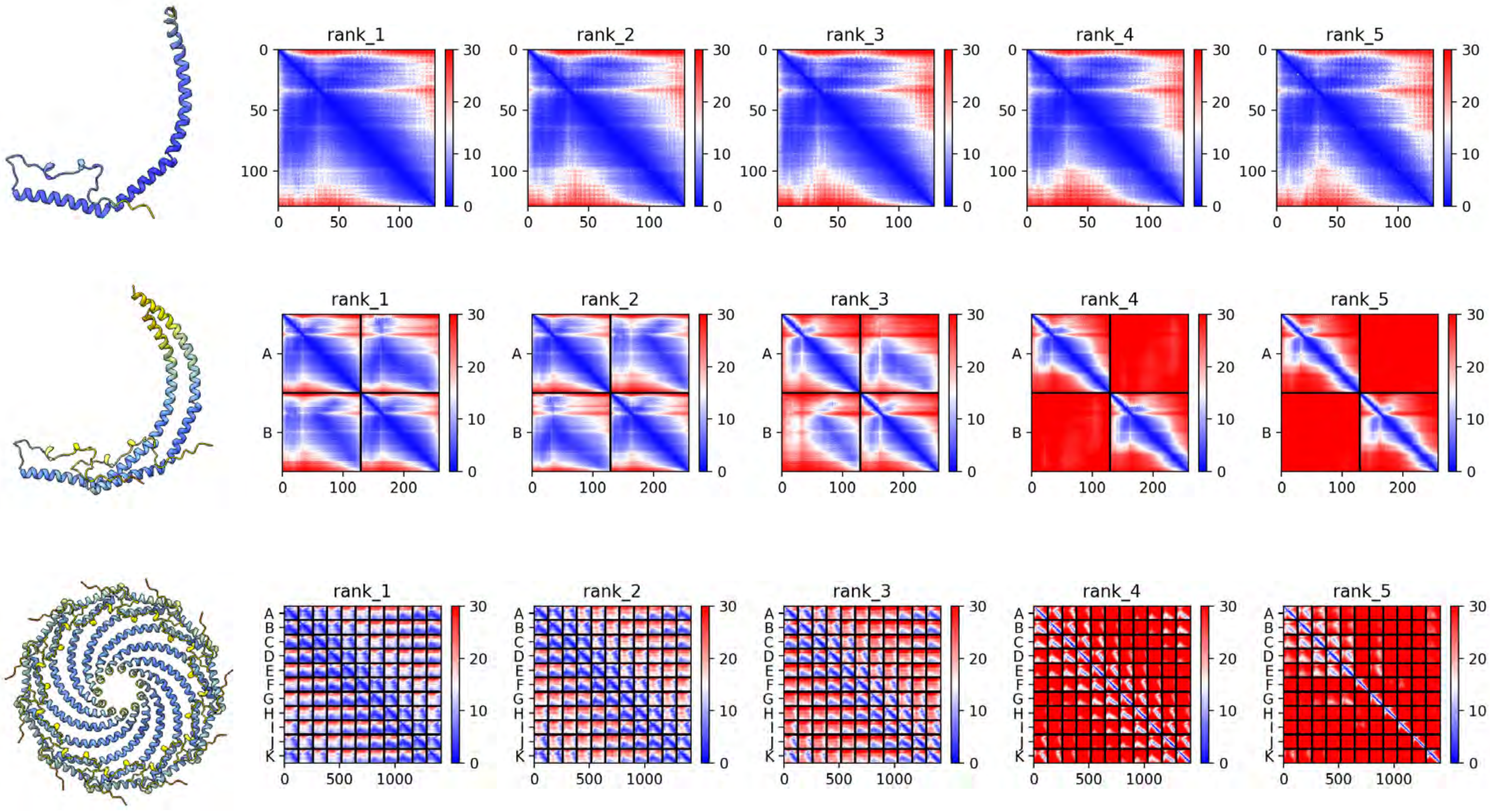

A0A1S3IH41 *L. unguis*

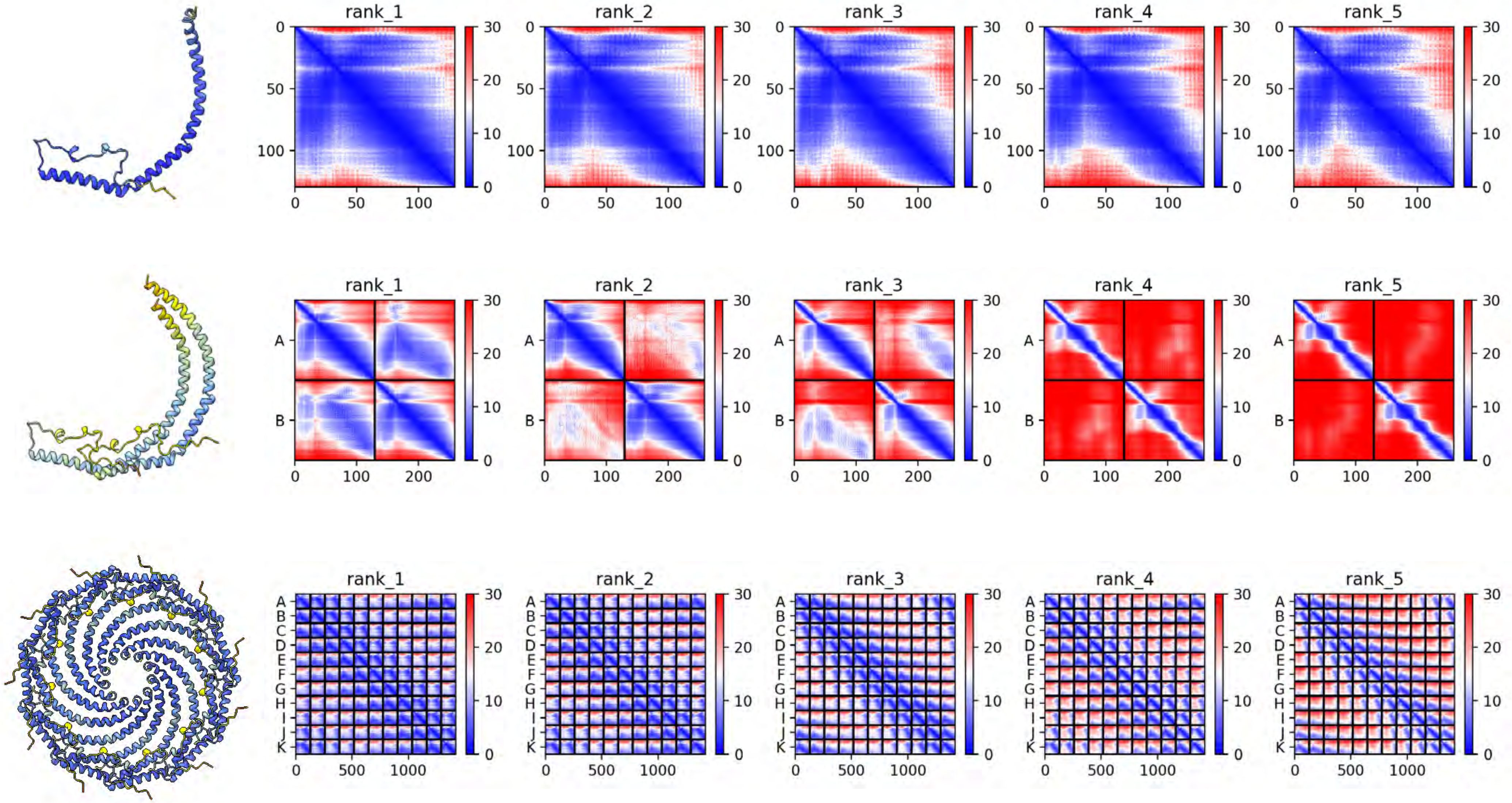

XP_014678552.1 *P. caudatus*

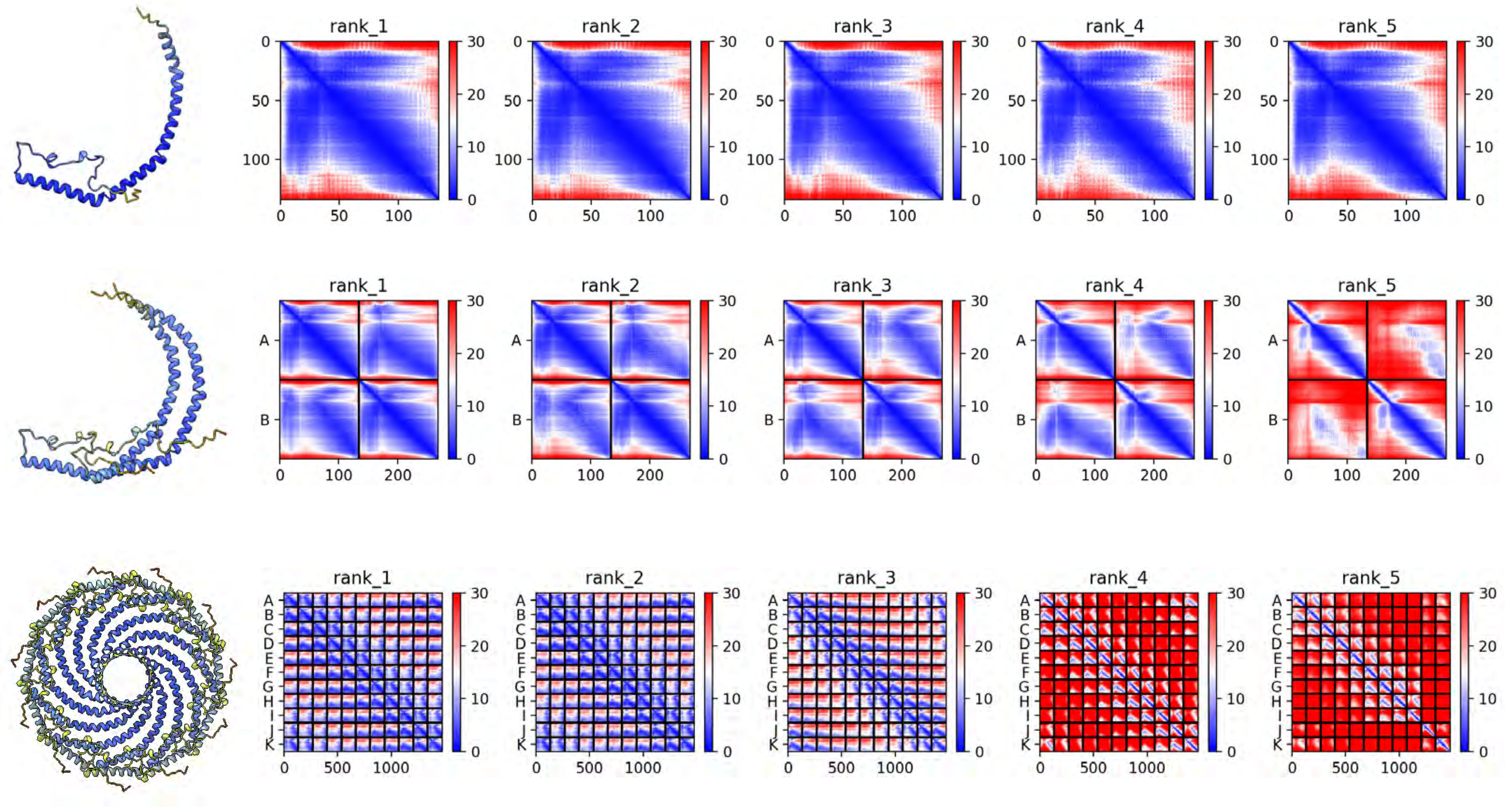

A0A7M7R2L2 *A. mellifera*

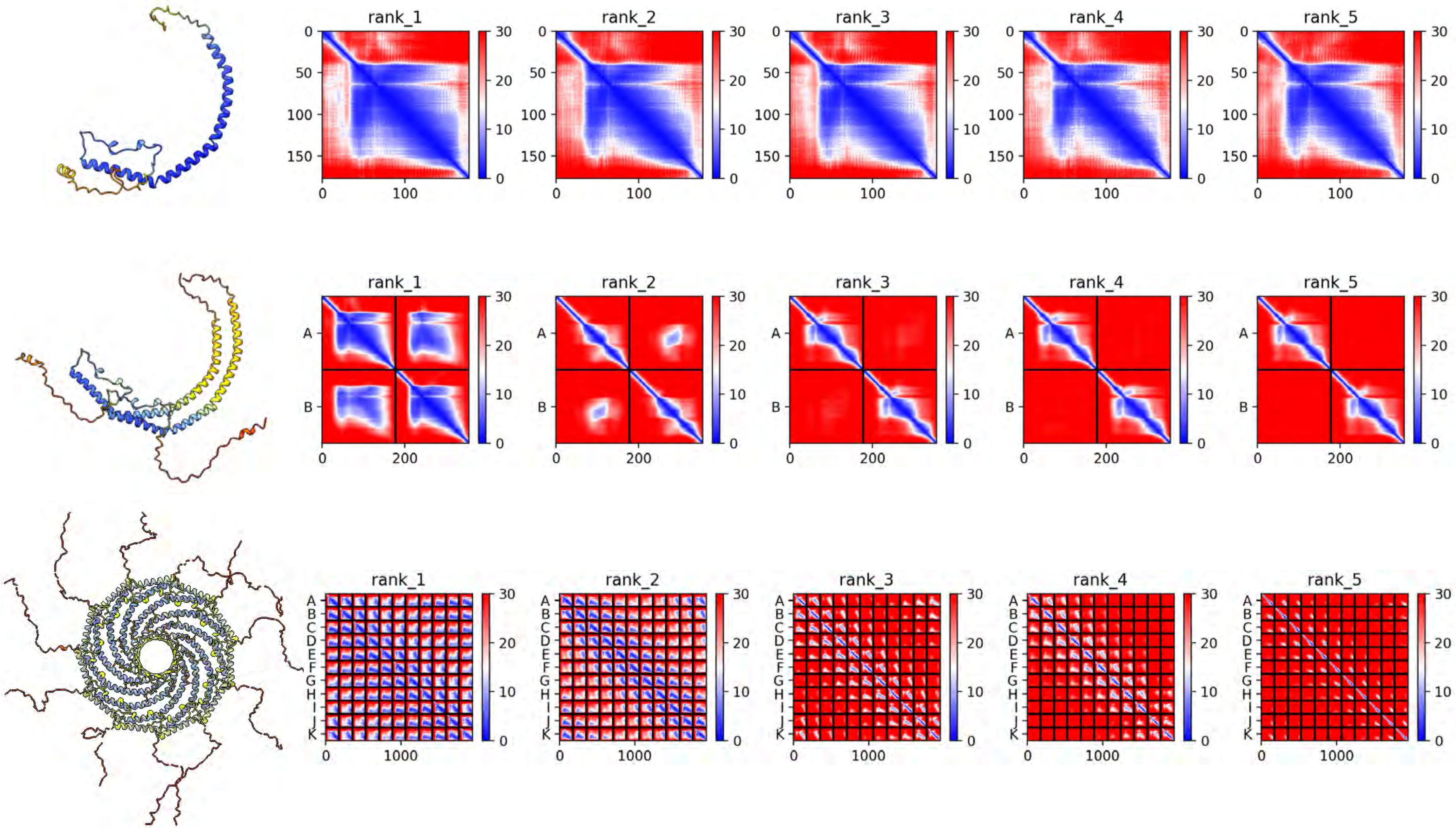

A0A3M7SAF6 *B. plicatilis*

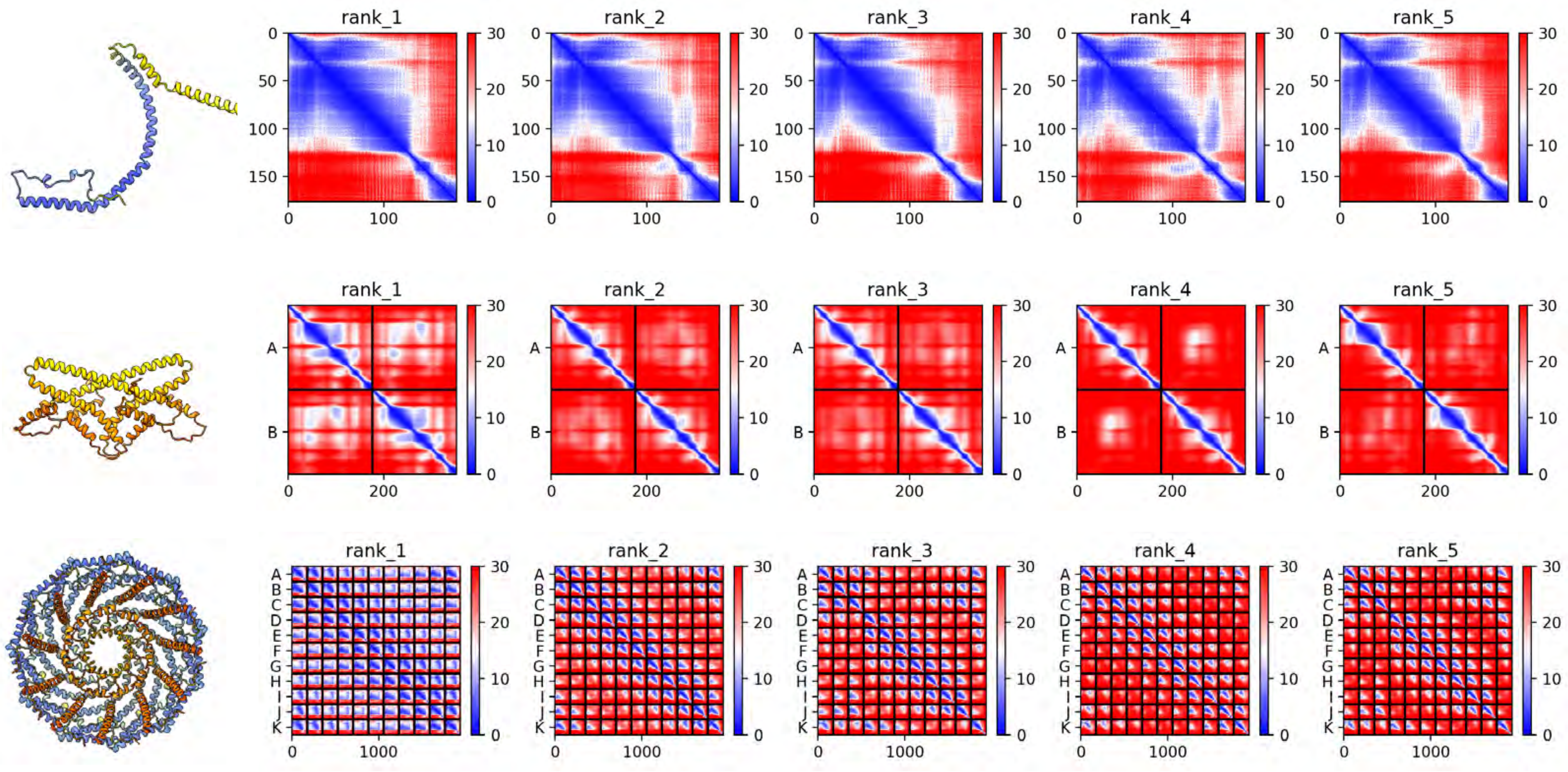

A0A1I8ILQ8 *M. lignano*

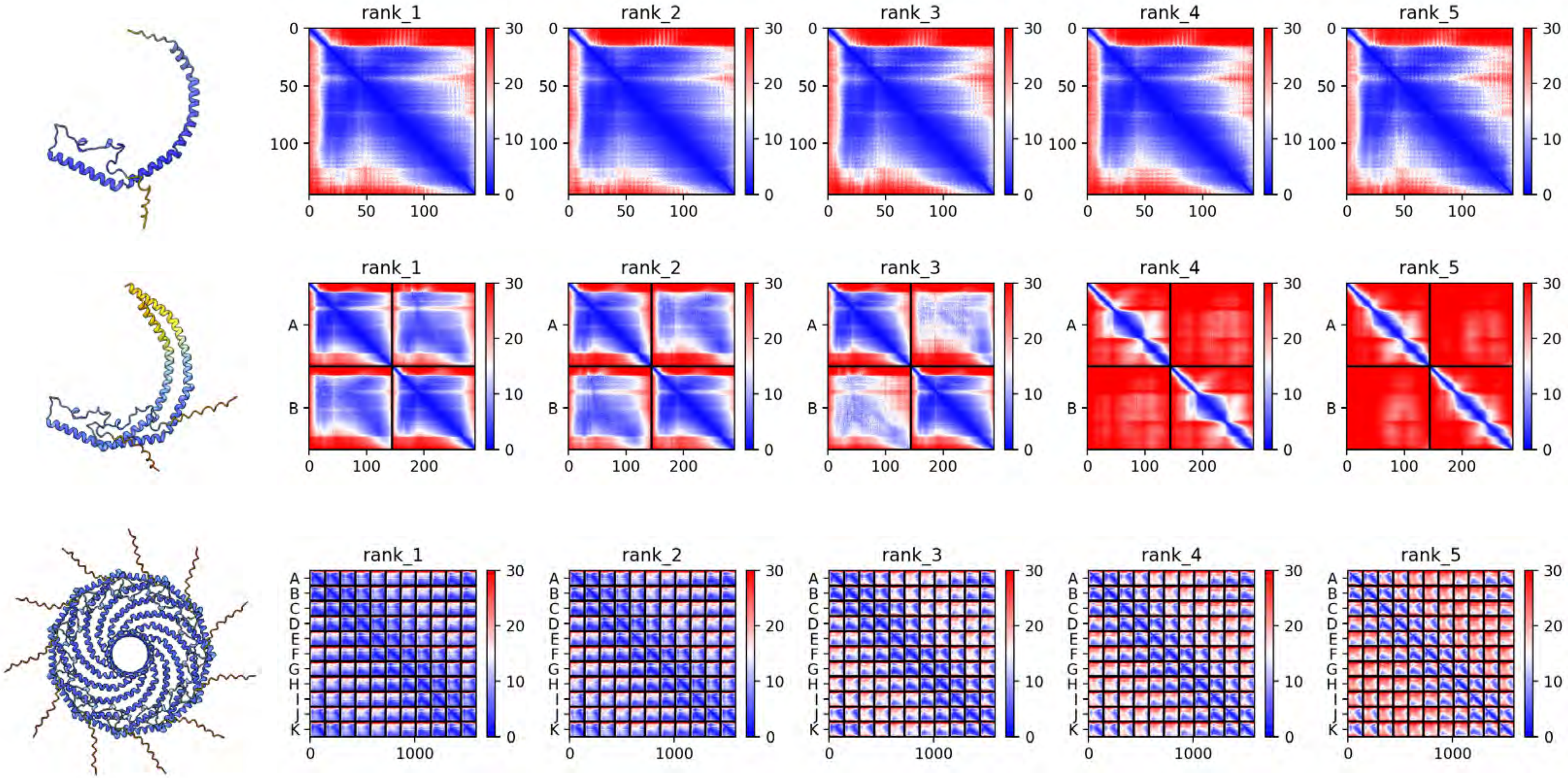

A0A1I8HHY8 *M. lignano*

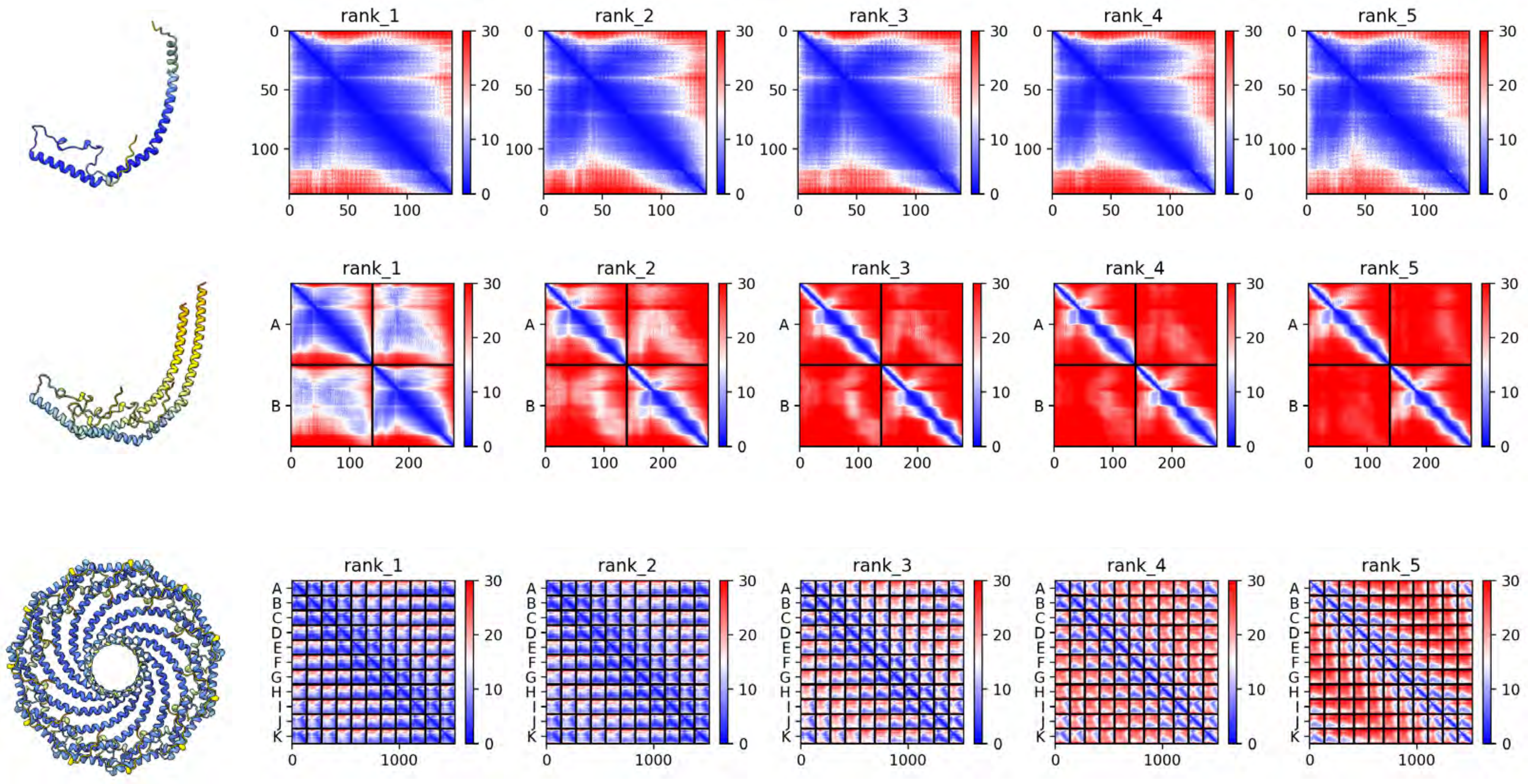

A0A267DBT9 *M. lignano*

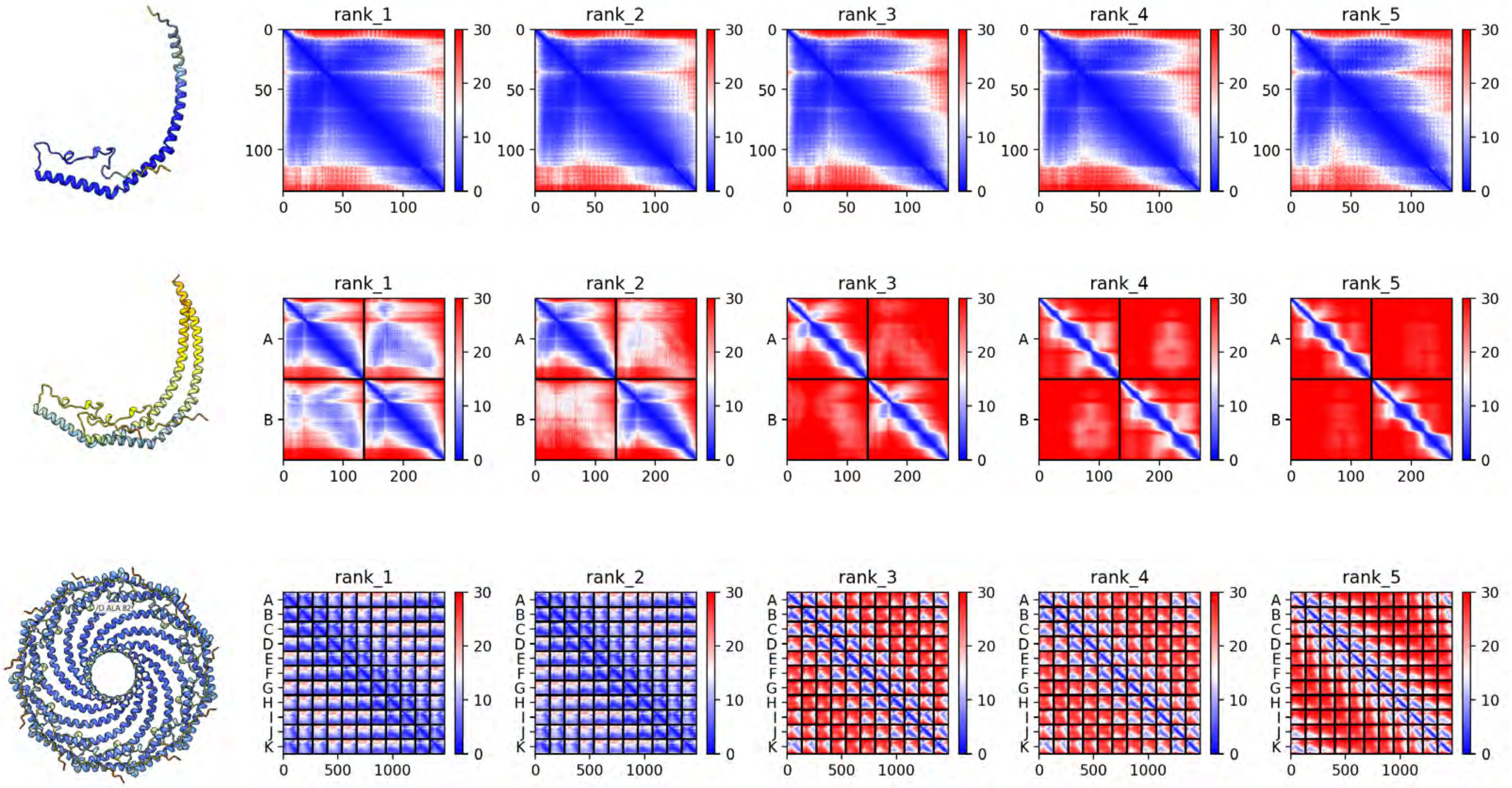

A0A267F2S4 *M. lignano*

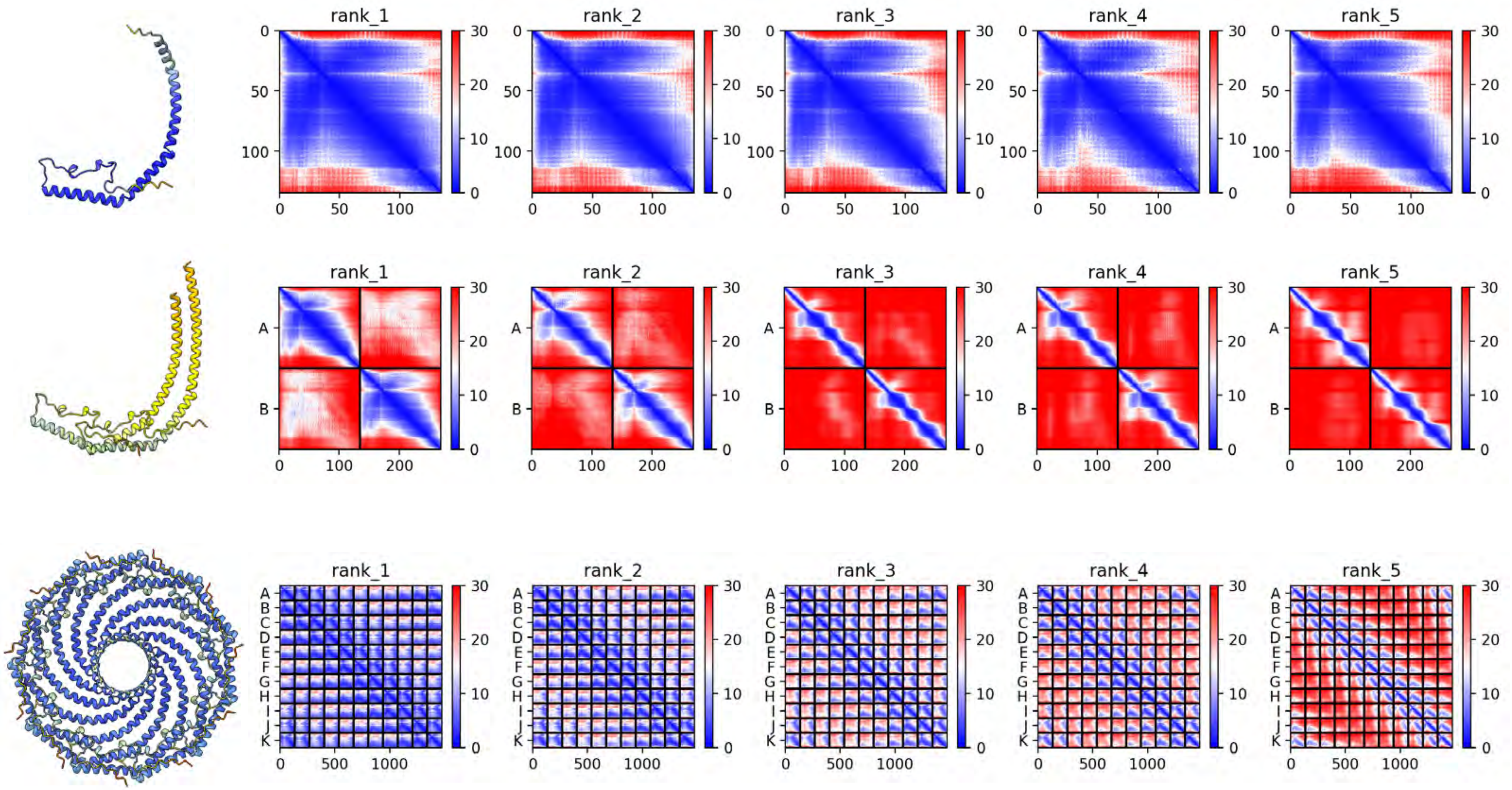

A0A3M7SB10 *B. plicatilis*

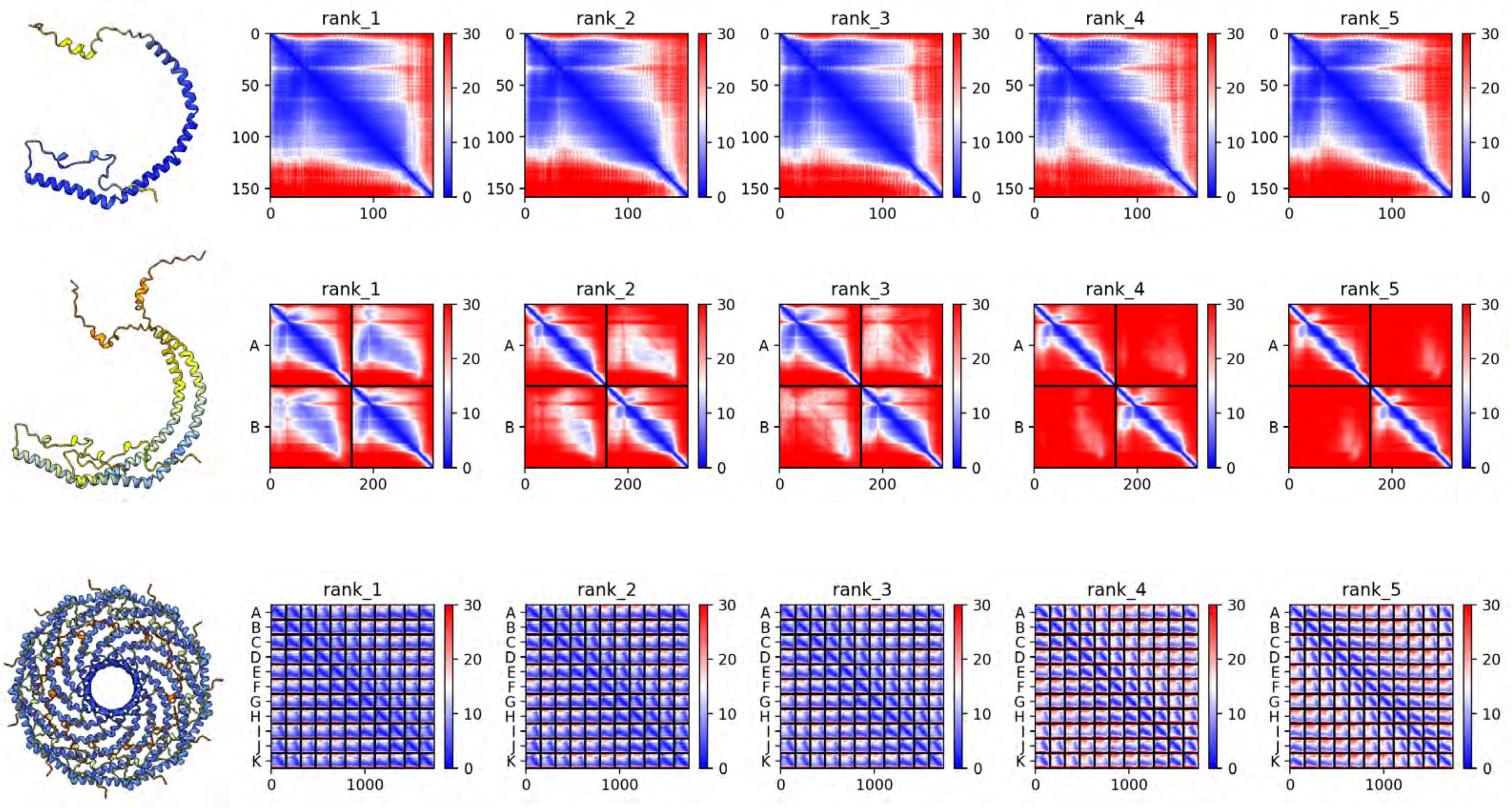

A0A3M7Q0Q1 *B. plicatilis*

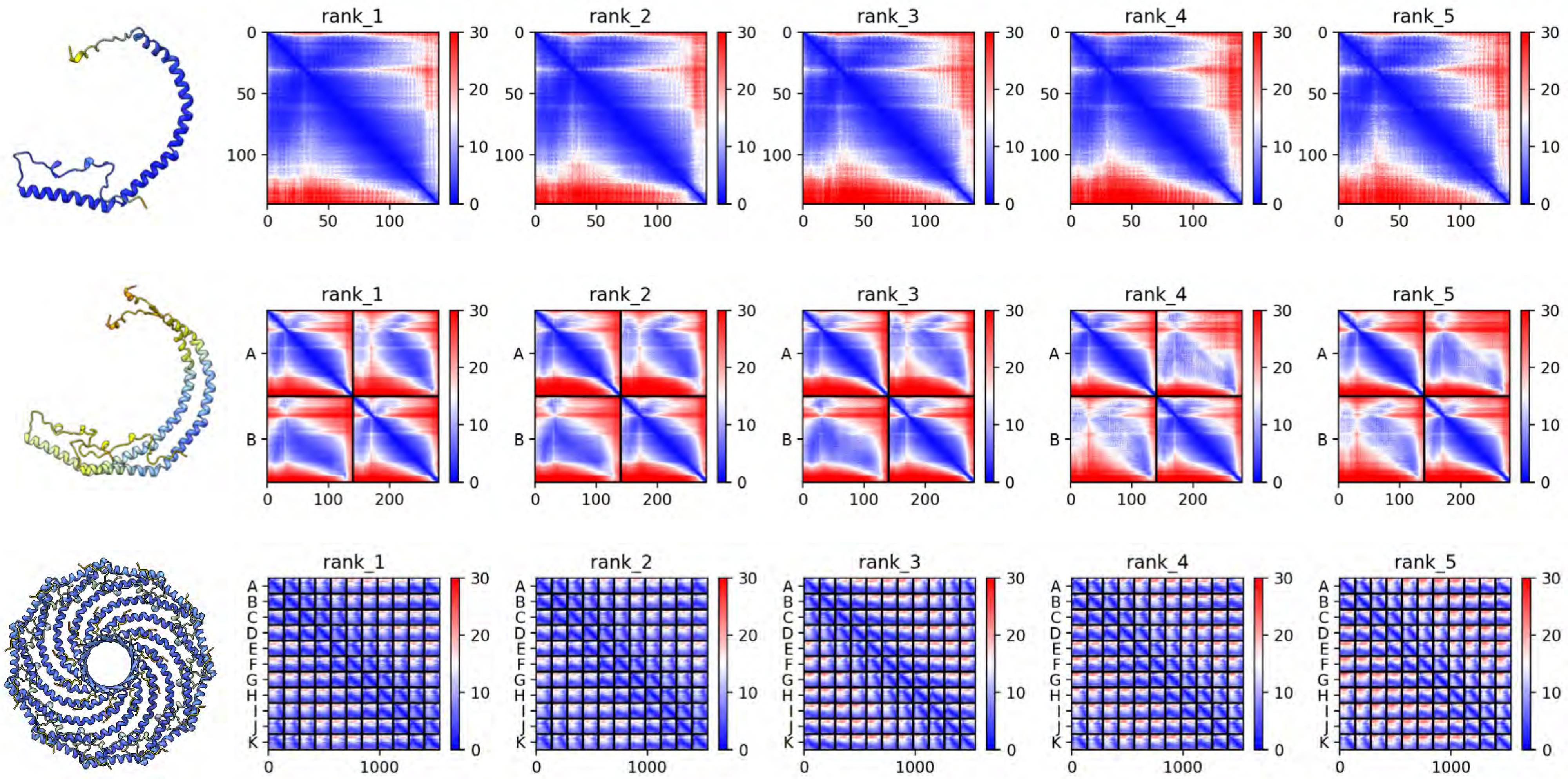

A0A3M7RD35 *B. plicatilis*

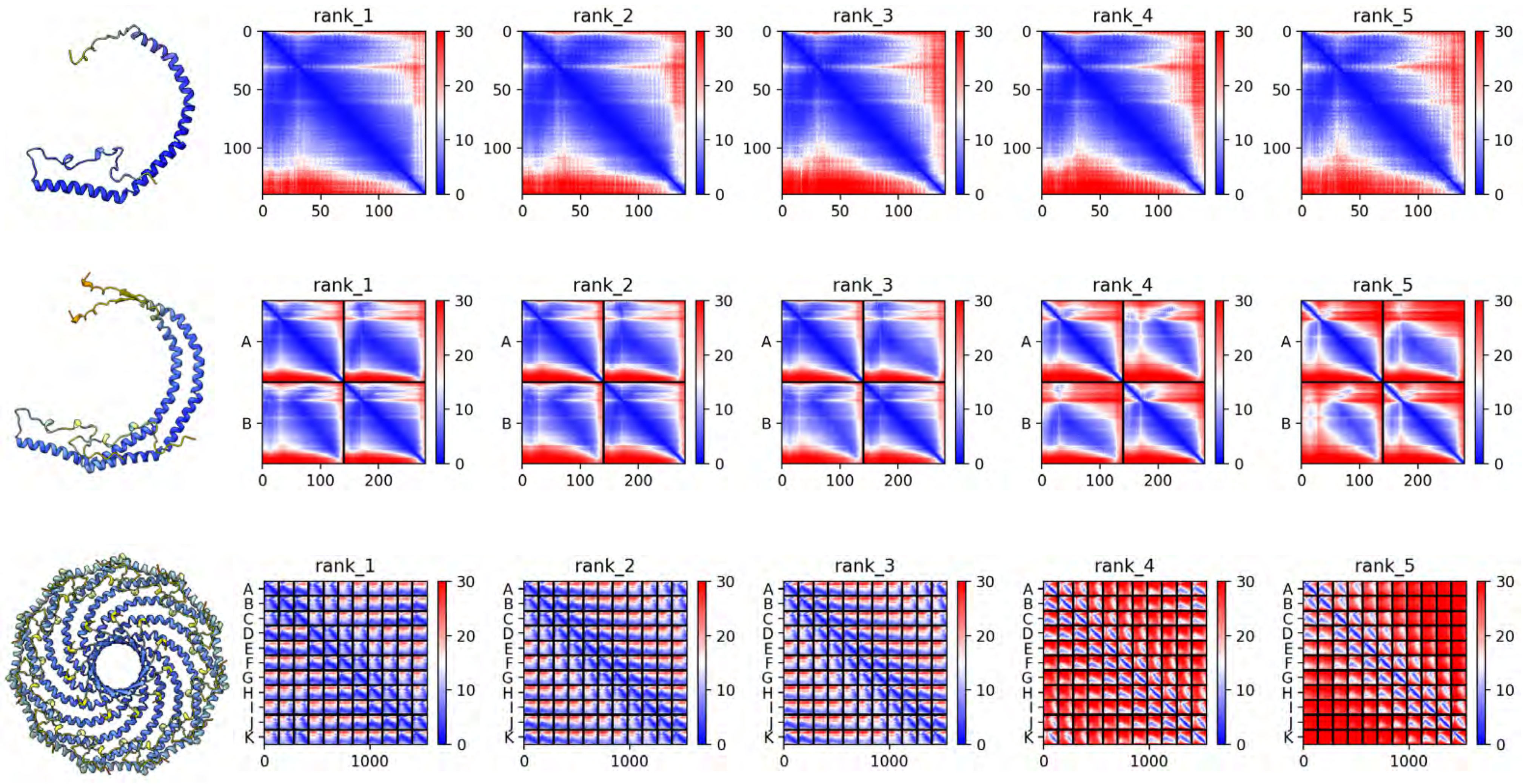

A0A3M7RCJ9 *B. plicatilis*

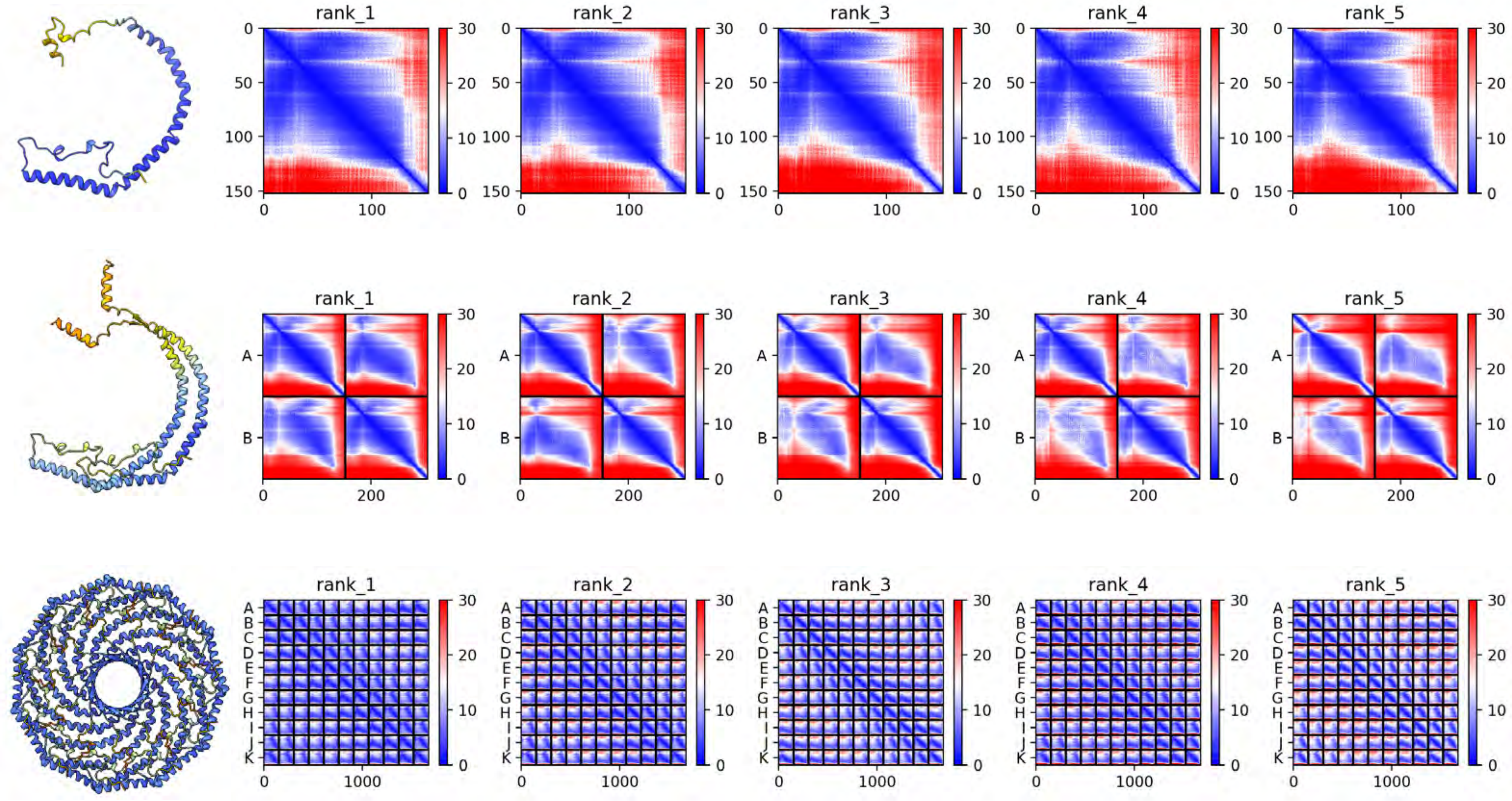

A0A3M7SAK0 *B. plicatilis*

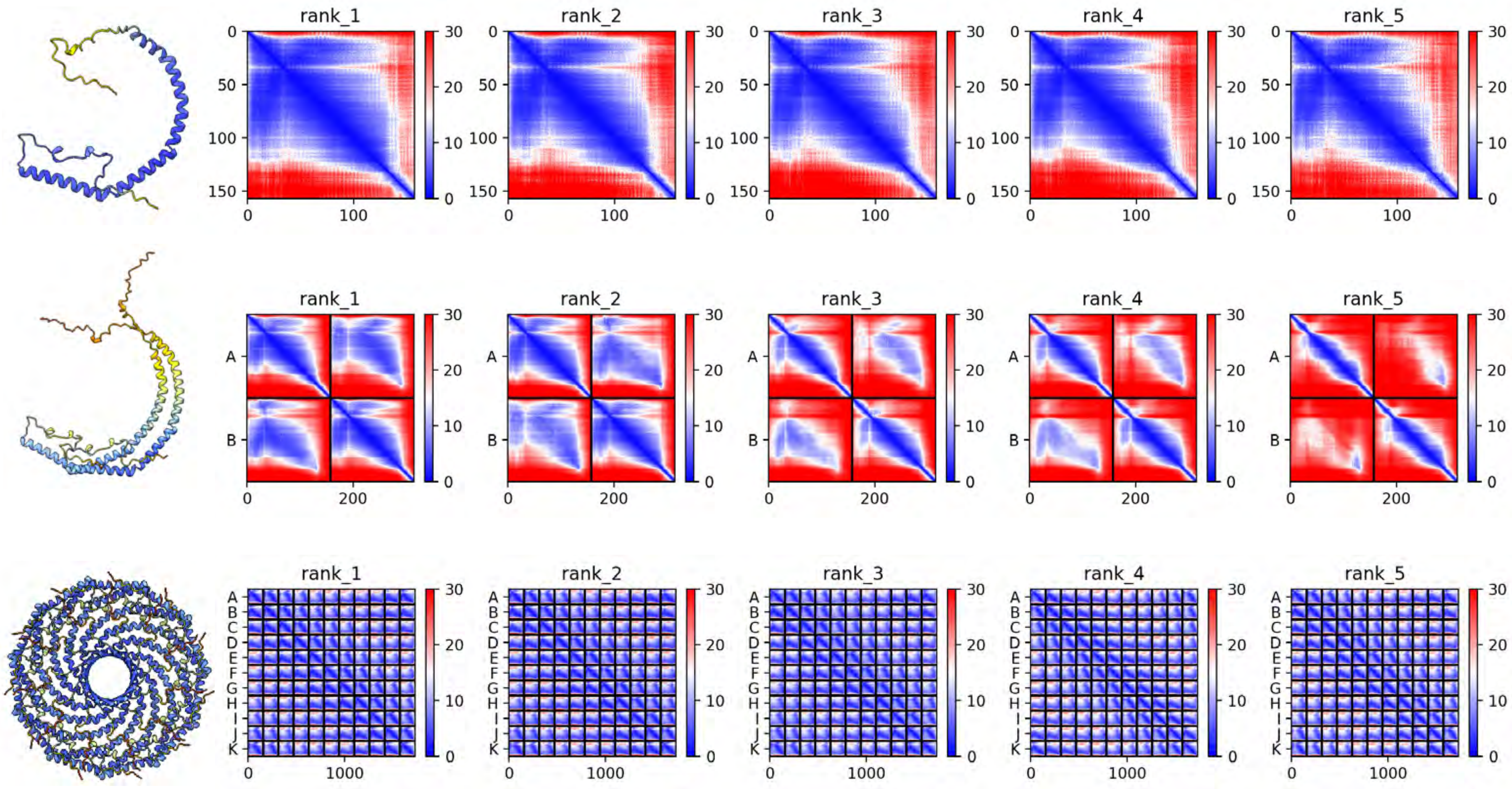

A0A1I8H2T4 *M. lignano*

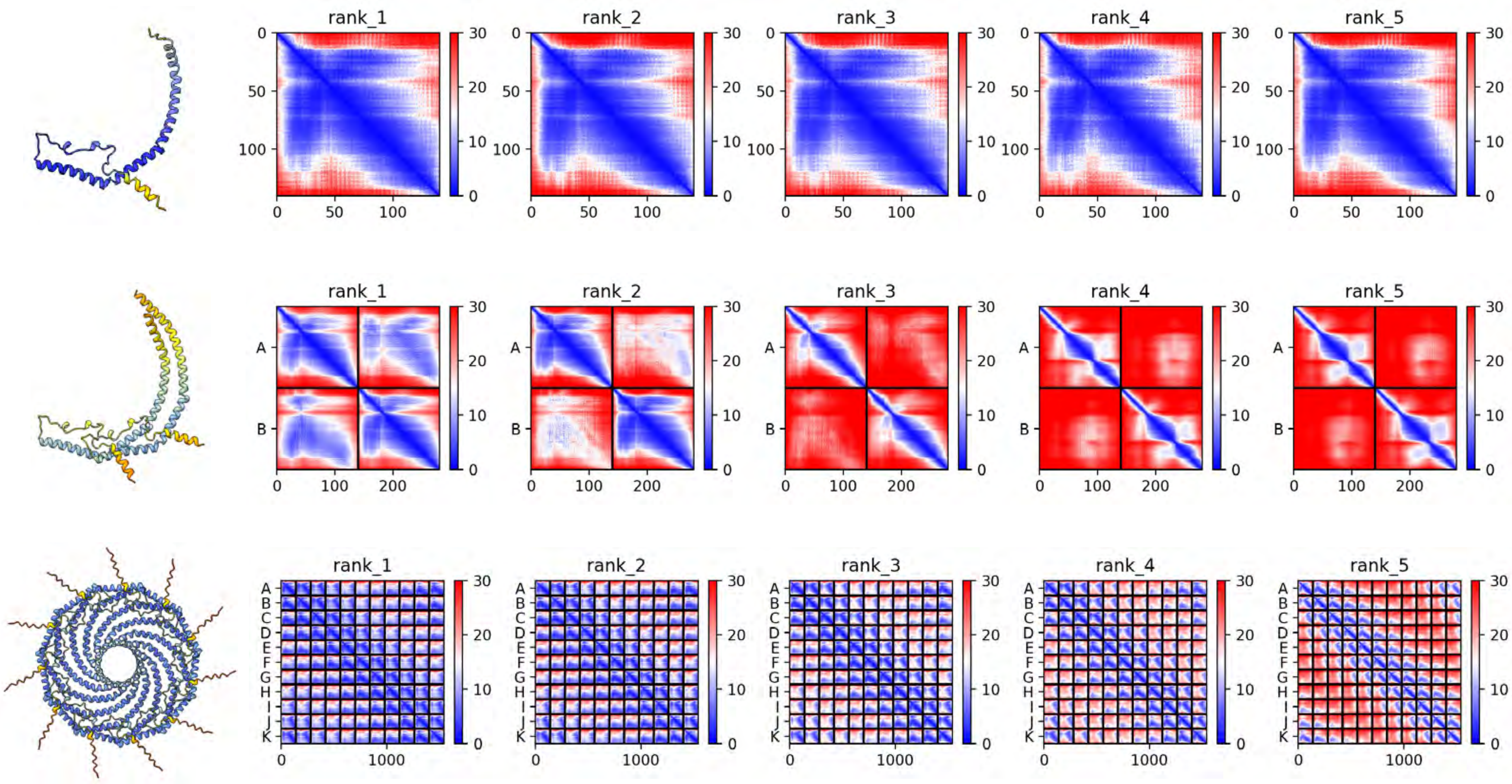

A0A1I8H327 *M. lignano*

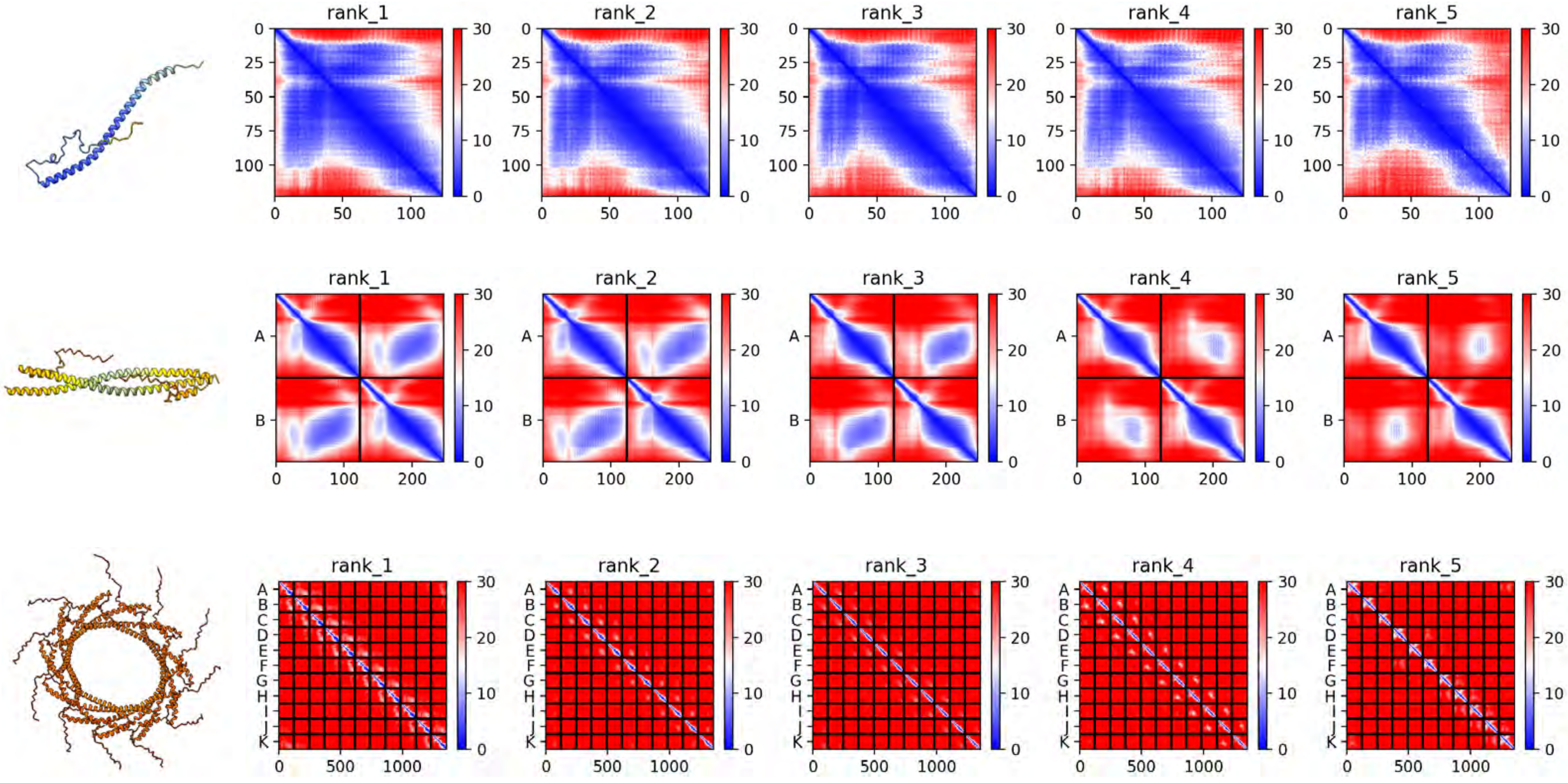

A0A267DS21 *M. lignano*

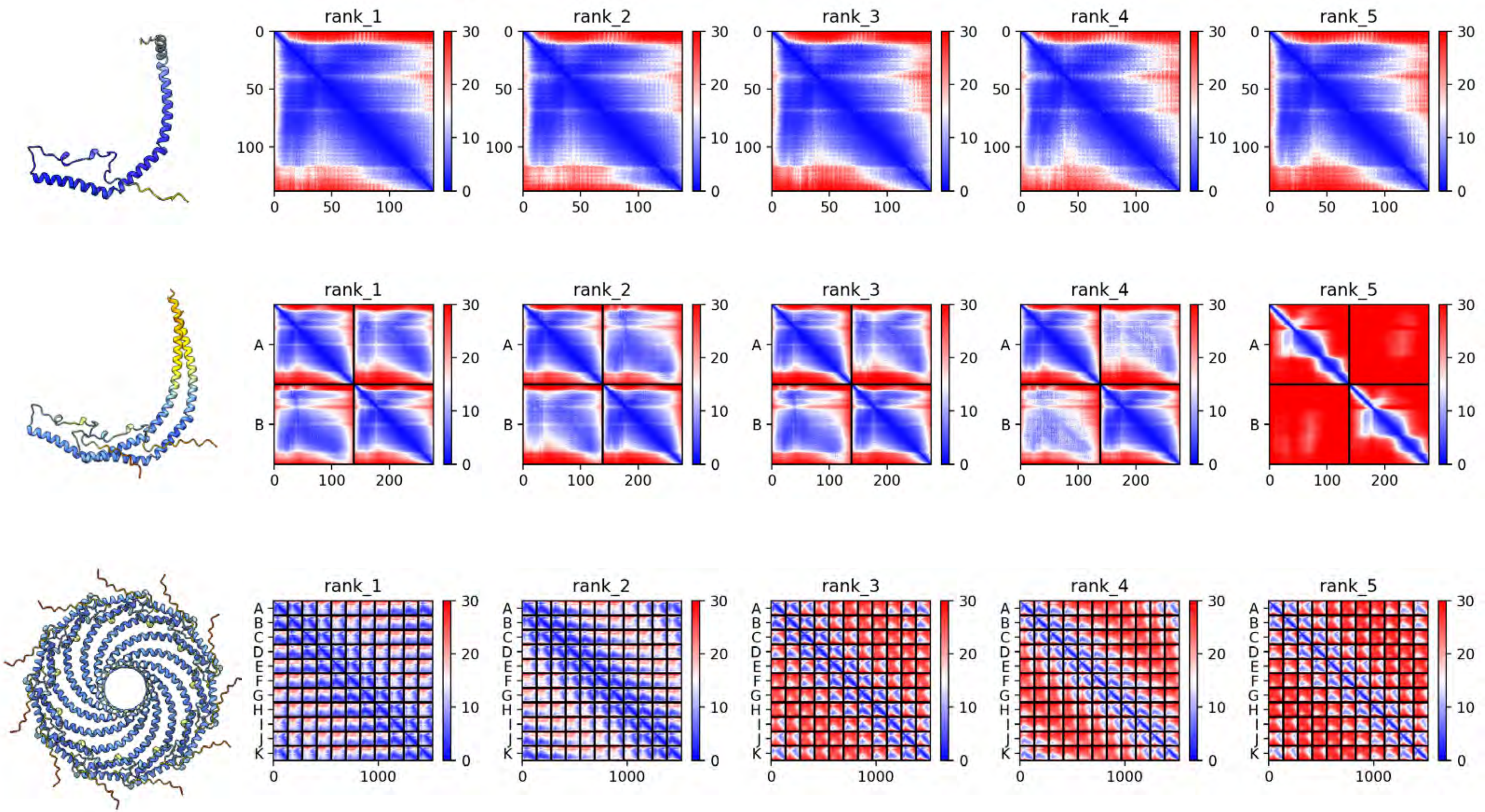

A0A267DU21 *M. lignano*

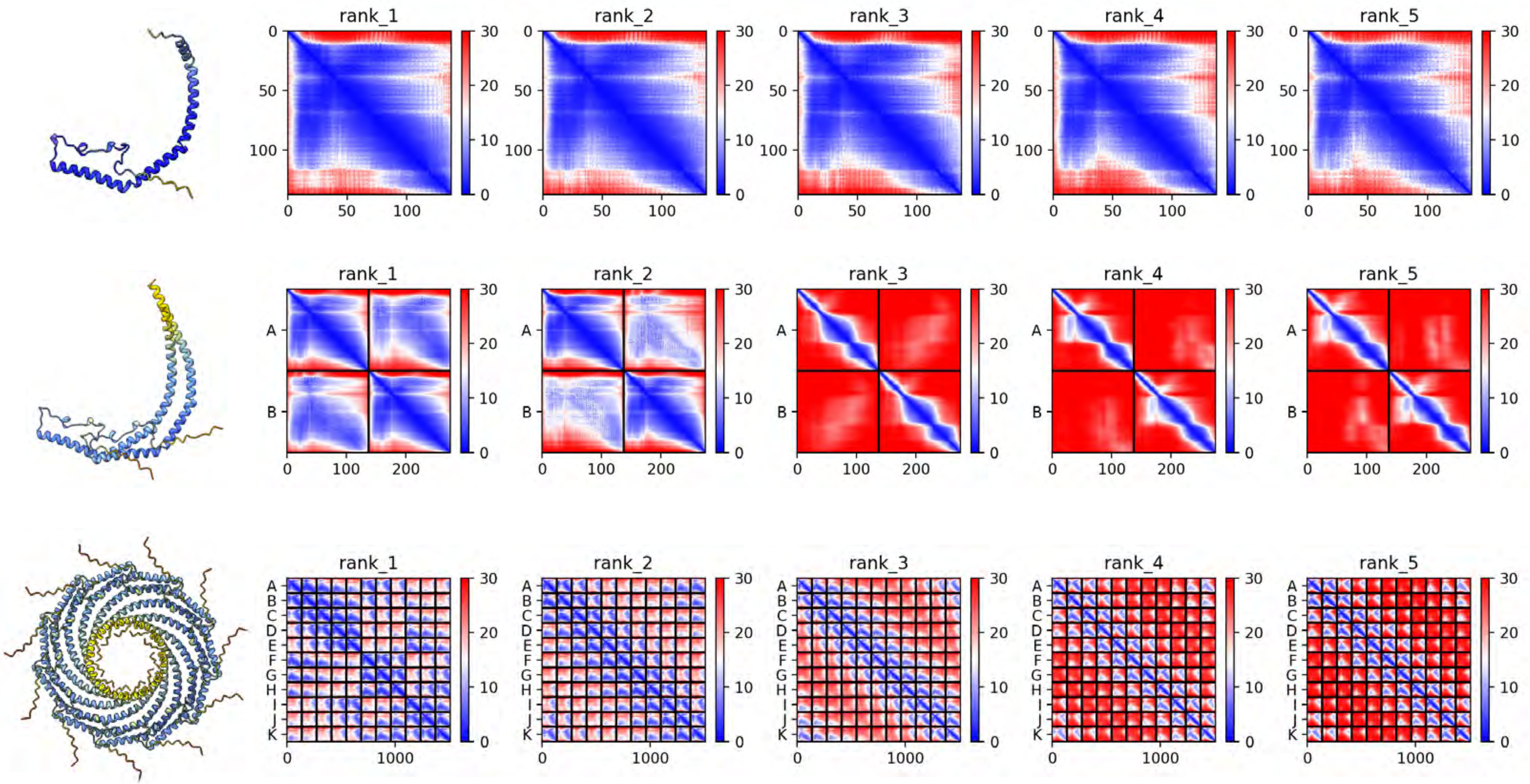

A0A1I8J849 *M. lignano*

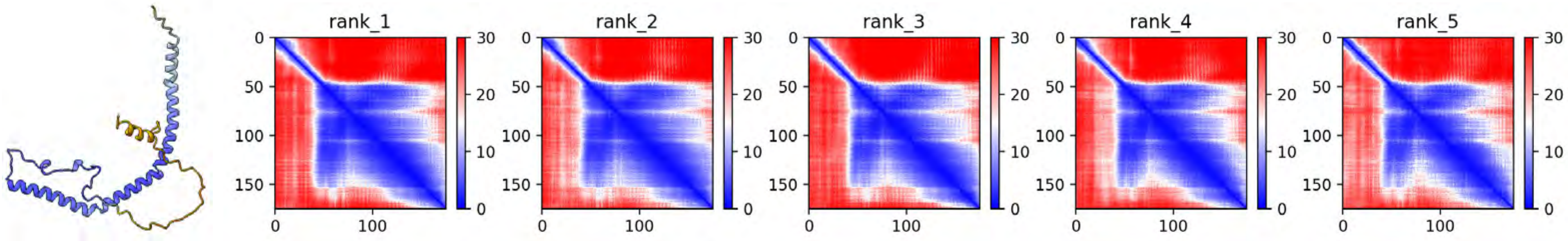

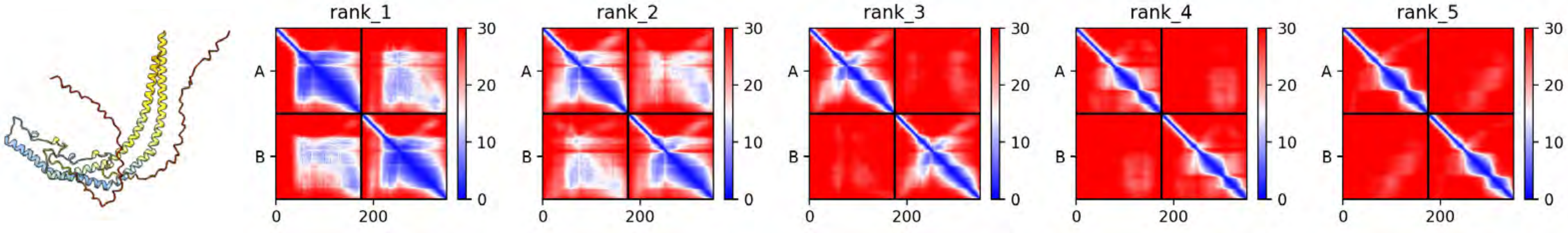

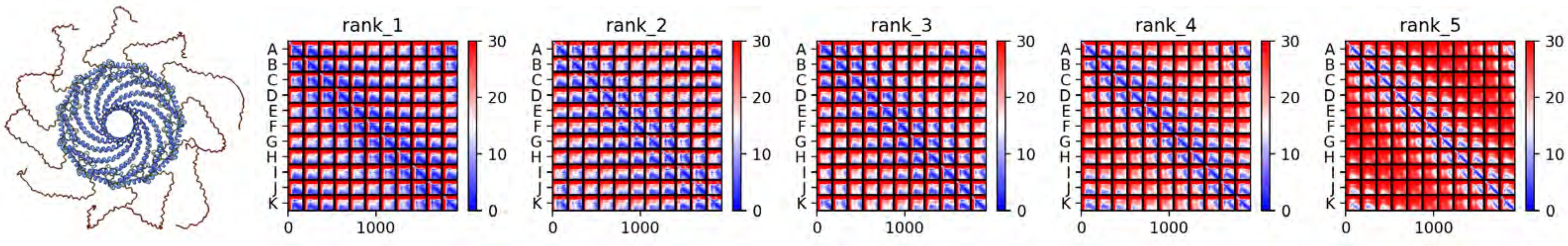

A0A267FNG7 *M. lignano*

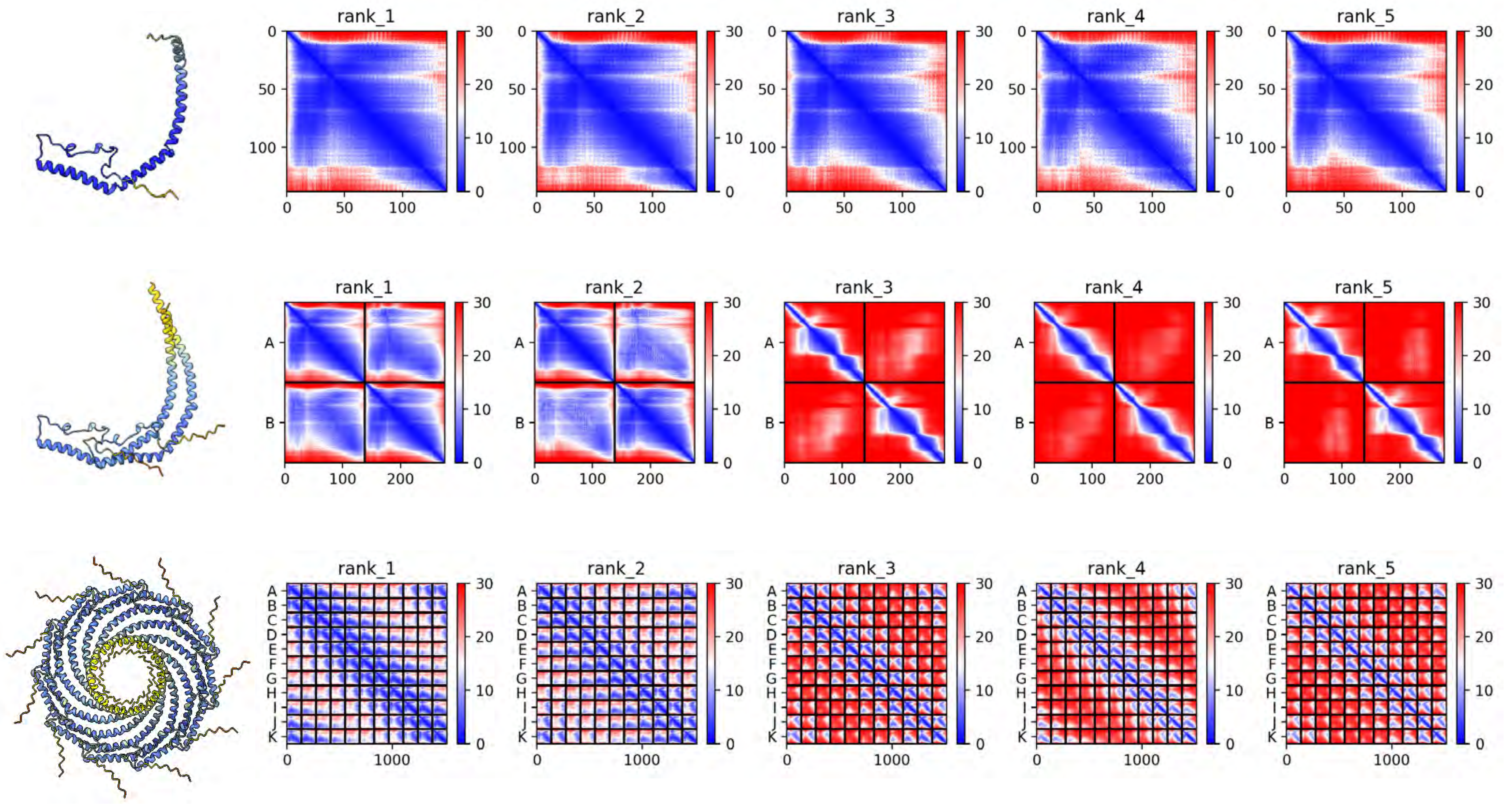

A0A1I8J741 *M. lignano*

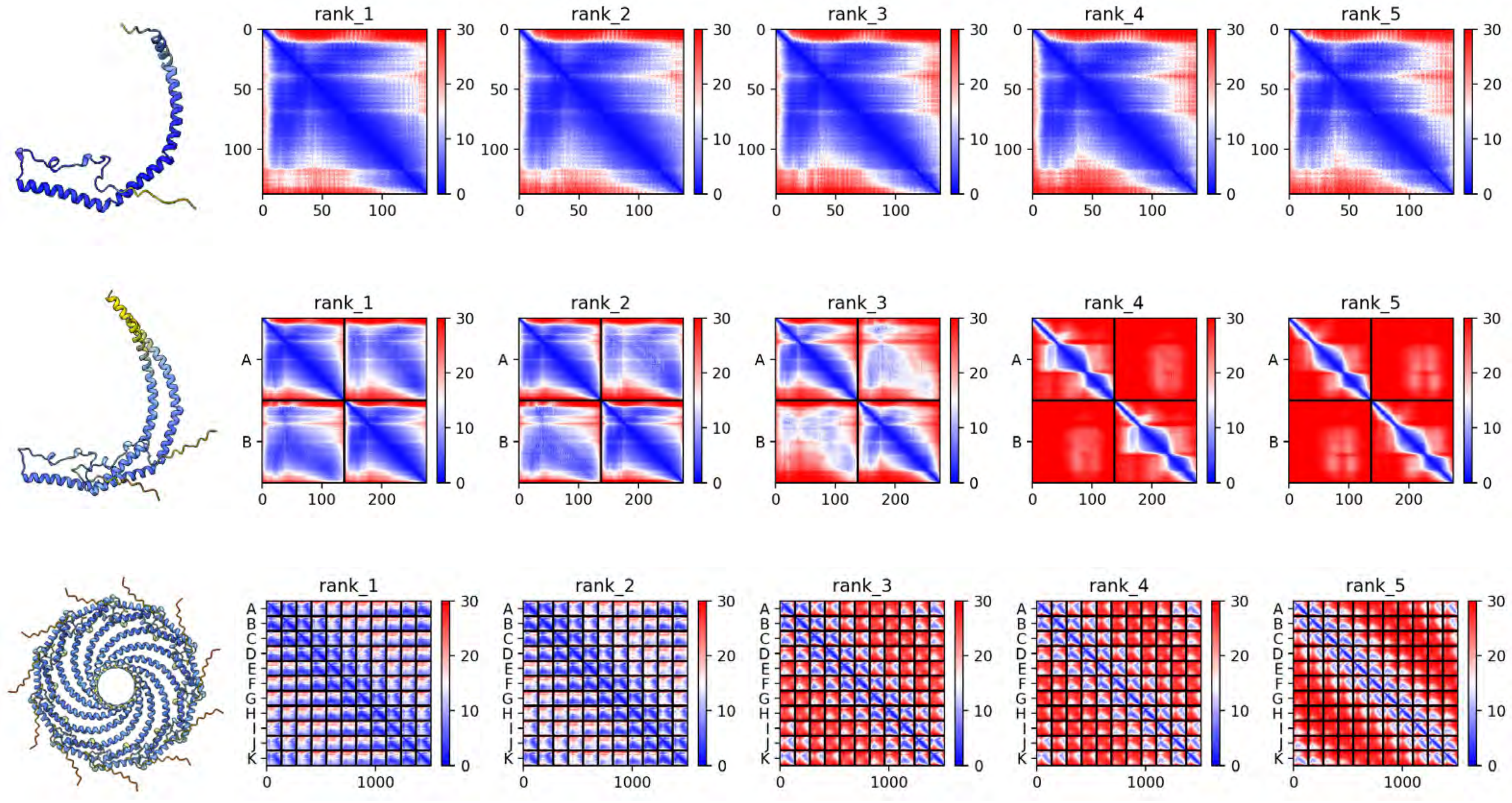

A0A267DC90 ***M. lignano***

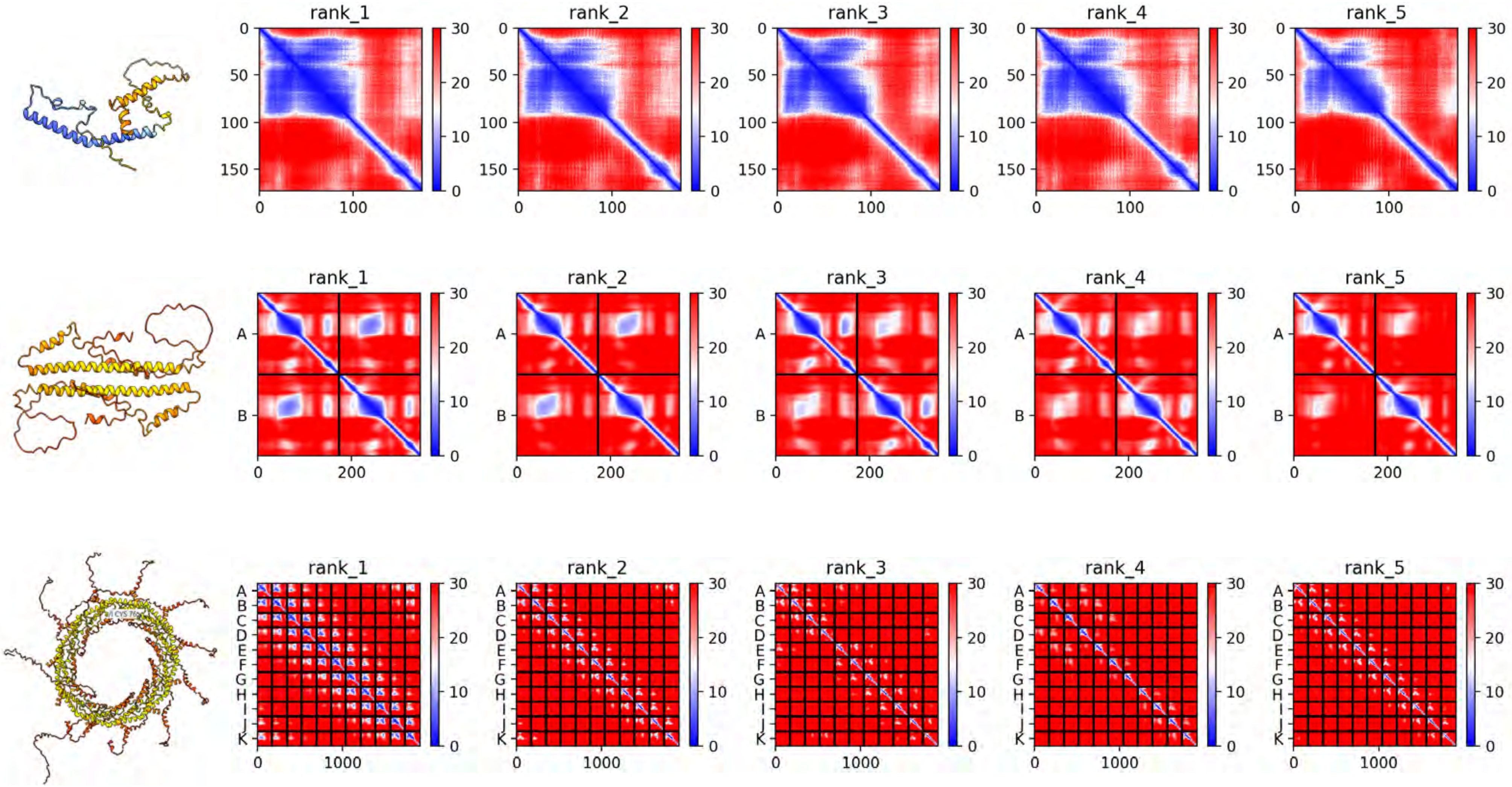

A0A267F6W6 *M. lignano*

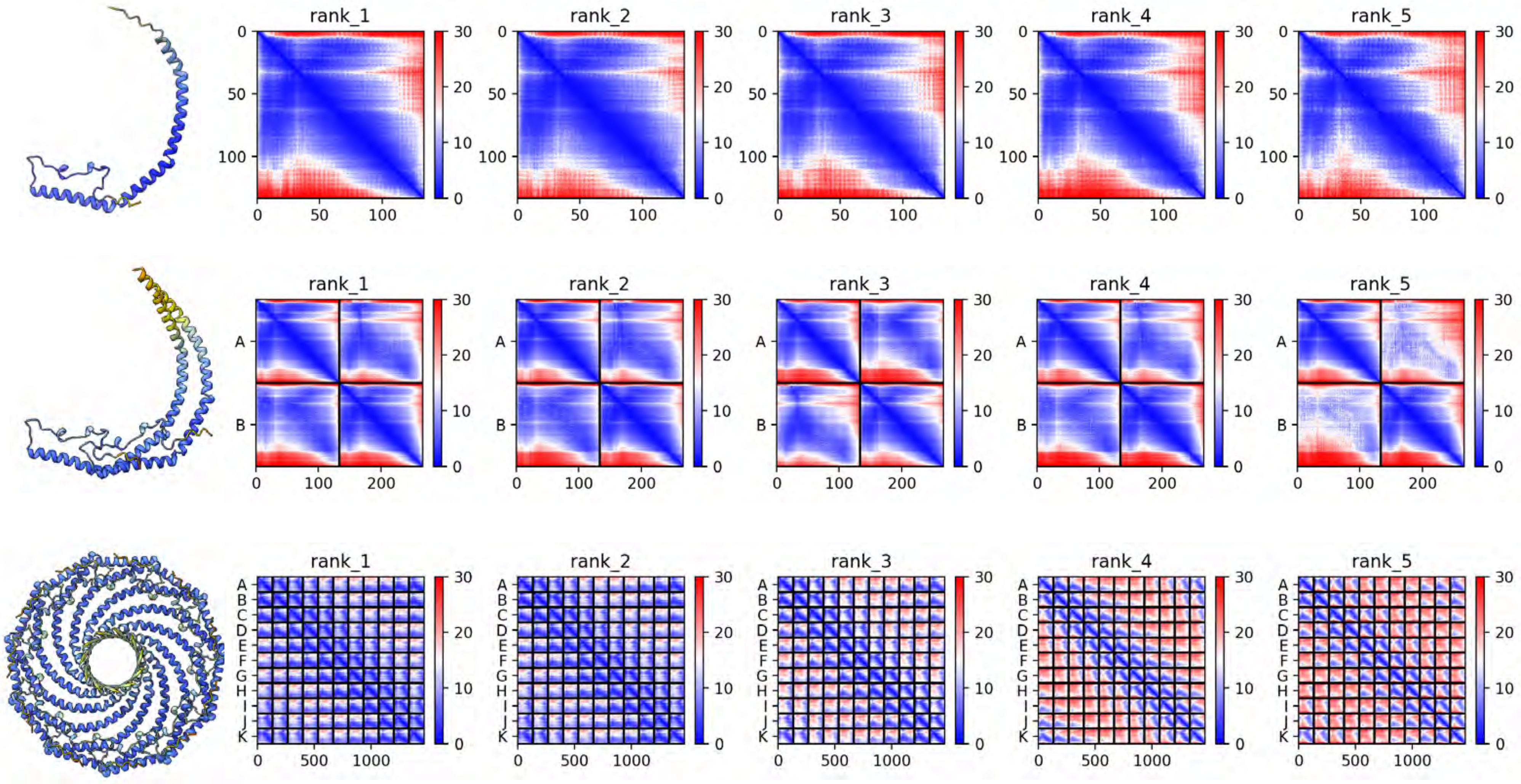

A0A1I8GFP4 *M. lignano*

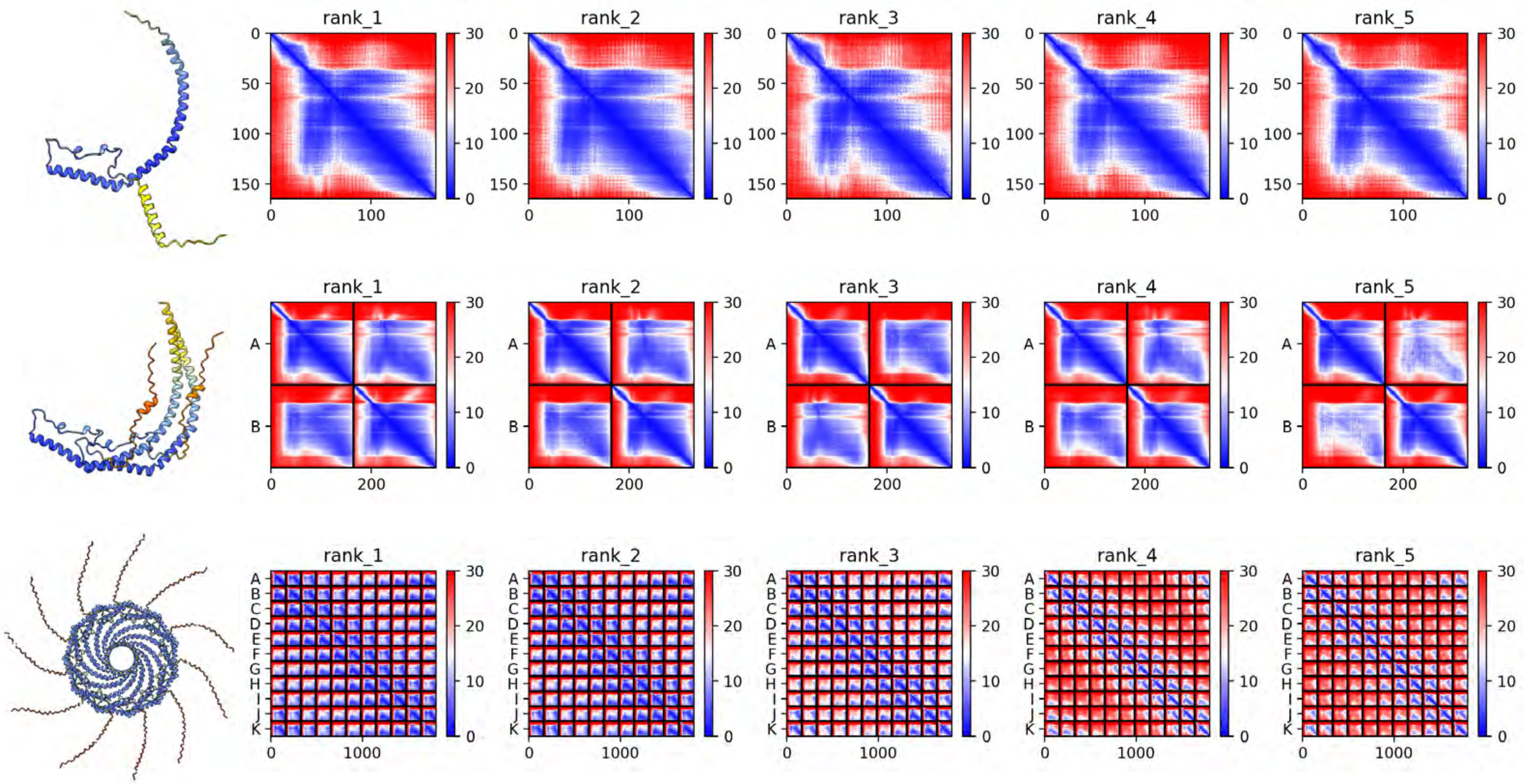

A0A1I8G233 *M. lignano*

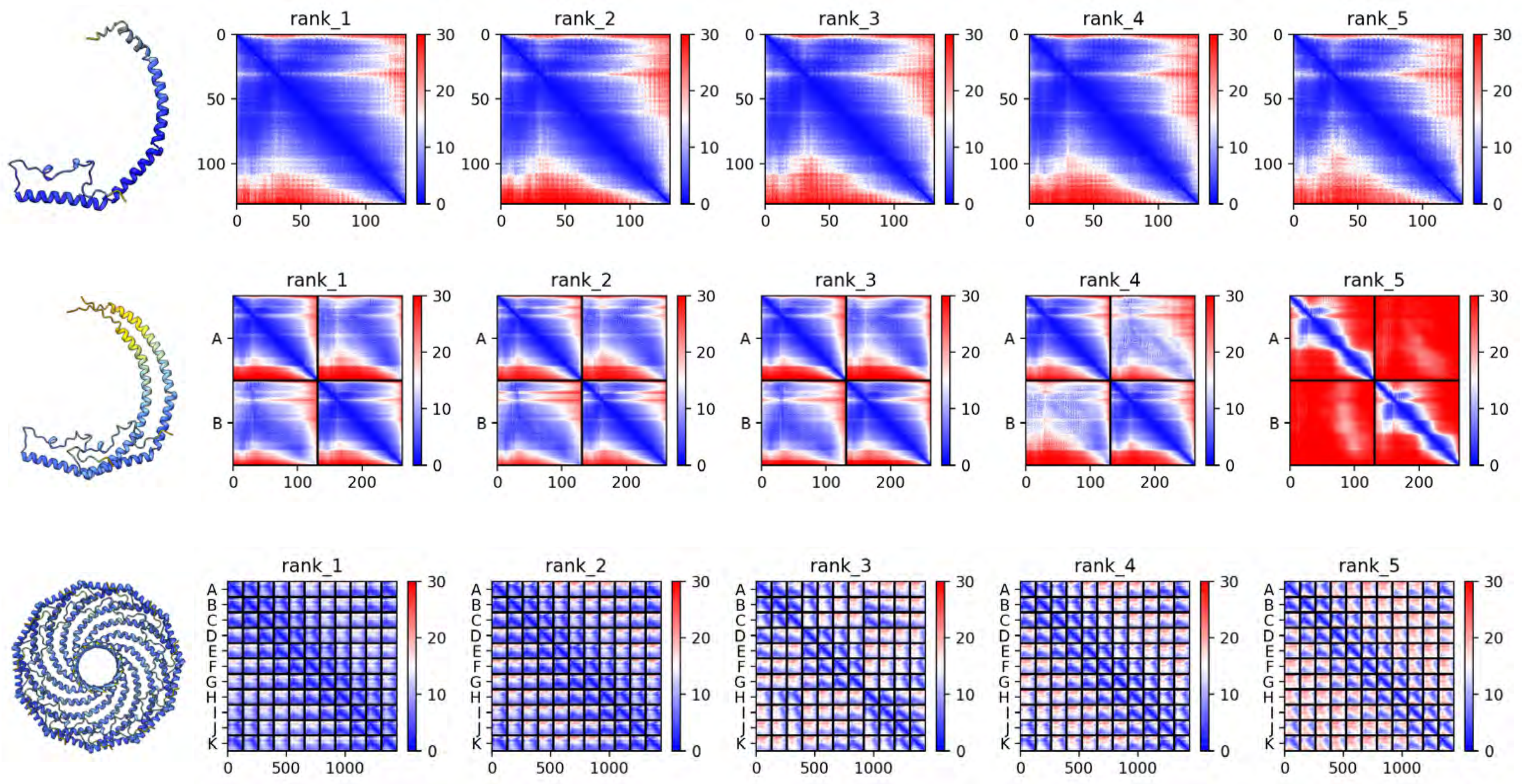

A0A267EVV7 *M. lignano*

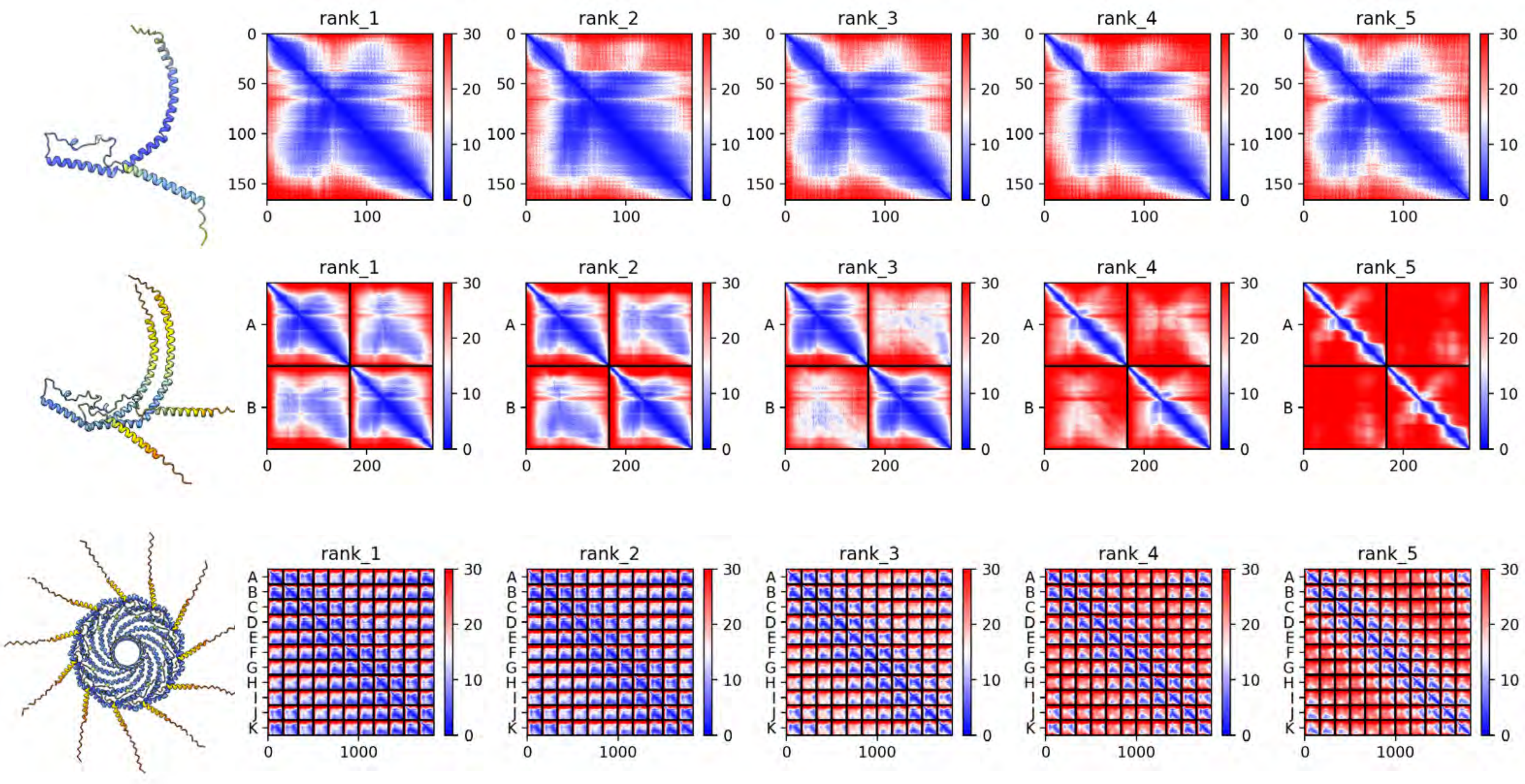

A0A2T7NUT7 *P. canaliculata*

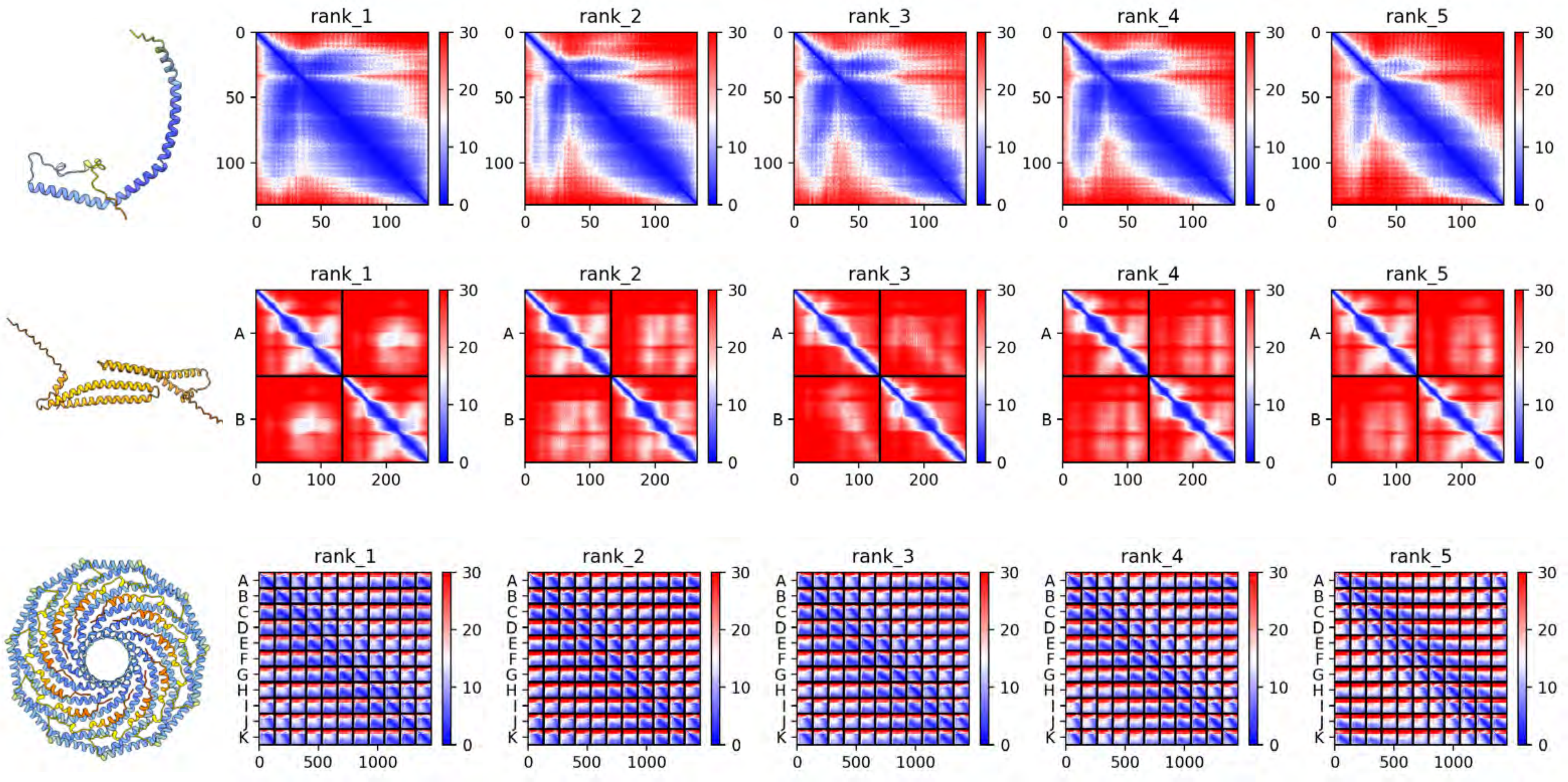

A0A2T7NUS2 *P. canaliculata*

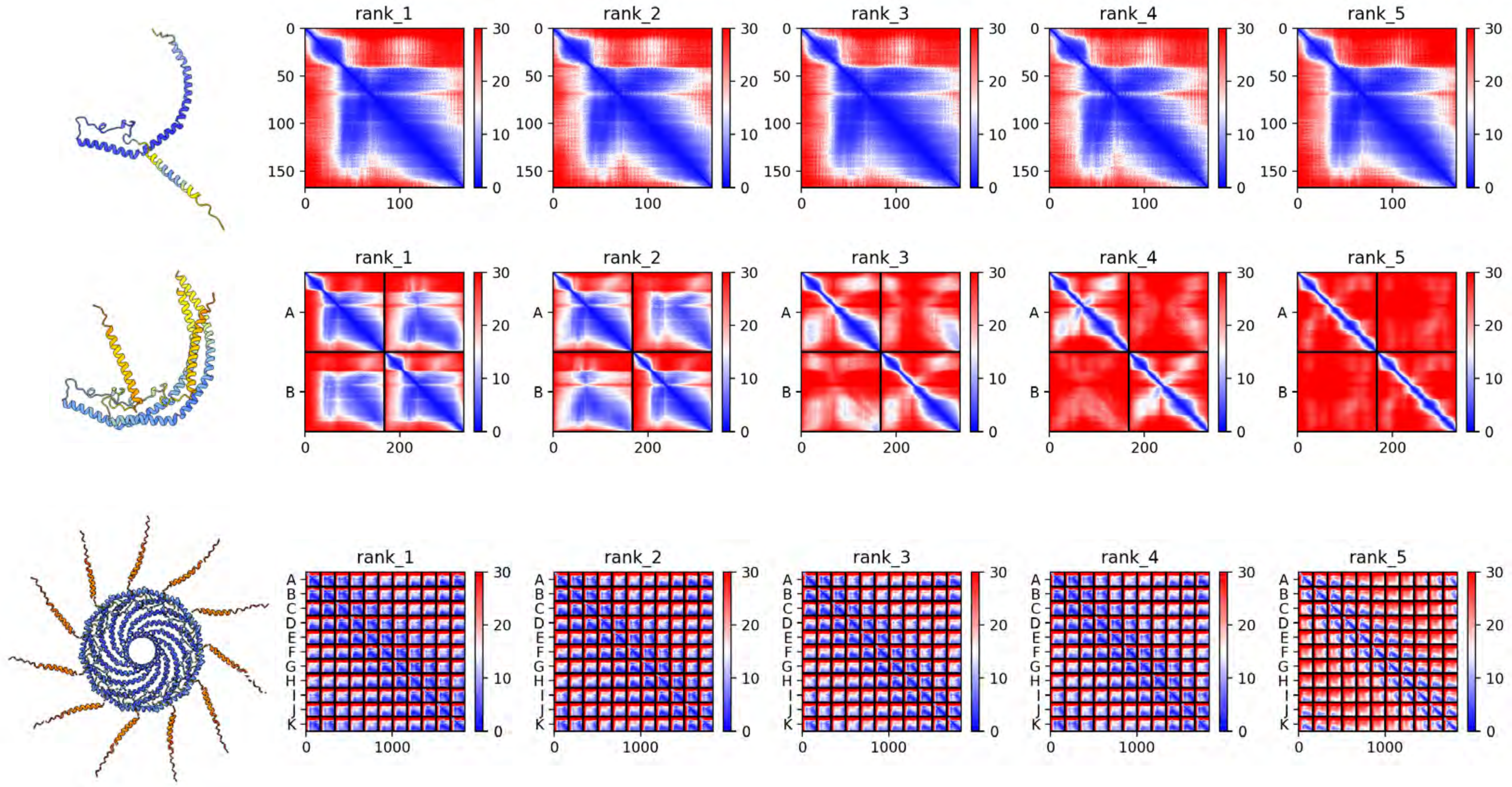

A0A2T7NUV7 *P. canaliculata*

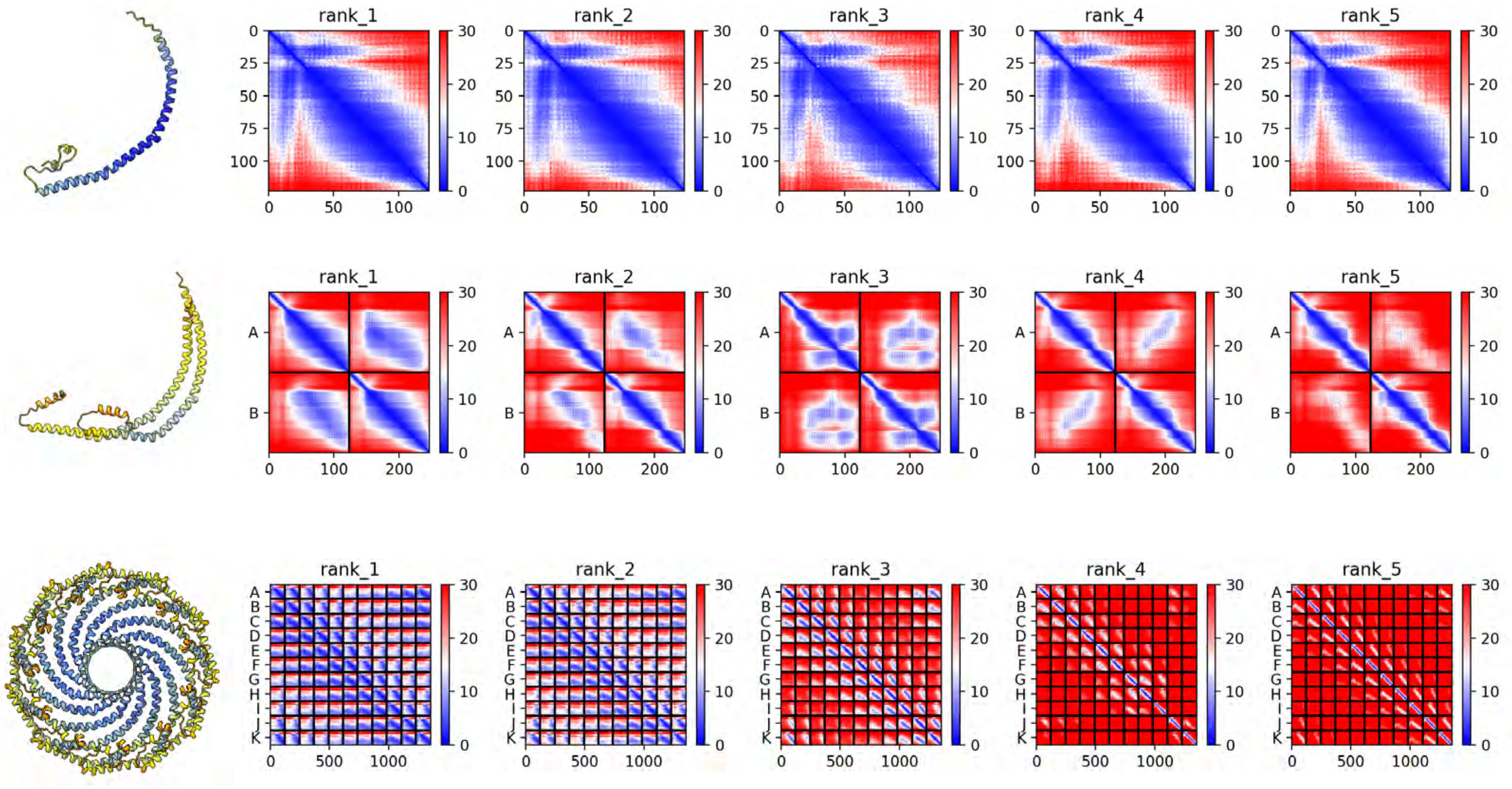

R7VDS7 *C. teleta*

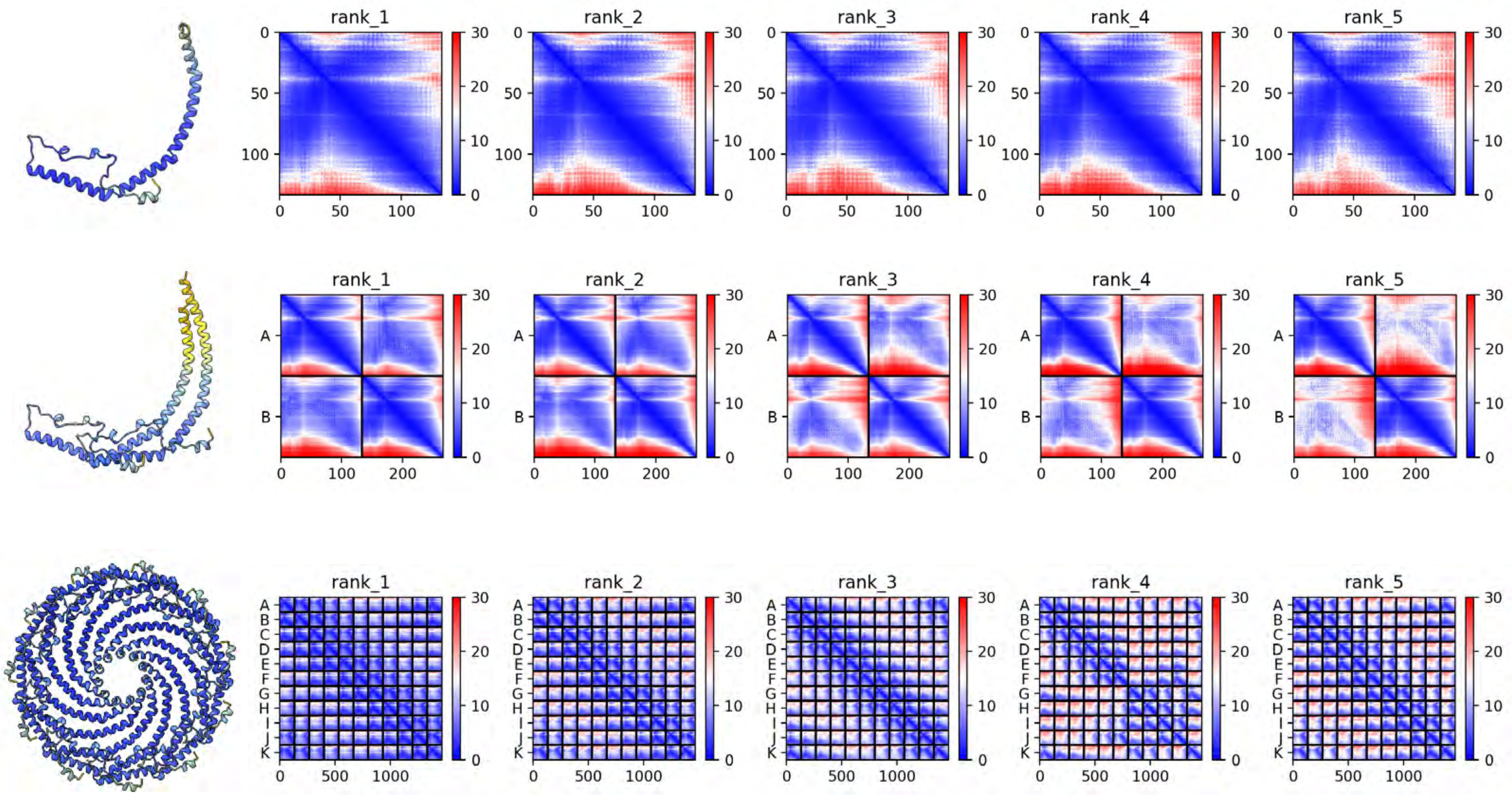

R7V531 *C. teleta*

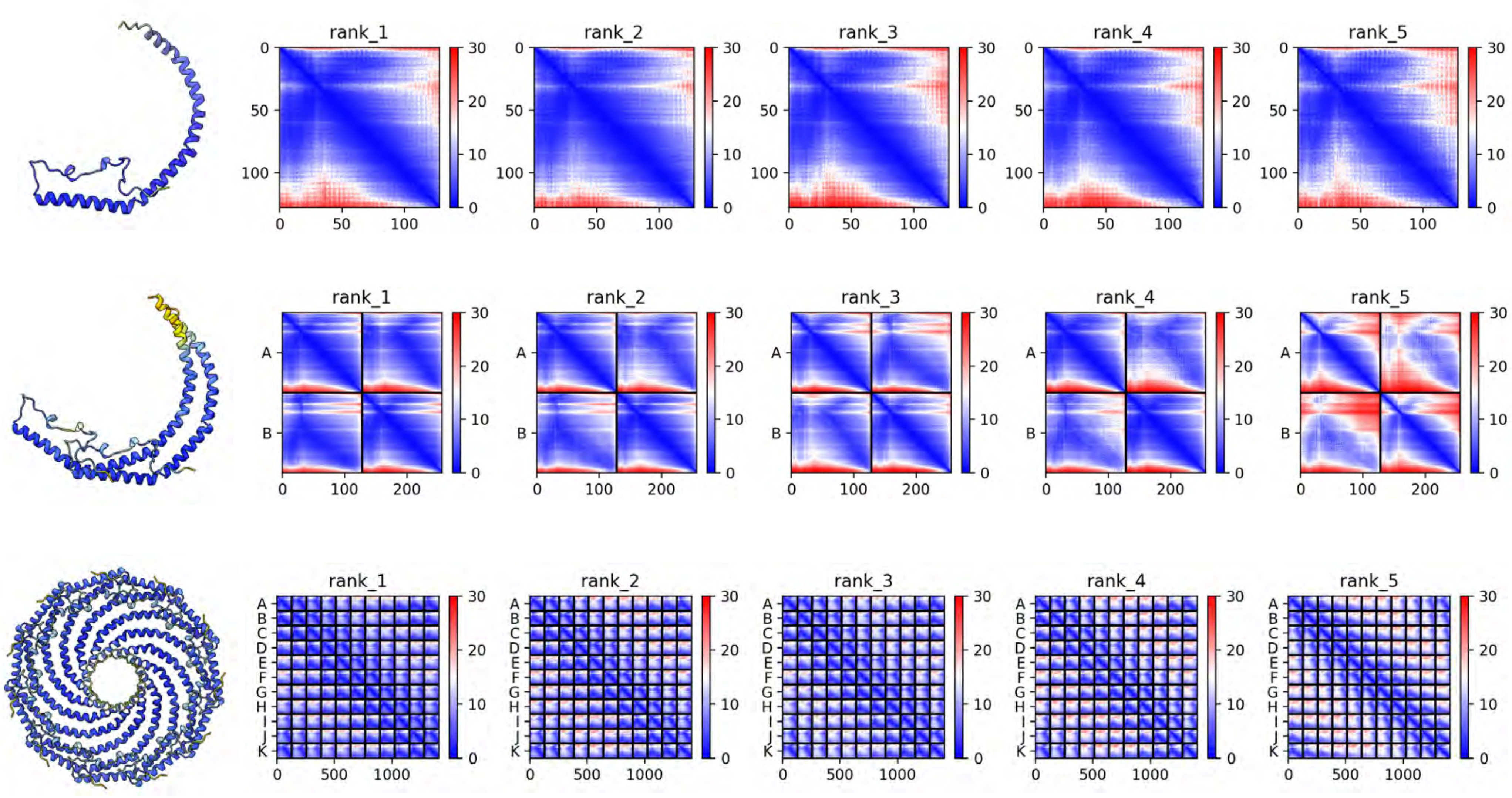

A0A7M7RAC4 *S. purpuratus*

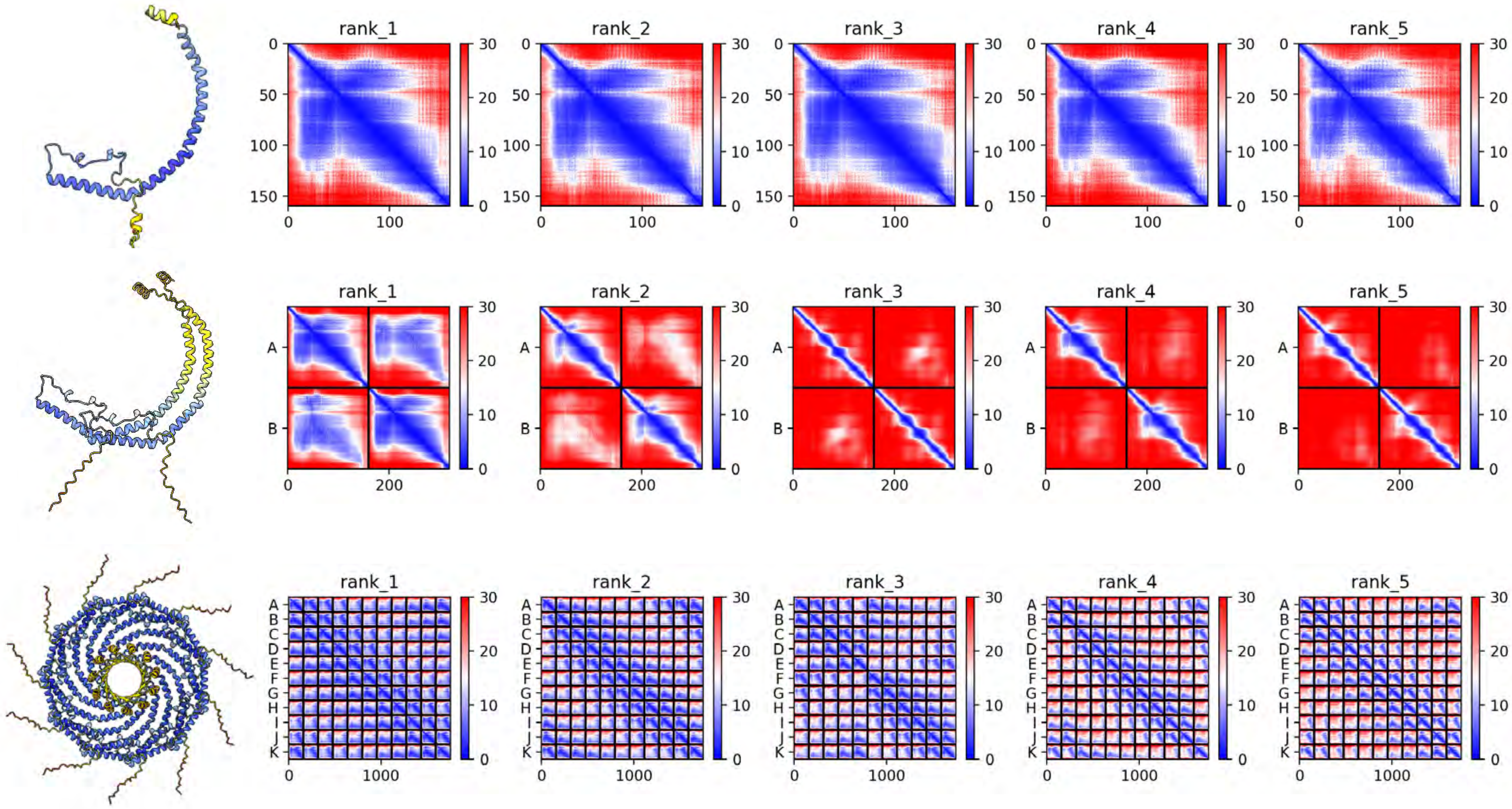

A0A2B4SEV1 *S. pistillata*

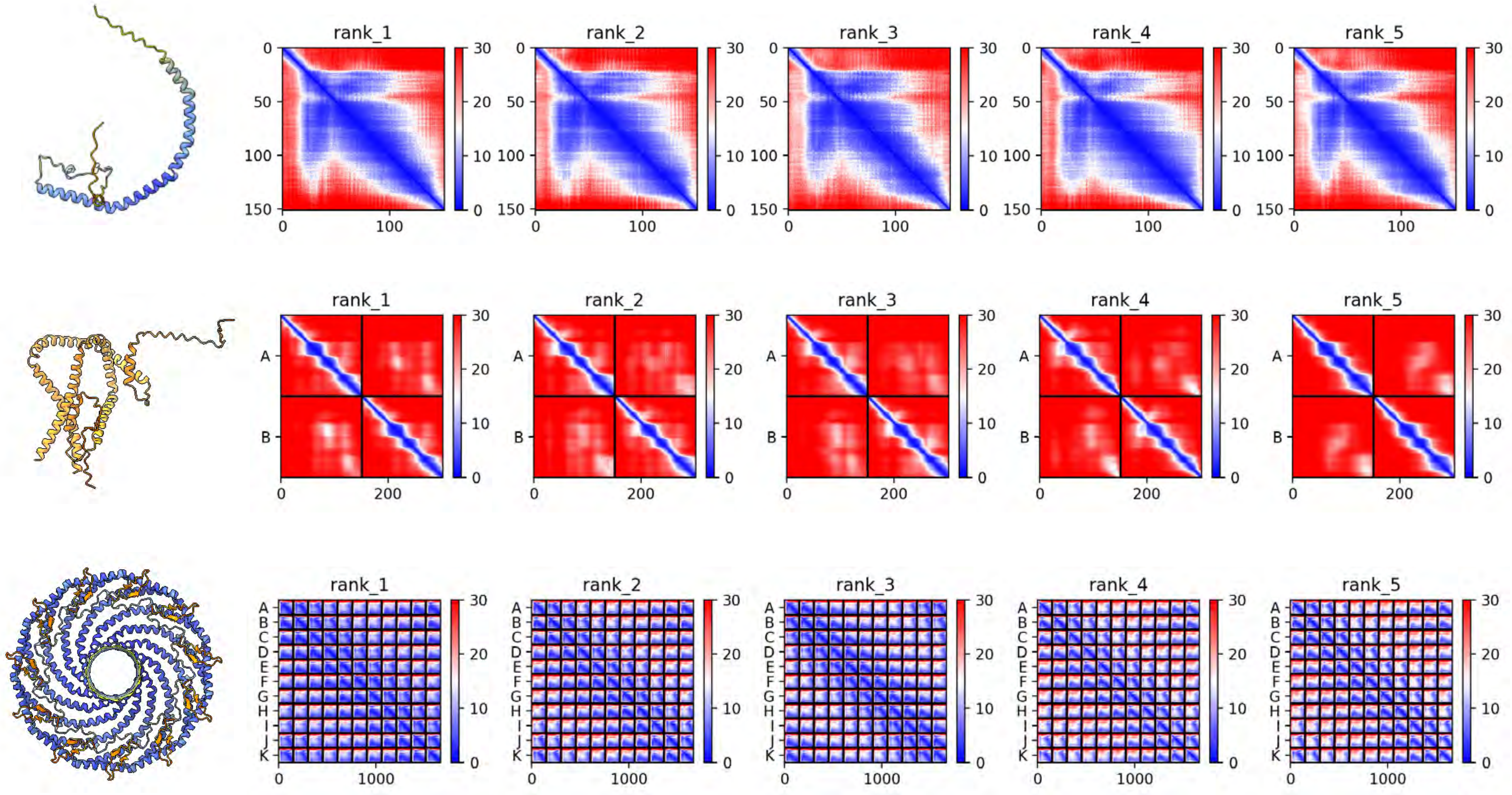

A0A2B4SAZ9 *S. pistillata*

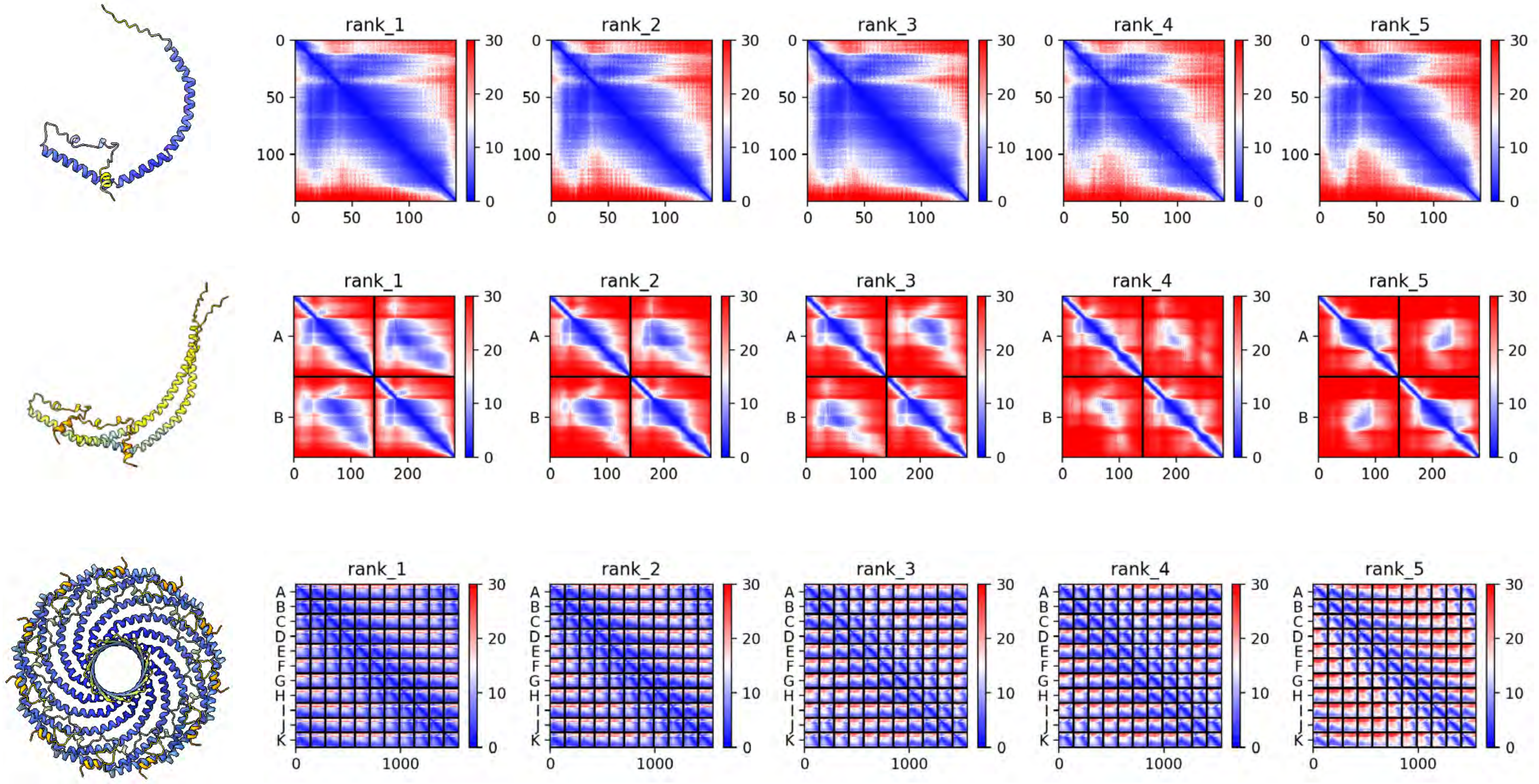

scaffold217_2 *Xenoturbella bocki*

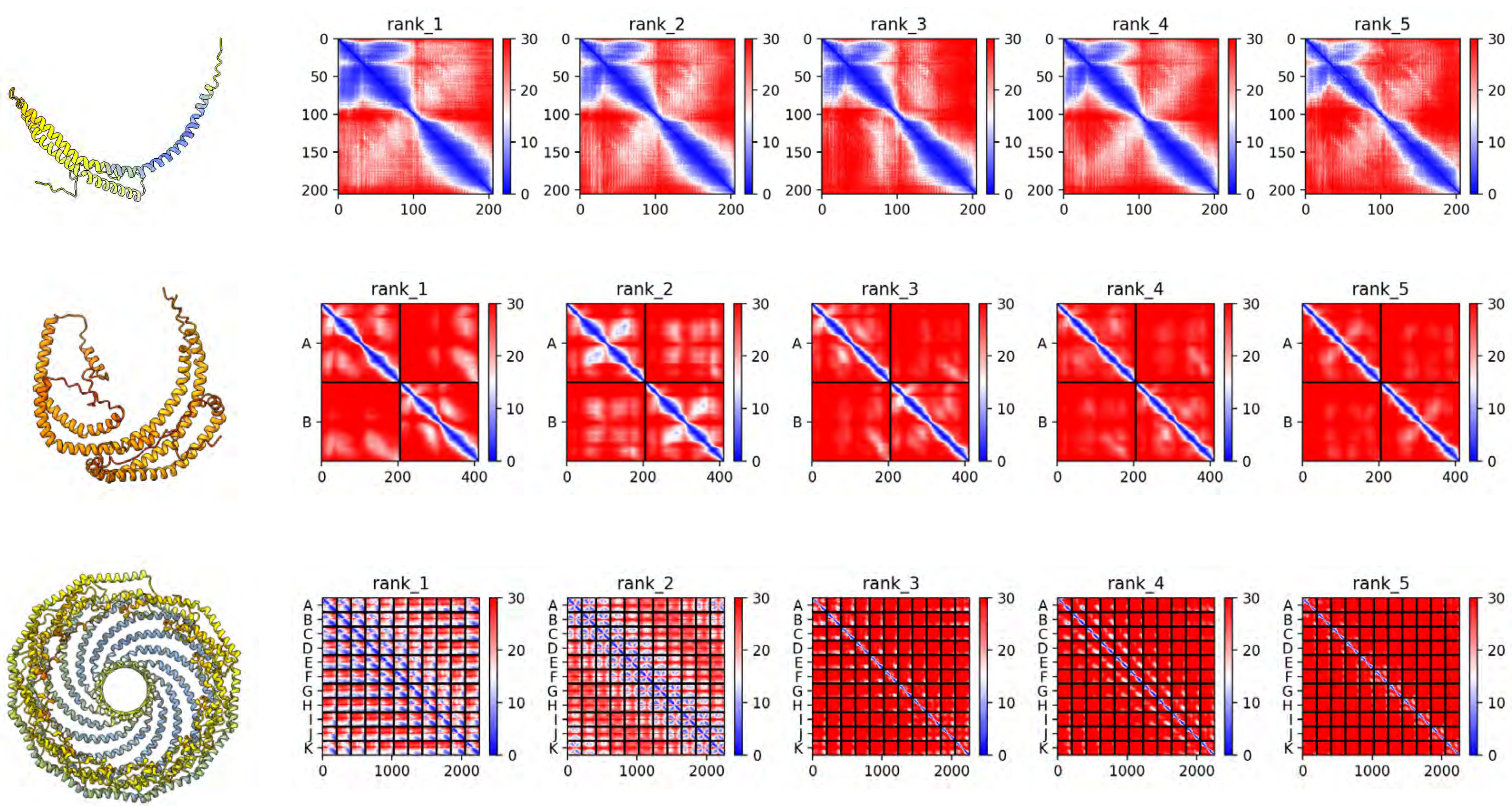

XP_065842155.1 *O. lobularis*

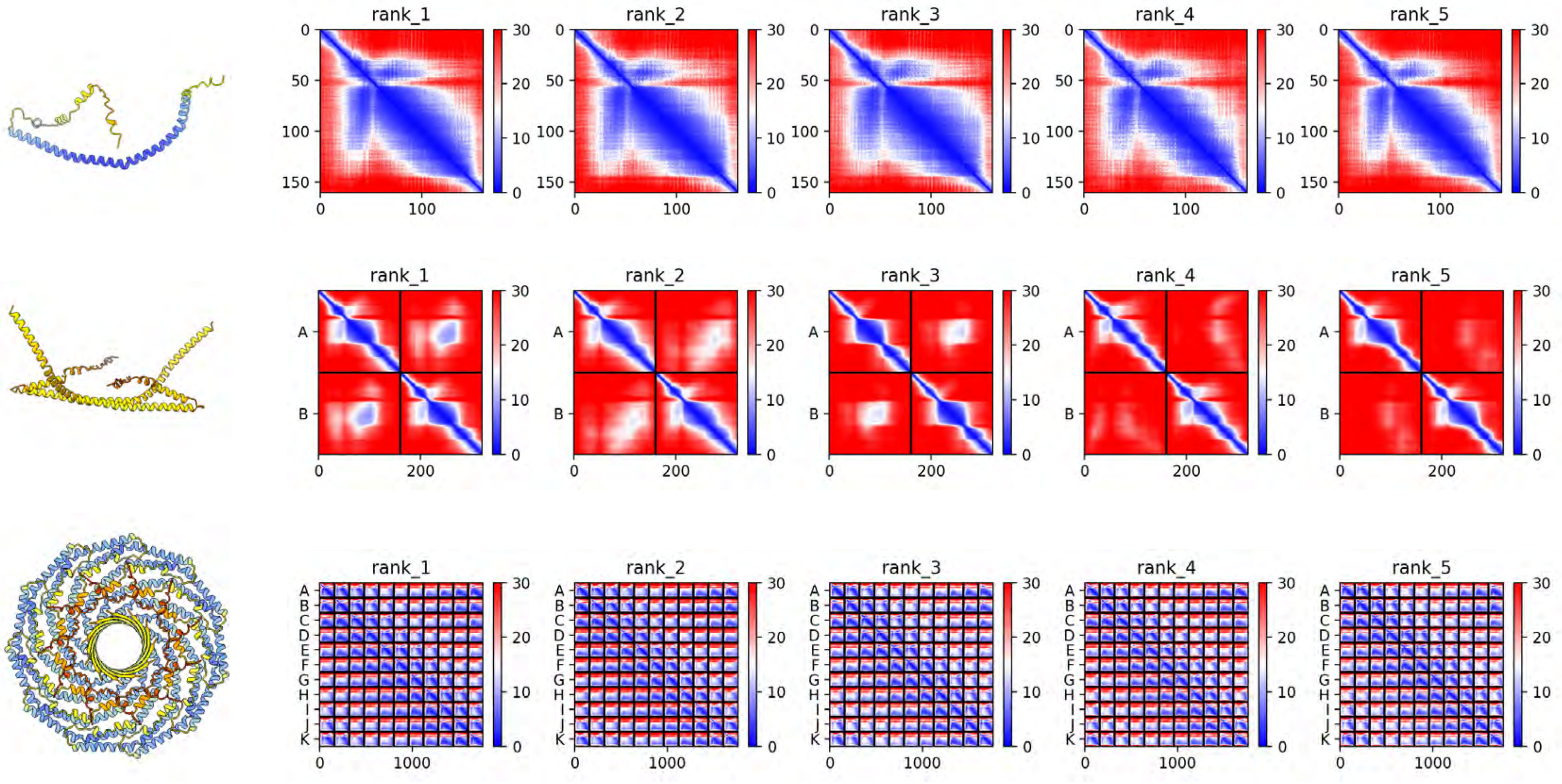

XP_065840741.1 *O. lobularis*

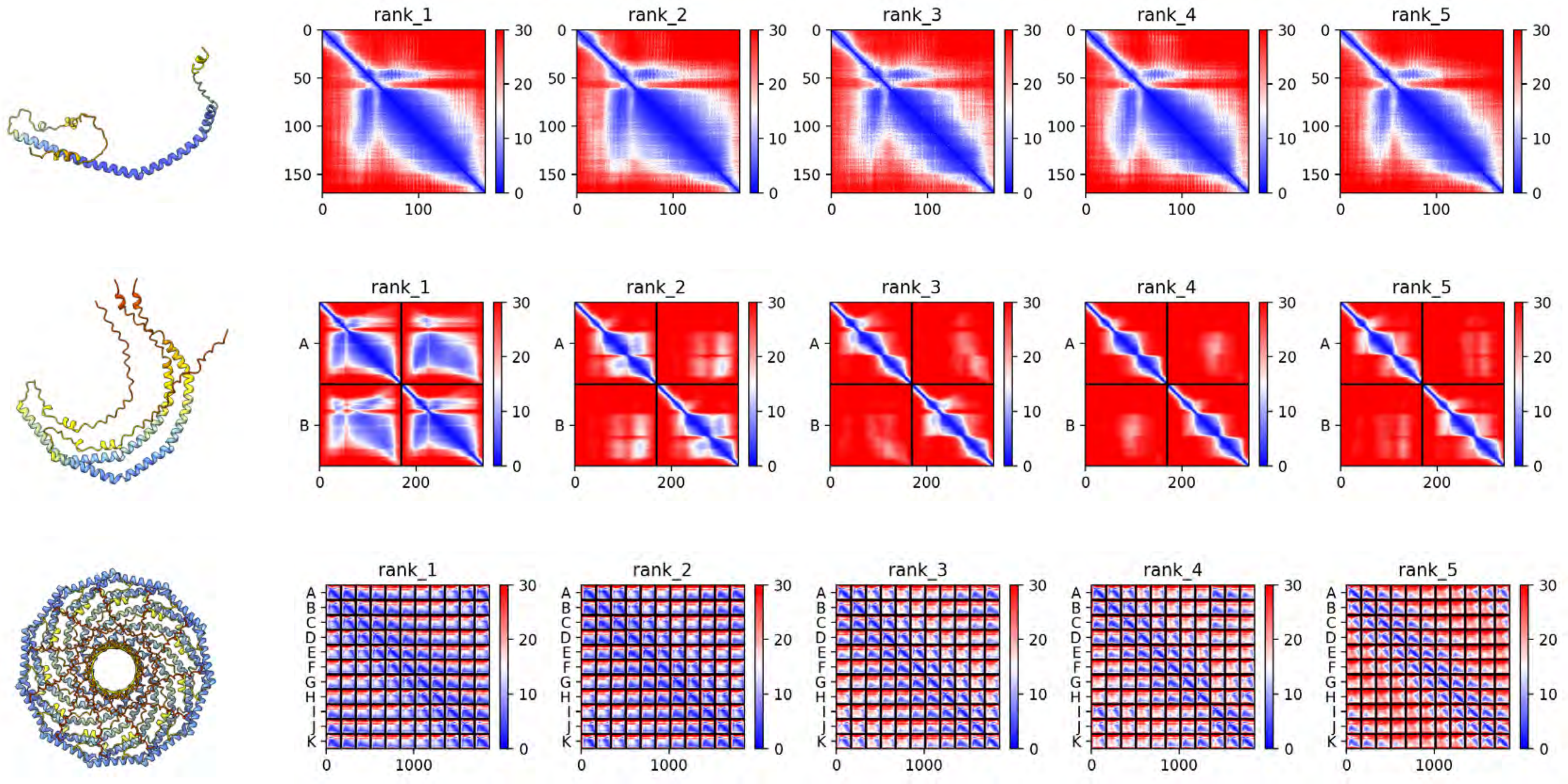

Em0021g891a *E. muelleri*

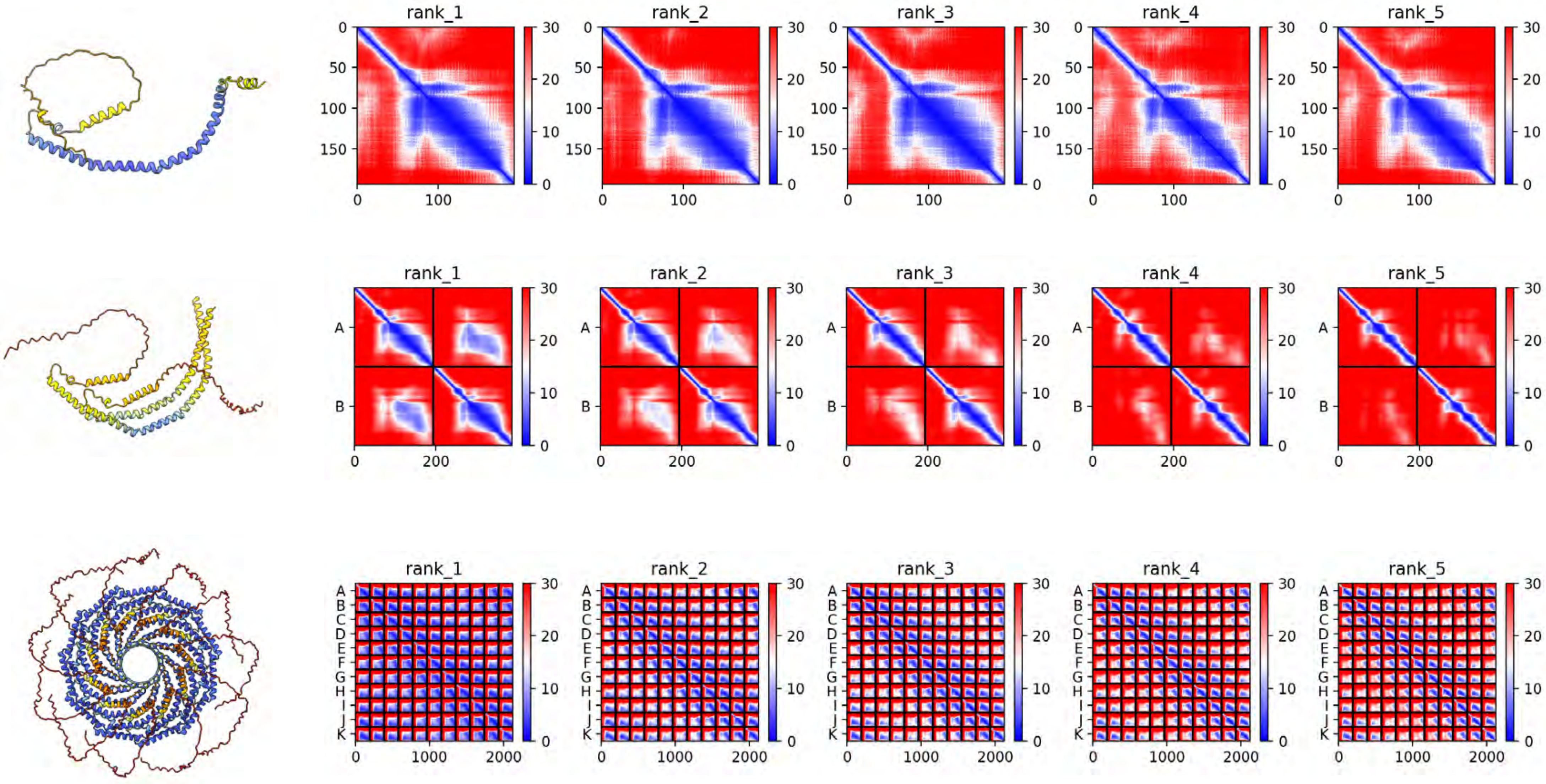

Em0021g893a *E. muelleri*

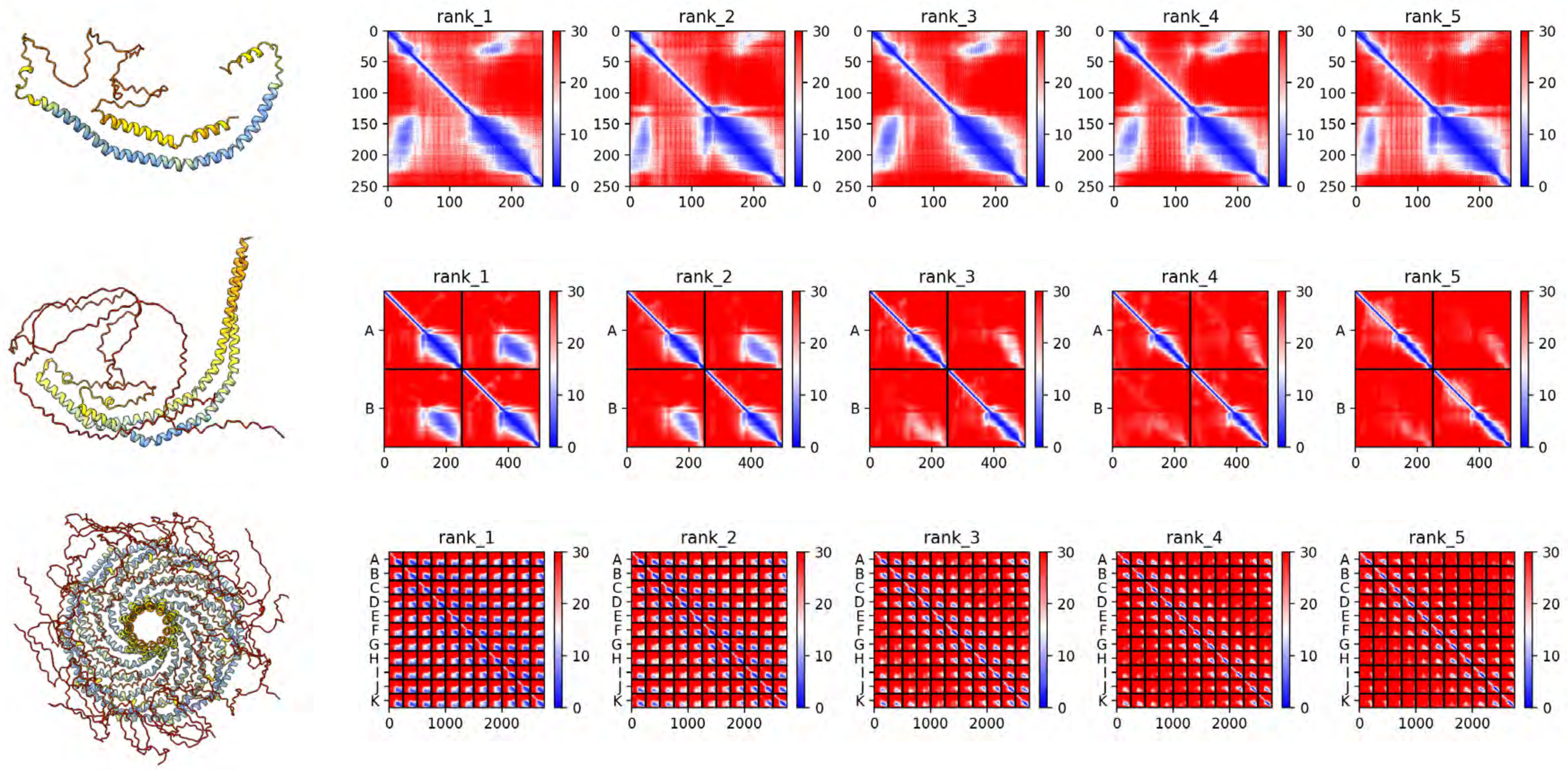

XP_001633849.3 *N. vectensis*

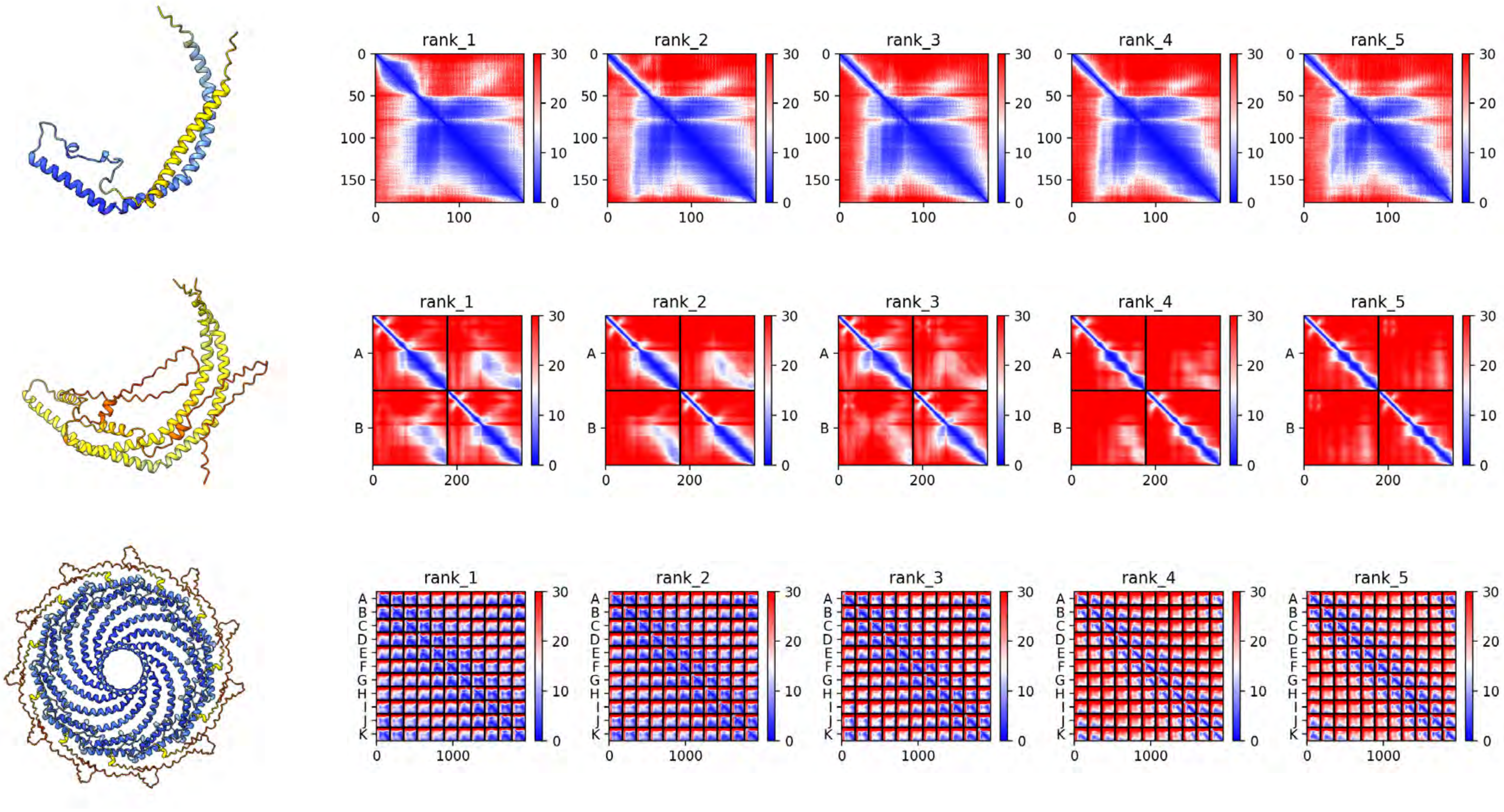

XP_001625439.2 *N. vectensis*

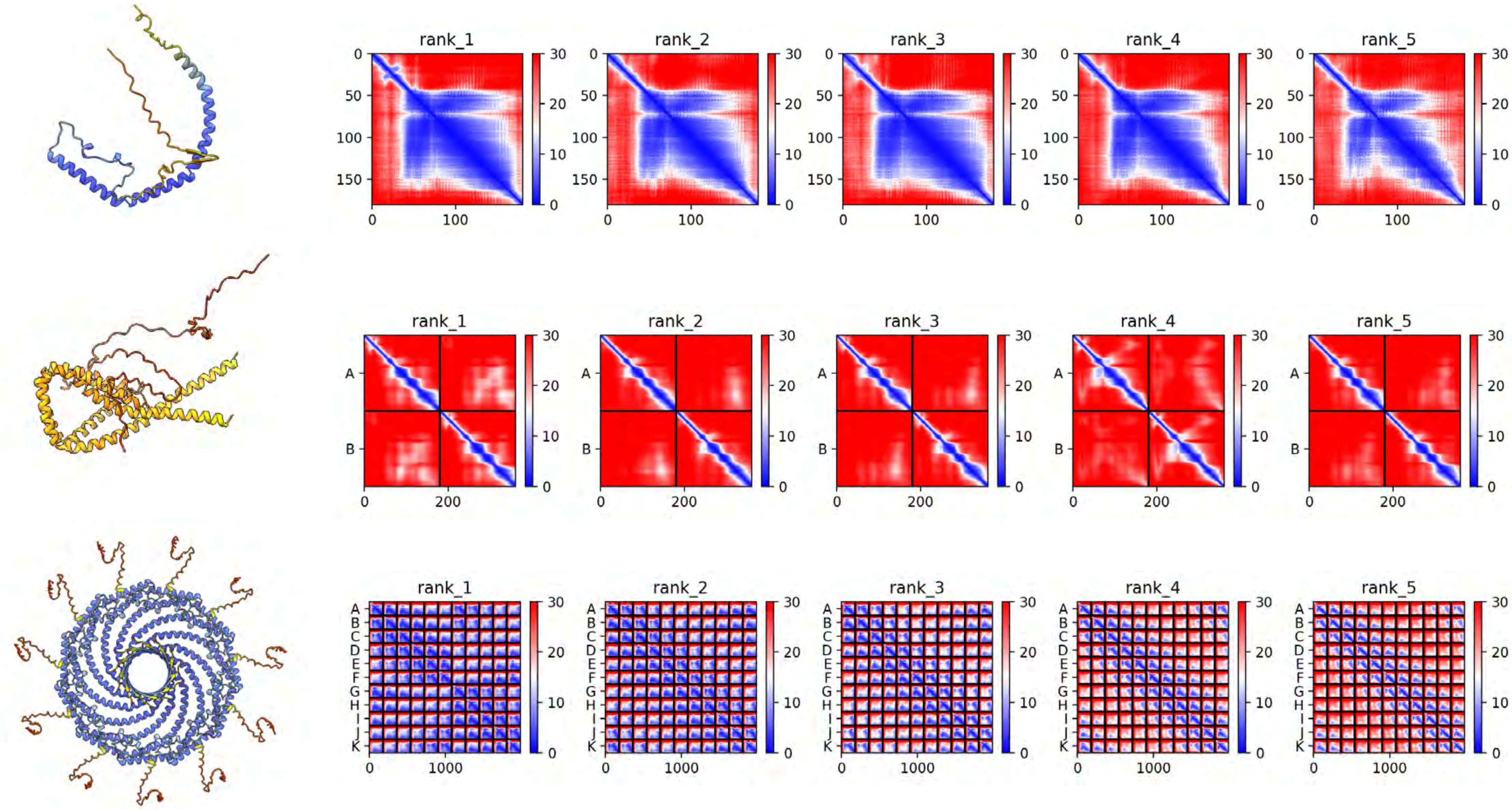

XP_029191350.1 *A. millepora*

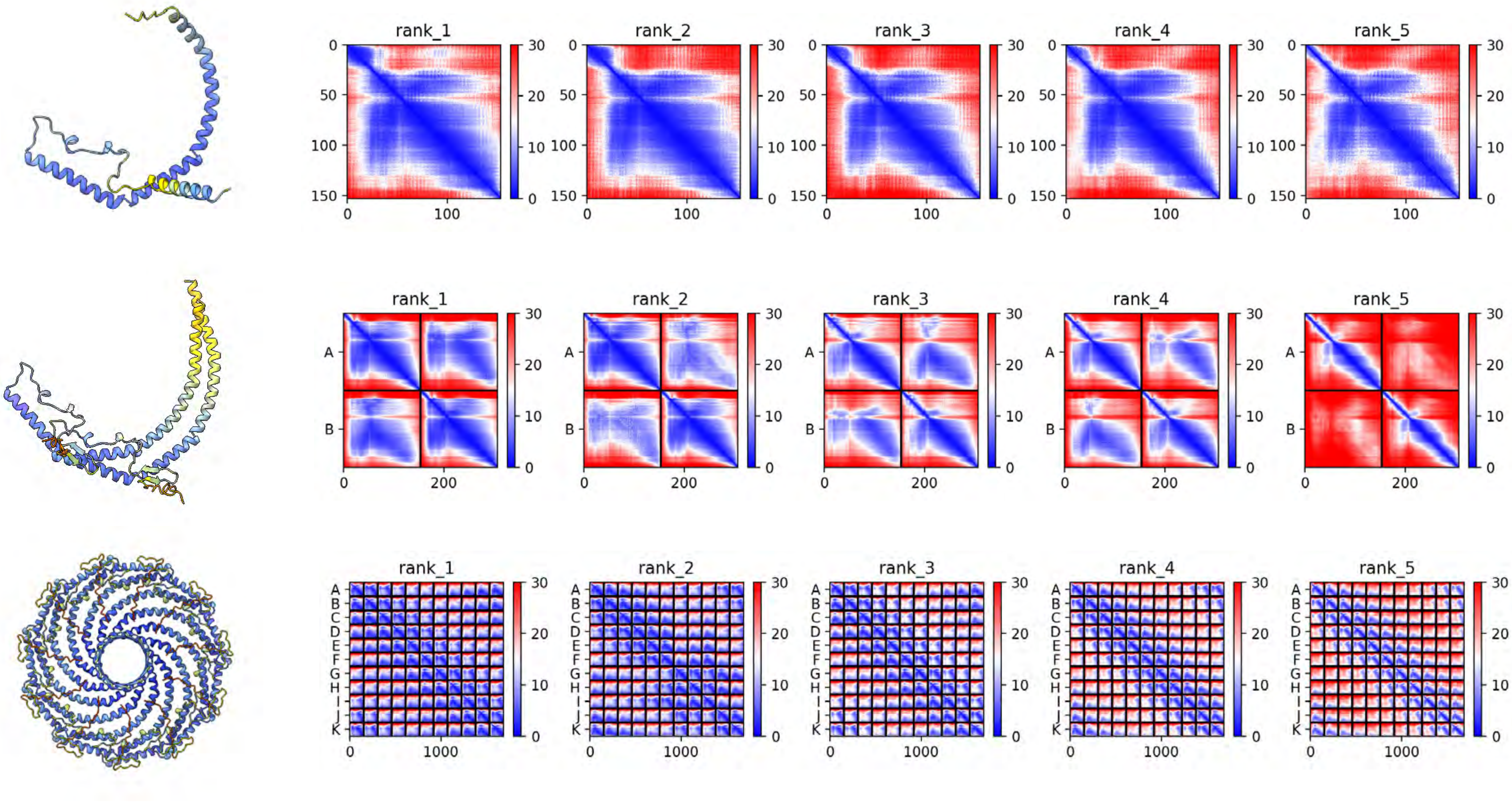

XP_033745936.1 *P. maximus*

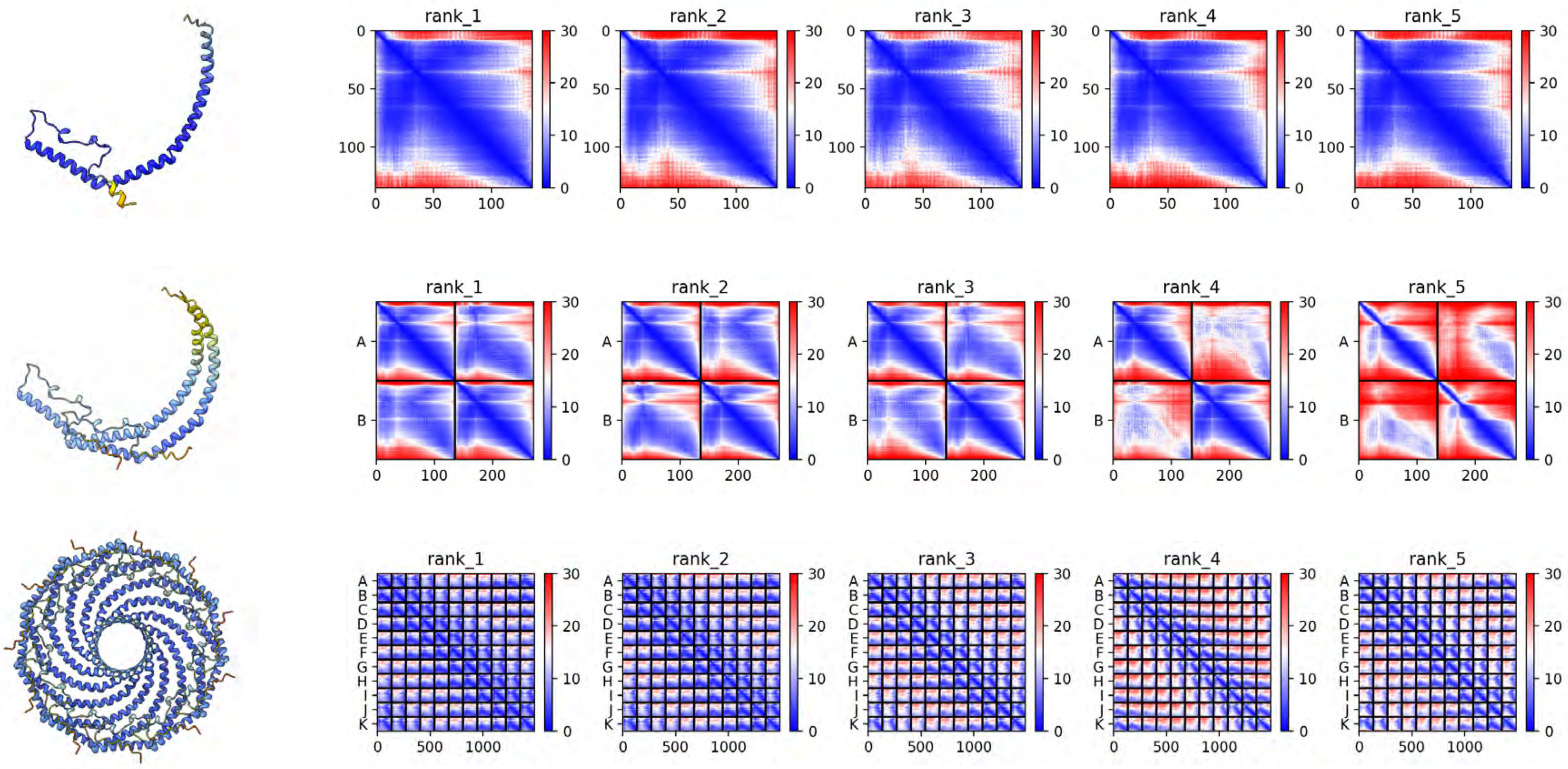

XP_033740634.1 *P. maximus*

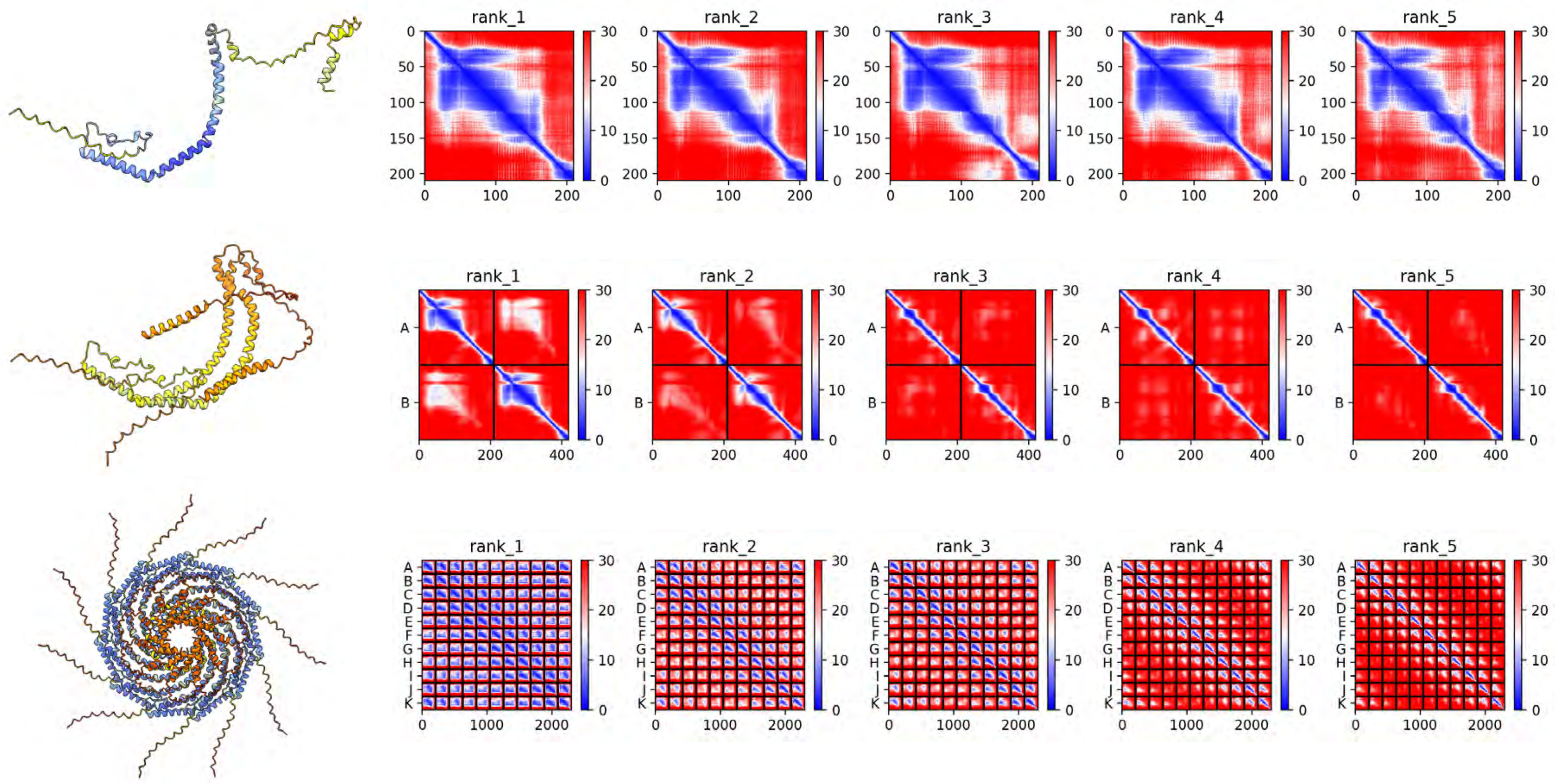

XP_052250592.1 *D. polymorpha*

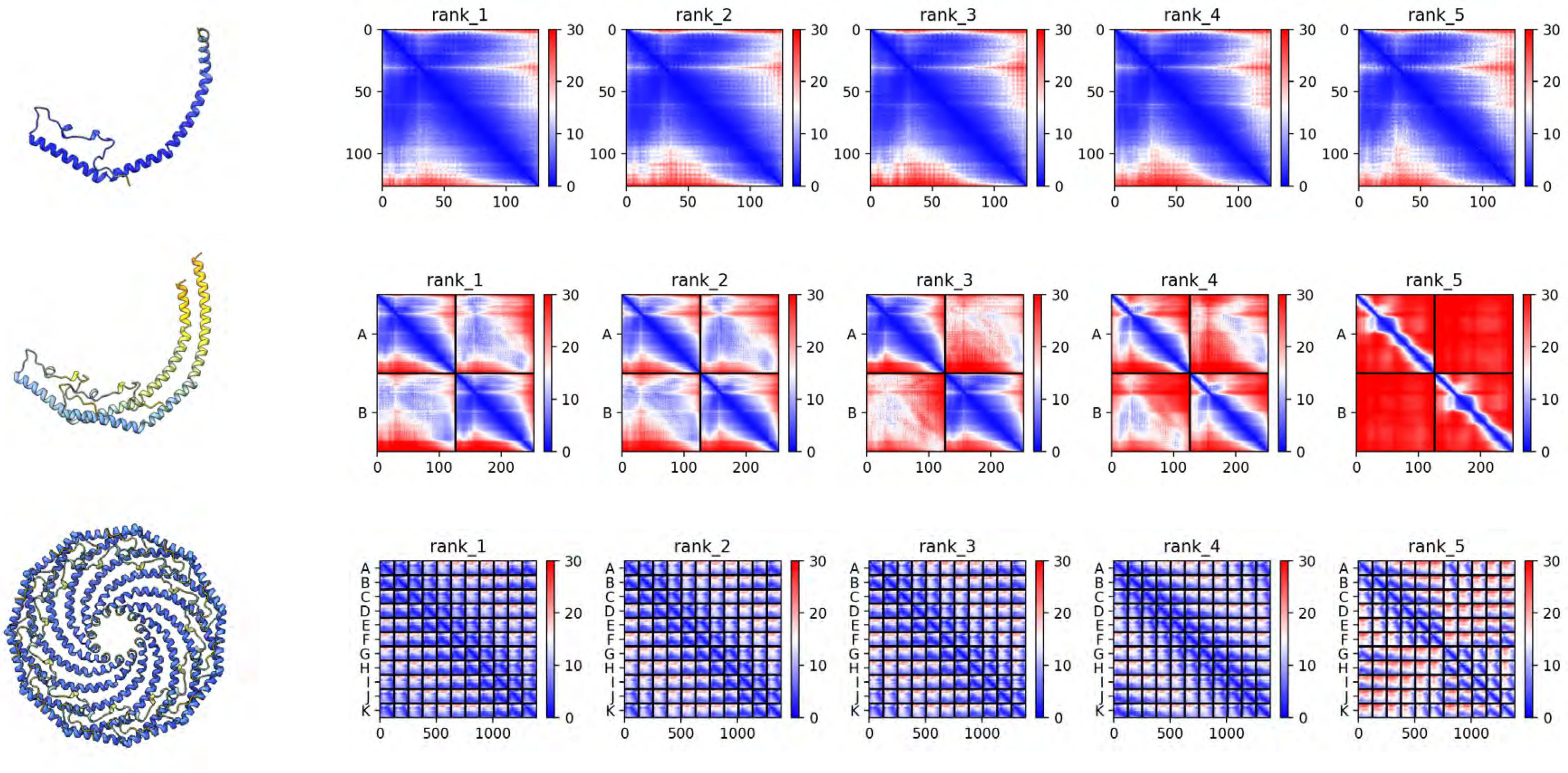

XP_052249606.1 *D. polymorpha*

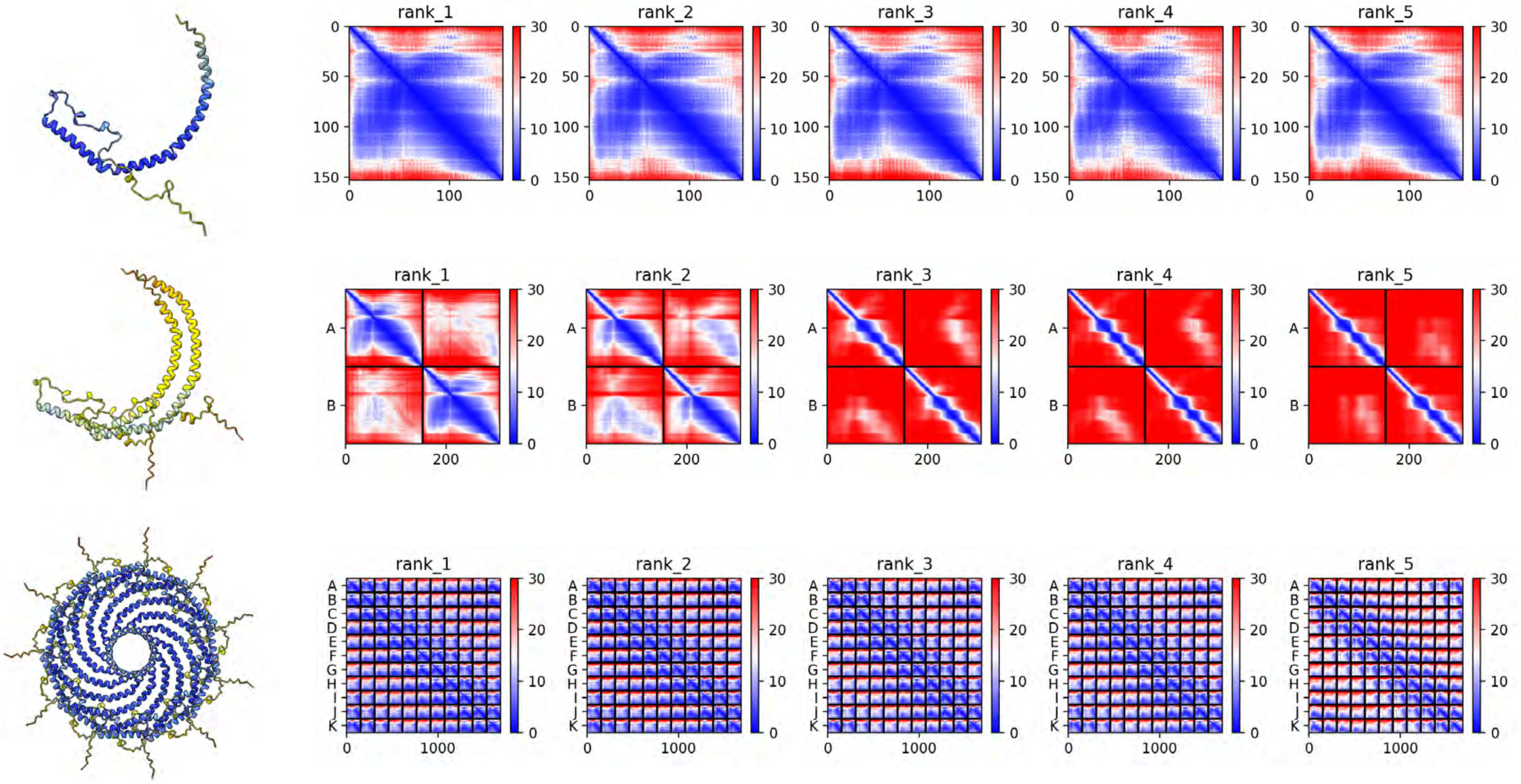

XP_052234244.1 *D. polymorpha*

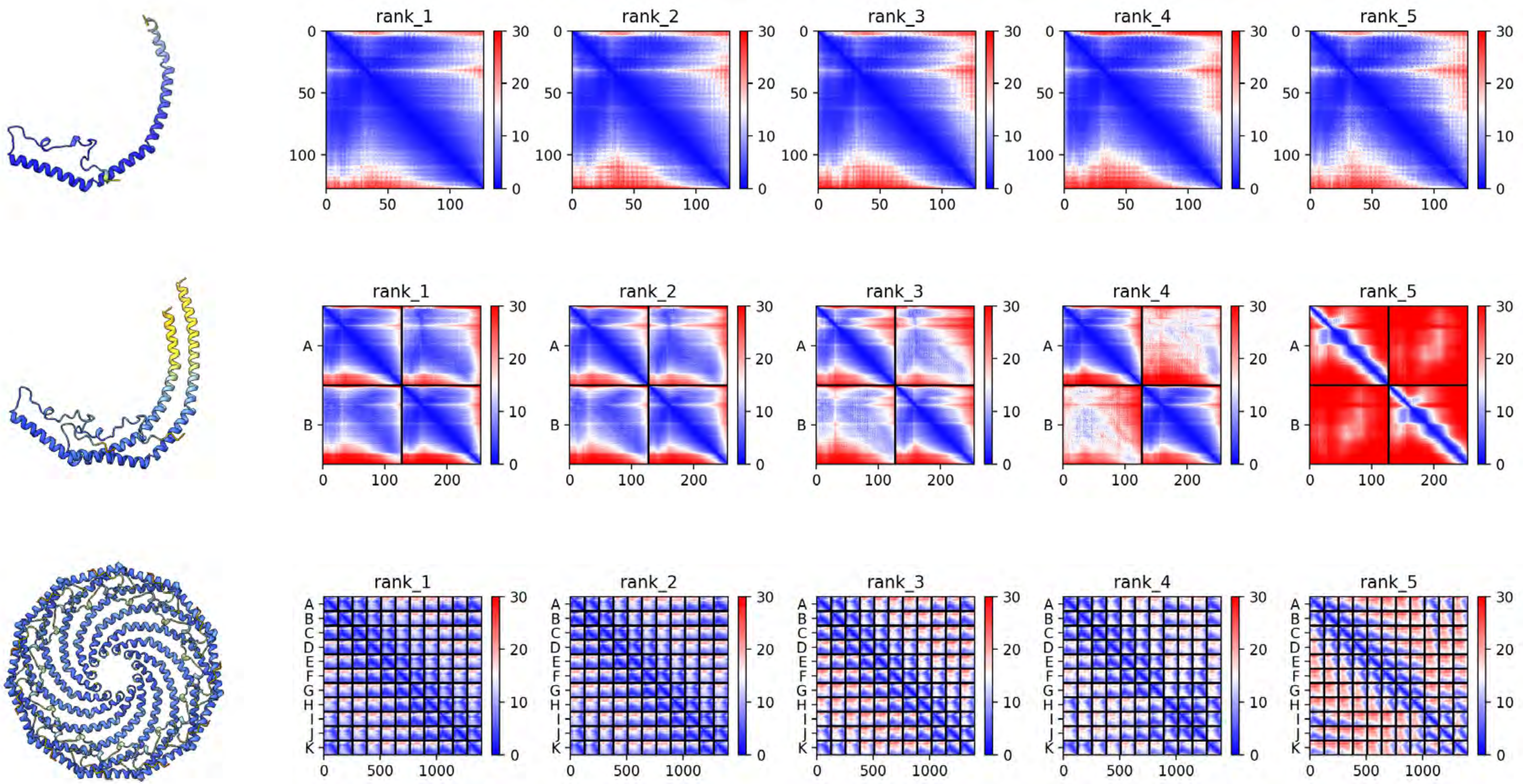

XP_013092506.1 *B. glabrata*

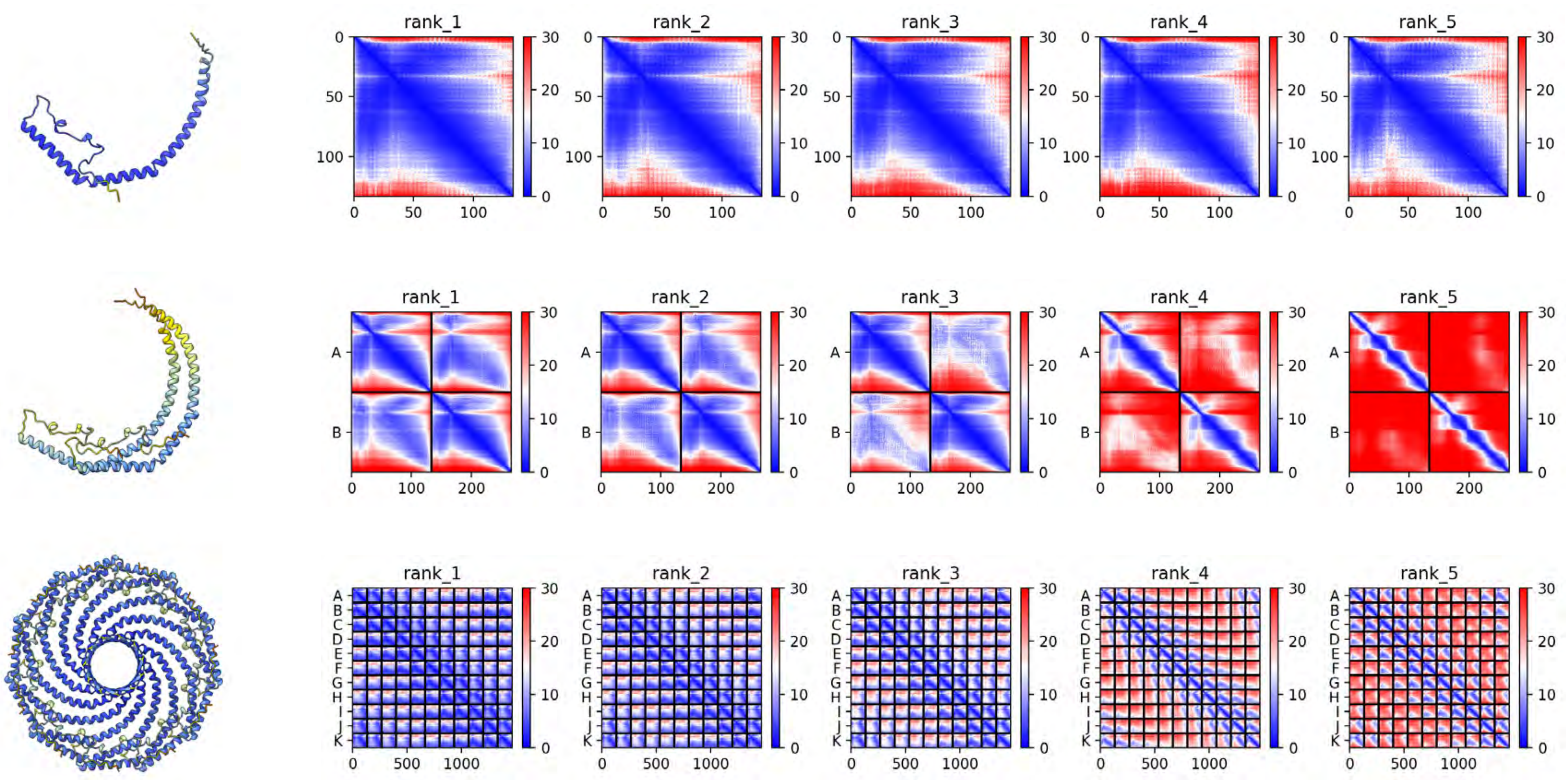

XP_050413640.1 *P. vulgata*

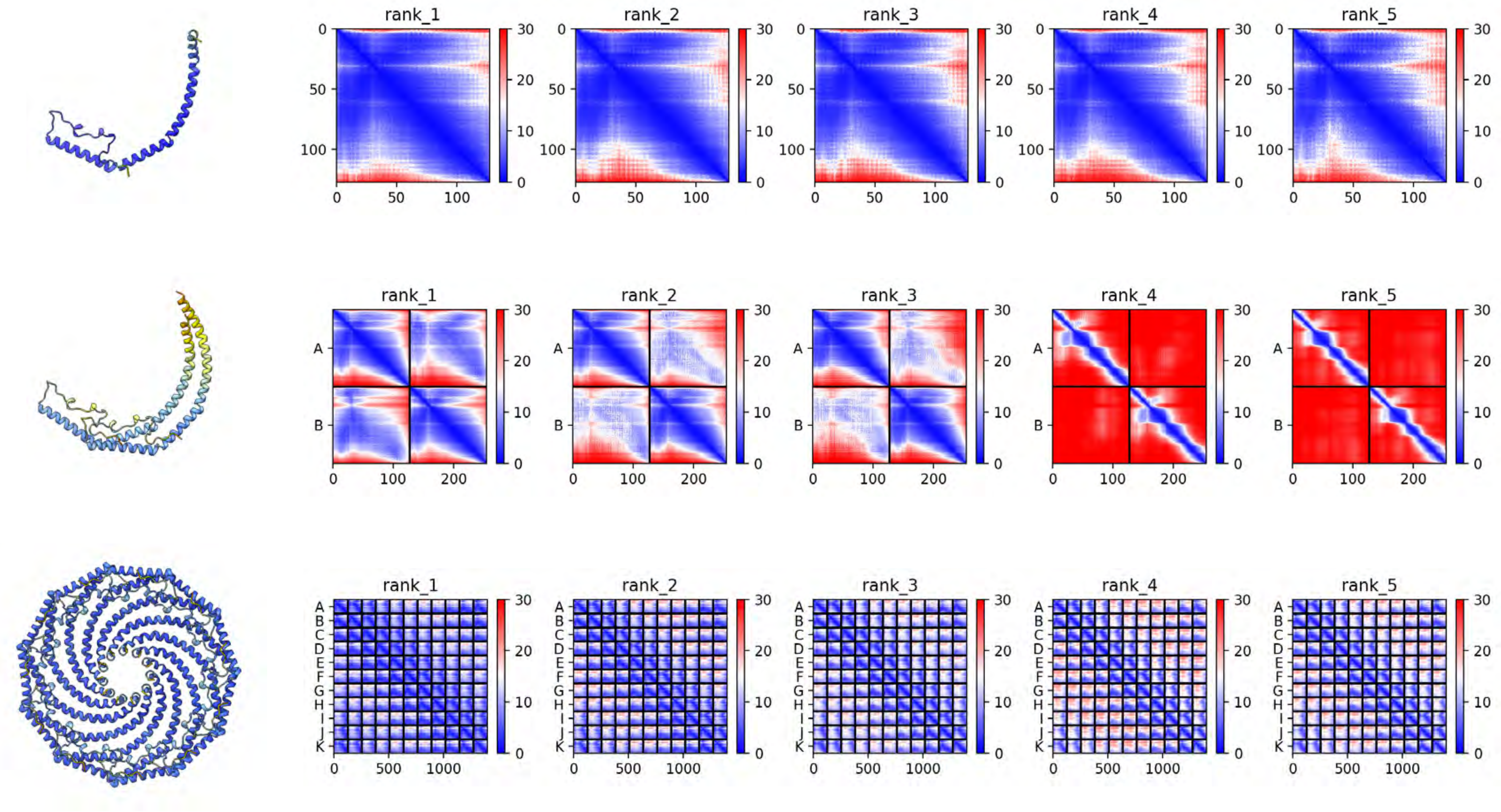

XP_055958200.1 *P. vulgata*

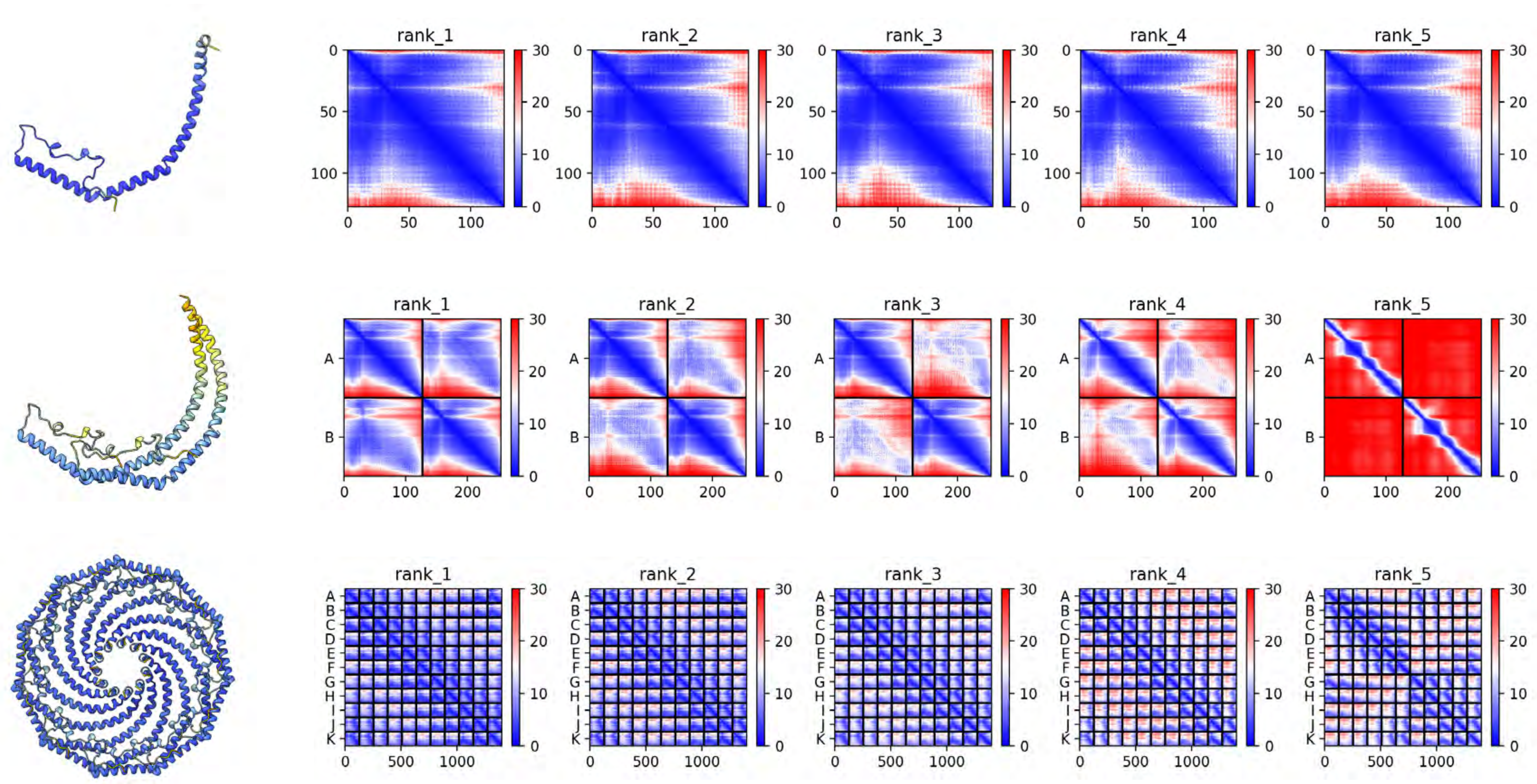

XP_050414932.1 *P. vulgata*

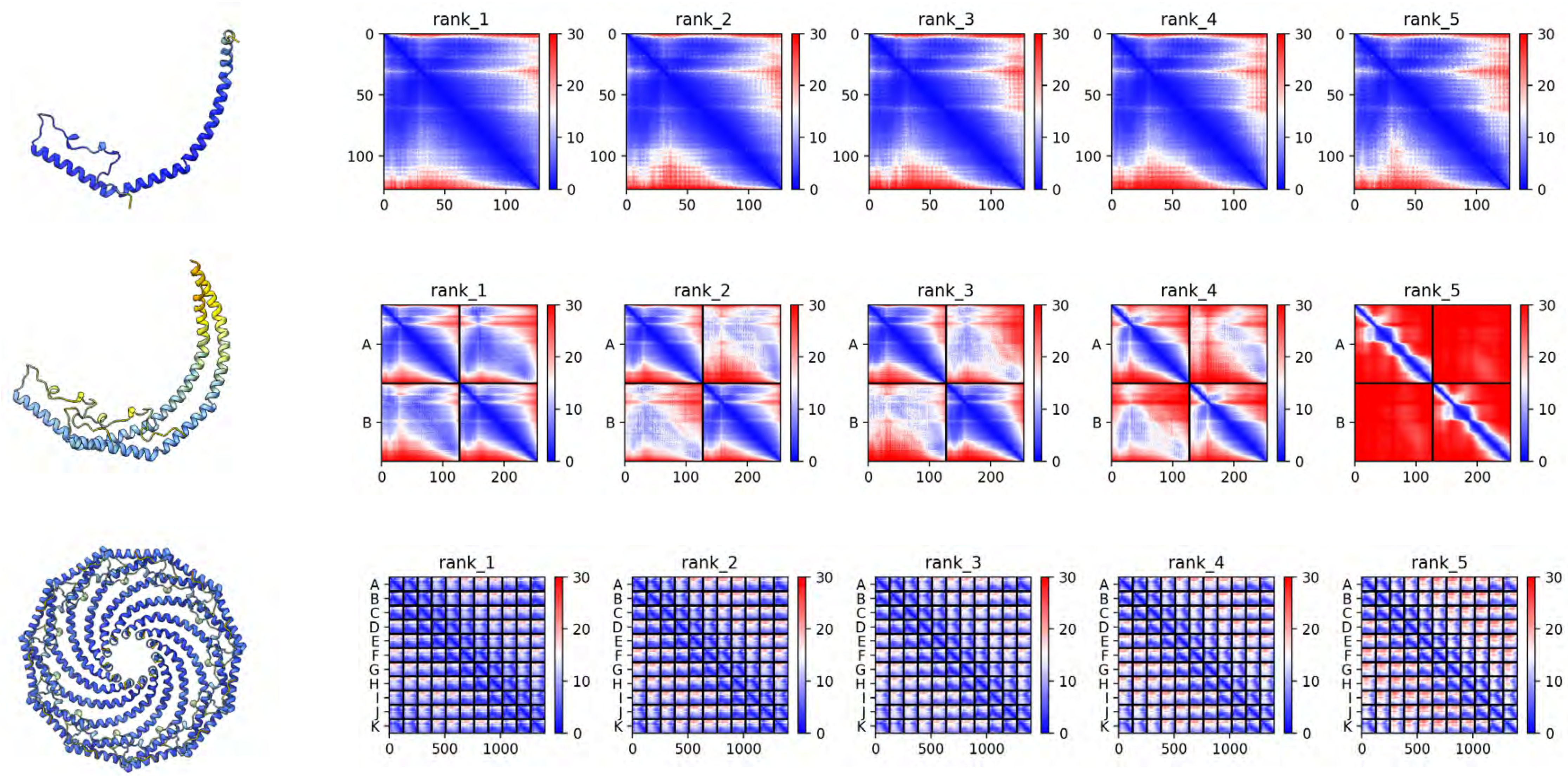

XP_050414695.1 *P. vulgata*

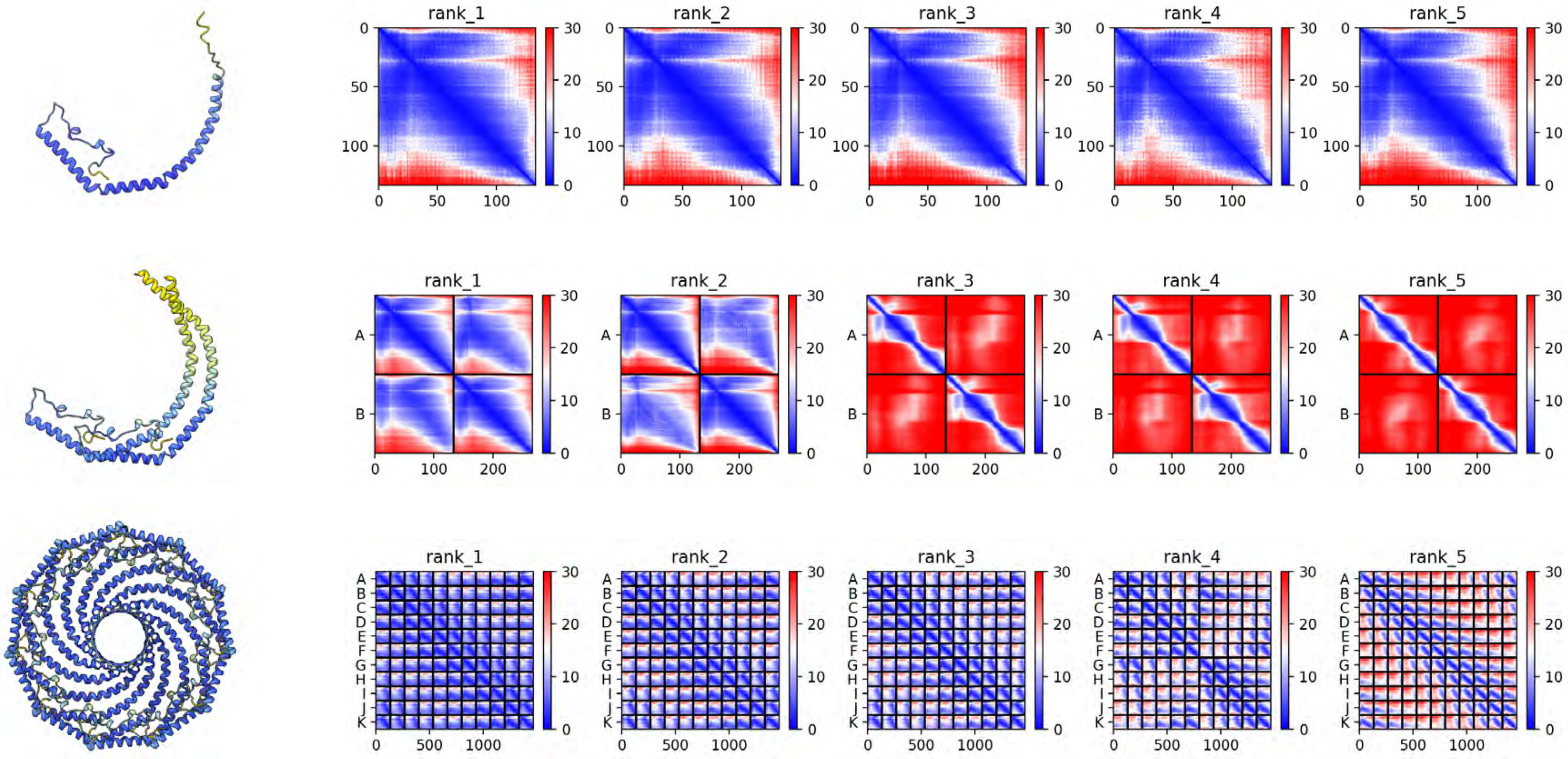

XP_052778958.1 *M. arenaria*

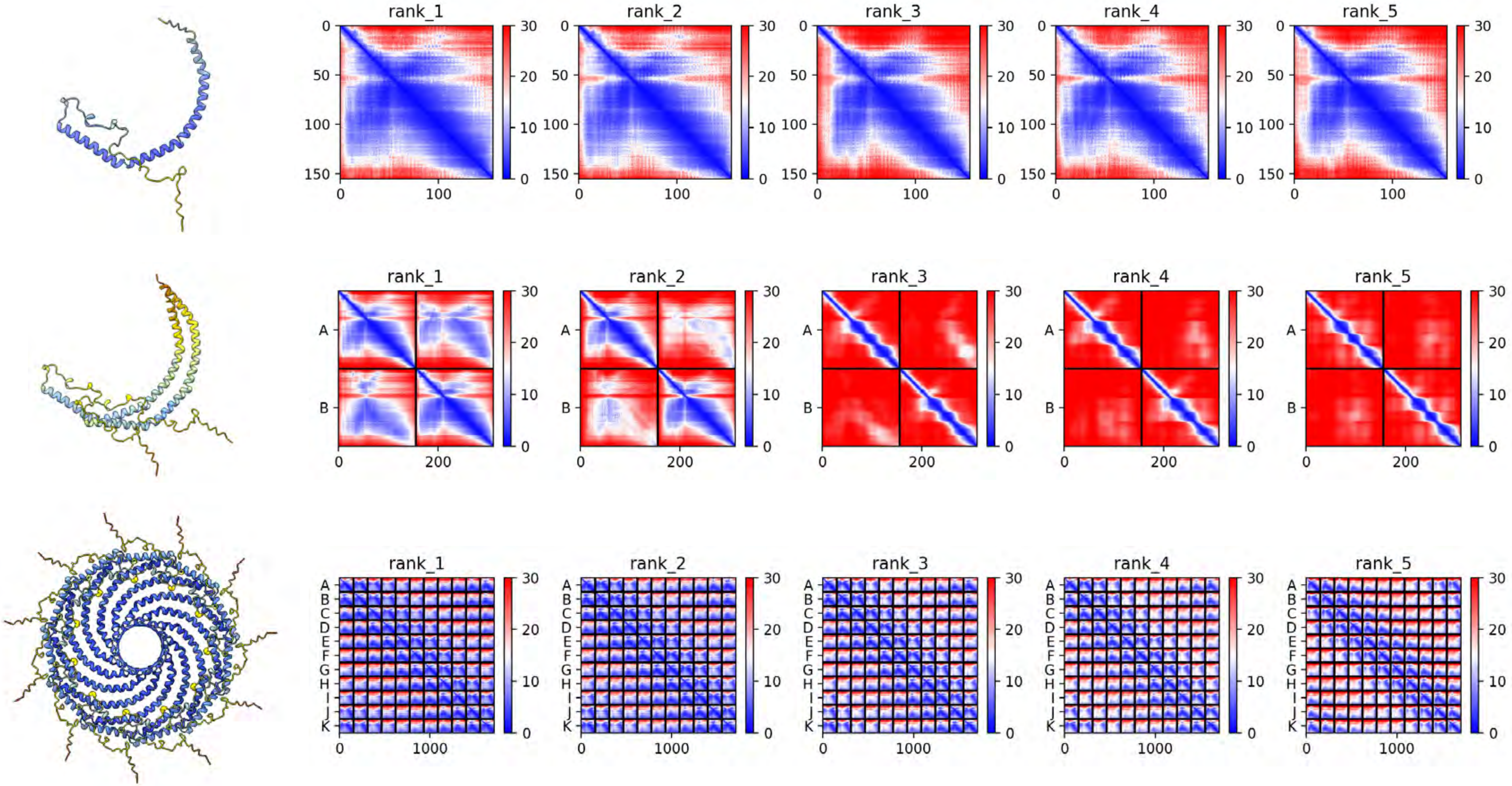

XP_052780907.1 *M. arenaria*

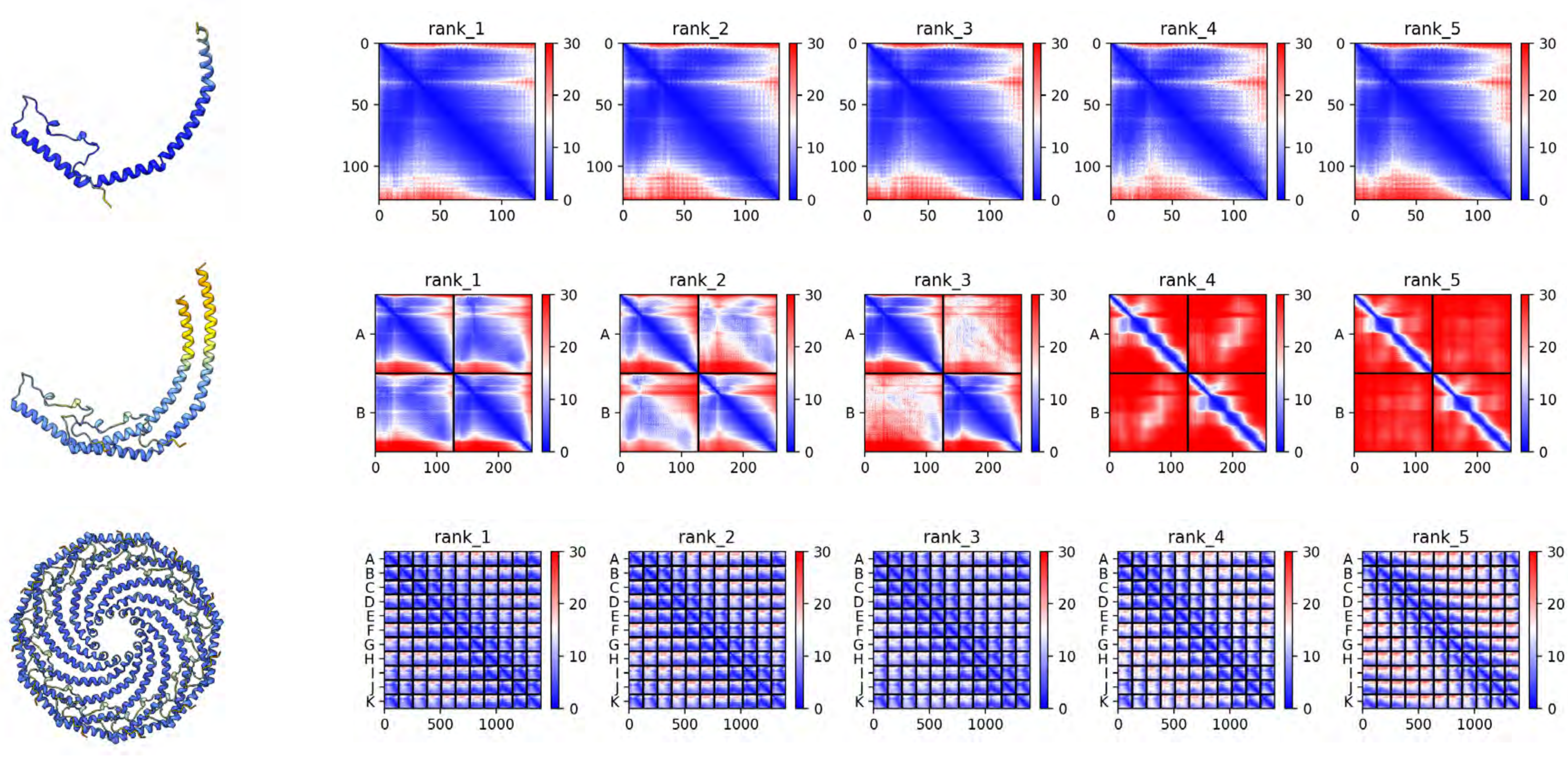

XP_052815466.1 *M. arenaria*

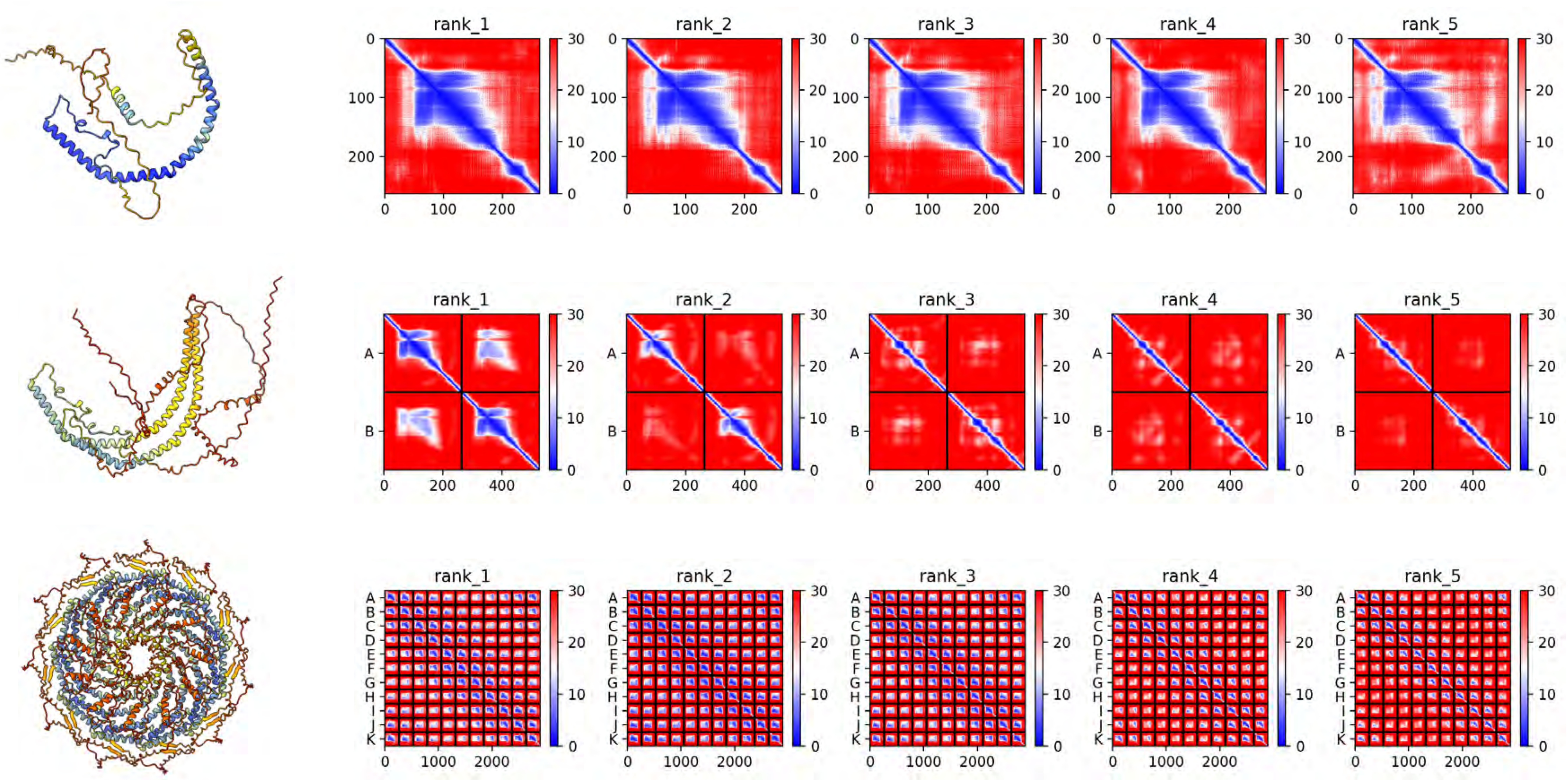

XP_052820175.1 *M. arenaria*

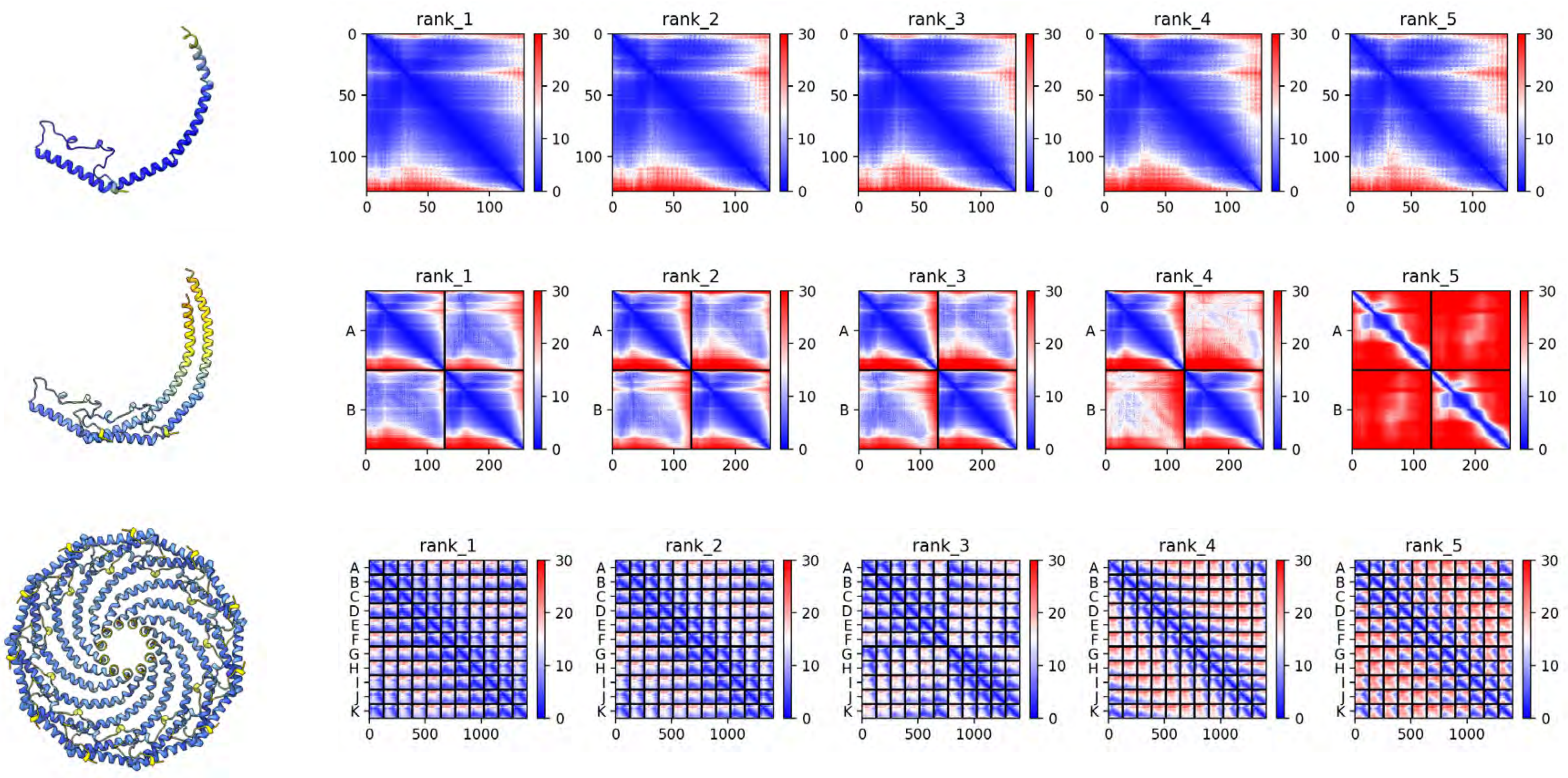

XP_052820174.1 *M. arenaria*

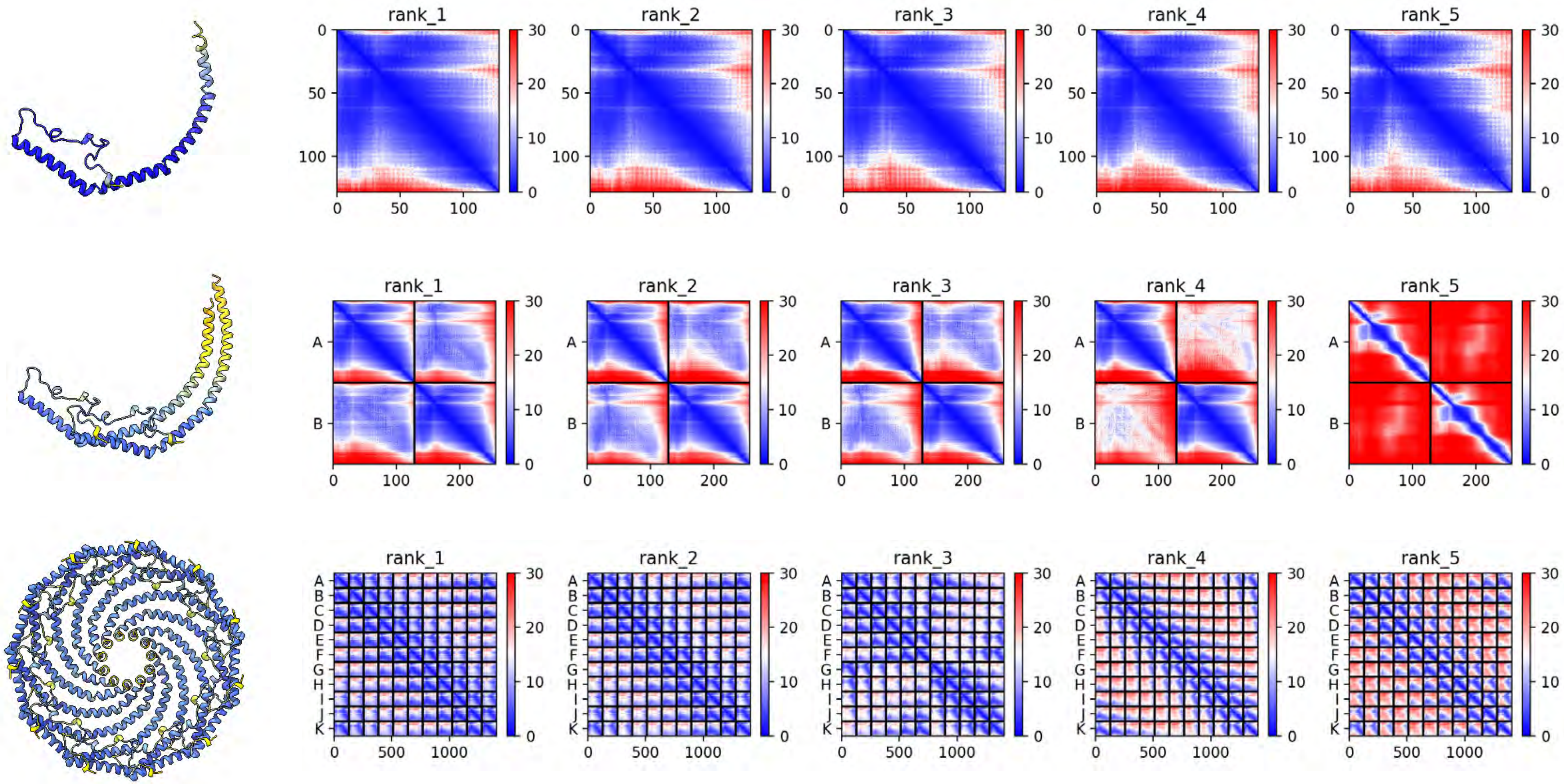

XP_052788808.1 *M. arenaria*

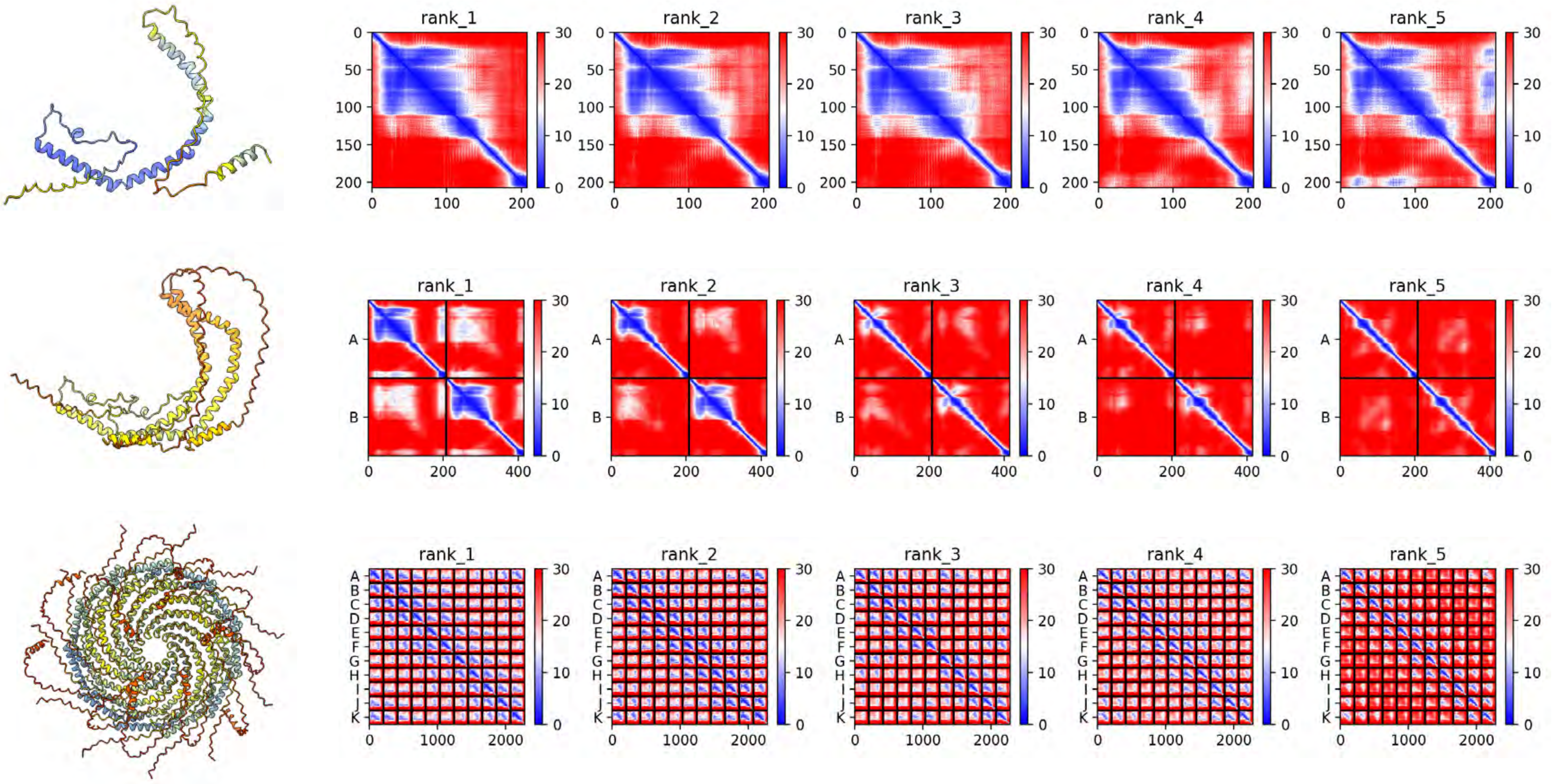

XP_052784964.1 *M. arenaria*

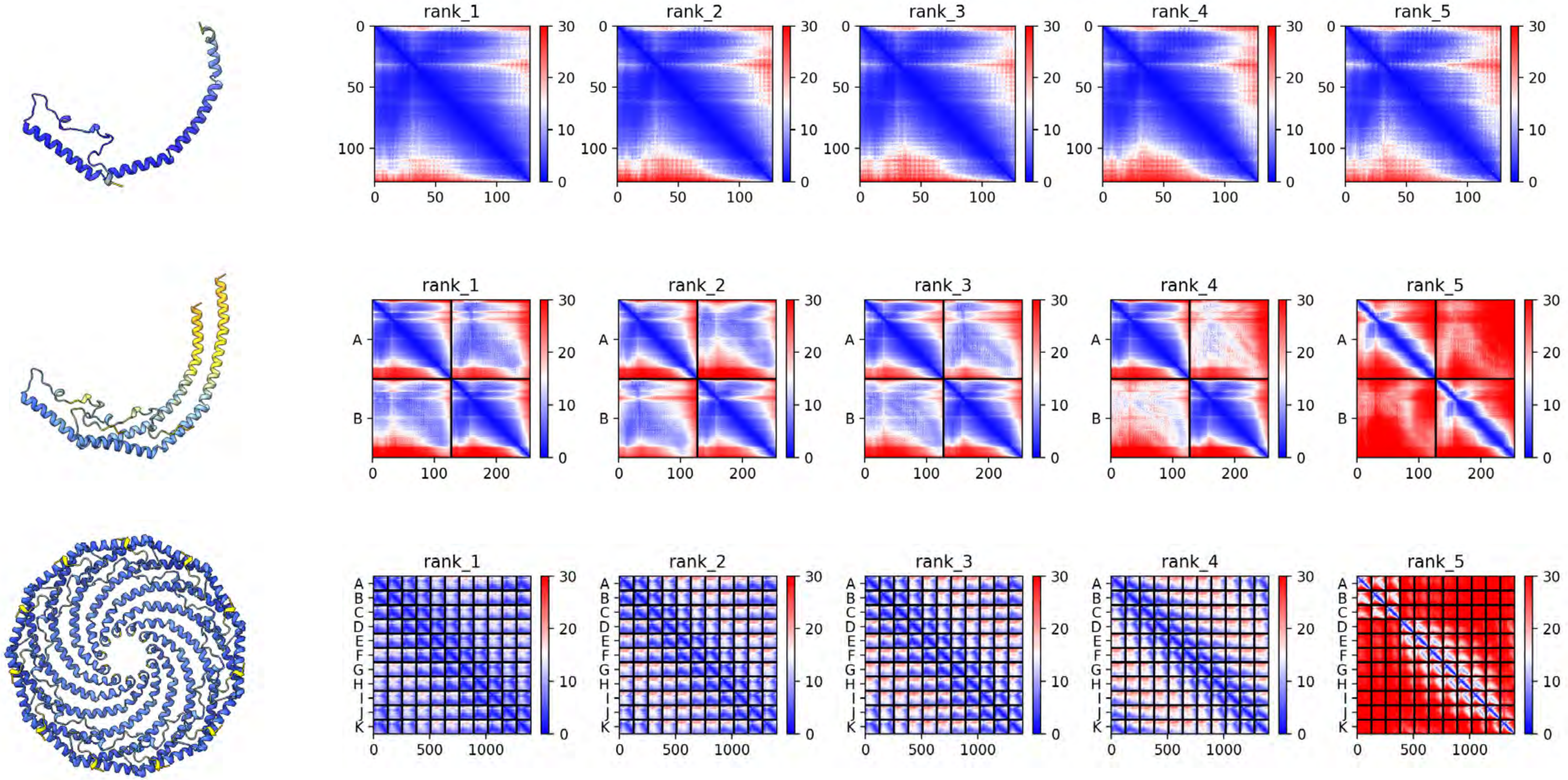

XP_053396032.1 *M. mercenaria*

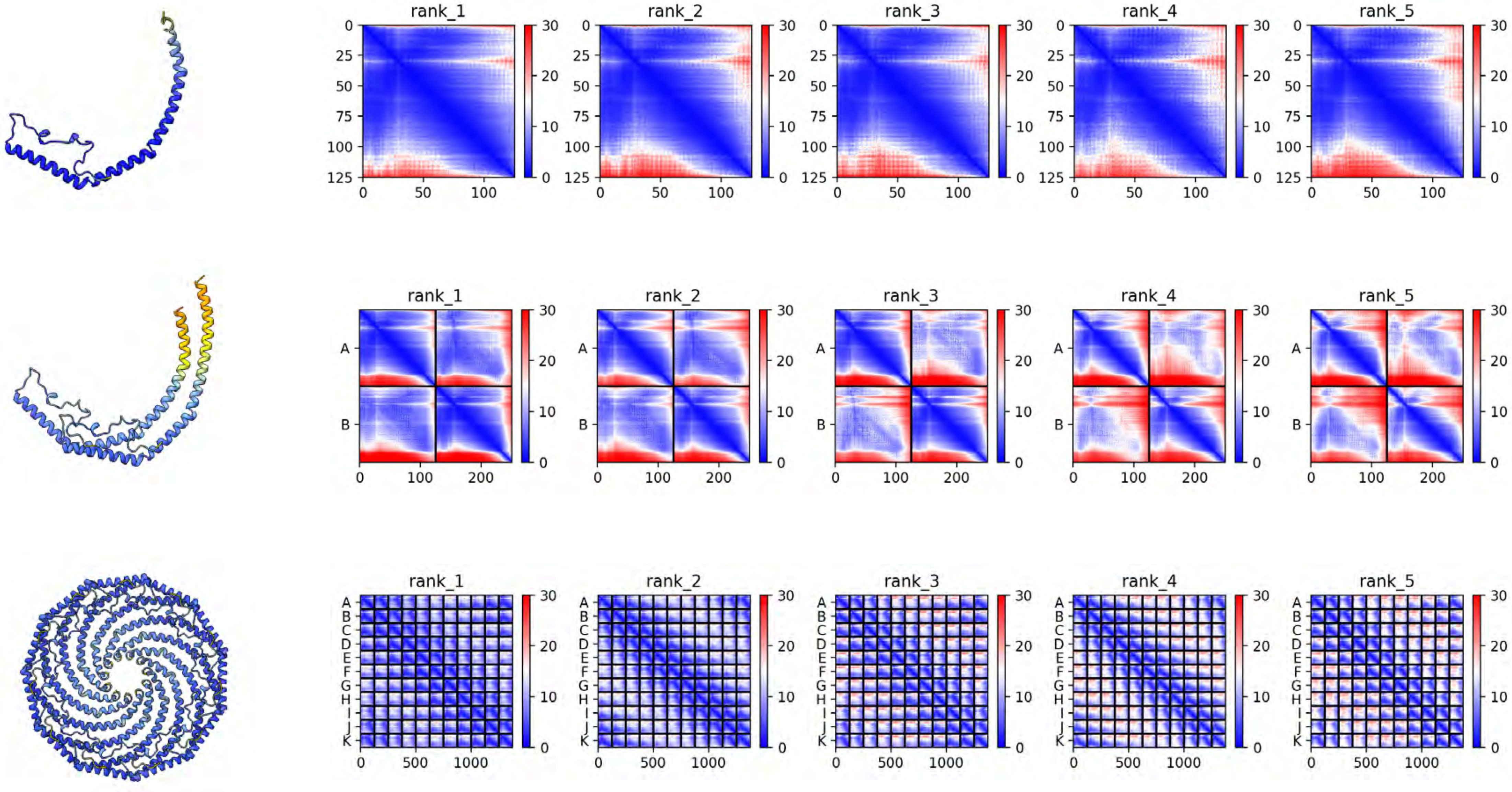

XP_045164732.2 *M. mercenaria*

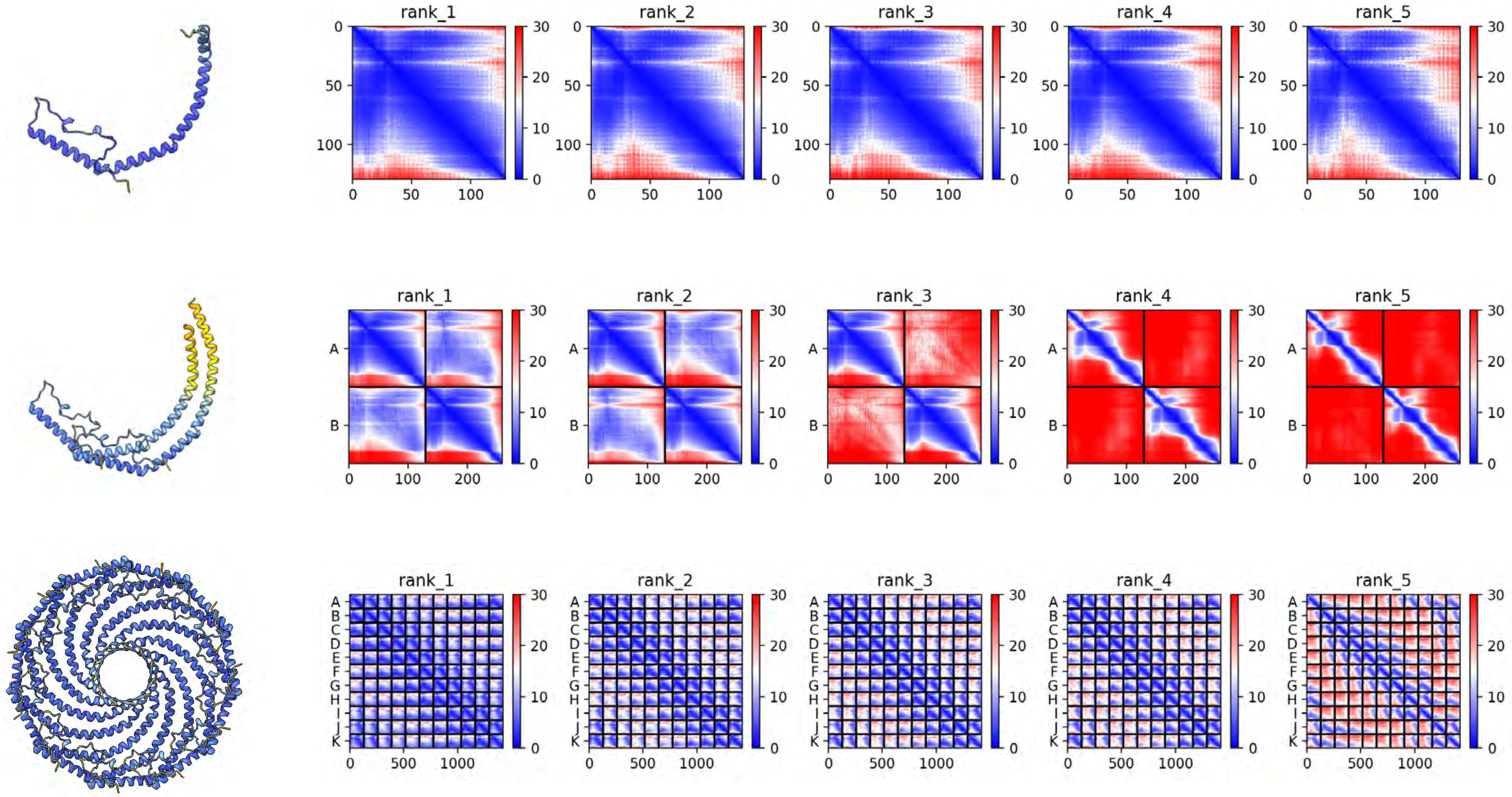

XP_053377544.1 *M. mercenaria*

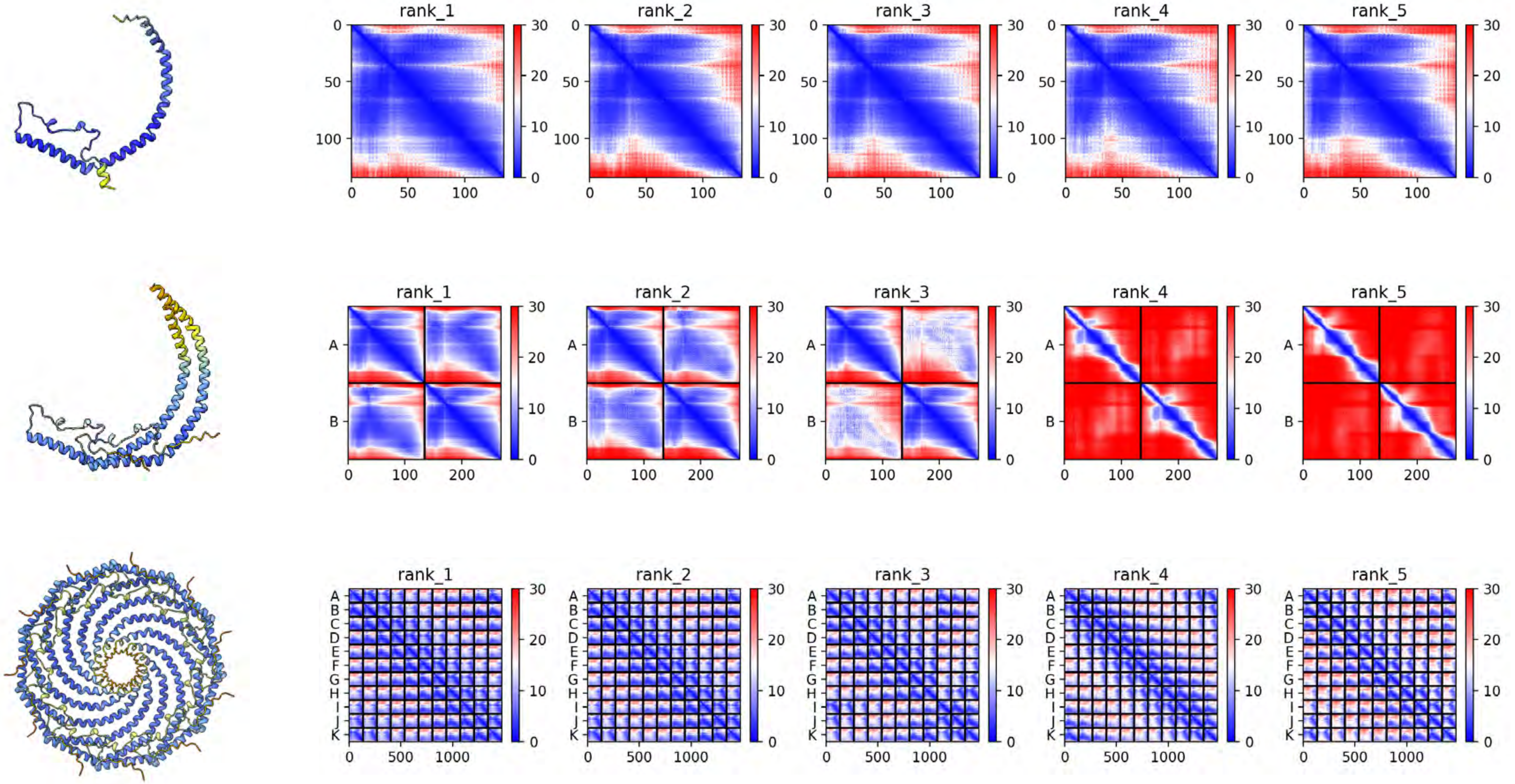

XP_045174338.2 *M. mercenaria*

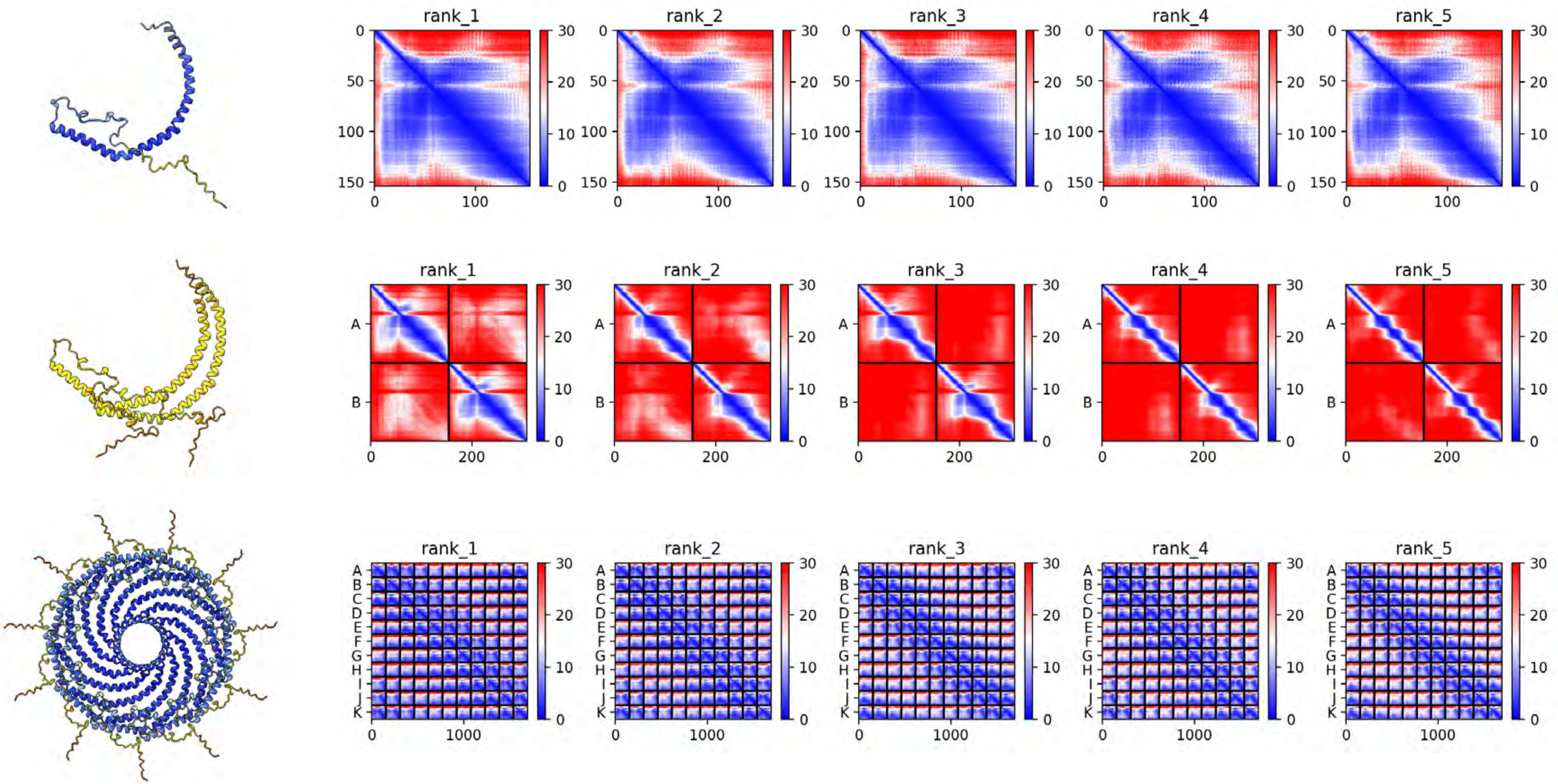

XP_033630427.1 *A. rubens*

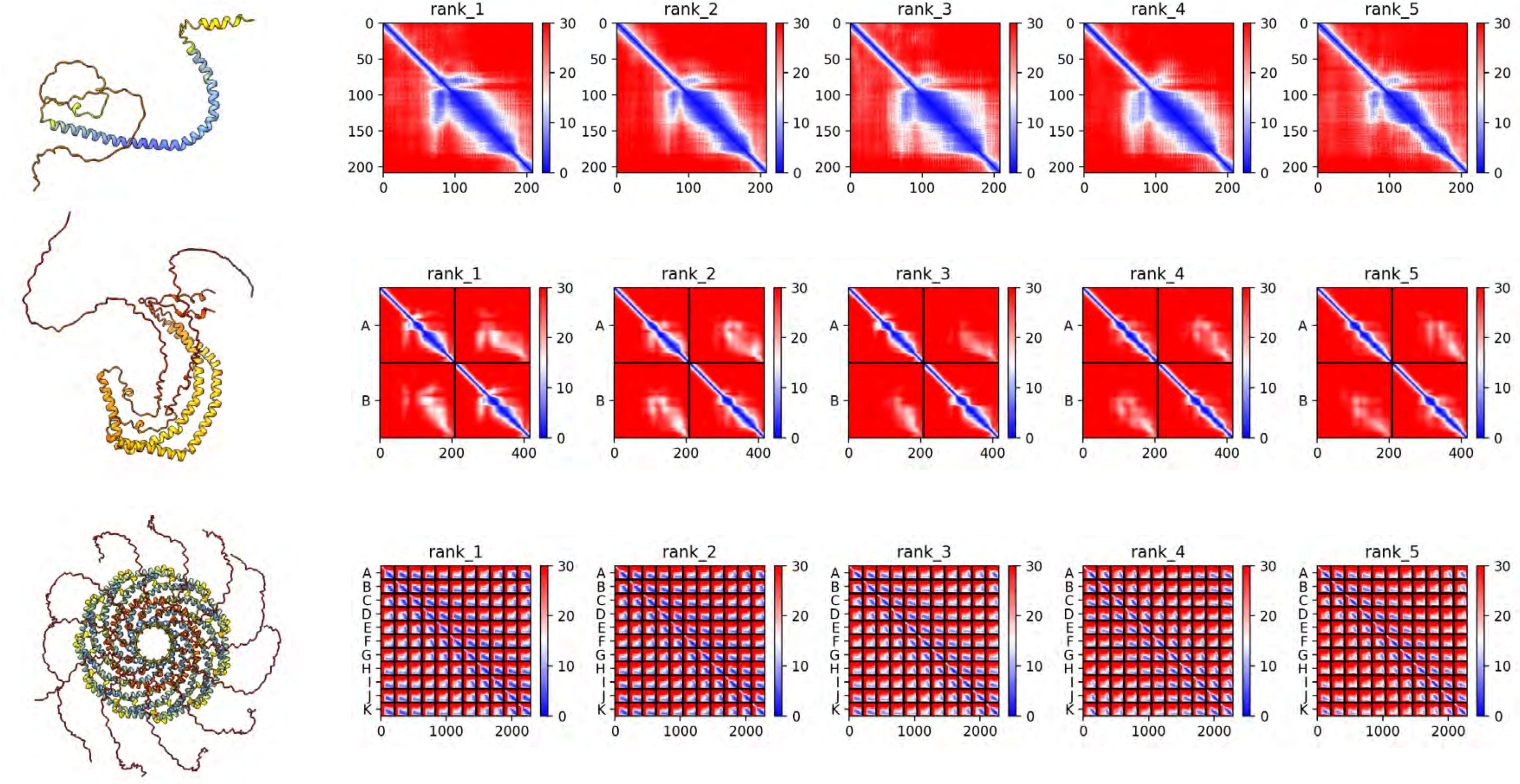

XP_033636910.1 *A. rubens*

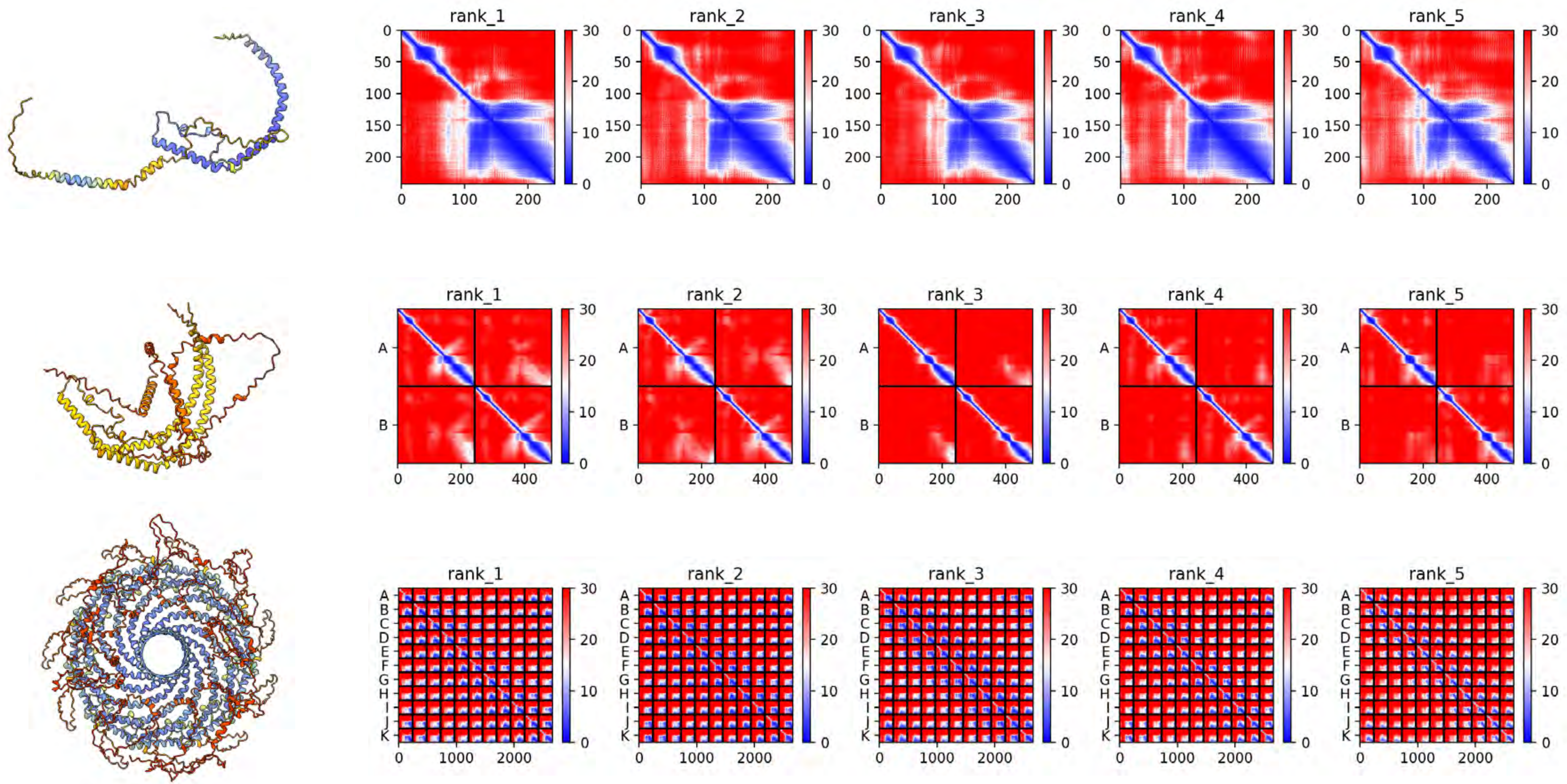

XP_041476235.1 *L. variegatus*

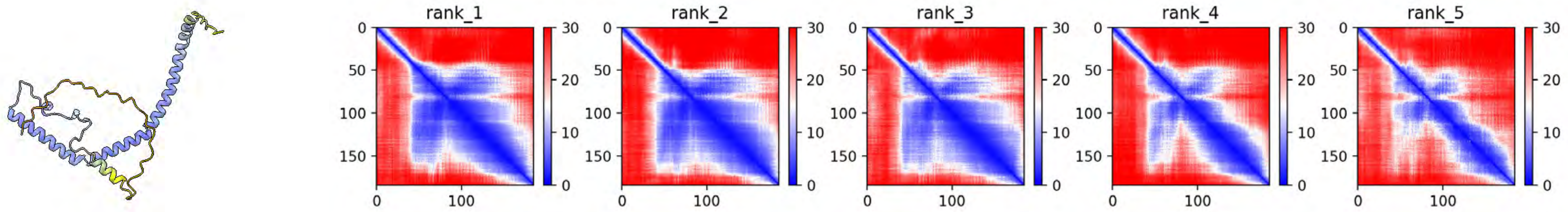

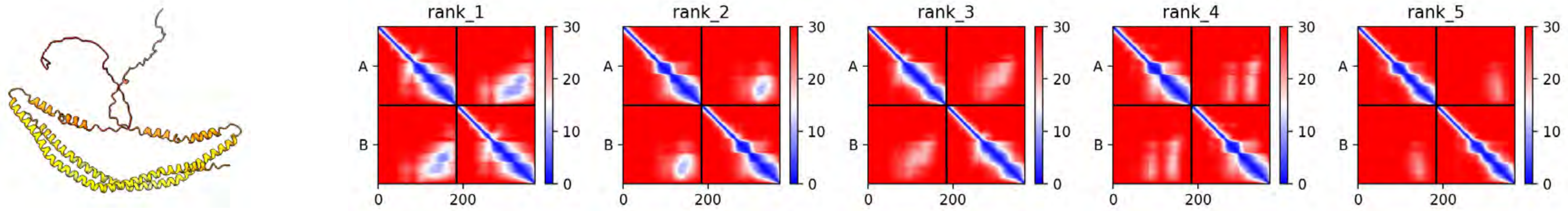

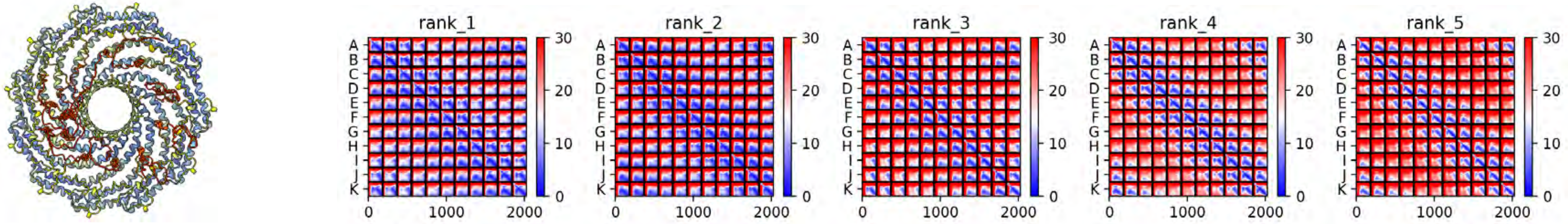

XP_041469030.1 *L. variegatus*

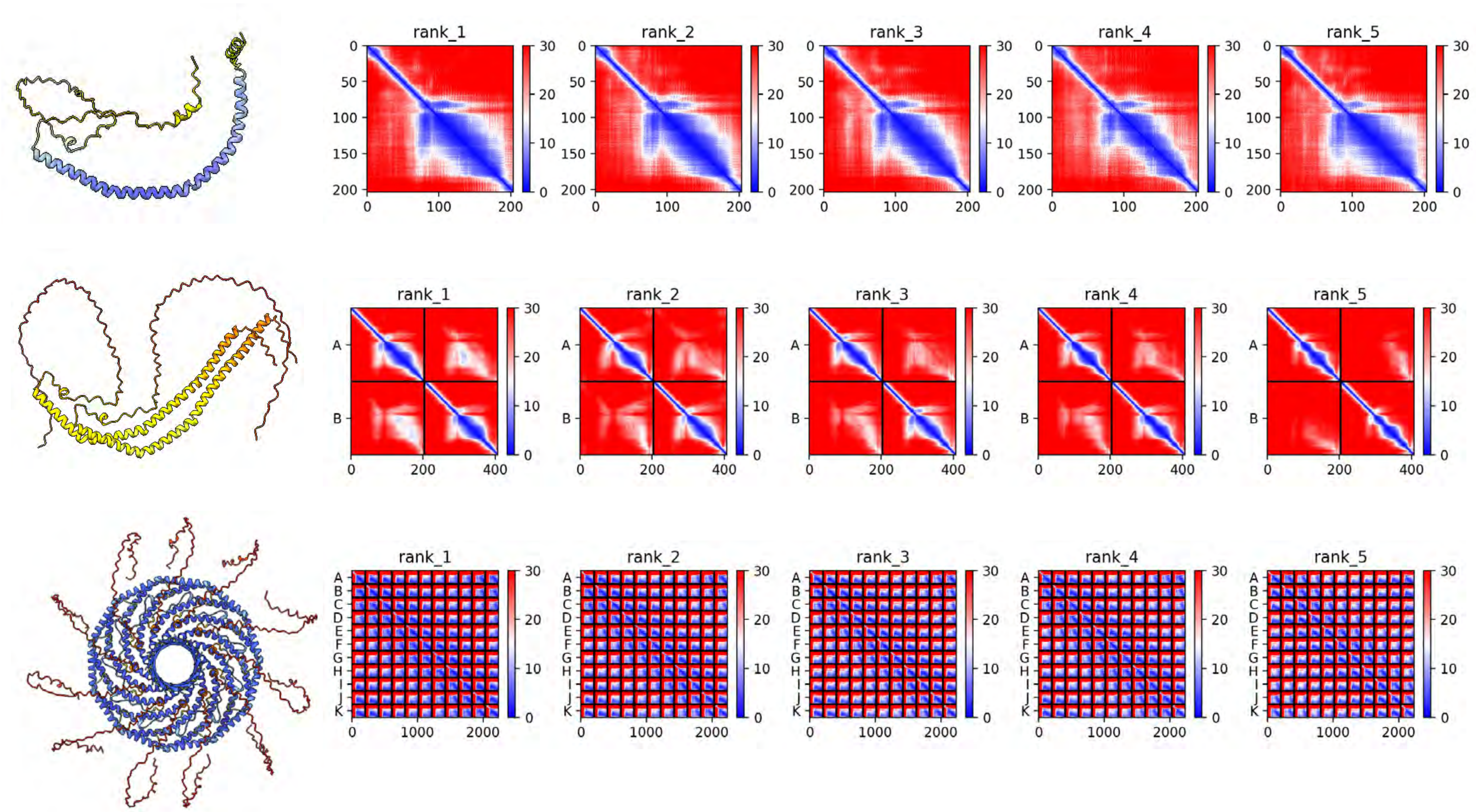

XP_041454781.1 *L. variegatus*

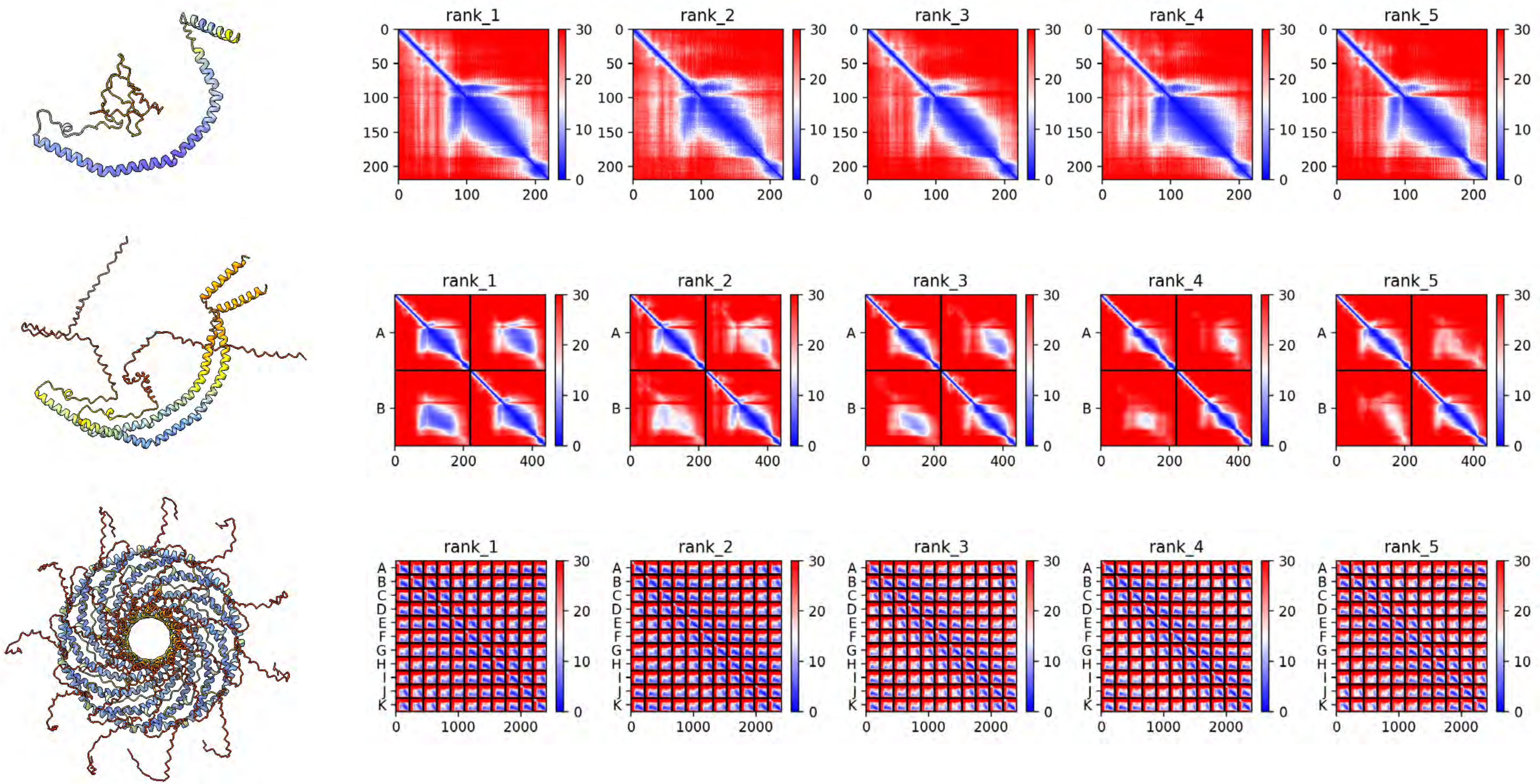

XP_041460678.1 *L. variegatus*

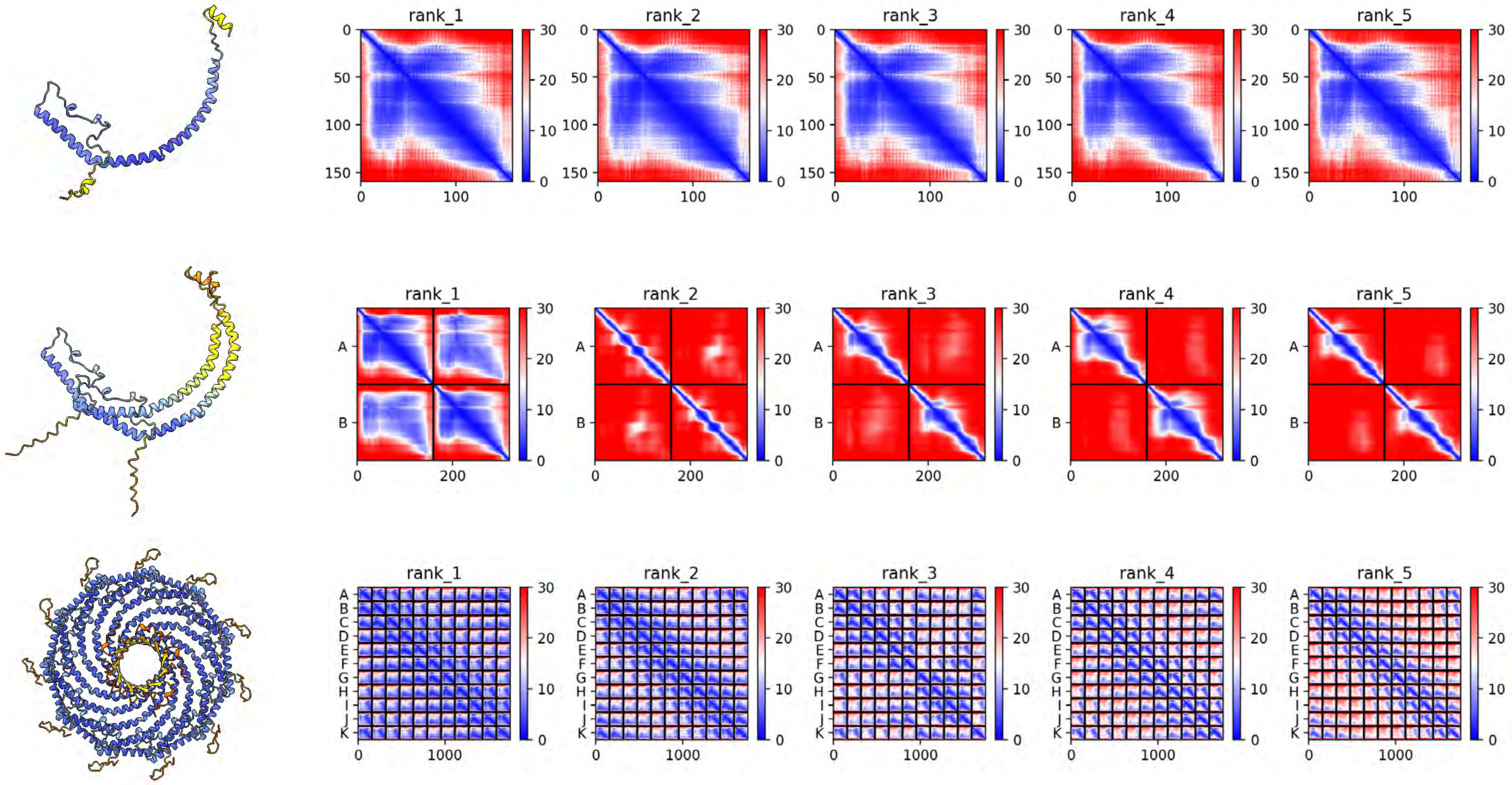

XP_054764459.1 *L. pictus*

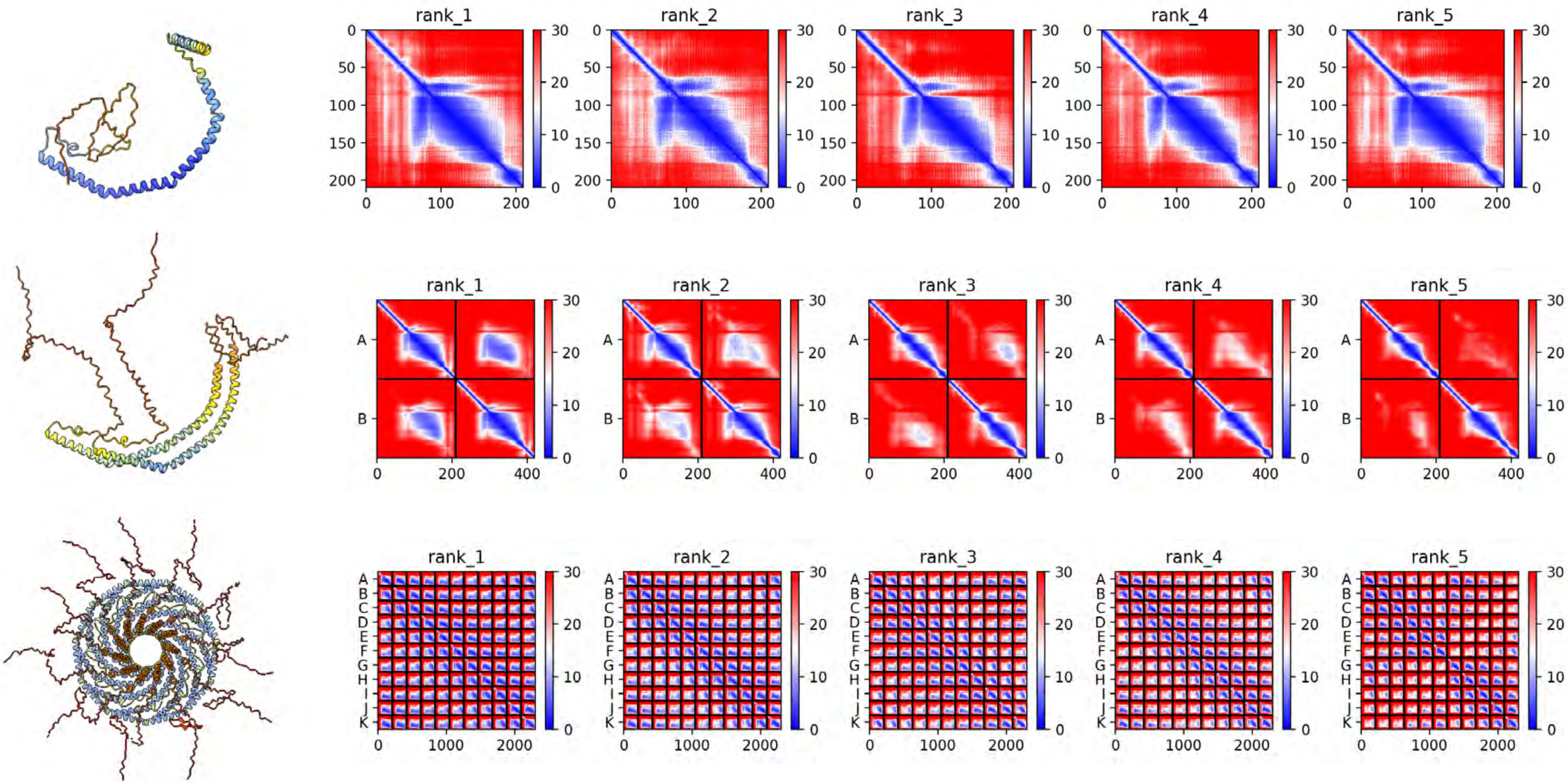

XP_054752263.1 *L. pictus*

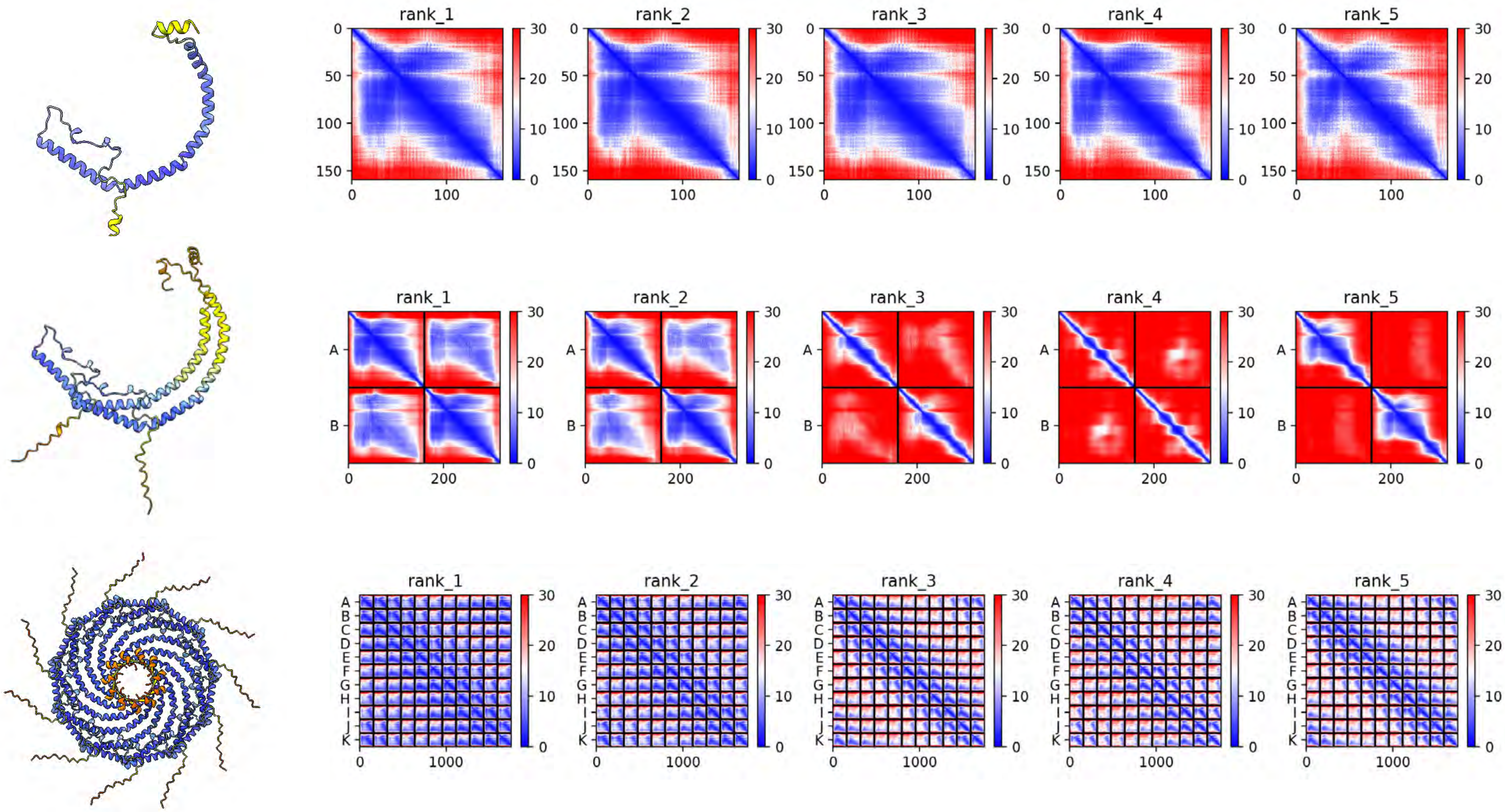

XP_054769514.1 *L. pictus*

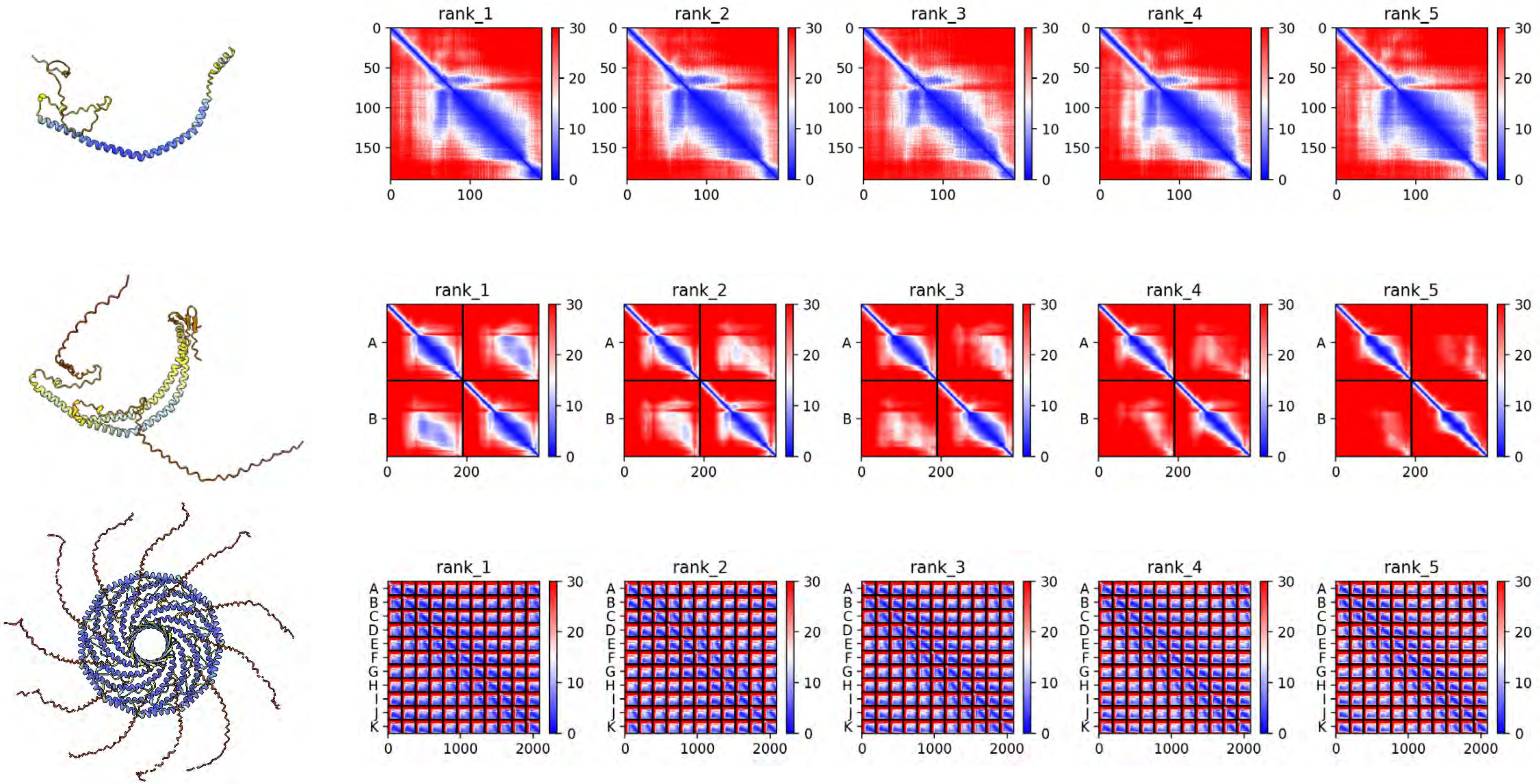

XP_054764941.1 *L. pictus*

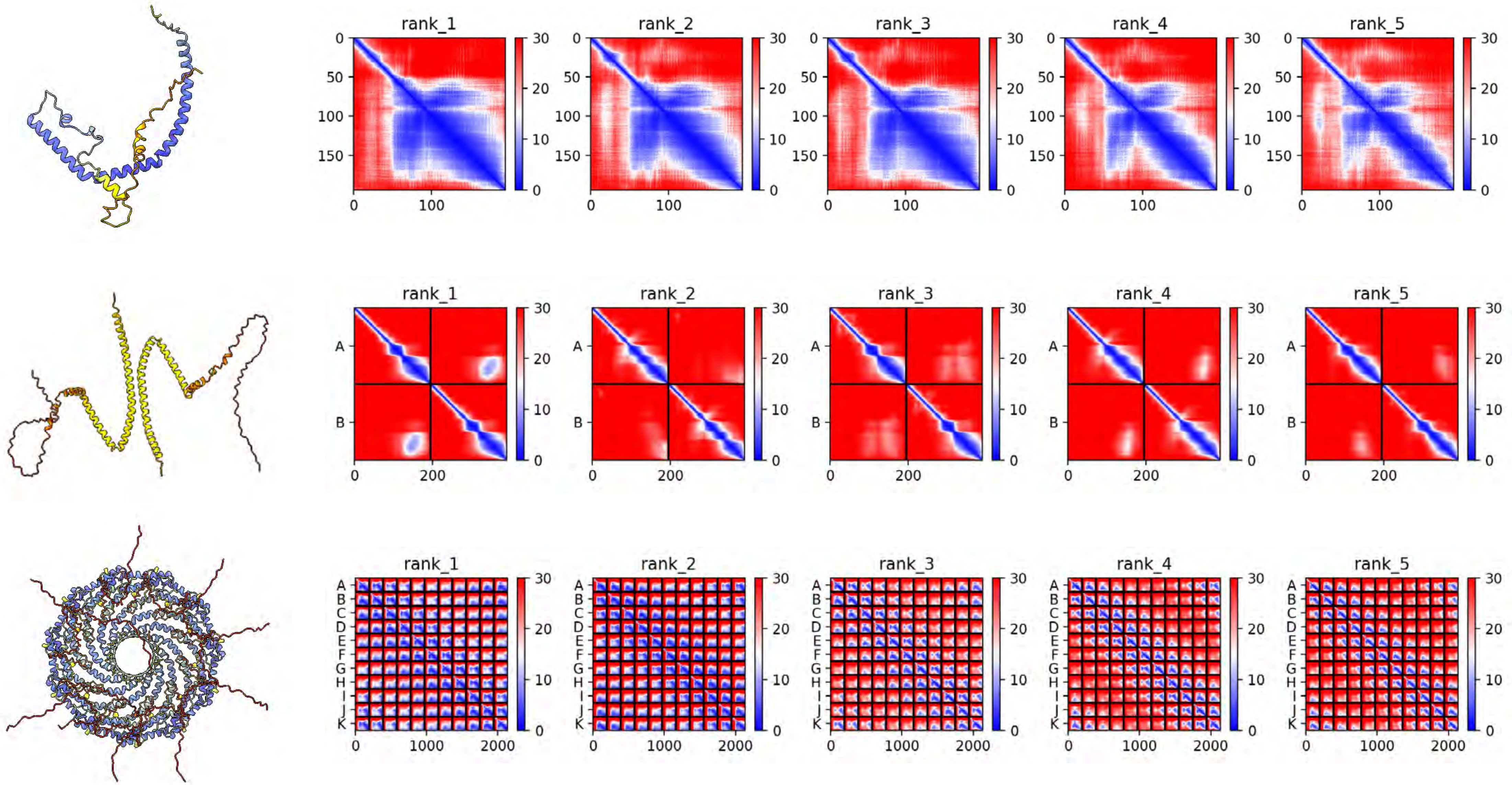

KAJ8040503.1 *H. leucospilota*

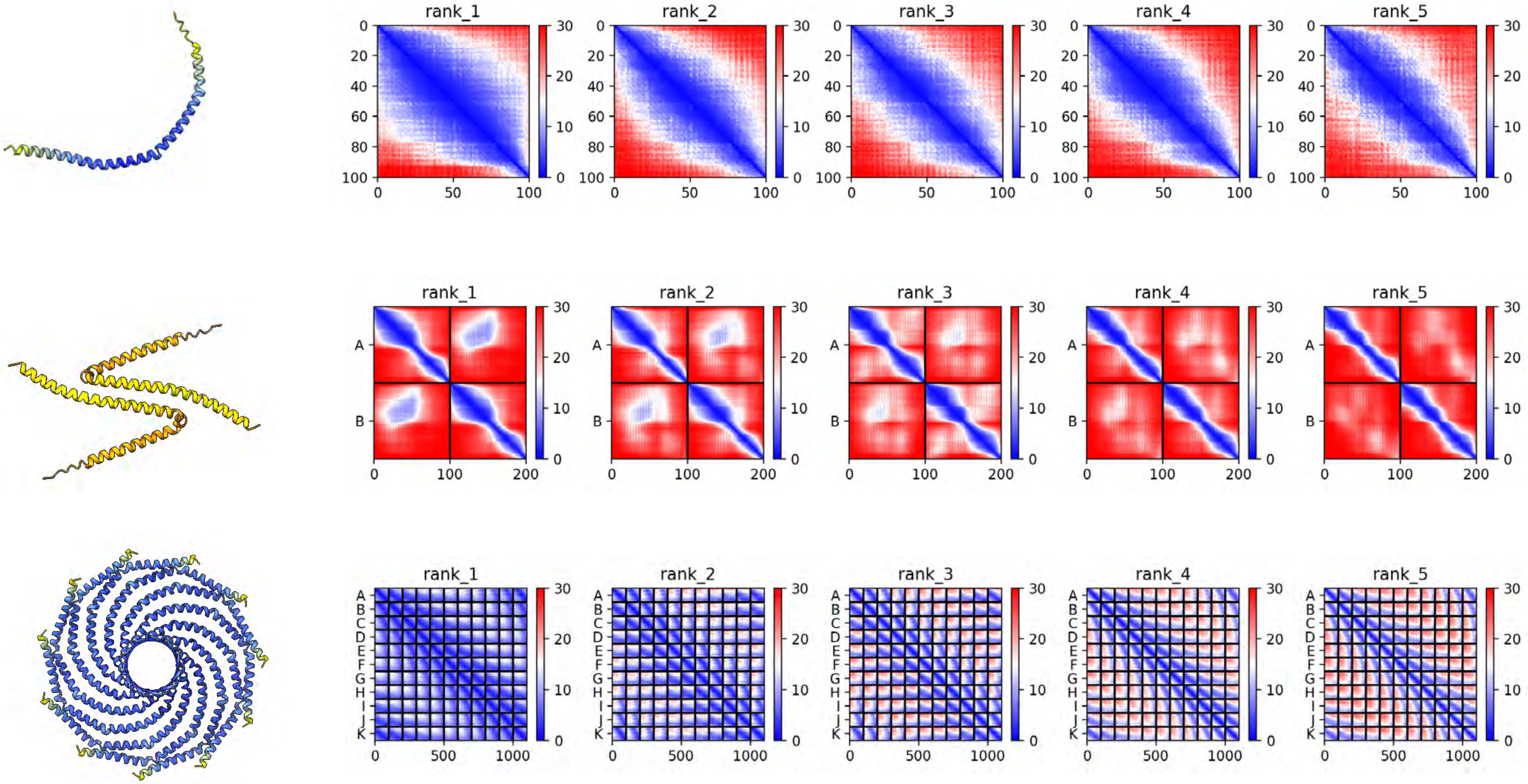

KAJ8046320.1 *H. leucospilota*

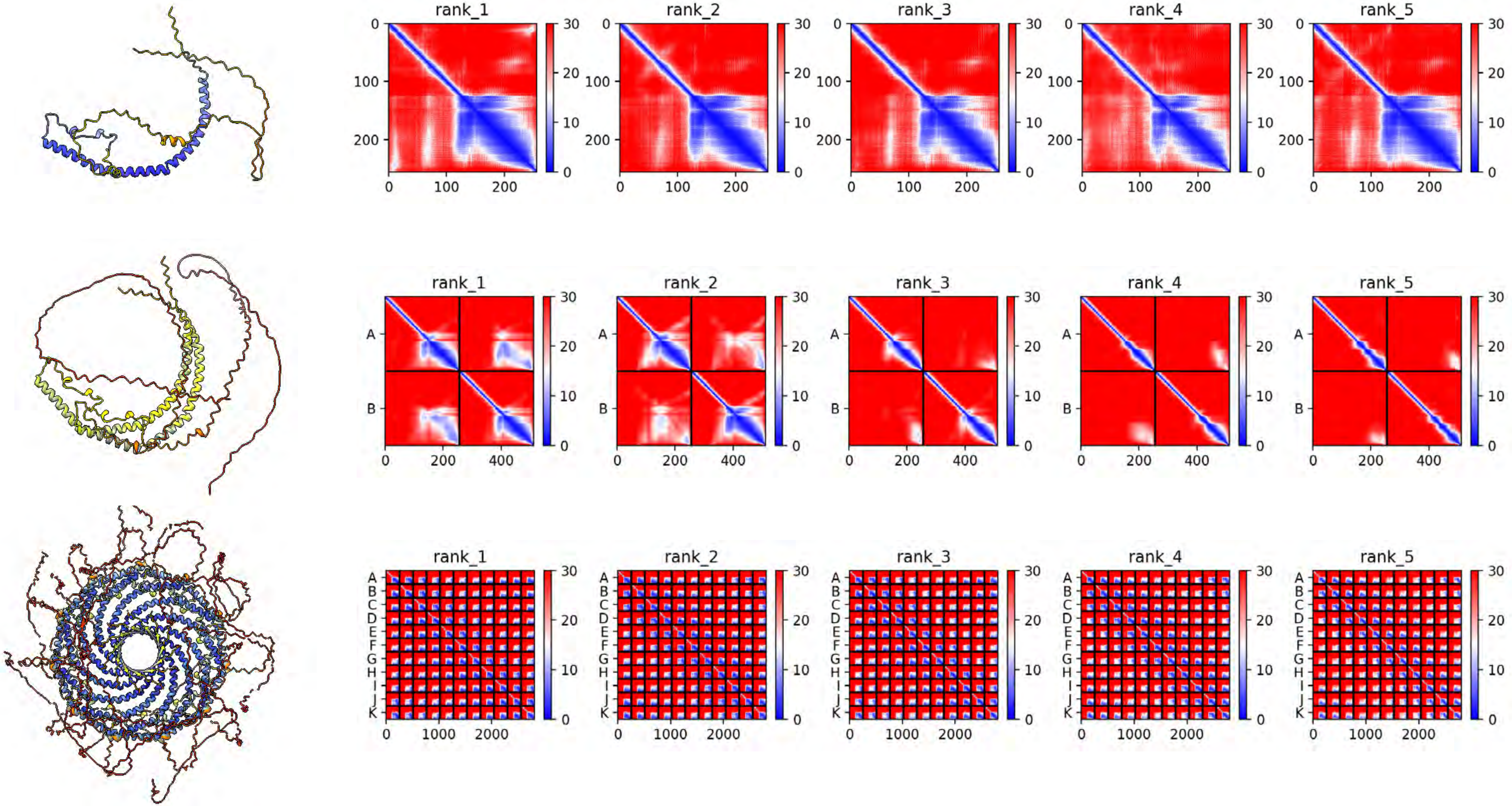

KAJ8022872.1 *H. leucospilota*

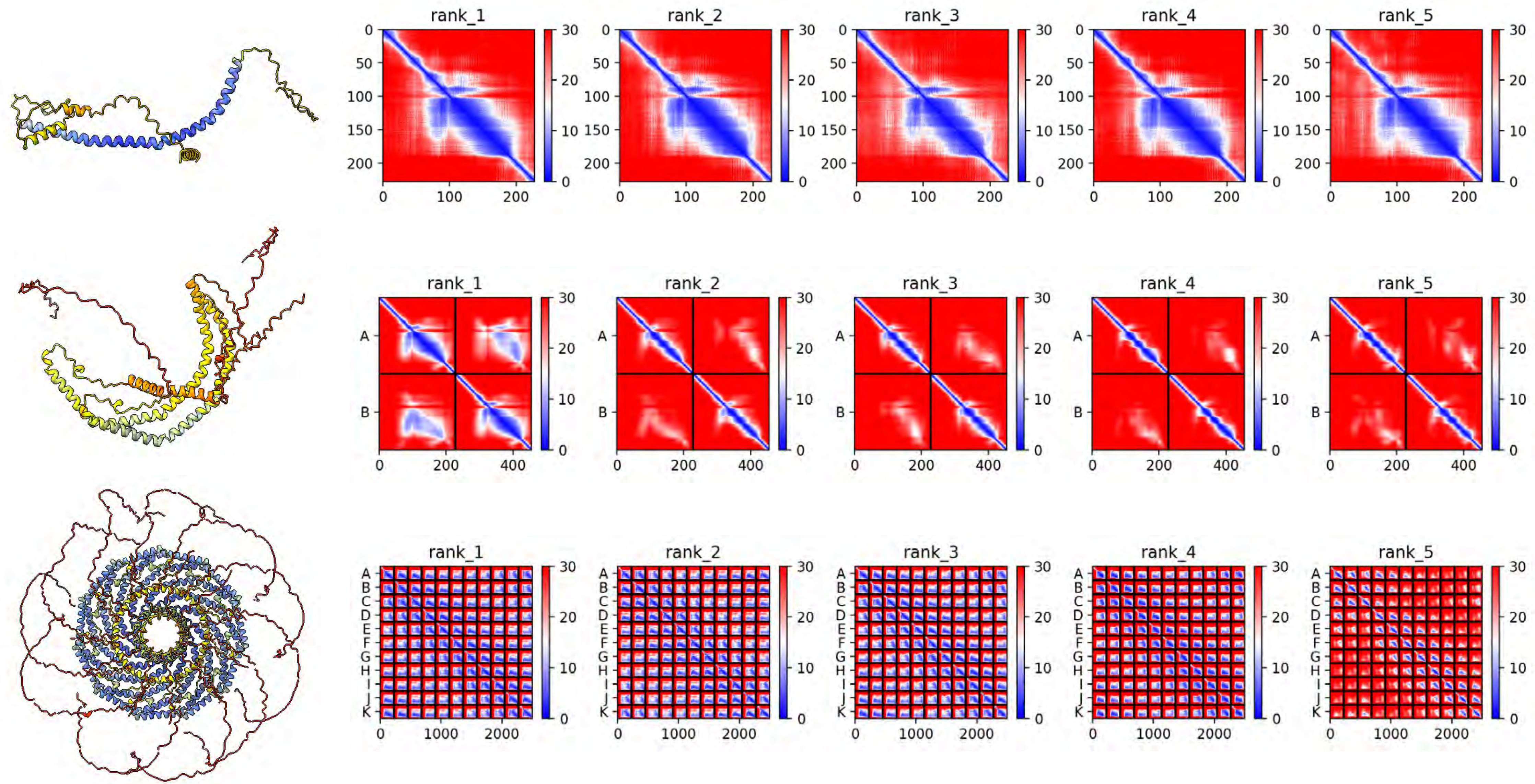

KAJ8020606.1 *H. leucospilota*

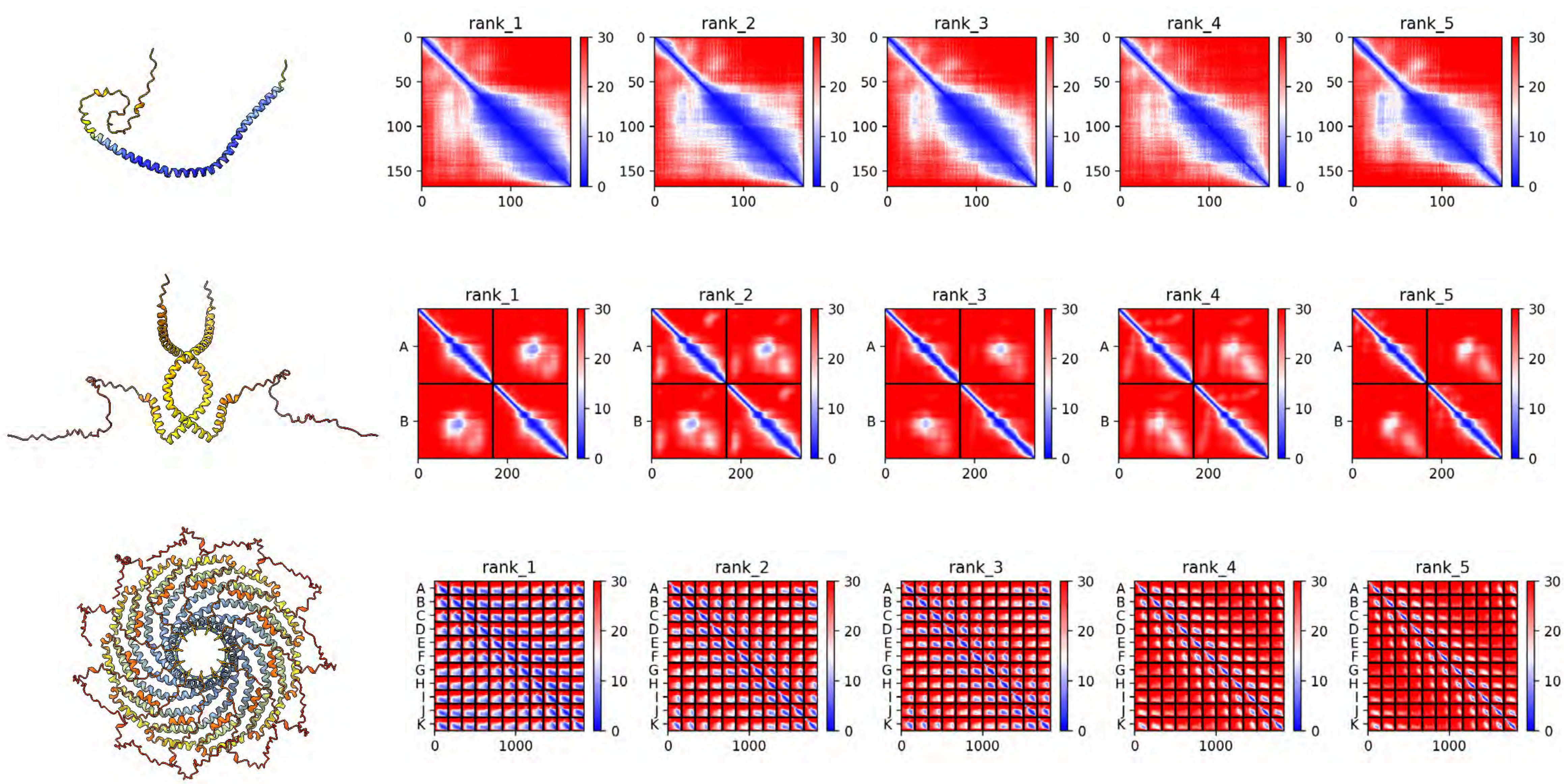

XP_061423086.1 *L. reissneri*

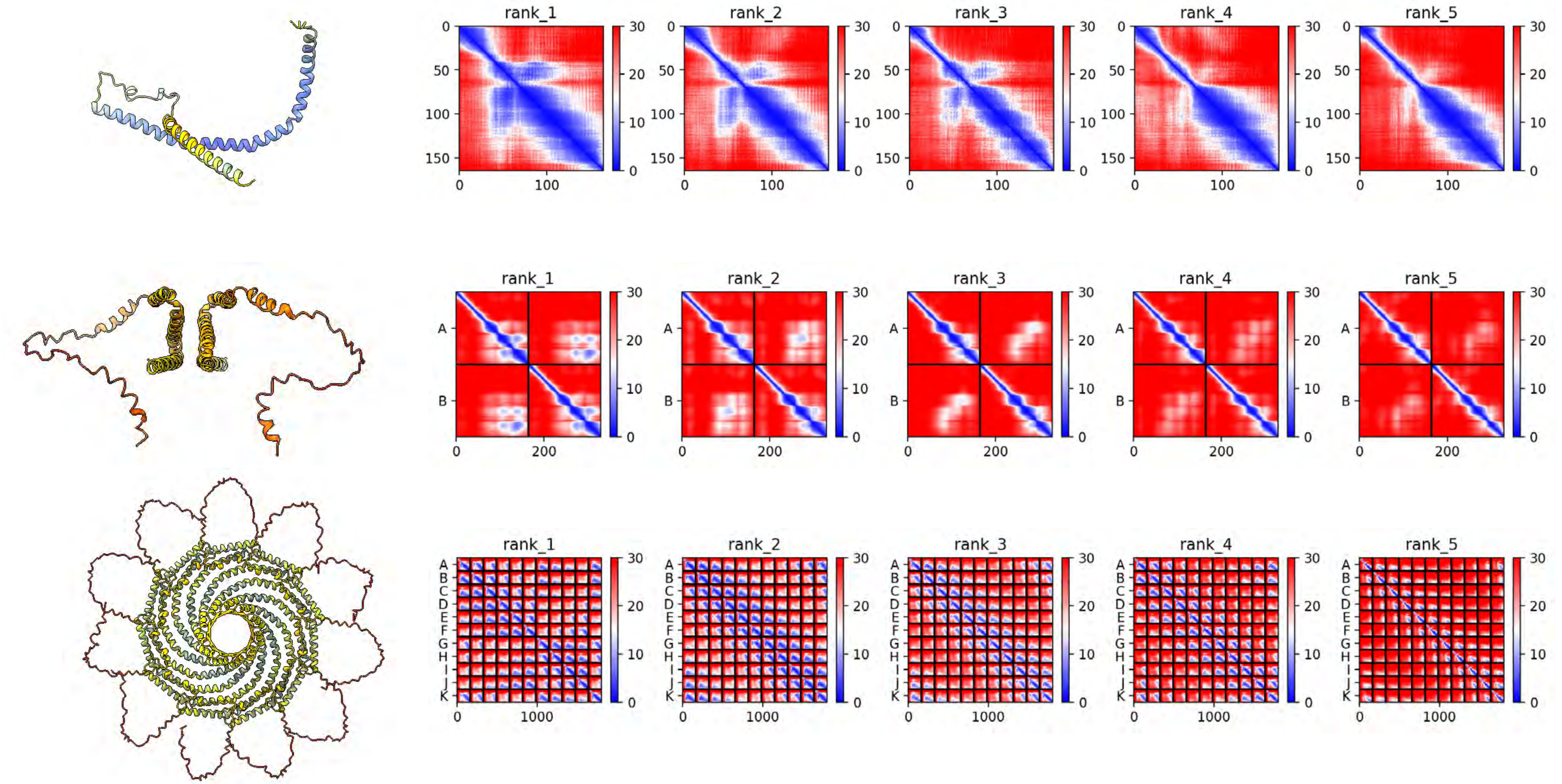

XP_061428890.1 *L. reissneri*

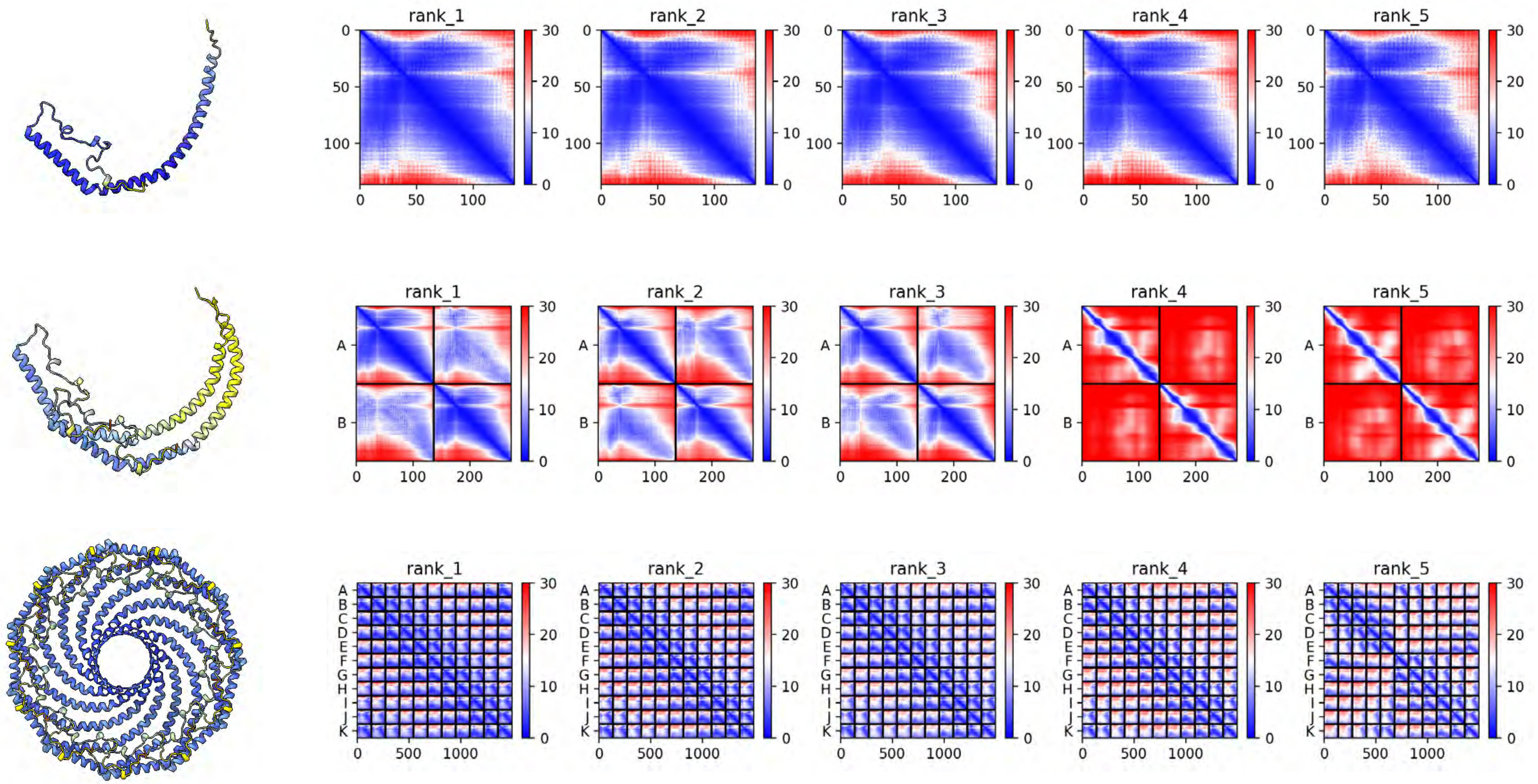

XP_061428788.1 *L. reissneri*

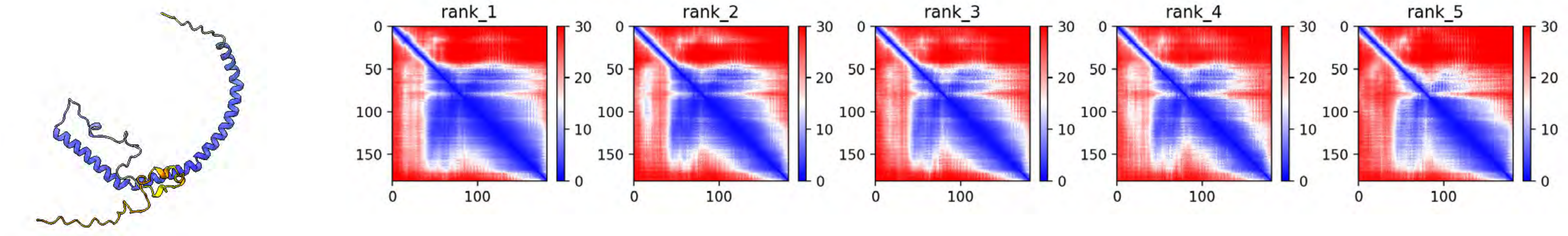

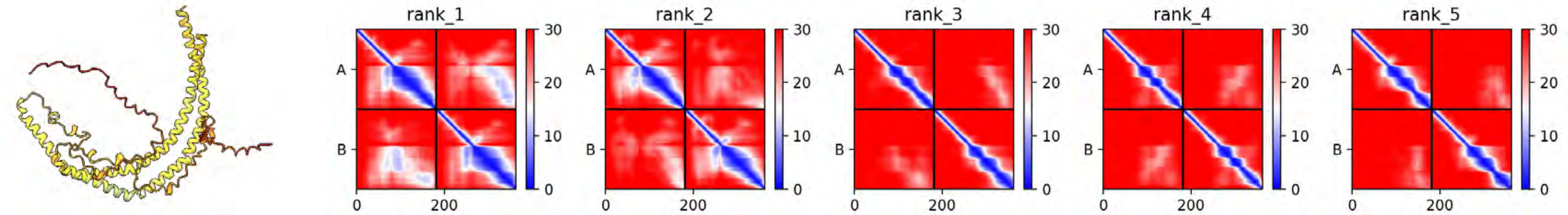

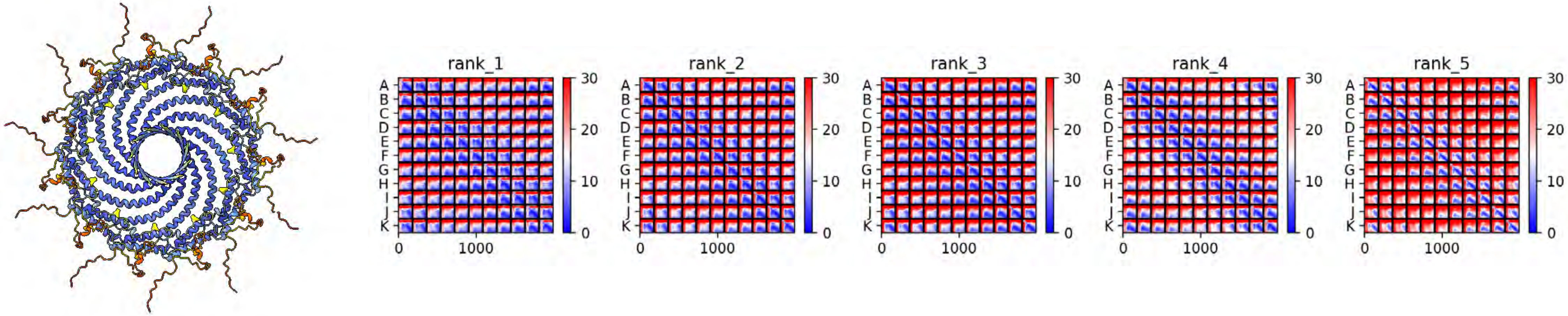

XP_032827412.1 *P. marinus*

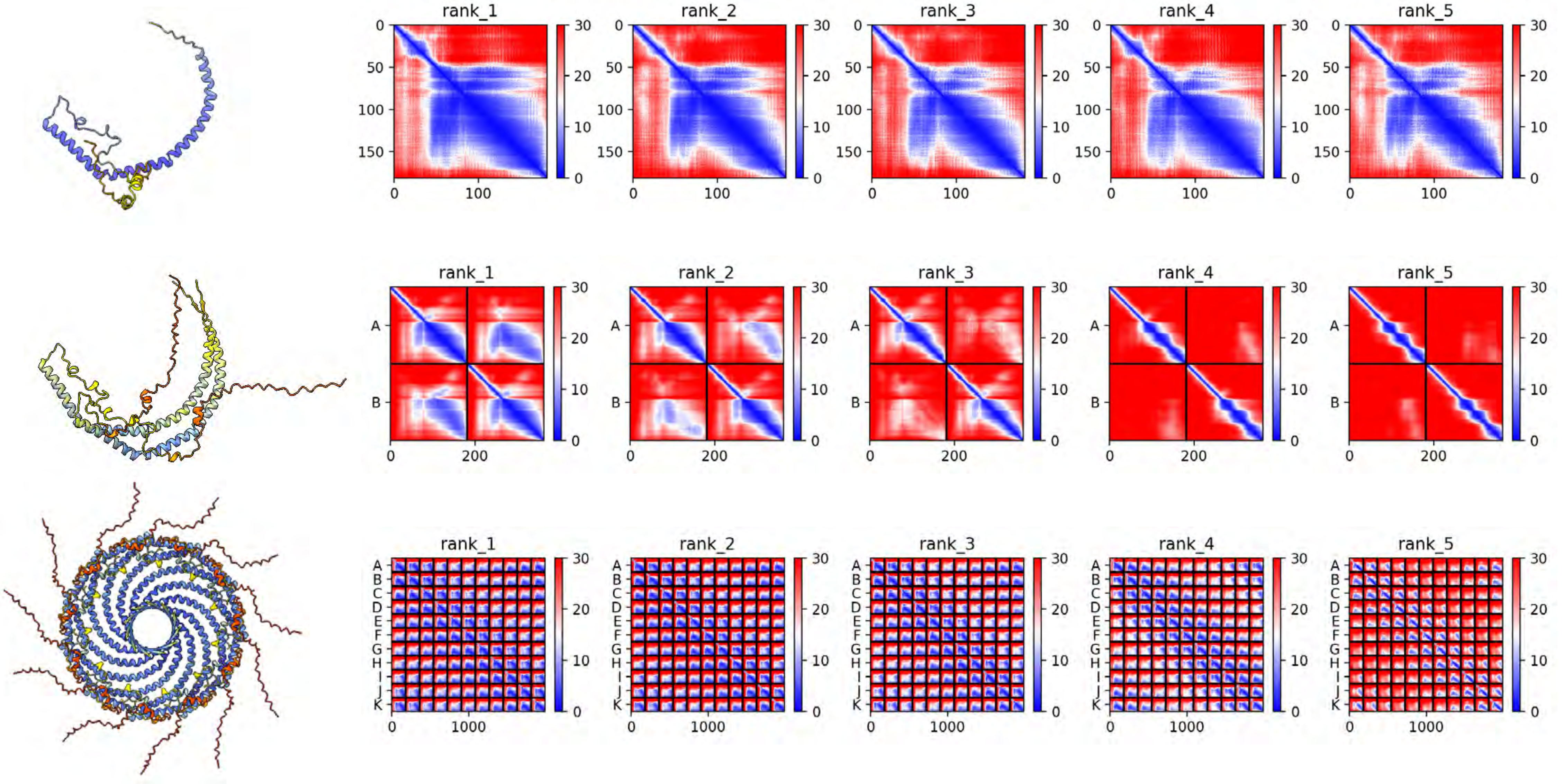

XP_032819264.1 *P. marinus*

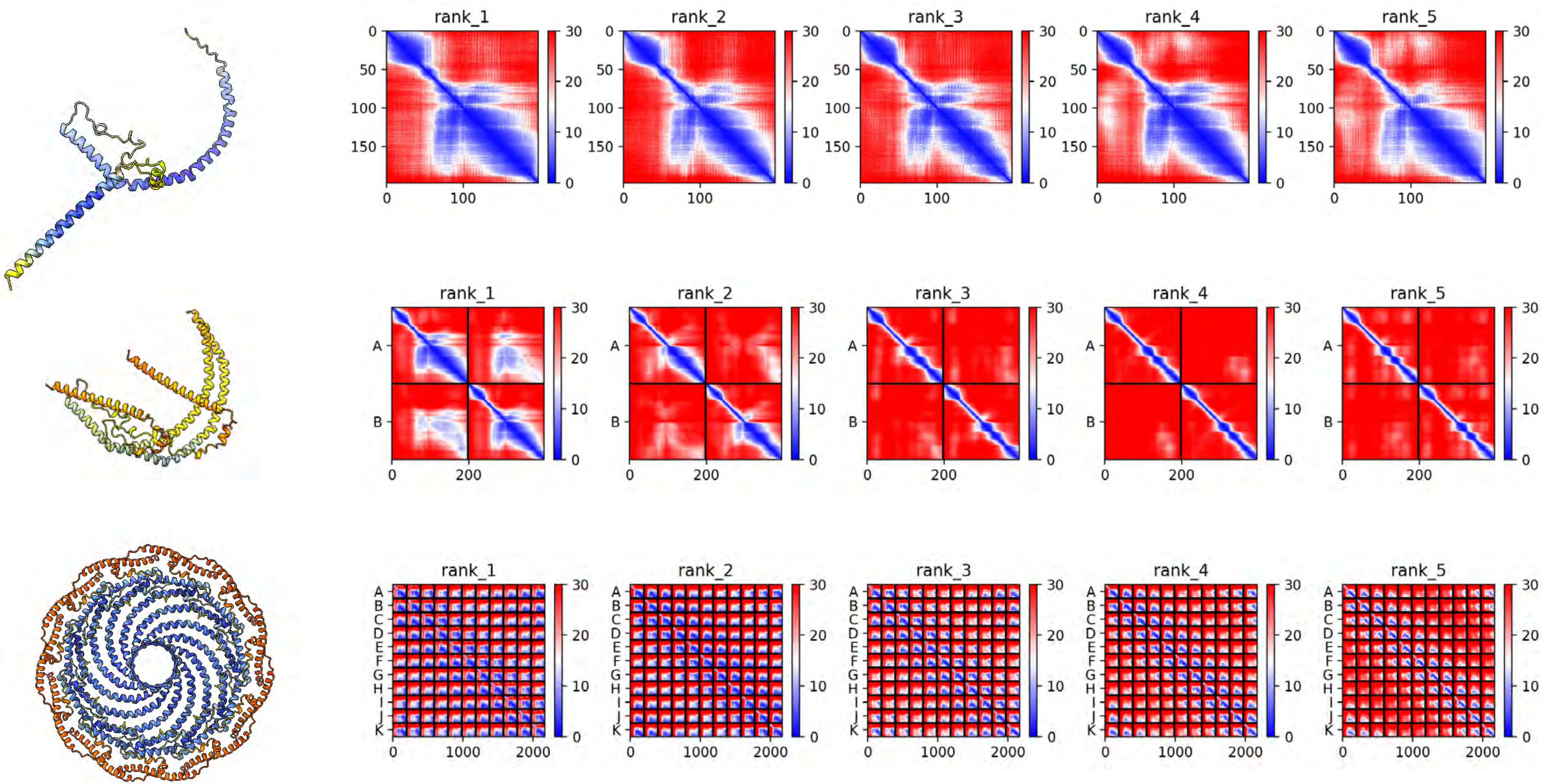

XP_032819263.1 *P. marinus*

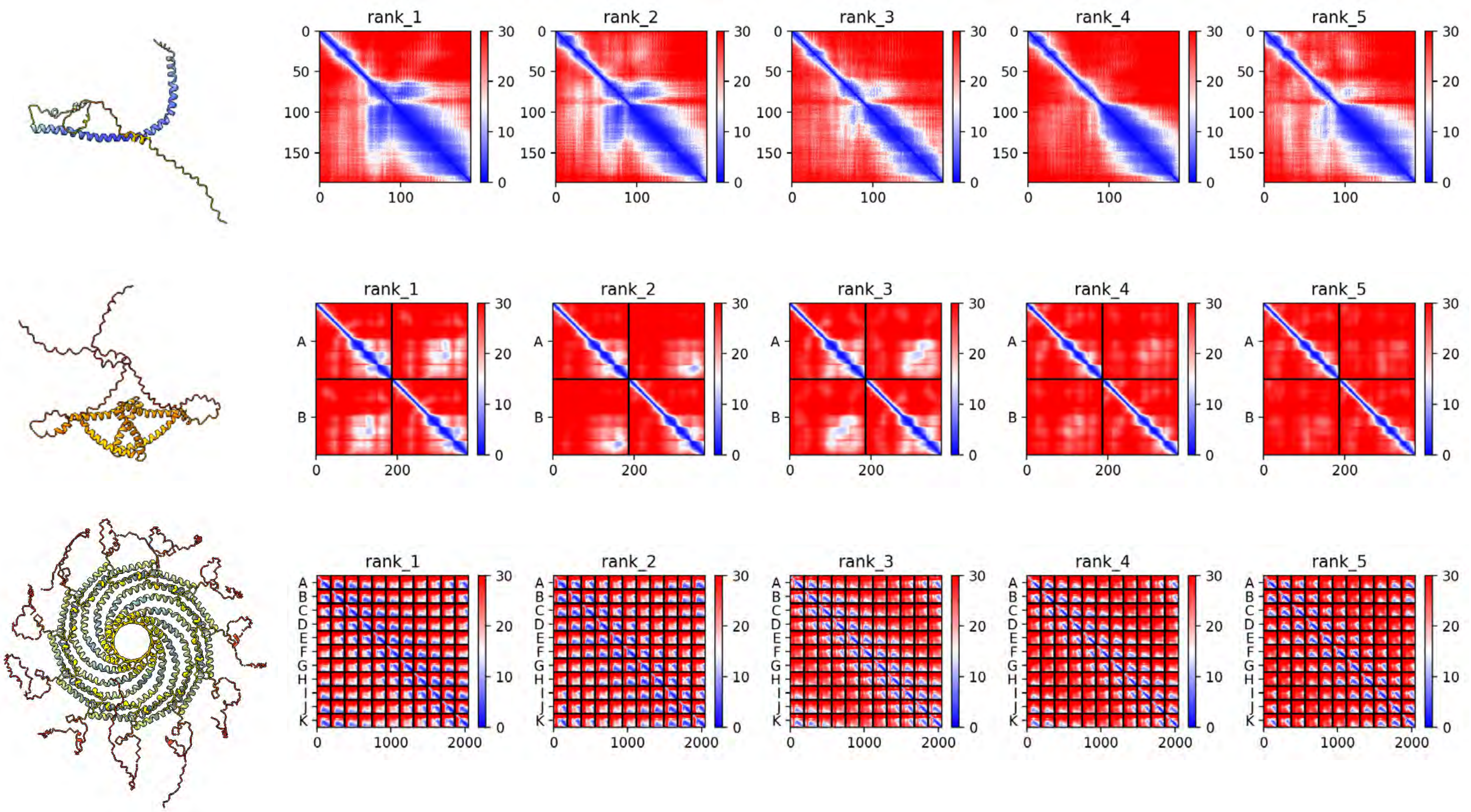

XP_032827409.1 *P. marinus*

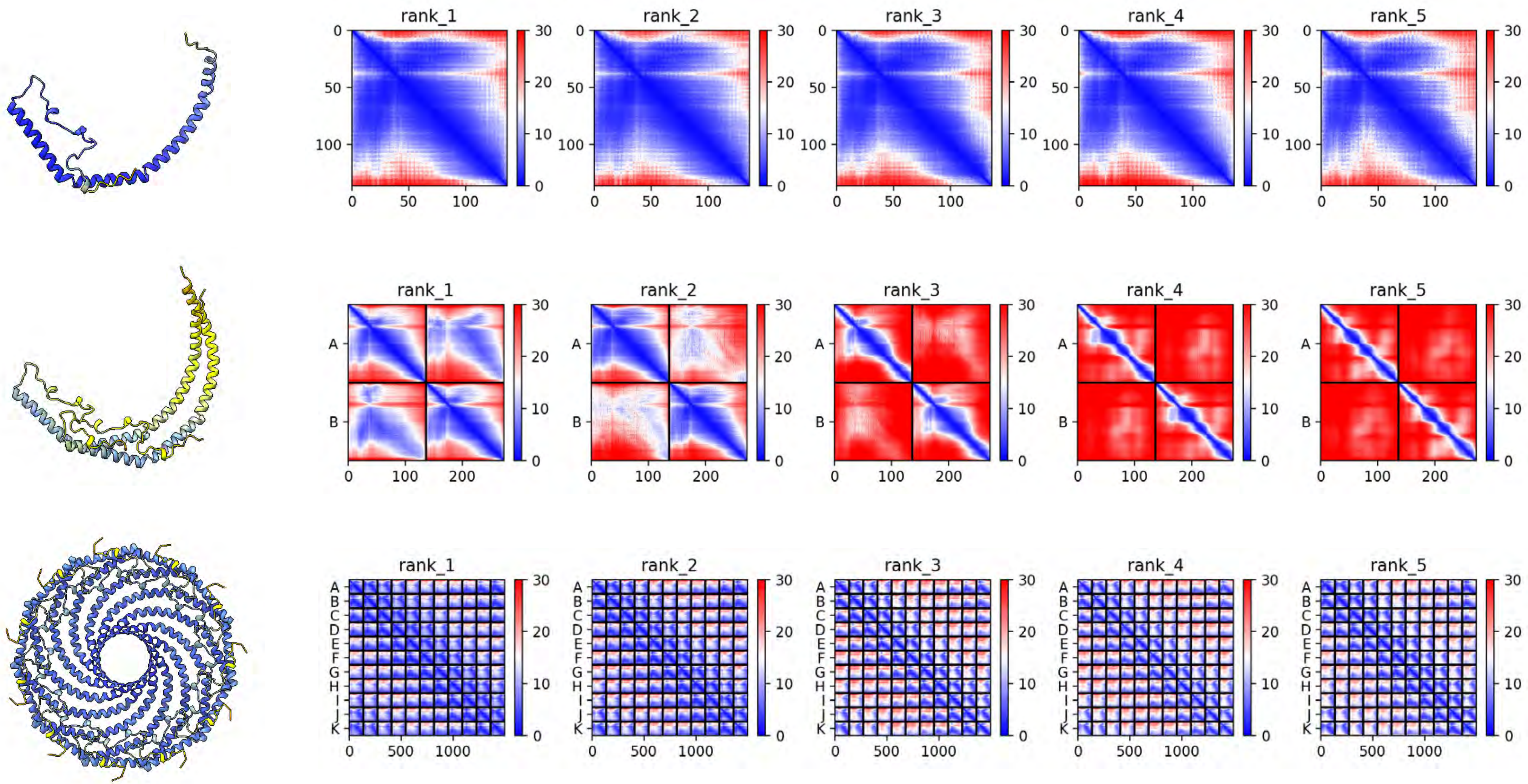

XP_032827408.1 *P. marinus*

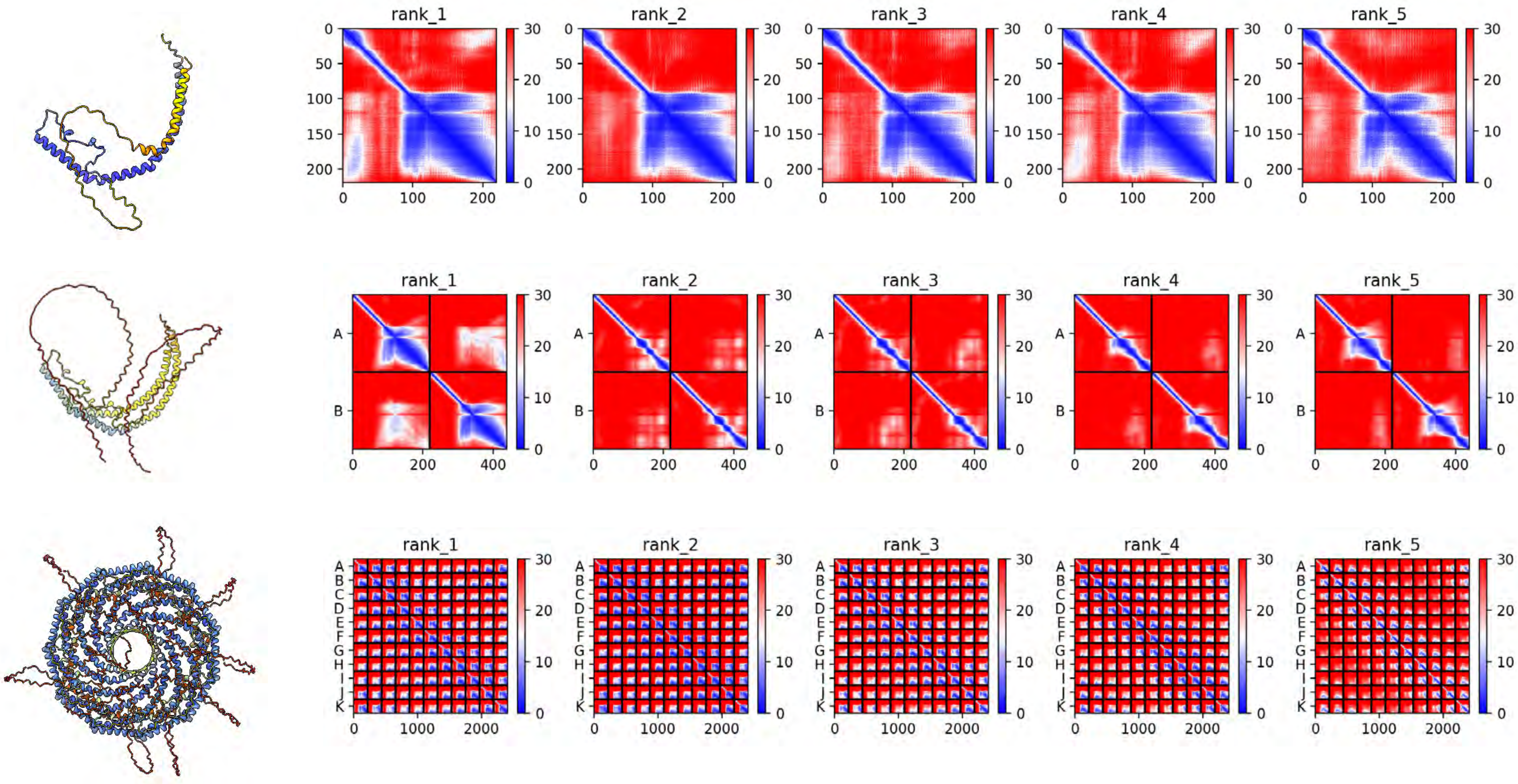

XP_035693613.1 *B. floridae*

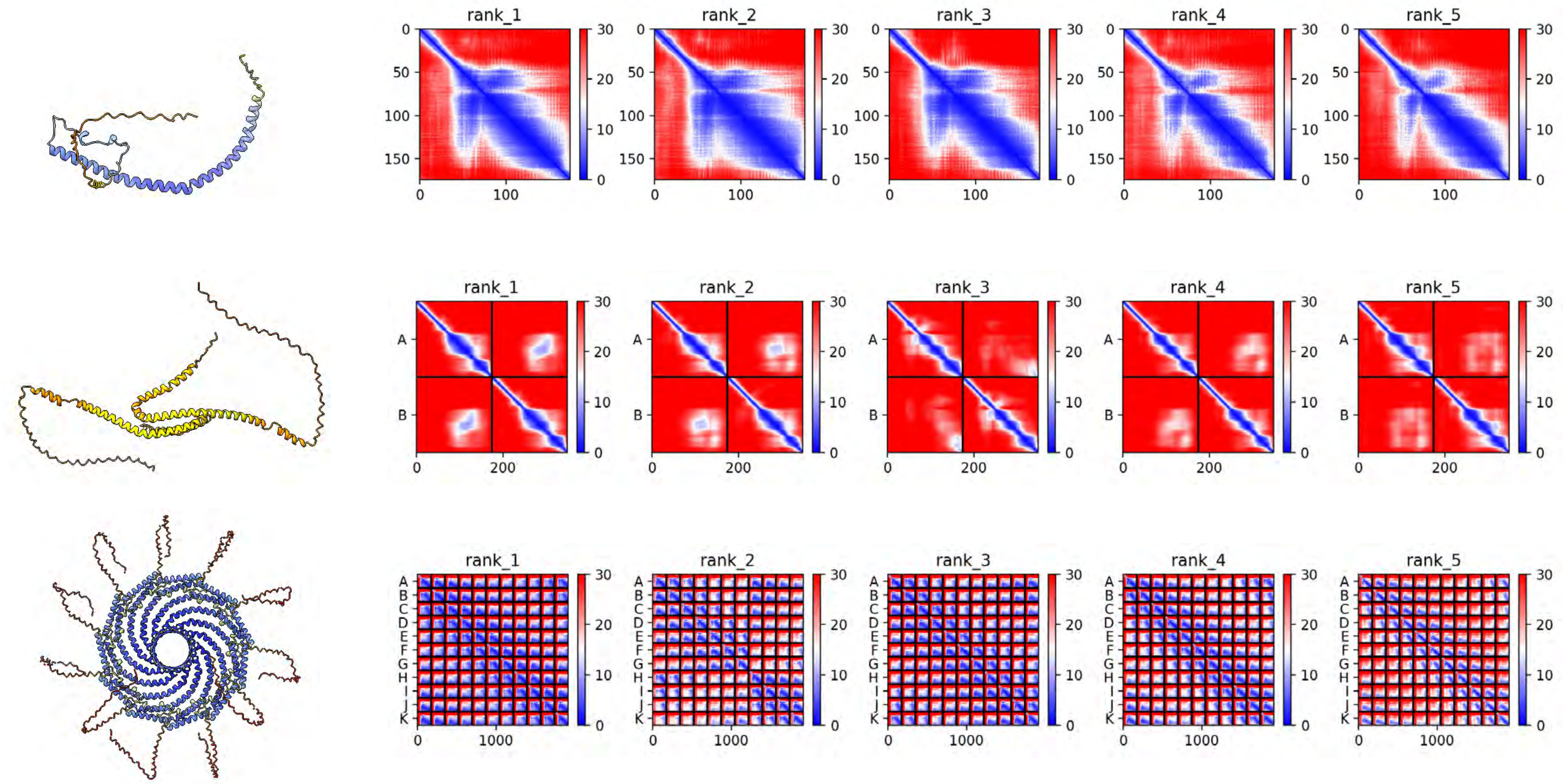

XP_035698192.1 *B. floridae*

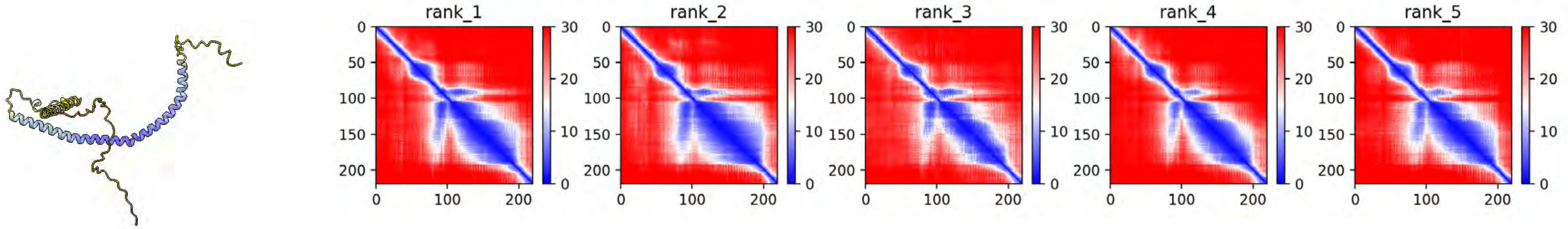

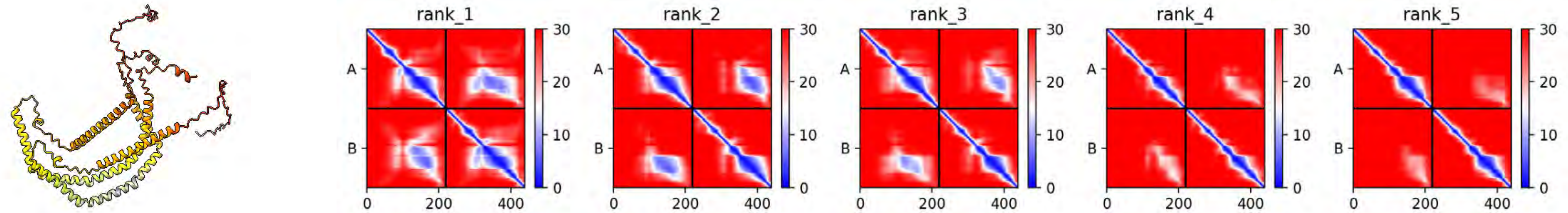

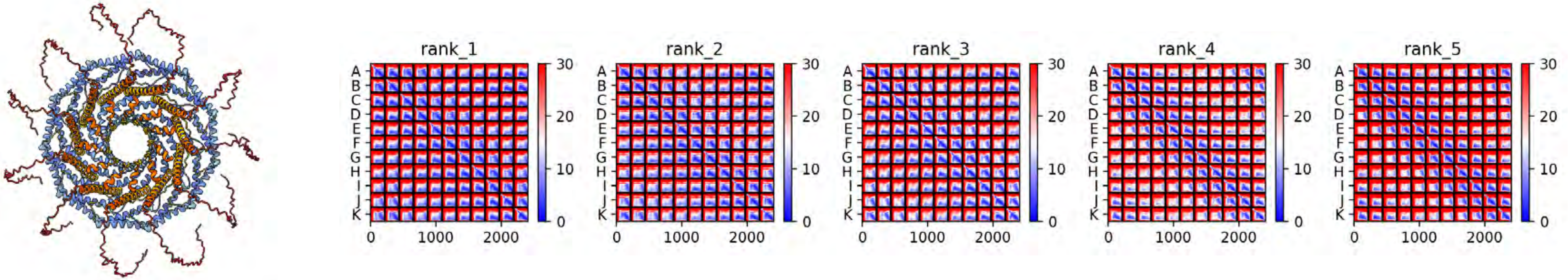

XP_035680487.1 *B. floridae*

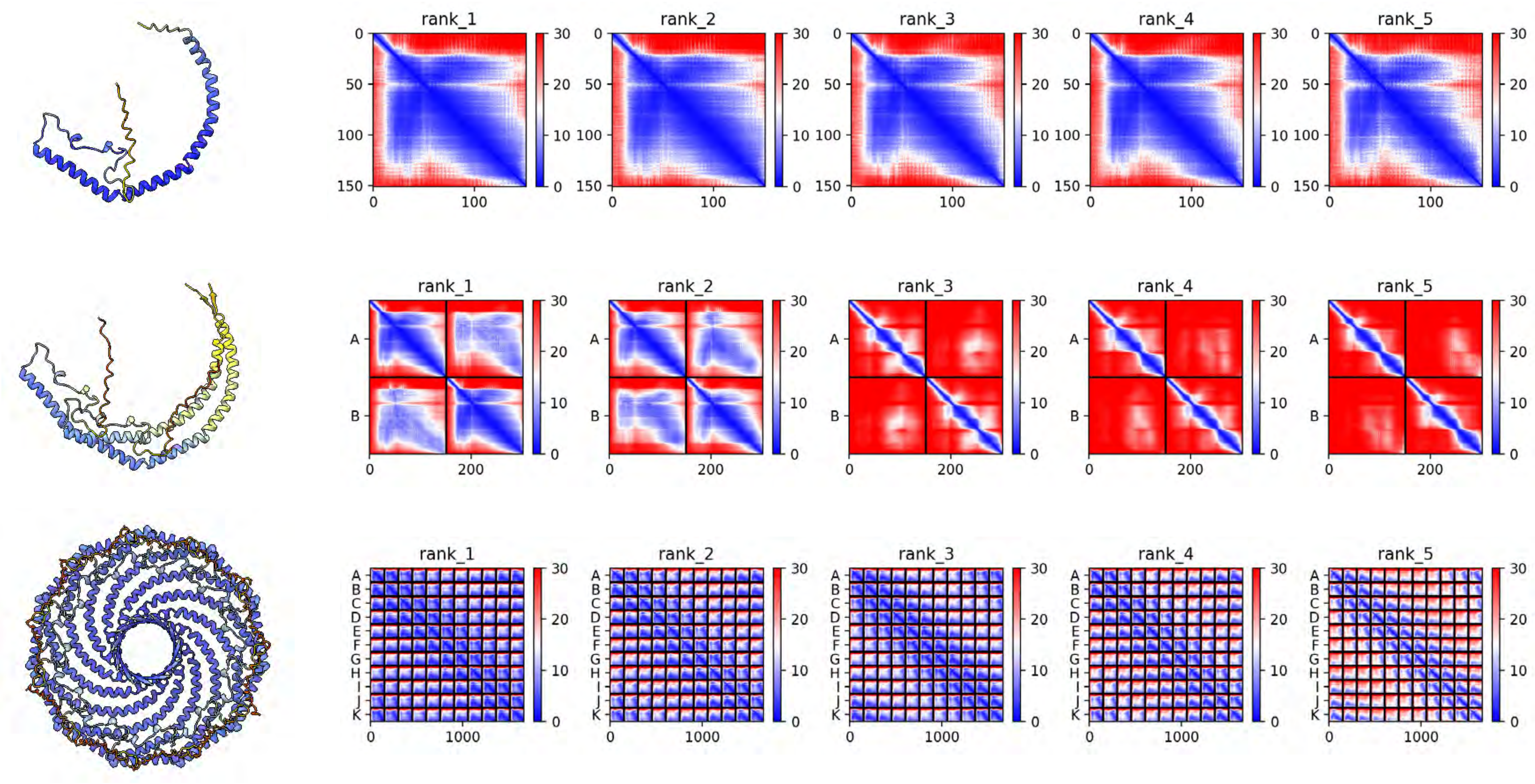

XP_035680488.1 *B. floridae*

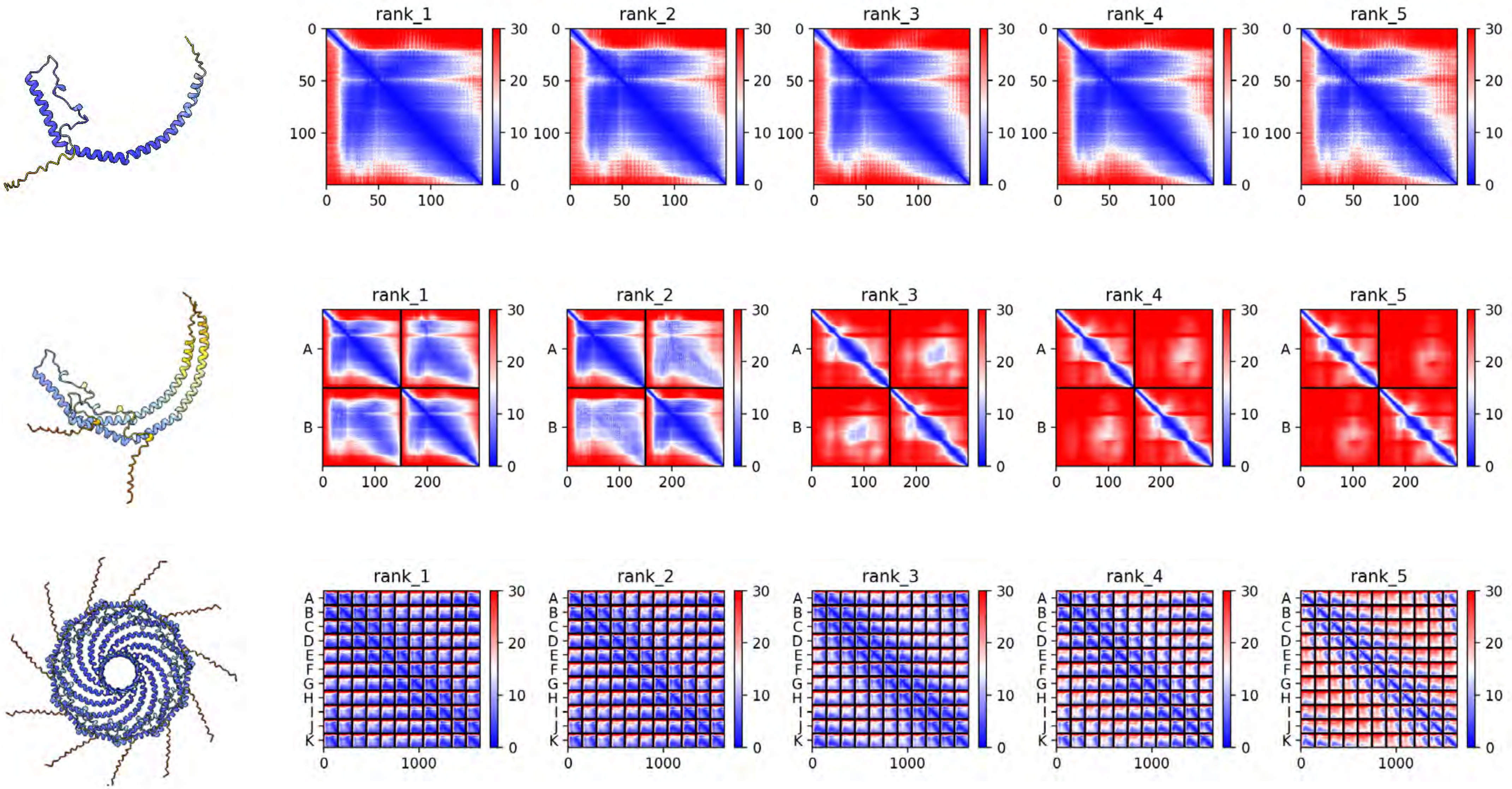

CF919476 *B. floridae*

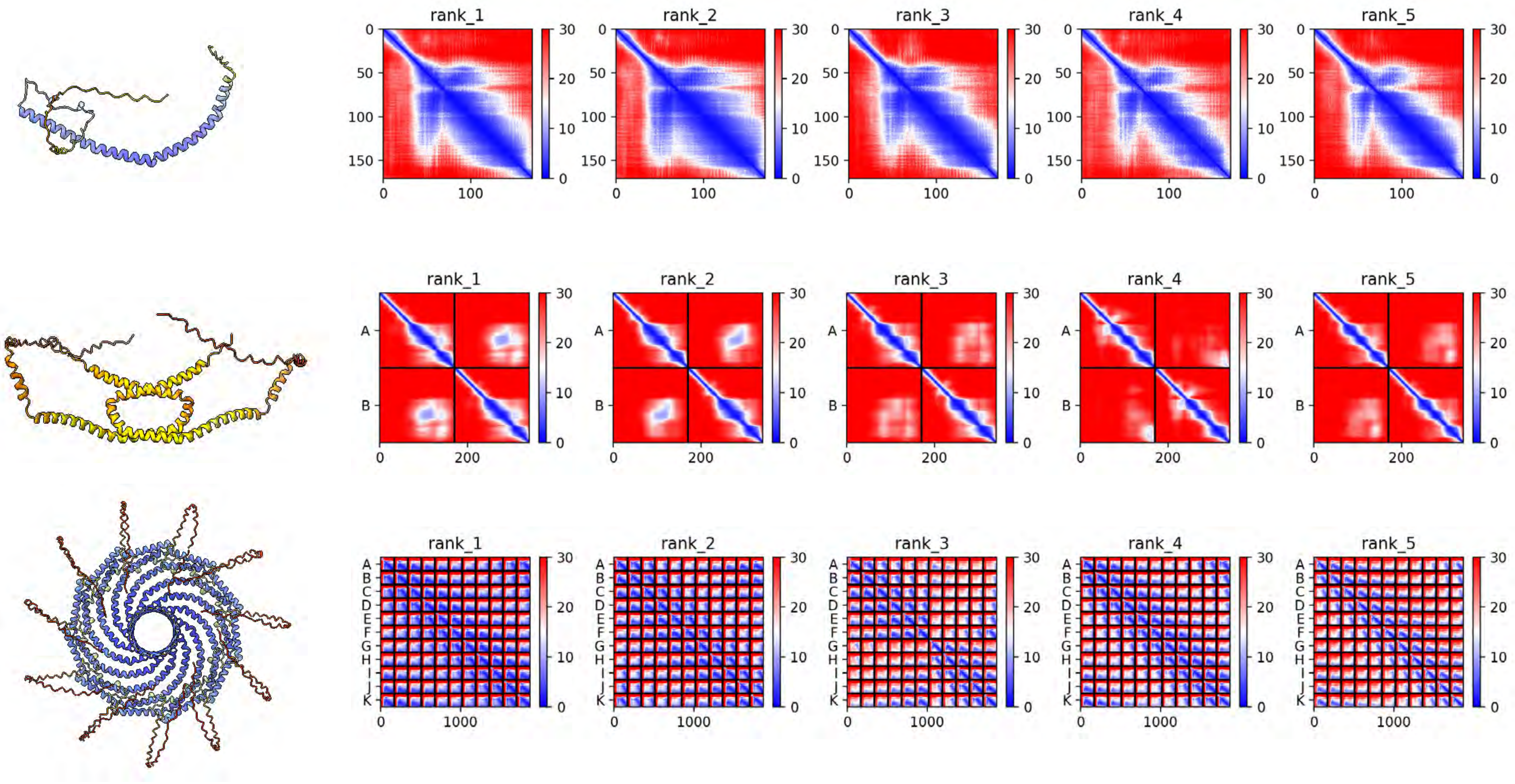

NP_001099134.2 *G. gallus*

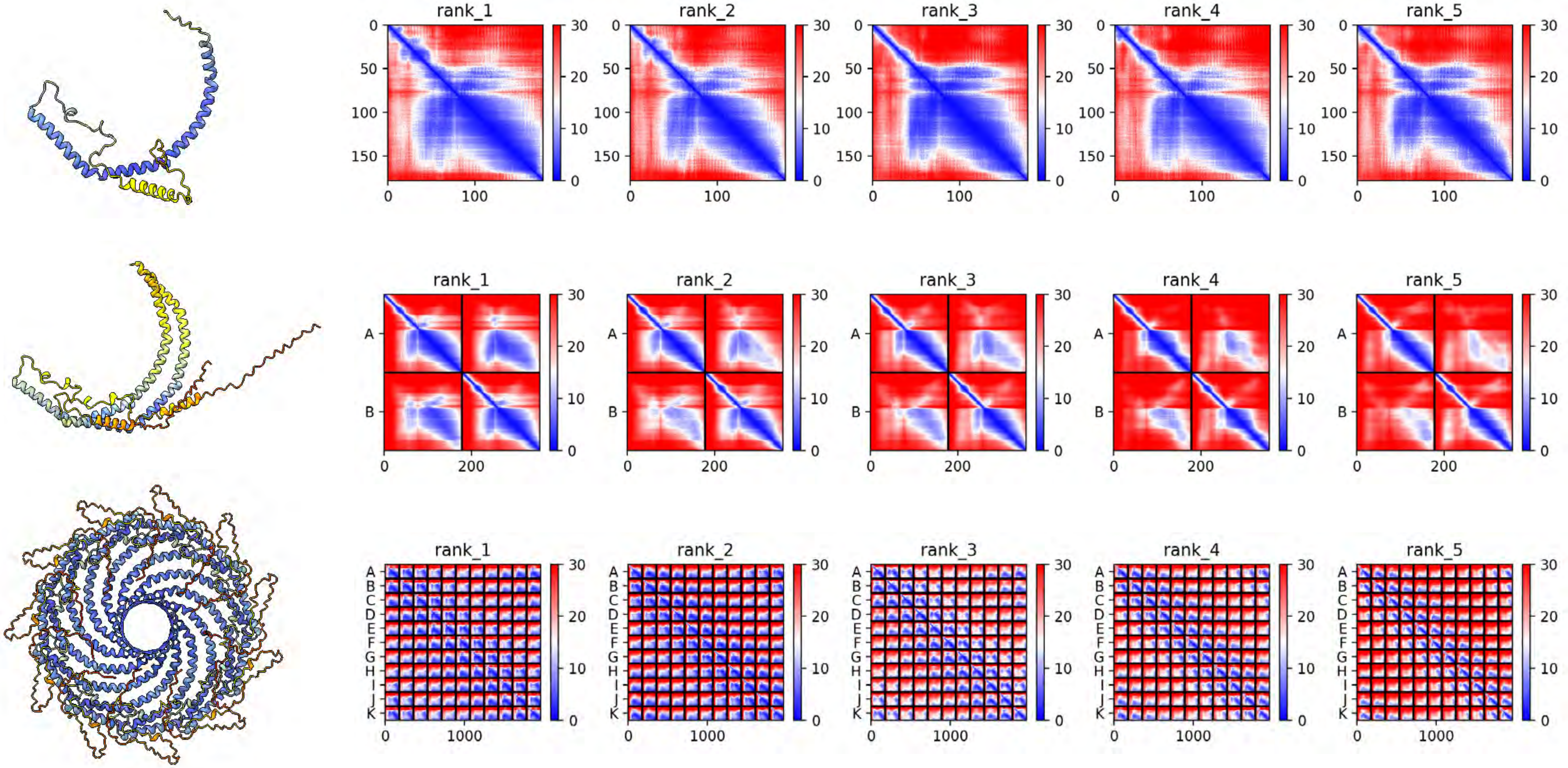

NP_001007087.2 *G. gallus*

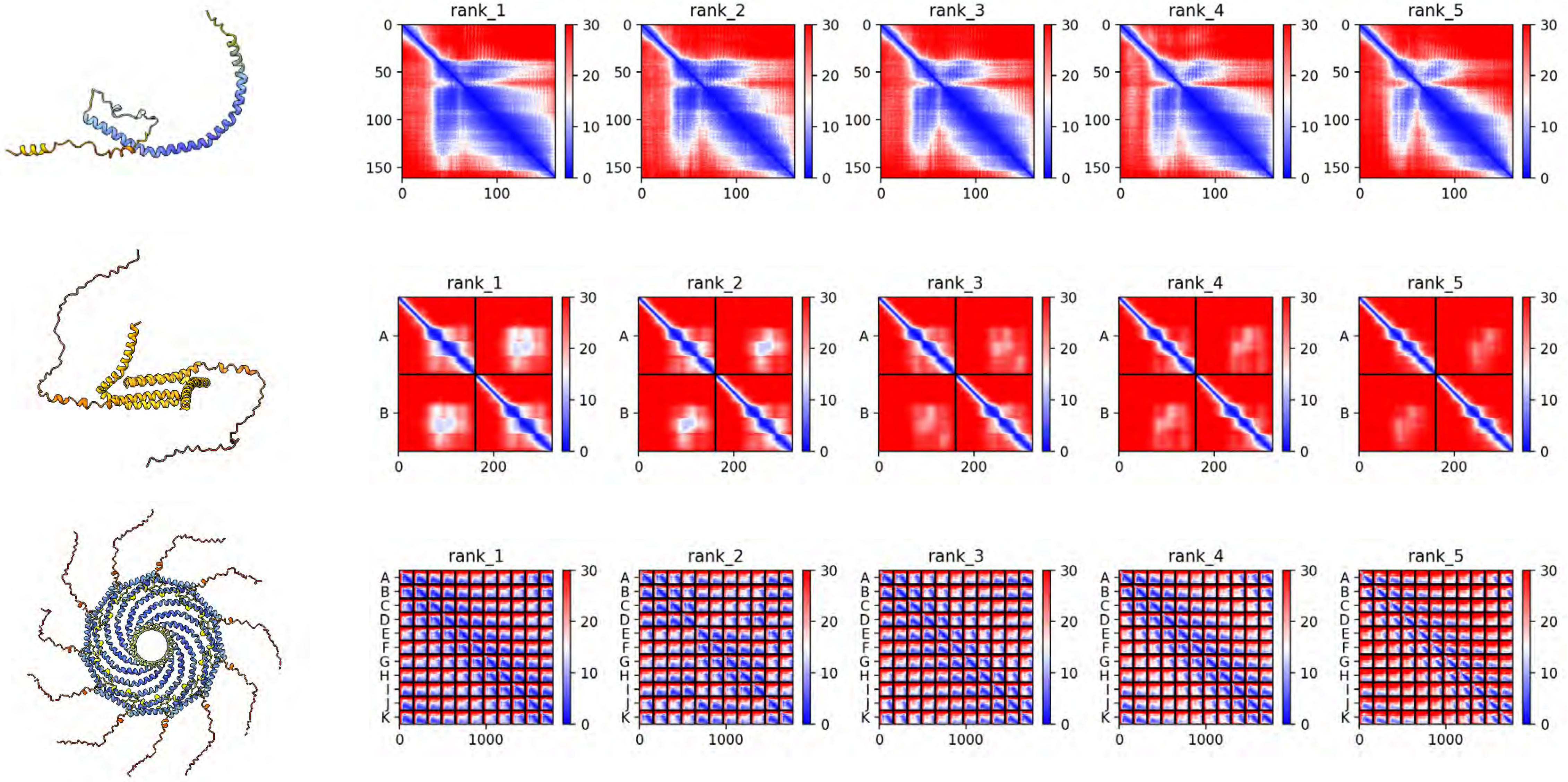

NP_989701.2 *G. gallus*

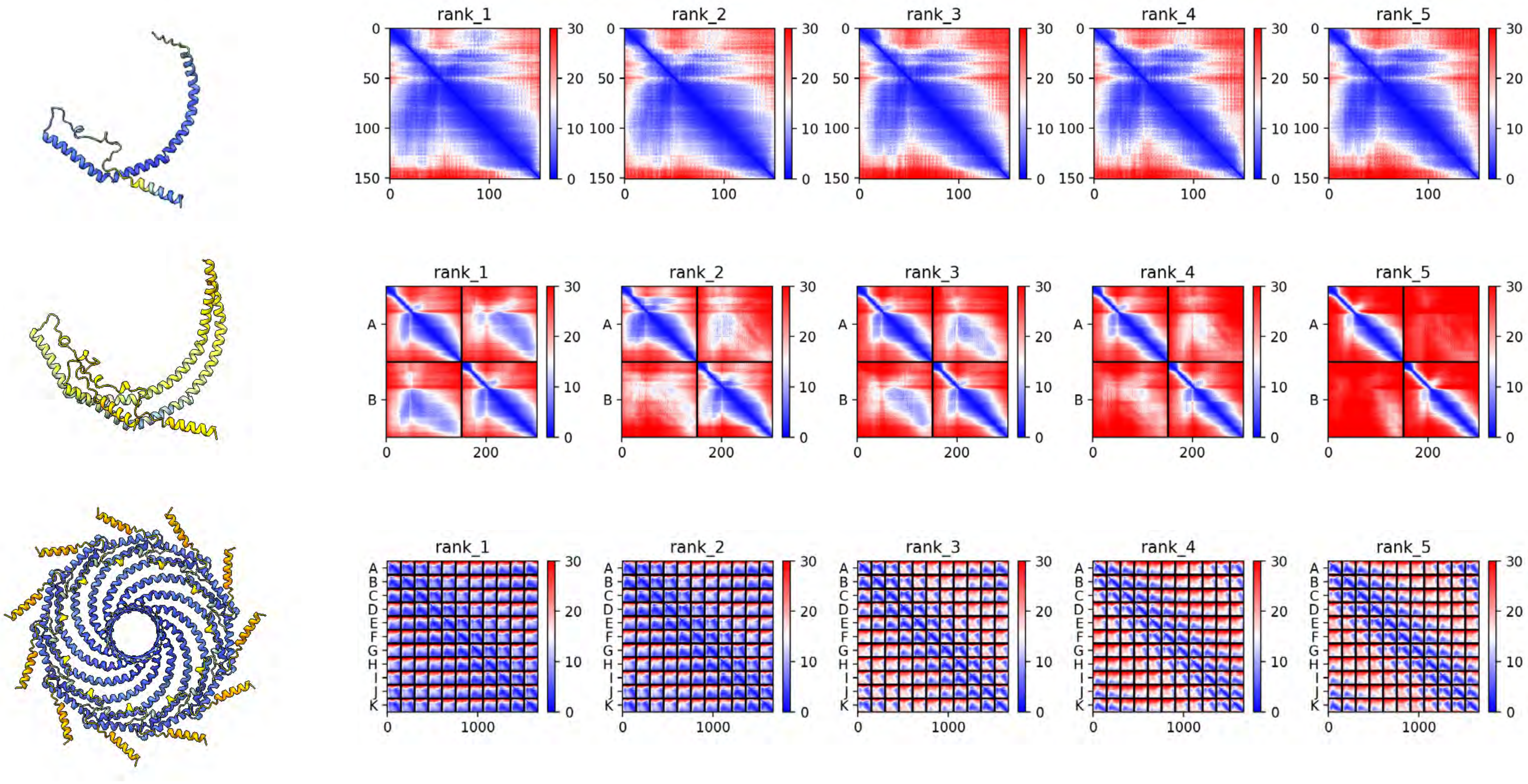

A0A0D2VH37 *C. owczarzaki*

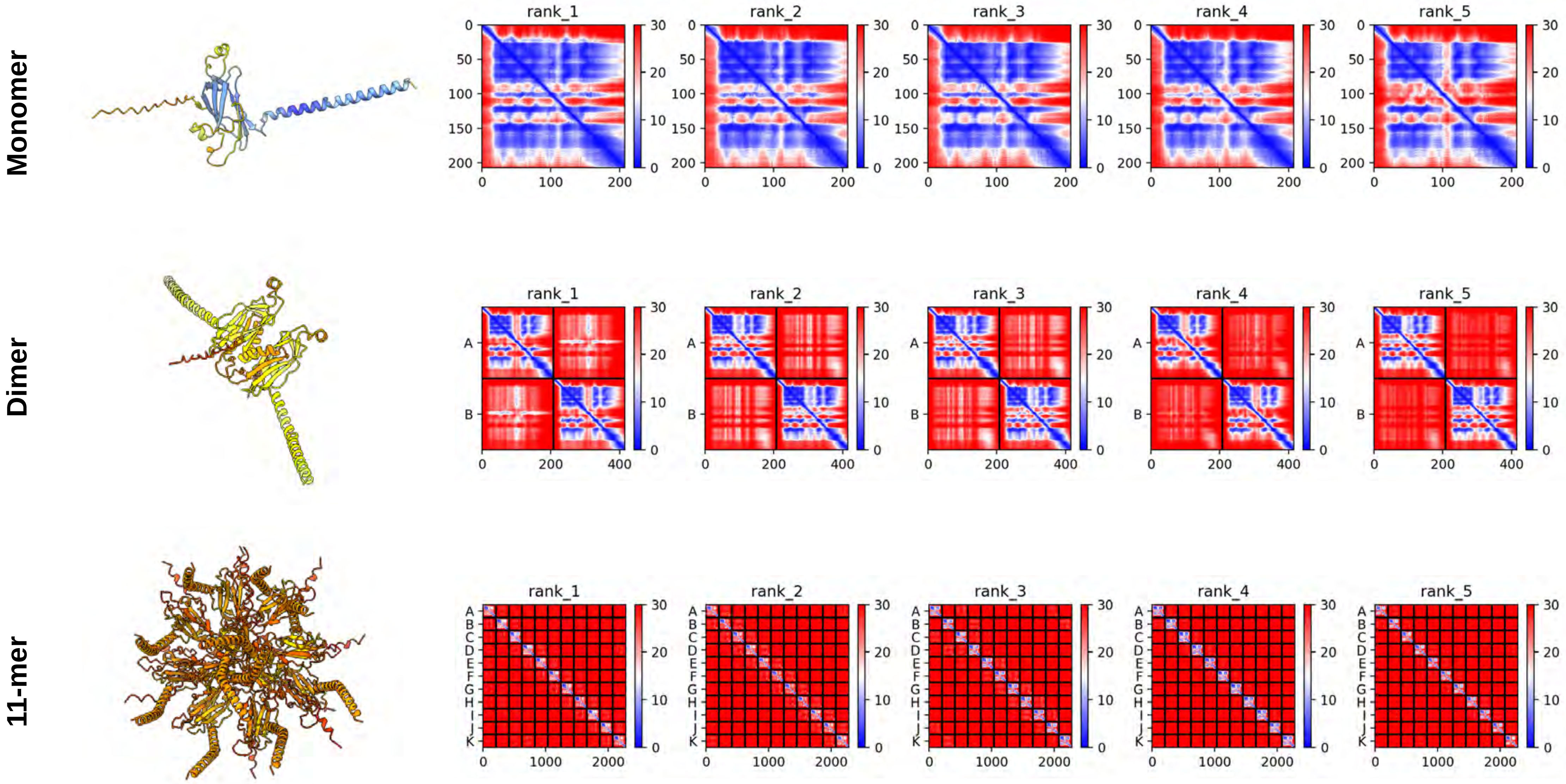

Supplement: Data S4 — shows predicted structures of caveolin monomers and oligomers using AlphaFold2.2. [file jcb_202411175_datas4.pdf]
