# Supplementary material for: Evolutionarily diverse caveolins share a common structural framework built around amphipathic disks
Source: J Cell Biol. 2025 Aug 7;224(9):e202411175. doi: 10.1083/jcb.202411175 (PMC12330381; doi:10.1083/jcb.202411175)

File S5. Predicted structures of select caveolin monomers, dimers, and 11-mers using AlphaFold3.

Note:

Models were all colored by pLDDT values as color key indicated in each page.

The pTM/ipTM values were listed under pLDDT color key.

The Expected Position Error plots were displayed on the right side of each page, next to the predicted model.

F2U793 *S. Rosetta*

Monomer

Very high (pIDDT > 90) | Confident (90 > pIDDT > 70) | Low (70 > pIDDT > 50) | Very low (pIDDT < 50)

ipTM = - pTM = 0.27 learn more

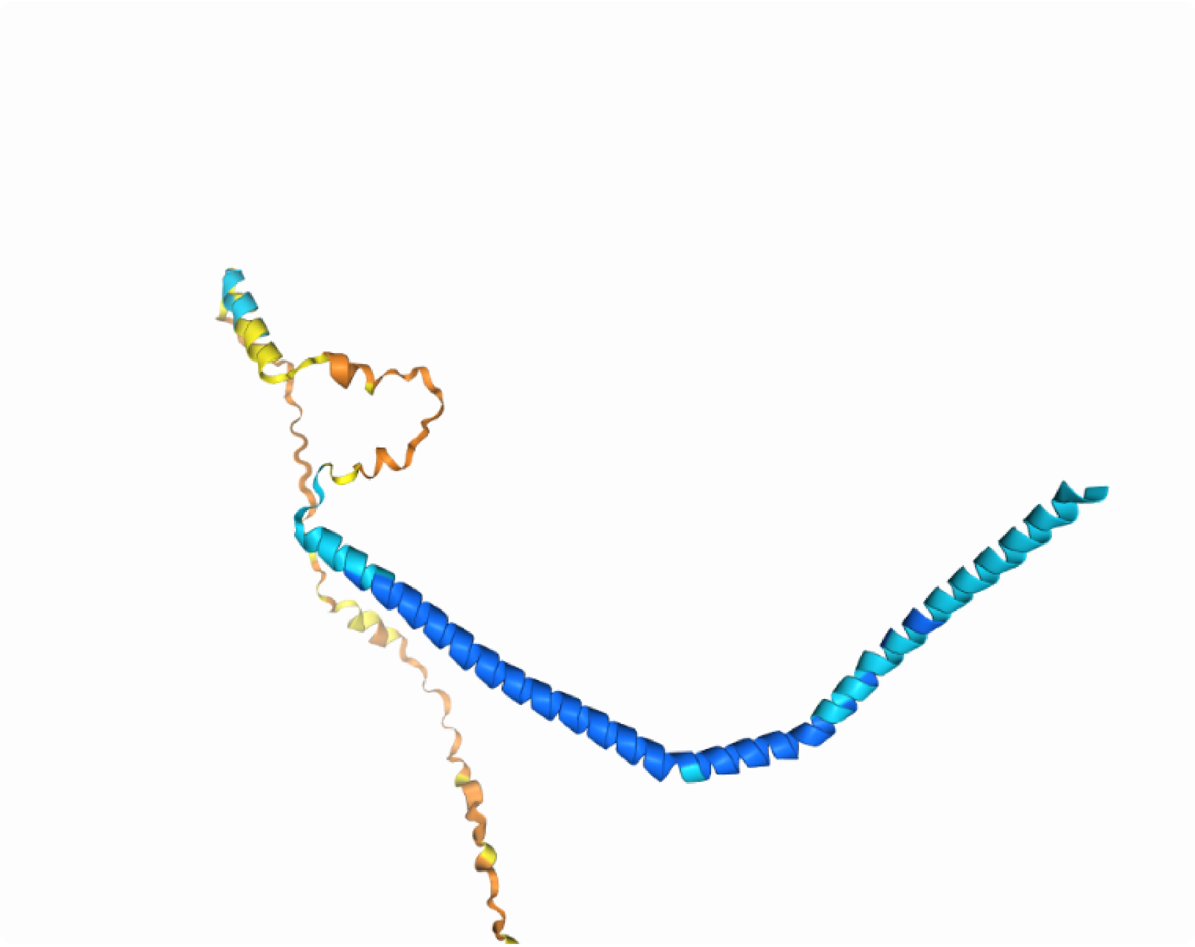

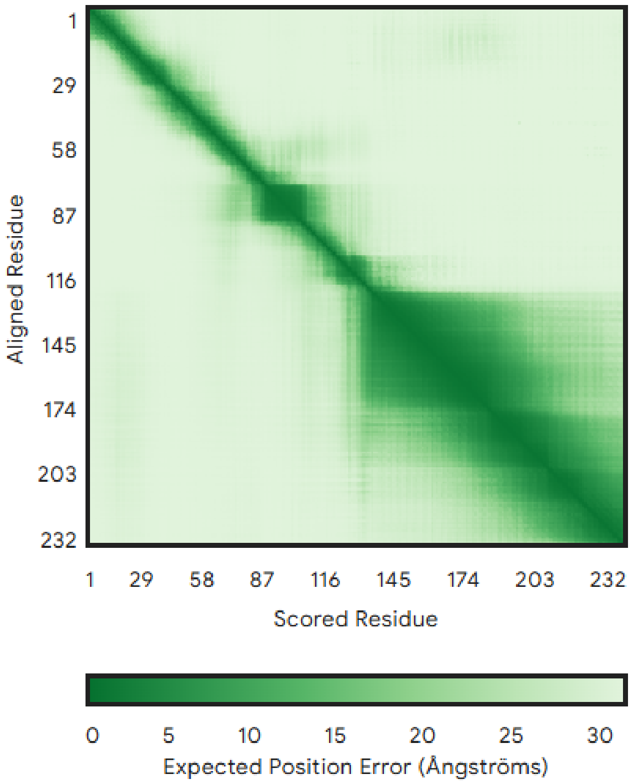

F2U793 *S. Rosetta*

Dimer

Very high (pLDDT > 90) | Confident (90 > pLDDT > 70) | Low (70 > pLDDT > 50) | Very low (pLDDT < 50)

ipTM = 0.14 pTM = 0.21 learn more

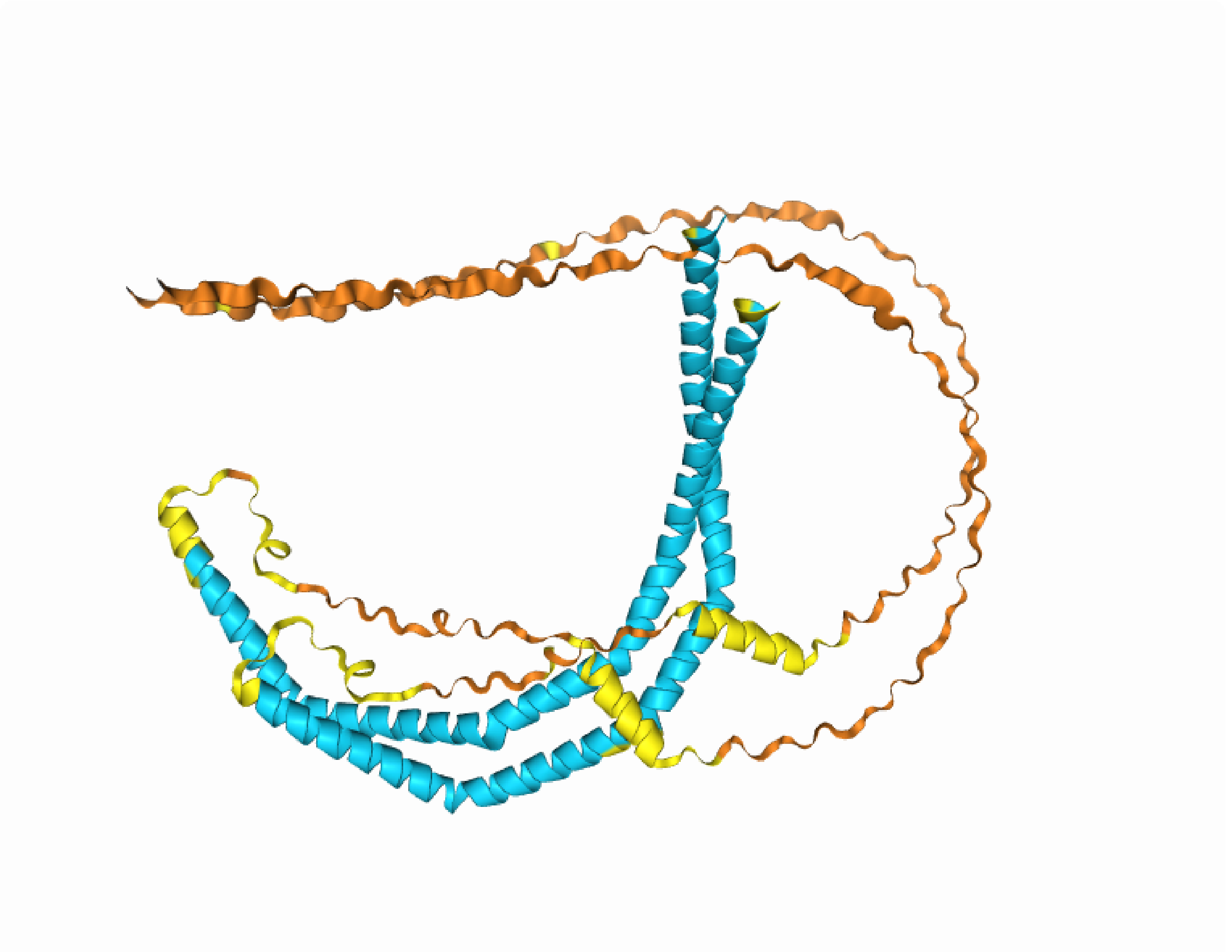

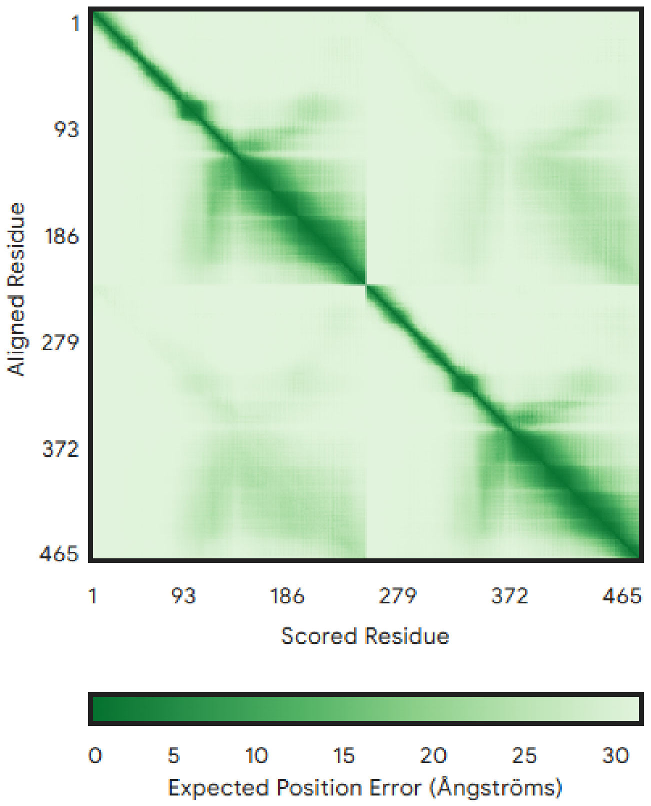

F2U793 *S. Rosetta*

11-mer

Very high (pIDDT > 90) | Confident (90 > pIDDT > 70) | Low (70 > pIDDT > 50) | Very low (pIDDT < 50)

ipTM = 0.56 pTM = 0.57 learn more

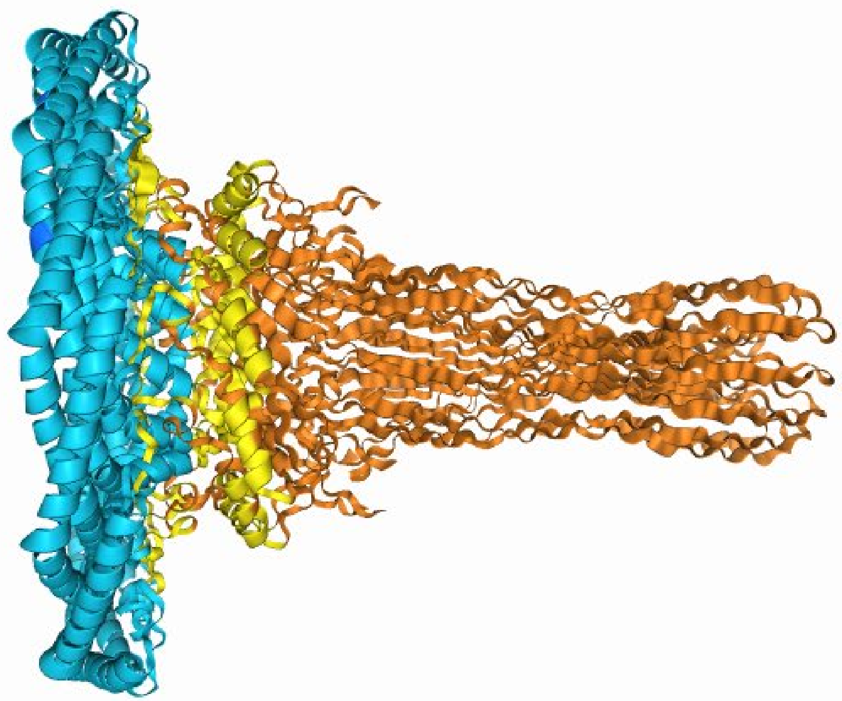

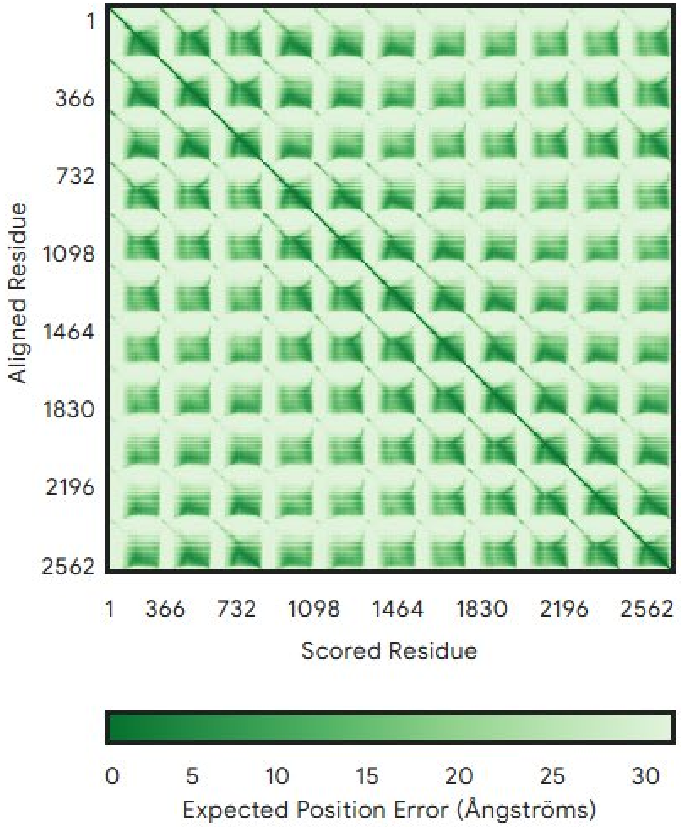

A0A1X7UHP5 *A. queenslandica*

Monomer

Very high (plDDT > 90) | Confident (90 > pIDDT > 70) | Low (70 > pIDDT > 50) | Very low (pIDDT < 50)

ipTM = - pTM = 0.27 learn more

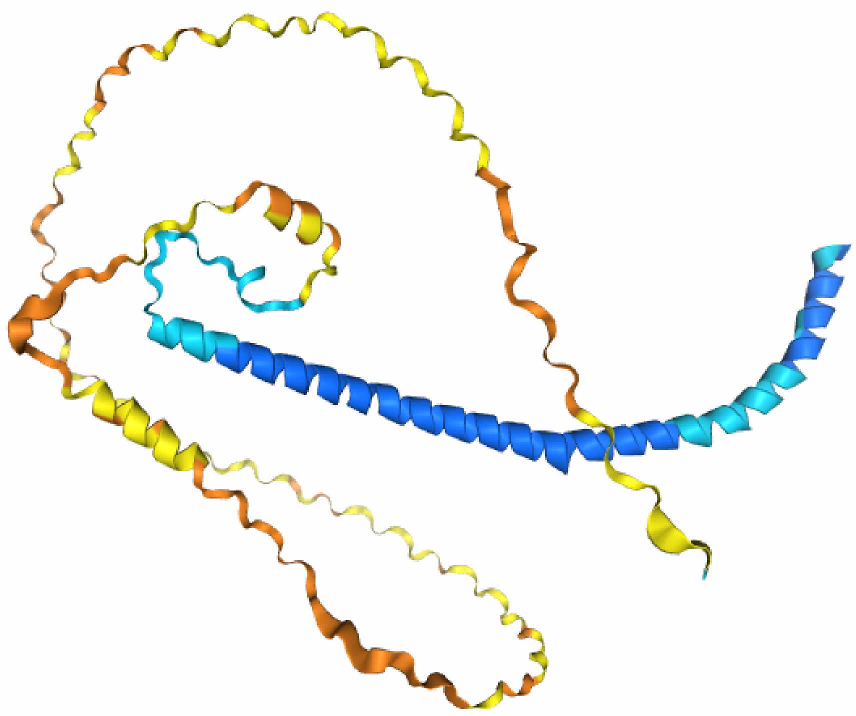

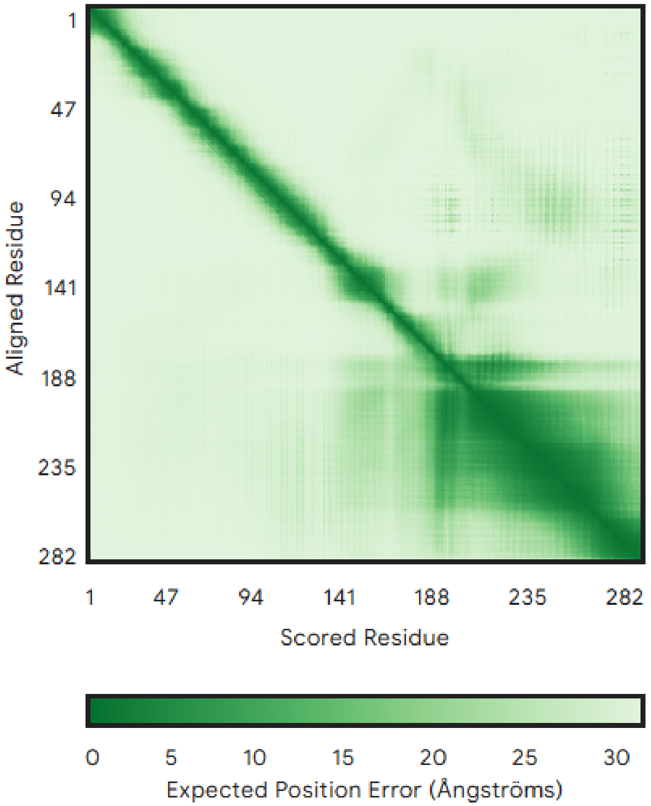

A0A1X7UHP5 *A. queenslandica*

Dimer

Very high (plDDT > 90) | Confident (90 > plDDT > 70) | Low (70 > plDDT > 50) | Very low (plDDT < 50)

ipTM = 0.08 pTM = 0.16 learn more

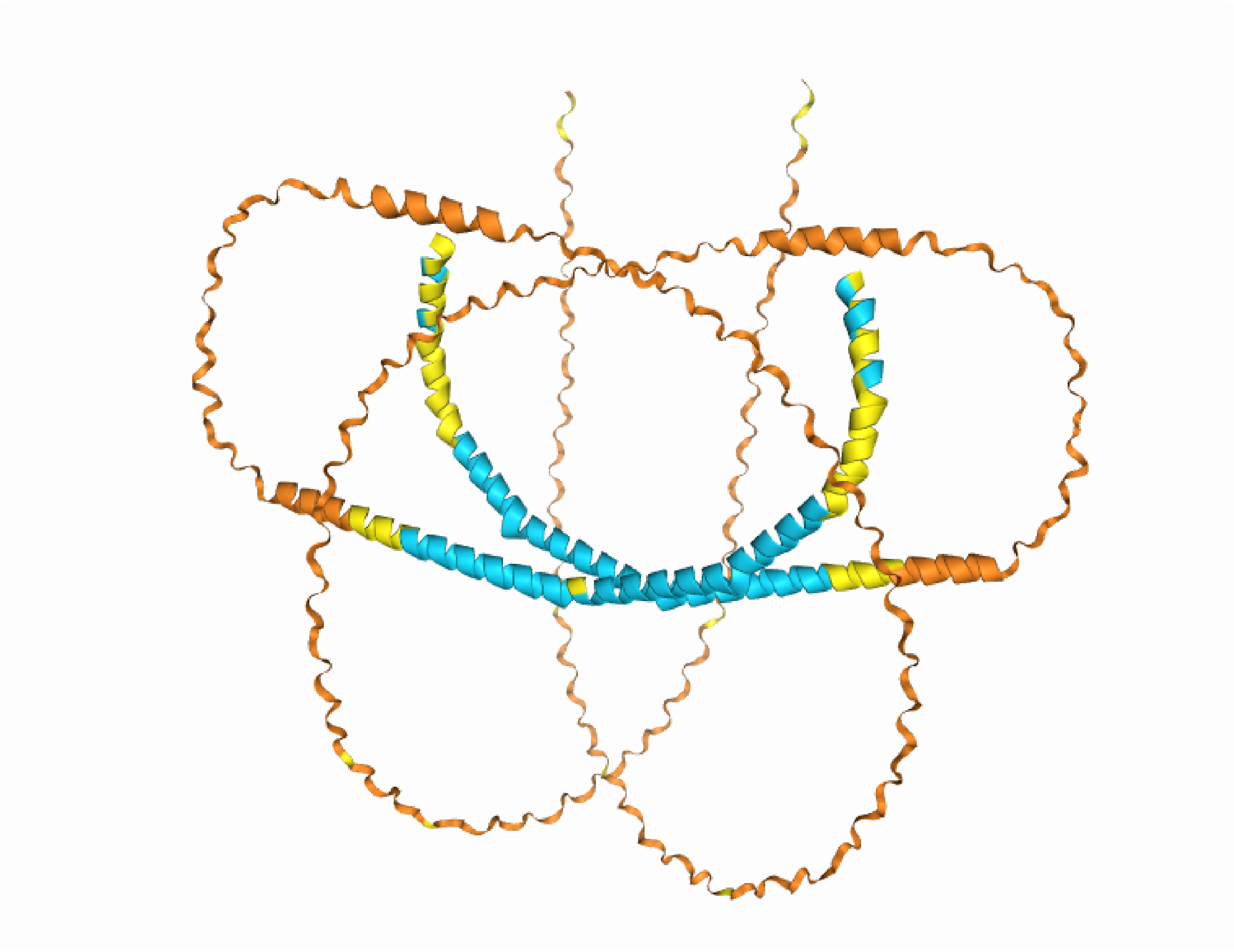

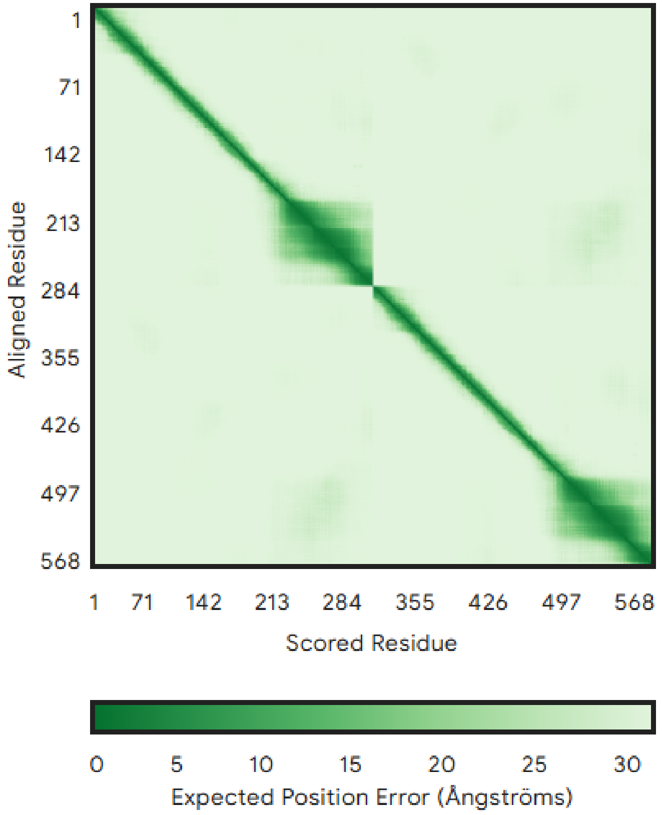

A0A1X7UHP5 ***A. queenslandica***

11-mer

Very high (pIDDT > 90) | Confident (90 > pIDDT > 70) | Low (70 > pIDDT > 50) | Very low (pIDDT < 50)

ipTM = 0.39 pTM = 0.4 learn more

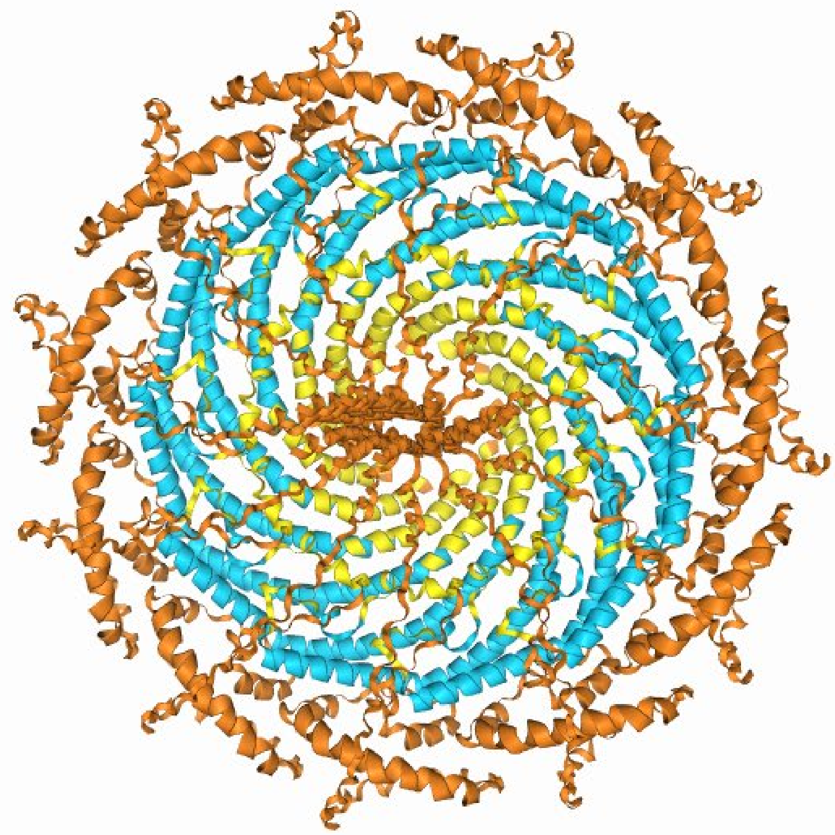

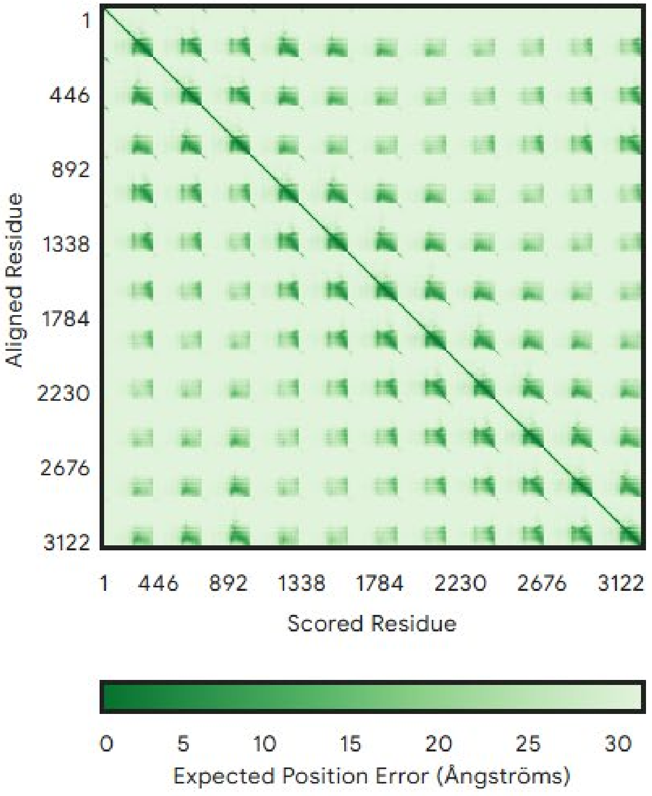

B3RWV2 *T. adhaerens*

Monomer

Very high (pIDDT > 90) | Confident (90 > pIDDT > 70) | Low (70 > pIDDT > 50) | Very low (pIDDT < 50)

ipTM = - pTM = 0.31 learn more

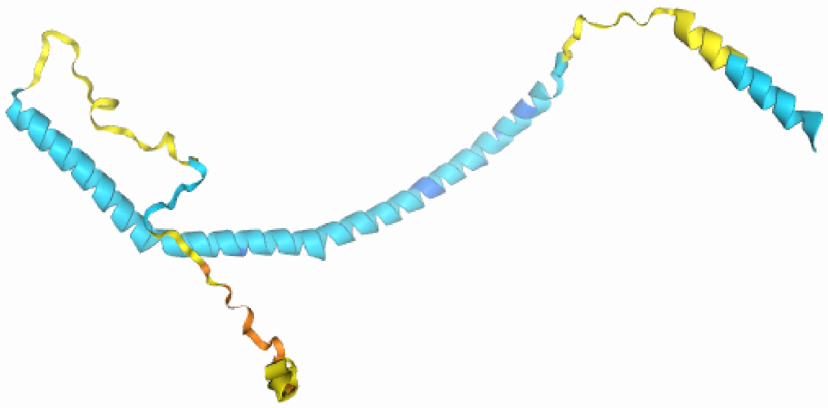

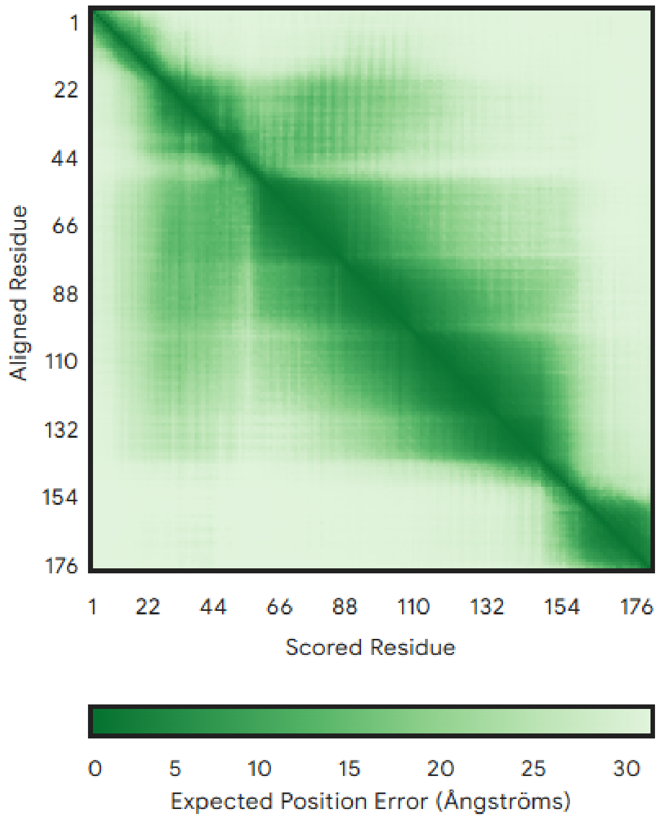

B3RWV2 *T. adhaerens*

Dimer

Very high (pLDDT > 90) | Confident (90 > pLDDT > 70) | Low (70 > pLDDT > 50) | Very low (pLDDT < 50)

ipTM = 0.07 pTM = 0.17 learn more

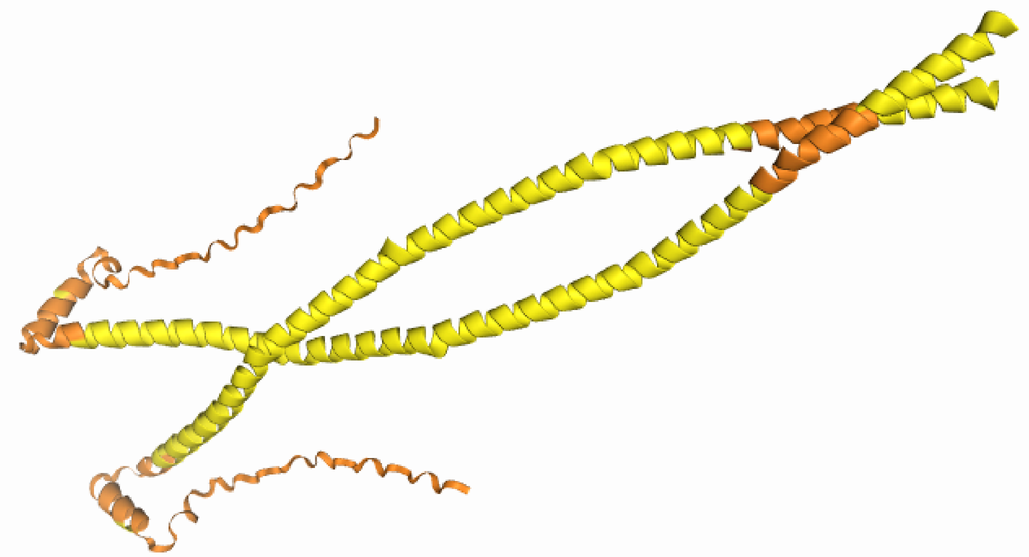

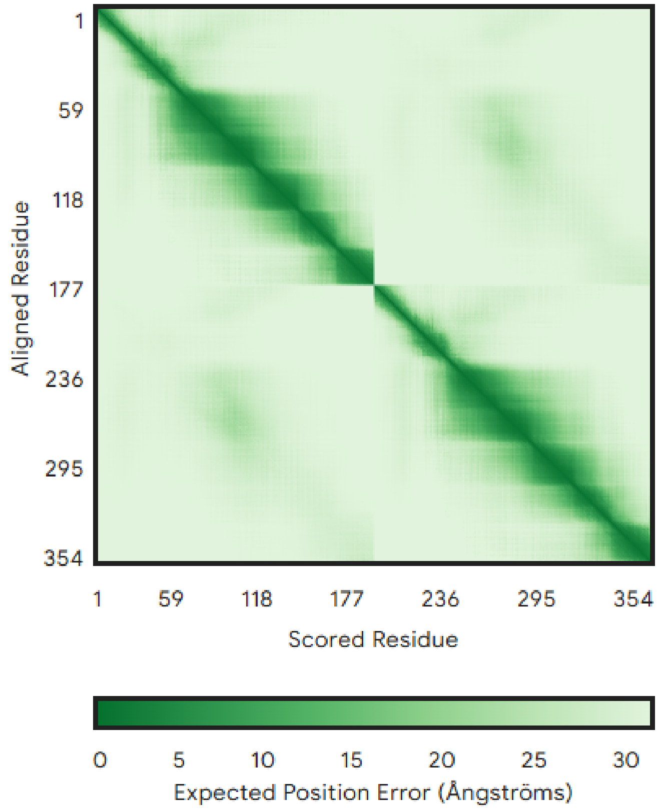

B3RWV2 *T. adhaerens*

11-mer

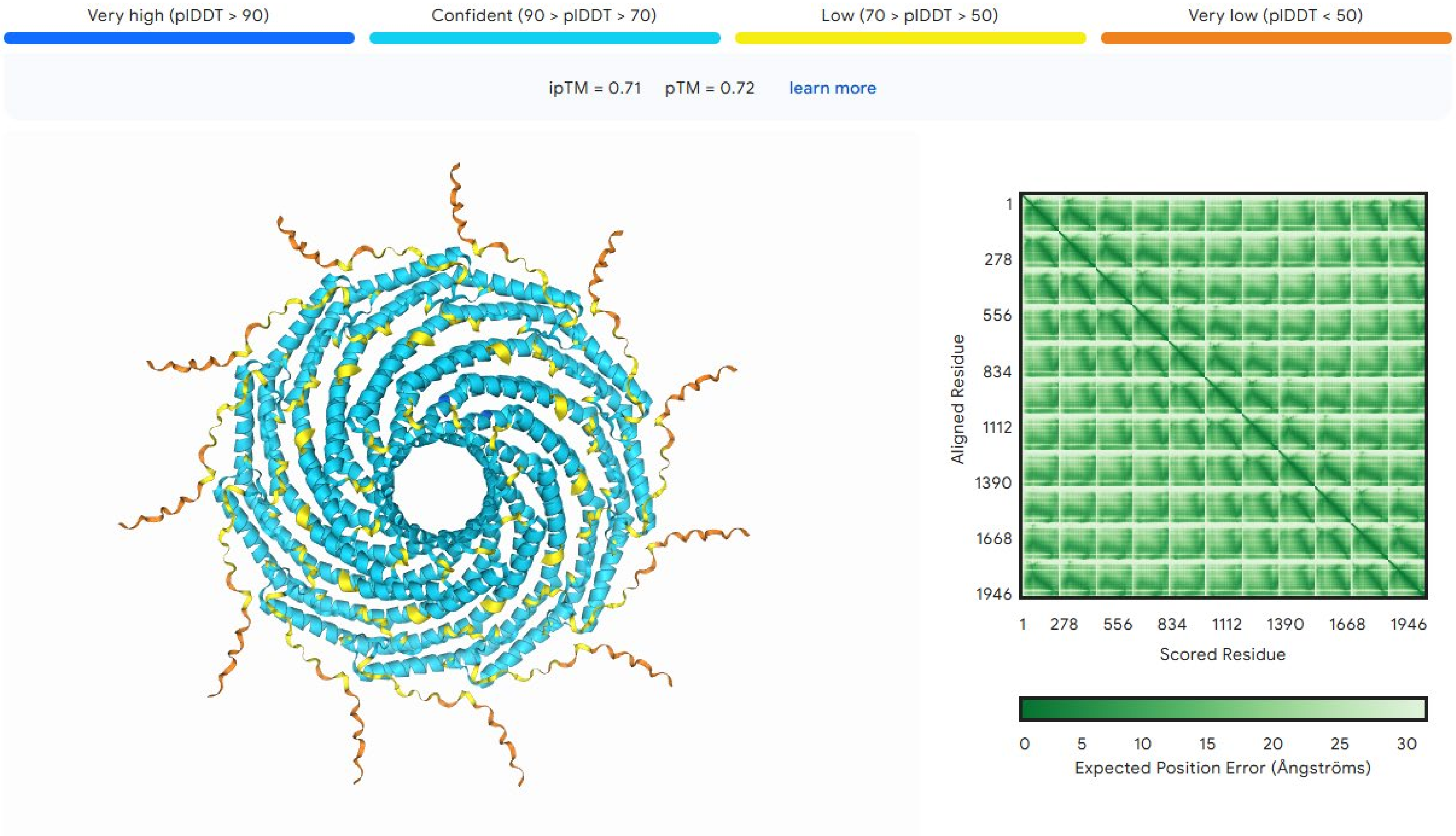

A0A2B4SAZ9 *S. pistillata*

Monomer

Very high (plDDT > 90) | Confident (90 > pIDDT > 70) | Low (70 > pIDDT > 50) | Very low (pIDDT < 50)

ipTM = - pTM = 0.36 learn more

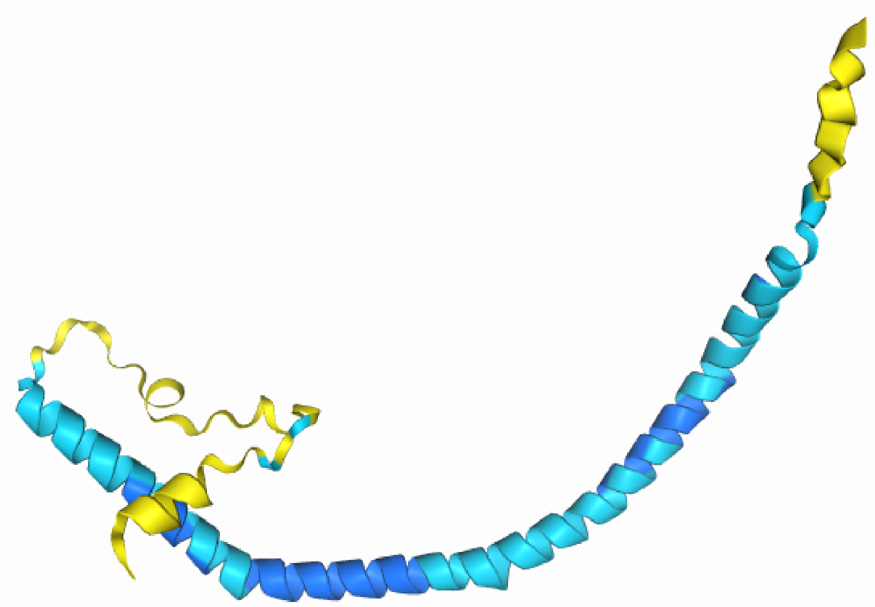

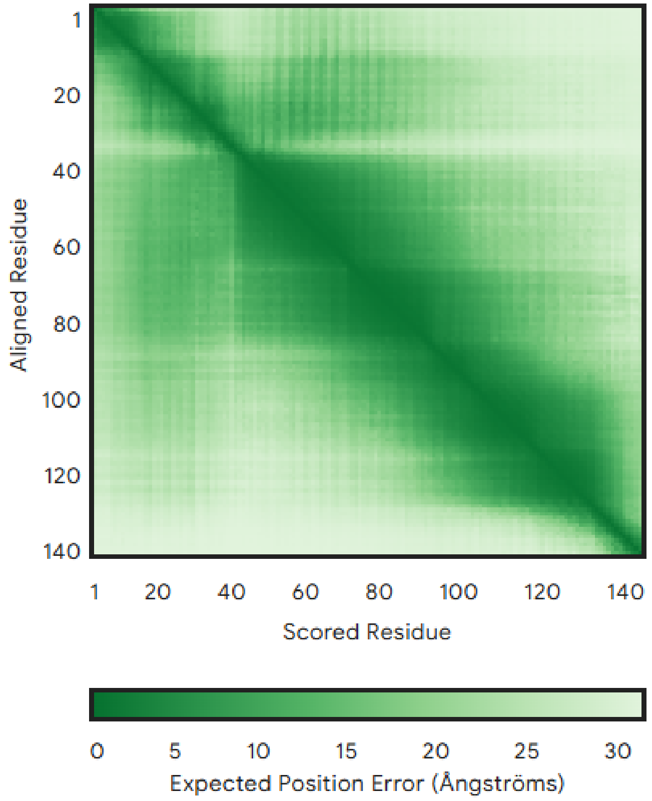

A0A2B4SAZ9 *S. pistillata*

Dimer

Very high (pIDDT > 90) | Confident (90 > pIDDT > 70) | Low (70 > pIDDT > 50) | Very low (pIDDT < 50)

ipTM = 0.1 pTM = 0.22 learn more

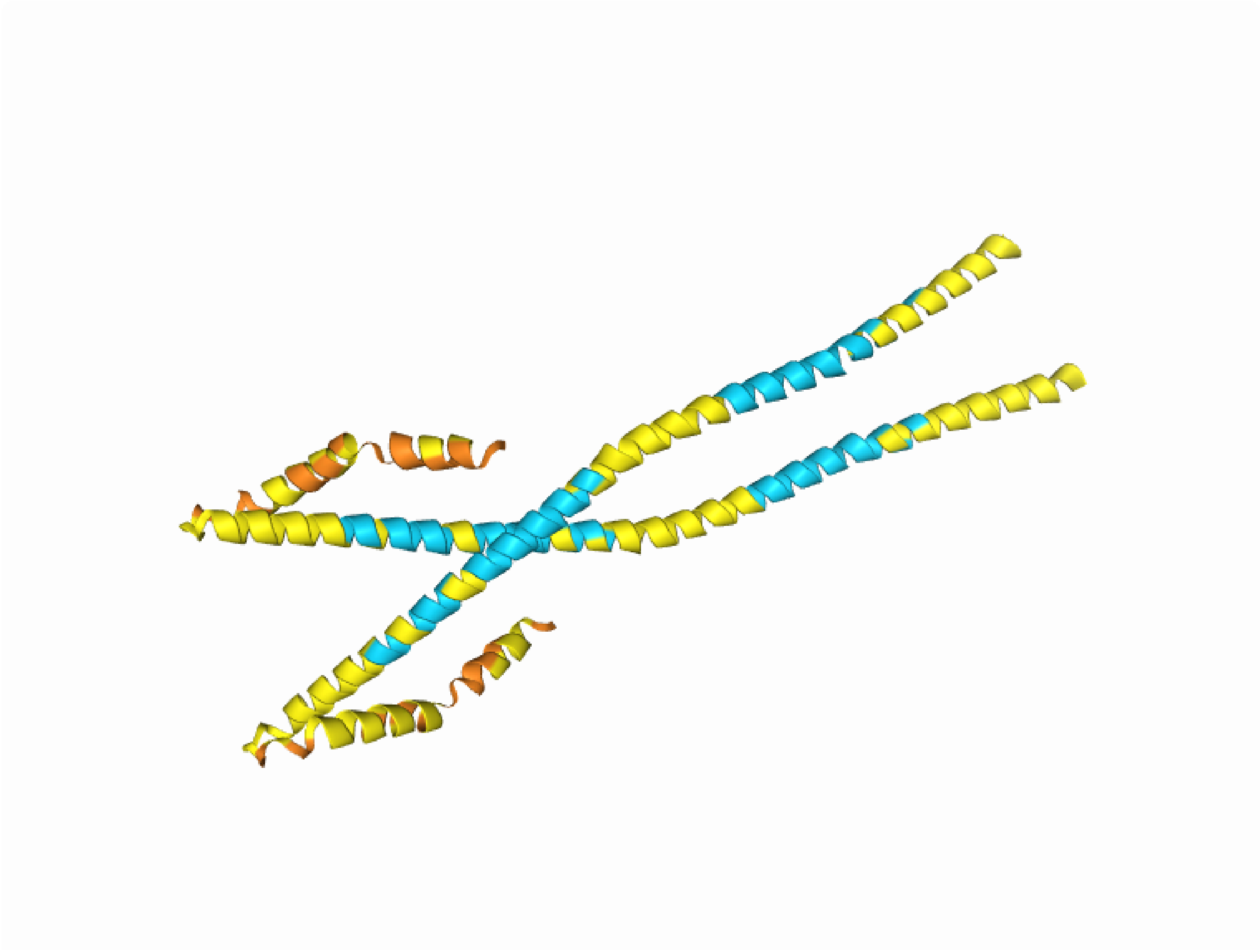

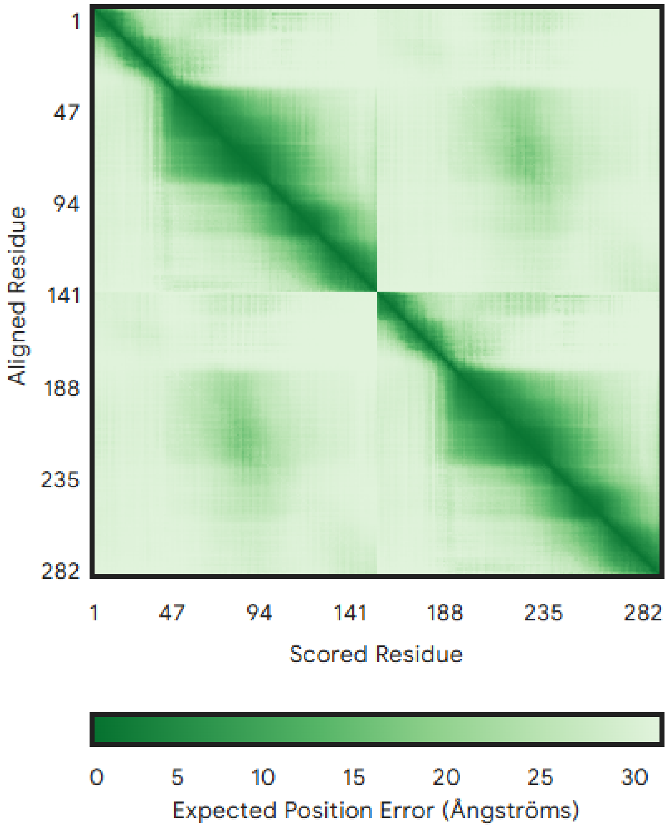

A0A2B4SAZ9 *S. pistillata*

11-mer

Very high (pIDDT > 90) | Confident (90 > pIDDT > 70) | Low (70 > pIDDT > 50) | Very low (pIDDT < 50)

ipTM = 0.63 pTM = 0.64 learn more

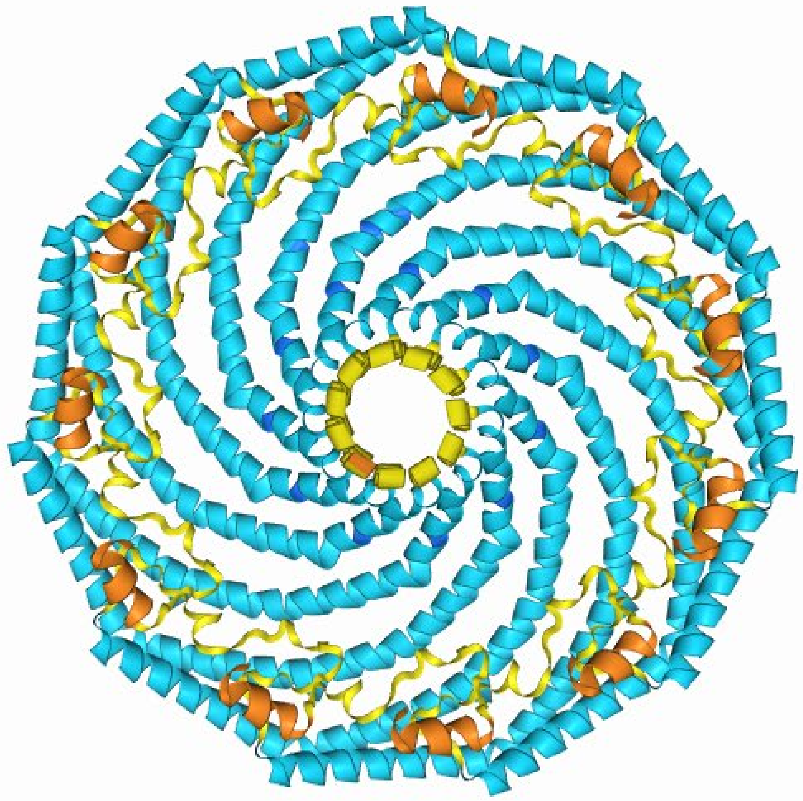

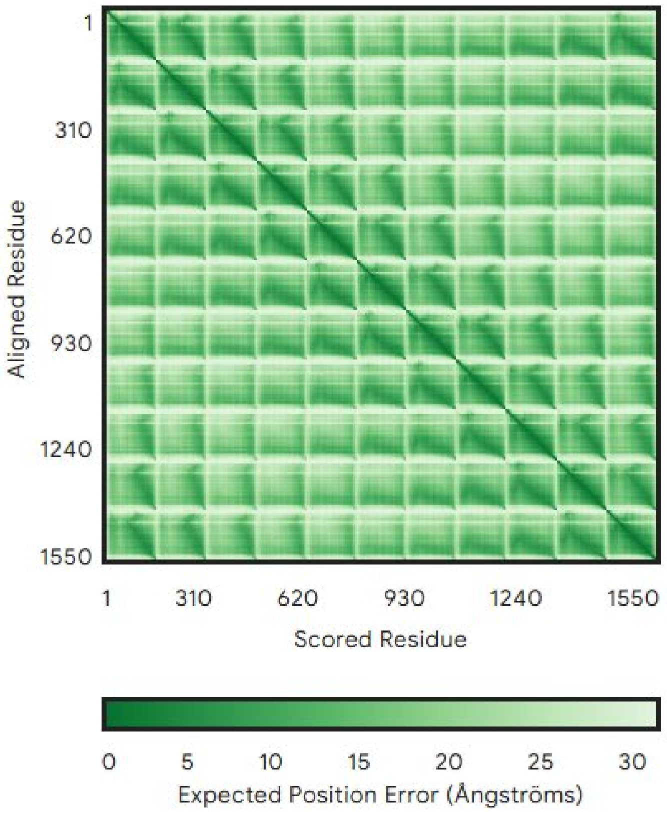

scaffold217_2 *Xenoturbella bocki*

Monomer

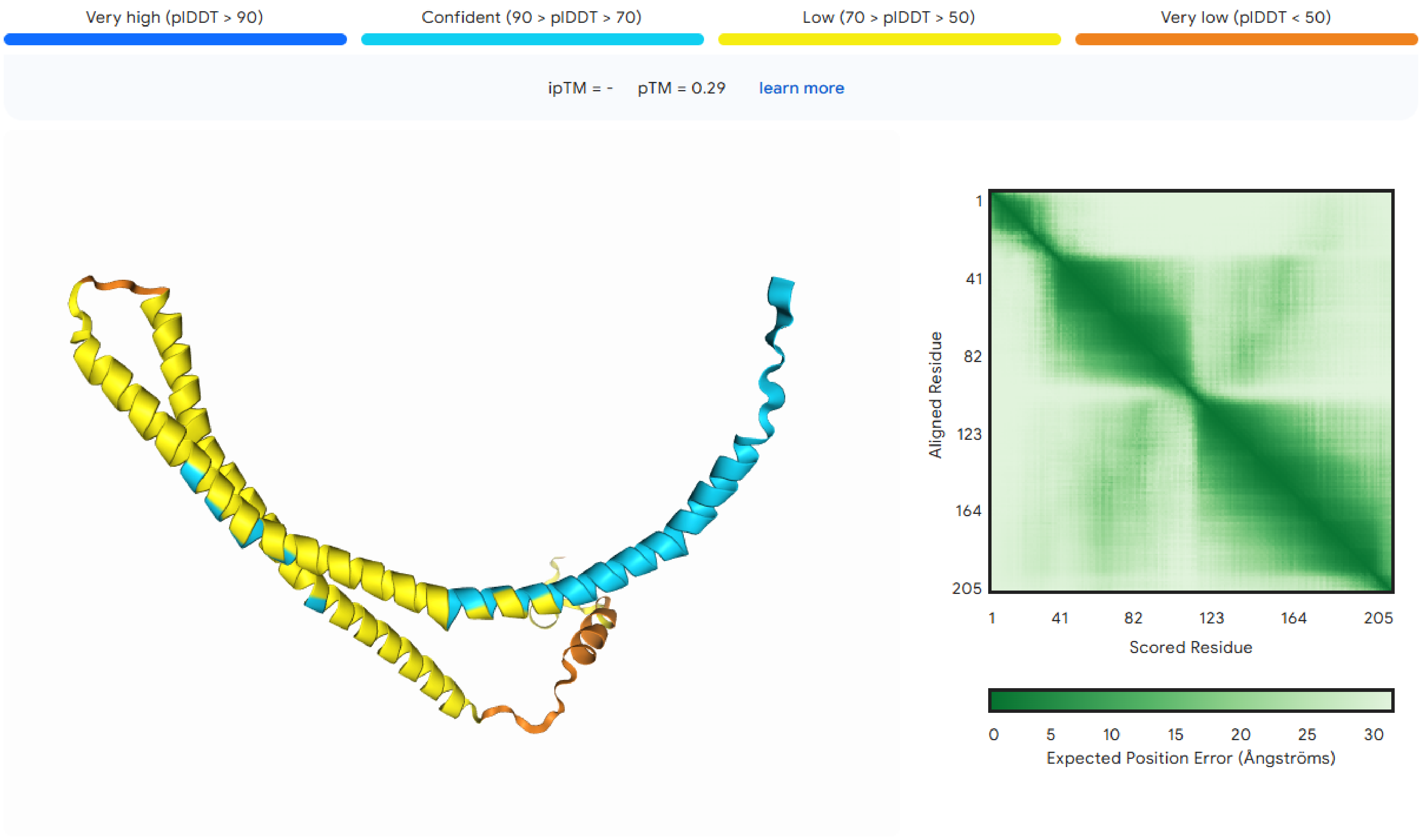

scaffold217_2 *Xenoturbella bocki*

Dimer

Very high (plDDT > 90) | Confident (90 > plDDT > 70) | Low (70 > plDDT > 50) | Very low (plDDT < 50)

ipTM = 0.08 pTM = 0.15 learn more

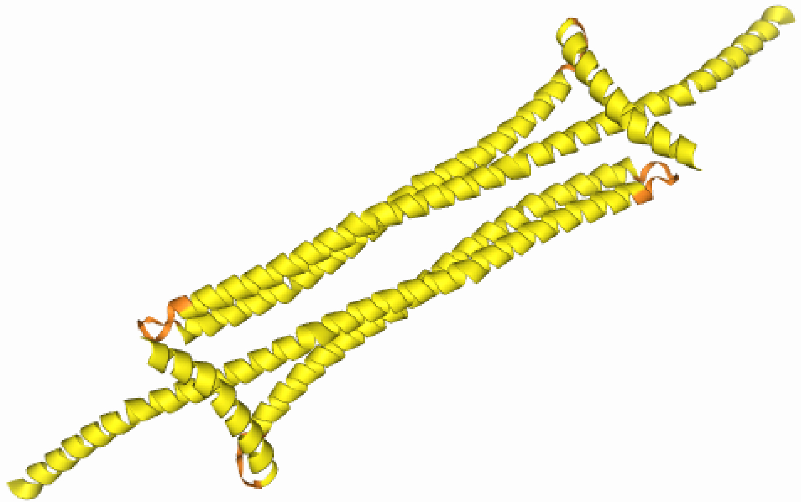

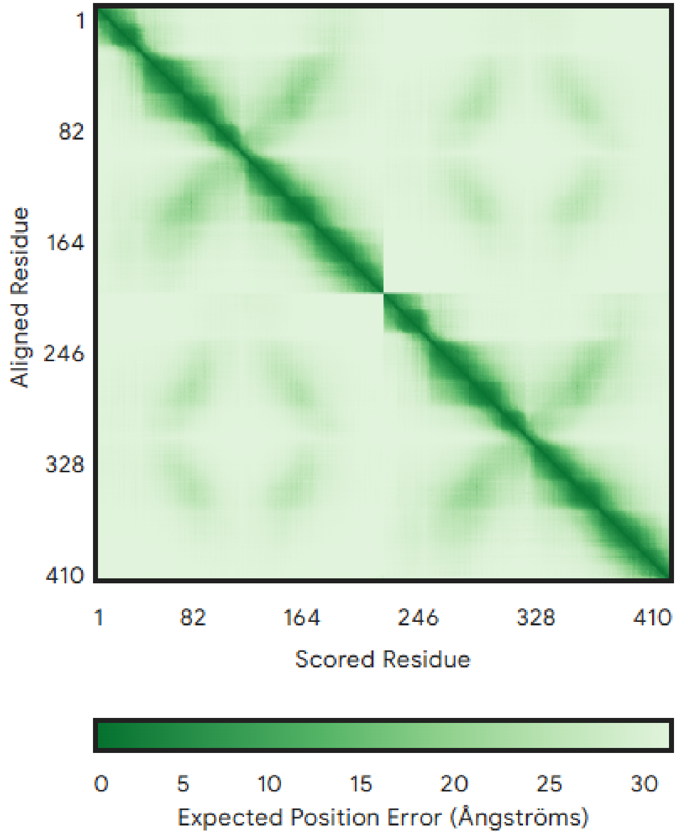

scaffold217_2 *Xenoturbella bocki*

11-mer

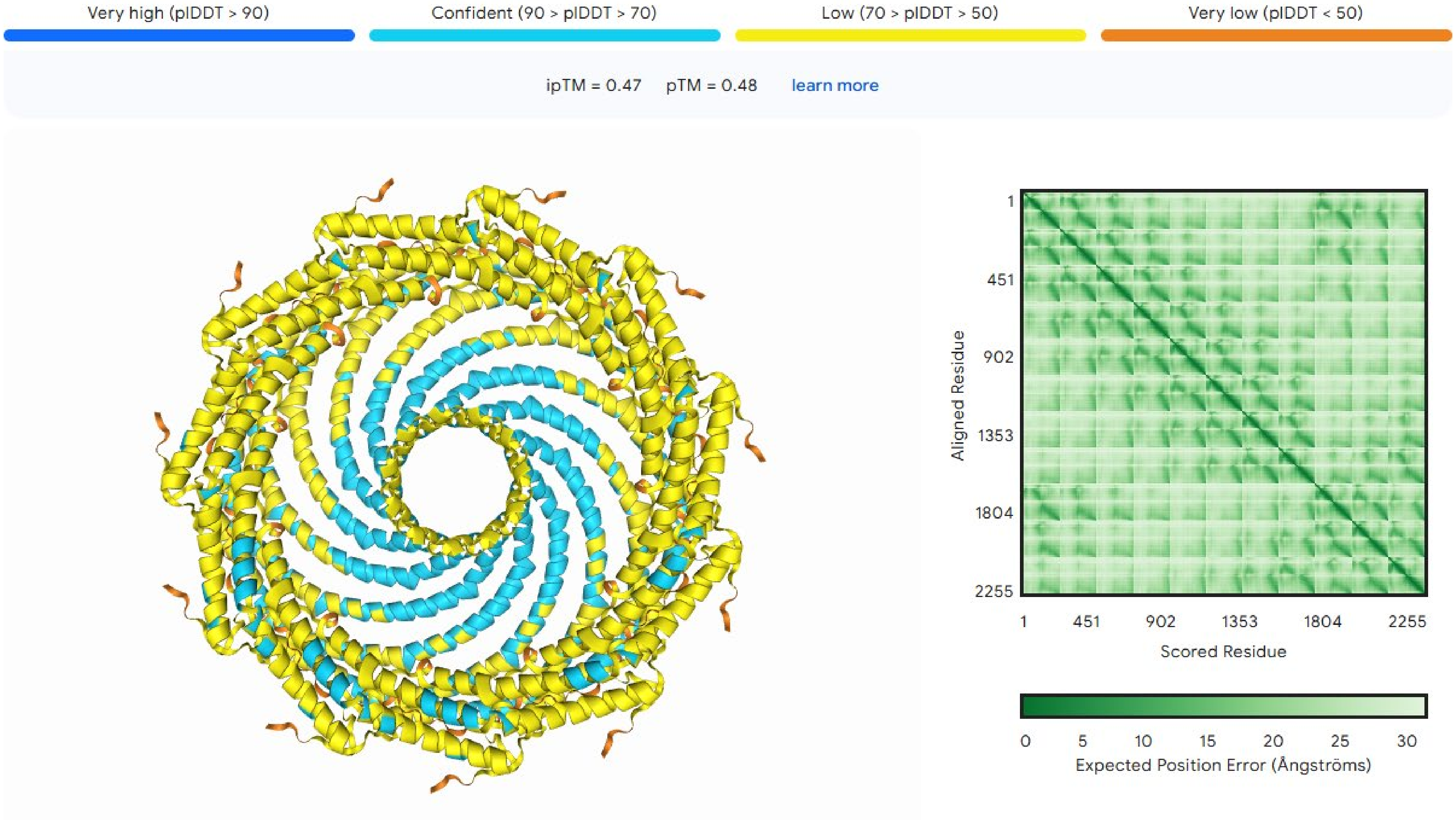

Q03135 (CAV1) *H. sapiens*

Monomer

Very high (pIDDT > 90) | Confident (90 > pIDDT > 70) | Low (70 > pIDDT > 50) | Very low (pIDDT < 50)

ipTM = - pTM = 0.41 learn more

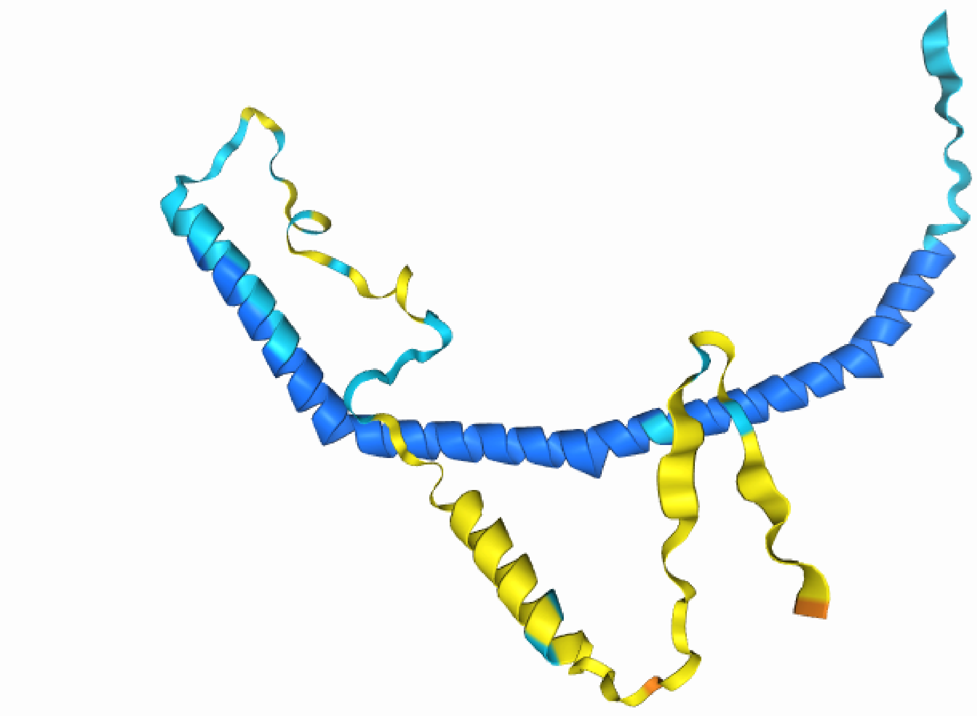

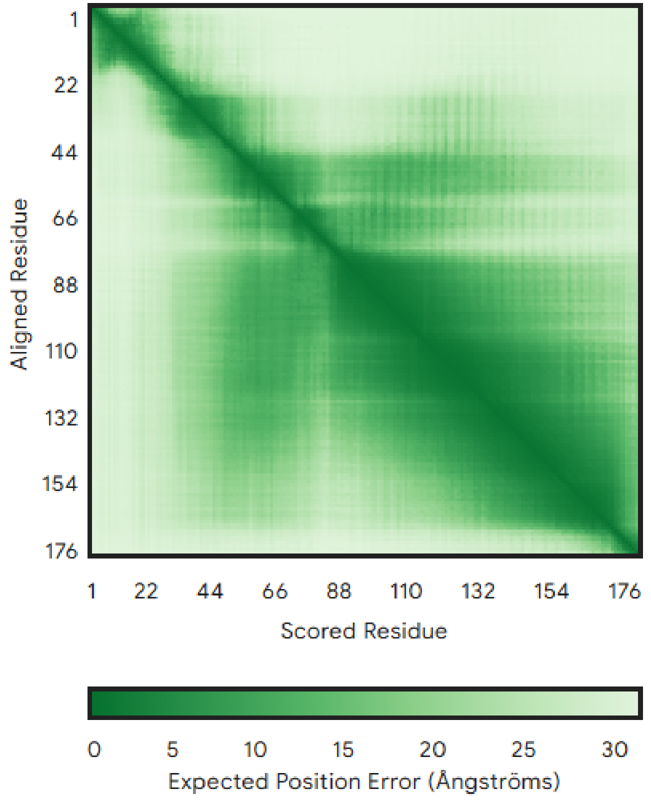

Q03135 (CAV1) *H. sapiens*

Dimer

Very high (pIDDT > 90) | Confident (90 > pIDDT > 70) | Low (70 > pIDDT > 50) | Very low (pIDDT < 50)

ipTM = 0.07 pTM = 0.18 learn more

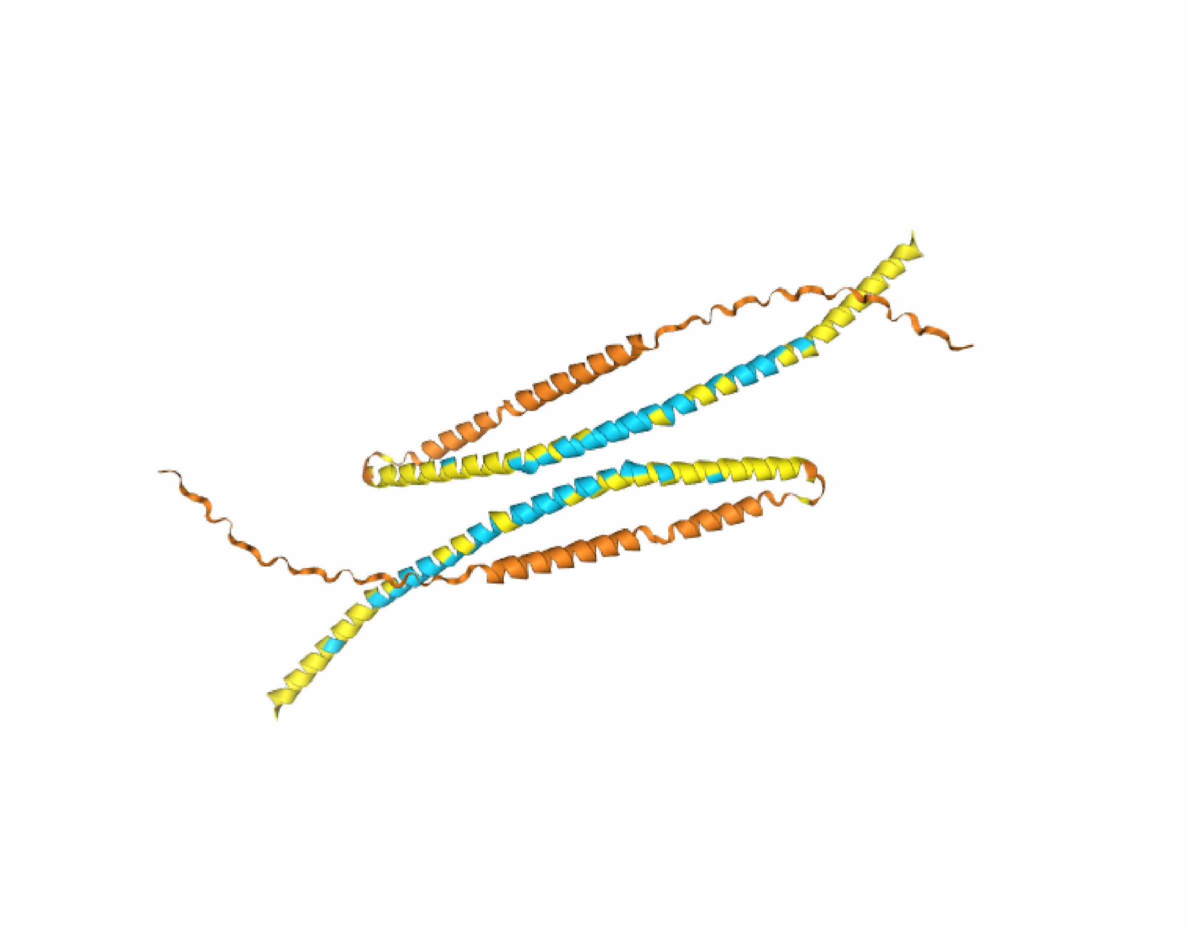

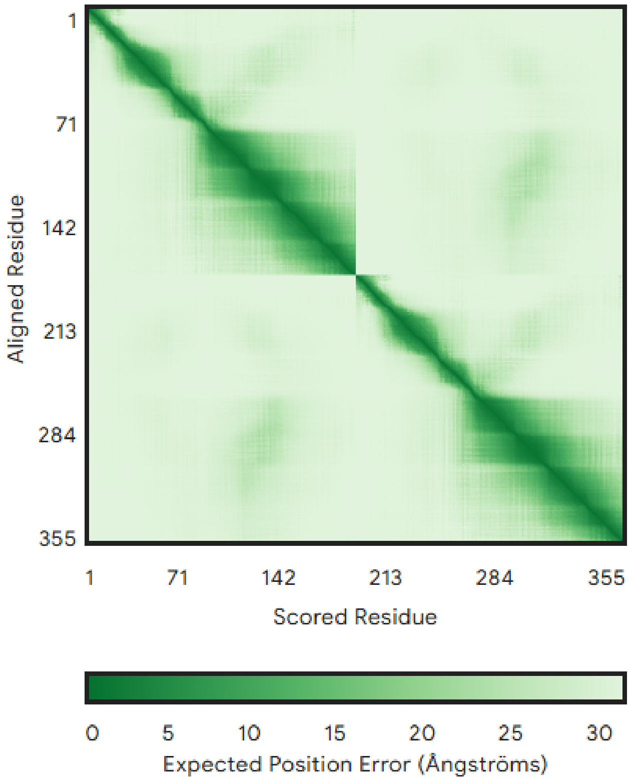

Q03135 (CAV1) *H. sapiens*

11-mer

Very high (pIDDT > 90) | Confident (90 > pIDDT > 70) | Low (70 > pIDDT > 50) | Very low (pIDDT < 50)

ipTM = 0.66 pTM = 0.66 learn more

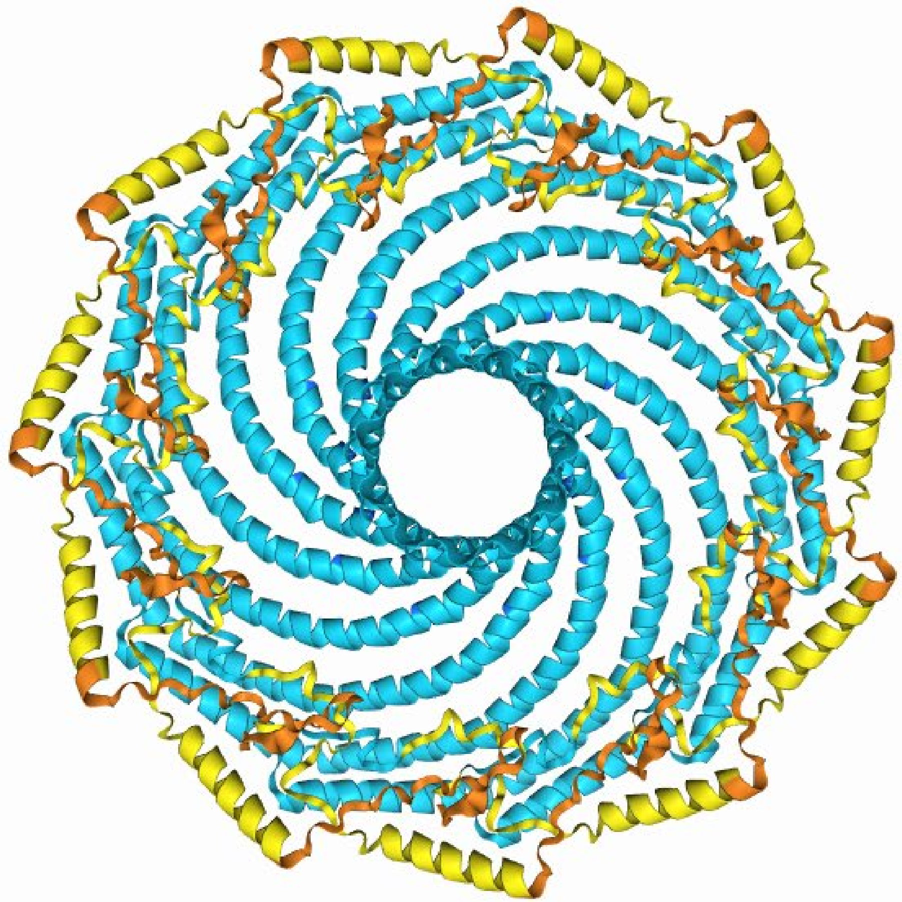

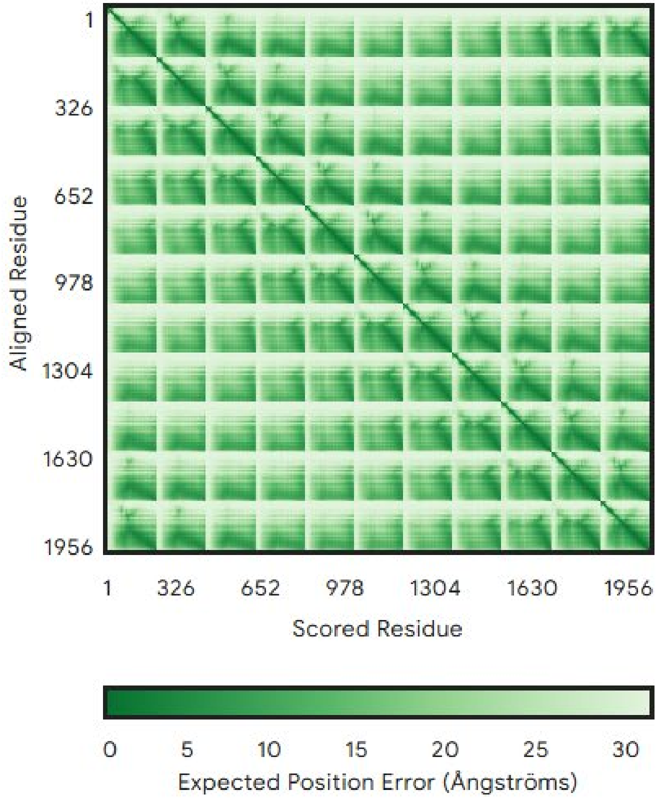

A0A7M7T4C2 *S. purpuratus*

Monomer

Very high (pIDDT > 90)	Confident (90 > pIDDT > 70)	Low (70 > pIDDT > 50)	Very low (pIDDT < 50)

ipTM = -   pTM = 0.33   learn more

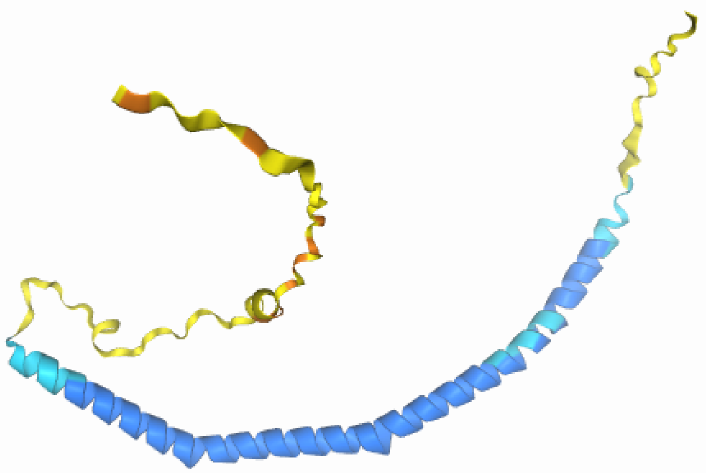

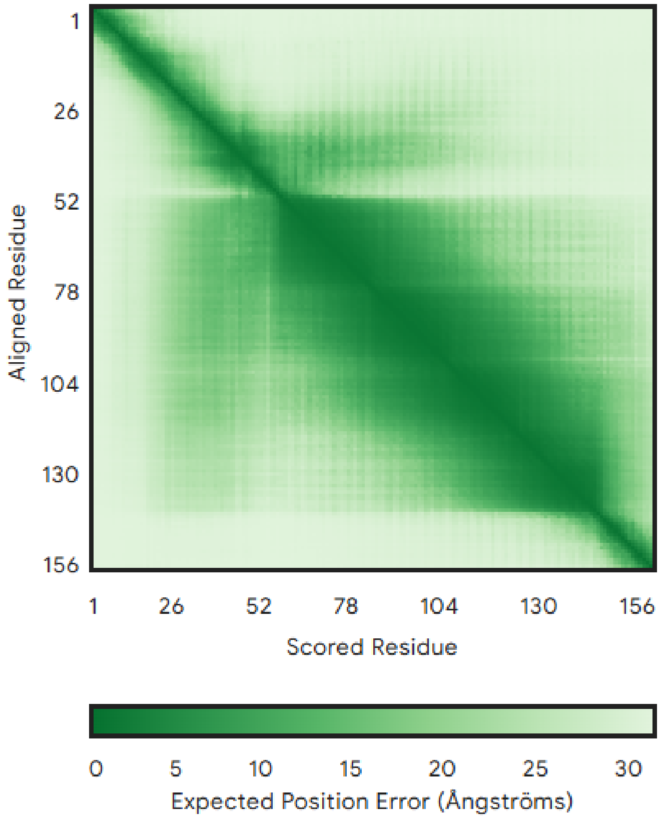

A0A7M7T4C2 *S. purpuratus*

Dimer

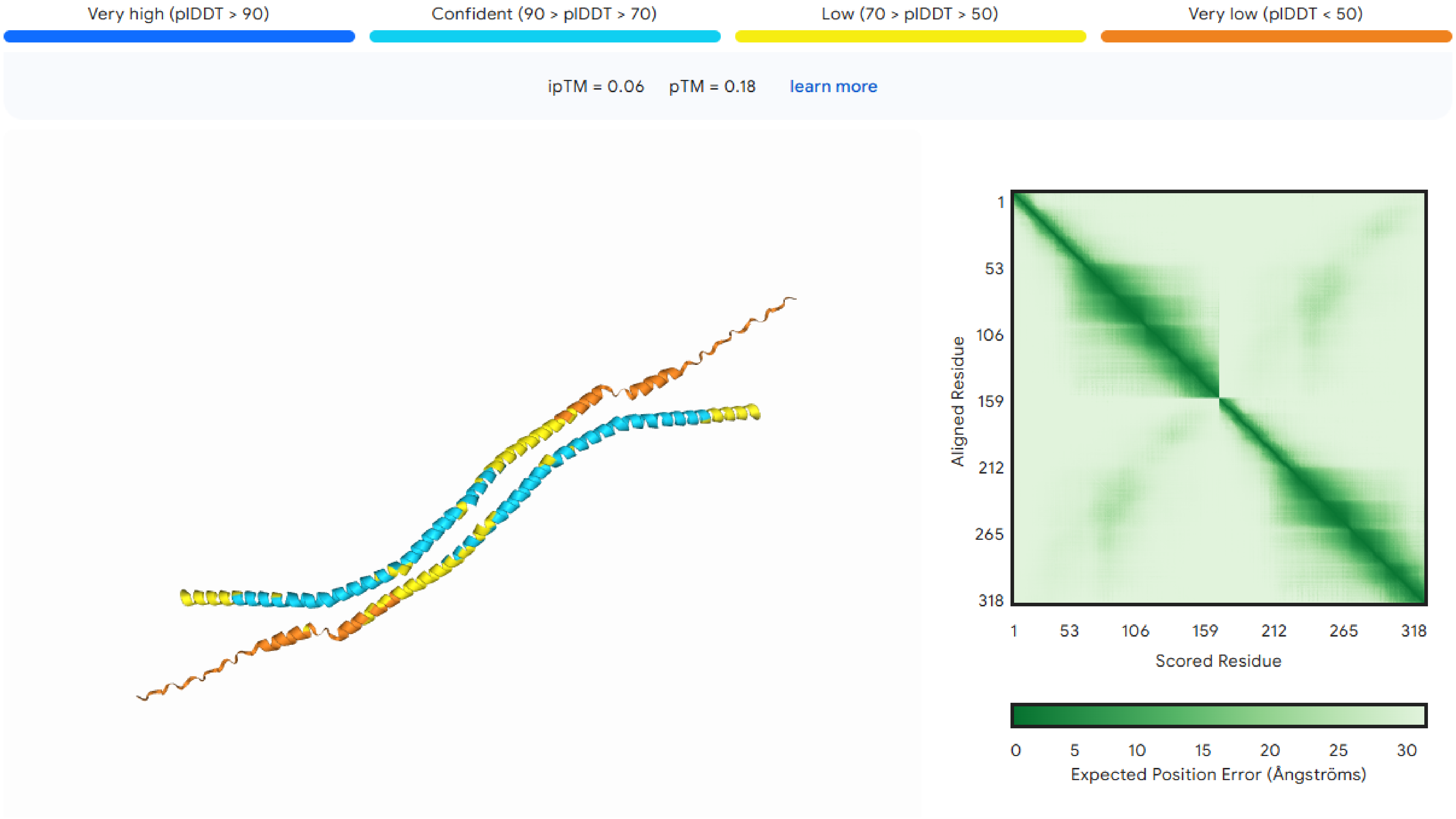

A0A7M7T4C2 ***S. purpuratus***

11-mer

Very high (pIDDT > 90) | Confident (90 > pIDDT > 70) | Low (70 > pIDDT > 50) | Very low (pIDDT < 50)

ipTM = 0.72 pTM = 0.72 learn more

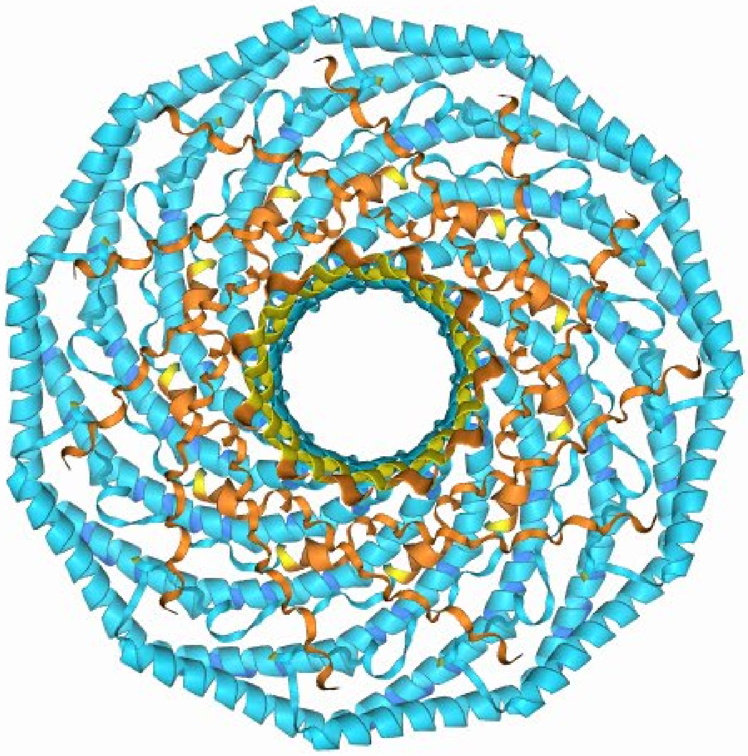

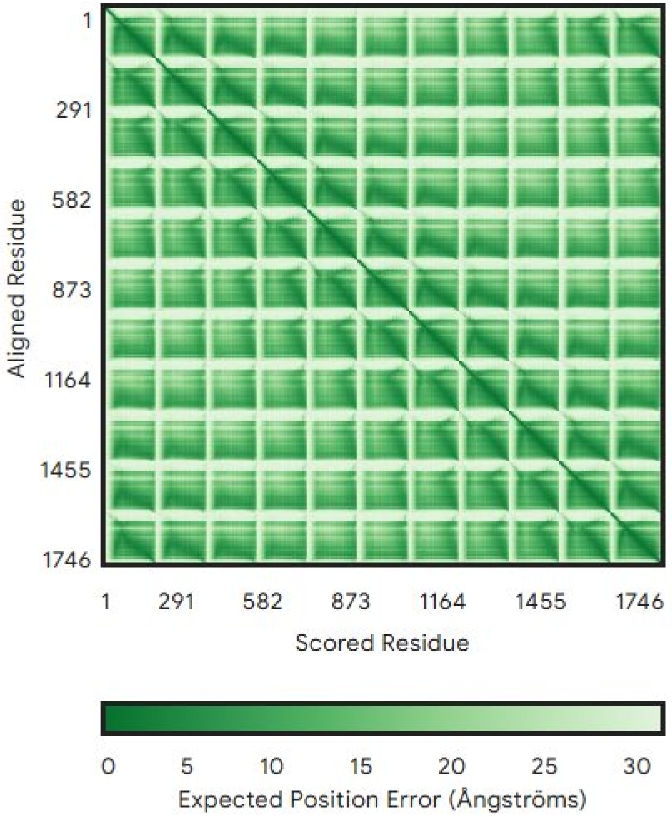

XP_014678552.1 *P. caudatus*

Monomer

Very high (pIDDT > 90) | Confident (90 > pIDDT > 70) | Low (70 > pIDDT > 50) | Very low (pIDDT < 50)

ipTM = - pTM = 0.44 learn more

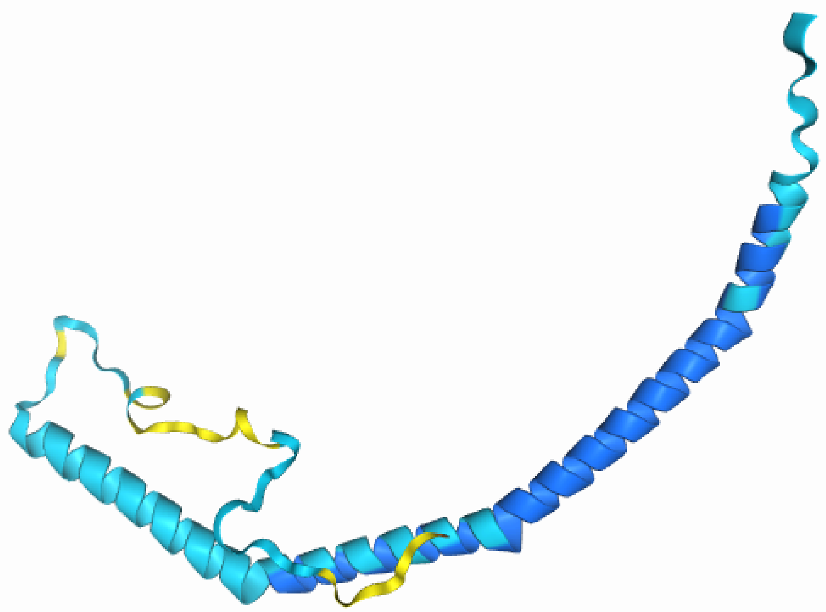

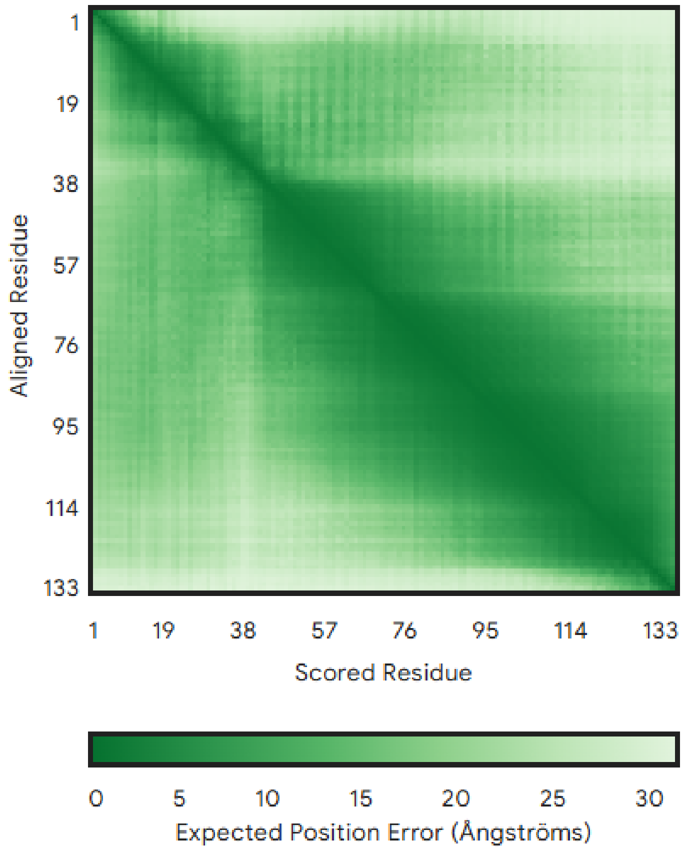

XP_014678552.1 *P. caudatus*

Dimer

Very high (pIDDT > 90) | Confident (90 > pIDDT > 70) | Low (70 > pIDDT > 50) | Very low (pIDDT < 50)

ipTM = 0.07 pTM = 0.2 learn more

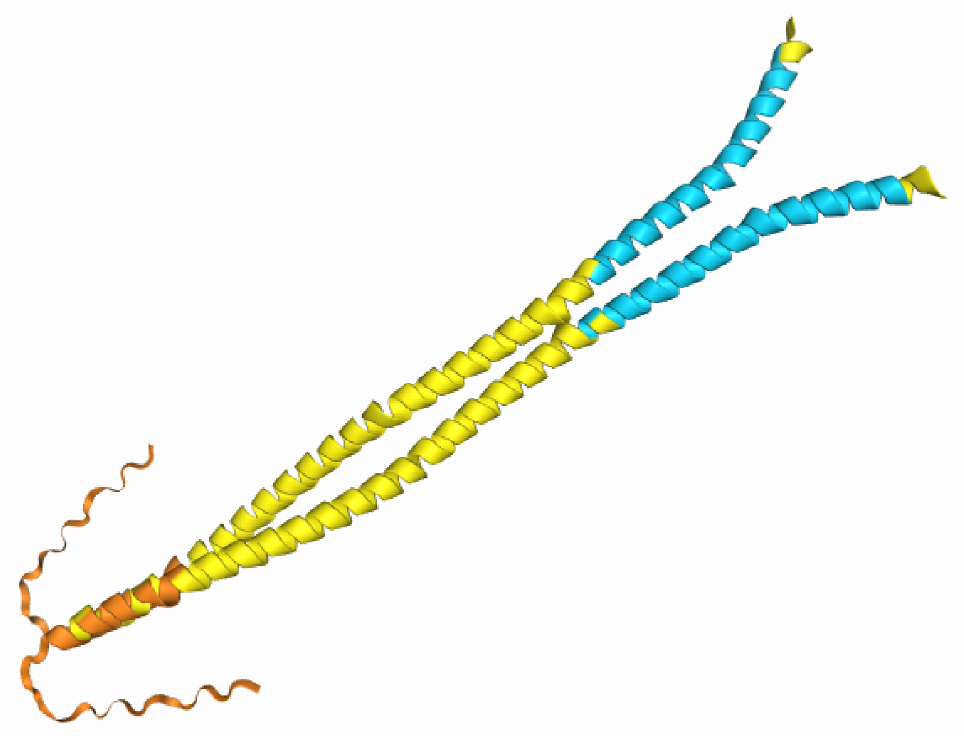

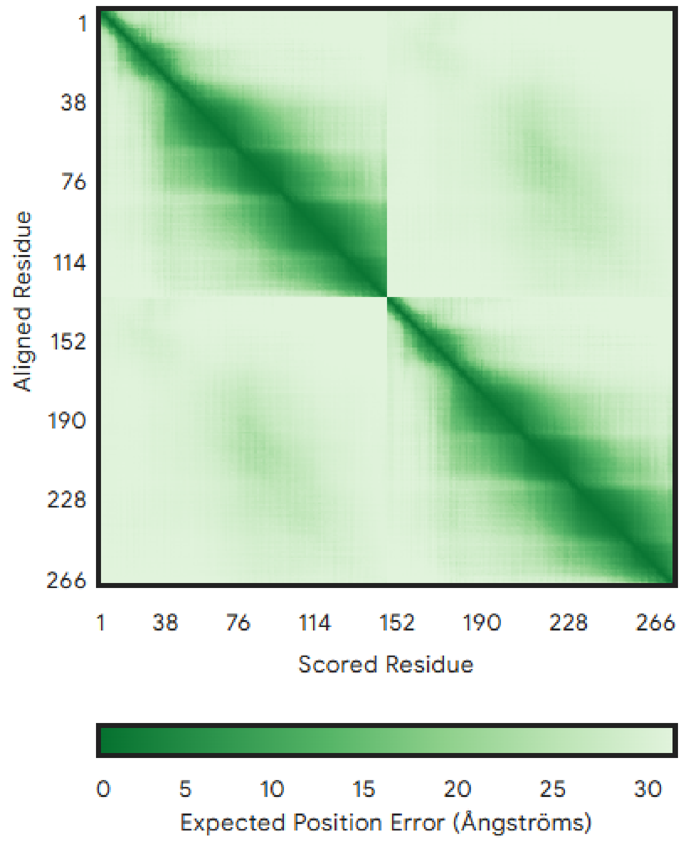

XP_014678552.1 *P. caudatus*

11-mer

Very high (plDDT > 90) Confident (90 > plDDT > 70) Low (70 > plDDT > 50) Very low (plDDT < 50)

ipTM = 0.74 pTM = 0.75 learn more

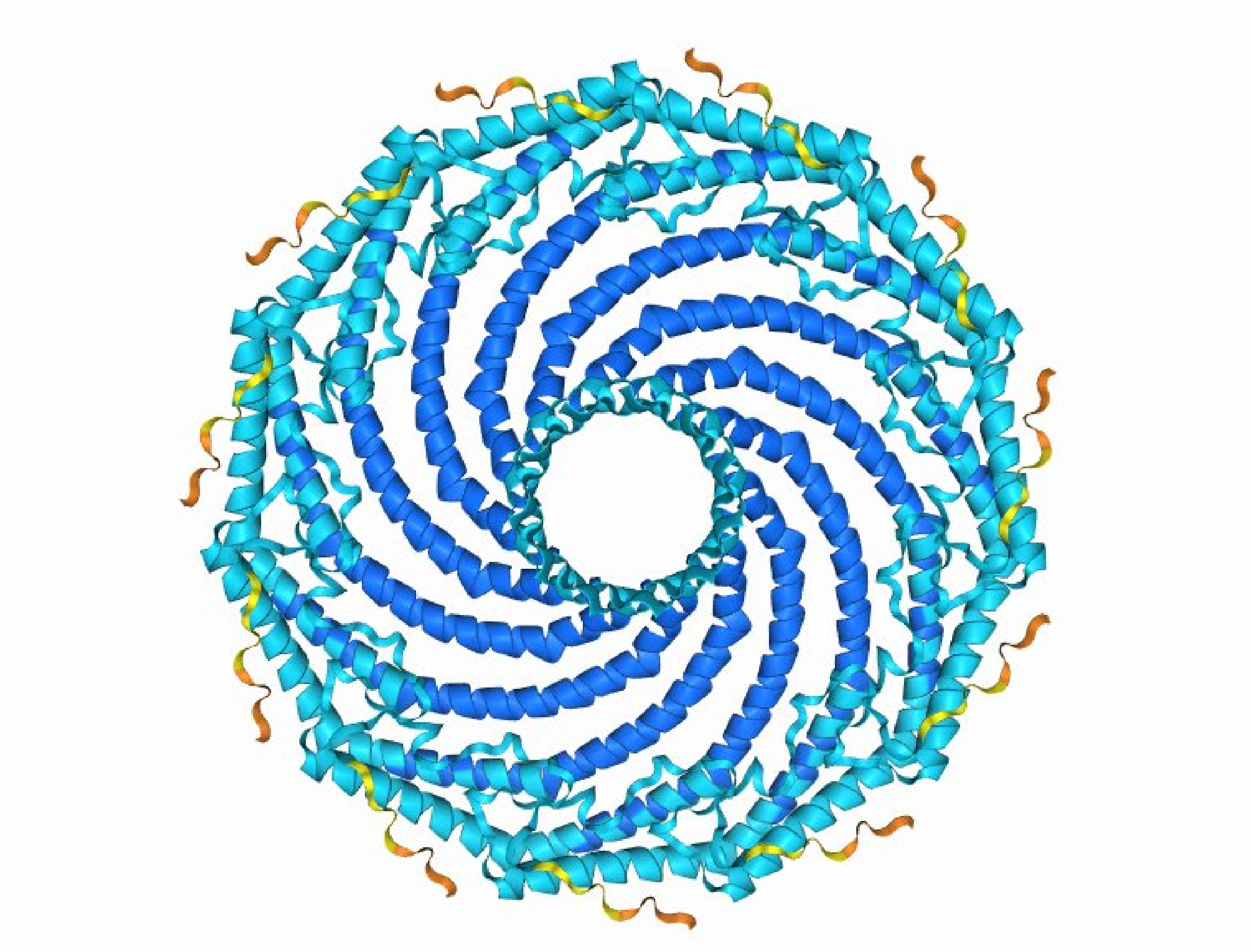

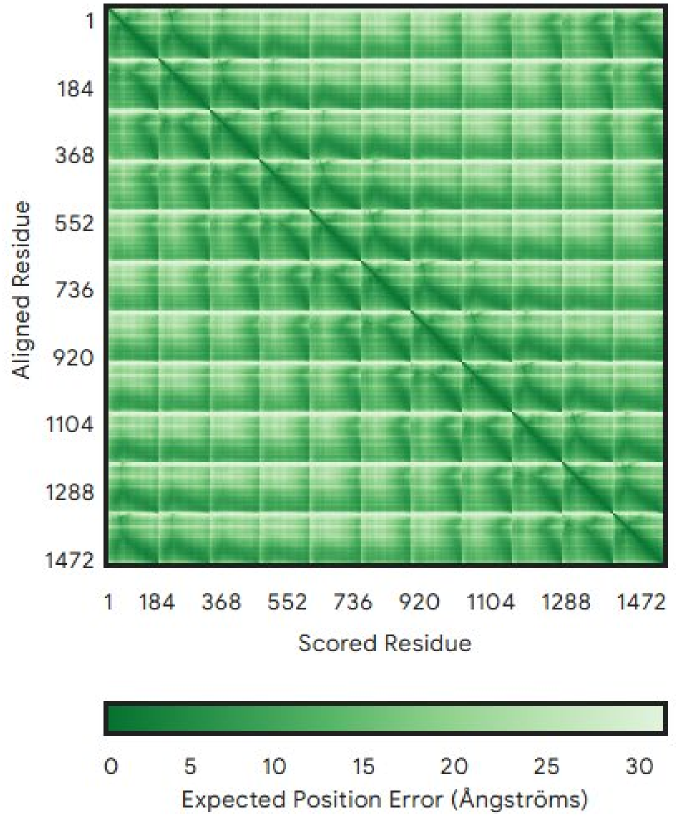

A0A7M7R2L2 *A. mellifera*

Monomer

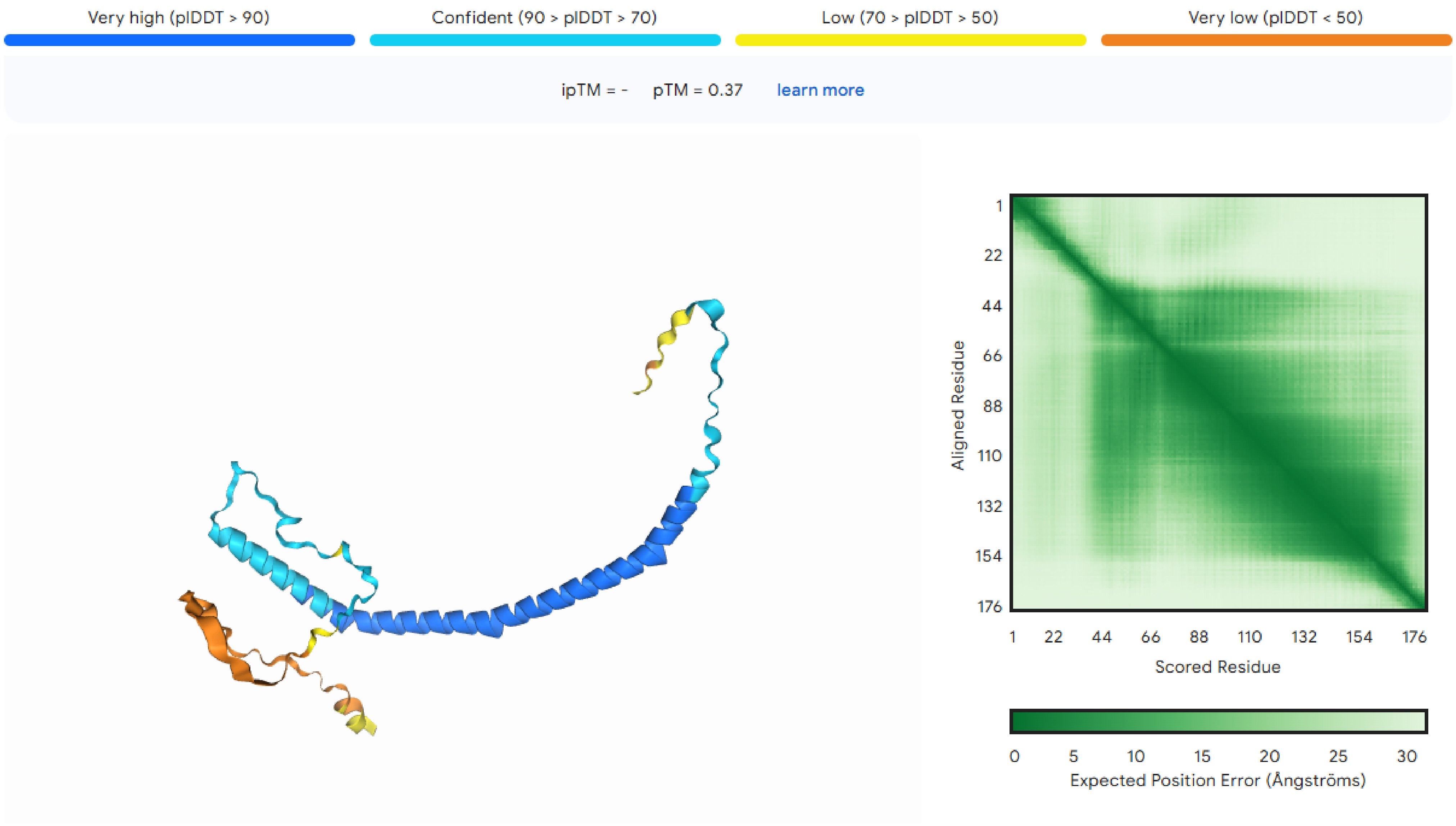

A0A7M7R2L2 *A. mellifera*

Dimer

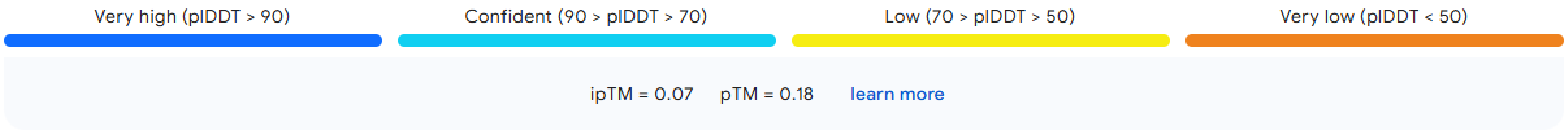

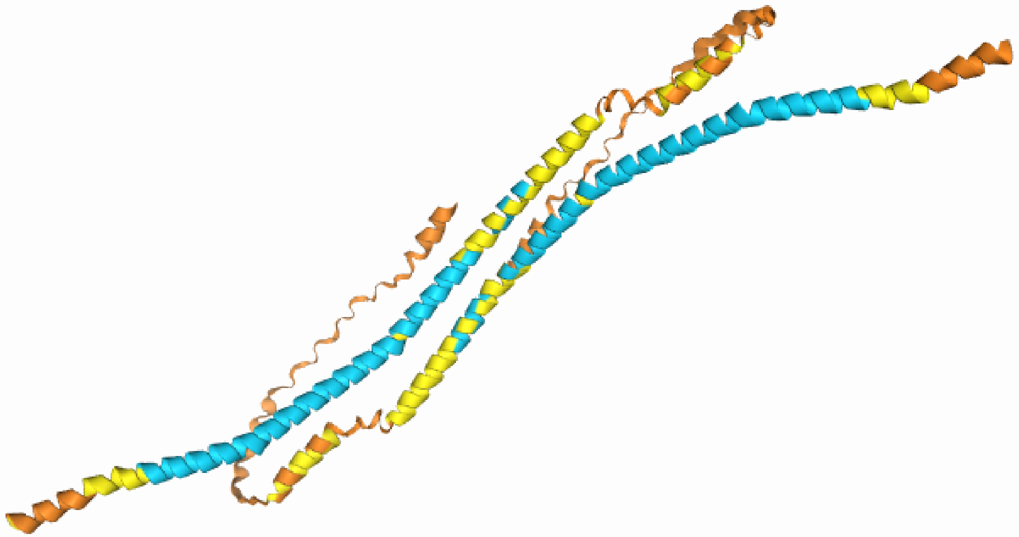

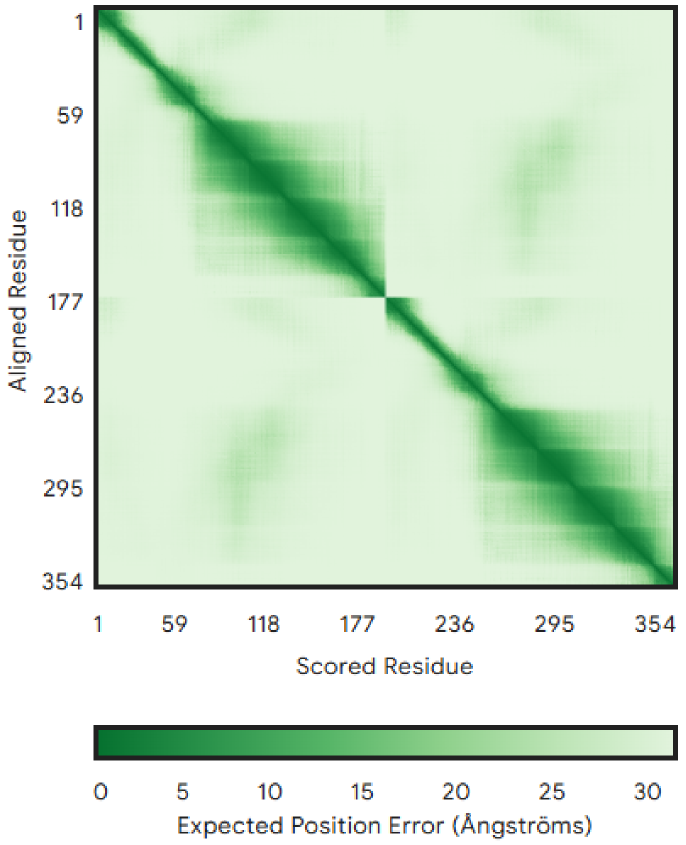

A0A7M7R2L2 *A. mellifera*

11-mer

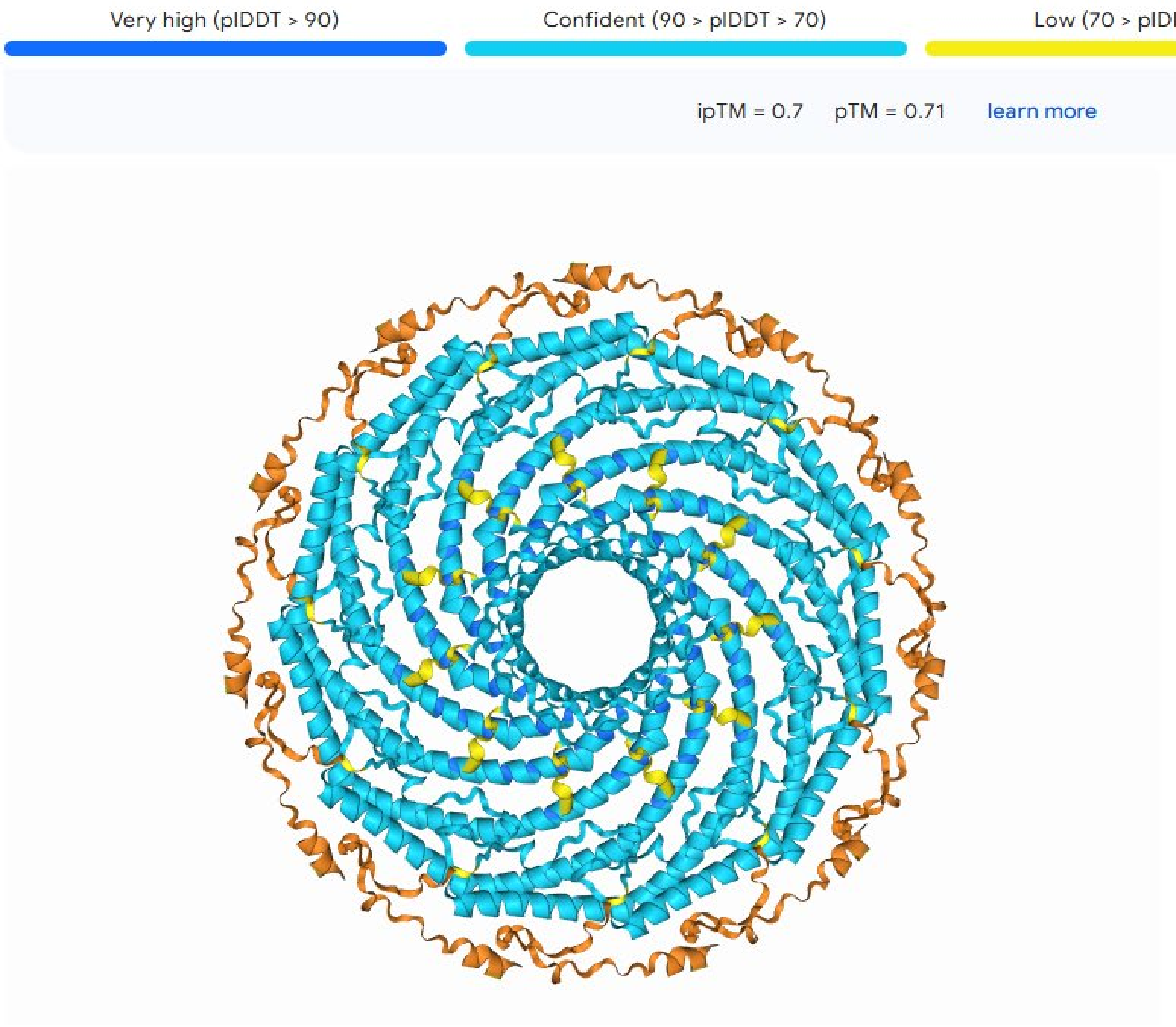

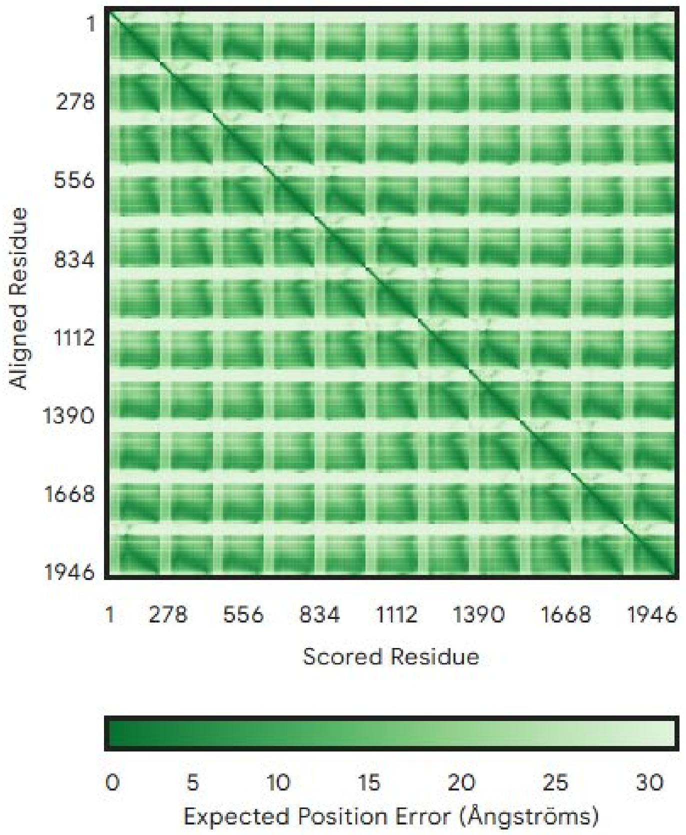

Q18879 *C. elegans*

Monomer

Very high (pIDDT > 90) | Confident (90 > pIDDT > 70) | Low (70 > pIDDT > 50) | Very low (pIDDT < 50)

ipTM = - pTM = 0.3 learn more

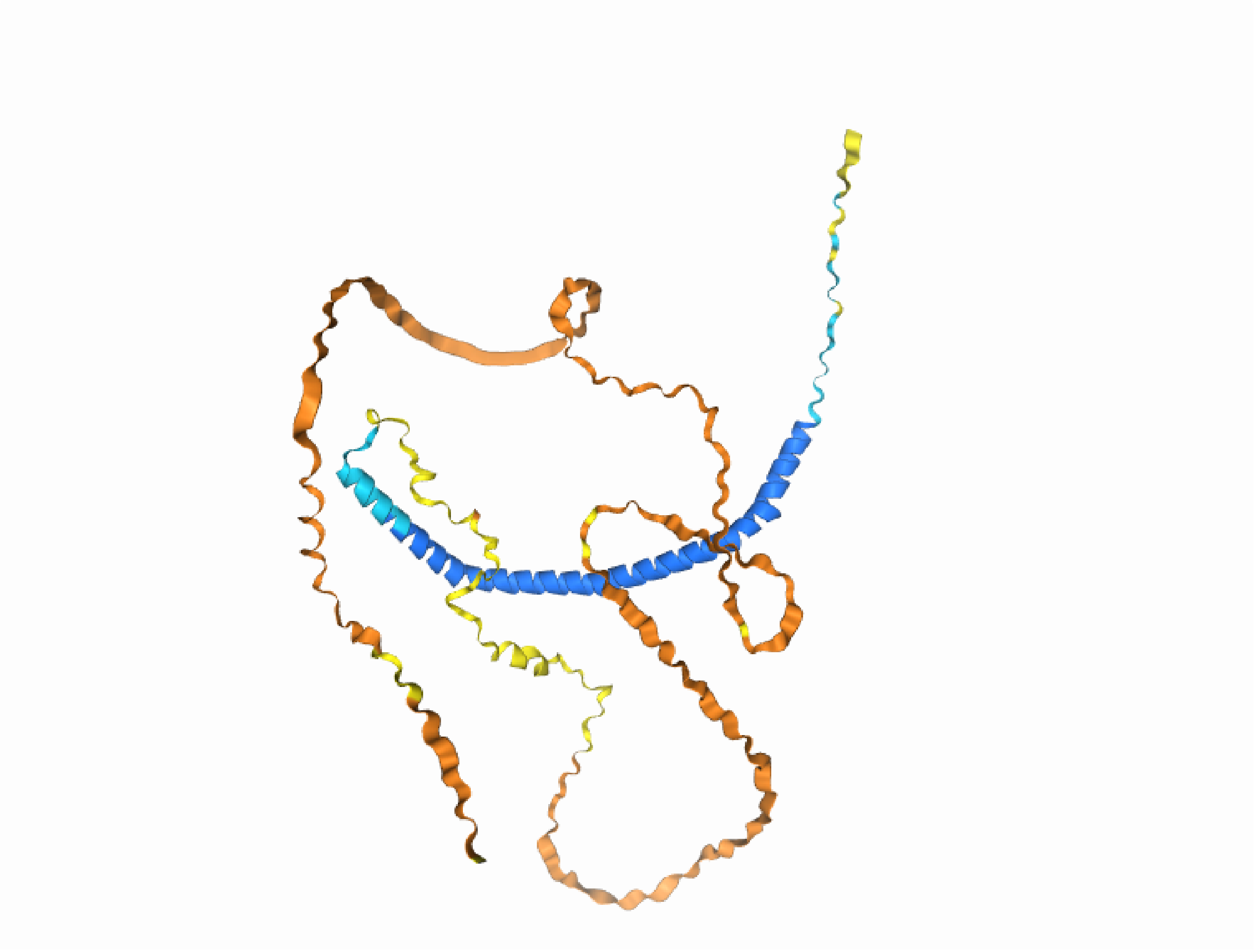

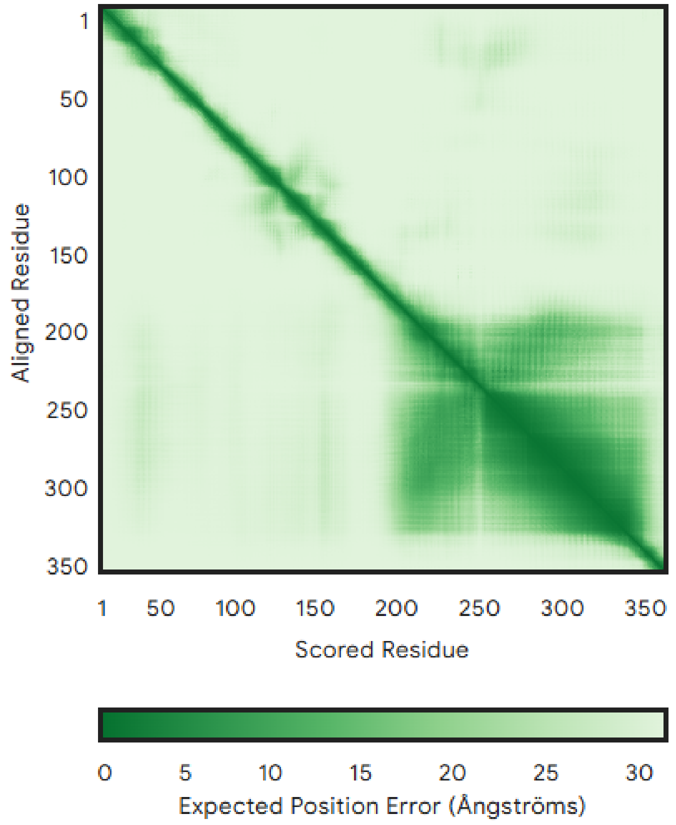

Q18879 ***C. elegans***

Dimer

Very high (pIDDT > 90) | Confident (90 > pIDDT > 70) | Low (70 > pIDDT > 50) | Very low (pIDDT < 50)

ipTM = 0.09   pTM = 0.15   learn more

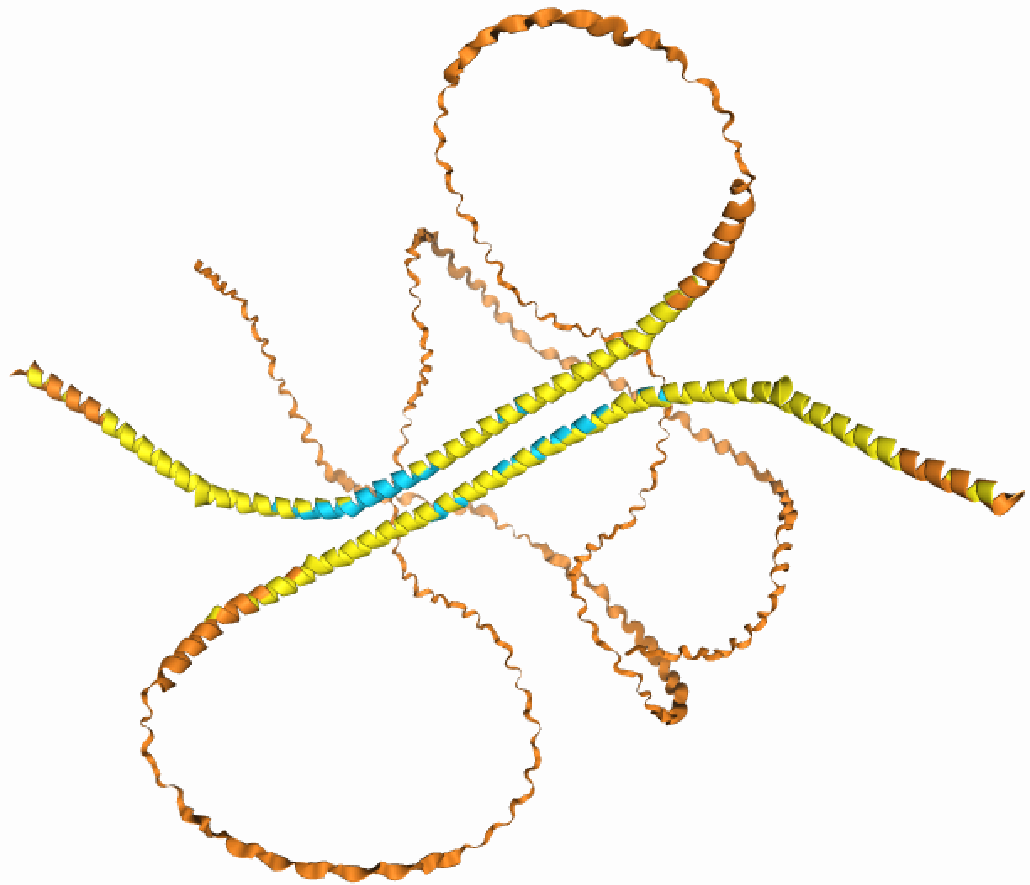

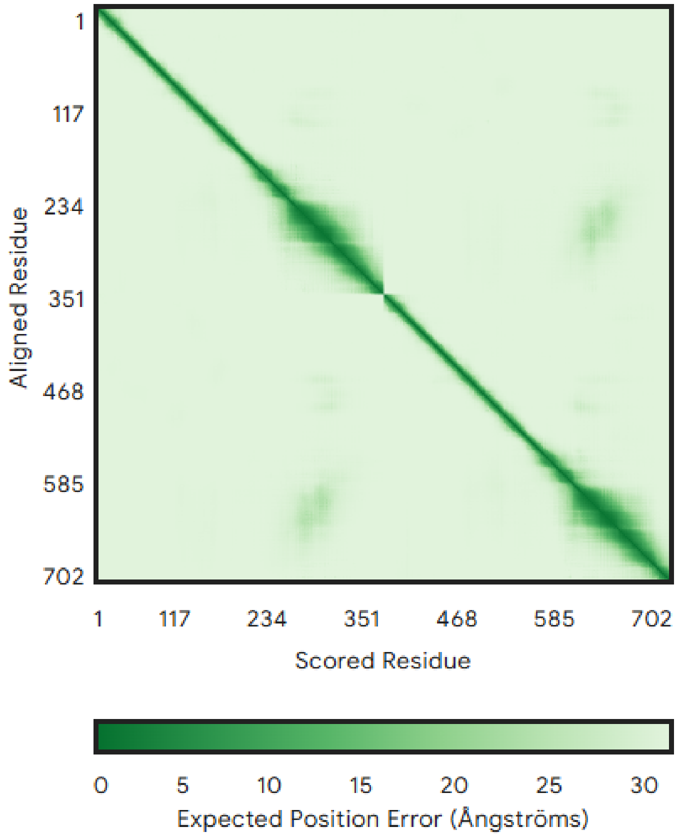

Q18879 ***C. elegans***

11-mer

Very high (pIDDT > 90) Confident (90 > pIDDT > 70) Low (70 > pIDDT > 50) Very low (pIDDT < 50)

ipTM = 0.51 pTM = 0.51 learn more

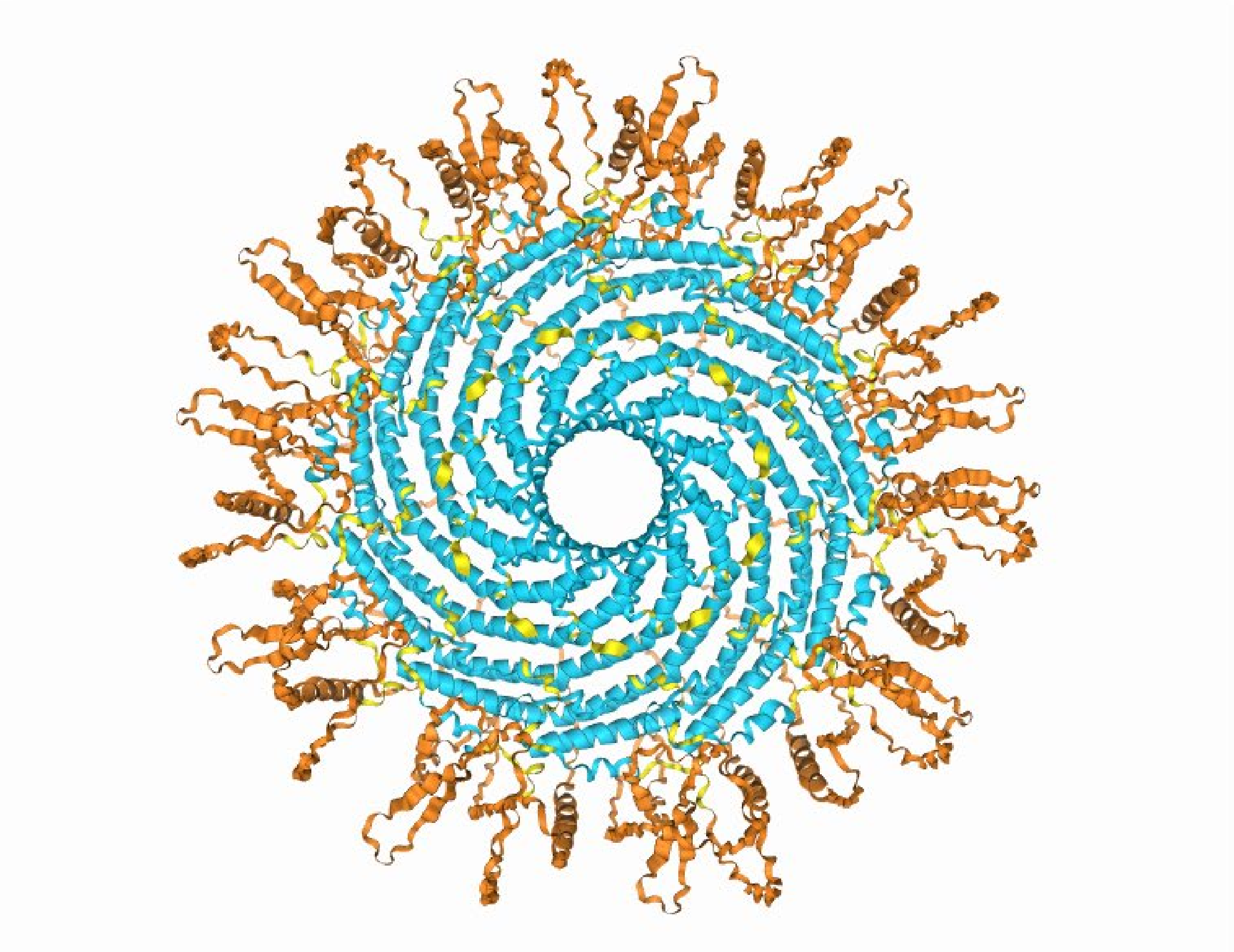

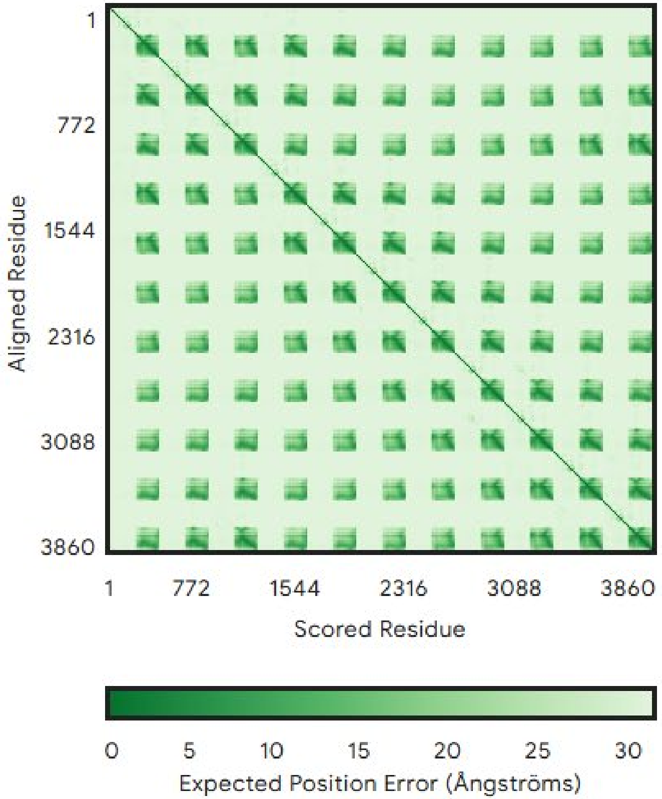

A0A3M7RD35 *B. plicatilis*

Monomer

Very high (pIDDT > 90) | Confident (90 > pIDDT > 70) | Low (70 > pIDDT > 50) | Very low (pIDDT < 50)

ipTM = - pTM = 0.42 learn more

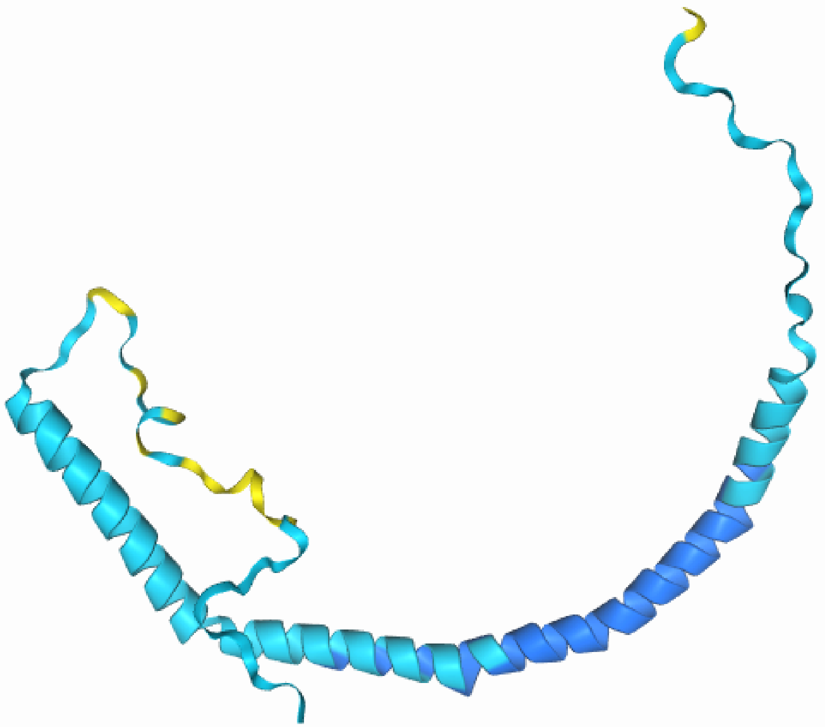

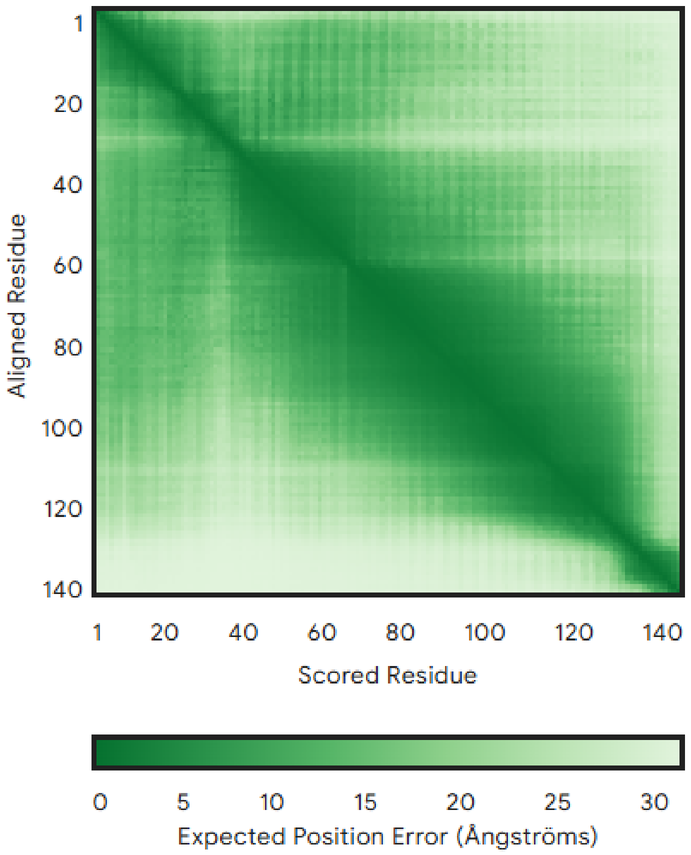

A0A3M7RD35 *B. plicatilis*

Dimer

Very high (pIDDT > 90) | Confident (90 > pIDDT > 70) | Low (70 > pIDDT > 50) | Very low (pIDDT < 50)

ipTM = 0.06 pTM = 0.21 learn more

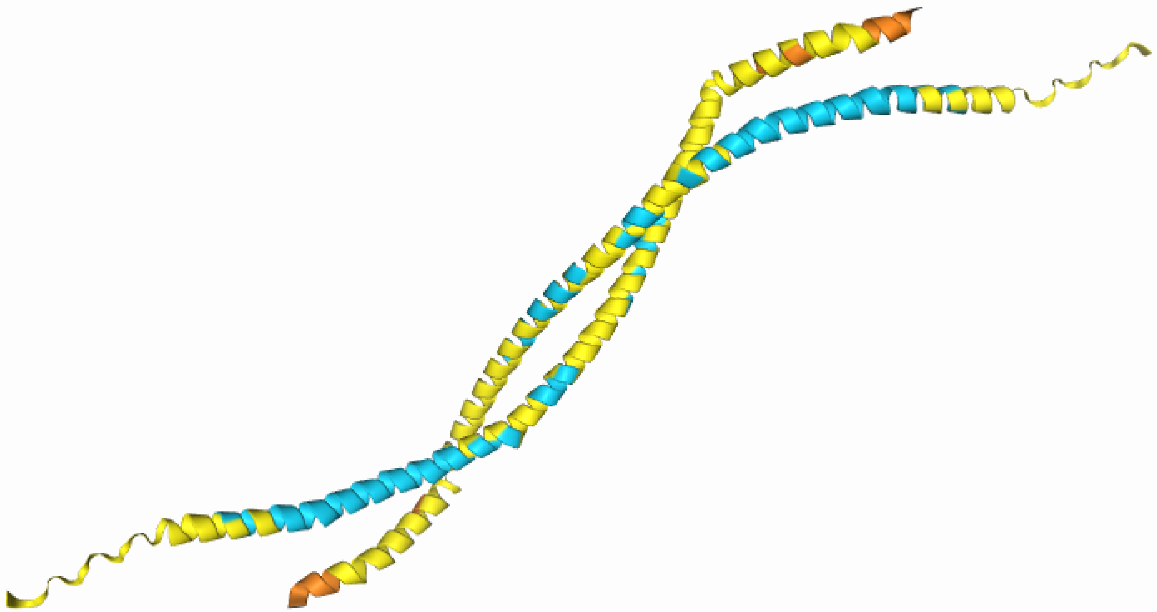

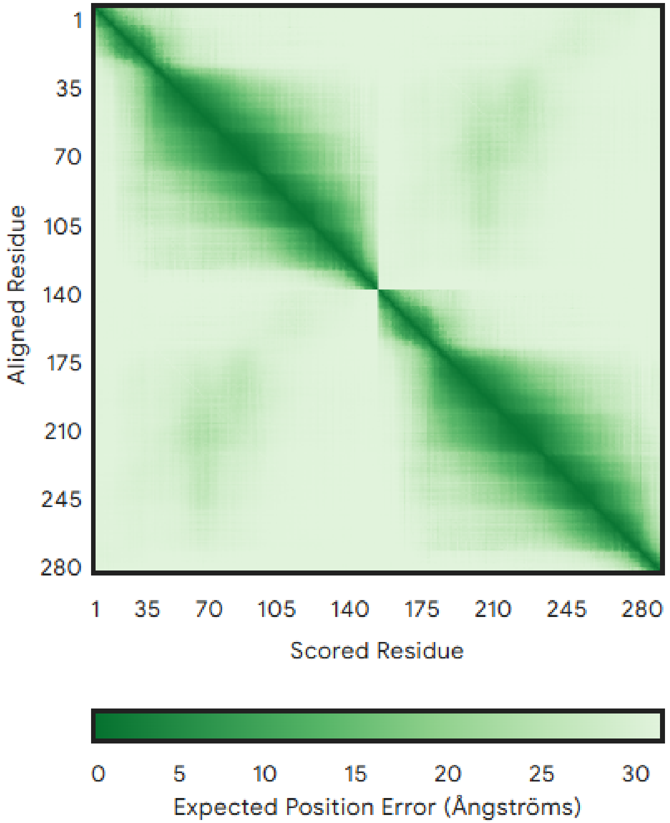

A0A3M7RD35 *B. plicatilis*

11-mer

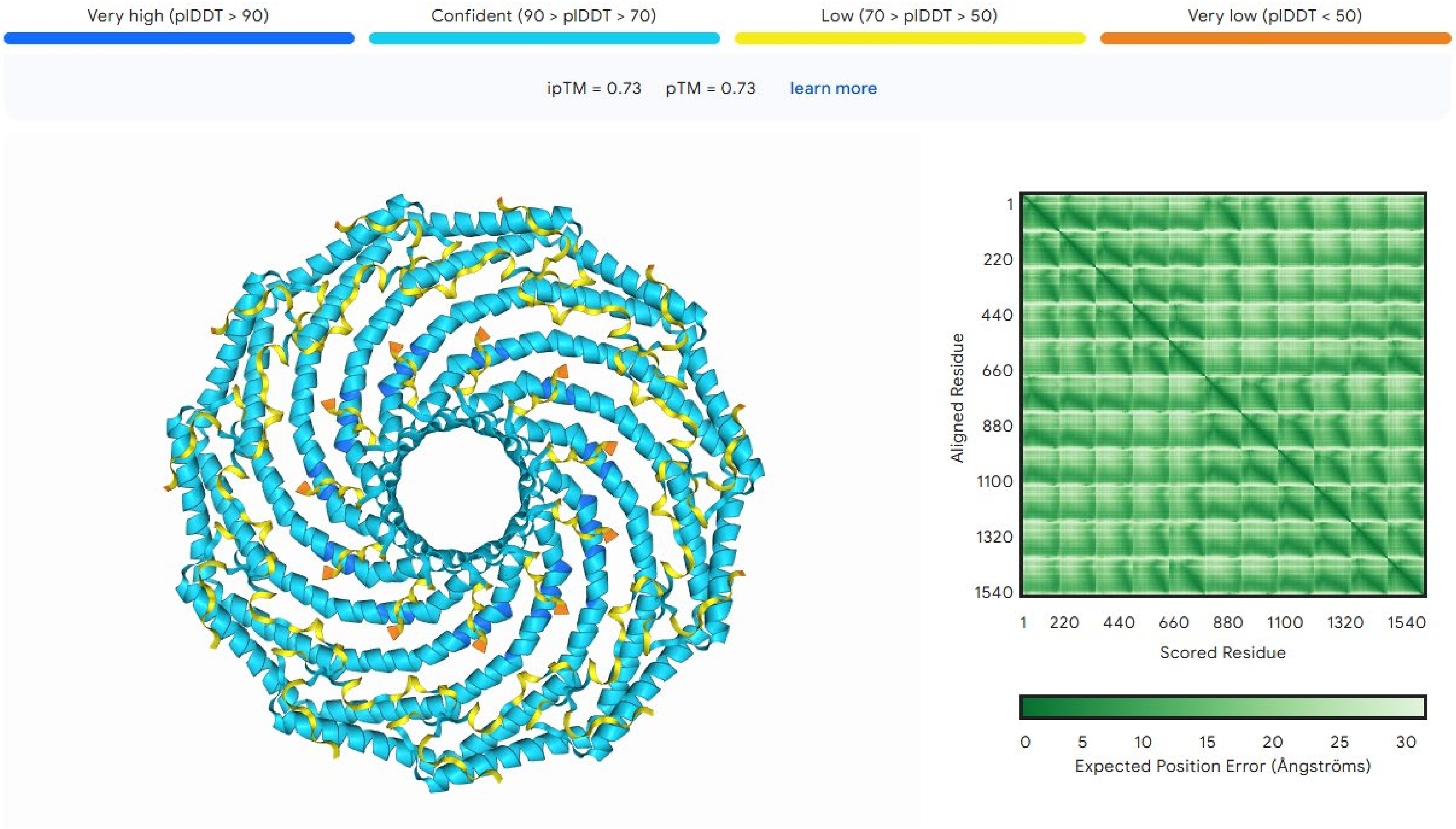

A0A267DC90 *M. lignano*

Monomer

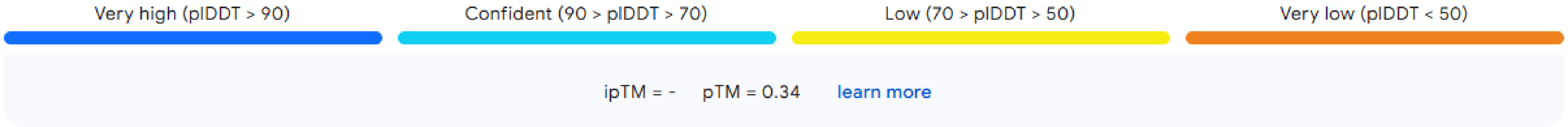

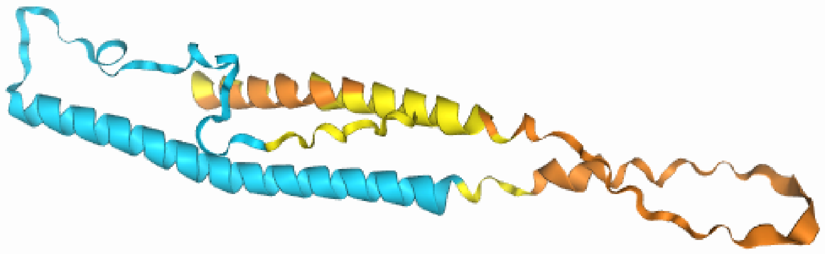

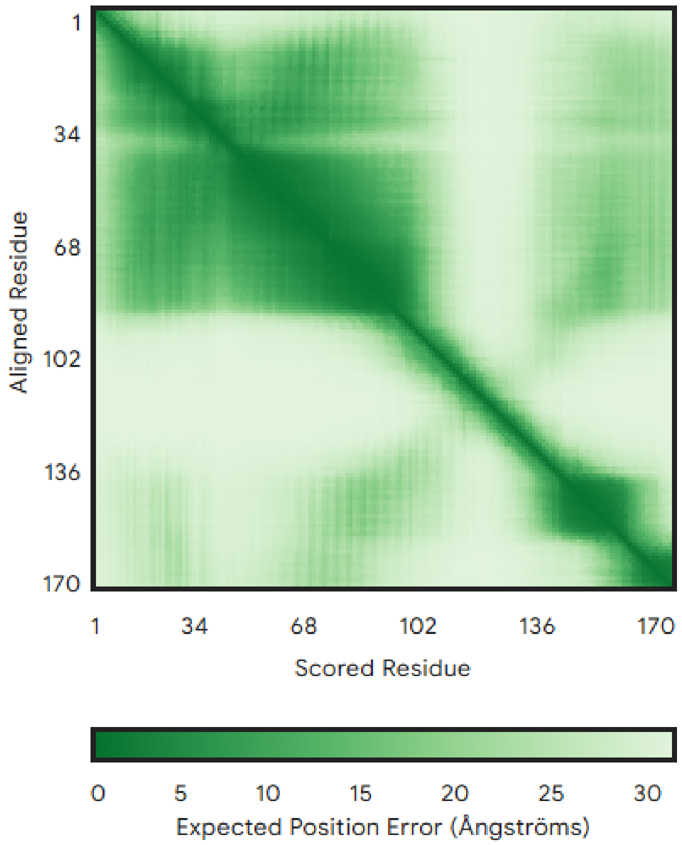

A0A267DC90 *M. lignano*

Dimer

Very high (pIDDT > 90) | Confident (90 > pIDDT > 70) | Low (70 > pIDDT > 50) | Very low (pIDDT < 50)

ipTM = 0.1   pTM = 0.22   learn more

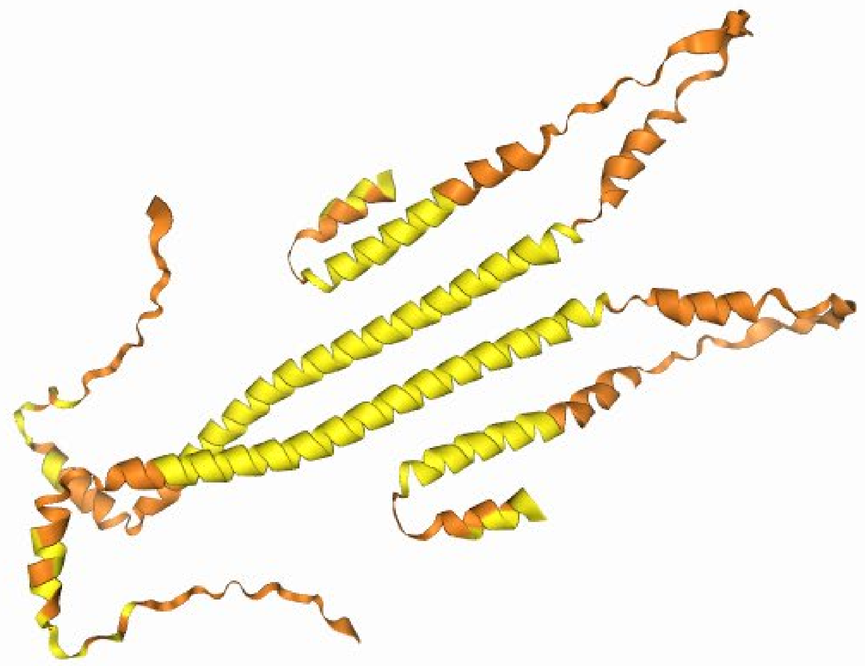

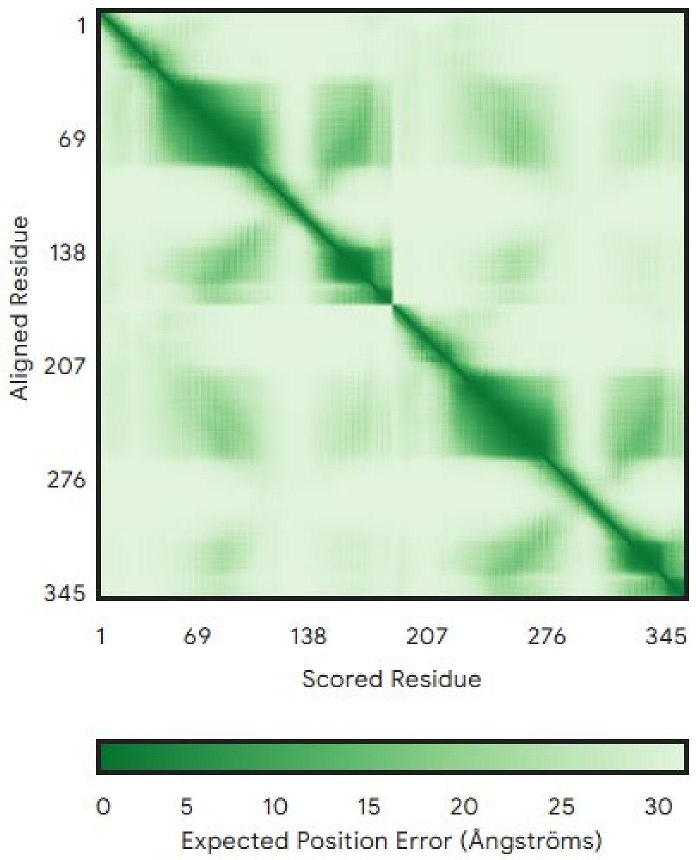

A0A267DC90 *M. lignano*

11-mer

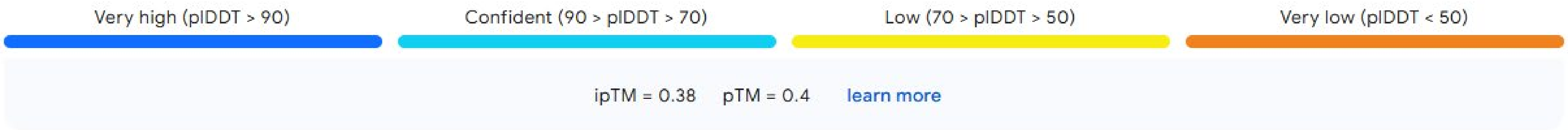

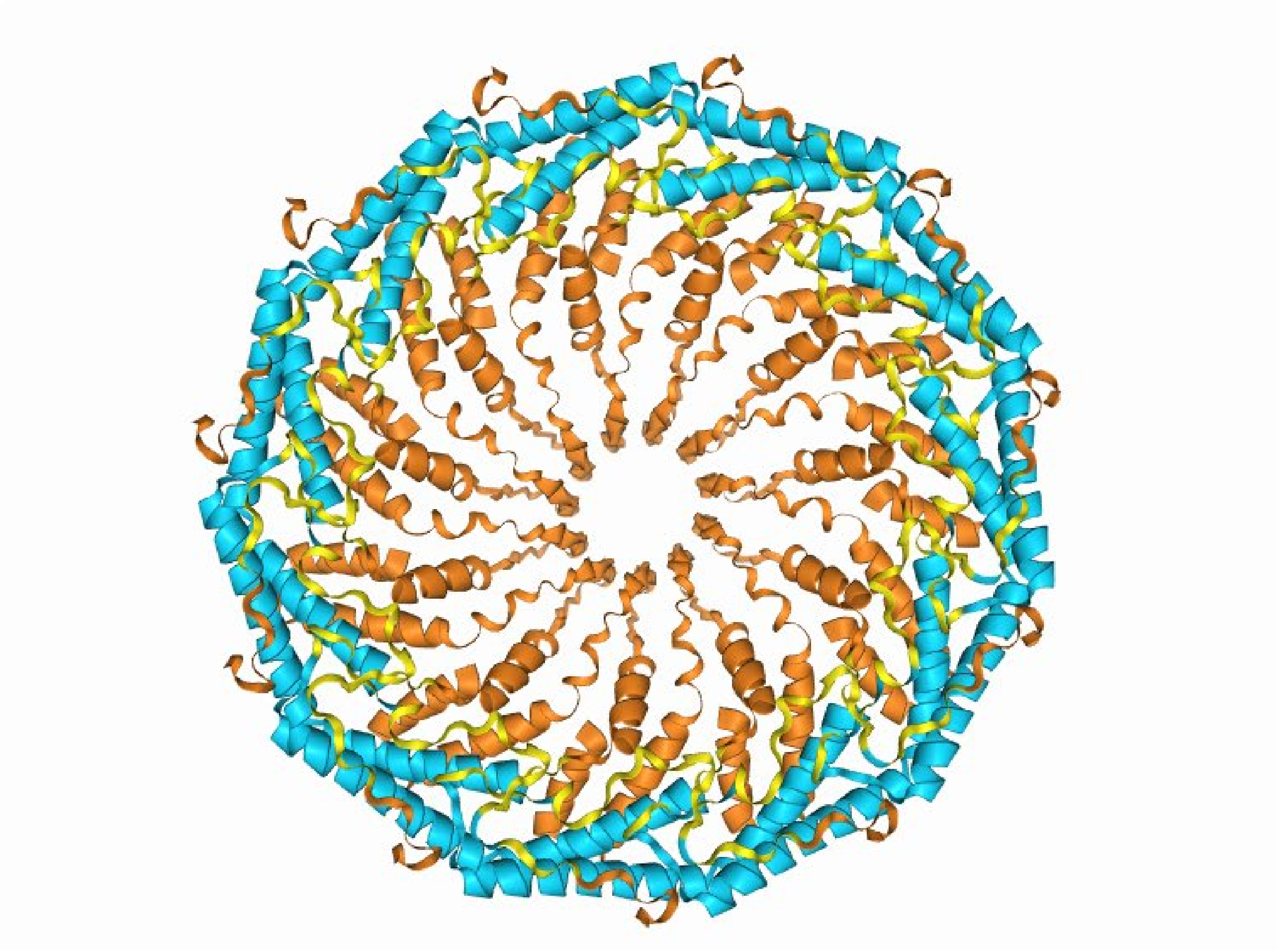

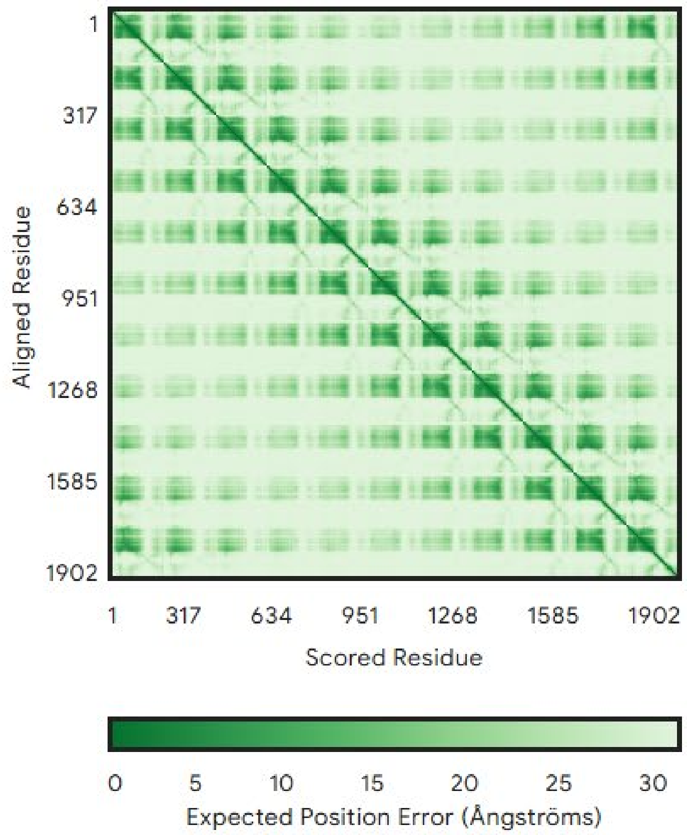

A0A2T7NUA4 *P. canaliculata*

Monomer

Very high (pIDDT > 90) | Confident (90 > pIDDT > 70) | Low (70 > pIDDT > 50) | Very low (pIDDT < 50)

ipTM = - pTM = 0.31 learn more

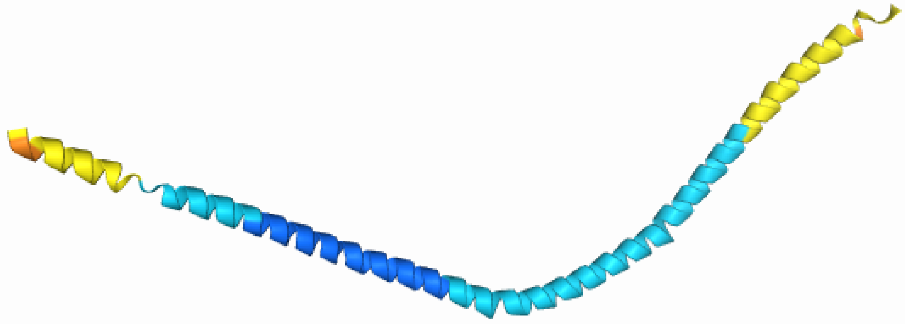

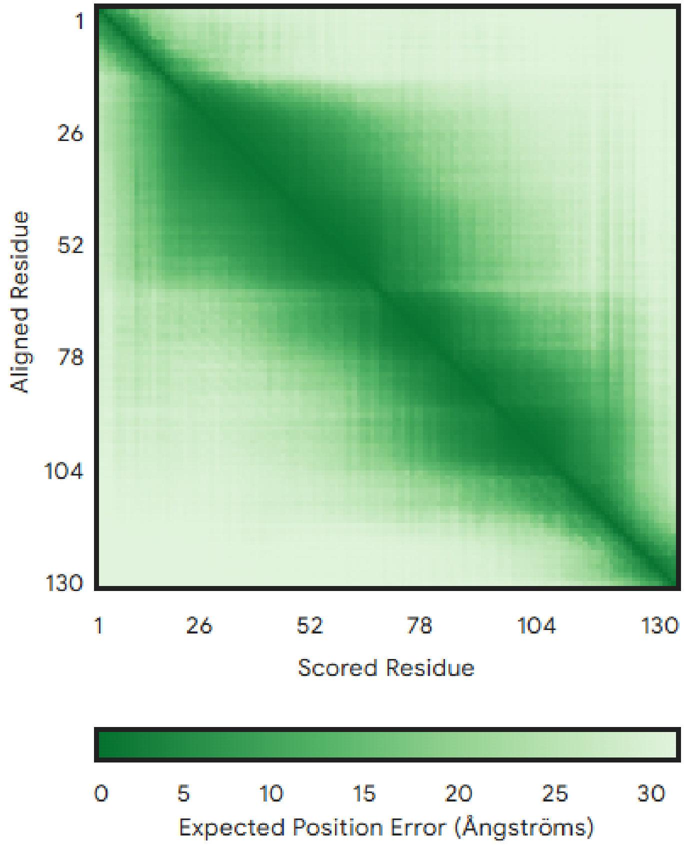

A0A2T7NUA4 *P. canaliculata*

Dimer

Very high (pIDDT > 90) Confident (90 > pIDDT > 70) Low (70 > pIDDT > 50) Very low (pIDDT < 50)

ipTM = 0.14 pTM = 0.26 learn more

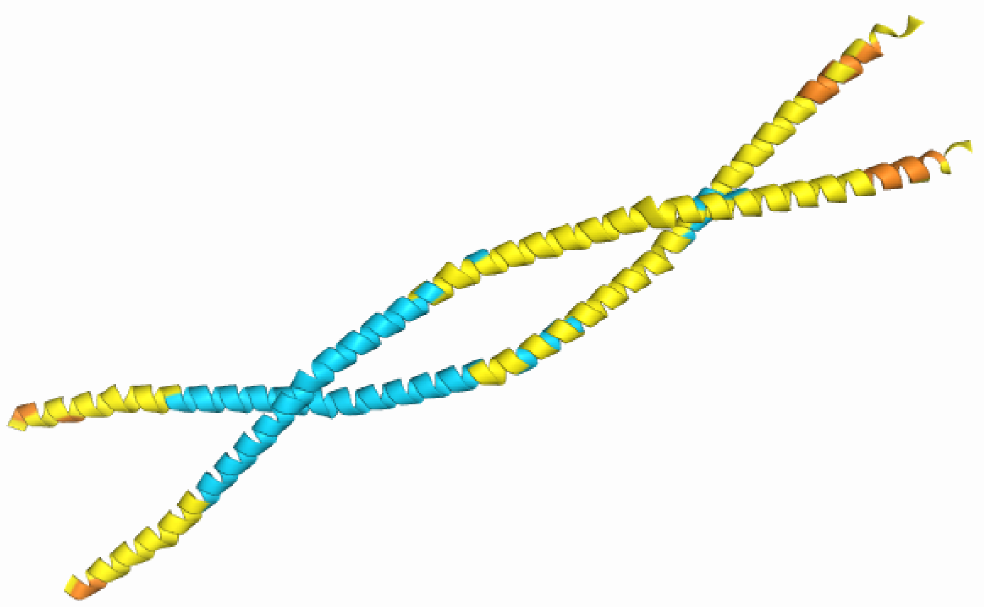

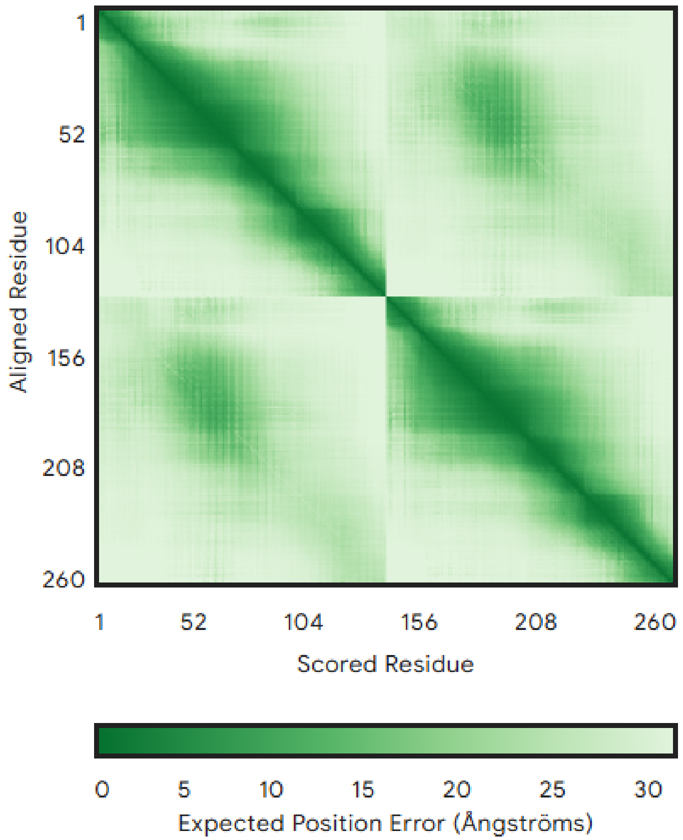

A0A2T7NUA4 ***P. canaliculata***

11-mer

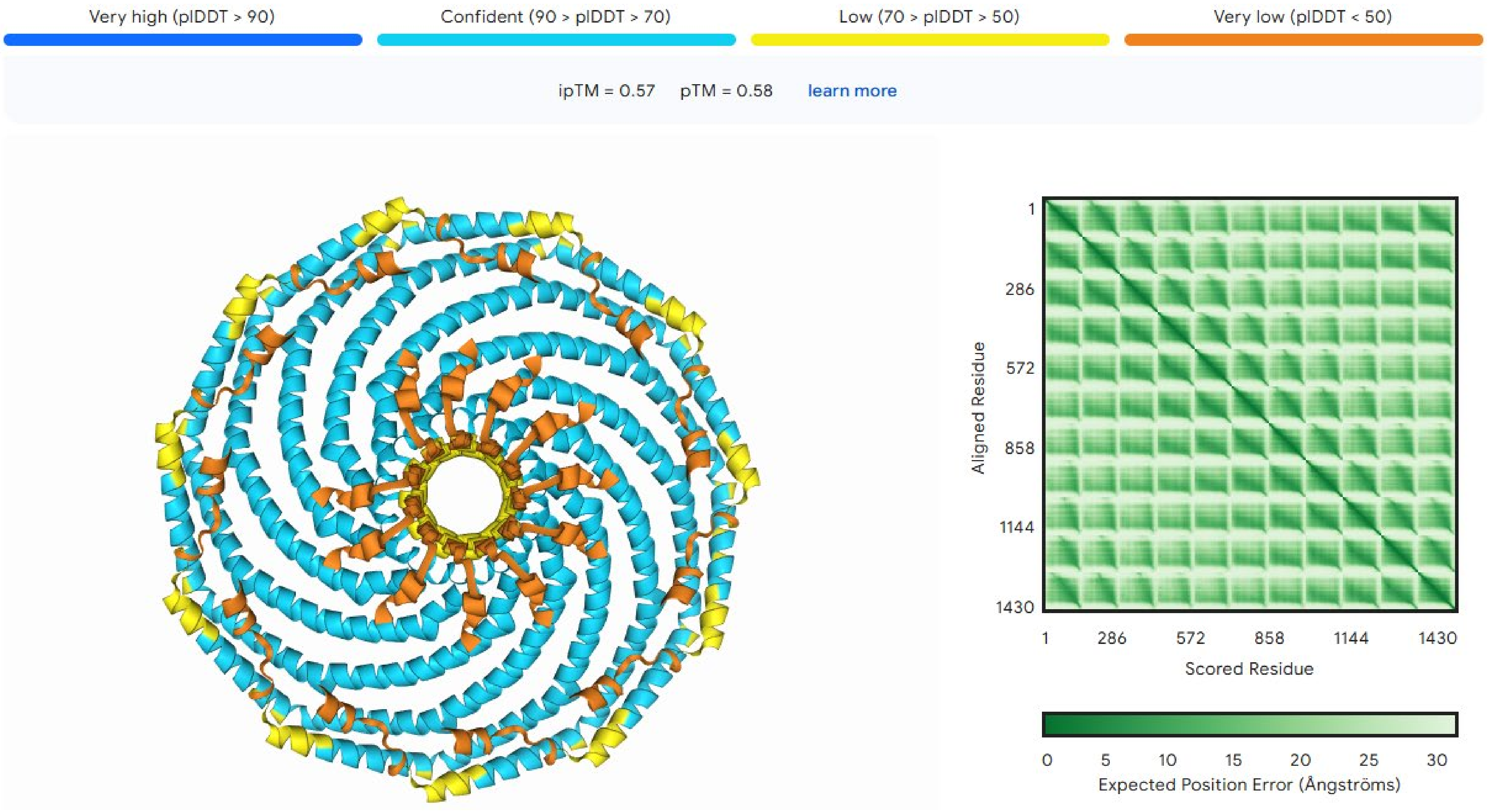

A0A1S3IG42 *L. unguis*

Monomer

Very high (pIDDT > 90) | Confident (90 > pIDDT > 70) | Low (70 > pIDDT > 50) | Very low (pIDDT < 50)

ipTM = - pTM = 0.47 learn more

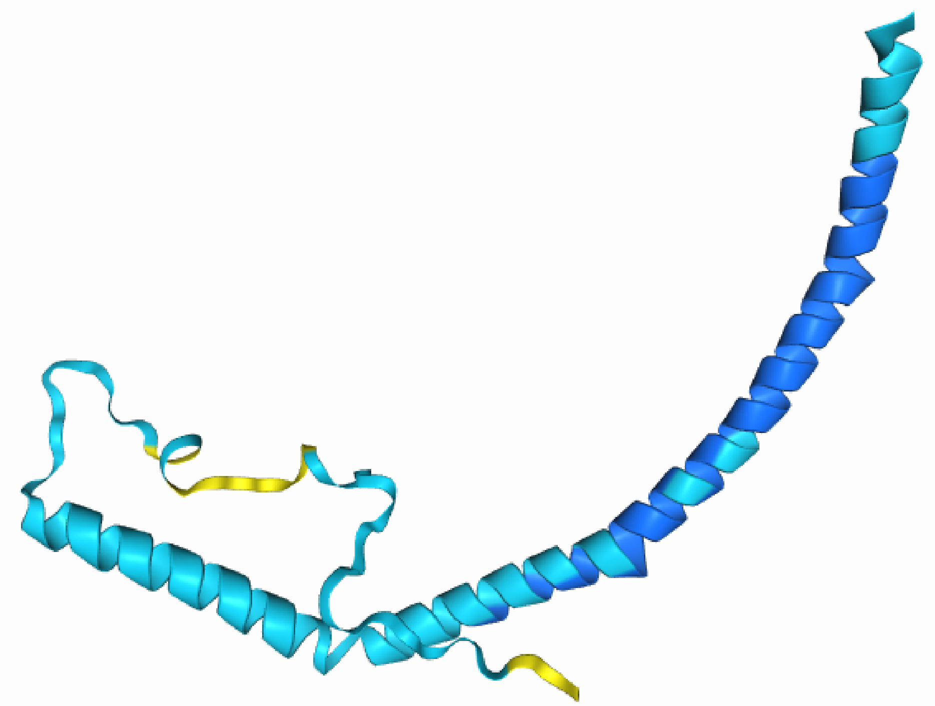

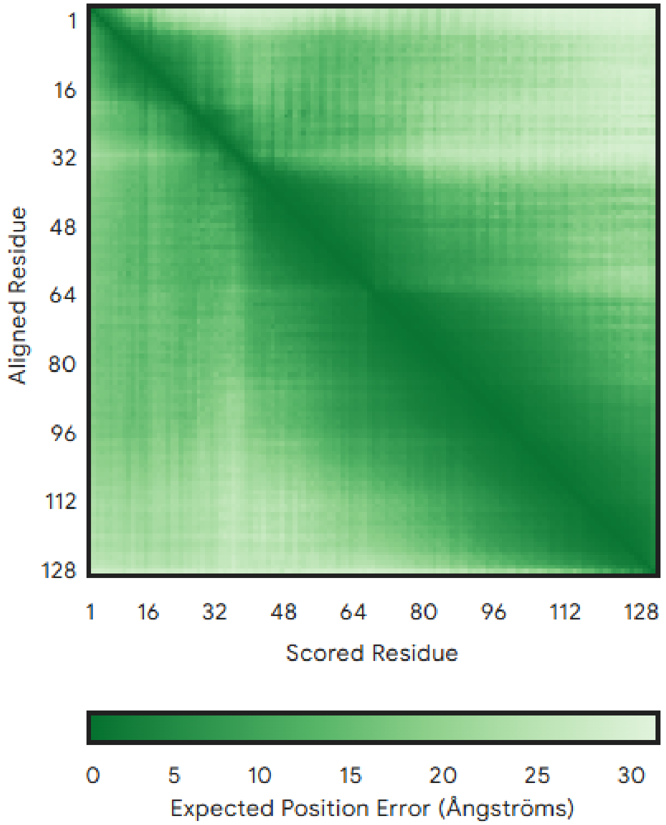

A0A1S3IG42 *L. unguis*

Dimer

Very high (pIDDT > 90) | Confident (90 > pIDDT > 70) | Low (70 > pIDDT > 50) | Very low (pIDDT < 50)

ipTM = 0.06   pTM = 0.2   learn more

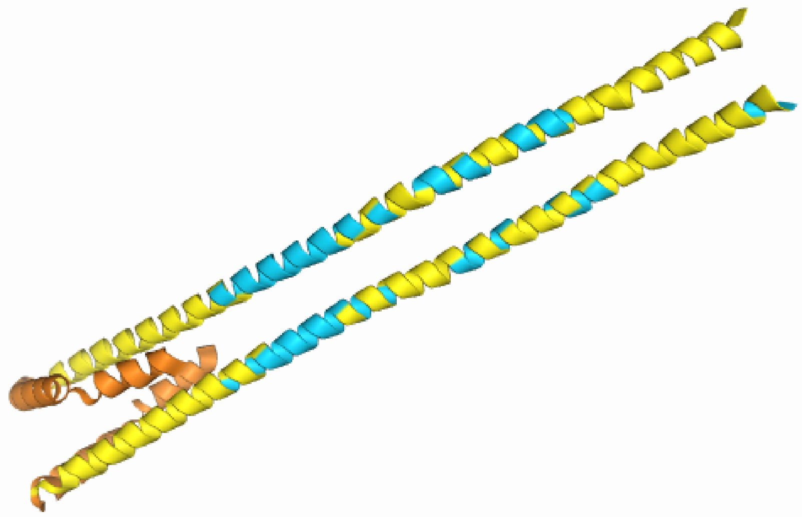

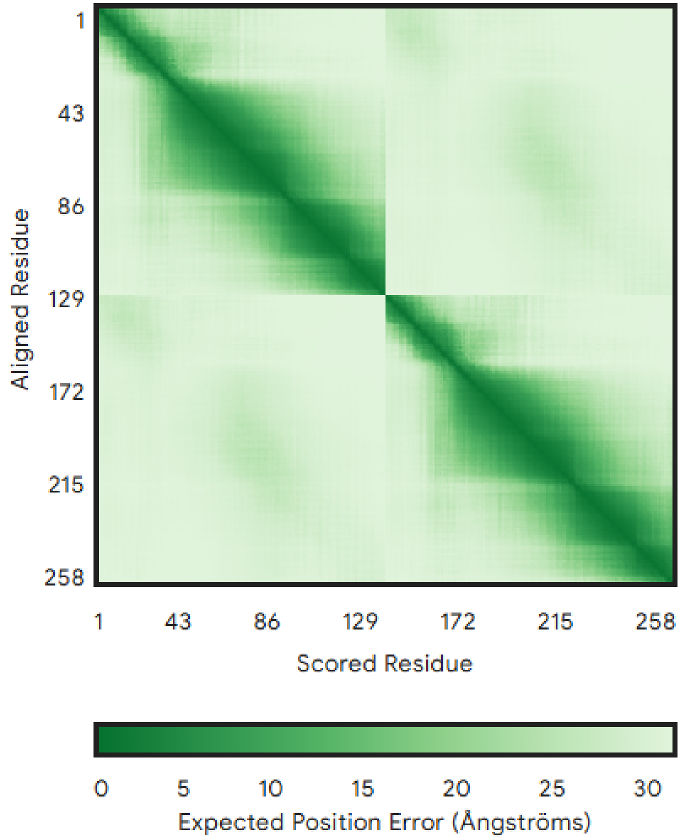

A0A1S3IG42 *L. unguis*

11-mer

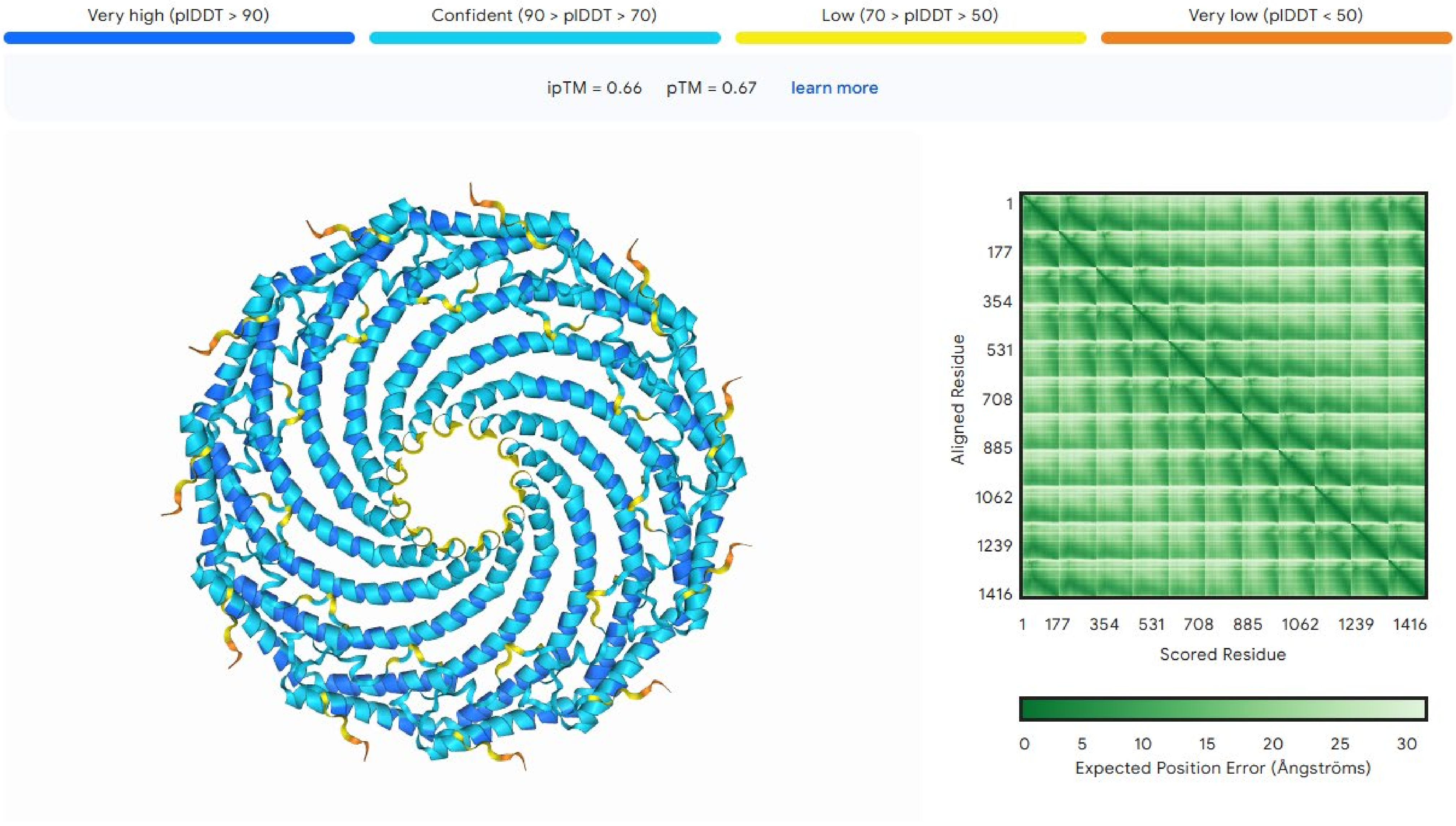

R7V531 *C. teleta*

Monomer

Very high (pIDDT > 90) | Confident (90 > pIDDT > 70) | Low (70 > pIDDT > 50) | Very low (pIDDT < 50)

ipTM = - pTM = 0.42 learn more

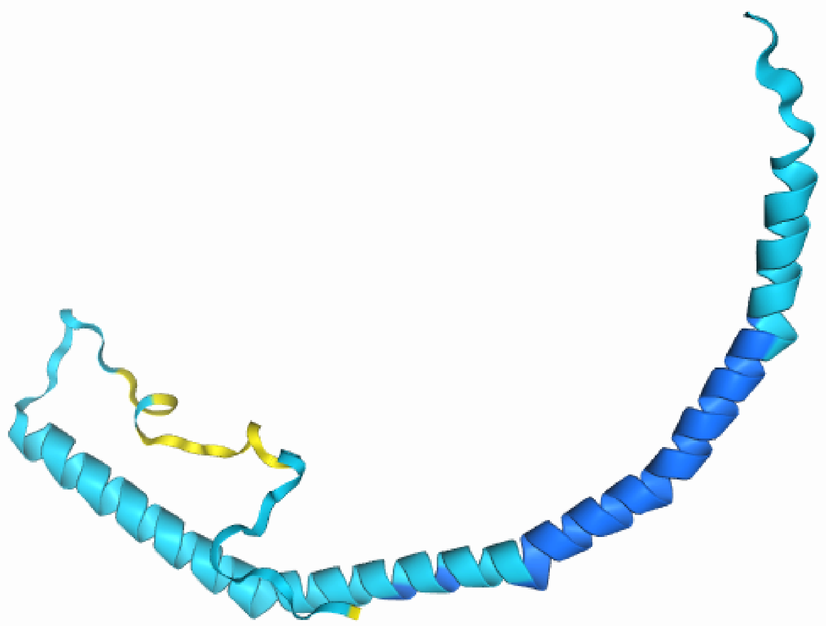

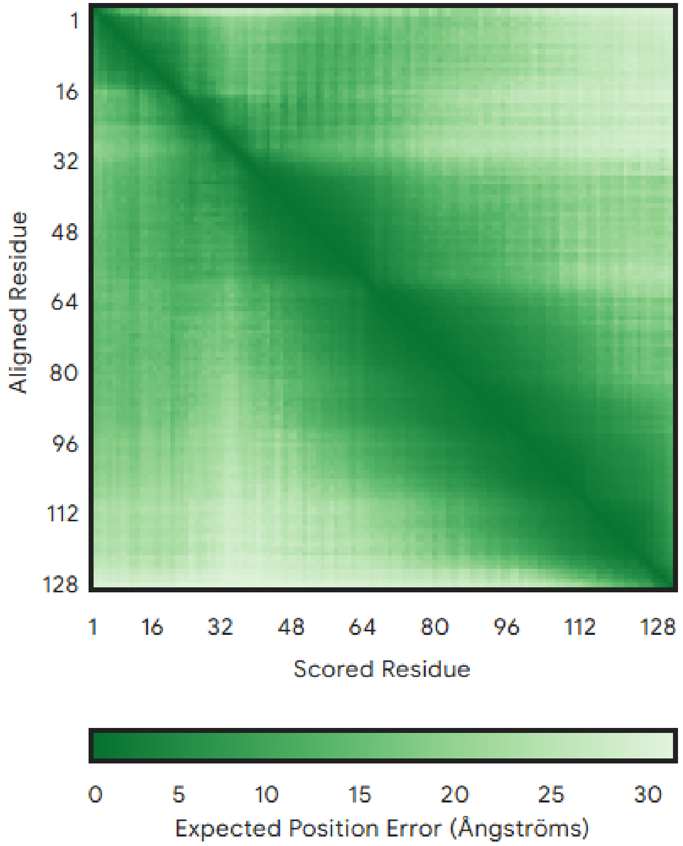

R7V531 *C. teleta*

Dimer

Very high (pIDDT > 90) | Confident (90 > pIDDT > 70) | Low (70 > pIDDT > 50) | Very low (pIDDT < 50)

ipTM = 0.05 pTM = 0.21 learn more

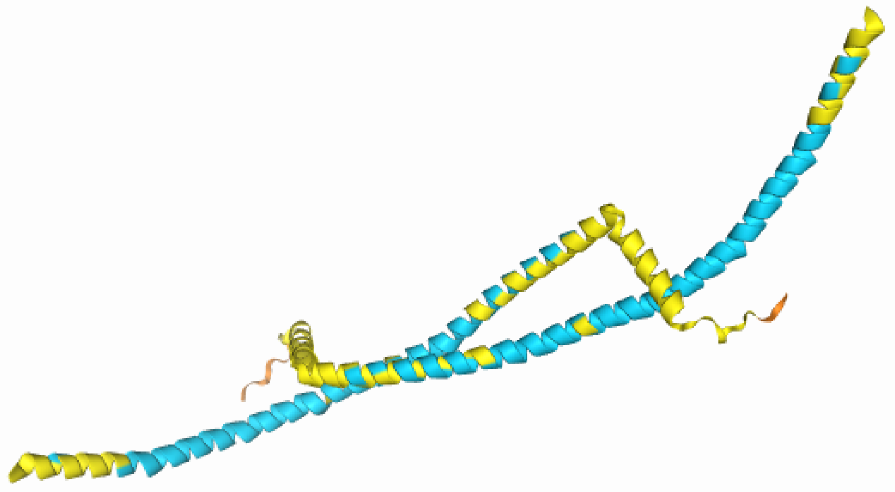

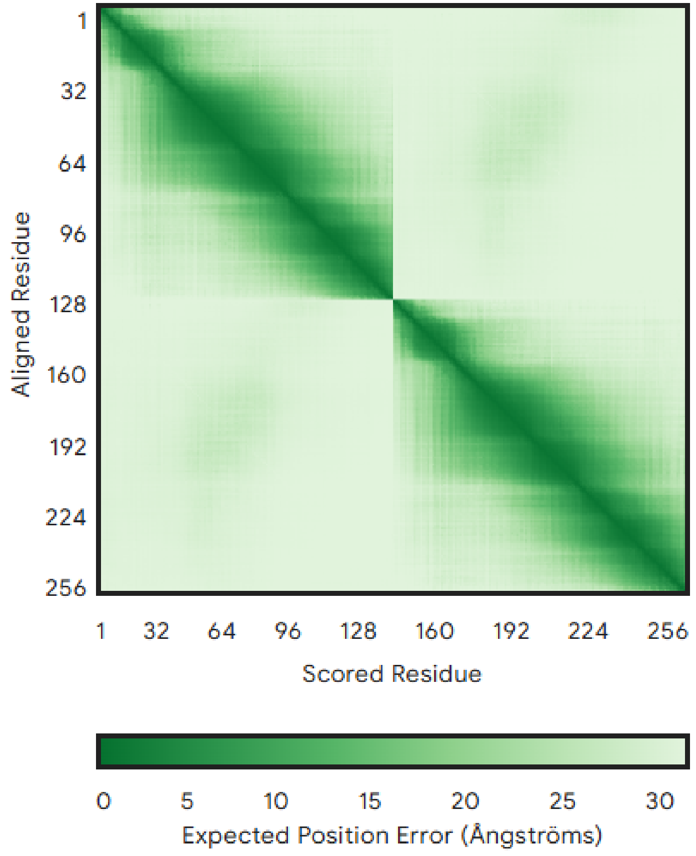

R7V531 *C. teleta*

11-mer

Very high (pIDDT > 90) | Confident (90 > pIDDT > 70) | Low (70 > pIDDT > 50) | Very low (pIDDT < 50)

ipTM = 0.65 pTM = 0.67 learn more

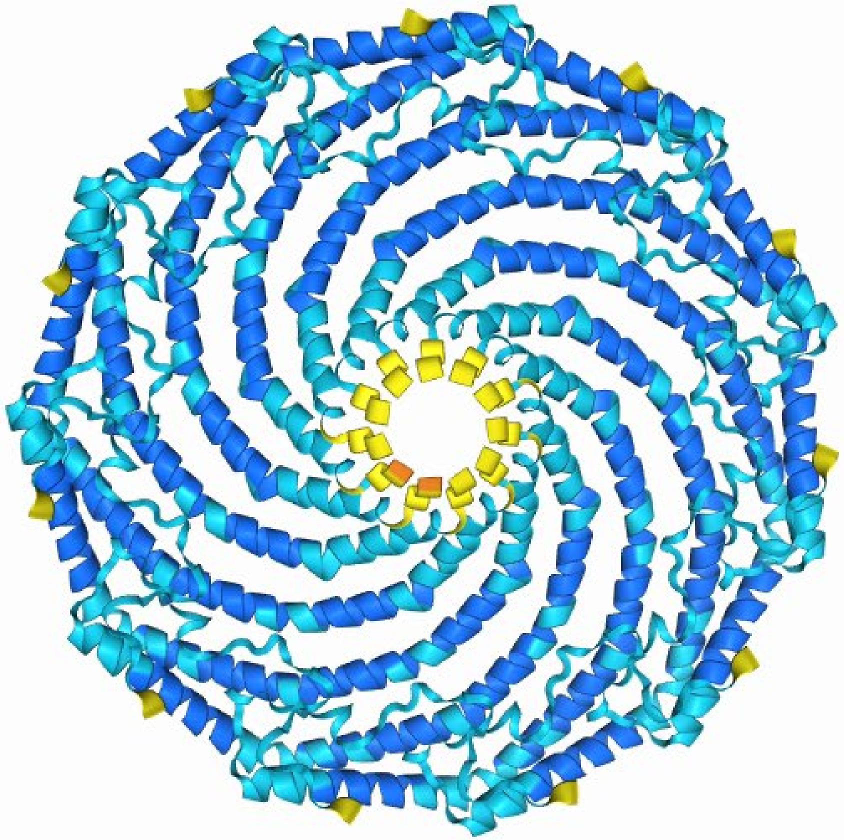

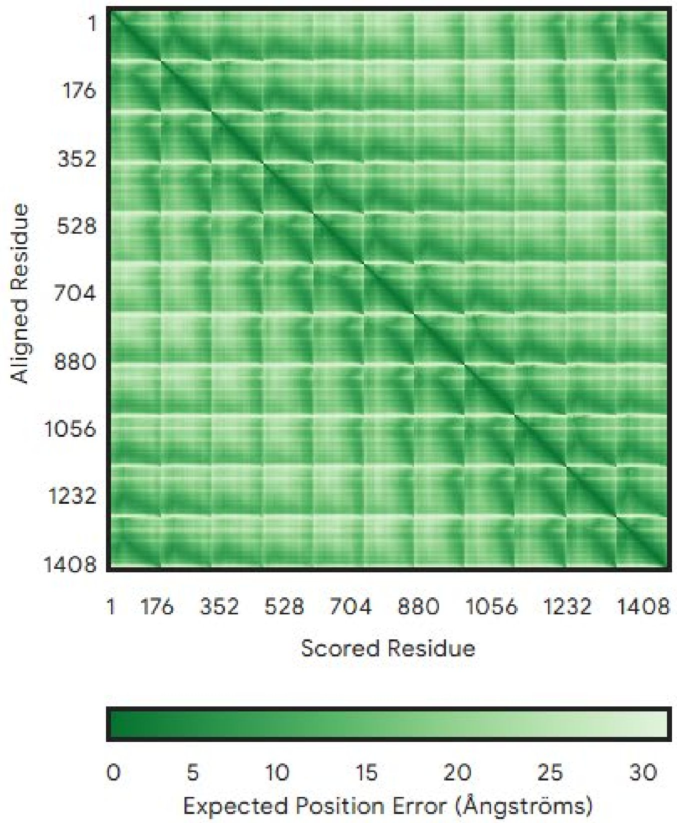

Supplement: Data S5 — shows predicted structures of select caveolin monomer, dimers, and 11-mers using AlphaFold3. [file jcb_202411175_datas5.pdf]
