# Supplementary material for: Evolutionarily diverse caveolins share a common structural framework built around amphipathic disks
Source: J Cell Biol. 2025 Aug 7;224(9):e202411175. doi: 10.1083/jcb.202411175 (PMC12330381; doi:10.1083/jcb.202411175)

File S6. Comparison of AlphaFold prediction results for select caveolins across different AlphaFold versions.

Note:

Models were all colored by pLDDT values as below color key indicated:

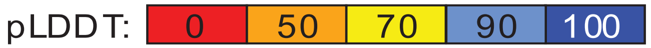

F2U793 *S. Rosetta*

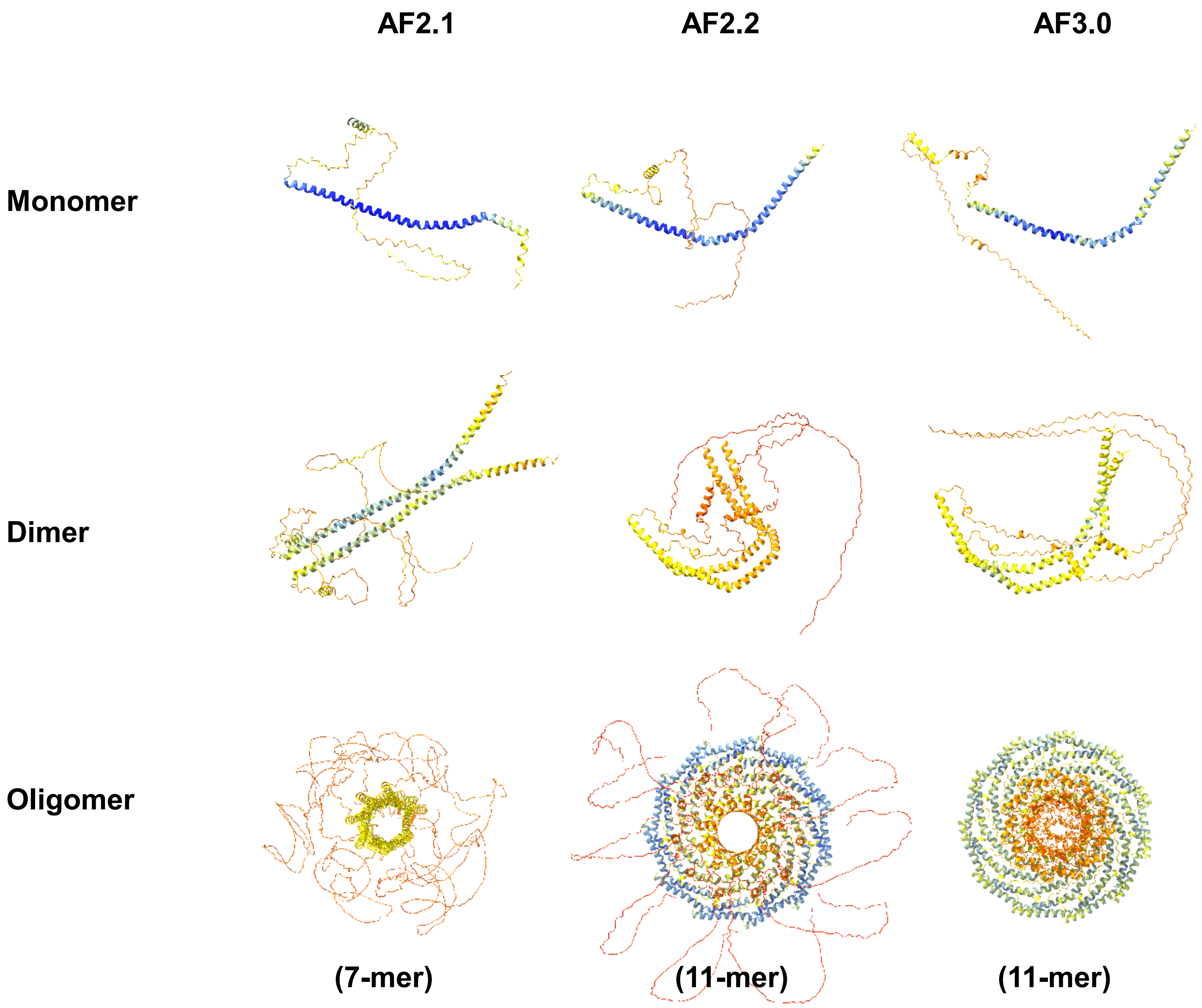

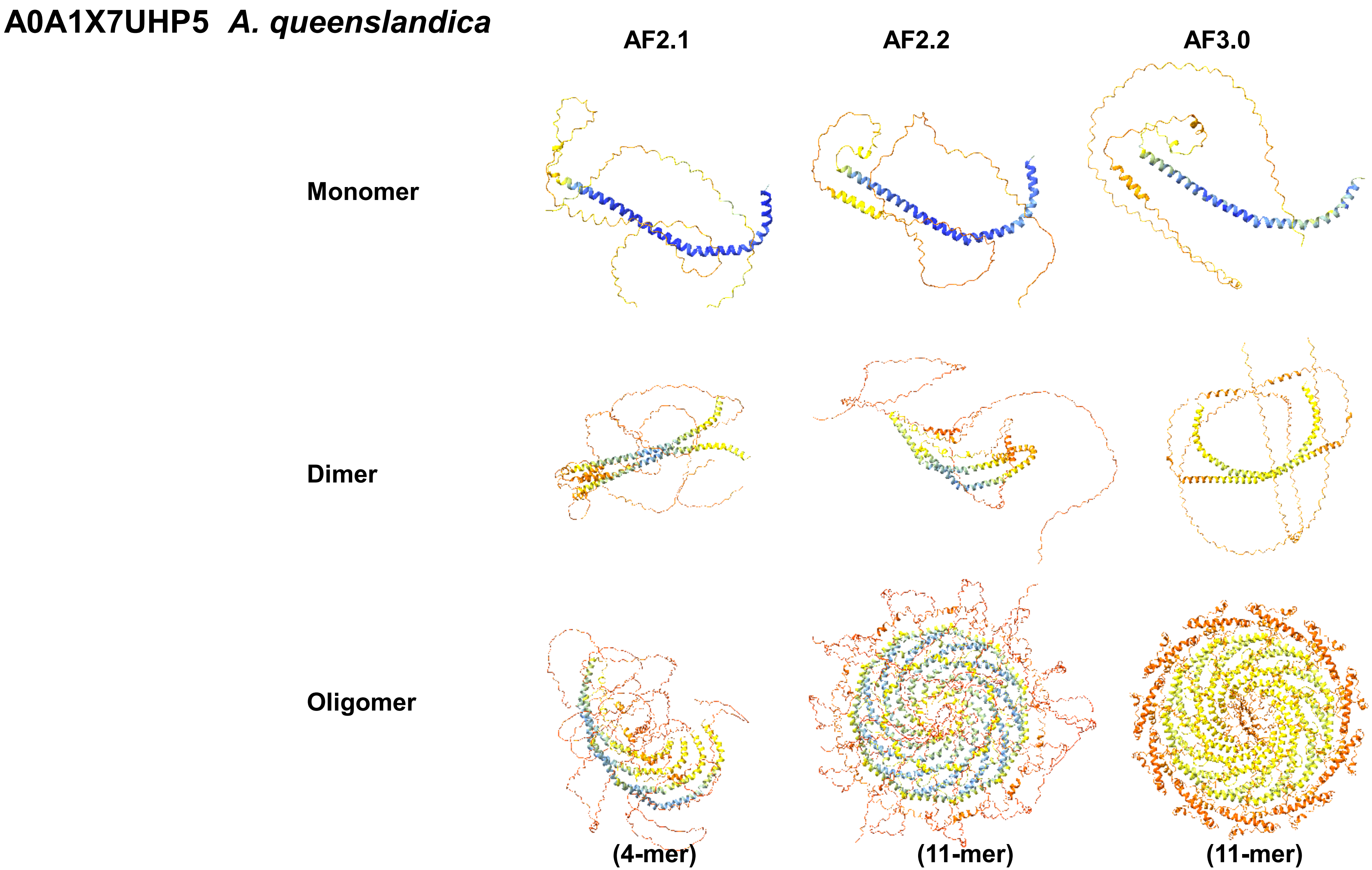
A0A1X7UHP5 A. queenslandica
AF2.1
AF2.2
AF3.0
Monomer
Dimer
Oligomer
(4-mer)
(11-mer)
(11-mer)

B3RWV2 *T. adhaerens*

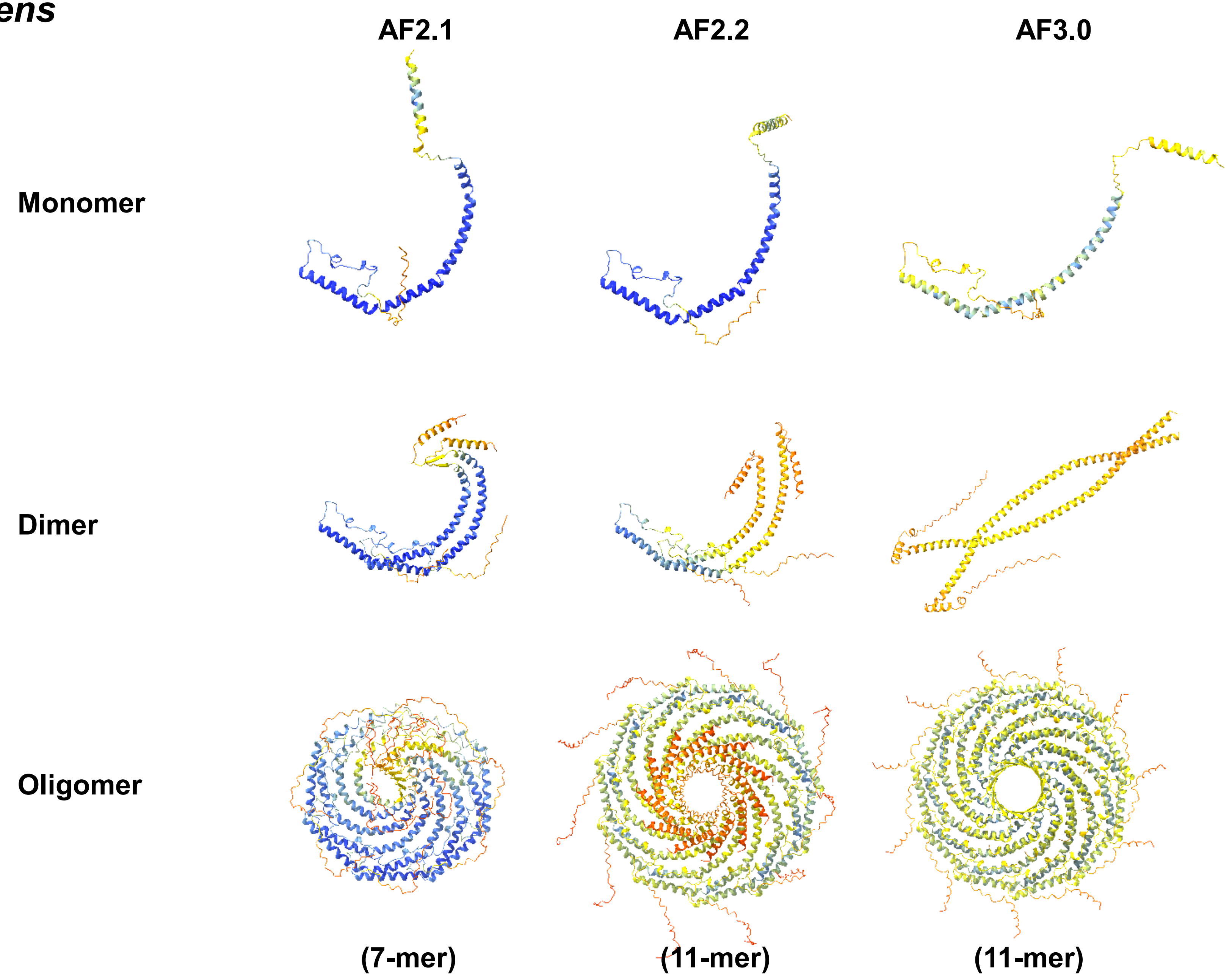

A0A2B4SAZ9 *S. pistillata*

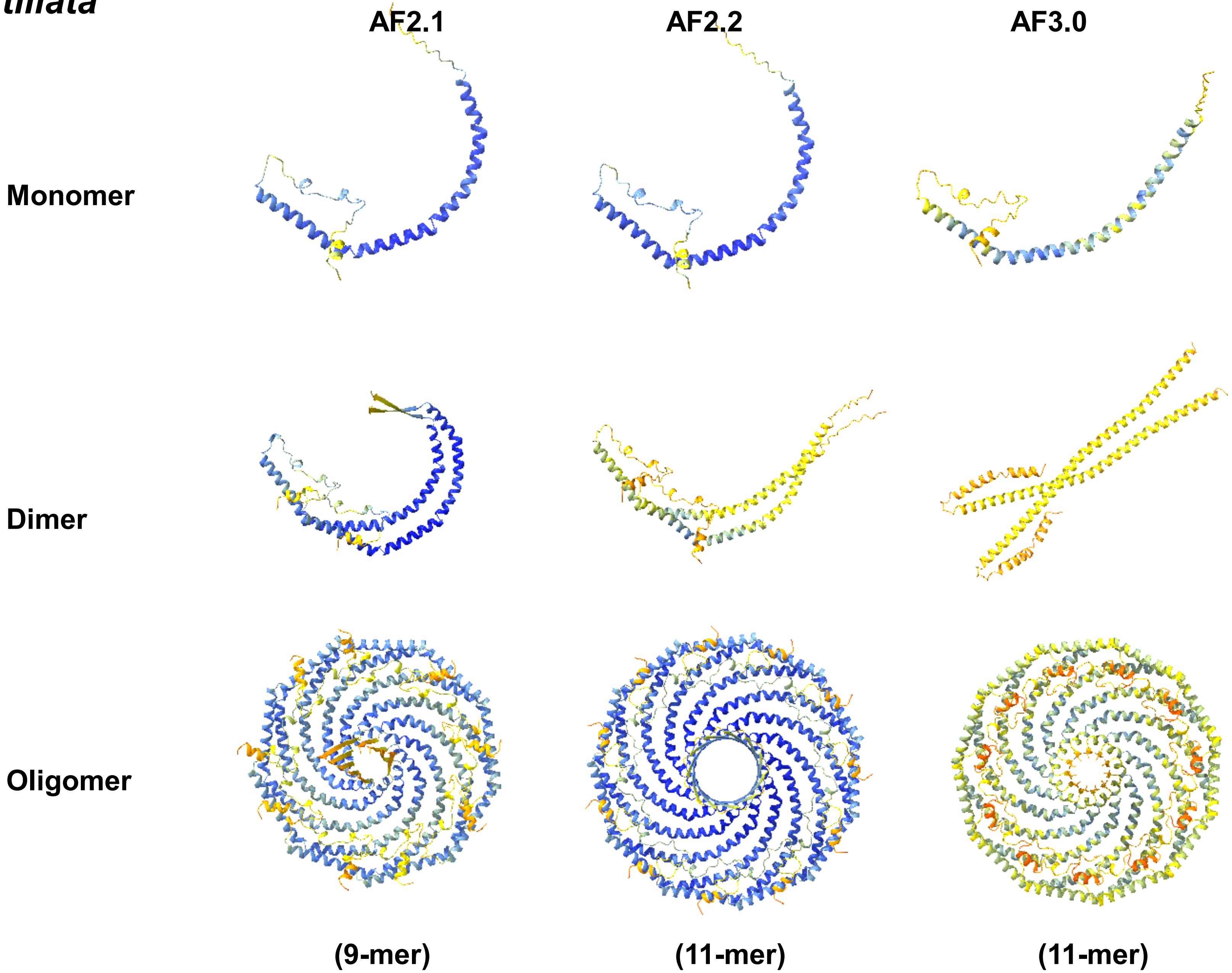

scaffold217_2 *Xenoturbella bocki*

AF2.1 **AF2.2** **AF3.0**

Monomer

Dimer

Oligomer

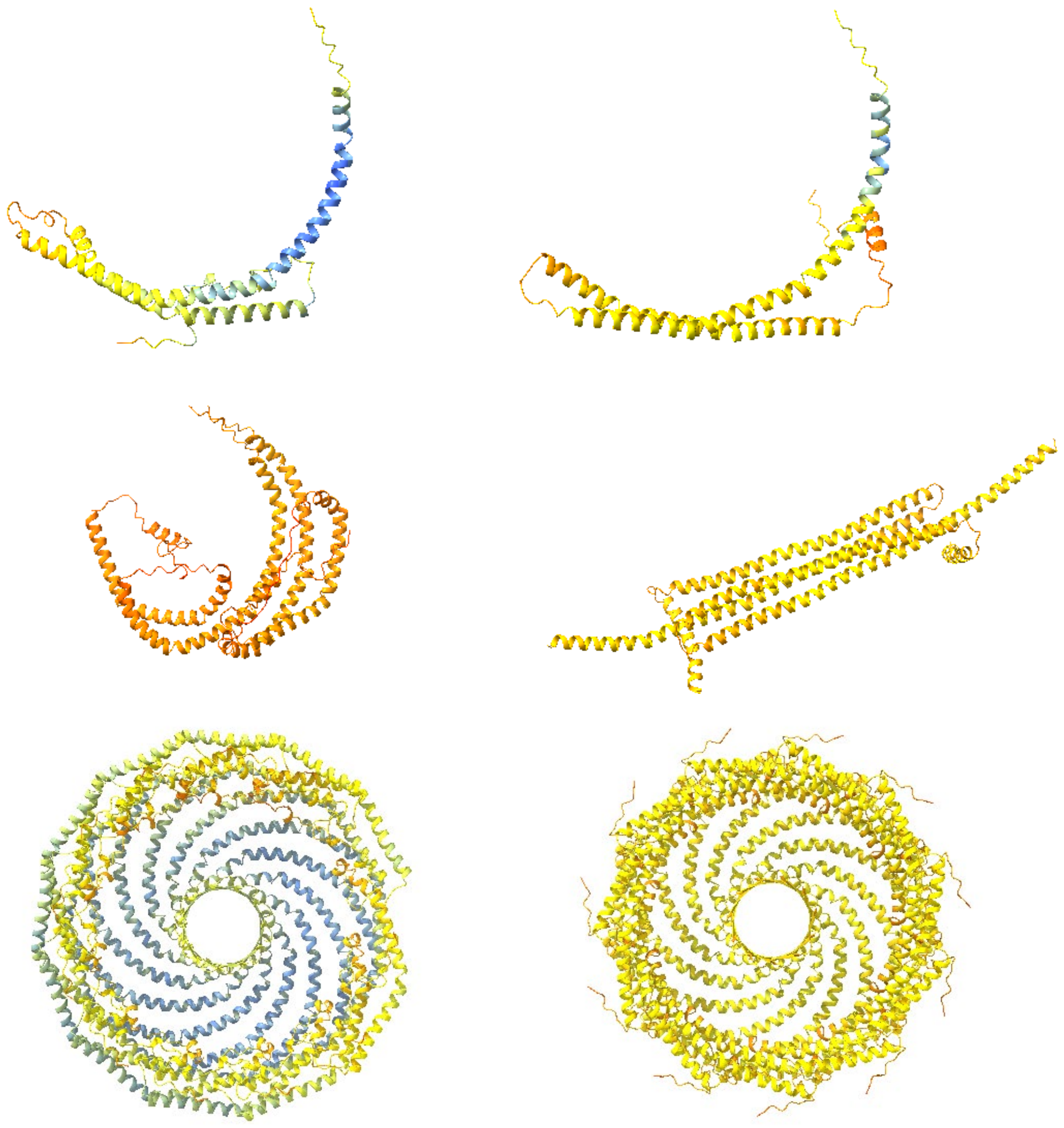

(11-mer) **(11-mer)**

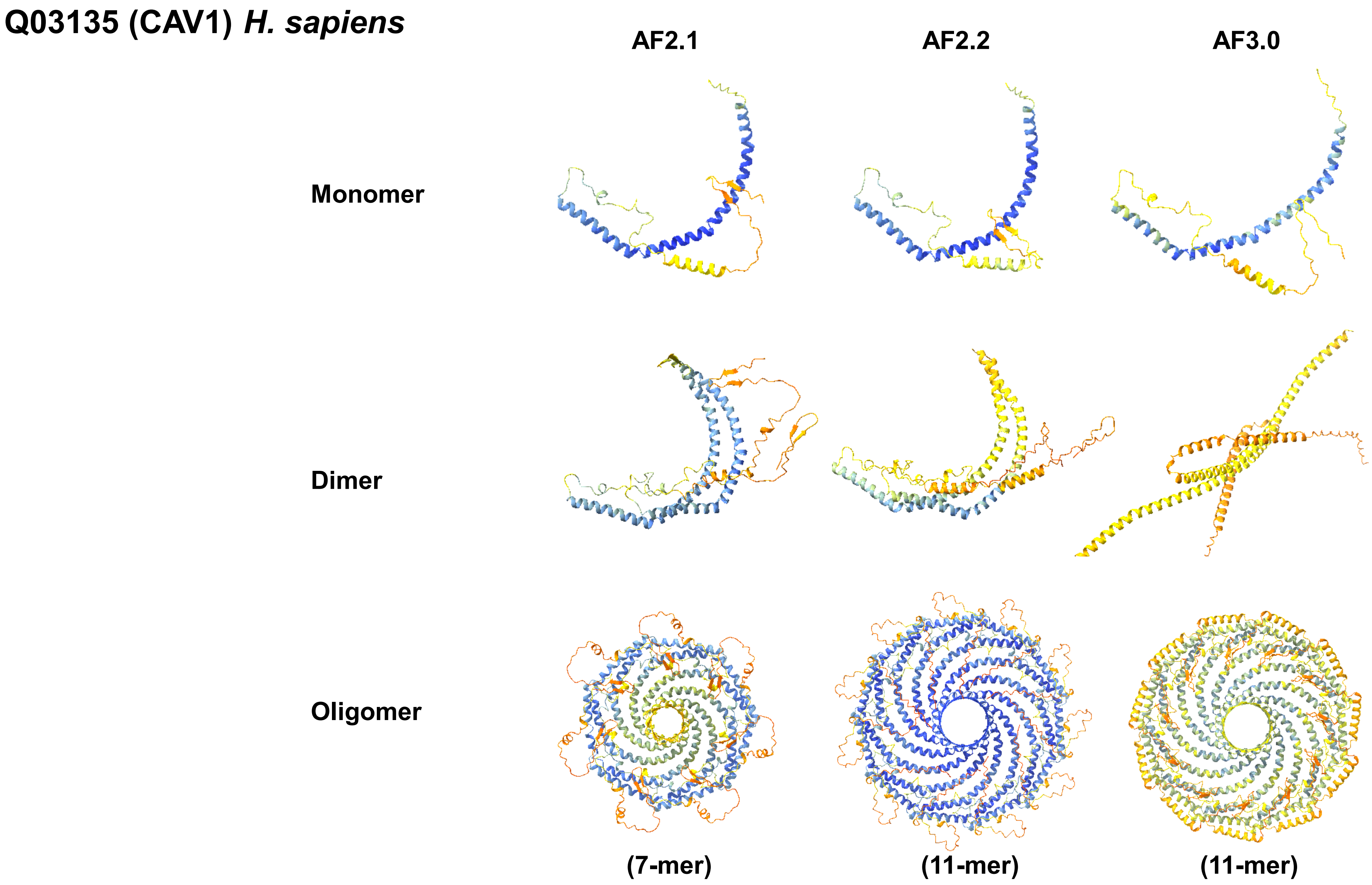
Q03135 (CAV1) *H. sapiens*
AF2.1
AF2.2
AF3.0
Monomer
Dimer
Oligomer
(7-mer)
(11-mer)
(11-mer)

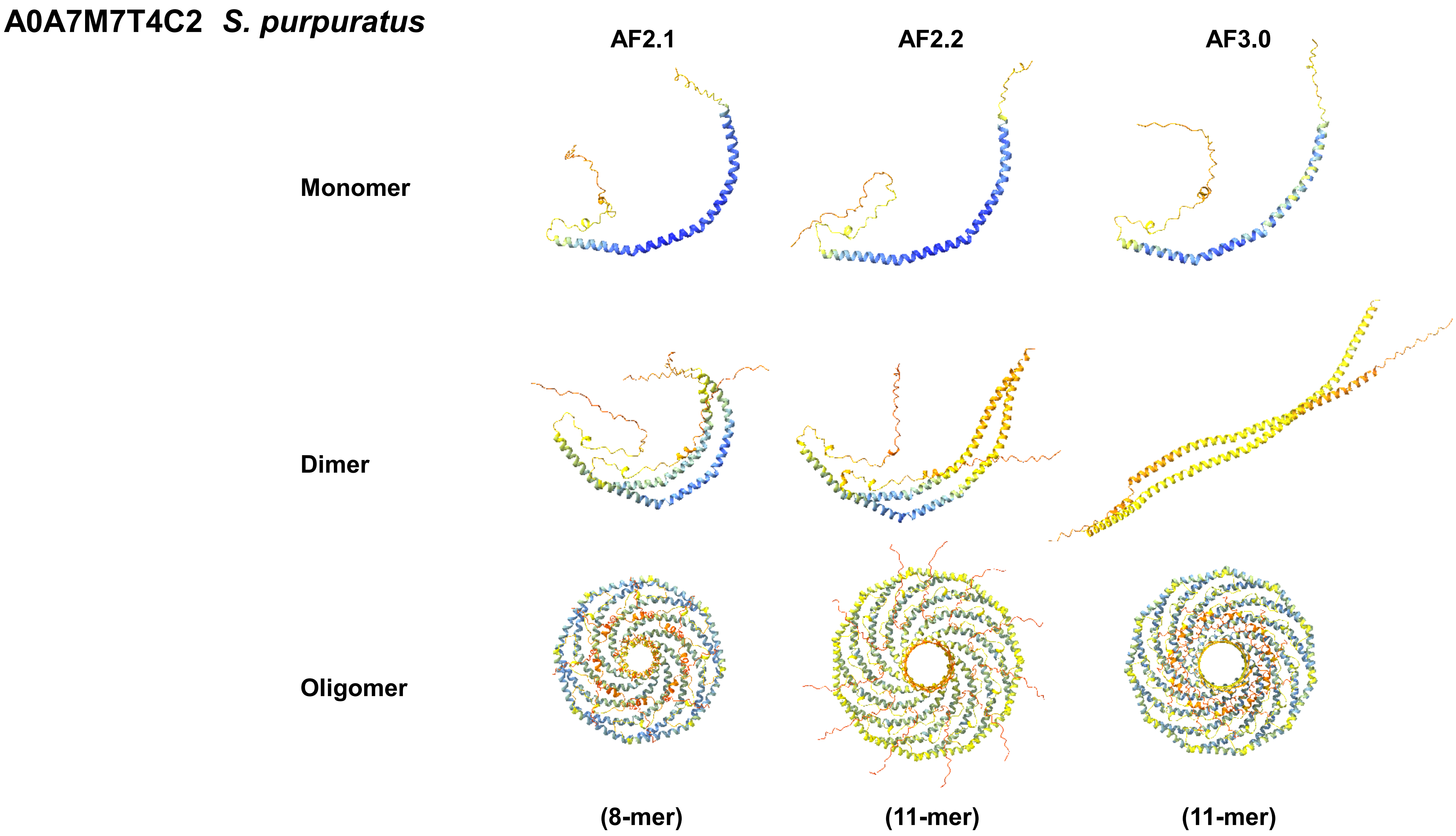
A0A7M7T4C2 *S. purpuratus*
AF2.1
AF2.2
AF3.0
Monomer
Dimer
Oligomer
(8-mer)
(11-mer)
(11-mer)

XP_014678552.1 *P. caudatus*

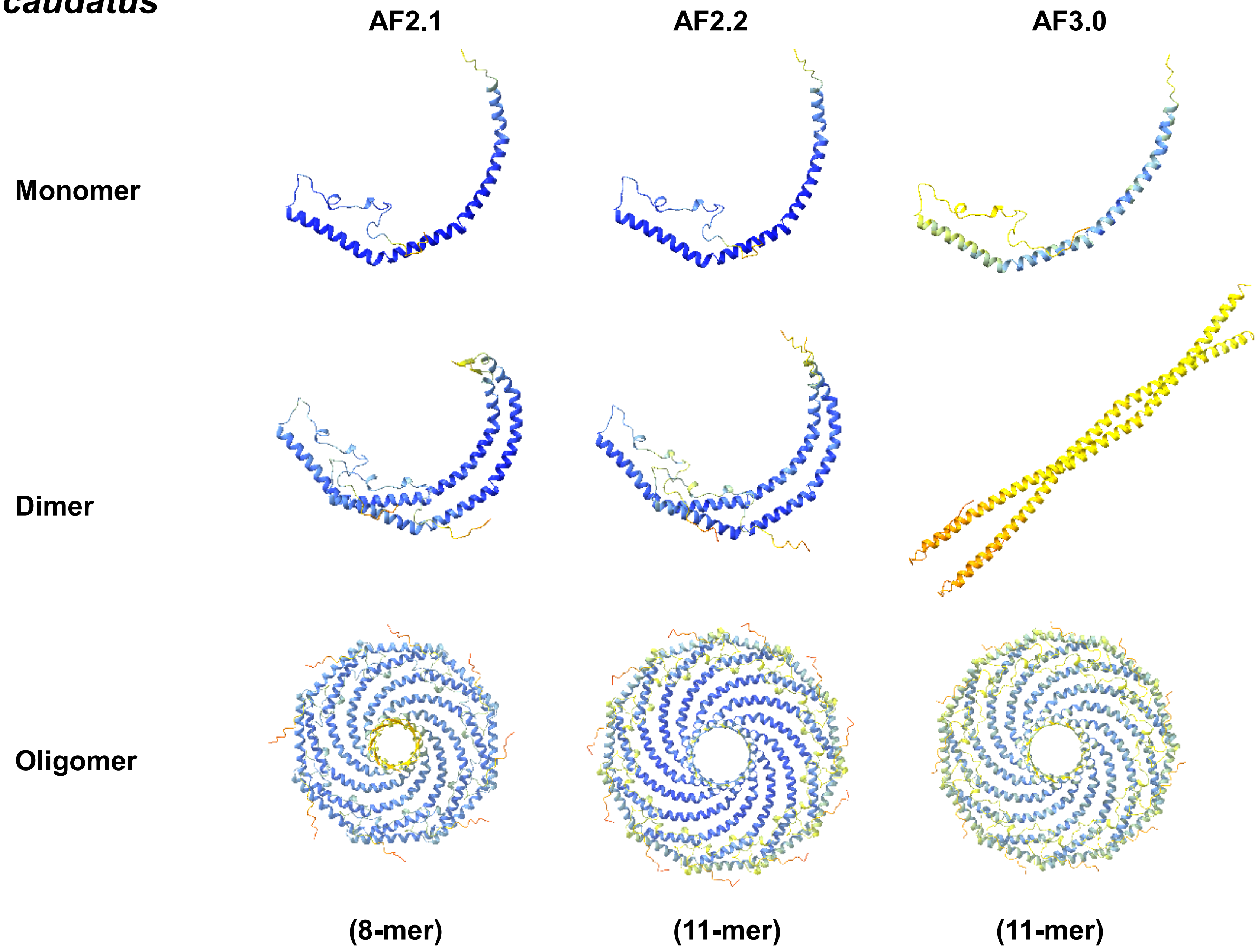

A0A7M7R2L2 *A. mellifera*

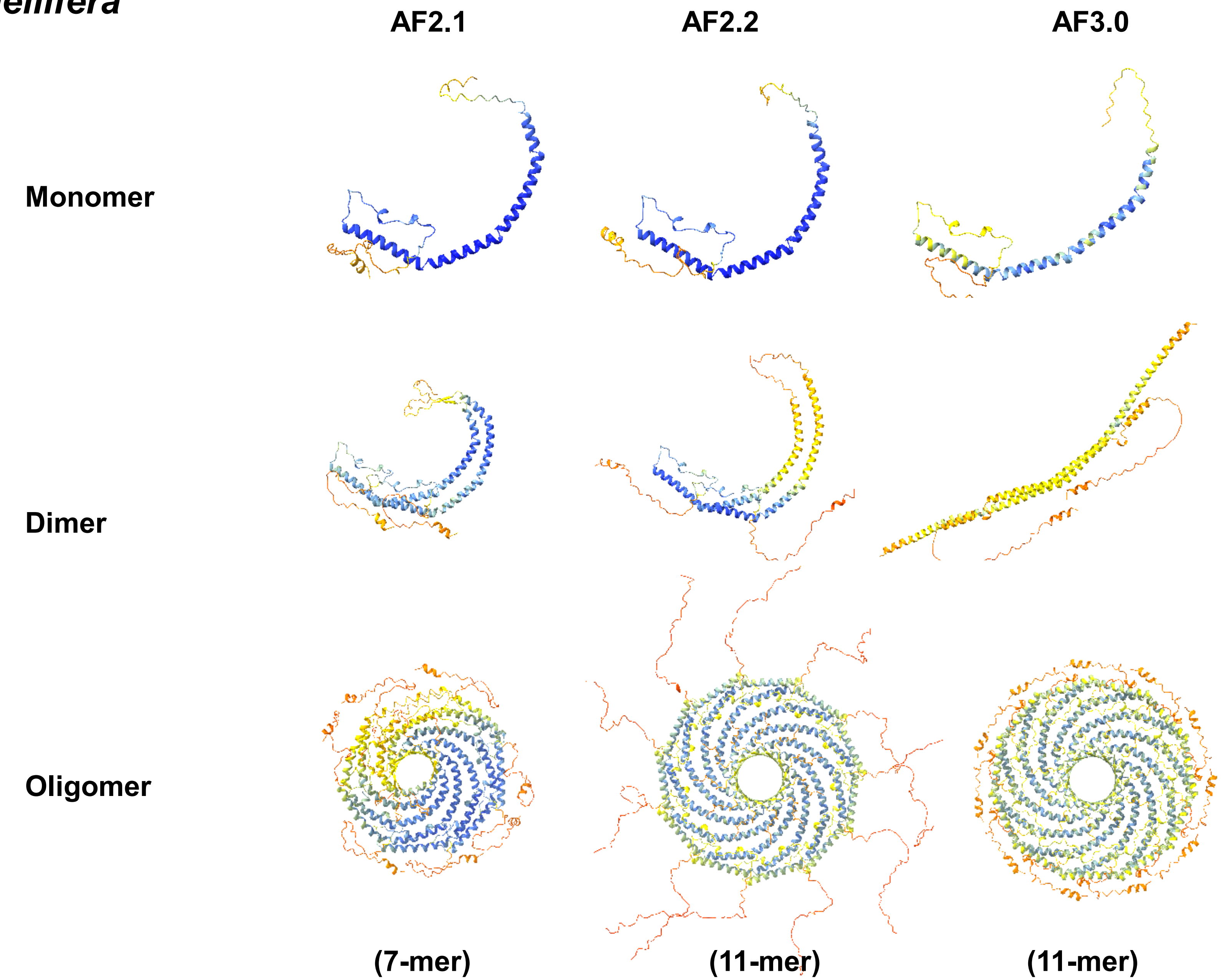

Q18879 *C. elegans*

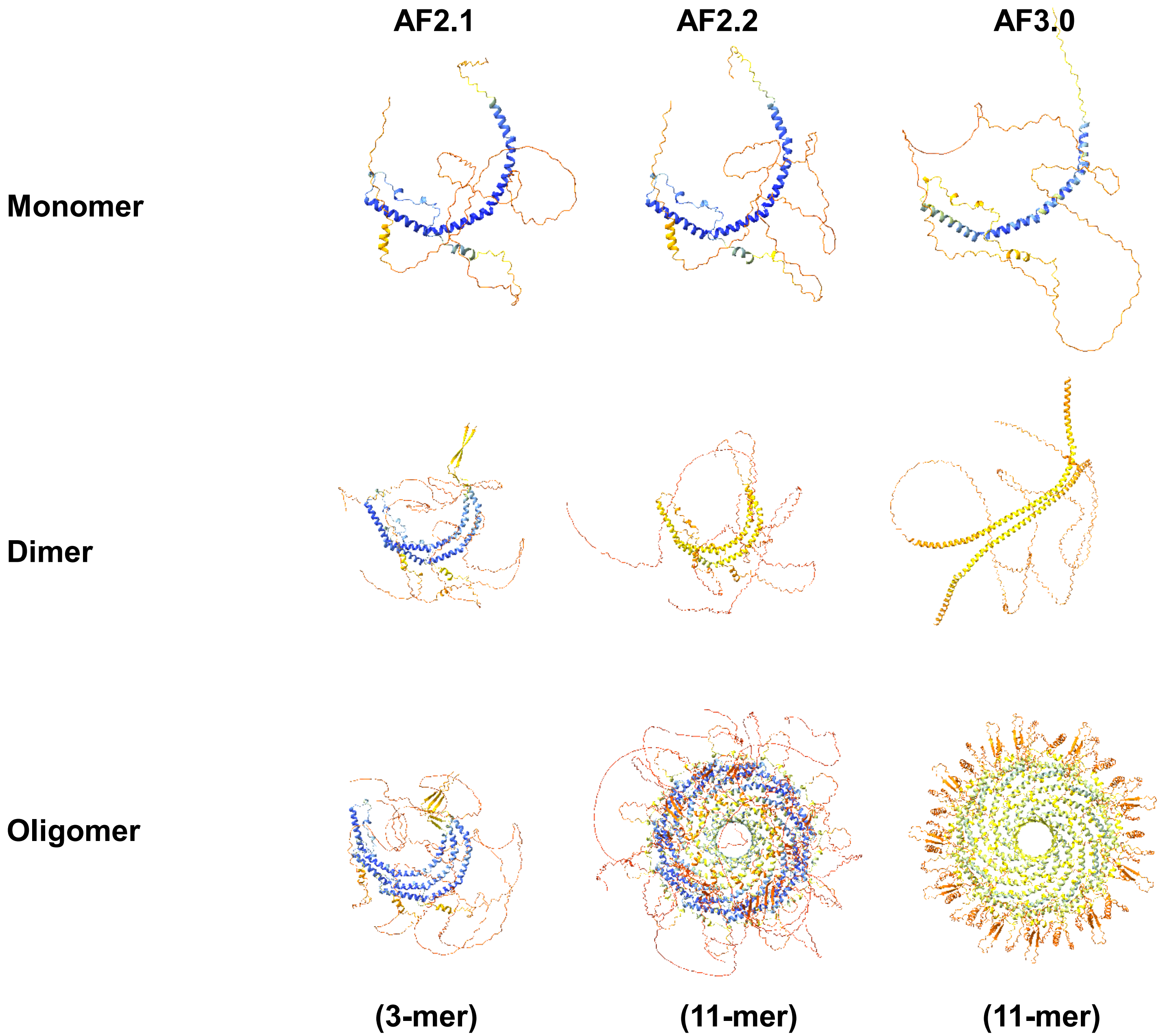

A0A3M7RD35 *B. plicatilis*

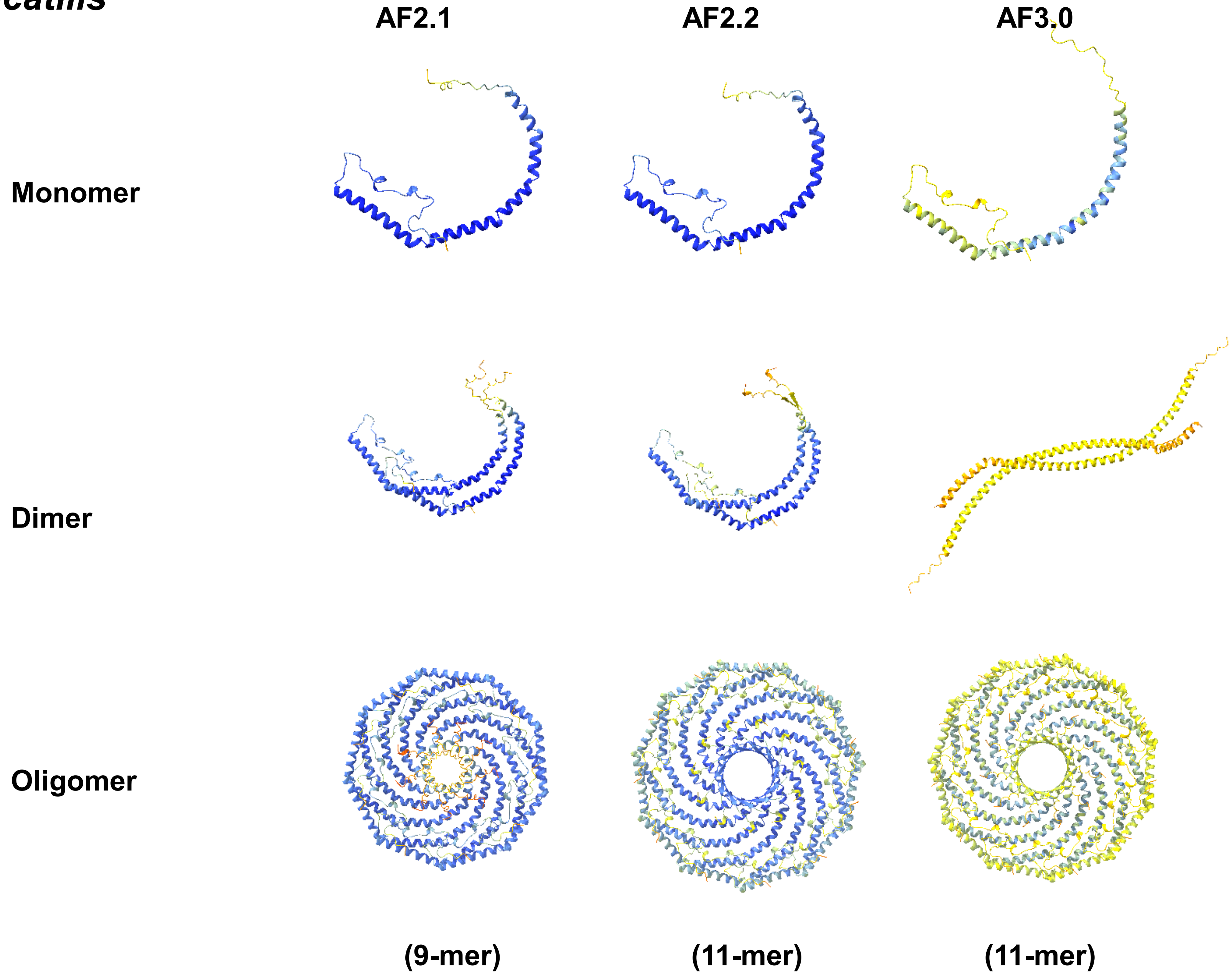

A0A267DC90 *M. lignano*

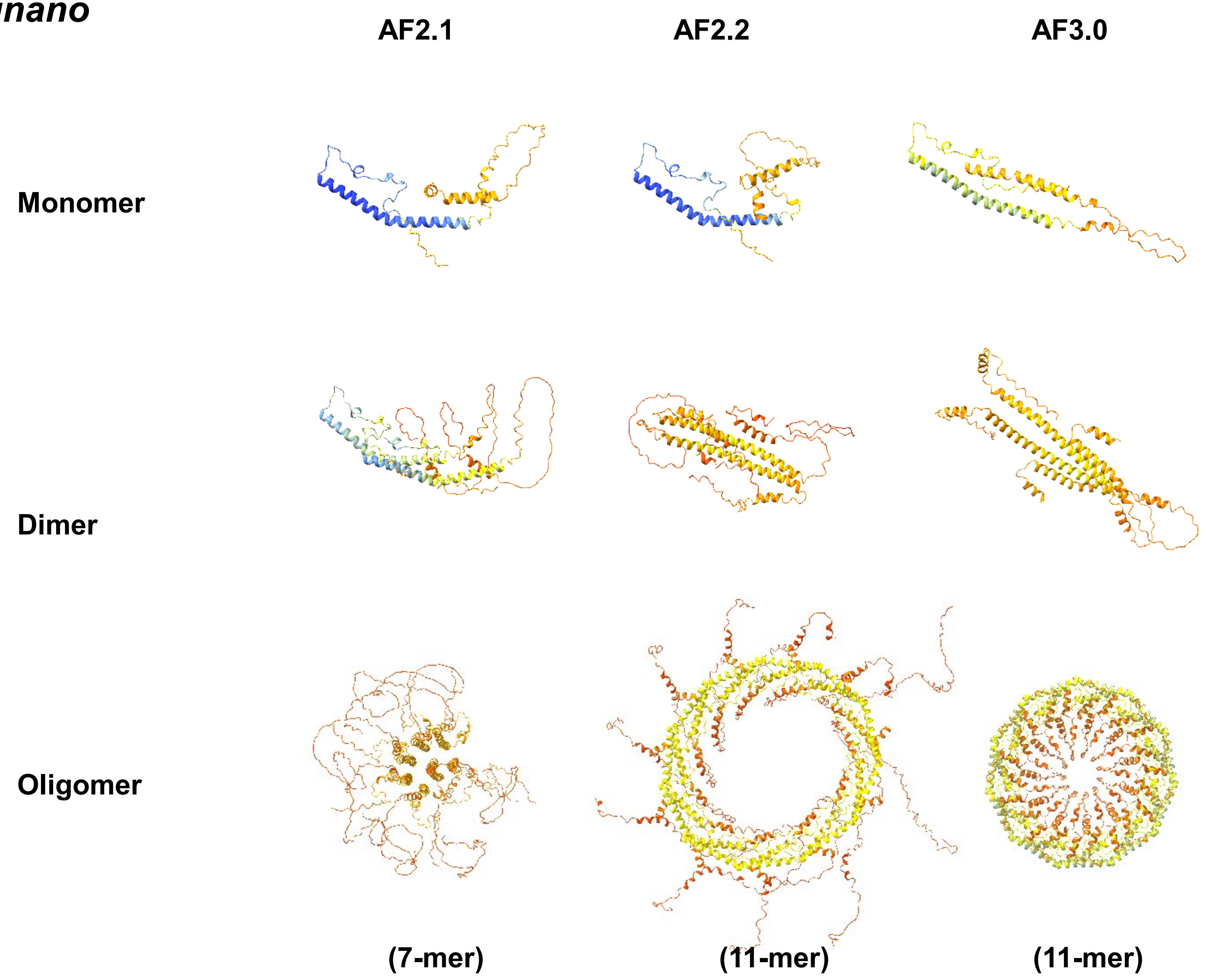

A0A2T7NUA4 *P. canaliculata*

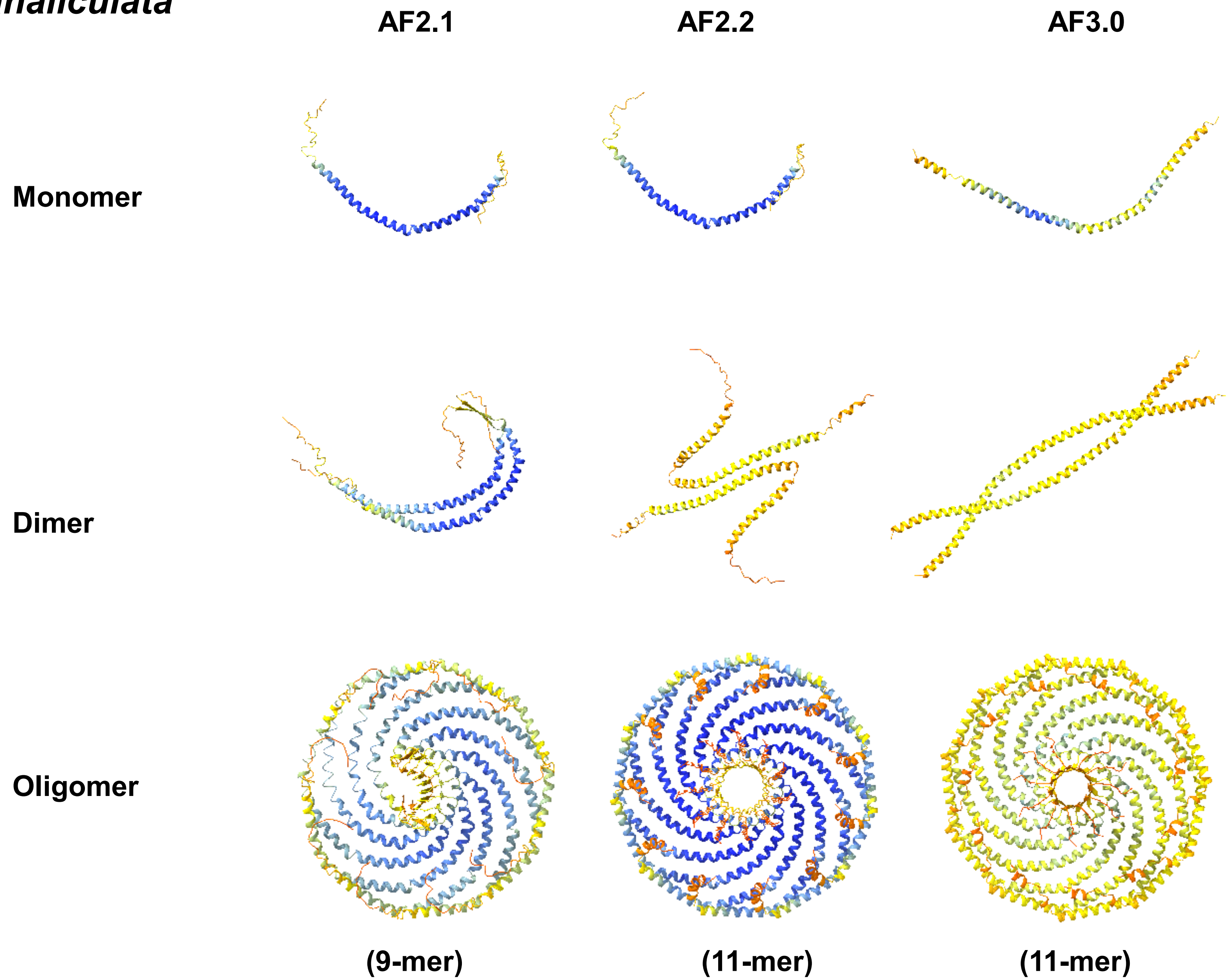

A0A1S3IG42 *L. unguis*

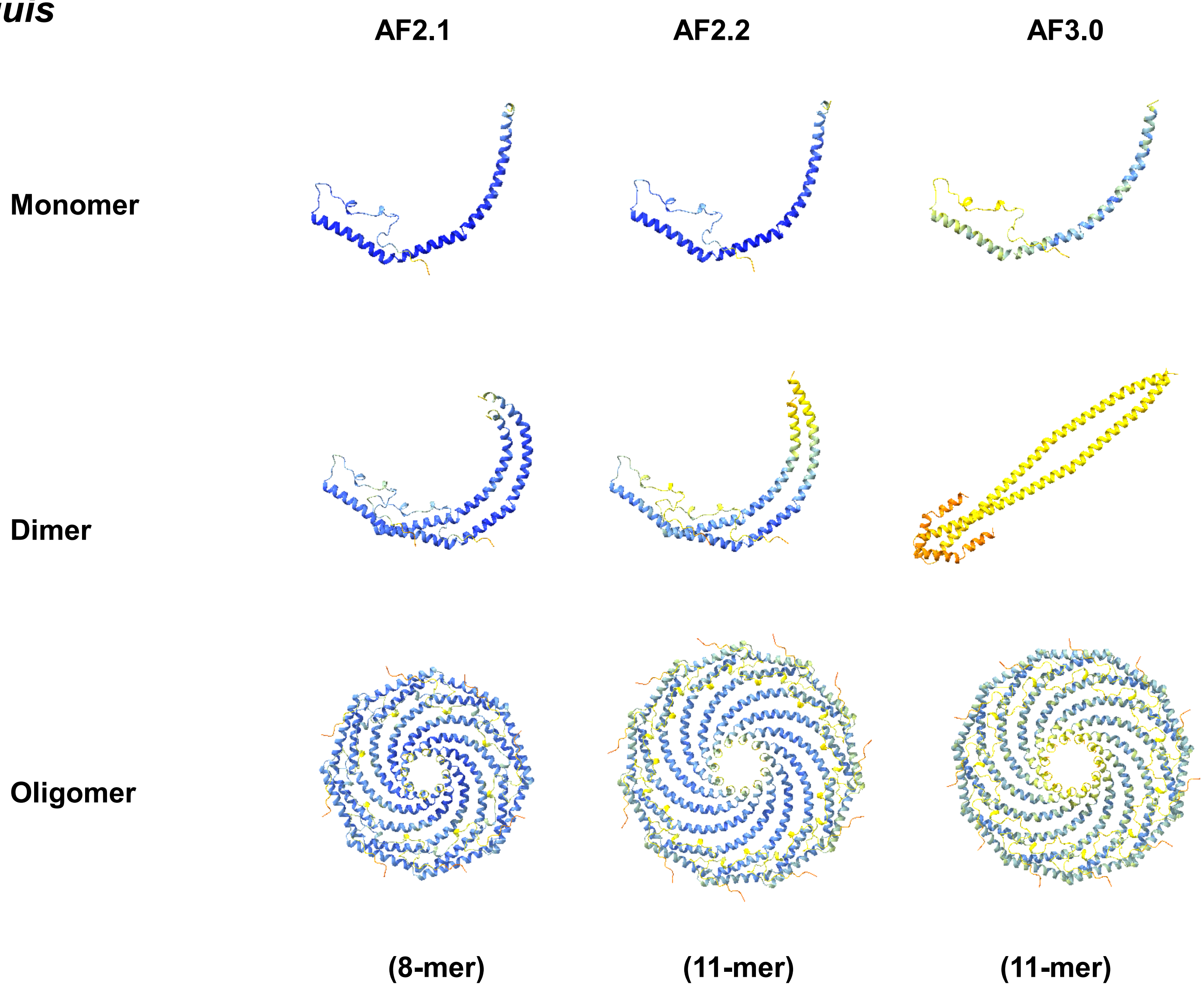

R7V531 *C. teleta*

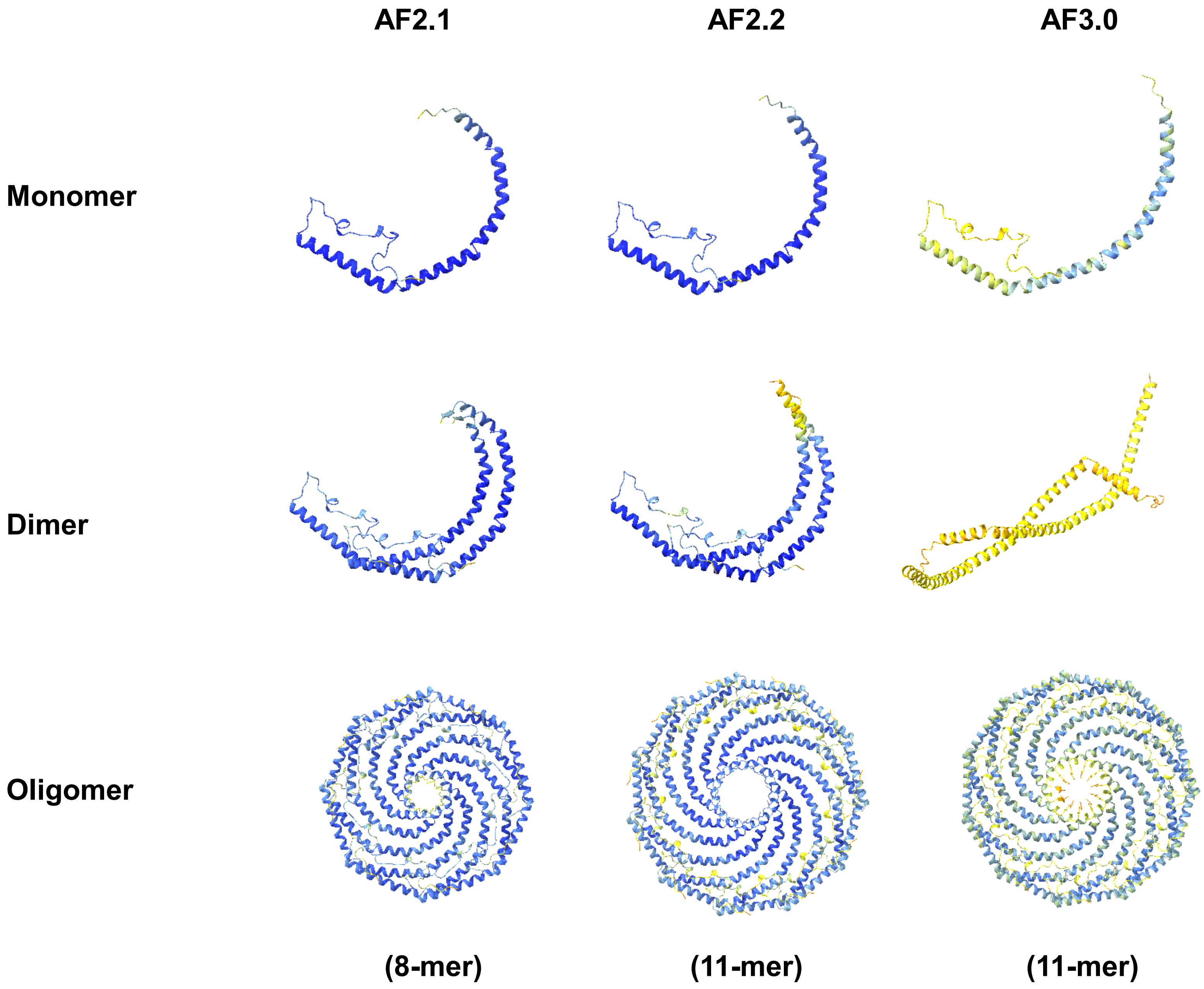

Supplement: Data S6 — shows comparison of AlphaFold prediction results for select caveolins across different AlphaFold versions. [file jcb_202411175_datas6.pdf]
