# Supplementary material for: Evolutionarily diverse caveolins share a common structural framework built around amphipathic disks
Source: J Cell Biol. 2025 Aug 7;224(9):e202411175. doi: 10.1083/jcb.202411175 (PMC12330381; doi:10.1083/jcb.202411175)

Figure legend.

1. The Western blotting results were scanned using the LI-COR Odyssey system. The 800 nm channel was used to capture signals from the target protein, while the 680 nm channel was used to detect the NativeMark™ Unstained Protein Standard (The membrane with molecular weight markers was cut and stained with Coomassie Brilliant Blue, the bands can be better detected in the 680nm channel).

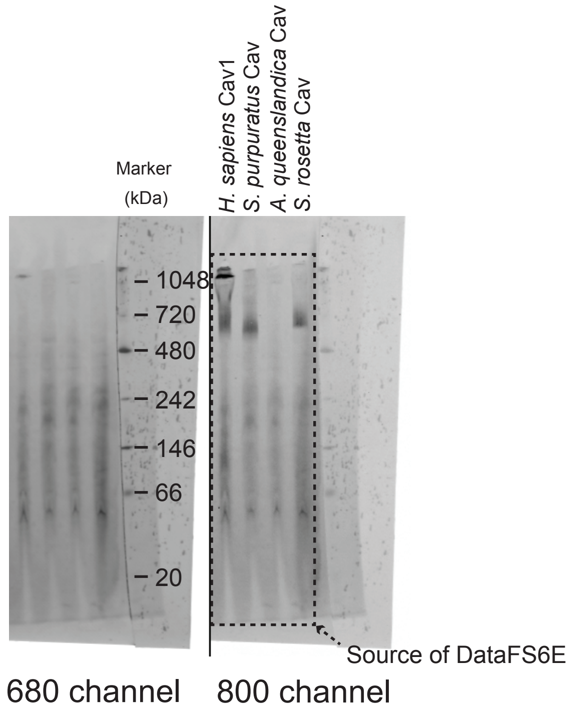

Supplement: SourceData F6 — is the source file for Fig. 6. [file jcb_202411175_sourcedataf6.pdf]
