# Supplementary material for: Evolutionarily diverse caveolins share a common structural framework built around amphipathic disks
Source: J Cell Biol. 2025 Aug 7;224(9):e202411175. doi: 10.1083/jcb.202411175 (PMC12330381; doi:10.1083/jcb.202411175)

Figure legend.

1. The Western blotting results were scanned using the LI-COR Odyssey system. The 800 nm channel was used to capture signals from the target protein, while the 680 nm channel was used to detect the molecular weight bands (191, 64, 51, 39, 28, 19, and 14 kDa) from the SeeBlue™ Plus2 Pre-stained Protein Standard. The 97 kDa band was visible in the 800 nm channel.
2. The samples Q03135 and A0A1X7UHP5 were analyzed on the same PFDV membrane (Membrane 1). Since A0A1X7UHP5 had a lower expression level, the 800 nm channel image was adjusted to a lower minimum threshold value using ImageJ during image analysis. As a result, the 800 nm channel results are presented twice for Membrane 1 in the figure below.
3. The samples F2U793 and A0A7M7T4C2 were analyzed on the same PFDV membrane (Membrane 2).
4. Abbreviations used in the labeling:
   - S40K: Soluble part after 40,000 rpm spin;
   - P40K: Pellet after 40,000 rpm spin;
   - FT: Flowthrough during nickel-beads purification;
   - Void: Void fraction from FPLC purification;
   - P1-n: Peaks from FPLC.

Membrane 1 (Q03135 and A0A1X7UHP5 fractions)

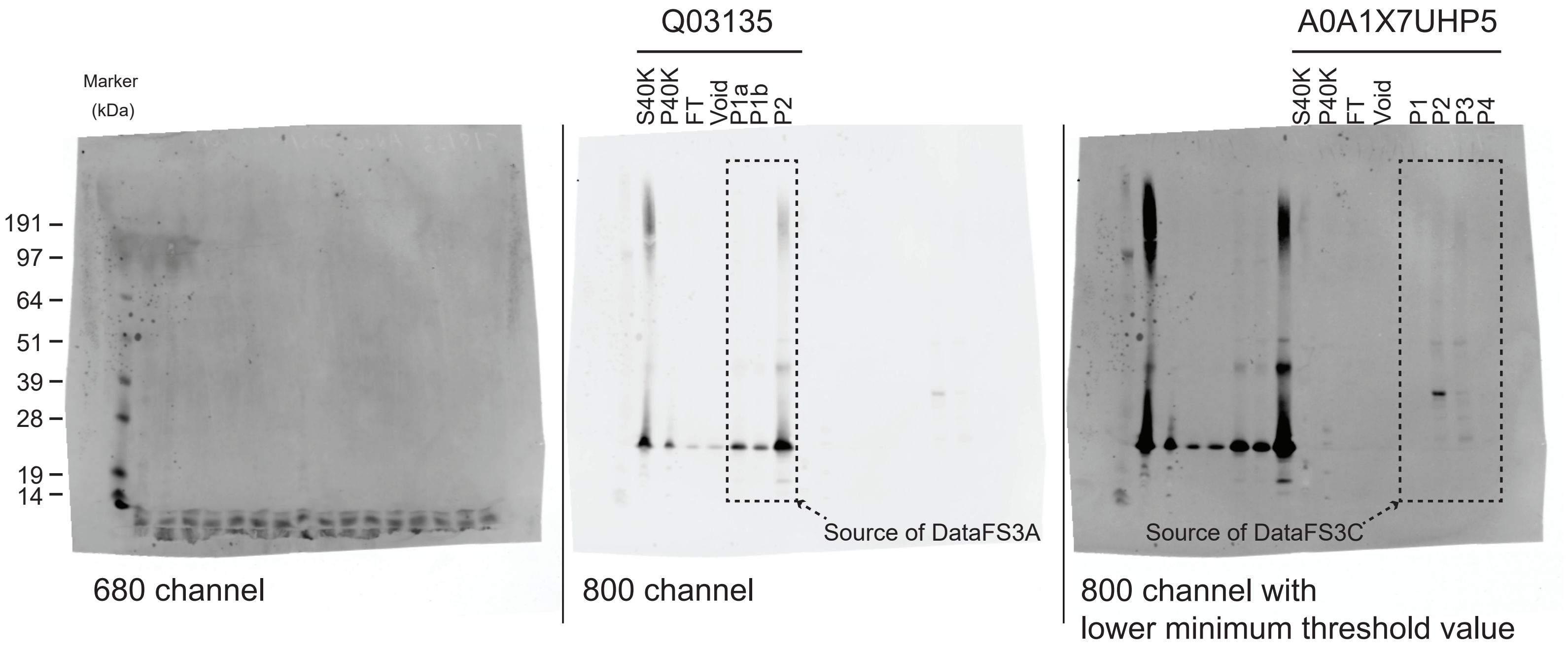

Membrane 2 (F2U793 and A0A7M7T4C2 fractions)

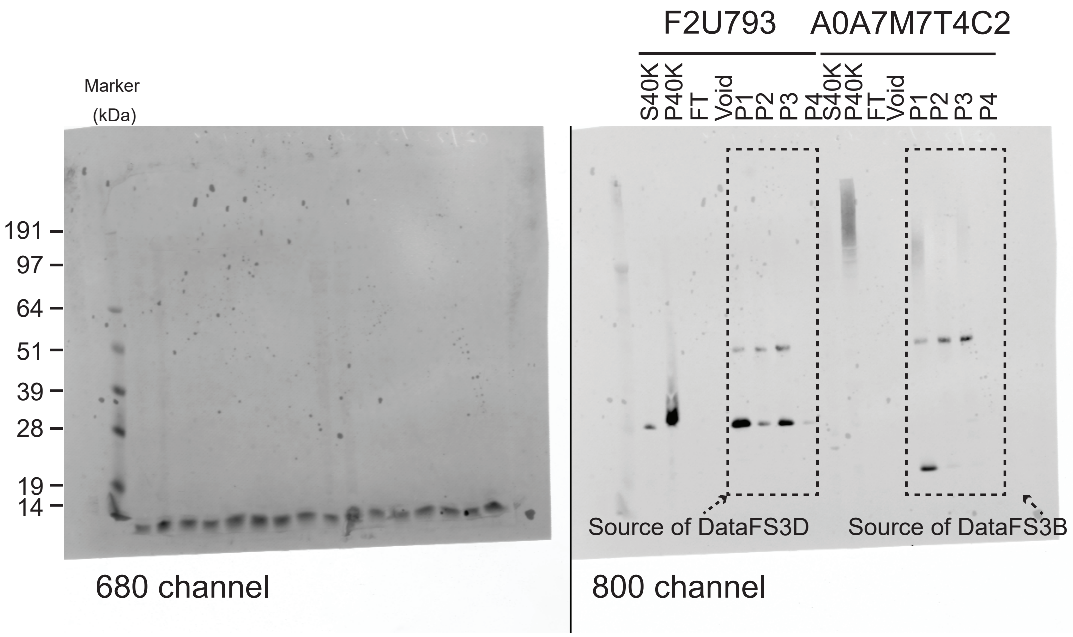

Supplement: SourceData FS4 — is the source file for Fig. S4. [file jcb_202411175_sourcedatafs4.pdf]
