# Supplementary material for: Evolutionarily diverse caveolins share a common structural framework built around amphipathic disks
Source: J Cell Biol. 2025 Aug 7;224(9):e202411175. doi: 10.1083/jcb.202411175 (PMC12330381; doi:10.1083/jcb.202411175)
Supplement: Data S2 — shows alignment of the caveolin sequences clustered in Fig. S1 and S2. [file jcb_202411175_datas2.pdf]

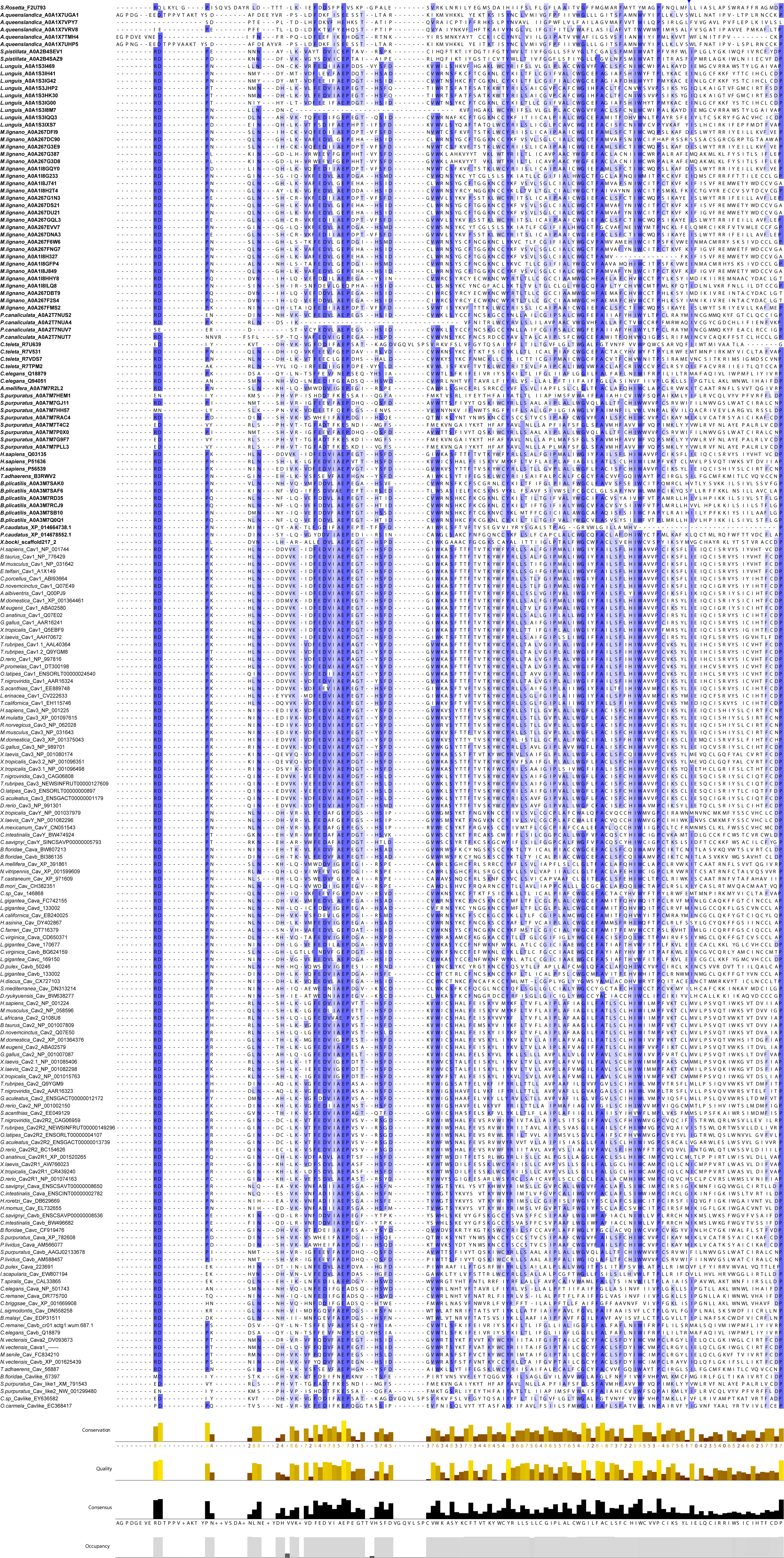
Conservation
Quality
Consensus
Occupancy
